# Design, Syntheses, and Pharmacological Evaluations of Core Ring Expanded Fentanyl Analogues as Potential Counteracting Agents Against Fentanyl Induced Respiratory Depression

**DOI:** 10.1021/acs.jmedchem.5c00528

**Published:** 2025-11-03

**Authors:** Abeje A. Silte, Ennian Li, Balaji S. Kale, Logan Neel, Neha Upadhyay, Rachael Flammia, Rui Lyu, Celsey M. St Onge, Ahmed Reda, Samuel Woodard, James C. Gillespie, Daniel Kim, Dana E. Selley, William L. Dewey, Piyusha P. Pagare, Yan Zhang

**Affiliations:** † Department of Medicinal Chemistry, School of Pharmacy, 6889 Virginia Commonwealth University, 800 East Leigh Street, Richmond, Virginia 23298, United States; ‡ Department of Pharmacology and Toxicology, School of Medicine, Virginia Commonwealth University, 410 North 12th Street, Richmond, Virginia 23298, United States; § Center for Drug Discovery, Virginia Commonwealth University, 800 East Leigh Street, Richmond, Virginia 23298, United States; ∥ Institute for Drug and Alcohol Studies, Virginia Commonwealth University, 203 East Cary Street, Richmond, Virginia 23298, United States

## Abstract

The escalating synthetic opioid crisis necessitates novel treatments, especially for fentanyl overdose. This study presents 84 ring-expanded fentanyl analogs, replacing its piperidine core with 4-azepane and 5-azocane structures. *In vivo* antagonism studies identified 15 compounds that effectively blocked synthetic opioid antinociception. Further dose–response analysis identified four potent antagonists (**16**, **46**, **53**, and **69**) against both fentanyl and morphine. Notably, Compound **53** demonstrated the highest potency with AD_50_ of 2.02 mg/kg against morphine and 4.02 mg/kg against fentanyl. Compound **53** exhibits a favorable pharmacokinetic profile, including moderate human metabolic stability, low efflux, and efficient, sustained CNS penetration, making it a promising centrally acting MOR antagonist candidate. Significantly, whole-body plethysmography confirmed that compound **53** reversed fentanyl-induced respiratory depression. These results suggest that expanding the core ring structure of fentanyl is a promising strategy to develop potent mu opioid receptor antagonists to inhibit both antinociception and respiratory depression offering potential solutions to fentanyl overdose.

## Graphical abstract

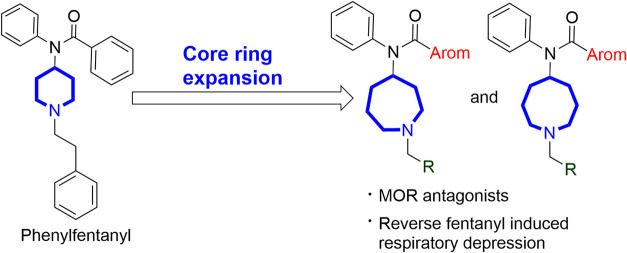

## Introduction

The opioid crisis in the United States has reached an alarming level. Since 2002, the number of overdose deaths involving opioids has surged by over 30%, driven initially by prescription opioids and heroin. More recently, the landscape has dramatically shifted with illicitly manufactured fentanyl and its analogs now the primary culprits.^1^ The pervasive abuse of opioids, coupled with their potent respiratory depressant effects, has fueled this devastating epidemic. In 2023, a staggering 107,543 individuals died from drug overdoses in the U.S., with 81,083 of these deaths attributed to opioids alone.^2^ This grim statistic is projected to escalate rapidly as the accessibility and consumption of illicit fentanyl and its analogs continue to expand, leading to heightened toxicity within the drug supply.^3^ According to the Centers for Disease Control and Prevention, fentanyl remains responsible for the most deaths in the USA,^4^ and the misuse of opioids, particularly in North America, raises serious concerns for public health officials due to the high rate of overdose deaths and the predicted 1–2 million deaths from opioid usage by 2029.^5^

Fentanyl, a synthetic opioid significantly more potent than heroin, poses a unique and grave threat in the USA.^4^ The distinct pharmacological properties of fentanyl, a synthetic opioid, significantly contribute to its elevated overdose risk. It has an exceptionally high affinity and intrinsic activity at the mu opioid receptor (MOR), making it more than 50 times more potent than heroin.^6,7^ Its high lipophilicity allows it to quickly cross lipid membranes, leading to rapid activation of the central MOR. This results in intense euphoria, respiratory depression, and other classic effects associated with MOR activation.^8–10^ High potency of fentanyl means that amounts as small as 2–3 mg of intravenous fentanyl can induce life-threatening respiratory depression in as short as 2 min, while heroin overdoses usually take at least 30 min to reach lethal levels.^11,12^ The fentanyl overdose deaths can occur very quickly, within 5 min, potentially before remedial action can be taken.^6^ A more significant concern is that tolerance to opioid-induced respiratory depression develops more slowly than tolerance to opioid-induced euphoria. This discrepancy increases the risk when doses are escalated or more potent opioids are used, as individuals may chase the euphoric effects without realizing the heightened danger of respiratory failure.^7^

The MOR mediates opioids’ analgesic effects and abuse liability.^13^ Buprenorphine, methadone, naloxone, and naltrexone are among the pharmacotherapies used to treat opioid use disorders (OUD) (Figure 1).^14,15^ Buprenorphine, a partial MOR agonist, and methadone, a full MOR agonist, are used to treat OUD through opioid replacement or detoxification therapy. Buprenorphine and the MOR antagonist naloxone (NLX) are combined in both the detoxification and maintenance phases of OUD treatment, while naltrexone (NTX, a MOR antagonist) is used to prevent relapse. NLX (Narcan) is the gold standard for treating an acute opioid overdose.^14,16^ NLX, however, cannot be used as a preventative measure, and the extended effects of fentanyl and more potent fentanyl analogues like carfentanil sometimes require repeated doses of NLX due to its short duration of action.^17,18^ Given the rapid onset of fentanyl-related toxicity and the fact that many overdoses occur in isolation, there is often a missed opportunity to administer NLX within the critical time window.^19^ Patients treated for opioid overdose are at a heightened risk for experiencing repeat overdoses.^20,21^ A significant concern is that NLX exhibits relatively low potency in reversing the respiratory depression associated with fentanyl overdoses. This is particularly troubling given that respiratory failure is recognized as the leading cause of death in cases of fentanyl overdose.^22–25^ Recently, the FDA approved nalmefene, a longer-acting NLX-like medication, to help address fentanyl and fentanyl analogues’ overdoses. Despite ongoing efforts to enhance the availability and awareness of NLX and nalmefene, the number of overdose deaths resulting from fentanyl and its agonist analogues continue to be a persistent national crisis.^26–29^ However, MOR antagonists, including NLX and nalmefene, have limited effectiveness in mitigating the dangerous fentanyl-induced respiratory depression, as this phenomenon may not be exclusively mediated through MOR signaling.^30,31^ This highlights the urgent need for innovative therapeutic strategies that can counteract the respiratory depression induced by fentanyl and its analogues.

**Figure 1:**
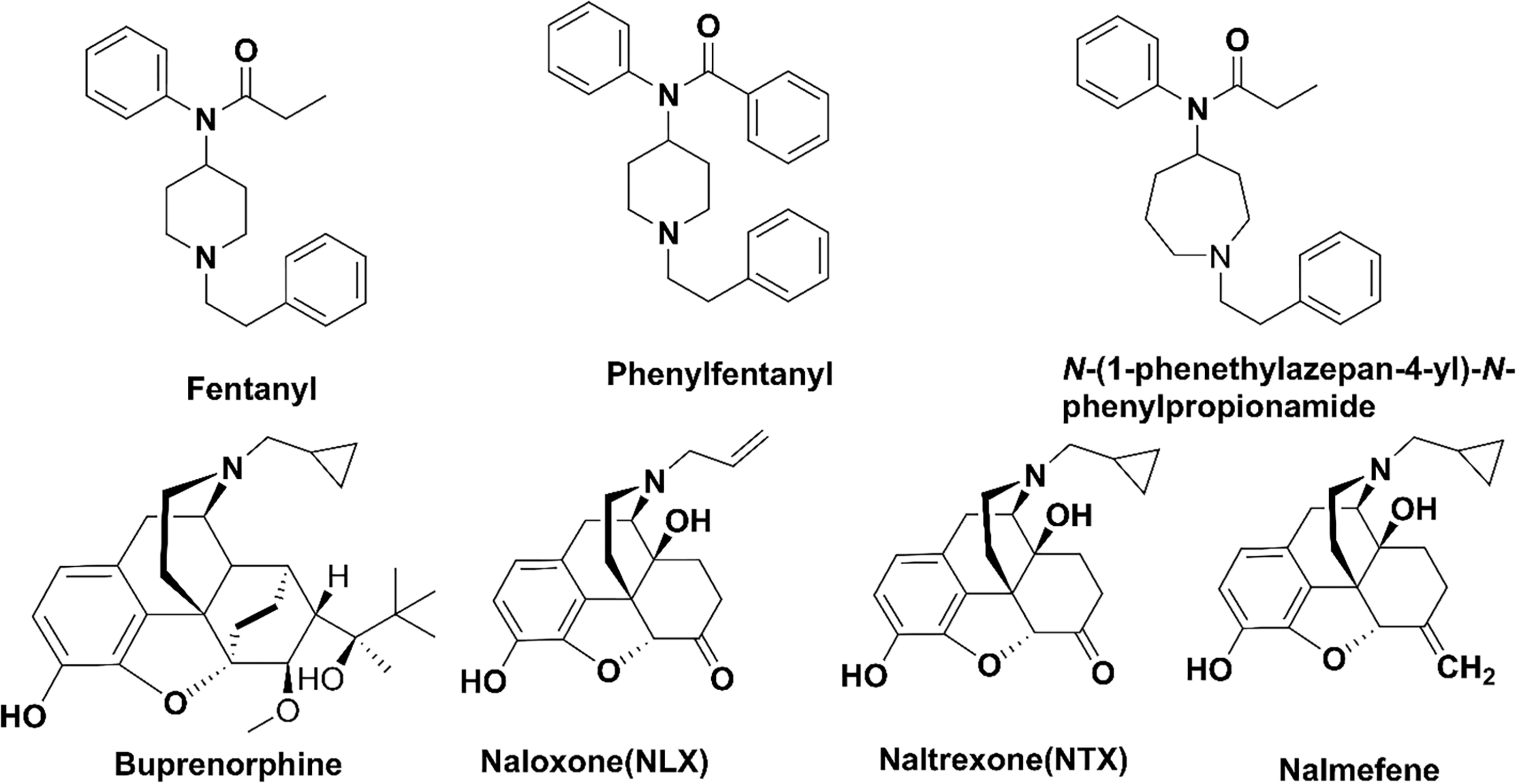
Chemical structures of fentanyl, phenylfentanyl, and FDA approved counteracting reagents against OUD.

Fentanyl, a 2-phenylethyl-substituted 4-anilinopiperidine derivative carrying a propionyl amide moiety linked to the aniline-nitrogen, a scaffold consisting of four modifiable moieties, has been modified since Janssen’s 1960 first disclosure^32^ to create structural features of fentanyl primarily seeking analgesics with superior pharmacokinetic properties, onset time, and effective dosage.^33,34^ As shown in Figure 2 the four fundamental modifiable moieties include: (a) the core piperidine ring, (b) the anilino phenyl ring, (c) the *N*-alkyl moiety, and (d) an acyl moiety linked to the anilino-nitrogen.^35–37^

**Figure 2:**
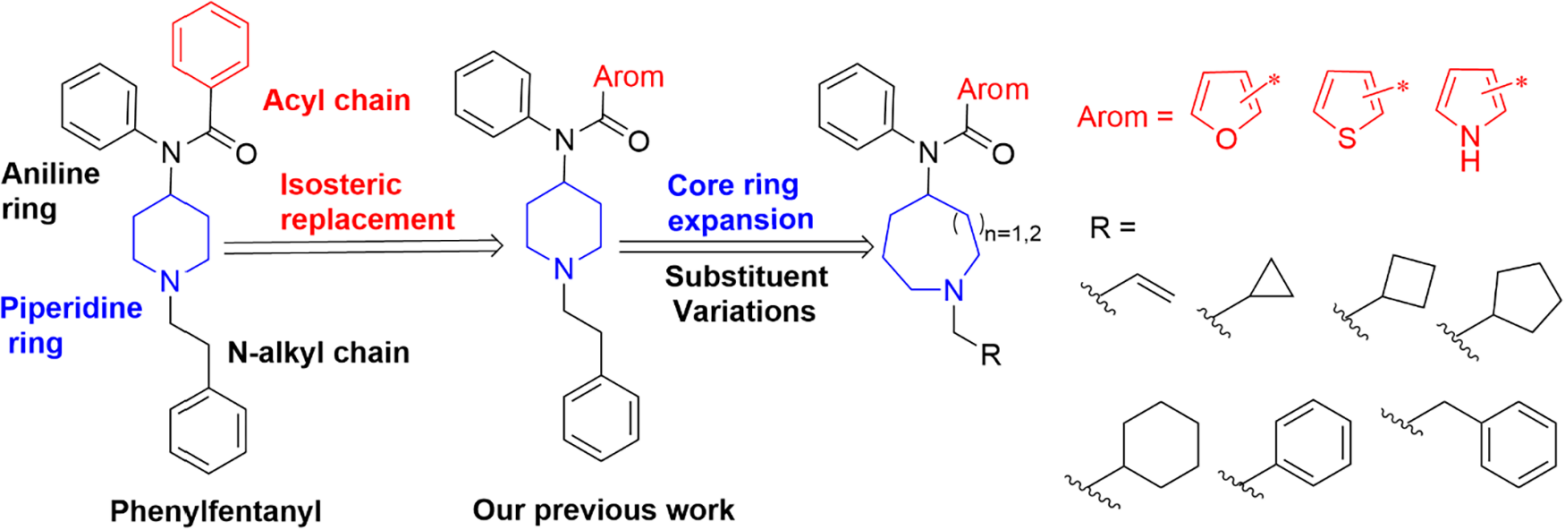
Molecular design.

Riley et al.^38–40^ reported a series of core ring-expanded fentanyl analogues of 4-phenylperhydroazepines while maintaining the acyl group and varying the *N*-alkyl substituents. These modifications resulted in analogues exhibiting lower agonistic activity at the opioid receptors as one shown in Figure 1. As demonstrated recently by Arita et al.^41^ exploring atropisomerism in the design of fentanyl-based MOR antagonists may yield compounds with improved pharmacological profile. And also it is reported that the presence of 2- or 3- furanyl substitution on the fentanyl core has been associated with a reduction in agonist efficacy and in some cases a transition to antagonistic properties at opioid receptors.^42^ Meanwhile, phenylfentanyl (Figure 1), with replacing ethyl by phenyl group on the acyl moiety, also showed a significant reduction agonistic activity at the MOR, with only 5.18 ± 0.70% *E*_max_ of DAMGO in functional assays.^43^ Those observations suggest that replacements on the acyl chain as well as core ring expansion on the fentanyl skeleton may lead to lower efficacy or even antagonism on the MOR. In this study, we decided to replace the acyl moiety with a hetero aromatic ring while further exploring both 4-azepane and 5-azocane core ring systems. Furthermore, we incorporated different substitutions with distinct bulkiness profiles, such as allyl, cyclopropyl, cyclobutyl, cyclopentyl, cyclohexyl, benzyl, and phenylethyl, to adjust the spatial orientation of the *N*-alkyl side chain, aiming to optimize binding affinity on the MOR and potentially lead to the development of novel and more potent antagonists.

## Results and Discussion

### Molecular Design

In our earlier work on phenylfentanyl and its analogues,^43^ phenylfentanyl acted as a neutral antagonist at the MOR while showing inhibitory effects to both fentanyl and DAMGO in the calcium flux assays. In this work, by expanding its core piperidine ring to larger ring systems, i.e., 4-azepane and 5-azocane, we will increase the conformational flexibility of the skeleton. Meanwhile, isosteric replacements of the phenyl ring with heteroaromatic rings like 2- and 3-substituted furan, thiophene, and pyrrole as previously reported, along with the ring expansion strategy may alter molecular interaction (Figure 2).

### Chemical Syntheses

Following the discovery of fentanyl,^44,45^ several synthetic pathways have been developed in order to gain access to fentanyl analogues that are varied in terms of their biological and structural composition.^31^ We explored and optimized the synthetic route demonstrated by Mayer and co-workers.^46^ Herein we efficiently synthesized two series of fentanyl analogs modified with a core ring expansion and various side chains as shown in Scheme 1. Briefly, commercially available *tert*-butyl-4-oxoazepane-1-carboxylate reacted with aniline mediated by sodium triacetoxyborohydride in the presence of acetic acid to give the desired intermediate *tert*-butyl-4-(phenylamino)-azepane-1-carboxylate **1a** with an excellent yield. Intermediate **1a** was then acylated using different acyl chlorides either purchased from vendors or prepared in-house in the presence of triethylamine, affording N-protected *tert*-butyl-4-(phenylcarboxamido)­azocane-1-carboxylate **2­(a**–**f)**. Subsequent deprotection of compounds **2a**–**f** was achieved using trifluoroacetic acid in dichloromethane, yielding intermediates that were carried forward to the next step without further purification. The intermediate from the deprotection step were then reacted directly with various alkyl or aryl halides under basic conditions in acetonitrile to yield free bases. To obtain the desired salt forms, these free bases were dissolved in a small amount of methanol and treated with hydrogen chloride in methanol, followed by quenching with diethyl ether to yield their hydrochloride salts (**4**–**45**). Similarly, compound **46**–**87** were obtained by following the above general procedure starting with the commercially available *tert*-butyl-5-methyleneazocane-1-carboxylate. The new 4-azepane derivatives possess a chiral center, however, the observed multiplicity and additional signals in their ^1^H NMR and ^13^C NMR spectra are indicative of diastereomer formation. The hypothesis for these results could be due to protonation of the nitrogen atom in the 7-membered ring introduces a second chiral center, giving rise to diastereomeric species. Furthermore, ^1^H NMR spectra reveal a predominant isomer, suggesting a favored configuration. A proposed rational for this configurational preference is illustrated in the Supporting Information as showed in Figure S1. As opioid receptors are capable of recognizing ligands in a stereospecific manner as such the S-enantiomer exerted antagonistic effect for the MOR, whereas the R-enantiomer exerted agonist effect.^41^ It is reasonable to anticipate that these stereoisomers will not possess equal potency. Further enantiomer separations would be pursued for the most potent compounds once identified.

**Scheme 1:**
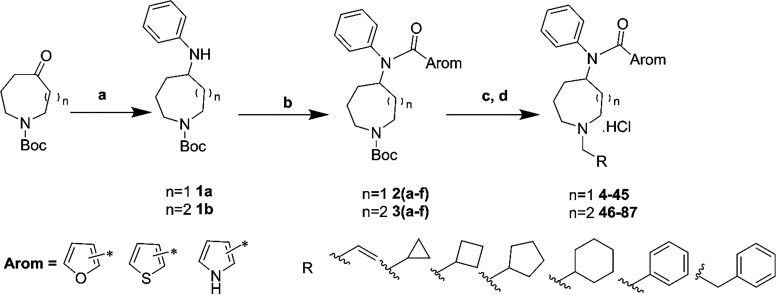
Synthesis of Core Ring Expanded Fentanyl Analogues Bearing Heterocyclic Rings^a^

a Reagents and conditions: (a) Aniline, AcOH, Na­(OAc)_3_BH, DCM, r.t.; (b) acyl chlorides either commercially available or prepared in-house, TEA, DCM, r.t.; (c) TFA, DCM r.t.; R-Br, K_2_CO_3_, CH_3_CN, 80 °C reflux; and (d) HCl/MeOH, Et_2_O.

A total of 84 (42 containing 4-azepane ring and 42 containing 5-azocane ring) target compounds were synthesized by alteration of acylating agents and alkylating agents. All the target compounds were subjected to full characterization via analytical approaches including ^1^H NMR, ^13^C NMR, MS, and the purities were determined by HPLC before advancing to pharmacological assessments.

### Warm Water Tail Immersion Assay Studies

The warm-water tail immersion pain model, a technique that quantifies the latency of tail withdrawal in mice immersed in warm water under varying doses of tested agents, has been extensively used in prior investigations to determine their acute agonistic or antagonistic properties.^47–49^ *In vivo* studies can reveal potential systemic side effects, toxicities, and interactions that would be missed in a more simplified *in vitro* setting. Once an effect is observed *in vivo*, *in vitro* studies can be used to delve deeper into the underlying mechanisms at the cellular and molecular level. Also due to the nation-wide shortage of radioligand supplies and our stringent timeline of the project, we decided to pursue warm water tail immersion assays first to understand how these compounds would behave at the whole animal model level. One of the advantages of such an approach is that the outcome from such a study will reflect both pharmacodynamic and pharmacokinetic properties of these new chemical entities. Conversely, warm water tail immersion assay disadvantages include a narrow scope that may not reflect peripheral, chronic, or neuropathic pain mechanisms.^50,51^ Furthermore, potential issues like stress-induced changes in pain threshold, habituation to repeated stimuli, and some degree of subjectivity in end point determination can introduce variability and necessitate careful experimental design.^52^ We have also recognized the safety and welfare of the animal subjects and optimized our protocol in order to minimize the number of animal subjects involved in our studies.

#### Single Dose Screening

First, all the synthesized compounds were assessed via previously outlined methodology^53^ of warm-water tail immersion experiments in Swiss Webster mice to evaluate their antinociceptive potential or their ability to block the antinociceptive effects of morphine or fentanyl. All 84 compounds, saline as a negative control and morphine and fentanyl as positive controls, were first administered at a single dose of 10 mg/kg subcutaneously. As shown in Figure 3A only compounds **6**, **7**, **13**, and **25** from the 4-azepane ring derivatives were identified showing significant antinociceptive effects compared to the vehicle while compound **68** (Figure 3B) could not be evaluated since it resulted in severe side effects at the dose of 10 mg/kg. All remaining compounds did not manifest obvious antinociception at 10 mg/kg and were subsequently studied for their ability to antagonize the antinociception effect of morphine (10 mg/kg, s.c.) and fentanyl (0.1 mg/kg, s.c.). Of interest all the compounds carrying the 5-azocane scaffold showing lower antinociceptive effects, which clearly highlighted the impact of core ring expansion to reduce opioid receptor activation.

**Figure 3:**
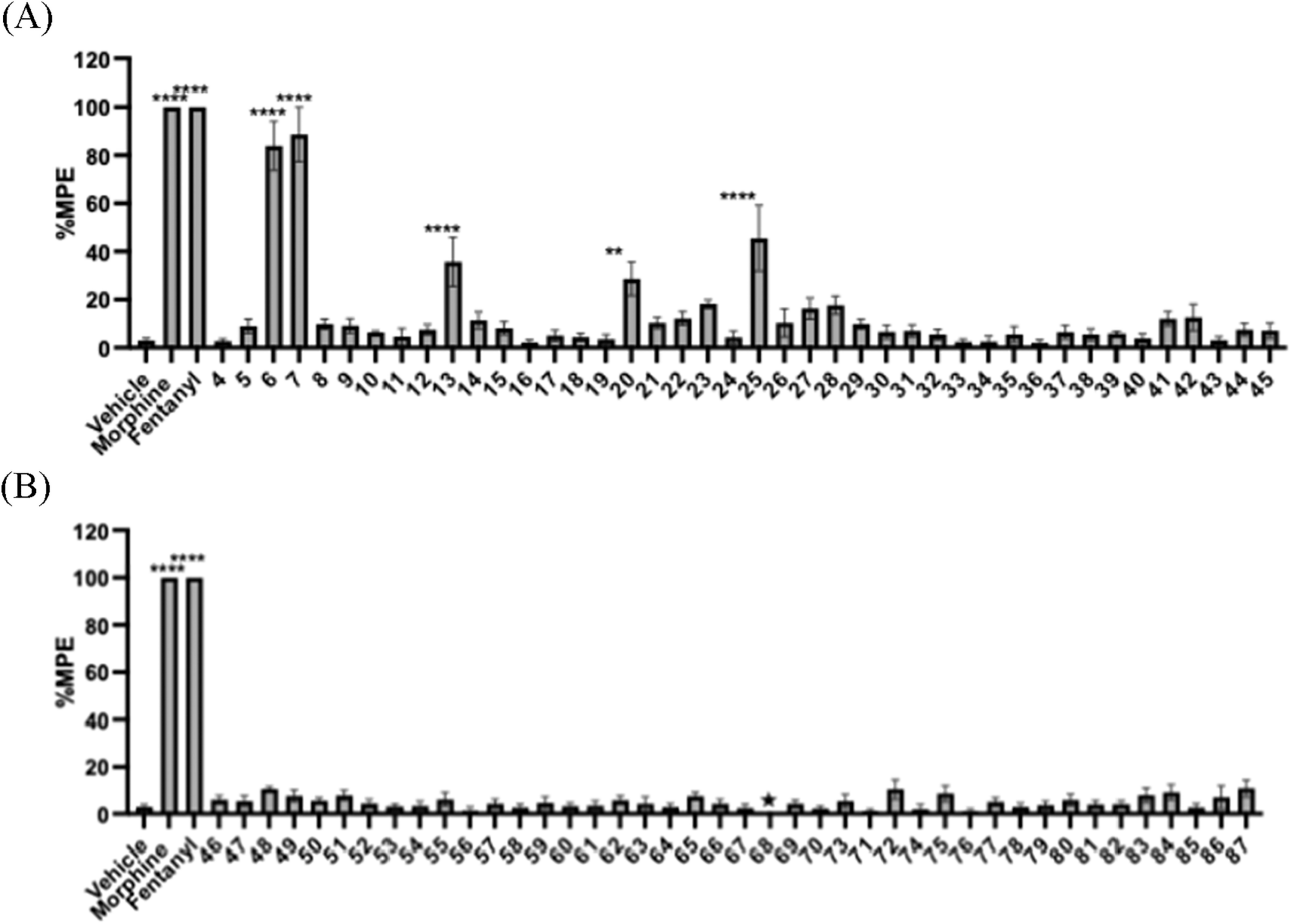
Warm-water tail immersion assay results of compounds (A) **4–45**, (B) **46–87** as agonists at a single dose of 10 mg/kg (s.c.). All compounds were administered subcutaneously (s.c.). Saline was used as the negative control while morphine 10 mg/kg (s.c.) and fentanyl 0.1 mg/kg (s.c.) were used as positive controls. Data are presented as mean values ± SD *P < 0.05, **P < 0.01, ***P < 0.0005, ***P < 0.0001, compared to vehicle (s.c.).

As shown in Figure 4, 15 out of 79 compounds i.e., compounds **10**, **16**, **37**, **40**, **43 46**, **47**, **48**, **53**, **54**, **55**, **63**, **69**, **70**, and **78** blocked the antinociception of morphine at a dose of 10 mg/kg. Of the 15 compounds, 5 compounds belonged to 4-azepane derivatives, and 10 compounds belonged to 5-azocane derivatives. Further study of these 15 compounds at a dose of 10 mg/kg to antagonize fentanyl’s antinociception resulted in 4 compounds, i.e., compound **16** (from 4-azepane derivative) (Figure 5A) and compounds **46**, **53**, and **69** from 5-azocane derivatives (Figure 5B) identified as the most potent candidates for further characterizations.

**Figure 4:**
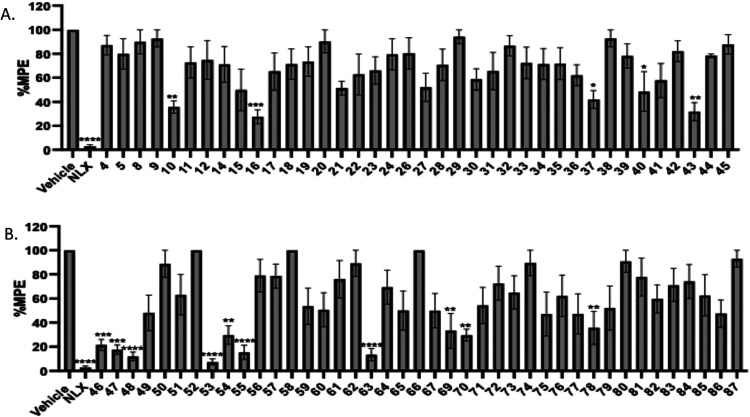
Warm-water tail immersion assay results of (A) **4–45**, (B) **46–87** compounds as antagonists at a single dose of 10 mg/kg (s.c.) in the presence of morphine (10 mg/kg, s.c.). Saline was used as the negative control and naloxone was used as a positive control. Data are presented as mean values ± SD *P < 0.01, ****P < 0.0001, compared to vehicle (s.c.).

**Figure 5:**
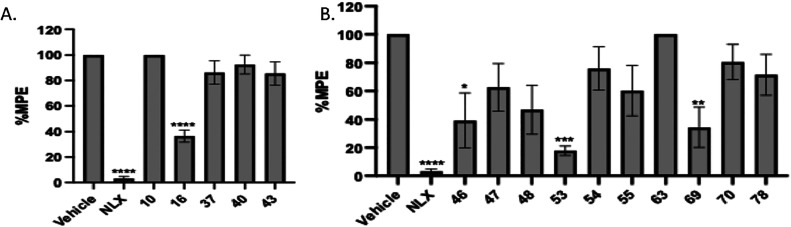
Warm-water tail immersion assay results of selected compounds 15 compounds (A) 4-azepane ring derivatives (**10**, **16**, **37**, **40**, and **43**), (B) 5-azocane ring derivatives (**46**, **47**, **48**, **53**, **54**, **55**, **63**, **69**, **70**, and **78**) as antagonists at a single dose of 10 mg/kg (s.c.) in the presence of fentanyl (0.1 mg/kg, s.c.). Saline was used as the control and data are presented as mean values ± SD *P < 0.01, ****P < 0.0001, compared to vehicle (s.c.).

#### Dose Response Studies

Following *in vivo* single dose screening of all compounds, dose response studies were conducted for the identified four compounds. As shown in Figure 6 and summarized in Table 1, the dose response studies of the most potent derivatives **16, 46, 53**, and **69** when tested against 10 mg/kg of morphine showed an AD_50_ = 6.39, 2.89, 2.02, and 4.04 mg/kg, respectively. When tested against 0.1 mg/kg fentanyl these compounds showed an AD_50_ = 9.81, 6.89, 4.02, and 8.14 mg/kg, respectively. The potency of compound **53** against morphine (10 mg/kg) is 2-fold higher compared to against fentanyl (0.1 mg/kg). However, when directly compared to naloxone, a well-established opioid antagonist, the observed antagonistic potency of our compounds was notably lower (Table 1).

**Figure 6:**
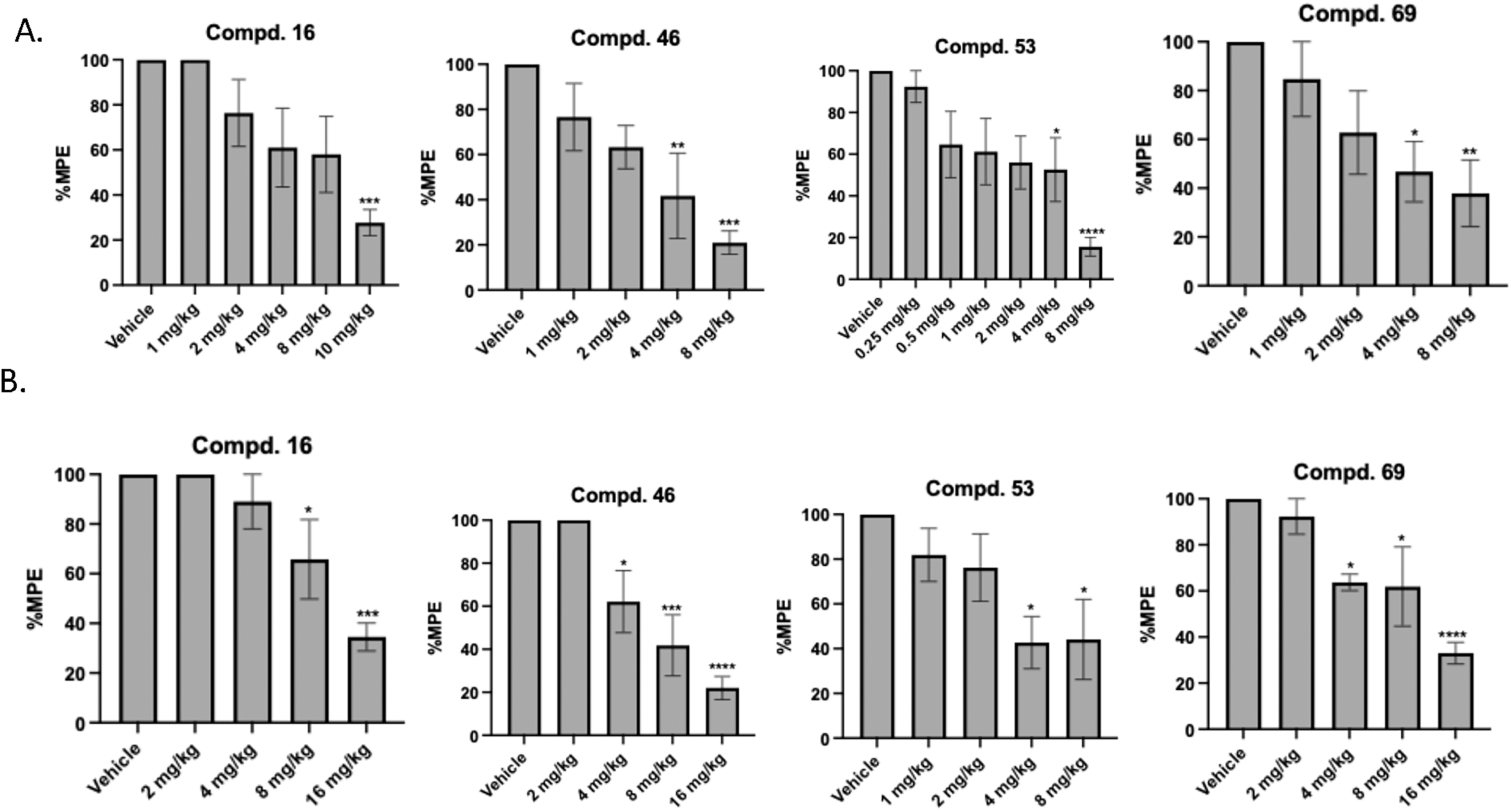
Dose response study of most potent derivatives (**16**, **46**, **53**, and **69**) against (A) 10 mg/kg morphine, s.c. (B) 0.1 mg/kg fentanyl was administered (s.c.). Tail withdrawal latencies were measured 20 min post morphine or fentanyl administration. Data are presented as mean values ± SD.

**Table 1:** Potency of Compounds Antagonize Morphine and Fentanyl-Mediated Antinociception

	AD_50_ mg/kg (95% CL)	
compound	against 10 mg/kg morphine	against 0.1 mg/kg fentanyl
NLX^a^	0.05 (0.03–0.09)	0.005 (0.002–0.009)
16	6.39 (3.51–11.62)	9.81 (6.31–15.23)
46	2.89 (2.28–3.67)	6.89 (5.45–8.72)
53	2.02 (1.89–3.44)	4.02 (2.20–7.33)
69	4.04 (2.58–6.34)	8.14 (4.99–13.27)

a Data have been reported in refs 47 and 50 are provided here for comparative analysis.

### *In Vitro* Characterization

We then conducted a number of *in vitro* studies to better understand the mechanism of actions underlying the *in vivo* effects of our potent compounds. By eliminating the intricacies of the entire organism, these *in vitro* investigations yielded vital information about the compounds’ cellular actions, allowing us to identify the targets and pathways responsible for the observed *in vivo* effects and improve our comprehension of their potential as a treatment.

#### Calcium Mobilization Assay

The calcium mobilization assay is a technique used to measure changes in intracellular calcium levels, which are key indicators of cellular signaling, especially in response to activation of G-protein-coupled receptors (GPCRs). It typically utilizes fluorescent dyes that bind to the intracellular calcium, producing a measurable signal in response to receptor activation. To study the calcium-dependent signaling from the Gi/o-coupled MOR, a chimeric Gqi4 protein was coexpressed in monoclonal CHO cells, allowing detection of both agonist-induced calcium flux and antagonist-induced inhibition.^54^ To further characterize the antagonistic potential of the four compounds of interest, intracellular calcium mobilization assays in mMOR-CHO cells was conducted using previously reported procedure.^53^ Here, the compounds were tested for their potency to antagonize the effects of the MOR agonists DAMGO and fentanyl, at their respective EC_80_ concentrations (Table 2). While none of them showed any apparent effect to induce calcium flux themselves (Figure S2). In detail, as shown in Figure S2, all four compounds antagonized DAMGO with potency in the range of micromolar while compound **69** as the most potent one with an IC_50_ of 10.9 ± 2.3 μM and compound **16**, **46**, and **53** demonstrated their antagonistic effects with an IC_50_ values of 44.1 ± 2.7, 31.3 ± 6.7, and 33.6 ± 6.0 μM respectively. Similarly, all four compounds antagonized the Ca-flux induced by fentanyl potently, from which compound **16** (IC_50_ 1.12 ± 0.31 μM), showed an 18 times higher potency as compared to phenylfentanyl (IC_50_ 20.4 ± 1.3 μM), and compounds **53** and **69** with IC_50_ values of 4.51 ± 0.08 and 3.18 ± 0.19 μM showed 5- and 6-times higher potency respectively compared to phenylfentanyl. Interestingly, compounds **16**, **46, 53**, and **69** appeared to antagonize fentanyl with a higher potency compared to that seen against DAMGO, which indicating their chemical entity selectivity to fentanyl over DAMGO. The overall profile identified for compounds **16**, **46**, **53**, and **69** in the *in vitro* functional studies was found to be concordant with the results obtained from the *in vivo* tail immersion assays. Albeit, all the compounds were less potent compared to naltrexone (NTX). All results are summarized in Table 2.

**Table 2:** Calcium Mobilization Assay Data

compounds	inhibition of calcium flux induced by DAMGO IC_50_ (μM)^a^	inhibition of calcium flux induced by Fentanyl IC_50_ (μM)^a^
NTX	0.021 ± 0.015	0.007 ± 0.001
Phenylfentanyl^b^	4.10 ± 0.60	20.4 ± 1.3
16	44.1 ± 2.7	1.12 ± 0.31
46	31.3 ± 6.7	17.4 ± 12.5
53	33.6 ± 6.0	4.51 ± 0.08
69	10.9 ± 2.3	3.18 ± 0.19

a Assay was performed in triplicates against DAMGO (500 nM), Fentanyl (250 nM).

b Data cited from ref 43.

#### Radioligand Binding and [^35^S]-GTPγS Functional Studies

Subsequently, we characterized these compounds **16**, **46**, **53**, and **69** using both radioligand competition binding assays at the mu-opioid receptor (MOR), kappa-opioid receptor (KOR), and delta-opioid receptor (DOR) and [^35^S]-GTPγS functional assays on the MOR once the reagents were made available. These experiments aimed to determine their binding affinities at all three opioid receptor subtypes and to evaluate their functional potencies and efficacies specifically at the MOR.^47,55,56^

As shown in Table 3, the radioligand binding data of all the four most potent compounds suggested that these compounds carried high binding affinities at the MOR with much lower binding affinities to the KOR and DOR. Regarding their functional activities on the MOR, all four compounds showed double-digit nanomolar potencies with low or no efficacy on the MOR which matched well with their calcium flux potency profiles. Compared with phenylfentanyl, all four compounds showed reasonably binding affinity and selectivity with isosteric replacement of the phenyl group with its counterpart’s furan, thiophene, and pyrrole rings. Furthermore, for compounds bearing identical heteroaromatic groups (**46** and **53**) in the amide portion, the attachment points (2′ or 3′) on the heteroaromatic ring, along with similar substitution patterns, appeared to exert similar effects on both binding affinity and selectivity.

**Table 3:**
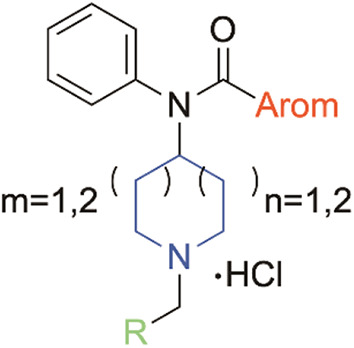

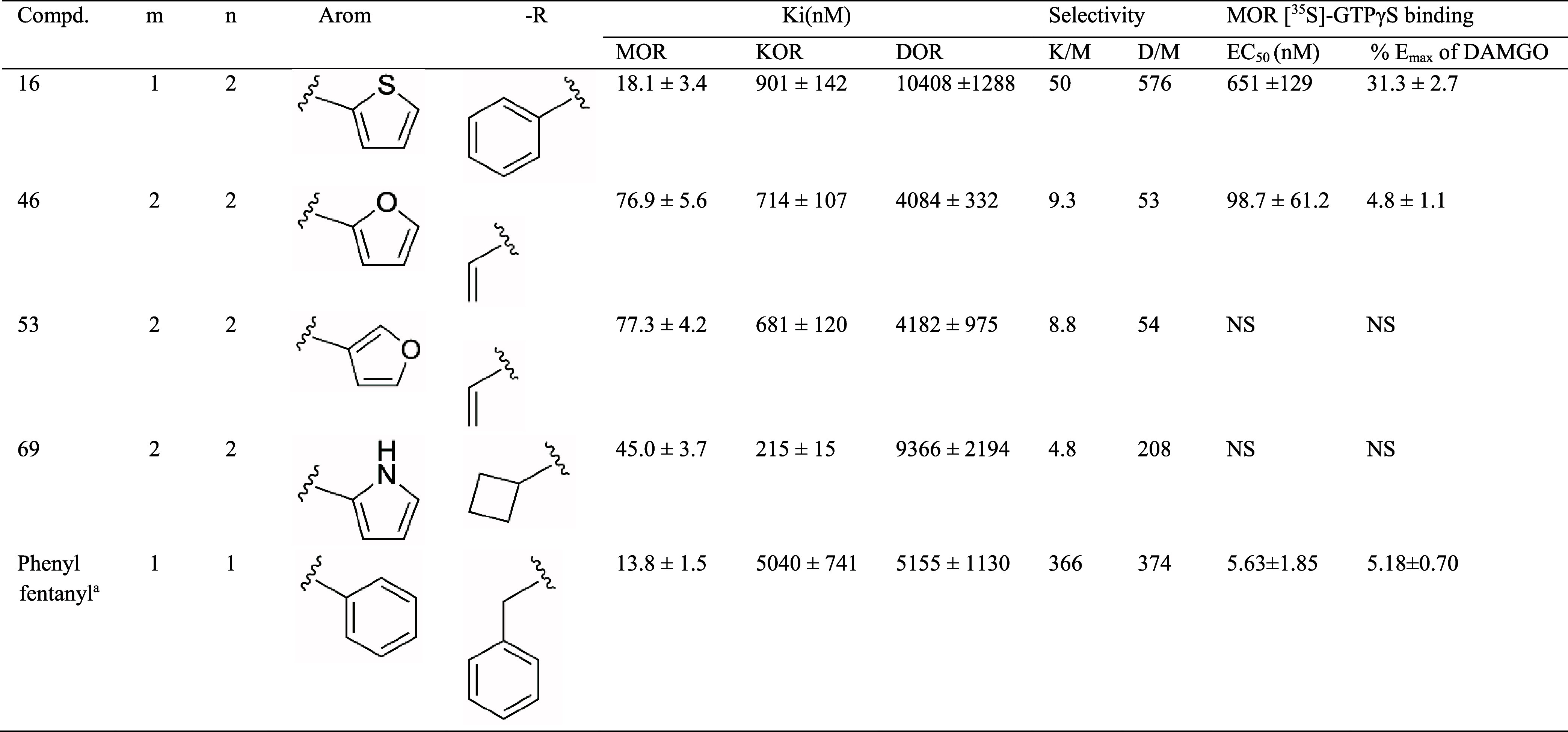
Opioid Receptor Binding Affinity and MOR [^35^S]-GTPγS Functional Assay Results for Ring-Expanded

a Data cited from ref 43. NS, no stimulation up to 30 μM.

### Respiratory Depression Studies of Compound **53**

#### *In Vivo* Whole-Body Plethysmography Study

Respiratory depression is a major cause of opioid overdose deaths.^12^ Fentanyl and its agonist equivalents cause respiratory depression by reducing the response to raised and lowered pCO_2_ levels.^59^ This reduces the impulse to breathe. Reduced respiratory drive causes slower breathing and apnea, which can be fatal in severe cases.^60,61^

Opioid-induced respiratory depression (OIRD) in mice was evaluated using whole-body plethysmography (WBP).^62–64^ This technique measures key respiratory parameters, including respiratory rate, tidal volume, and minute volume. Following a 20 min baseline period, mice received a subcutaneous injection of 0.3 mg/kg fentanyl. Respiratory parameters were then continuously monitored for 20 min.

As showed in Table 3, compounds **53** and **69** did not show any apparent agonistic activity at the MOR, whereas in Table 1, **53** exhibited two times higher potency compared to **69** against both morphine and fentanyl. In Table 2, **53** showed a reasonable inhibition effect of the calcium flux induced by DAMGO. Collectively, **53** was chosen to study the counteracting effect on the fentanyl-induced respiratory depression. When administered **53** alone at a dose of 32 mg/kg, did not induce respiratory depression but rather increased in both minute volume and respiratory rate (Figure S2). More significantly, compound **53** demonstrated a potent reversal effect on fentanyl-induced respiratory depression at both 10 and 32 mg/kg doses (Figure 7A). This beneficial effect was observed within a short time frame, appearing 10 min postadministration for the 32 mg/kg dose and 15 min postadministration for the 10 mg/kg dose. Importantly, this reversal effect proved to be sustained throughout the entire 35 min duration of the experiment. Mechanistically, this reversal action appears to stem from a combined effect on both respiratory frequency (Figure 7B) and tidal volume (Figure 7C), suggesting a multifaceted mechanism of action for compound **53** in mitigating fentanyl-induced respiratory depression. When compared with naloxone, the respiratory rate, tidal volume and minute volume of compound **53** at 10 and 32 mg/kg were almost similar after 20 min.

**Figure 7:**
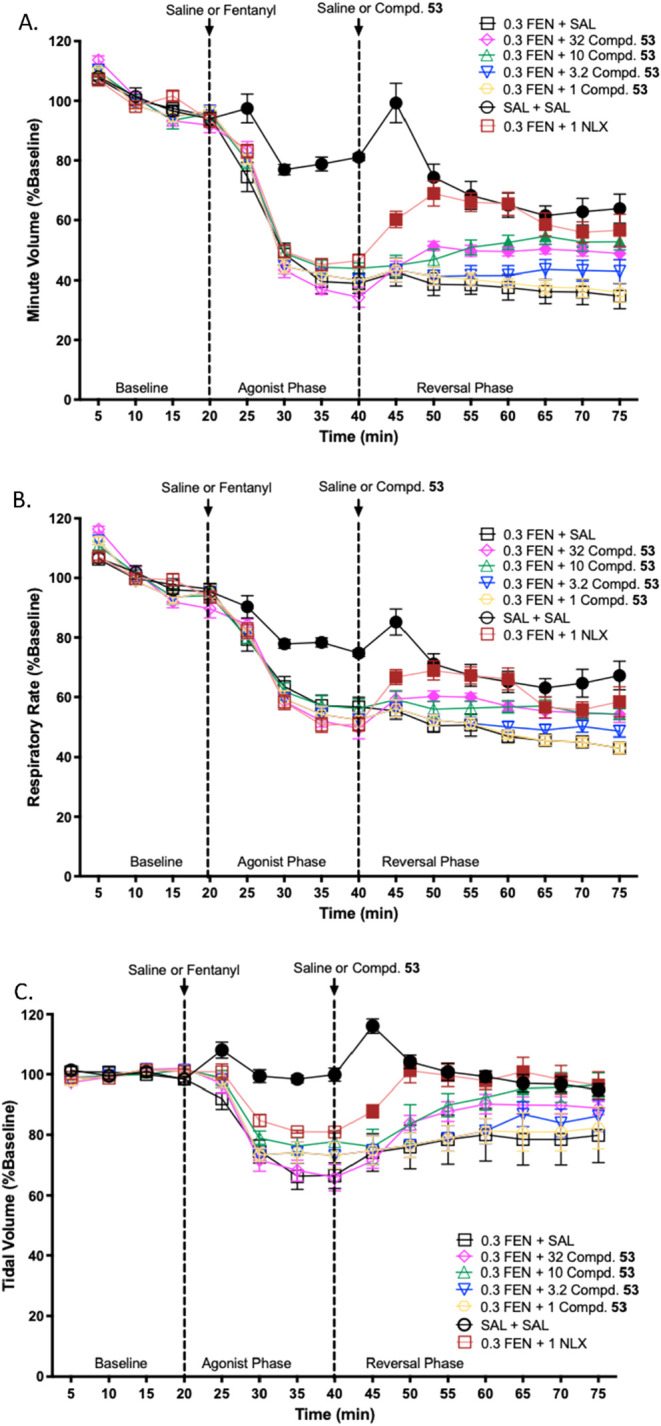
Effects of Compound **53** on fentanyl induced respiratory depression in mice. (A) Minute volume; (B) Respiratory rate and (C) Tidal volume. Error bars represent the standard error of normalized mean values within individual 5 min bins. Closed symbols indicate significant differences compared to the fentanyl (0.3 FEN + SAL)-treated controls at individual time points (*p* ≤ 0.05) via one-way ANOVA.

### *In Vivo* BBB Penetration Studies

*In vivo* time-dependent BBB-penetration studies were carried out to determine the CNS permeability of compound **53**. Compound **53** was administered s.c. at a dose of 10 mg/kg following which mice were sacrificed at different time points, and their plasma and blood samples were collected. After the blood samples were centrifuged to obtain plasma, the plasma and brain homogenate samples were analyzed to determine the amount of compound using liquid chromatography–tandem mass spectrometry (LC–MS/MS), and the brain-to-plasma ratios were calculated (Table 4). Compound **53** appeared in plasma with a concentration of 0.58 μg/mL as early as 5 min after administration, reaching the highest plasma concentration (0.97 μg/mL) at 10 min. Brain concentrations of compound **53** at 5, 10, 30, and 60 min were 0.48, 0.72, 0.64, and 0.82 μg/g, respectively, indicating that the compound readily penetrated into the CNS following administration. Notably, although plasma concentrations declined after 10 min, brain concentrations were relatively maintained, leading to a progressive rise in the brain-to-plasma concentration ratio over time. The highest brain-to-plasma ratio (1.24) was observed at 60 min, suggesting preferential accumulation or sustained retention of the compound in the brain relative to plasma (Table 4). These findings indicate that compound **53** efficiently crossed the blood–brain barrier and achieved sustained CNS exposure.

**Table 4:** Time Dependent BBB Penetration of Compd. **53** (10 mg/kg s.c.) in Mice (*n* = 3, Mean ± SD)

time (min)	5	10	30	60
brain (μg/g)	0.48 ± 0.05	0.72 ± 0.06	0.64 ± 0.16	0.82 ± 0.29
plasma (μg/mL)	0.58 ± 0.13	0.97 ± 0.09	0.80 ± 0.06	0.66 ± 0.14
brain-to-plasma ratio	0.82	0.74	0.80	1.24

### *In Vitro* Metabolism and Permeability

Evaluation of the metabolic stability of compound **53** was conducted using both human and mouse (CD-1) liver S9 fractions, with clozapine, diclofenac, estrone, and terfenadine included as reference compounds.^57^ As shown in Table 5, compound **53** demonstrated moderate metabolic stability in human liver S9, exhibiting a half-life (*t*_1/2_) of 26.3 min and an intrinsic clearance (CL_int_) of 27.1 μL/min/mg. A marked difference in metabolic stability was observed in mouse liver S9, where compound **53** displayed a considerably shorter half-life (*t*_1/2_ = 16.4 min) and a higher intrinsic clearance (CL_int_ = 42.3 μL/min/mg), indicating more rapid metabolic turnover within the rodent system. These findings reveal significant species-dependent differences in hepatic metabolism that are likely to influence systemic exposure, impact dosing frequency considerations, and be critical for the accuracy of translational pharmacokinetic predictions throughout preclinical development.

**Table 5:** *In Vitro* Metabolism Profile of Compound **53** and Control Compound^a^

compd.	*T*_1/2_-human (min)	CL_int_-human (μL/min/mg)	*T*_1/2_-mouse (min)	CL_int_-mouse (μL/min/mg)
**53**	26.3	27.1	16.4	42.3
clozapine	98.1	7.1	45.4	15.3
diclofenac	30.9	22.5	81.2	8.5
estrone	61.4	11.3	22.3	31.3
terfenadine	77.0	9.0	38.6	18.1

a Liver S9 fractions were used for both human and mouse (CD-1) determination.

Bidirectional transport assays in Caco-2 cells were conducted to determine the permeability and efflux potential of compound **53**. Efflux ratios were calculated with and without the presence of transporter inhibitors (verapamil for P-gp, KO143 for BCRP).^58^ Colchicine and estrone sulfate served as positive controls for P-gp and BCRP, respectively. As shown in Table 6, compound **53** demonstrated a low efflux potential, with efflux ratios of 0.9 for both P-gp and BCRP. These ratios showed a slight reduction to 0.7 with verapamil and 0.5 with KO143. The assay system’s integrity and sensitivity were confirmed by the high, inhibitor-sensitive efflux ratios observed for the positive controls, colchicine and estrone sulfate.

**Table 6:** *In Vitro* Absorption Profile of Compound **53** in Caco-2 Cells

compd.	substrate	efflux ratio	efflux ratio + inhibitor^a^
**53**	P-gp	0.9	0.7
colchicine		67.4	7.1
**53**	BCRP	0.9	0.5
estrone sulfate		33.6	2.6

a Verapamil used as P-gp inhibitor, KO143 used as BCRP inhibitor.

The consistently low efflux ratios observed for compound **53**, both in the absence and presence of transporter inhibitors, suggest minimal interaction with the major efflux transporters P-gp and BCRP. This favorable transporter profile not only reduces the risk of transporter-mediated drug–drug interactions but also suggests a higher likelihood of central nervous system (CNS) penetration, which is an essential property for a centrally acting MOR antagonist intended to counteract fentanyl-induced respiratory depression.

### Molecular Docking Studies of Fentanyl and Compound **53**

In order to rationalize the biological implications of our *in vitro* findings and gain a deeper understanding of how the 5-azocane ring scaffold influences binding interactions at the MOR, we conducted molecular docking studies. These computational investigations aimed to elucidate the binding pose and interactions of our novel 5-azocane-based ligands within the MOR active site. Initially, compound **53** was docked into the inactive MOR receptor (PDB ID: 4DKL)^65^ for 1000 genetic algorithm run. The docking solutions were ranked using the CHEM-PLP scoring function and subsequently analyzed. The results demonstrated that compound **53** formed stable interactions within the binding site of the MOR throughout the docking solutions (Figure 8). Specifically, compound **53** was shown to form interactions with residues such as D147^3.32^, Y148^3.33^, M151^3.36^, H297^8.52^, and V236^5.42^ (Ballesteros-Weinstein numbering)^66^ These interactions align with those observed for the antagonist β-funaltrexamine (β-FNA) and other epoxymorphinan antagonists, suggesting a comparable binding mode (Figure 9).^67–69^ On the other hand, compared to other known opioid antagonists, compound **53** did not interact with the allosteric binding site of the inactive MOR located between transmembrane helices TM5 and TM6.^70,71^ However, the furan oxygen of compound **53** was shown to form stronger hydrogen bond interactions with H297^8.52^, compared to typical epoxymorphinan antagonists, which rely on water-mediated hydrogen bonds with this residue through their phenolic groups. These highlights key difference in their binding profiles (Figure 8). In comparison to fentanyl, compound **53** showed a distinct interaction profile. It did not form, or only formed weak interactions, with transmembrane segments TM2 and TM3, which are critical for the agonistic effect of fentanyl at the MOR. Key residues such as W133^ECL1^, Q124^2.60^, and I144^3.29^ contributed significantly to the binding of fentanyl were either absent or weakly engaged by compound **53**.^72^ This difference in the interaction explains the divergent biological profiles of compound **53** and fentanyl (Figure 10).^72^

**Figure 8:**
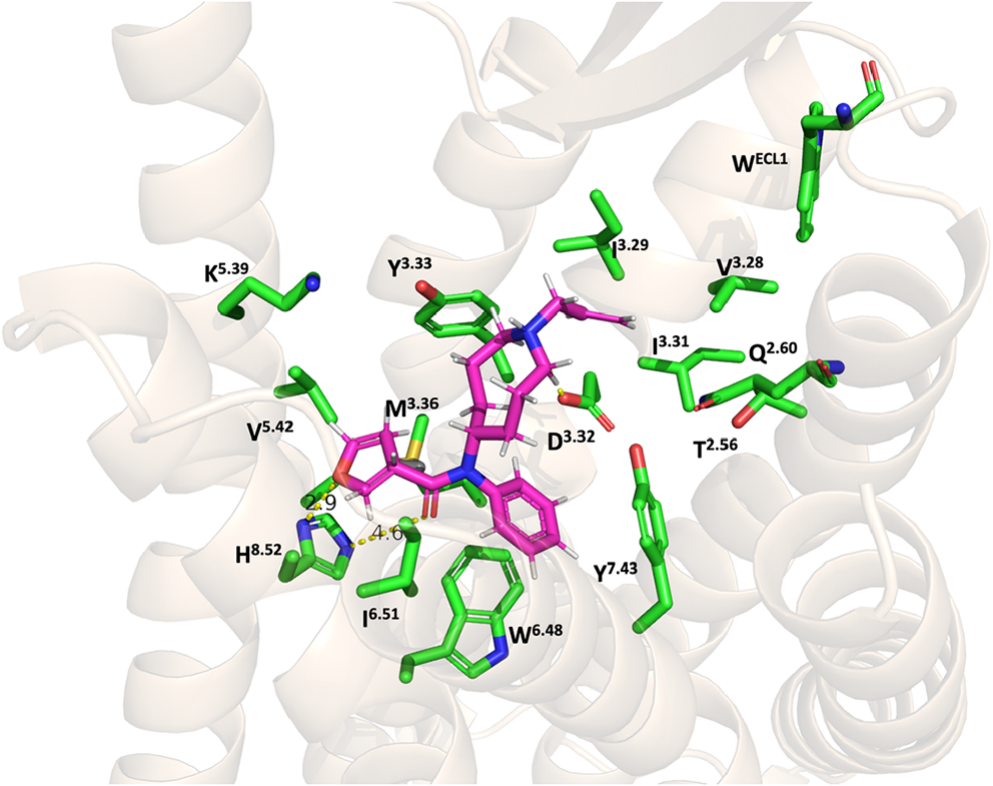
Binding pose of compound **53** in the inactive MOR (PDB: 4DKL) from docking study. MOR was shown as cartoon. Compound **53** (magenta sticks) and key amino acids (green sticks) are shown as stick models.

**Figure 9:**
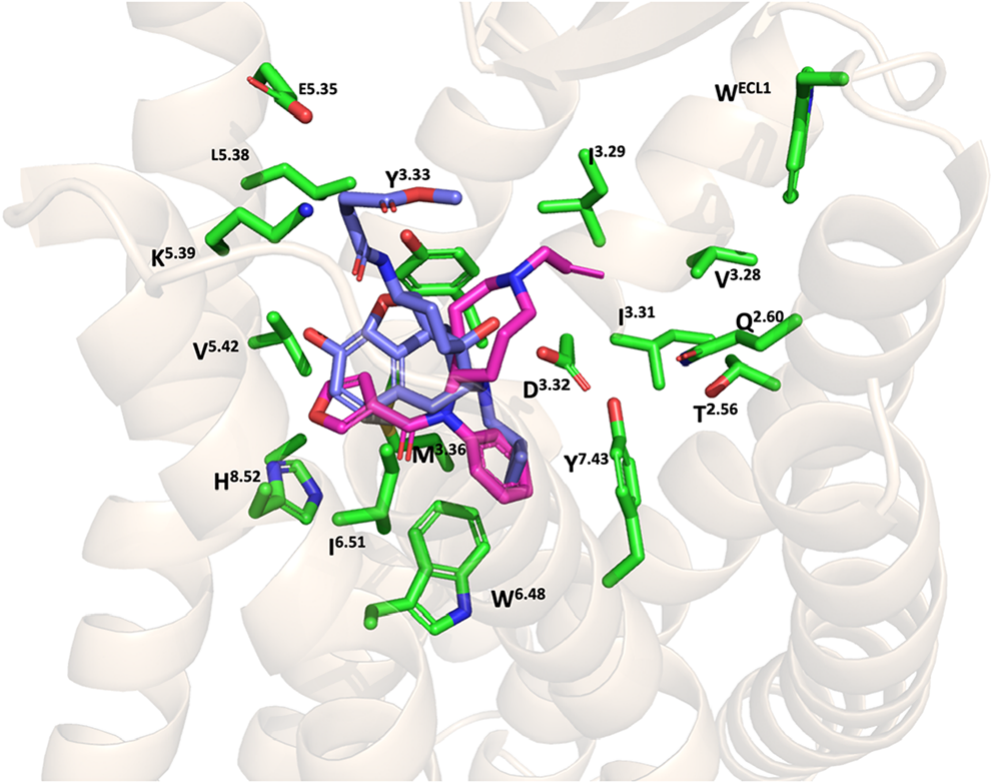
Alignment of the binding pose of compound **53** and β-FNA at the inactive MOR (4DKL). The MOR is shown as cartoons. Compound **53**, β-FNA and key amino acid residues are shown on the sticks. Carbon atoms: Compound **53** (magenta) β-FNA (blue); key amino residues for (green); oxygen atoms (red); nitrogen atoms (blue).

**Figure 10:**
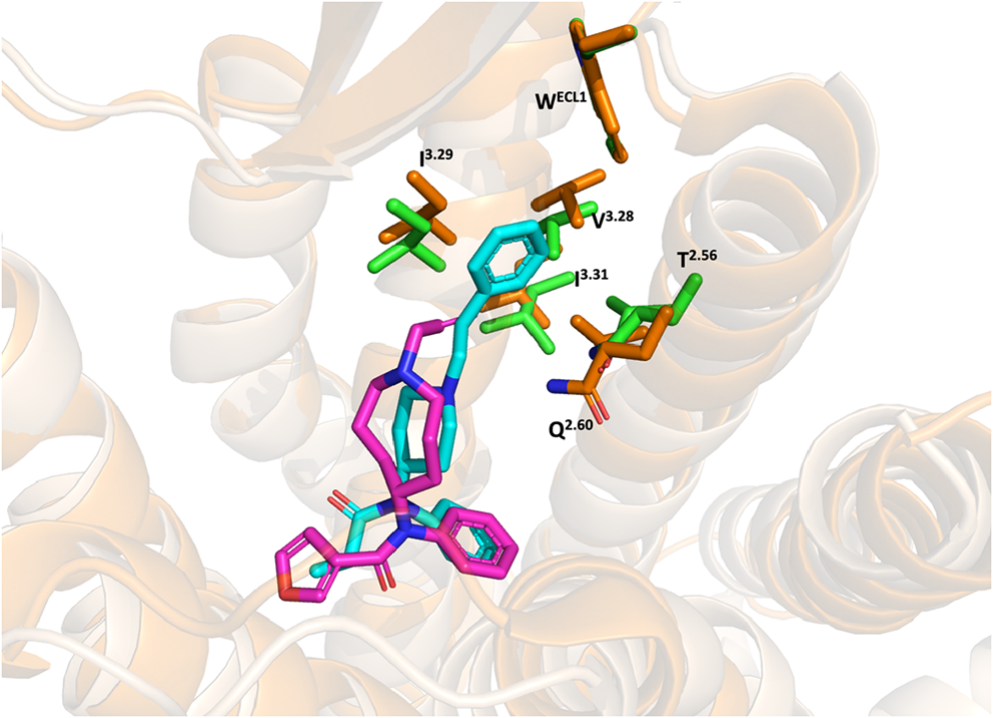
Alignment of the binding pose of compound **53** at the inactive MOR (4DKL) and Fentanyl at the active MOR (PDB: 8EF5). The MOR is shown as tints and orange cartoons, respectively. Compound **53**, Fentanyl and key amino acid residues are shown on the sticks. Carbon atoms: compound **53** (magenta) Fentanyl (cyan); key amino residues for (green); oxygen atoms (red); nitrogen atoms (blue).

## Conclusion

In this study, we successfully designed and synthesized 84 novel ring-expanded fentanyl analogs, employing the concept of ring expansion from piperidine of fentanyl to 4-azepane and 5-azocane ones while incorporating various *N*-alkyl substituents into the skeleton. Preliminary *in vivo* screening identified 15 compounds that effectively blocked morphine and fentanyl antinociceptive effects. Further dose–response analyses highlighted four potent compounds (**16**, **46**, **53**, and **69**) while compound **53** exhibiting the highest potency with AD_50_ of 2.89 and 4.02 mg/kg against morphine and fentanyl, respectively. Its antagonism at the MOR was further confirmed by *in vitro* calcium mobilization assays and binding assays. Compound **53** also demonstrated a favorable pharmacokinetic profile, including moderate human metabolic stability, low efflux, and efficient, sustained CNS penetration, crucial for a centrally acting antagonist. Crucially, *in vivo* whole-body plethysmography confirmed the ability of compound **53** to reverse fentanyl-induced respiratory depression. Molecular docking studies rationalized its antagonist profile by revealing a distinct binding mode within the MOR active site, similar to β-funaltrexamine, but notably lacking interactions with the allosteric site and key agonistic residues engaged by fentanyl. In summary, 5-azocane-containing compounds reported herein demonstrate promising MOR antagonist activity with a significantly improved respiratory profile compared to traditional opioids. These findings support the potential of this scaffold to develop novel therapeutics to counteract against fentanyl overdose through further comprehensive structure activity relationship and pharmacodynamic studies, including withdrawal and abuse liability.

## Experimental Section

### Chemistry

All nonaqueous reactions were carried out under a predried nitrogen gas atmosphere. All solvents and reagents were purchased from either Sigma-Aldrich or Alfa Aesar and were used as received without further purification. Analytical thin-layer chromatography analyses were carried out on Analtech Uniplate F254 plates, and flash column chromatography (FCC) was performed over silica gel (230–400 mesh, Merck). ^1^H (400 MHz) and ^13^C (100 MHz) nuclear magnetic resonance (NMR) spectra were recorded on a Bruker Ultrashield 400 Plus spectrometer, and chemical shifts were expressed in ppm. Mass spectra were obtained on an Applied BioSystems 3200Q trap with a turbo V source for TurbolonSpray. Analytical reversed-phase high-performance liquid chromatography (HPLC) was performed on a Waters Arc HPLC system using XBridge C_18_ 3.5 μm (4.6 × 50 mm) column. Melting points were obtained with an OptiMelt melting point apparatus from standard research systems without correction. All analyses were conducted at ambient temperature with a flow rate of 0.2 mL/min. The mobile phase is acetonitrile (70%)/water with 0.1% trifluoroacetic acid (30%). The UV detector was set up at 210 nm. Compound purities were calculated as the percentage peak area of the analyzed compound, and retention times (*R*_t_) were presented in minutes. The purity of all newly synthesized compounds was identified as ≥95%.

#### Synthesis of *tert*-Butyl 4-(Phenylamino)­azepane-1-carboxylate (**1a**)

To a solution of *tert*-butyl 4-oxoazepane-1-carboxylate (1.0 g, 4.69 mmol), acetic acid (0.40 mL, 7.03 mmol), aniline (0.64 mL, 7.03 mmol) in DCM (30 mL) on an ice bath was added sodium triacetoxyborohydride (1.99 g, 9.38 mmol) in portions at 0 °C, the resulting brown mixture was then stirred overnight being allowed to warm to room temperature. To the reaction mixture MeOH (5 mL) was then added and diluted with DCM (100 mL). The organic phase was washed with saturated NaHCO_3_ (3 × 50 mL) and brine (3 × 50 mL) and dried over Na_2_SO_4_, filtered and solvent removed under reduced pressure. The residue was then purified by FCC (hexane/EtOAc, 5/1) to afford compound **1a** (1.03 g, 67.4%) as a yellow solid.

#### Synthesis of *tert*-Butyl 5-(Phenylamino)­azocane-1-carboxylate (**1b**)

To a solution of *tert*-butyl 5-oxoazocane-1-carboxylate (5.0 g, 22 mmol), acetic acid (1.9 mL, 33 mmol), aniline (3.0 mL, 33 mmol) in DCM (100 mL) on an ice bath was added sodium triacetoxyborohydride (9.3 g, 44 mmol) in portions at 0 °C, the resulting brown mixture was then stirred overnight being allowed to warm to room temperature. To the reaction mixture MeOH (5 mL) was then added and diluted with DCM (100 mL). The organic phase was washed with saturated NaHCO_3_ (3 × 50 mL) and brine (3 × 50 mL) and dried over Na_2_SO_4_, filtered and solvent removed under reduced pressure. The residue was then purified by FCC (hexane/EtOAc, 5/1) to afford compound **1b** (4.5 g, 67.3%) as a yellow solid.

#### General Synthesis of Hetero Aromatic Intermediates (**2­(a**–**f)**)

To a solution of compound **1a** (1 g, 3.28 mmol), triethyl amine (0.81 mL, 5.81 mmol) in a predried DCM (30 mL) was slowly added the corresponding acyl chloride (3.48 mmol) on an ice bath. The reaction mixture was allowed to come to r.t. with stirring then left for stirring overnight under nitrogen gas. The progress was checked by TLC and filtered through Celite and washed the organic layer with water and brine, dried over Na_2_SO_4_ and purified by silica gel column chromatography, by using hexane/EtOAc, 4/1 to 1/1 as an eluent to afford compound **2­(a-f)** (yields between 35 to 80%) as a yellow solid. After purification, intermediates **2­(a-f)** was dissolved in DCM and slowly added trifluoroacetic acid at 0 °C. Stirred the reaction mixture overnight and dried it on rotary evaporator and used for the next step without further purification.

Similarly, the synthesis of hetero aromatic intermediates **3­(a-f)** following the same procedure as **2­(a-f)**, starting with **1b** to afford the products with yields of 43% to 60%.

#### General Synthesis of Fentanyl Analogues in the Free Base Form

The mixture of deprotected intermediates (1 equiv) with alkyl or acyl chloride (1 equiv) was reflux for 12 h. After cooling, all solvent was removed under reducing pressure. The crude residue was purified by FCC (DCM/MeOH/NH_3_·H_2_O, 20/1/0.1) to give the title compounds with yields of 25–55%.

#### General Procedure for Synthesis of Final Salt

To a solution of free base (1 equiv) in MeOH (1 mL) was added a solution of HCl/MeOH (4 equiv) dropwise at 0 °C. The clear solution was stirred for another 15 min at the same temperature following which the white solid precipitated out as the addition of ethyl ether (10 mL). The suspension was allowed to stir at r.t. for another 3 h and then filtered to give the target salts.

#### *tert*-Butyl 4-(Phenylamino)­azepane-1-carboxylate (**1a**)

The title compound was prepared following the general procedure as an off-white solid in 88% yield. ^1^H NMR (400 MHz, DMSO-*d*_6_) δ 7.17–7.13 (m, 2H), 6.68–6.65 (t, *J* = 7.2, 3.8 Hz, 1H), 6.55–6.53 (d, *J* = 8.0 Hz, 2H), 3.59–3.40 (m, 2H), 3.29–3.26 (m, 4H), 2.15–2.12 (m, 1H), 2.01–1.96 (m, 1H), 1.85–1.82 (m, 1H), 1.65–1.62 (m, 2H), 1.47 (s, 9H). ^13^C NMR (100 MHz, CDCl_3_) δ 155.52, 155.48, 146.90, 129.33, 117.23. 117.16, 113.23, 79.33, 53.05, 52.72, 46.52, 46.06, 43.35, 42.97, 35.00, 34.80, 33.41, 33.06, 24.90, 24.69. HRMS *m*/*z*: calcd for C_17_H_26_N_2_O_2_Na [M + Na]^+^: 313.1892; found, 313.1902.

#### *tert*-Butyl 5-(Phenylamino)­cyclooctane-1-carboxylate (**1b**)

The title compound was prepared following the general procedure as an off-white solid in 93% yield. ^1^H NMR (400 MHz, DMSO-*d*_6_) δ 7.05–7.01 (m, 2H), 6.49–6.45 (dd, *J* = 8.2, 6.9 Hz, 3H), 5.41 (s, 1H), 3.52–3.48 (m, 2H), 3.47–3.41 (m, 1H), 3.12–3.03 (m, 2H), 1.73–1.54 (m, 8H), 1.42 (s, 9H). ^13^C NMR (100 MHz, DMSO-*d*_6_) δ 154.90, 154.84, 149.06, 148.27, 129.41, 129.32, 116.09, 115.62, 114.34, 112.85, 79.00, 78.69, 51.21, 47.19, 47.02, 46.88, 46.53, 31.87, 30.74, 28.65, 24.66, 23.59. HRMS *m*/*z*: calcd for C_18_H_28_N_2_O_2_Na [M + Na]^+^: 327.2048; found, 327.2043.

#### *tert*-Butyl 4-(*N*-Phenylfuran-2-carboxamido)­azepane-1-carboxylate (**2a**)

The title compound was prepared following the general procedure as an off-white solid in 45% yield. ^1^H NMR (400 MHz, CDCl_3_) δ 7.49–7.32 (dd, *J* = 7.4, 3.6 Hz, 3H), 7.23–7.11 (d, *J* = 5.3 Hz, 1H), 7.10–7.08 (m, 2H), 6.05–6.04 (m, 1H), 5.41 (m, 1H), 4.68–4.66 (m, *J* = 14.4, 4.2 Hz, 1H), 3.52–3.48 (m, *J* = 78.1, 14.6, 4.5 Hz, 1H), 3.32–3.29 (m, *J* = 9.8, 8.4, 5.1 Hz, 2H), 3.09–2.80 (m, *J* = 14.0, 10.8, 2.7 Hz, 1H), 1.99–1.93 (m, 2H), 1.82–1.81 (d, *J* = 4.5 Hz, 1H), 1.67–1.61 (m, 2H), 1.42–1.31 (m, 1H), 1.17 (d, *J* = 14.8 Hz, 9H). ^13^C NMR (100 MHz, DMSO-*d*_6_): 158.67, 156.99, 153.93, 146.45, 144.20, 129.81, 129.58, 117.10, 111.49, 110.48, 77.81, 77.68, 55.89, 55.40, 45.37, 44.46, 41.92, 41.49, 32.01, 31.78, 31.38, 30.99, 27.47, 27.39, 24.29, 24.09. HRMS *m*/*z*: calcd for C_22_H_29_N_2_O_4_ [M + H]^+^: 385.2127; found, 385.2120.

#### *tert*-Butyl 4-(*N*-Phenylthiophene-2-carboxamido)­azepane-1-carboxylate (**2b**)

The title compound was prepared following the general procedure as an off-white solid in 40% yield. ^1^H NMR (400 MHz, CDCl_3_) δ 7.35–7.32 (dd, *J* = 7.4, 3.6 Hz, 3H), 7.19–7.18 (d, *J* = 5.3 Hz, 1H), 7.14–7.11 (m, 2H), 6.66–6.64 (m, 1H), 6.48–6.47 (m, 1H), 4.36–4.30 (m, *J* = 14.4, 4.2 Hz, 1H), 3.39–3.29 (m, *J* = 78.1, 14.6, 4.5 Hz, 2H), 3.11–3.04 (m, *J* = 9.8, 8.4, 5.1 Hz, 2H), 1.96–1.93 (m, 4H), 1.90–1.68 (m, 4H), 1.27 (s, 9H). ^13^C NMR (100 MHz, DMSO-*d*_6_): 161.85, 155.50, 139.56, 139.02, 131.94, 130.89, 130.72, 129.39, 128.93, 128.90, 126.62, 79.32, 79.29, 57.93, 57.56, 46.59, 45.34, 43.03, 42.82, 33.62, 33.02, 32.85, 32.19, 28.42, 25.29. HRMS *m*/*z*: calcd for C_22_H_29_N_2_O_3_S [M + H]^+^: 401.1899; found, 401.1894.

#### *tert*-Butyl 4-(*N*-Phenylfuran-3-carboxamido)­azepane-1-carboxylate (**2c**)

The title compound was prepared following the general procedure as an off-white solid in 48% yield. ^1^H NMR (400 MHz, CDCl_3_) δ 7.35–7.33 (dd, *J* = 7.4, 3.6 Hz, 3H), 7.21–7.08 (d, *J* = 5.3 Hz, 2H), 7.05–7.04 (m, 1H), 6.45–6.43 (m, 1H), 6.08 (s, 1H), 4.69–4.68 (m, *J* = 14.4, 4.2 Hz, 1H), 3.70–3.51 (m, *J* = 78.1, 14.6, 4.5 Hz, 1H), 3.31–3.27 (m, *J* = 9.8, 8.4, 5.1 Hz, 2H), 3.08–3.01 (m, *J* = 14.0, 10.8, 2.7 Hz, 1H), 2.01–1.92 (m, 2H), 1.84–1.82 (d, *J* = 4.5 Hz, 1H), 1.66–1.60 (m, 2H), 1.59–1.55 (m, 1H), 1.33–1.29 (d, *J* = 14.8 Hz, 9H). ^13^C NMR (100 MHz, DMSO-*d*_6_): 162.49, 155.49, 145.21, 141.79, 139.55, 130.66, 129.34, 129.06, 128.83, 122.49, 111.30, 79.31, 79.26, 57.05, 56.69, 46.57, 45.31, 42.97, 42.77, 33.65, 33.07, 32.88, 32.19, 29.40, 25.24. HRMS *m*/*z*: calcd for C_22_H_29_N_2_O_4_ [M + H]^+^: 385.2127; found, 385.2119.

#### *tert*-Butyl 4-(*N*-Phenyl-1*H*-pyrrole-2-carboxamido)­azepane-1-carboxylate (**2d**)

The title compound was prepared following the general procedure as an off-white solid in 45% yield. ^1^H NMR (400 MHz, CDCl_3_) δ 9.70 (s, 1H), 7.37 (dd, *J* = 7.5, 2.1 Hz, 3H), 7.18–7.09 (m, 2H), 6.72 (d, *J* = 2.8 Hz, 1H), 5.83–5.75 (m, 1H), 4.73 (d, *J* = 8.3 Hz, 1H), 4.59–4.48 (m, 1H), 3.63 (m, *J* = 79.3, 14.6, 4.4 Hz, 1H), 3.29 (m, *J* = 24.0, 19.2, 16.5, 11.6 Hz, 2H), 3.05 (m, *J* = 14.6, 8.9, 5.8 Hz, 1H), 2.05–1.91 (m, 3H), 1.91–1.73 (m, 1H), 1.73–1.50 (m, 2H), 1.31 (d, *J* = 15.3 Hz, 10H). ^13^C NMR (100 MHz, CDCl_3_) δ 160.67, 155.51, 139.51, 130.93, 129.36, 128.85, 128.81, 125.48, 120.78, 113.31, 113.28, 109.68, 79.25, 60.38, 56.88, 56.52, 46.55, 45.31, 42.94, 42.69, 33.77, 33.13, 32.98, 32.24, 29.70, 28.45, 28.41, 25.24, 21.03, 14.20. HRMS *m*/*z*: calcd for C_22_H_29_N_3_O_3_Na [M + Na]^+^: 406.2107; found, 406.2012.

#### *tert*-Butyl 4-(*N*-Phenyl-1*H*-pyrrole-3-carboxamido)­azepane-1-carboxylate (**2e**)

The title compound was prepared following the general procedure as an off-white solid in 45% yield. ^1^H NMR (400 MHz, CDCl_3_) δ 9.59 (s, 1H), 7.37 (dd, *J* = 7.6, 2.0 Hz, 3H), 7.18–7.10 (m, 2H), 6.72 (q, *J* = 2.3 Hz, 1H), 5.79 (q, *J* = 3.0 Hz, 1H), 4.80–4.66 (m, 1H), 4.58–4.48 (m, 1H), 3.79–3.48 (m, 1H), 3.29 (m, *J* = 17.6, 10.4, 4.9 Hz, 2H), 3.04 (m, *J* = 14.3, 11.1, 3.3 Hz, 1H), 2.05–1.91 (m, 2H), 1.90–1.75 (m, 1H), 1.72–1.50 (m, 3H), 1.31 (d, *J* = 15.2 Hz, 10H). ^13^C NMR (100 MHz, CDCl_3_) δ 160.64, 155.50, 139.51, 130.93, 129.35, 128.83, 125.49, 120.72, 113.28, 109.70, 56.88, 56.51, 46.56, 45.32, 42.94, 42.70, 33.76, 33.14, 32.98, 32.24, 28.45, 28.41, 25.23. HRMS *m*/*z*: calcd for C_22_H_30_N_3_O_3_ [M + H]^+^: 384.2287; found, 384.2265.

#### *tert*-Butyl 4-(*N*-Phenylthiophene-3-carboxamido)­azepane-1-carboxylate (**2f**)

The title compound was prepared following the general procedure as an off-white solid in 45% yield. ^1^H NMR (400 MHz, DMSO-*d*_6_): 7.34–7.31 (m, 3H), 7.29–7.26 (m, 1H), 7.20–7.19 (m, 3H), 6.80 (m, 1H), 4.55–4.53 (m, 1H), 3.51–3.48 (m, 1H), 3.44–3.40 (m, 1H), 3.32–3.13 (m, 2H), 2.40–1.98 (m, 2H), 1.81–1.80 (m, 1H), 1.67–1.53 (m, 2H), 1.41–1.30 (m, 1H), 1.27–1.25 (m, 9H).^13^C NMR (100 MHz, DMSO-*d*_6_): 164.09, 154.99, 140.61,138.16,130.76, 129.38, 129.09, 128.47, 128.32, 125.51, 78.86, 78.74, 57.43, 56.86, 55.36, 46.50, 45.57, 43.18, 42.77, 33.17, 33.07, 32.64, 32.30, 28.55, 28.46, 25.55, 25.32. HRMS *m*/*z*: calcd for C_22_H_29_N_2_O_3_S [M + H]^+^: 401.1899; found, 401.1894.

#### *tert*-Butyl 5-(*N*-Phenylfuran-2-carboxamido)­azocane-1-carboxylate (**3a**)

The title compound was prepared following the general procedure as an off-white solid in 45% yield. ^1^H NMR (400 MHz, CDCl_3_) δ 7.43–7.22 (m, 3H), 7.17–7.07 (m, 2H), 7.04 (q, *J* = 1.8, 1.3 Hz, 1H), 6.51 (d, *J* = 1.4 Hz, 1H), 6.04 (dd, *J* = 1.9, 0.9 Hz, 1H), 4.40 (tt, *J* = 9.2, 3.6 Hz, 1H), 3.48–3.22 (m, 2H), 3.09 (m, *J* = 21.9, 14.2, 7.2, 5.0 Hz, 2H), 2.01–1.77 (m, 4H), 1.78–1.62 (m, 4H), 1.28 (s, 9H). ^13^C NMR (100 MHz, CDCl_3_) δ 162.30, 155.48, 145.21, 141.72, 129.92, 129.42, 128.47, 122.88, 111.23, 79.20, 59.85, 46.56, 46.51, 31.72, 31.49, 28.43, 26.51, 25.21. HRMS *m*/*z*: calcd for C_23_H_31_N_2_O_4_ [M + H]^+^: 399.2284; found, 399.2253.

#### *tert*-Butyl 5-(*N*-Phenylfuran-3-carboxamido)­azocane-1-carboxylate (**3b**)

The title compound was prepared following the general procedure as an brown oily in 55% yield.^1^H NMR (400 MHz, CDCl_3_) δ 7.30 (tt, *J* = 4.1, 2.6 Hz, 3H), 7.22 (dd, *J* = 1.7, 0.8 Hz, 1H), 7.14–7.06 (m, 2H), 6.05 (dd, *J* = 3.5, 1.8 Hz, 1H), 5.41 (d, *J* = 3.5 Hz, 1H), 4.43 (tt, *J* = 8.9, 3.5 Hz, 1H), 3.51–3.29 (m, 2H), 3.17–2.97 (m, 2H), 2.01–1.67 (m, 9H), 1.30 (s, 9H). ^13^C NMR (100 MHz, CDCl_3_) δ 158.53, 155.47, 147.61, 144.07, 141.38, 129.65, 129.29, 128.27, 115.68, 110.79, 79.25, 46.49, 46.47, 31.79, 31.39, 29.70, 28.45, 26.50, 25.08. HRMS *m*/*z*: calcd for C_23_H_31_N_2_O_4_ [M + H]^+^: 399.2284; found, 399.2280.

#### *tert*-Butyl 5-(*N*-Phenylthiophene-2-carboxamido)­azocane-1-carboxylate (**3c**)

The title compound was prepared following the general procedure as an off-white solid in 45% yield. ^1^H NMR (400 MHz, CDCl_3_): 7.40–7.38 (m, 3H), 7.28–7.27 (m, 1H), 7.26–7.21 (m, 2H), 6.76–6.74 (m, 1H), 6.59- 6.57 (m, 1H), 4.46–4.43 (m, 1H), 3.51–3.20 (m, 2H), 3.19–3.12 (m, 2H), 2.04–1.99 (m, 4H), 1.98–1.81 (m, 4H), 1.37 (s, 9H).^13^C NMR (100 MHz, CDCl_3_): 161.67, 155.44, 141.75, 139.57, 131.66, 130.36, 13.01, 129.46, 129.49, 126.53, 79.19, 61.06, 46.57, 46.54, 31.70, 31.49, 28.43, 25.56, 25.26. HRMS *m*/*z*: calcd for C_23_H_31_N_2_O_3_S [M + H]^+^: 415.2055; found, 415.2054.

#### *tert*-Butyl 5-(*N*-Phenyl-1*H*-pyrrole-2-carboxamido)­azocane-1-carboxylate (**3d**)

The title compound was prepared following the general procedure as an off-white solid in 55% yield. ^1^H NMR (400 MHz, DMSO-*d*_6_): 11.32 (s, 1H), 7.44–7.42 (m, 3H), 7.25–7.23 (m, 2H), 6.72–6.70 (m, 1H), 5.75–5.69 (m, 1H), 4.51–4.49 (m, 1H), 3.39–3.37 (m, 3H), 3.09–3.04 (m, 2H), 1.98- 1.82 (m, 2H), 1.75–1.68 (m, 5H), 1.38 (m, 9H).^13^C NMR (100 MHz, DMSO-*d*_6_): 164.09, 154.99, 140.61,138.16,130.76, 129.38, 129.09, 128.47, 128.32, 125.51, 78.86, 78.74, 57.43, 56.86, 55.36, 46.50, 45.57, 43.18, 42.77, 33.17, 33.07, 32.64, 32.30, 28.55, 28.46, 25.55, 25.32.HRMS *m*/*z*: calcd for C_23_H_32_N_3_O_3_ [M + H]^+^: 398.2444; found, 398.2442.

#### *tert*-Butyl 4-(*N*-Phenylthiophene-3-carboxamido)­azepane-1-carboxylate (**3e**)

The title compound was prepared following the general procedure as an off-white solid in 45% yield. ^1^H NMR (400 MHz, CDCl_3_) δ 7.25–7.17 (m, 3H), 7.07–7.01 (m, 2H), 6.97 (dd, *J* = 3.0, 1.3 Hz, 1H), 6.90 (dd, *J* = 5.1, 3.0 Hz, 1H), 6.78 (dd, *J* = 5.1, 1.3 Hz, 1H), 4.38 (t, *J* = 6.3 Hz, 1H), 3.56–3.31 (m, 2H), 3.14–2.97 (m, 2H), 1.95 (dq, *J* = 22.1, 6.3, 5.1 Hz, 4H), 1.75 (q, *J* = 6.4, 5.8 Hz, 4H), 1.32 (s, 10H). ^13^C NMR (100 MHz, CDCl_3_) δ 163.92, 155.41, 137.87, 129.24, 129.17, 128.53, 127.60, 124.01, 79.19, 46.55, 46.52, 32.00, 31.64, 28.44, 26.68, 25.28. HRMS *m*/*z*: calcd for C_23_H_31_N_2_O_3_S [M + H]^+^: 415.2055; found, 415.2050.

#### *tert*-Butyl 5-(*N*-Phenyl-1*H*-pyrrole-3-carboxamido)­azocane-1-carboxylate (**3f**)

The title compound was prepared following the general procedure as an off-white solid in 45% yield. ^1^H NMR (400 MHz, DMSO-*d*_6_) δ 10.80 (s, 1H), 7.45–7.32 (m, 3H), 7.23–7.13 (m, 2H), 6.44 (q, *J* = 2.4 Hz, 1H), 6.08 (dt, *J* = 3.3, 1.8 Hz, 1H), 5.60 (td, *J* = 2.7, 1.5 Hz, 1H), 4.44 (s, 1H), 4.11 (q, *J* = 5.4 Hz, 1H), 3.36–3.25 (m, 2H), 3.07 (dt, *J* = 13.4, 5.1 Hz, 2H), 1.95–1.78 (m, 2H), 1.78–1.56 (m, 6H), 1.31 (s, 9H). ^13^C NMR (100 MHz, DMSO-*d*_6_) δ 164.04, 154.92, 142.66, 130.78, 129.44, 128.12, 122.34, 119.91, 117.52, 110.26, 78.65, 49.07, 46.53, 46.40, 32.01, 31.73, 28.53, 26.46, 25.12. HRMS *m*/*z*: calcd for C_23_H_32_N_3_O_3_ [M + H]^+^: 398.2444; found, 398.2336.

#### *N*-(1-Allylazepan-4-yl)-*N*-phenylfuran-2-carboxamide Hydrogen Chloride (**4**)

The title compound was prepared following the general procedure as an off-white solid in 45% yield. Hydrochloride salt: ^1^H NMR (400 MHz, DMSO-*d*_6_): δ 9.77 (s, 1H), 7.34–7.16 (m, 15H), 4.82 (t, *J* = 12.0 Hz, 1H), 3.62–3.59 (m, 2H), 3.25–3.15 (m, 4H), 3.00–2.95 (m, 2H), 2.15–2.12 (m, 2H), 1.82–1.73 (m, 2H). ^13^C NMR (100 MHz, DMSO-*d*_6_) δ 158.34, 147.15, 145.52, 139.54, 130.83, 129.93, 129.87, 129.27, 128.17, 128.06, 126.36, 116.15, 111.66, 111.63, 63.66, 63.48, 61.39, 58.76, 57.28, 45.30, 41.95, 32.00, 31.43, 26.72, 21.69, 18.68. HRMS *m*/*z*: calcd for C_20_H_25_N_2_O_2_ [M + H]^+^: 325.1916; found, 325.1897. Mp 175.8–177.3 °C. The purity of the compound was checked by HPLC (*R*_t_ = 2.658 min) and was found to be 100% pure.

#### *N*-(1-(Cyclopropylmethyl)­azepan-4-yl)-*N*-phenylfuran-2-carboxamide Hydrogen Chloride (**5**)

The title compound was prepared following the general procedure as white powder in 74% yield. Hydrochloride salt: ^1^H NMR (400 MHz, DMSO-*d*_6_) δ 10.01 (d, *J* = 25.4 Hz, 1H), 7.63 (d, *J* = 1.7 Hz, 1H), 7.49 (p, *J* = 3.1 Hz, 3H), 7.31 (dd, *J* = 6.0, 3.1, 1.2 Hz, 2H), 6.32 (dd, *J* = 3.7, 1.7 Hz, 1H), 5.47 (dd, *J* = 8.8, 3.5 Hz, 1H), 4.66 (dd, *J* = 16.4, 12.3, 7.3, 4.6 Hz, 1H), 3.53 (dd, *J* = 13.4, 7.3 Hz, 1H), 3.30 (s, 1H), 3.18 (td, *J* = 13.2, 6.4 Hz, 2H), 3.00–2.83 (m, 3H), 2.21 (s, 1H), 2.19–1.91 (m, 3H), 1.91–1.75 (m, 2H), 1.74–1.53 (m, 1H), 1.09–1.01 (m, 1H), 0.60 (dd, *J* = 7.9, 6.2, 1.8 Hz, 2H), 0.36 (m, *J* = 5.0, 3.1, 2.7 Hz, 2H). ^13^C NMR (100 MHz, DMSO-*d*_6_) δ 147.23, 145.46, 130.81, 130.62, 129.93, 129.91, 129.24, 116.13, 111.63, 61.15, 60.96, 55.37, 53.30, 50.58, 50.03, 30.92, 28.99, 27.71, 19.82, 15.63, 6.22, 6.15, 4.70, 4.58, 4.41. HRMS *m*/*z*: calcd for C_21_H_27_N_2_O_2_ [M + H]^+^: 339.2073; found, 339.2059. Mp 208.3–209.9 °C. The purity of the compound was checked by HPLC (*R*_t_ = 2.672 min) and was found to be 99.70% pure.

#### *N*-(1-(Cyclobutylmethyl)­azepan-4-yl)-*N*-phenylfuran-2-carboxamide Hydrogen Chloride (**6**)

The title compound was prepared following the general procedure as a white sold in 51% yield. Hydrochloride salt: ^1^H NMR (400 MHz, DMSO-*d*_6_) δ 9.89 (d, *J* = 31.5 Hz, 1H), 7.62 (d, *J* = 1.6 Hz, 1H), 7.54–7.44 (m, 3H), 7.35–7.25 (m, 2H), 6.31 (dt, *J* = 3.4, 1.5 Hz, 1H), 5.46 (dd, *J* = 7.8, 3.5 Hz, 1H), 4.65 (m, *J* = 16.2, 12.3, 8.5, 4.0 Hz, 1H), 3.16–3.01 (m, 4H), 2.90–2.74 (m, 1H), 2.68 (dt, *J* = 15.1, 7.6 Hz, 1H), 2.33–2.10 (m, 1H), 2.09–1.92 (m, 4H), 1.92–1.78 (m, 4H), 1.76 (m, *J* = 7.8, 5.2, 3.6 Hz, 2H), 1.68–1.51 (m, 1H). ^13^C NMR (100 MHz, DMSO-*d*_6_) δ 158.32, 158.27, 147.28, 147.23, 145.45, 140.04, 139.75, 130.78, 130.61, 129.92, 129.89, 129.23, 129.17, 116.12, 111.62, 61.69, 61.46, 56.56, 55.37, 53.85, 53.58, 50.82, 50.39, 31.58, 30.88, 30.79, 30.77, 28.99, 27.61, 27.29, 27.27, 27.21, 26.98, 21.65, 19.77, 18.57, 18.55. HRMS *m*/*z*: calcd for C_22_H_29_N_2_O_2_ [M + H]^+^: 353.2229; found, 353.2206. Mp 232.6–234.2 °C. The purity of the compound was checked by HPLC (*R*_t_ = 2.740 min) and was found to be 99.66% pure.

#### *N*-(1-(Cyclopentylmethyl)­azepan-4-yl)-*N*-phenylfuran-2-carboxamide Hydrogen Chloride (**7**)

The title compound was prepared following the general procedure as a white solid in 70% yield. Hydrochloride salt: ^1^H NMR (400 MHz, DMSO-*d*_6_) δ 9.78 (d, *J* = 29.7 Hz, 1H), 7.62 (d, *J* = 1.7 Hz, 1H), 7.48 (dd, *J* = 4.9, 3.5, 2.1 Hz, 3H), 7.30 (dd, *J* = 6.0, 3.1, 1.4 Hz, 2H), 6.32 (dd, *J* = 3.6, 1.7 Hz, 1H), 5.46 (t, *J* = 3.5 Hz, 1H), 4.65 (pd, *J* = 8.7, 7.7, 3.2 Hz, 1H), 3.56–3.36 (m, 3H), 3.15 (q, *J* = 9.6, 8.7 Hz, 2H), 3.09–2.94 (m, 2H), 2.93–2.75 (m, 1H), 2.21 (s, 1H), 2.19–2.10 (m, 1H), 2.03 (dt, *J* = 21.0, 7.1 Hz, 2H), 1.96–1.70 (m, 4H), 1.70–1.45 (m, 5H), 1.29–1.13 (m, 2H). ^13^C NMR (100 MHz, DMSO-*d*_6_): 164.5, 140.1, 137.9, 137.6, 130.8, 129.7, 129.6, 129.1, 128.5, 128.4, 127.3, 125.8, 56.6, 49.9, 49.0, 47.3, 38.6, 36.2, 35.7, 32.3, 30.7, 29.9. HRMS *m*/*z*: calcd for C_23_H_31_N_2_O_2_ [M + H]^+^: 367.2386; found, 367.2379. Mp 161.5–163.2 °C. The purity of the compound was checked by HPLC (*R*_t_ = 2.803 min) and was found to be 98.92% pure.

#### *N*-(1-(Cyclohexylmethyl)­azepan-4-yl)-*N*-phenylfuran-2-carboxamide Hydrogen Chloride (**8**)

The title compound was prepared following the general procedure as a white sold in 85% yield. Hydrochloride salt: ^1^H NMR (400 MHz, DMSO-*d*_6_) δ 9.73 (d, *J* = 39.4 Hz, 1H), 7.55–7.40 (m, 4H), 7.30 (m, *J* = 5.6, 2.3 Hz, 2H), 6.77 (s, 1H), 5.97 (d, *J* = 2.1 Hz, 1H), 4.67 (dd, *J* = 16.3, 11.9, 6.8 Hz, 1H), 3.23–3.08 (m, 2H), 2.90 (m, *J* = 17.5, 5.7 Hz, 2H), 2.01 (m, *J* = 14.7, 11.3, 10.2 Hz, 2H), 1.87–1.48 (m, 9H), 1.30–1.02 (m, 3H), 0.91 (qt, *J* = 11.9, 3.4 Hz, 2H). ^13^C NMR (100 MHz, DMSO-*d*_6_): 164.5, 140.1, 137.9, 137.6, 130.8, 129.7, 129.6, 129.1, 128.5, 128.4, 127.3, 125.8, 56.6, 49.9, 49.0, 47.3, 38.6, 36.2, 35.7, 32.3, 30.7, 29.9. HRMS *m*/*z*: calcd., for C_24_H_33_N_2_O_2_ [M + H]^+^: 381.2542; found, 381.2528. Mp 164.3–166.1 °C. The purity of the compound was checked by HPLC (*R*_t_ = 2.918 min) and was found to be 99.26% pure.

#### *N*-(1-Benzylazepan-4-yl)-*N*-phenylfuran-2-carboxamide Hydrogen Chloride (**9**)

The title compound was prepared following the general procedure as a light-yellow oil in 58% yield. Hydrochloride salt: ^1^H NMR (400 MHz, DMSO-*d*_6_) δ 10.25 (d, *J* = 47.2 Hz, 1H), 7.66–7.60 (m, 1H), 7.60–7.51 (m, 2H), 7.51–7.41 (m, 6H), 7.32–7.21 (m, 2H), 6.32 (td, *J* = 3.6, 1.8 Hz, 1H), 5.55 (d, *J* = 3.5 Hz, 1H), 4.93 (p, *J* = 8.8 Hz, 1H), 4.32–4.15 (m, 2H), 3.03 (d, *J* = 12.4 Hz, 1H), 2.91 (q, *J* = 10.5 Hz, 1H), 2.77–2.64 (m, 1H), 2.27 (m, *J* = 12.2, 8.1, 4.4 Hz, 1H), 2.07 (dt, *J* = 12.1, 4.2 Hz, 1H), 1.98 (d, *J* = 13.8 Hz, 1H), 1.89–1.79 (m, 1H), 1.78–1.65 (m, 2H), 1.59 (dd, *J* = 11.3, 9.4 Hz, 1H), 1.29 (d, *J* = 14.2 Hz, 1H). ^13^C NMR (100 MHz, DMSO-*d*_6_): 164.5, 140.1, 137.9, 137.6, 130.8, 129.7, 129.6, 129.1, 128.5, 128.4, 127.3, 125.8, 56.6, 49.9, 49.0, 47.3, 38.6, 36.2, 35.7, 32.3, 30.7, 29.9. HRMS *m*/*z*: calcd for C_24_H_27_N_2_O_2_ [M + H]^+^: 375.2073; found, 375.2061. Mp 223.4–225.1 °C. The purity of the compound was checked by HPLC (*R*_t_ = 2.772 min) and was found to be 99.58% pure.

#### *N*-(1-Phenethylazepan-4-yl)-*N*-phenylfuran-2-carboxamide Hydrogen Chloride (**10**)

The title compound was prepared following the general procedure as a white sold in 88% yield. Hydrochloride salt: ^1^H NMR (400 MHz, DMSO-*d*_6_): 9.82 (s, 1H), 7.54–7.47 (m, 4H), 7.36–7.32 (m, 2H), 7.27–7.24 (d, 5H), 6.86–6.80 (d, 1H), 6.01–6.02 (d, 1H), 4.98–4.95 (p, 1H), 3.47–3.44 (m, 1H), 3.32–3.29 (m, 1H), 3.23–3.19 (m, 2H), 3.01–2.93 (m, 3H), 2.76–2.73 (m, 1H), 2.33–2.31 (m, 1H), 2.12–2.11 (m, 1H), 2.04–2.01 (m, 1H), 1.79–1.74 (m, 2H), 1.71–1.60 (m, 2H), 1.34–1.31 (m, 1H). ^13^C NMR (100 MHz, DMSO-*d*_6_): 164.5, 140.1, 137.9, 137.6, 130.8, 129.7, 129.6, 129.1, 128.5, 128.4, 127.3, 125.8, 56.6, 49.9, 49.0, 47.3, 38.6, 36.2, 35.7, 32.3, 30.7, 29.9. HRMS *m*/*z*: calcd for C_25_H_28_N_2_O_2_ [M + H]^+^: 389.2229; found, 388.2214. Mp 161.8–162.7 °C. The purity of the compound was checked by HPLC (*R*_t_ = 2.878 min) and was found to be 97.77% pure.

#### *N*-(1-Allylazepan-4-yl)-*N*-phenylthiophene-2-carboxamide Hydrogen Chloride (**11**)

The title compound was prepared following the general procedure as a pale solid in 80% yield. Hydrochloride salt: ^1^H NMR (400 MHz, DMSO-*d*_6_): 9.92 (s, 1H), 7.44–7.31 (m, 6H), 7.28–7.24 (m, 4H), 7.21–7.19 (d, 2H), 6.81–6.80­(d, 1H), 4.95 (p, 1H), 3.47–3.44 (m, 1H), 3.32–3.29 (m, 1H), 3.23–3.19 (m, 2H), 3.01–2.93 (m, 3H), 2.76–2.73 (m, 1H), 2.33–2.31 (m, 1H), 2.12–2.11 (m, 1H), 2.04–2.01 (m, 1H), 1.79–1.74 (m, 2H), 1.71–1.60 (m, 2H), 1.34–1.31 (m, 1H). ^13^C NMR (100 MHz, DMSO-*d*_6_): 164.5, 140.1, 137.9, 137.6, 130.8, 129.7, 129.6, 129.1, 128.5, 128.4, 127.3, 125.8, 56.6, 49.9, 49.0, 47.3, 38.6, 36.2, 35.7, 32.3, 30.7, 29.9. HRMS *m*/*z*: calcd for C_20_H_24_N_2_OS [M + H]^+^: 341.1668; found, 341.1672. Mp 165.5–167.3 °C. The purity of the compound was checked by HPLC (*R*_t_ = 2.732 min) and was found to be 96.91% pure.

#### *N*-(1-(Cyclopropylmethyl)­azepan-4-yl)-*N*-phenylthiophene-2-carboxamide Hydrogen Chloride (**12**)

The title compound was prepared following the general procedure as a white solid in 94% yield. Hydrochloride salt: ^1^H NMR (400 MHz, DMSO-*d*_6_) δ 10.19 (d, *J* = 26.8 Hz, 1H), 7.60 (dd, *J* = 5.1, 1.2 Hz, 1H), 7.54–7.38 (m, 3H), 7.35 (m, *J* = 4.1, 3.5, 2.2 Hz, 2H), 6.83 (dd, *J* = 5.1, 3.8 Hz, 1H), 6.39 (dd, *J* = 3.8, 1.2 Hz, 1H), 4.84–4.45 (m, 1H), 3.19 (dt, *J* = 13.5, 4.8 Hz, 2H), 3.07–2.79 (m, 3H), 2.41–2.15 (m, 1H), 2.15–1.94 (m, 2H), 1.94–1.77 (m, 2H), 1.77–1.30 (m, 2H), 1.08–1.03 (m, 1H), 0.70–0.52 (m, 2H), 0.37 (m, *J* = 4.5, 2.7, 1.9 Hz, 2H). ^13^C NMR (100 MHz, DMSO-*d*_6_): 164.5, 140.1, 137.9, 137.6, 130.8, 129.7, 129.6, 129.1, 128.5, 128.4, 127.3, 125.8, 56.6, 49.9, 49.0, 47.3, 38.6, 36.2, 35.7, 32.3, 30.7, 29.9. HRMS *m*/*z*: calcd for C_21_H_26_N_2_OS [M + H]^+^: 355.1844; found, 355.1840. Mp 161.5–163.2 °C. The purity of the compound was checked by HPLC (*R*_t_ = 2.663 min) and was found to be 99.16% pure.

#### *N*-(1-(Cyclobutylmethyl)­azepan-4-yl)-*N*-phenylthiophene-2-carboxamide Hydrogen Chloride (**13**)

The title compound was prepared following the general procedure as a white sold in 97% yield. Hydrochloride salt: ^1^H NMR (400 MHz, DMSO-*d*_6_) δ 9.64 (d, *J* = 47.7 Hz, 1H), 7.60 (dd, *J* = 5.0, 1.1 Hz, 1H), 7.50 (m, *J* = 6.5, 2.9, 1.3 Hz, 3H), 7.34 (m, *J* = 7.2, 4.4, 3.0 Hz, 2H), 6.82 (dd, *J* = 5.1, 3.8 Hz, 1H), 6.39 (dd, *J* = 3.8, 1.2 Hz, 1H), 4.80–4.50 (m, 1H), 3.22–2.95 (m, 5H), 2.95–2.77 (m, 1H), 2.77–2.60 (m, 1H), 2.35–2.15 (m, 1H), 2.15–1.96 (m, 4H), 1.96–1.69 (m, 6H), 1.69–1.47 (m, 1H). ^13^C NMR (100 MHz, DMSO-*d*_6_) δ 161.40, 139.81, 139.22, 132.06, 131.85, 131.26, 131.08, 130.13, 130.10, 129.58, 127.42, 61.72, 61.47, 53.70, 50.83, 50.46, 31.56, 30.86, 30.79, 30.76, 27.66, 27.24, 27.17, 26.95, 21.68, 19.87, 18.57, 18.55. HRMS *m*/*z*: calcd for C_24_H_27_N_2_OS [M + H]^+^: 369.2001; found, 369.2011. Mp 173.1–174.9 °C. The purity of the compound was checked by HPLC (*R*_t_ = 2.730 min) and was found to be 99.20% pure.

#### *N*-(1-(Cyclopentylmethyl)­azepan-4-yl)-*N*-phenylthiophene-2-carboxamide Hydrogen Chloride (**14**)

The title compound was prepared following the general procedure as a white solid in 85% yield. Hydrochloride salt: ^1^H NMR (400 MHz, DMSO-*d*_6_) δ 9.15 (d, *J* = 61.8 Hz, 1H), 7.60 (dd, *J* = 5.1, 1.2 Hz, 1H), 7.56–7.42 (m, 3H), 7.40–7.24 (m, 2H), 6.83 (dd, *J* = 5.0, 3.8 Hz, 1H), 6.39 (m, *J* = 4.9, 3.8, 1.2 Hz, 1H), 4.67 (dd, *J* = 10.4, 5.5 Hz, 1H), 3.59–3.37 (m, 2H), 3.18 (td, *J* = 8.3, 6.8, 4.1 Hz, 2H), 3.12–2.97 (m, 2H), 2.97–2.78 (m, 1H), 2.32–2.03 (m, 4H), 1.96 (dt, *J* = 16.0, 11.8 Hz, 1H), 1.90–1.69 (m, 4H), 1.69–1.41 (m, 5H), 1.19 (m, *J* = 13.8, 9.2, 8.5, 4.6 Hz, 2H). ^13^C NMR (100 MHz, DMSO-*d*_6_) δ 161.46, 161.40, 139.99, 139.83, 139.21, 139.16, 132.07, 131.86, 131.27, 131.14, 130.12, 129.59, 129.55, 127.42, 61.77, 61.51, 57.38, 57.17, 54.10, 51.18, 50.74, 35.27, 31.78, 31.16, 31.10, 31.06, 30.90, 28.82, 27.24, 25.11, 25.08, 25.03, 21.42, 19.50. HRMS *m*/*z*: calcd for C_25_H_29_N_2_OS [M + H]^+^: 383.2157; found, 383.2166. Mp 184.8–186.1 °C. The purity of the compound was checked by HPLC (*R*_t_ = 2.790 min) and was found to be 98.87% pure.

#### *N*-(1-(Cyclohexylmethyl)­azepan-4-yl)-*N*-phenylthiophene-2-carboxamide Hydrogen Chloride (**15**)

The title compound was prepared following the general procedure as a white solid in 97% yield. Hydrochloride salt: ^1^H NMR (400 MHz, DMSO-*d*_6_) δ 9.52 (d, *J* = 44.3 Hz, 1H), 7.60 (dd, *J* = 5.0, 1.2 Hz, 1H), 7.50 (m, *J* = 5.4, 1.6 Hz, 3H), 7.42–7.26 (m, 2H), 6.83 (dd, *J* = 5.0, 3.8 Hz, 1H), 6.39 (td, *J* = 3.8, 1.2 Hz, 1H), 4.67 (tt, *J* = 11.5, 6.4 Hz, 1H), 3.55–3.36 (m, 1H), 3.31–3.05 (m, 3H), 3.07–2.74 (m, 3H), 2.34–2.16 (m, 1H), 2.16–1.93 (m, 2H), 1.93–1.49 (m, 8H), 1.30–1.00 (m, 3H), 1.00–0.77 (m, 2H). ^13^C NMR (100 MHz, DMSO-*d*_6_) δ 161.45, 161.38, 139.99, 139.78, 139.22, 139.17, 132.06, 131.85, 131.83, 131.26, 131.14, 130.12, 130.10, 129.59, 129.54, 127.42, 62.64, 62.44, 57.62, 57.28, 54.28, 54.10, 51.38, 50.88, 32.99, 31.79, 31.34, 30.85, 30.83, 30.75, 30.66, 28.70, 27.02, 25.94, 25.43, 25.40, 21.26, 19.14. HRMS *m*/*z*: calcd for C_26_H_31_N_2_OS [M + H]^+^: 397.2143; found, 397.2320. Mp 161.5–163.2 °C. The purity of the compound was checked by HPLC (*R*_t_ = 2.918 min) and was found to be 99.36% pure.

#### *N*-(1-Benzylazepan-4-yl)-*N*-phenylthiophene-2-carboxamide Hydrogen Chloride (**16**)

The title compound was prepared following the general procedure as a white sold in 91% yield. Hydrochloride salt: ^1^H NMR (400 MHz, DMSO-*d*_6_): 9.92 (s, 1H), 7.44–7.31 (m, 6H), 7.28–7.24 (m, 4H), 7.21–7.19 (d, 2H), 6.81–6.80 (d, 1H), 4.95 (p, 1H), 3.47–3.44 (m, 1H), 3.32–3.29 (m, 1H), 3.23–3.19 (m, 2H), 3.01–2.93 (m, 3H), 2.76–2.73 (m, 1H), 2.33–2.31 (m, 1H), 2.12–2.11 (m, 1H), 2.04–2.01 (m, 1H), 1.79–1.74 (m, 2H), 1.71–1.60 (m, 2H), 1.34–1.31 (m, 1H). ^13^C NMR (100 MHz, DMSO-*d*_6_): 164.5, 140.1, 137.9, 137.6, 130.8, 129.7, 129.6, 129.1, 128.5, 128.4, 127.3, 125.8, 56.6, 49.9, 49.0, 47.3, 38.6, 36.2, 35.7, 32.3, 30.7, 29.9. HRMS *m*/*z*: calcd for C_24_H_27_N_2_OS [M + H]^+^: 391.1844; found, 391.1829. Mp 187.9–189.2 °C. The purity of the compound was checked by HPLC (*R*_t_ = 2.877 min) and was found to be 96.28% pure.

#### *N*-(1-Phenethylazepan-4-yl)-*N*-phenylthiophene-2-carboxamide Hydrogen Chloride (**17**)

The title compound was prepared following the general procedure as a white solid in 82% yield. Hydrochloride salt: ^1^H NMR (400 MHz, DMSO-*d*_6_) δ 9.92 (d, *J* = 57.5 Hz, 1H), 7.60 (dd, *J* = 5.0, 1.2 Hz, 1H), 7.54–7.47 (m, 3H), 7.35 (dd, *J* = 12.8, 6.1, 4.7, 3.0 Hz, 4H), 7.31–7.21 (m, 3H), 6.83 (dd, *J* = 5.0, 3.8 Hz, 1H), 6.46–6.31 (m, 1H), 4.68 (q, *J* = 20.5, 8.4, 6.8 Hz, 1H), 3.53 (dd, *J* = 24.1, 15.6 Hz, 2H), 3.27 (dt, *J* = 9.2, 6.2 Hz, 4H), 2.99 (q, *J* = 8.7, 7.0 Hz, 2H), 2.37–2.15 (m, 1H), 2.14–2.05 (m, 1H), 2.07–1.58 (m, 4H). ^13^C NMR (100 MHz, DMSO-*d*_6_) δ 161.41, 139.72, 139.16, 137.40, 132.08, 131.87, 131.33, 131.09, 130.13, 130.10, 129.61, 129.24, 129.11, 129.08, 127.42, 127.30, 57.80, 57.61, 57.10, 53.66, 50.74, 50.54, 30.81, 30.26, 30.14, 29.26, 28.01, 20.13. HRMS *m*/*z*: calcd for C_25_H_29_N_2_OS [M + H]^+^: 405.2001; found, 405.1995. Mp 163.2–165.8 °C. The purity of the compound was checked by HPLC (*R*_t_ = 2.875 min) and was found to be 97.75% pure.

#### *N*-(1-Allylazepan-4-yl)-*N*-phenylfuran-3-carboxamide Hydrogen Chloride (**18**)

The title compound was prepared following the general procedure as a white solid in 49% yield. Hydrochloride salt: ^1^H NMR (400 MHz, DMSO-*d*_6_) δ 9.61 (d, *J* = 68.8 Hz, 1H), 7.63 (d, *J* = 1.7 Hz, 1H), 7.57–7.41 (m, 3H), 7.41–7.20 (m, 2H), 6.32 (dt, *J* = 3.4, 1.6 Hz, 1H), 5.99–5.82 (m, 1H), 5.59–5.37 (m, 3H), 4.74–4.56 (m, 1H), 3.72 (t, *J* = 5.7 Hz, 2H), 3.27–3.12 (m, 2H), 3.09 (m, *J* = 10.7, 3.5, 3.1 Hz, 1H), 2.97–2.80 (m, 1H), 2.37–2.10 (m, 2H), 2.04–1.76 (m, 3H), 1.72–1.57 (m, 1H). ^13^C NMR (100 MHz, DMSO-*d*_6_): 164.5, 140.1, 137.9, 137.6, 130.8, 129.7, 129.6, 129.1, 128.5, 128.4, 127.3, 125.8, 56.6, 49.9, 49.0, 47.3, 38.6, 36.2, 35.7, 32.3, 30.7, 29.9. HRMS *m*/*z*: calcd for C_26_H_31_N_2_OS [M + H]^+^: 325.1916; found, 325.1897. Mp 248.7–249.5 °C. The purity of the compound was checked by HPLC (*R*_t_ = 2.657 min) and was found to be 98.94% pure.

#### *N*-(1-(Cyclopropylmethyl)­azepan-4-yl)-*N*-phenylfuran-3-carboxamide Hydrogen Chloride (**19**)

The title compound was prepared following the general procedure as a white solid in 76%yield. Hydrochloride salt: ^1^H NMR (400 MHz, DMSO-*d*_6_): 9.92 (s, 1H), 7.44–7.31 (m, 6H), 7.28–7.24 (m, 4H), 7.21–7.19 (d, 2H), 6.81–6.80­(d, 1H), 4.95 (p, 1H), 3.47–3.44 (m, 1H), 3.32–3.29 (m, 1H), 3.23–3.19 (m, 2H), 3.01–2.93 (m, 3H), 2.76–2.73 (m, 1H), 2.33–2.31 (m, 1H), 2.12–2.11 (m, 1H), 2.04–2.01 (m, 1H), 1.79–1.74 (m, 2H), 1.71–1.60 (m, 2H), 1.34–1.31 (m, 1H). ^13^C NMR (100 MHz, DMSO-*d*_6_): 164.5, 140.1, 137.9, 137.6, 130.8, 129.7, 129.6, 129.1, 128.5, 128.4, 127.3, 125.8, 56.6, 49.9, 49.0, 47.3, 38.6, 36.2, 35.7, 32.3, 30.7, 29.9. HRMS *m*/*z*: calcd for C_24_H_28_N_3_O [M + H]^+^: 339.2073; found, 339.2062. Mp 157.8–159.2 °C. The purity of the compound was checked by HPLC (*R*_t_ = 2.662 min) and was found to be 98.33% pure.

#### *N*-(1-(Cyclobutylmethyl)­azepan-4-yl)-*N*-phenylfuran-3-carboxamide Hydrogen Chloride (**20**)

The title compound was prepared following the general procedure as a light-yellow solid in 94% yield. Hydrochloride salt: ^1^H NMR (400 MHz, DMSO-*d*_6_): 10.34 (s, 1H), 7.44–7.31 (m, 6H), 7.28–7.24 (m, 4H), 7.21–7.19 (d, 2H), 6.81–6.80­(d, 1H), 4.95 (p, 1H), 3.47–3.44 (m, 1H), 3.32–3.29 (m, 1H), 3.23–3.19 (m, 2H), 3.01–2.93 (m, 3H), 2.76–2.73 (m, 1H), 2.33–2.31 (m, 1H), 2.12–2.11 (m, 1H), 2.04–2.01 (m, 1H), 1.79–1.74 (m, 2H), 1.71–1.60 (m, 2H), 1.34–1.31 (m, 1H). ^13^C NMR (100 MHz, DMSO-*d*_6_): 164.5, 140.1, 137.9, 137.6, 130.8, 129.7, 129.6, 129.1, 128.5, 128.4, 127.3, 125.8, 56.6, 49.9, 49.0, 47.3, 38.6, 36.2, 35.7, 32.3, 30.7, 29.9. HRMS *m*/*z*: calcd for C_24_H_28_N_3_O [M + H]^+^: 353.2229; found, 353.2214. Mp 218.9–220.2 °C. The purity of the compound was checked by HPLC (*R*_t_ = 2.772 min) and was found to be 99.70% pure.

#### *N*-(1-(Cyclopentylmethyl)­azepan-4-yl)-*N*-phenylfuran-3-carboxamide Hydrogen Chloride (**21**)

The title compound was prepared following the general procedure as an off-white solid in 54% yield. Hydrochloride salt: ^1^H NMR (400 MHz, DMSO-*d*_6_): 9.92 (s, 1H), 7.44–7.31 (m, 6H), 7.28–7.24 (m, 4H), 7.21–7.19 (d, 2H), 6.81–6.80­(d, 1H), 4.95 (p, 1H), 3.47–3.44 (m, 1H), 3.32–3.29 (m, 1H), 3.23–3.19 (m, 2H), 3.01–2.93 (m, 3H), 2.76–2.73 (m, 1H), 2.33–2.31 (m, 1H), 2.12–2.11 (m, 1H), 2.04–2.01 (m, 1H), 1.79–1.74 (m, 2H), 1.71–1.60 (m, 2H), 1.34–1.31 (m, 1H). ^13^C NMR (100 MHz, DMSO-*d*_6_): 164.5, 140.1, 137.9, 137.6, 130.8, 129.7, 129.6, 129.1, 128.5, 128.4, 127.3, 125.8, 56.6, 49.9, 49.0, 47.3, 38.6, 36.2, 35.7, 32.3, 30.7, 29.9. HRMS *m*/*z*: calcd for C_26_H_29_N_2_O [M + H]^+^: 367.2386; found, 367.2380. Mp 268.9–270.4 °C. The purity of the compound was checked by HPLC (*R*_t_ = 2.790 min) and was found to be 97.82% pure.

#### *N*-(1-(Cyclohexylmethyl)­azepan-4-yl)-*N*-phenylfuran-3-carboxamide Hydrogen Chloride (**22**)

The title compound was prepared following the general procedure as white powder in 74% yield. Hydrochloride salt: ^1^H NMR (400 MHz, DMSO-*d*_6_): 9.92 (s, 1H), 7.44–7.31 (m, 6H), 7.28–7.24 (m, 4H), 7.21–7.19 (d, 2H), 6.81–6.80­(d, 1H), 4.95 (p, 1H), 3.47–3.44 (m, 1H), 3.32–3.29 (m, 1H), 3.23–3.19 (m, 2H), 3.01–2.93 (m, 3H), 2.76–2.73 (m, 1H), 2.33–2.31 (m, 1H), 2.12–2.11 (m, 1H), 2.04–2.01 (m, 1H), 1.79–1.74 (m, 2H), 1.71–1.60 (m, 2H), 1.34–1.31 (m, 1H). ^13^C NMR (100 MHz, DMSO-*d*_6_): 164.5, 140.1, 137.9, 137.6, 130.8, 129.7, 129.6, 129.1, 128.5, 128.4, 127.3, 125.8, 56.6, 49.9, 49.0, 47.3, 38.6, 36.2, 35.7, 32.3, 30.7, 29.9. HRMS *m*/*z*: calcd for C_27_H_31_N_2_O [M + H]^+^: 381.2542; found, 381.2552. Mp 168.9–170.6 °C. The purity of the compound was checked by HPLC (*R*_t_ = 2.925 min) and was found to be 99.13% pure.

#### *N*-(1-Benzylazepan-4-yl)-*N*-phenylfuran-3-carboxamide Hydrogen Chloride (**23**)

The title compound was prepared following the general procedure as a white sold in 90% yield. Hydrochloride salt: ^1^H NMR (400 MHz, DMSO-*d*_6_): 9.92 (s, 1H), 7.44–7.31 (m, 6H), 7.28–7.24 (m, 4H), 7.21–7.19 (d, 2H), 6.81–6.80­(d, 1H), 4.95 (p, 1H), 3.47–3.44 (m, 1H), 3.32–3.29 (m, 1H), 3.23–3.19 (m, 2H), 3.01–2.93 (m, 3H), 2.76–2.73 (m, 1H), 2.33–2.31 (m, 1H), 2.12–2.11 (m, 1H), 2.04–2.01 (m, 1H), 1.79–1.74 (m, 2H), 1.71–1.60 (m, 2H), 1.34–1.31 (m, 1H). ^13^C NMR (100 MHz, DMSO-*d*_6_): 164.5, 140.1, 137.9, 137.6, 130.8, 129.7, 129.6, 129.1, 128.5, 128.4, 127.3, 125.8, 56.6, 49.9, 49.0, 47.3, 38.6, 36.2, 35.7, 32.3, 30.7, 29.9. HRMS *m*/*z*: calcd for C_28_H_33_N_2_O [M + H]^+^: 375.2073; found, 375.2069. Mp 253.4–255.8 °C. The purity of the compound was checked by HPLC (*R*_t_ = 2.765 min) and was found to be 99.79% pure.

#### *N*-(1-Phenethylazepan-4-yl)-*N*-phenylfuran-3-carboxamide Hydrogen Chloride (**24**)

The title compound was prepared following the general procedure as a white solid in 70% yield. Hydrochloride salt: ^1^H NMR (400 MHz, DMSO-*d*_6_): 9.92 (s, 1H), 7.44–7.31 (m, 6H), 7.28–7.24 (m, 4H), 7.21–7.19 (d, 2H), 6.81–6.80­(d, 1H), 4.95 (p, 1H), 3.47–3.44 (m, 1H), 3.32–3.29 (m, 1H), 3.23–3.19 (m, 2H), 3.01–2.93 (m, 3H), 2.76–2.73 (m, 1H), 2.33–2.31 (m, 1H), 2.12–2.11 (m, 1H), 2.04–2.01 (m, 1H), 1.79–1.74 (m, 2H), 1.71–1.60 (m, 2H), 1.34–1.31 (m, 1H). ^13^C NMR (100 MHz, DMSO-*d*_6_): 164.5, 140.1, 137.9, 137.6, 130.8, 129.7, 129.6, 129.1, 128.5, 128.4, 127.3, 125.8, 56.6, 49.9, 49.0, 47.3, 38.6, 36.2, 35.7, 32.3, 30.7, 29.9. HRMS *m*/*z*: calcd for C_24_H_27_N_2_O_2_[M + H]^+^: 389.2229; found, 389.2230. Mp 162.8–164.1 °C. The purity of the compound was checked by HPLC (*R*_t_ = 2.873 min) and was found to be 97.95% pure.

#### *N*-(1-Allylazepan-4-yl)-*N*-phenyl-1*H*-pyrrole-2-carboxamide Hydrogen Chloride (**25**)

The title compound was prepared following the general procedure as a white powder in 26.2% yield. ^1^H NMR (400 MHz, DMSO-*d*_6_) δ 11.38 (s, 1H), 10.47 (s, 1H), 7.50 (p, *J* = 3.6, 3.2 Hz, 3H), 7.30 (m, *J* = 9.5, 8.0, 3.5 Hz, 2H), 6.75 (q, *J* = 2.3 Hz, 1H), 5.96 (m, *J* = 17.2, 10.3, 7.0, 3.2 Hz, 1H), 5.73 (dd, *J* = 3.9, 2.4 Hz, 1H), 5.56–5.42 (m, 2H), 4.70 (m, *J* = 24.5, 8.9, 7.8, 4.1 Hz, 1H), 4.48 (m, *J* = 3.9, 2.0 Hz, 1H), 3.69 (dt, *J* = 9.8, 4.5 Hz, 2H), 3.32 (d, *J* = 3.3 Hz, 1H), 3.18–3.02 (m, 2H), 2.89–2.77 (m, 1H), 2.16 (dt, *J* = 15.1, 5.5 Hz, 1H), 2.11–1.97 (m, 2H), 1.92–1.74 (m, 2H), 1.72–1.52 (m, 1H). ^13^C NMR (100 MHz, DMSO-*d*_6_) δ 160.60, 131.37, 131.14, 129.94, 129.88, 129.18, 129.15, 128.37, 125.42, 125.37, 125.16, 121.88, 113.25, 109.07, 65.38, 59.11, 58.97, 55.95, 53.83, 53.05, 50.49, 31.63, 30.98, 29.33, 28.20, 20.03, 15.63. HRMS *m*/*z*: calcd for C_20_H_25_N_3_O: [M + H]^+^: 324.2076; found: 324.2081. Mp 93.8–95.0 °C. The purity of the compound was checked by HPLC (*R*_t_ = 2.670 min) and was found to be 100% pure.

#### *N*-(1-(Cyclopropylmethyl)­azepan-4-yl)-*N*-phenyl-1*H*-pyrrole-2-carboxamide Hydrogen Chloride (**26**)

The title compound was prepared following the general procedure as a white powder in 45.7% yield. ^1^H NMR (400 MHz, DMSO-*d*_6_) δ 11.38 (s, 1H), 10.04 (s, 1H), 7.50 (dt, *J* = 5.4, 2.7 Hz, 3H), 7.31 (dt, *J* = 6.5, 2.9 Hz, 2H), 6.75 (td, *J* = 2.7, 1.4 Hz, 1H), 5.73 (dd, *J* = 4.1, 2.2 Hz, 1H), 4.79–4.64 (m, 1H), 4.48 (m, *J* = 3.9, 1.8 Hz, 1H), 3.59–3.47 (m, 1H), 3.25–3.11 (m, 2H), 2.96 (dt, *J* = 7.4, 5.0 Hz, 2H), 2.37–2.13 (m, 1H), 2.11–1.95 (m, 2H), 1.84 (m, *J* = 17.8, 8.9 Hz, 2H), 1.73–1.52 (m, 1H), 0.60 (m, *J* = 9.3, 4.6, 2.7 Hz, 2H), 0.37 (m, *J* = 6.2, 3.8, 3.0 Hz, 2H). ^13^C NMR (100 MHz, DMSO-*d*_6_) δ 160.62, 140.28, 131.35, 129.90, 129.19, 125.39, 121.88, 113.25, 109.07, 65.38, 61.05, 60.87, 53.24, 50.57, 31.21, 29.17, 27.87, 21.68, 19.77, 15.64, 6.21, 6.16, 4.72, 4.59. HRMS *m*/*z*: calcd for C_21_H_27_N_3_O: [M + H]^+^: 338.2232; found: 338.2229. Mp 151.2–152.8 °C. The purity of the compound was checked by HPLC (*R*_t_ = 2.718 min) and was found to be 99.08% pure.

#### *N*-(1-(Cyclobutylmethyl)­azepan-4-yl)-*N*-phenyl-1*H*-pyrrole-2-carboxamide Hydrogen Chloride (**27**)

The title compound was prepared following the general procedure as a white powder in 32.9% yield. ^1^H NMR (400 MHz, DMSO-*d*_6_) δ 11.37 (s, 1H), 9.88 (s, 1H), 7.59–7.42 (m, 3H), 7.30 (m, *J* = 5.3, 3.7, 3.1, 1.7 Hz, 2H), 6.76 (m, *J* = 2.4, 1.1 Hz, 1H), 5.78–5.68 (m, 1H), 4.79–4.60 (m, 1H), 4.48 (m, *J* = 3.9, 2.5, 1.4 Hz, 1H), 3.29–3.23 (m, 1H), 3.20–2.99 (m, 5H), 2.86–2.63 (m, 2H), 2.18–1.97 (m, 4H), 1.92–1.69 (m, 6H), 1.69–1.49 (m, 1H). ^13^C NMR (100 MHz, DMSO-*d*_6_) δ 160.60, 131.34, 131.21, 129.89, 129.18, 125.38, 121.88, 113.25, 109.07, 61.66, 53.62, 50.81, 31.15, 30.78, 29.17, 27.29, 27.27, 27.23, 26.98, 21.65, 19.78, 18.58, 18.55. HRMS M/Z: calcd for C_22_H_29_N_3_O: [M + H]^+^: 352.2389; found: 352.2397. Mp 99.6–101.2 °C. The purity of the compound was checked by HPLC (*R*_t_ = 2.775 min) and was found to be 99.24% pure.

#### *N*-(1-(Cyclopentylmethyl)­azepan-4-yl)-*N*-phenyl-1*H*-pyrrole-2-carboxamide Hydrogen Chloride (**28**)

The title compound was prepared following the general procedure as a white powder in 10.5% yield. ^1^H NMR (400 MHz, DMSO-*d*_6_) δ 11.38 (s, 1H), 9.87 (s, 1H), 7.57–7.46 (m, 3H), 7.30 (m, *J* = 5.2, 3.9, 2.5 Hz, 2H), 6.75 (m, *J* = 2.5, 1.1 Hz, 1H), 5.73 (dt, *J* = 3.8, 2.4 Hz, 1H), 4.78–4.63 (m, 1H), 4.48 (m, *J* = 3.8, 2.4, 1.2 Hz, 1H), 3.53–3.37 (m, 2H), 3.15 (dd, *J* = 10.5, 5.7 Hz, 2H), 3.09–2.94 (m, 2H), 2.32–1.93 (m, 4H), 1.91–1.73 (m, 4H), 1.68–1.42 (m, 5H), 1.29–1.13 (m, 2H). ^13^C NMR (100 MHz, DMSO-*d*_6_) δ 160.66, 160.60, 140.37, 140.25, 131.35, 131.27, 129.91, 129.19, 125.39, 121.88, 113.24, 109.07, 61.57, 61.38, 56.18, 54.11, 53.79, 51.12, 50.57, 35.28, 32.06, 31.52, 31.35, 31.26, 31.24, 31.08, 28.96, 27.28, 25.09, 25.03, 21.31, 19.24. HRMS *m*/*z*: calcd for C_23_H_31_N_3_O: [M + H]^+^: 366.2547; found: 366.2522. Mp 158.6–160.3 °C. The purity of the compound was checked by HPLC (*R*_t_ = 2.840 min) and was found to be 100% pure.

#### *N*-(1-(Cyclohexylmethyl)­azepan-4-yl)-*N*-phenyl-1*H*-pyrrole-2-carboxamide Hydrogen Chloride (**29**)

The title compound was prepared following the general procedure as a white powder in 20.3% yield. ^1^H NMR (400 MHz, DMSO-*d*_6_) δ 11.38 (d, *J* = 3.5 Hz, 1H), 9.74 (s, 1H), 7.58–7.45 (m, 3H), 7.35–7.24 (m, 2H), 6.75 (m, *J* = 2.6, 1.2 Hz, 1H), 5.80–5.70 (m, 1H), 4.80–4.62 (m, 1H), 4.47 (m, *J* = 3.8, 1.8 Hz, 1H), 3.52–3.43 (m, 1H), 3.15 (d, *J* = 10.3 Hz, 2H), 2.88 (m, *J* = 20.0, 6.7 Hz, 3H), 2.30–1.93 (m, 3H), 1.89–1.72 (m, 4H), 1.72–1.45 (m, 5H), 1.29–1.10 (m, 3H), 0.99–0.84 (m, 2H). ^13^C NMR (100 MHz, DMSO-*d*_6_) δ 160.60, 131.35, 129.91, 129.19, 125.40, 121.88, 113.24, 109.07, 62.59, 62.42, 56.39, 51.35, 50.82, 33.00, 32.10, 31.63, 30.93, 30.83, 30.76, 28.87, 27.11, 25.95, 25.46, 25.42, 21.23, 19.04. HRMS *m*/*z*: calcd for C_24_H_33_N_3_O: [M + H]^+^: 380.2702; found: 380.2690. Mp 197.2–198.6 °C. The purity of the compound was checked by HPLC (*R*_t_ = 2.938 min) and was found to be 99.46% pure.

#### *N*-(1-Benzylazepan-4-yl)-*N*-phenyl-1*H*-pyrrole-2-carboxamide Hydrogen Chloride (**30**)

The title compound was prepared following the general procedure as a pale-yellow oil in 40.6% yield. ^1^H NMR (400 MHz, DMSO-*d*_6_) δ 11.38 (d, *J* = 3.5 Hz, 1H), 9.74 (s, 1H), 7.58–7.45 (m, 3H), 7.35–7.24 (m, 2H), 6.75 (m, *J* = 2.6, 1.2 Hz, 1H), 5.80–5.70 (m, 1H), 4.80–4.62 (m, 1H), 4.47 (m, *J* = 3.8, 1.8 Hz, 1H), 3.52–3.43 (m, 1H), 3.15 (d, *J* = 10.3 Hz, 2H), 2.88 (m, *J* = 20.0, 6.7 Hz, 3H), 2.30–1.93 (m, 3H), 1.89–1.72 (m, 4H), 1.72–1.45 (m, 5H), 1.29–1.10 (m, 3H), 0.99–0.84 (m, 2H). ^13^C NMR (100 MHz, DMSO-*d*_6_) δ 160.60, 131.35, 129.91, 129.19, 125.40, 121.88, 113.24, 109.07, 62.59, 62.42, 56.39, 51.35, 50.82, 33.00, 32.10, 31.63, 30.93, 30.83, 30.76, 28.87, 27.11, 25.95, 25.46, 25.42, 21.23, 19.04. HRMS *m*/*z*: calcd for C_24_H_27_N_3_O: [M + H]^+^: 374.2232; found: 374.2226. The purity of the compound was checked by HPLC (*R*_t_ = 2.842 min) and was found to be 99.14% pure.

#### *N*-(1-Phenethylazepan-4-yl)-*N*-phenyl-1*H*-pyrrole-2-carboxamide Hydrogen Chloride (**31**)

The title compound was prepared following the general procedure as a white powder in 49.5% yield. ^1^H NMR (400 MHz, DMSO-*d*_6_) δ 11.45–11.29 (m, 1H), 10.17 (s, 1H), 7.57–7.42 (m, 3H), 7.38–7.17 (m, 7H), 6.76 (td, *J* = 2.7, 1.4 Hz, 1H), 5.79–5.70 (m, 1H), 4.73 (dt, *J* = 16.6, 11.3 Hz, 1H), 4.49 (m, *J* = 3.8, 1.8 Hz, 1H), 3.58–3.39 (m, 2H), 3.26 (m, *J* = 17.2, 8.5, 4.5 Hz, 4H), 3.06–2.90 (m, 2H), 2.36–2.13 (m, 1H), 2.12–1.99 (m, 2H), 1.97–1.74 (m, 2H), 1.72–1.50 (m, 1H). ^13^C NMR (100 MHz, DMSO-*d*_6_) δ 137.49, 131.40, 129.89, 129.22, 129.15, 129.10, 129.07, 127.27, 121.89, 113.26, 109.08, 65.37, 57.77, 53.59, 50.77, 31.10, 30.12, 20.07, 15.63. HRMS *m*/*z*: calcd for C_25_H_29_N_3_O: [M + H]^+^: 388.2389; found: 388.2374. Mp 138.8–139.5 °C. The purity of the compound was checked by HPLC (*R*_t_ = 2.878 min) and was found to be 99.72% pure.

#### *N*-(1-Allylazepan-4-yl)-*N*-phenyl-1*H*-pyrrole-3-carboxamide Hydrogen Chloride (**32**)

The title compound was prepared following the general procedure as a pale-yellow oil in 17.7% yield. ^1^H NMR (400 MHz, DMSO-*d*_6_) δ 10.92 (s, 1H), 10.61 (d, *J* = 45.7 Hz, 1H), 7.46 (dd, *J* = 7.6, 3.4 Hz, 4H), 7.25 (m, *J* = 9.3, 4.8, 2.4 Hz, 4H), 6.47 (q, *J* = 2.5 Hz, 1H), 6.10–5.87 (m, 2H), 5.60 (p, *J* = 2.4 Hz, 1H), 5.55–5.40 (m, 3H), 4.78–4.49 (m, 2H), 3.69 (t, *J* = 5.9 Hz, 3H), 3.19–3.02 (m, 3H), 2.88–2.76 (m, 1H), 2.40–2.25 (m, 1H), 2.20–2.08 (m, 1H), 2.07–1.94 (m, 3H), 1.90–1.78 (m, 3H), 1.64 (q, *J* = 9.6, 7.8 Hz, 2H). ^13^C NMR (100 MHz, DMSO-*d*_6_) δ 141.02, 131.35, 131.02, 129.75, 129.68, 128.71, 128.63, 128.45, 128.41, 125.10, 122.53, 119.30, 117.77, 110.30, 110.26, 59.07, 58.99, 52.96, 50.69, 50.05, 31.04, 29.47, 28.32, 22.01, 20.06. HRMS *m*/*z*: calcd for C_20_H_25_N_3_O: [M + H]^+^: 324.2076; found: 324.2083. The purity of the compound was checked by HPLC (*R*_t_ = 2.580 min) and was found to be 99.72% pure.

#### *N*-(1-(Cyclopropylmethyl)­azepan-4-yl)-*N*-phenyl-1*H*-pyrrole-3-carboxamide Hydrogen Chloride (**33**)

The title compound was prepared following the general procedure as a white powder in 30.5% yield. ^1^H NMR (400 MHz, DMSO-*d*_6_) δ 10.72 (s, 1H), 7.27 (m, *J* = 4.2, 1.9 Hz, 3H), 7.09–6.98 (m, 2H), 6.29 (q, *J* = 2.4 Hz, 1H), 5.87 (dt, *J* = 3.4, 1.8 Hz, 1H), 5.43 (td, *J* = 2.6, 1.5 Hz, 1H), 4.60–4.48 (m, 1H), 2.74 (t, *J* = 44.4 Hz, 4H), 2.42 (d, *J* = 6.7 Hz, 2H), 1.94–1.73 (m, 2H), 1.69–1.36 (m, 4H), 0.72 (q, *J* = 7.3, 6.1 Hz, 1H), 0.32 (h, *J* = 4.6 Hz, 2H), 0.00 (q, *J* = 4.7 Hz, 2H). ^13^C NMR (100 MHz, DMSO-*d*_6_) δ 164.18, 141.34, 131.34, 129.55, 128.53, 122.43, 119.53, 117.67, 110.31, 62.20, 56.19, 54.36, 51.19, 49.06, 32.06, 7.90, 4.41, 4.22. HRMS *m*/*z*: calcd for C_21_H_27_N_3_O: [M + H]^+^: 338.2232; found: 338.2192. Mp 84.4–85.5 °C. The purity of the compound was checked by HPLC (*R*_t_ = 2.580 min) and was found to be 99.72% pure.

#### *N*-(1-(Cyclobutylmethyl)­azepan-4-yl)-*N*-phenyl-1*H*-pyrrole-3-carboxamide Hydrogen Chloride (**34**)

The title compound was prepared following the general procedure as a pale-yellow oil in 40.7% yield. ^1^H NMR (400 MHz, DMSO-*d*_6_) δ 10.89 (s, 1H), 9.59 (d, *J* = 47.9 Hz, 1H), 7.45 (m, *J* = 4.0, 2.1 Hz, 3H), 7.24 (m, *J* = 6.4, 4.4, 2.1 Hz, 2H), 6.47 (q, *J* = 2.5 Hz, 1H), 6.05 (dt, *J* = 3.2, 1.6 Hz, 1H), 5.59 (td, *J* = 2.6, 1.5 Hz, 1H), 4.64 (d, *J* = 29.4 Hz, 2H), 3.14–3.00 (m, 5H), 2.75–2.65 (m, 1H), 2.12–1.97 (m, 5H), 1.90–1.71 (m, 8H). ^13^C NMR (100 MHz, DMSO-*d*_6_) δ 131.32, 129.72, 128.70, 122.55, 119.32, 117.79, 110.29, 72.74, 60.72, 49.06, 30.79, 27.24, 18.56. HRMS *m*/*z*: calcd for C_22_H_29_N_3_O: [M + H]^+^: 352.2389; found: 352.2397. The purity of the compound was checked by HPLC (*R*_t_ = 2.633 min) and was found to be 97.16% pure.

#### *N*-(1-(Cyclopentylmethyl)­azepan-4-yl)-*N*-phenyl-1*H*-pyrrole-3-carboxamide Hydrogen Chloride (**35**)

The title compound was prepared following the general procedure as a pale-yellow oil in 20.0% yield. ^1^H NMR (400 MHz, DMSO-*d*_6_) δ 10.90 (s, 1H), 9.63 (d, *J* = 42.0 Hz, 1H), 7.54–7.39 (m, *J* = 2.2 Hz, 3H), 7.24 (dt, *J* = 7.0, 2.3 Hz, 2H), 6.47 (q, *J* = 2.4 Hz, 1H), 6.04 (dt, *J* = 3.5, 1.7 Hz, 1H), 5.59 (t, *J* = 2.2 Hz, 1H), 4.74–4.56 (m, 1H), 3.15 (dd, *J* = 10.4, 4.9 Hz, 2H), 3.03 (m, *J* = 18.6, 7.0, 4.1 Hz, 3H), 2.18 (t, *J* = 7.8 Hz, 1H), 2.03 (d, *J* = 8.1 Hz, 2H), 1.80 (dd, *J* = 14.8, 7.6 Hz, 4H), 1.67–1.44 (m, 6H), 1.22–1.13 (m, 3H). ^13^C NMR (100 MHz, DMSO-*d*_6_) δ 131.33, 129.71, 128.72, 122.54, 110.30, 61.62, 51.33, 35.29, 31.28, 31.20, 25.08, 25.03. HRMS *m*/*z*: calcd for C_23_H_31_N_3_O: [M + H]^+^: 366.2545; found: 366.2527. The purity of the compound was checked by HPLC (*R*_t_ = 2.672 min) and was found to be 97.52% pure.

#### *N*-(1-(Cyclohexylmethyl)­azepan-4-yl)-*N*-phenyl-1*H*-pyrrole-3-carboxamide Hydrogen Chloride (**36**)

The title compound was prepared following the general procedure as a pale-yellow oil in 20.3% yield. ^1^H NMR (400 MHz, DMSO-*d*_6_) δ 10.89 (s, 1H), 9.28 (d, *J* = 52.3 Hz, 1H), 7.46 (m, *J* = 4.3, 2.2 Hz, 3H), 7.24 (dt, *J* = 6.7, 1.5 Hz, 3H), 6.47 (q, *J* = 2.4 Hz, 1H), 6.04 (h, *J* = 1.6 Hz, 1H), 5.59 (dd, *J* = 3.0, 1.6 Hz, 1H), 4.75–4.57 (m, 1H), 3.12 (d, *J* = 15.4 Hz, 2H), 2.97–2.79 (m, 3H), 2.15–1.93 (m, 3H), 1.89–1.55 (m, 11H), 1.18 (dd, *J* = 14.5, 4.6 Hz, 2H), 0.94–0.86 (m, 2H). ^13^C NMR (100 MHz, DMSO-*d*_6_) δ 131.33, 129.73, 128.72, 122.55, 119.32, 117.79, 110.30, 62.66, 62.46, 54.27, 32.99, 30.77, 30.60, 25.95, 25.41. HRMS *m*/*z*: calcd for C_24_H_33_N_3_O [M + H]^+^: 380.2702; found, 380.2683. The purity of the compound was checked by HPLC (*R*_t_ = 2.735 min) and was found to be 97.49% pure.

#### *N*-(1-Benzylazepan-4-yl)-*N*-phenyl-1*H*-pyrrole-3-carboxamide Hydrogen Chloride (**37**)

The title compound was prepared following the general procedure as a pale-yellow oil in 20.6% yield. ^1^H NMR (400 MHz, DMSO-*d*_6_) δ 10.89 (s, 1H), 9.28 (d, *J* = 52.3 Hz, 1H), 7.46 (m, *J* = 4.3, 2.2 Hz, 3H), 7.24 (dt, *J* = 6.7, 1.5 Hz, 3H), 6.47 (q, *J* = 2.4 Hz, 1H), 6.04 (h, *J* = 1.6 Hz, 1H), 5.59 (dd, *J* = 3.0, 1.6 Hz, 1H), 4.75–4.57 (m, 1H), 3.12 (d, *J* = 15.4 Hz, 2H), 2.97–2.79 (m, 3H), 2.15–1.93 (m, 3H), 1.89–1.55 (m, 11H), 1.18 (dd, *J* = 14.5, 4.6 Hz, 2H), 0.94–0.86 (m, 2H). ^13^C NMR (100 MHz, DMSO-*d*_6_) δ 131.33, 129.73, 128.72, 122.55, 119.32, 117.79, 110.30, 62.66, 62.46, 54.27, 32.99, 30.77, 30.60, 25.95, 25.41. HRMS *m*/*z*: calcd for C_24_H_27_N_3_O [M + H]^+^: 374.2232; found, 374.2226. The purity of the compound was checked by HPLC (*R*_t_ = 2.637 min) and was found to be 97.24% pure.

#### *N*-(1-Phenethylazepan-4-yl)-*N*-phenyl-1*H*-pyrrole-3-carboxamide Hydrogen Chloride (**38**)

The title compound was prepared following the general procedure as a pale-yellow oil in 17.9% yield. ^1^H NMR (400 MHz, DMSO-*d*_6_) δ 10.89 (s, 1H), 10.14 (d, *J* = 48.4 Hz, 1H), 7.45 (t, *J* = 5.3 Hz, 4H), 7.29 (dt, *J* = 26.5, 6.4 Hz, 8H), 6.47 (s, 1H), 6.05 (d, *J* = 5.3 Hz, 1H), 5.59 (s, 1H), 4.67 (d, *J* = 35.2 Hz, 2H), 3.24 (s, 5H), 2.99 (t, *J* = 7.9 Hz, 3H), 2.05 (d, *J* = 21.6 Hz, 2H), 1.86 (s, 2H), 1.61 (d, *J* = 16.7 Hz, 2H). ^13^C NMR (100 MHz, DMSO-*d*_6_) δ 164.29, 137.52, 131.39, 131.11, 129.74, 129.70, 129.23, 129.10, 129.07, 128.72, 128.65, 127.26, 122.55, 119.38, 119.32, 117.78, 110.32, 57.71, 53.51, 50.92, 31.16, 30.22, 30.12, 29.53, 20.10. HRMS *m*/*z*: calcd for C_25_H_29_N_3_O [M + H]^+^: 388.2389; found, 388.2401. The purity of the compound was checked by HPLC (*R*_t_ = 2.677 min) and was found to be 100% pure.

#### *N*-(1-Allylazepan-4-yl)-*N*-phenylthiophene-3-carboxamide Hydrochloride (**39**)

The title compound was prepared following the general procedure as a dark oil in 38% yield. ^1^H NMR (400 MHz, DMSO-*d*_6_): 10.59 (d, 1H, *J* = 46.01 Hz), 7.39–7.34 (m, 3H), 7.29–7.27 (m, 1H), 7.24–7.21 (m, 3H), 6.78–6.77 (m, 1H), 6.00–5.94 (m, 1H), 5.53–5.46 (m, 2H), 4.69–4.55 (m, 1H), 3.70–3.68 (m, 2H), 3.46–3.42 (m, 1H), 3.21–3.09 (m, 3H), 2.18–2.13 (m, 1H), 2.11–2.04 (m, 2H), 1.89–1.83 (m, 2H), 1.74–1.68 (m, 1H). ^13^C NMR (100 MHz, DMSO-*d*_6_): 164.31, 141.20, 140.56, 137.95, 137.88, 130.69, 130.37, 129.70, 129.62, 129.57, 129.47, 128.54, 128.43, 128.41, 128.35, 125.68, 125.16, 59.10, 59.02, 57.47, 56.99, 53.83, 52.99, 50.63, 50.01, 31.40, 30.81, 29.26, 28.14, 21.99, 20.06. HRMS *m*/*z*: calcd for C_20_H_24_N_2_OS [M + H]^+^: 341.1688; found, 341.1680. The purity of the compound was checked by HPLC (*R*_t_ = 2.708 min) and was found to be 98.14% pure.

#### *N*-(1-(Cyclopropylmethyl)­azepan-4-yl)-*N*-phenylthiophene-3-carboxamide Hydrochloride (**40**)

The title compound was prepared following the general procedure as a pale-yellow oil in 21% yield. ^1^H NMR (400 MHz, DMSO-*d*_6_): 10.12 (d, 1H, *J* = 27.41 Hz), 7.41–7.34 (m, 3H), 7.30–7.28 (m, 1H), 7.24–7.22 (m, 3H), 6.79–6.77­(m, 1H), 4.66–4.57 (m, 1H), 3.24–3.17 (m, 2H), 2.98–2.89 (m, 2H), 2.19–2.05 (m, 3H), 1.89–1.69 (m, 3H), 1.24–1.06 (m, 2H), 0.88–0.86 (m, 1H), 0.62–0.59 (m, 2H), 0.38–0.37 (m, 2H).^13^C NMR (100 MHz, DMSO-*d*_6_): 164.32, 140.65, 137.94, 137.89, 130.67, 130.44, 129.68, 129.65, 129.49, 128.55, 128.43, 125.67, 61.04, 60.91, 57.14, 53.68, 50.71, 50.09, 31.57, 31.02, 29.08, 27.82, 21.74, 19.82, 6.22. HRMS *m*/*z*: calcd for C_21_H_26_N_2_OS [M + H]^+^: 355.1844; found, 355.1851. The purity of the compound was checked by HPLC (*R*_t_ = 2.750 min) and was found to be 99.37% pure.

#### *N*-(1-(Cyclobutylmethyl)­azepan-4-yl)-*N*-phenylthiophene-3-carboxamide Hydrochloride (**41**)

The title compound was prepared following the general procedure as a pale-yellow oil in 33% yield. ^1^H NMR (400 MHz, DMSO-*d*_6_): 9.97 (d, 1H, *J* = 33.80 Hz), 7.40–7.34 (m, 3H), 7.29–7.27 (m, 1H), 7.24–7.20 (m, 3H), 6.78–6.77 (m, 1H), 4.65–4.55 (m, 1H), 3.10–3.09 (m, 4H), 2.89–2.73 (m, 2H), 2.15–2.01 (m, 5H), 1.90–1.66 (m, 8H). ^13^C NMR (100 MHz, DMSO-*d*_6_): 164.31, 140.65, 137.93, 137.88, 130.65, 130.46, 129.68, 129.64, 129.49, 128.54, 128.42, 125.68, 61.65, 61.46, 57.09, 53.91, 53.57, 50.94, 50.50, 31.59, 30.96, 30.78, 29.08, 27.74, 27.30, 27.26, 27.23, 27.01, 21.71. HRMS *m*/*z*: calcd for C_22_H_28_N_2_OS [M + H]^+^: 369.2001; found, 369.2011. The purity of the compound was checked by HPLC (*R*_t_ = 2.823 min) and was found to be 98.78% pure.

#### *N*-(1-(Cyclopentylmethyl)­azepan-4-yl)-*N*-phenylthiophene-3-carboxamide Hydrochloride (**42**)

The title compound was prepared following the general procedure as a white solid in 25% yield. ^1^H NMR (400 MHz, DMSO-*d*_6_): 9.72 (d, 1H, *J* = 39.28 Hz), 7.48–7.43 (m, 3H), 7.38–7.36 (m, 1H), 7.32–7.29 (m, 3H), 6.87–6.85 (m, 1H), 4.73–4.66 (m, 1H), 3.61–3.50 (m, 1H), 3.38–3.25 (m, 1H), 3.16–3.10 (m, 3H), 2.27–2.23 (m, 2H), 2.18–2.15 (m, 2H), 1.94–1.87 (m, 4H), 1.72–1.58 (m, 5H), 1.31–1.28 (m, 2H). ^13^C NMR (100 MHz, DMSO-*d*_6_): 164.38, 140.83, 140.62, 137.87, 137.84, 130.63, 130.49, 129.68, 129.66, 129.54, 129.49, 128.57, 128.41, 125.68, 61.69, 61.52, 57.49, 57.23, 54.22, 53.90, 51.32, 50.73, 35.27, 31.79, 31.26, 31.18, 31.01, 28.88, 27.29, 25.07, 25.02. HRMS *m*/*z*: calcd for C_23_H_30_N_2_OS [M + H]^+^: 383.2157; found, 383.2157. Mp 163.4–164.9 °C. The purity of the compound was checked by HPLC (*R*_t_ = 2.913 min) and was found to be 100.00% pure.

#### *N*-(1-(Cyclohexylmethyl)­azepan-4-yl)-*N*-phenylthiophene-3-carboxamide Hydro Chloride (**43**)

The title compound was prepared following the general procedure as a pale-yellow oil in 26% yield. ^1^H NMR (400 MHz, DMSO-*d*_6_): 9.42 (d, 1H, *J* = 47.59 Hz), 7.37–7.36 (m, 3H), 7.30–7.28 (m, 1H), 7.23–7.21 (m, 3H), 6.78–6.76­(m, 1H), 4.63–4.61 (m, 1H), 3.20–3.15 (m, 2H), 2.91–2.88 (m, 3H), 2.11–2.03 (m, 3H), 1.85–1.60 (m, 10H), 1.24–1.13 (m, 3H), 0.97–0.91 (m, 2H).^13^C NMR (100 MHz, DMSO-*d*_6_): 164.32, 140.68, 137.87, 130.65, 130.54, 129.68, 129.51, 128.56, 128.43, 125.68, 62.68, 62.48, 57.24, 54.37, 54.22, 51.55, 51.02, 33.00, 31.82, 31.32, 30.78, 30.72, 30.63, 28.81, 27.18, 25.93, 25.41. HRMS *m*/*z*: calcd for C_24_H_32_N_2_OS [M + H]^+^: 397.2314; found, 397.2320. The purity of the compound was checked by HPLC (*R*_t_ = 3.000 min) and was found to be 100.00% pure.

#### *N*-(1-Benzylazepan-4-yl)-*N*-phenylthiophene-3-carboxamide Hydrochloride (**44**)

The title compound was prepared following the general procedure as a white solid in 18% yield. ^1^H NMR (400 MHz, DMSO-*d*_6_): 10.55 (s, 1H), 7.61–7.59 (m, 2H), 7.46–7.44 (m, 3H), 7.39–7.33 (m, 3H), 7.29–7.27 (m, 1H), 7.23–7.19 (m, 3H), 6.77–6.76 (m, 1H), 4.67–4.54 (m, 1H), 4.30–4.29 (m, 2H), 3.29–3.25 (m, 2H), 3.13–3.11 (m, 2H), 2.18–2.06 (m, 3H), 1.87–1.80 (m, 2H), 1.71–1.69 (m, 1H). ^13^C NMR (100 MHz, DMSO-*d*_6_): 164.28, 140.65, 137.86, 131.74, 131.70, 130.85, 130.80, 130.64, 130.41, 129.88, 129.70, 129.64, 129.50, 129.25, 128.54, 128.48, 128.42, 125.67, 59.75, 57.17, 54.00, 53.01, 51.07, 50.11, 31.37, 31.07, 28.86, 27.60, 21.55, 19.44. HRMS *m*/*z*: calcd for C_24_H_26_N_2_OS [M + H]^+^: 391.1844; found, 391.1829. Mp 232.9–233.5 °C. The purity of the compound was checked by HPLC (*R*_t_ = 2.842 min) and was found to be 99.79% pure.

#### *N*-(1-Phenethylazepan-4-yl)-*N*-phenylthiophene-3-carboxamide Hydrochloride (**45**)

The title compound was prepared following the general procedure as a white solid in 39% yield. ^1^H NMR (400 MHz, DMSO-*d*_6_): 10.16 (d, 1H, *J* = 46.90 Hz), 7.39–7.22 (m, 12H), 6.79–6.78 (m, 1H), 4.68–4.65 (m, 1H), 3.55–3.42 (m, 2H), 3.28–3.23 (m, 3H), 3.02–2.98 (m, 3H), 2.35–2.21 (m, 1H), 2.11–1.89 (m, 2H), 1.88–1.69 (m, 3H). ^13^C NMR (100 MHz, DMSO-*d*_6_): 164.32, 141.06, 140.55, 137.90, 137.54, 130.71, 130.45, 129.68, 129.63, 129.47, 129.22, 129.09, 128.55, 128.44, 127.25, 125.66, 57.69, 57.60, 57.39, 57.10, 53.89, 53.41, 50.87, 50.48, 31.66, 30.97, 30.19, 30.10, 29.31, 28.07, 21.92, 20.02. HRMS *m*/*z*: calcd for C_25_H_28_N_2_OS [M + H]^+^: 405.2001; found, 405.1983. Mp 208.7–209.4 °C. The purity of the compound was checked by HPLC (*R*_t_ = 2.923 min) and was found to be 100.00% pure.

#### *N*-(1-Allylazepan-4-yl)-*N*-phenylfuran-2-carboxamide Hydrogen Chloride (**46**)

The title compound was prepared following the general procedure as a yellowish solid in 29% yield. ^1^H NMR (400 MHz, DMSO-*d*_6_) δ 10.20 (d, *J* = 82 Hz, 1H), 7.63 (d, *J* = 2.1 Hz, 1H), 7.48 (p, *J* = 4.0, 3.5 Hz, 3H), 7.32 (dt, *J* = 5.5, 3.4 Hz, 2H), 6.30 (dd, *J* = 3.3, 1.8 Hz, 1H), 6.01–5.59 (m, *J* = 15.0, 5.1 Hz, 1H), 5.52–5.44 (m, 3H), 4.73–4.55 (m, 1H), 3.69 (q, *J* = 6.4 Hz, 2H), 3.23–3.13 (m, 2H), 3.30 (dd, *J* = 14.1, 7.2 Hz, 1H), 2.06–1.78 (m, 7H), 1.73–1.67 (m, 1H). ^13^C NMR (100 MHz, DMSO-*d*_6_) δ158.2, 147.5, 145.3, 140.5, 130.8, 129.8, 129.1, 128.5, 125.3, 115.9, 111.6, 59.3, 58.2, 56.9, 51.5, 32.0, 30.7, 21.4, 20.8. HRMS *m*/*z*: calcd for C_21_H_26_N_2_O_2_ [M + H]^+^: 339.2073; found, 339.2078. Mp 197.6–198.4 °C. The purity of the compound was checked by HPLC (*R*_t_ = 2.653 min) and was found to be 98.5% pure.

#### *N*-(1-(Cyclopropylmethyl)­azepan-4-yl)-*N*-phenylfuran-2-carboxamide Hydrogen Chloride (**47**)

The title compound was prepared following the general procedure as a yellowish solid in 37% yield. ^1^H NMR (400 MHz, DMSO-*d*_6_) δ 10.41 (d, *J* = 50.0 Hz, 1H), 7.60 (d, *J* = 1.6 Hz, 1H), 7.45–7.43 (m, 3H), 7.30–7.27 (m, 2H), 6.30–6.29 (m, 1H), 5.48 (dd, *J* = 13.0, 3.7 Hz, 1H), 4.62 (dt, *J* = 42.5, 10.0 Hz, 1H), 3.48–3.40 (m, 2H), 3.35–3.23 (m, 1H), 3.08–3.03 (m, 1H), 2.96–2.91 (m, 2H), 2.06–1.87 (m, 6H), 1.76–1.63 (m, 2H), 1.11–1.06 (m, 1H), 0.61–0.57 (m, 2H), 0.38–0.37 (m, 2H). ^13^C NMR (100 MHz, DMSO-*d*_6_) δ 158.26, 147.51, 145.38, 140,30, 130.70, 129.81, 129,01, 116.01, 111.64, 60.51, 59.31, 56.51, 54.70, 50.51, 32.32, 31.43, 21.01, 20.43, 6.26, 4.68. HRMS *m*/*z*: calcd for C_22_H_28_N_2_O_2_ [M + H]^+^: 353.2229; found, 353.2242. Mp 223.8–224.6 °C. The purity of the compound was checked by HPLC (*R*_t_ = 2.687 min) and was found to be 98.63% pure.

#### *N*-(1-(Cyclobutylmethyl)­azepan-4-yl)-*N*-phenylfuran-2-carboxamide Hydrogen Chloride (**48**)

The title compound was prepared following the general procedure as a yellowish solid in 41% yield. ^1^H NMR (400 MHz, DMSO-*d*_6_) δ 10.15 (d, *J* = 55.0 Hz, 1H), 7.60 (d, *J* = 1.8 Hz, 1H), 7.44–7.43 (m, 3H), 7.28 (dt, *J* = 5.7, 2.5 Hz, 2H), 6.29 (dd, *J* = 3.6, 1.8 Hz, 1H), 5.44 (dd, *J* = 12.5, 3.5 Hz, 1H), 4.58 (dt, *J* = 46.6, 10.5 Hz, 1H), 3.16–3.04 (m, 4H), 2.97–2.91 (m, 1H), 2.73–2.70 (m, 1H), 2.04–1.97 (m, 6H), 1.90–1.60 (m, 9H). ^13^C NMR (100 MHz, DMSO-*d*_6_) δ157.22, 146.50, 144.34, 139.41, 129.80, 129.65, 128.79, 128.08, 114.96, 110.60, 60.07, 58.93, 55.32, 53.83, 50.14, 49.60, 31.18, 30.23, 29.91, 26.33, 20.09, 19.44, 17.66, 17.60. HRMS *m*/*z*: calcd for C_23_H_30_N_2_O_2_ [M + H]^+^: 367.2386; found, 367.2396. Mp 236.1–237.3 °C. The purity of the compound was checked by HPLC (*R*_t_ = 2.755 min) and was found to be 99.77% pure.

#### *N*-(1-(Cyclopentylmethyl)­azepan-4-yl)-*N*-phenylfuran-2-carboxamide Hydrogen Chloride (**49**)

The title compound was prepared following the general procedure as a yellowish solid in 43% yield. ^1^H NMR (400 MHz, DMSO-*d*_6_) δ 10.10 (d, *J* = 40.0 Hz, 1H), 7.61 (d, *J* = 1.5 Hz, 1H), 7.46–7.43 (m, 3H), 7.30–7.27 (m, 2H), 6.31–6.29 (m, 1H), 5.47 (dd, *J* = 11.0, 3.5 Hz, 1H), 4.62 (dt, *J* = 41.0, 10.1 Hz, 1H), 3.44–3.37 (m, 1H), 3.22 (bs, 1H), 3.04–2.99 (m, 3H), 2.22–2.14 (m, 1H), 2.05–1.48 (m, 15H), 1.26–1.17 (m, 2H). ^13^C NMR (100 MHz, DMSO-*d*_6_) δ158.12, 147.38, 145.25, 140.27, 130.68, 129.68, 128.97, 115.86, 111.50, 60.56, 59.26, 51.00, 50.48, 35.24, 32.26, 31.58, 31.32, 24.97, 20.75, 20.00. HRMS *m*/*z*: calcd for C_24_H_32_N_2_O_2_ [M + H]^+^: 381.2542; found, 381.2532. Mp 226.3–227.4 °C. The purity of the compound was checked by HPLC (*R*_t_ = 2.825 min) and was found to be 100% pure.

#### *N*-(1-(Cyclohexylmethyl)­azepan-4-yl)-*N*-phenylfuran-2-carboxamide Hydrogen Chloride (**50**)

The title compound was prepared following the general procedure as a yellowish solid in 48% yield. ^1^H NMR (400 MHz, DMSO-*d*_6_) δ 9.88 (d, *J* = 40.5 Hz, 1H), 7.61–7.61 (m, 1H), 7.45–7.44 (m, 3H), 7.30–7.27 (m, 2H), 6.31–6.29 (m, 1H), 5.46 (dd, *J* = 10.0, 3.6 Hz, 1H), 4.63 (dt, *J* = 40.0, 10.1 Hz, 1H), 3.34–3.36 (m, 1H), 3.21 (bs, 1H), 3.02–2.98 (m, 1H), 2.89–2.84 (m, 2H), 2.04– 1.58 (m, 15H), 1.27–1.06 (m, 3H), 1.97–0.86 (m, 2H). ^13^C NMR (100 MHz, DMSO-*d*_6_) δ158.02, 147.28, 145.15, 140.15, 130.57, 129.58, 128.57, 115.77, 111.40, 61.46, 60.03, 50.95, 50.39, 32.86, 32.72, 32.21, 32.71, 30.78, 25.80, 25.28, 20.52, 19.74. HRMS *m*/*z*: calcd for C_25_H_35_N_2_O_2_ [M + H]^+^: 395.2699; found, 395.2712. Mp 134.2–135.4 °C. The purity of the compound was checked by HPLC (*R*_t_ = 2.915 min) and was found to be 99.37% pure.

#### *N*-(1-Benzylazepan-4-yl)-*N*-phenylfuran-2-carboxamide Hydrogen Chloride (**51**)

The title compound was prepared following the general procedure as a yellowish solid in 40% yield. ^1^H NMR (400 MHz, DMSO-*d*_6_) δ 10.59 (bs, 1H), 7.61–7.59 (m, 3H), 7.44–7.43 (m, 6H), 7.29–7.27 (m, 2H), 6.29–6.27 (m, 1H), 5.45 (dd, *J* = 14.1, 3.7 Hz, 1H), 4.63 (dt, *J* = 45.0, 10.5 Hz, 1H), 4.27 (t, *J* = 6.1 Hz, 2H), 3.39–3.31 (m, 1H), 3.22–3.15 (m, 2H), 2.98–2.95 (m, 1H), 2.07–1.66 (m, 8H). ^13^C NMR (100 MHz, DMSO-*d*_6_) δ157.96, 147.24, 145.08, 140.14, 131.53, 130.72, 130.53, 129.53, 129.03, 128.82, 115.71, 111.33, 58.93, 57.78, 50.29, 48.83, 32.07, 31.17, 20.61, 19.99. HRMS *m*/*z*: calcd for C_25_H_29_N_2_O_2_ [M + H]^+^: 389.2229; found, 389.2217. Mp 100.1–101.0 °C. The purity of the compound was checked by HPLC (*R*_t_ = 2.772 min) and was found to be 99.74% pure.

#### *N*-(1-Phenethylazepan-4-yl)-*N*-phenylfuran-2-carboxamide Hydrogen Chloride (**52**)

The title compound was prepared following the general procedure as a yellowish solid in 52% yield. ^1^H NMR (400 MHz, DMSO-*d*_6_) δ 10.59 (d, *J* = 95 Hz, 1H), 7.61–7.61 (m, 1H), 7.46–7.43 (m, 3H), 7.34–7.24 (m, 7H), 6.31–6.29 (m, 1H), 5.47 (dd, *J* = 15.6, 3.5 Hz, 1H), 4.62 (t, *J* = 9.5 Hz, 1H), 3.48 (t, 1H), 3.32–3.2 (m, 4H), 3.14–3.00 (m, 3H), 2.08–1.66 (m, 8H). ^13^C NMR (100 MHz, DMSO-*d*_6_) δ 158.06, 147.32, 145.16, 140.30, 137.46, 130.65, 130.44, 129.62, 129.10, 128.91, 127.08, 57.39, 56.23, 51.59, 51.15, 31.86, 30.82, 29.98, 21.18, 20.61. HRMS *m*/*z*: calcd for C_26_H_31_N_2_O_2_ [M + H]^+^: 403.2386; found, 403.2387. Mp 103.3–104.1 °C. The purity of the compound was checked by HPLC (*R*_t_ = 2.853 min) and was found to be 98.27% pure.

#### *N*-(1-Allylazocan-5-yl)-*N*-phenylfuran-3-carboxamide Hydrogen Chloride (**53**)

The title compound was prepared following the general procedure as a yellowish solid in 27% yield. ^1^H NMR (400 MHz, DMSO-*d*_6_) δ 10.27 (d, *J* = 86.2 Hz, 1H), 7.61 (d, *J* = 2.0 Hz, 1H), 7.45 (p, *J* = 4.1, 3.7 Hz, 3H), 7.30 (dt, *J* = 5.4, 3.5 Hz, 2H), 6.31 (dd, *J* = 3.6, 1.7 Hz, 1H), 5.97 (m, 1H), 5.63–5.34 (m, 3H), 4.78–4.47 (m, 1H), 3.70 (q, *J* = 6.4 Hz, 2H), 3.27–3.09 (m, 2H), 3.00 (dd, *J* = 14.1, 7.2 Hz, 1H), 2.14–1.96 (m, 3H), 1.87 (tt, *J* = 14.3, 7.4 Hz, 3H), 1.71 (t, *J* = 11.4 Hz, 1H). ^13^C NMR (100 MHz, DMSO-*d*_6_) δ 158.20, 158.12, 147.49, 147.40, 145.30, 140.45, 140.08, 130.81, 130.58, 129.77, 129.07, 128.97, 128.46, 128.43, 125.31, 124.99, 115.93, 111.56, 59.24, 58.19, 56.89, 51.47, 31.97, 30.70, 21.43, 20.77. HRMS *m*/*z*: calcd for C_21_H_27_N_2_O_2_ [M + H]^+^: 339.2073; found, 339.2078. Mp 186.5–187.6 °C. The purity of the compound was checked by HPLC (*R*_t_ = 2.665 min) and was found to be 97.77% pure. HPLC data: purity 97.77%, retention time 2.665 min.

#### *N*-(1-(Cyclopropylmethyl)­azocan-5-yl)-*N*-phenylfuran-3-carboxamide Hydrogen Chloride (**54**)

The title compound was prepared following the general procedure as a yellowish solid in 33% yield. ^1^H NMR (400 MHz, DMSO-*d*_6_) δ 10.38 (d, *J* = 50.4 Hz, 1H), 7.62 (d, *J* = 1.8 Hz, 1H), 7.45 (m, 3H), 7.30 (m, 2H), 6.31 (dd, *J* = 3.6, 1.7 Hz, 1H), 5.47 (dd, *J* = 13.2, 3.5 Hz, 1H), 4.65 (dt, *J* = 42.0, 10.2 Hz, 1H), 3.57–3.38 (m, 2H), 3.27 (m, 1H), 3.12–3.01 (m, 1H), 2.95 (td, *J* = 7.6, 5.1 Hz, 2H), 2.19–1.83 (m, 6H), 1.82–1.60 (m, 2H), 1.10 (m, 1H), 0.60 (m, 2H), 0.49–0.30 (m, 2H). ^13^C NMR (100 MHz, DMSO-*d*_6_) δ 158.19, 158.12, 147.47, 147.41, 145.31, 140.37, 140.10, 130.77, 130.63, 129.76, 129.05, 128.98, 115.93, 111.57, 60.42, 59.24, 56.51, 54.71, 50.47, 32.22, 31.36, 21.00, 20.41, 6.21, 6.15, 4.61. HRMS *m*/*z*: calcd for C_22_H_29_N_2_O_2_ [M + H]^+^: 353.2229; found, 353.2235. Mp 194.7–195.8 °C. The purity of the compound was checked by HPLC (*R*_t_ = 2.697 min) and was found to be 97.85% pure. HPLC data: purity 97.85%, retention time 2.697 min.

#### *N*-(1-(Cyclobutylmethyl)­azocan-5-yl)-*N*-phenylfuran-3-carboxamide Hydrogen Chloride (**55**)

The title compound was prepared following the general procedure as a yellowish solid in 42% yield. ^1^H NMR (400 MHz, DMSO-*d*_6_) δ 10.19 (d, *J* = 60.0 Hz, 1H), 7.61 (d, *J* = 1.9 Hz, 1H), 7.52–7.37 (m, 3H), 7.29 (dt, *J* = 5.8, 2.4 Hz, 2H), 6.31 (dd, *J* = 3.6, 1.7 Hz, 1H), 5.46 (dd, *J* = 12.4, 3.6 Hz, 1H), 4.63 (dt, *J* = 46.5, 10.4 Hz, 1H), 3.32–3.25 (m, 1H), 3.22–3.00 (m, 4H), 2.96 (dd, *J* = 14.0, 7.1 Hz, 1H), 2.80–2.65 (m, 1H), 2.11–1.96 (m, 5H), 1.96–1.70 (m, 8H), 1.70–1.57 (m, 1H). ^13^C NMR (100 MHz, DMSO-*d*_6_) δ 158.19, 158.12, 147.46, 147.41, 145.31, 140.38, 140.10, 130.77, 130.61, 129.76, 129.05, 128.98, 115.92, 111.56, 61.04, 59.90, 56.49, 54.79, 51.11, 50.92, 32.15, 31.20, 30.88, 30.81, 27.30, 27.24, 21.06, 20.40, 18.62, 18.56. HRMS *m*/*z*: calcd for C_23_H_31_N_2_O_2_ [M + H]^+^: 367.2386; found, 367.2398. Mp 224.2–225.6 °C. The purity of the compound was checked by HPLC (*R*_t_ = 2.770 min) and was found to be 100% pure.

#### *N*-(1-(Cyclopentylmethyl)­azocan-5-yl)-*N*-phenylfuran-3-carboxamide Hydrogen Chloride (**56**)

The title compound was prepared following the general procedure as a yellowish solid in 45% yield. ^1^H NMR (400 MHz, DMSO-*d*_6_) δ 10.05 (d, *J* = 40.8 Hz, 1H), 7.62 (d, *J* = 1.6 Hz, 1H), 7.45 (dd, *J* = 4.6, 2.0 Hz, 3H), 7.36–7.22 (m, 2H), 6.31 (dd, *J* = 3.6, 1.7 Hz, 1H), 5.47 (dd, *J* = 11.1, 3.6 Hz, 1H), 4.63 (dt, *J* = 41.3, 10.2 Hz, 1H), 3.42 (t, *J* = 12.2 Hz, 2H), 3.28–3.17 (m, 1H), 3.11–2.94 (m, 3H), 2.19 (p, *J* = 7.7 Hz, 1H), 2.11–1.87 (m, 6H), 1.81 (m, 2H), 1.67–1.54 (m, 3H), 1.54–1.42 (m, 2H), 1.32–1.16 (m, 2H). ^13^C NMR (100 MHz, DMSO-*d*_6_) δ 158.19, 158.11, 147.45, 147.41, 145.32, 140.35, 140.13, 130.75, 130.63, 129.75, 129.04, 128.99, 115.93, 111.57, 60.63, 59.34, 56.22, 54.92, 51.07, 50.55, 35.32, 35.29, 32.33, 31.65, 31.39, 31.30, 25.04, 20.81, 20.06. HRMS *m*/*z*: calcd for C_24_H_33_N_2_O_2_ [M + H]^+^: 381.2542; found, 381.2532. Mp 217.3–218.7 °C. The purity of the compound was checked by HPLC (*R*_t_ = 2.838 min) and was found to be 100% pure.

#### *N*-(1-(Cyclohexylmethyl)­azocan-5-yl)-*N*-phenylfuran-3-carboxamide Hydrogen Chloride (**57**)

The title compound was prepared following the general procedure as a yellowish solid in 38% yield. ^1^H NMR (400 MHz, DMSO-*d*_6_) δ 9.84 (d, *J* = 40.0 Hz, 1H), 7.62 (d, *J* = 1.7 Hz, 1H), 7.50–7.43 (m, 3H), 7.30 (tt, *J* = 4.4, 2.7 Hz, 2H), 6.31 (dd, *J* = 3.5, 1.8 Hz, 1H), 5.47 (dd, *J* = 10.5, 3.5 Hz, 1H), 4.63 (dt, *J* = 40.1, 10.2 Hz, 1H), 3.47–3.11 (m, 3H), 3.00 (dd, *J* = 14.1, 6.8 Hz, 1H), 2.88 (q, *J* = 6.8 Hz, 2H), 2.16–1.84 (m, 6H), 1.86–1.75 (m, 2H), 1.75–1.50 (m, 6H), 1.34–1.05 (m, 3H), 0.93 (pd, *J* = 12.0, 11.5, 5.2 Hz, 2H). ^13^C NMR (100 MHz, DMSO-*d*_6_) δ 158.19, 158.12, 147.45, 147.42, 145.32, 140.32, 140.15, 130.74, 130.64, 129.75, 129.04, 128.99, 115.94, 111.57, 65.38, 61.63, 60.20, 56.09, 51.12, 50.55, 33.03, 32.89, 32.38, 31.76, 31.08, 30.96, 25.97, 25.47, 25.45, 20.69, 19.91. HRMS *m*/*z*: calcd for C_25_H_35_N_2_O_2_ [M + H]^+^: 395.2699; found, 395.2694. Mp 125.5–126.7 °C. The purity of the compound was checked by HPLC (*R*_t_ = 2.928 min) and was found to be 99.27% pure. HPLC data: purity 99.27%, retention time 2.928 min.

#### *N*-(1-Benzylazocan-5-yl)-*N*-phenylfuran-3-carboxamide Hydrogen Chloride (**58**)

The title compound was prepared following the general procedure as a yellowish solid in 44% yield. ^1^H NMR (400 MHz, DMSO-*d*_6_) δ 10.64 (d, *J* = 41.9 Hz, 1H), 7.63 (dd, *J* = 10.5, 5.7 Hz, 3H), 7.55–7.34 (m, *J* = 3.2, 2.7 Hz, 6H), 7.29 (dd, *J* = 6.5, 3.0 Hz, 2H), 6.38–6.10 (m, 1H), 5.48 (dd, *J* = 14.0, 3.6 Hz, 1H), 4.65 (dt, *J* = 46.3, 10.3 Hz, 1H), 4.28 (t, *J* = 6.0 Hz, 2H), 3.34 (d, *J* = 11.1 Hz, 1H), 3.25–3.15 (m, 2H), 2.98 (dt, *J* = 13.5, 6.6 Hz, 1H), 2.15–2.00 (m, 3H), 1.94 (h, *J* = 6.0, 4.9 Hz, 3H), 1.72 (m, *J* = 28.5, 11.1, 10.3 Hz, 2H). ^13^C NMR (100 MHz, DMSO-*d*_6_) δ 158.19, 158.13, 147.47, 147.41, 145.31, 140.37, 140.11, 131.75, 131.58, 130.95, 130.77, 130.63, 129.76, 129.26, 129.20, 129.05, 128.99, 115.94, 111.57, 59.16, 58.01, 56.45, 54.54, 50.52, 50.29, 49.06, 32.30, 31.40, 20.85, 20.22. HRMS *m*/*z*: calcd for C_25_H_29_N_2_O_2_ [M + H]^+^: 389.2229; found, 389.2217. Mp 98.5–99.7 °C. The purity of the compound was checked by HPLC (*R*_t_ = 2.780 min) and was found to be 99.46% pure.

#### *N*-(1-Phenethylazocan-5-yl)-*N*-phenylfuran-3-carboxamide Hydrogen Chloride (**59**)

The title compound was prepared following the general procedure as a yellowish solid in 54% yield. ^1^H NMR (400 MHz, DMSO-*d*_6_) δ 10.48 (d, *J* = 96.7 Hz, 1H), 7.62 (d, *J* = 1.9 Hz, 1H), 7.45 (m, *J* = 6.9, 3.2 Hz, 3H), 7.29 (m, *J* = 15.8, 8.7, 7.1, 2.2 Hz, 7H), 6.31 (dd, *J* = 3.6, 1.7 Hz, 1H), 5.48 (dd, *J* = 15.4, 3.6 Hz, 1H), 4.73 (t, *J* = 9.4 Hz, 1H), 3.56–3.44 (m, 1H), 3.26 (m, *J* = 21.8, 12.8, 4.5 Hz, 4H), 3.18–2.97 (m, 3H), 2.15–1.77 (m, 6H), 1.69 (m, *J* = 14.7, 14.0, 4.7 Hz, 2H). ^13^C NMR (100 MHz, DMSO-*d*_6_) δ 158.21, 158.11, 147.48, 147.40, 145.31, 140.45, 140.09, 137.61, 137.58, 130.80, 130.59, 129.78, 129.25, 129.23, 129.07, 128.99, 127.23, 115.94, 111.58, 57.54, 56.76, 56.38, 54.84, 51.74, 51.31, 32.02, 30.97, 30.13, 30.05, 21.33, 20.76. HRMS *m*/*z*: calcd for C_26_H_31_N_2_O_2_ [M + H]^+^: 403.2386; found, 403.2387. Mp 101.1–102.5 °C. The purity of the compound was checked by HPLC (*R*_t_ = 2.852 min) and was found to be 98.34% pure.

#### *N*-(1-Allylazepan-4-yl)-*N*-phenylthiophene-2-carboxamide Hydrogen Chloride (**60**)

The title compound was prepared following the general procedure as a yellowish solid in 49% yield. ^1^H NMR (400 MHz, DMSO-*d*_6_) δ 10.04 (d, *J* = 85.3 Hz, 1H), 7.58 (dd, *J* = 5.0, 1.2 Hz, 1H), 7.53–7.41 (m, 3H), 7.34 (m, *J* = 7.0, 5.1, 3.0 Hz, 2H), 6.82 (dd, *J* = 5.0, 3.8 Hz, 1H), 6.38 (td, *J* = 3.7, 1.2 Hz, 1H), 6.08–5.87 (m, 1H), 5.59–5.38 (m, 2H), 4.76–4.55 (m, 1H), 3.71 (q, *J* = 6.2 Hz, 2H), 3.51 (s, 2H), 3.37 (td, *J* = 11.6, 9.9, 4.8 Hz, 2H), 3.31–3.13 (m, 2H), 3.13–2.92 (m, 2H), 2.18–1.97 (m, 3H), 1.97–1.80 (m, 3H), 1.69 (d, *J* = 11.3 Hz, 2H). ^13^C NMR (100 MHz, DMSO-*d*_6_) δ 161.29, 131.67, 131.31, 131.10, 129.97, 129.44, 129.35, 128.42, 128.38, 127.36, 125.41, 125.07, 58.22, 51.58, 32.01, 30.63, 21.48, 20.84. HRMS *m*/*z*: calcd for C_21_H_26_N_2_OS [M + H]^+^: 355.1844; found, 355.1847. Mp 198.9–199.8. The purity of the compound was checked by HPLC (*R*_t_ = 2.878 min) and was found to be 99.90% pure.

#### *N*-(1-(Cyclopropylmethyl)­azepan-4-yl)-*N*-phenylthiophene-2-carboxamide Hydrogen Chloride (**61**)

The title compound was prepared following the general procedure as a pale solid in 35.4% yield. ^1^H NMR (400 MHz, DMSO-*d*_6_) δ 9.74 (d, *J* = 58.9 Hz, 1H), 7.59 (dd, *J* = 5.0, 1.3 Hz, 1H), 7.48 (dd, *J* = 4.6, 2.3 Hz, 3H), 7.34 (dt, *J* = 7.4, 2.7 Hz, 2H), 6.82 (dd, *J* = 5.0, 3.7 Hz, 1H), 6.38 (td, *J* = 3.8, 1.2 Hz, 1H), 4.76–4.55 (m, 1H), 3.46 (s, 1H), 3.41–3.31 (m, 2H), 3.16–3.01 (m, 1H), 2.96 (t, *J* = 6.7 Hz, 2H), 2.06 (t, *J* = 14.9 Hz, 3H), 1.92 (s, 3H), 1.85–1.57 (m, 2H), 1.16–0.97 (m, 1H), 0.61 (m, *J* = 8.5, 3.2 Hz, 2H), 0.38 (d, *J* = 5.0 Hz, 2H). ^13^C NMR (100 MHz, DMSO-*d*_6_) δ 161.27, 161.20, 140.35, 140.11, 139.46, 139.42, 131.84, 131.66, 131.27, 131.14, 129.95, 129.41, 129.35, 127.36, 59.29, 56.91, 50.58, 32.27, 31.32, 21.04, 20.48, 6.23, 6.15, 4.60. HRMS *m*/*z*: calcd for C_22_H_28_N_2_OS [M + H]^+^: 369.2001; found, 369.1977. 208.4–209.7 °C. The purity of the compound was checked by HPLC (*R*_t_ = 2.803 min) and was found to be 99.53% pure.

#### *N*-(1-(Cyclobutylmethyl)­azepan-4-yl)-*N*-phenylthiophene-2-carboxamide Hydrogen Chloride (**62**)

The title compound was prepared following the general procedure as a pale solid in 38% yield. ^1^H NMR (400 MHz, DMSO-*d*_6_) δ 9.73 (d, *J* = 63.5 Hz, 1H), 7.58 (dd, *J* = 5.0, 1.2 Hz, 1H), 7.47 (dt, *J* = 5.3, 2.0 Hz, 3H), 7.41–7.28 (m, 2H), 6.81 (dd, *J* = 5.1, 3.8 Hz, 1H), 6.38 (td, *J* = 3.7, 1.2 Hz, 1H), 4.66 (dt, *J* = 44.9, 10.5 Hz, 1H), 3.38–3.29 (m, 1H), 3.16 (d, *J* = 6.3 Hz, 2H), 3.10 (td, *J* = 7.4, 5.3 Hz, 2H), 2.97 (dd, *J* = 13.7, 6.9 Hz, 1H), 2.76–2.64 (m, 1H), 2.12–1.98 (m, 5H), 1.93–1.73 (m, 7H), 1.66 (t, *J* = 11.7 Hz, 2H). ^13^C NMR (100 MHz, DMSO-*d*_6_) δ 161.27, 161.20, 139.46, 139.41, 131.83, 131.66, 131.26, 131.13, 130.16, 129.95, 129.41, 129.35, 127.36, 61.11, 60.63, 59.91, 51.19, 51.04, 32.20, 31.17, 30.94, 30.87, 30.80, 27.42, 27.36, 27.30, 27.23, 21.08, 20.63, 20.47, 18.66, 18.63, 18.56. HRMS *m*/*z*: calcd for C_23_H_30_N_2_OS [M + H]^+^: 383.2157; found, 383.2164. Mp 235.8–237.1 °C. The purity of the compound was checked by HPLC (*R*_t_ = 2.908 min) and was found to be 98.96% pure.

#### *N*-(1-(Cyclopentylmethyl)­azepan-4-yl)-*N*-phenylthiophene-2-carboxamide Hydrogen Chloride (**63**)

The title compound was prepared following the general procedure as a pale solid 36% yield. ^1^H NMR (400 MHz, DMSO-*d*_6_) δ 9.85 (s, 1H), 7.59 (dd, *J* = 5.1, 1.2 Hz, 1H), 7.47 (m, *J* = 5.9, 1.9 Hz, 3H), 7.34 (m, *J* = 4.2, 3.6, 2.2 Hz, 3H), 6.82 (dd, *J* = 5.1, 3.8 Hz, 1H), 6.38 (td, *J* = 3.6, 1.2 Hz, 1H), 4.76–4.55 (m, 1H), 3.41 (d, *J* = 11.1 Hz, 2H), 3.28–3.10 (m, 2H), 3.06 (m, *J* = 13.6, 6.9, 6.1 Hz, 4H), 2.28–2.16 (m, 2H), 2.03 (d, *J* = 8.0 Hz, 2H), 1.93 (s, 3H), 1.81 (s, 4H), 1.69–1.60 (m, 3H), 1.23 (s, 3H). ^13^C NMR (100 MHz, DMSO-*d*_6_) δ 161.28, 161.21, 140.33, 139.45, 131.83, 131.66, 131.24, 131.14, 130.15, 129.95, 129.35, 127.36, 59.84, 59.38, 51.24, 50.63, 35.35, 35.31, 35.29, 32.54, 32.37, 31.40, 31.24, 25.08, 25.05, 24.91, 23.87, 20.86, 20.35, 20.17. HRMS *m*/*z*: calcd for C_24_H_32_N_2_OS [M + H]^+^: 397.2314; found, 397.2296. Mp 209.5–211.3 °C. The purity of the compound was checked by HPLC (*R*_t_ = 3.067 min) and was found to be 99.56% pure.

#### *N*-(1-(Cyclohexylmethyl)­azepan-4-yl)-*N*-phenylthiophene-2-carboxamide Hydrogen Chloride (**64**)

The title compound was prepared following the general procedure as a pale solid in 53.7% yield. ^1^H NMR (400 MHz, DMSO-*d*_6_) δ 9.36 (d, *J* = 39.6 Hz, 1H), 7.59 (dd, *J* = 5.0, 1.2 Hz, 1H), 7.53–7.42 (m, 3H), 7.40–7.31 (m, 2H), 6.82 (dd, *J* = 5.1, 3.8 Hz, 1H), 6.42–6.34 (m, 1H), 4.79–4.57 (m, 1H), 3.47 (s, 3H), 3.28–3.18 (m, 1H), 3.05–2.98 (m, 1H), 2.90 (q, *J* = 6.6 Hz, 2H), 2.04 (dd, *J* = 14.9, 8.7 Hz, 3H), 1.96–1.87 (m, 2H), 1.79–1.55 (m, 8H), 1.30–1.09 (m, 3H), 1.01–0.86 (m, 2H). ^13^C NMR (100 MHz, DMSO-*d*_6_) δ 161.29, 139.43, 131.85, 131.67, 131.25, 131.16, 129.96, 129.39, 127.37, 60.21, 51.38, 50.62, 33.02, 32.87, 32.41, 31.58, 30.95, 30.78, 25.96, 25.43, 25.40, 20.76, 20.06. HRMS *m*/*z*: calcd for C_25_H_34_N_2_OS [M + H]^+^: 411.2470; found, 411.2479. Mp 217.1–218.9 °C. The purity of the compound was checked by HPLC (*R*_t_ = 3.118 min) and was found to be 97.30% pure.

#### *N*-(1-Benzylazepan-4-yl)-*N*-phenylthiophene-2-carboxamide Hydrogen Chloride (**65**)

The title compound was prepared following the general procedure as oily in 43% yield. ^1^H NMR (400 MHz, DMSO-*d*_6_) δ 10.76 (s, 1H), 7.65 (dd, *J* = 6.4, 4.1, 2.1 Hz, 2H), 7.58 (dd, *J* = 5.1, 1.2 Hz, 1H), 7.49–7.46 (m, 3H), 7.45–7.42 (m, 4H), 7.34 (dd, *J* = 7.1, 3.2, 1.3 Hz, 2H), 6.81 (dd, *J* = 5.0, 3.8 Hz, 1H), 6.38 (m, *J* = 4.9, 3.7, 1.2 Hz, 1H), 4.68 (dt, *J* = 41.8, 10.5 Hz, 1H), 4.34–4.26 (m, 2H), 3.46–3.28 (m, 2H), 3.17 (s, 3H), 2.99–2.91 (m, 1H), 2.19–2.09 (m, 1H), 1.93–1.65 (m, 5H). ^13^C NMR (100 MHz, DMSO-*d*_6_) δ 161.26, 161.21, 140.32, 140.11, 139.45, 139.40, 132.95, 131.83, 131.76, 131.74, 131.67, 131.58, 131.24, 131.15, 130.95, 130.14, 129.94, 129.86, 129.76, 129.48, 129.39, 129.35, 129.26, 129.20, 127.35, 59.22, 58.54, 58.01, 50.68, 50.53, 50.34, 49.05, 32.35, 31.39, 20.84, 20.37, 20.27, 15.64. HRMS *m*/*z*: calcd for C_25_H_28_N_2_OS [M + H]^+^: 405.2001; found, 405.1994. The purity of the compound was checked by HPLC (*R*_t_ = 2.875 min) and was found to be 99.88% pure.

#### *N*-(1-Phenethylazepan-4-yl)-*N*-phenylthiophene-2-carboxamide Hydrogen Chloride (**66**)

The title compound was prepared following the general procedure as a pale solid in 37.2% yield. ^1^H NMR (400 MHz, DMSO-*d*_6_) δ 9.54 (d, *J* = 92.7 Hz, 1H), 7.59 (dd, *J* = 5.0, 1.2 Hz, 1H), 7.48 (td, *J* = 4.8, 2.2 Hz, 3H), 7.42–7.31 (m, 4H), 7.30–7.20 (m, 3H), 6.82 (dd, *J* = 5.1, 3.8 Hz, 1H), 6.39 (q, *J* = 3.3, 2.2 Hz, 1H), 4.84–4.57 (m, 1H), 3.51 (t, *J* = 11.9 Hz, 1H), 3.33–3.22 (m, 5H), 3.21–3.10 (m, 1H), 2.99 (dt, *J* = 12.4, 4.4 Hz, 2H), 2.08 (dt, *J* = 11.0, 5.0 Hz, 3H), 1.87 (d, *J* = 24.8 Hz, 3H), 1.71 (d, *J* = 10.7 Hz, 1H). ^13^C NMR (100 MHz, DMSO-*d*_6_) δ 161.32, 139.47, 137.41, 137.38, 131.87, 131.69, 131.34, 131.11, 130.00, 129.46, 129.38, 129.26, 129.10, 127.38, 127.31, 65.38, 56.51, 52.21, 32.02, 30.69, 30.24, 30.10, 21.54, 20.95, 15.64. HRMS *m*/*z*: calcd for C_26_H_30_N_2_OS [M + H]^+^: 419.2157; found, 419.2159. Mp 195.7–197.2. The purity of the compound was checked by HPLC (*R*_t_ = 3.018 min) and was found to be 97.10% pure.

#### *N*-(1-Allylazepan-4-yl)-*N*-phenyl-1*H*-pyrrole-2-carboxamide Hydrogen Chloride (**67**)

The title compound was prepared following the general procedure as a pale solid in 32% yield. ^1^H NMR (400 MHz, DMSO-*d*_6_) δ 10.04 (d, *J* = 85.3 Hz, 1H), 7.58 (dd, *J* = 5.0, 1.2 Hz, 1H), 7.53–7.41 (m, 3H), 7.34 (m, *J* = 7.0, 5.1, 3.0 Hz, 2H), 6.82 (dd, *J* = 5.0, 3.8 Hz, 1H), 6.38 (td, *J* = 3.7, 1.2 Hz, 1H), 6.08–5.87 (m, 1H), 5.59–5.38 (m, 2H), 4.76–4.55 (m, 1H), 3.71 (q, *J* = 6.2 Hz, 2H), 3.51 (s, 2H), 3.37 (td, *J* = 11.6, 9.9, 4.8 Hz, 2H), 3.31–3.13 (m, 2H), 3.13–2.92 (m, 2H), 2.18–1.97 (m, 3H), 1.97–1.80 (m, 3H), 1.69 (d, *J* = 11.3 Hz, 2H).^13^C NMR (100 MHz, DMSO-*d*_6_): 164.5, 140.1, 137.9, 137.6, 130.8, 129.7, 129.6, 129.1, 129.1, 128.5, 128.4, 127.3, 125.8, 56.6, 49.9, 49.0, 47.3, 38.6, 36.2, 35.7, 32.3, 30.7, 29.9. HRMS *m*/*z*: calcd for C_21_H_27_N_3_O [M + H]^+^: 338.2232; found, 338.2240. Mp 229.8–231.7 °C. The purity of the compound was checked by HPLC (*R*_t_ = 2.562 min) and was found to be 99.55% pure.

#### *N*-(1-(Cyclopropylmethyl)­azepan-4-yl)-*N*-phenyl-1*H*-pyrrole-2-carboxamide Hydrogen Chloride (**68**)

The title compound was prepared following the general procedure as a pale solid in 35% yield. ^1^H NMR (400 MHz, DMSO-*d*_6_) δ 10.74 (d, *J* = 35.4 Hz, 1H), 9.92 (s, 1H), 7.53–7.25 (m, 4H), 7.25–7.02 (m, 3H), 6.78 (d, *J* = 5.0 Hz, 1H), 4.64 (dt, *J* = 38.8, 10.1 Hz, 1H), 3.44 (d, *J* = 12.3 Hz, 3H), 3.39–3.19 (m, 2H), 3.06 (dt, *J* = 13.9, 6.7 Hz, 1H), 2.08–1.86 (m, 5H), 1.92–1.57 (m, 3H), 1.26–1.06 (m, 1H), 0.60 (h, *J* = 5.2, 4.6 Hz, 2H), 0.41 (t, *J* = 4.9 Hz, 2H). ^13^C NMR (100 MHz, DMSO-*d*_6_): 164.5, 140.1, 137.9, 137.6, 130.8, 129.7, 129.6, 129.1, 129.1, 128.5, 128.4, 127.3, 125.8, 56.6, 49.9, 49.0, 47.3, 38.6, 36.2, 35.7, 32.3, 30.7, 29.9. HRMS *m*/*z*: calcd for C_22_H_29_N_3_O [M + H]^+^: 352.2389; found, 352.2377. Mp 189.7–190.5 °C. The purity of the compound was checked by HPLC (*R*_t_ = 2.860 min) and was found to be 98.90% pure.

#### *N*-(1-(Cyclobutylmethyl)­azepan-4-yl)-*N*-phenyl-1*H*-pyrrole-2-carboxamide Hydrogen Chloride (**69**)

The title compound was prepared following the general procedure as a pale solid in 30% yield. ^1^H NMR (400 MHz, DMSO-*d*_6_) δ 11.35 (s, 1H), 9.98 (d, *J* = 88.6 Hz, 1H), 7.58–7.39 (m, 3H), 7.29 (m, *J* = 3.4, 2.5, 1.4 Hz, 2H), 6.74 (td, *J* = 2.7, 1.4 Hz, 1H), 5.73 (dt, *J* = 3.8, 2.5 Hz, 1H), 4.87–4.57 (m, 1H), 4.48 (m, *J* = 4.0, 2.5, 1.4 Hz, 1H), 3.40–3.25 (m, 2H), 3.25–3.08 (m, 3H), 2.96 (dt, *J* = 13.6, 6.8 Hz, 1H), 2.75 (m, *J* = 23.3, 7.8 Hz, 1H), 2.16–1.95 (m, 6H), 1.95–1.78 (m, 6H), 1.61 (t, *J* = 11.1 Hz, 1H). ^13^C NMR (100 MHz, DMSO-*d*_6_) δ 161.26, 161.21, 140.32, 140.11, 139.45, 139.40, 132.95, 131.83, 131.76, 131.74, 131.67, 131.58, 131.24, 131.15, 130.95, 130.14, 129.94, 129.86, 129.76, 129.48, 129.39, 129.35, 129.26, 129.20, 127.35, 62.29, 59.22, 58.54, 58.01, 56.86, 50.68, 50.53, 50.34, 49.05, 32.35, 31.39, 30.90, 20.84, 20.37, 20.27, 19.56, 15.64. HRMS *m*/*z*: calcd for C_23_H_31_N_3_O [M + H]^+^: 366.2545; found, 366.2555. Mp 167.9–169.1 °C. The purity of the compound was checked by HPLC (*R*_t_ = 2.630 min) and was found to be 97.59% pure.

#### *N*-(1-(Cyclopentylmethyl)­azepan-4-yl)-*N*-phenyl-1*H*-pyrrole-2-carboxamide Hydrogen Chloride (**70**)

The title compound was prepared following the general procedure as a pale solid in 33% yield. ^1^H NMR (400 MHz, DMSO-*d*_6_) δ 11.44–11.26 (m, 1H), 10.01 (d, *J* = 57.0 Hz, 1H), 7.52–7.42 (m, 3H), 7.30 (m, *J* = 5.1, 3.0, 1.2 Hz, 2H), 5.73 (dt, *J* = 3.8, 2.5 Hz, 1H), 4.68 (q, *J* = 14.4, 10.3 Hz, 1H), 4.62–4.40 (m, 1H), 3.23 (d, *J* = 4.6 Hz, 1H), 3.10–2.97 (m, 3H), 2.28–2.11 (m, 2H), 2.11–1.86 (m, 7H), 1.86–1.76 (m, 3H), 1.67–1.42 (m, 6H), 1.32–1.15 (m, 2H). ^13^C NMR (100 MHz, DMSO-*d*_6_) δ 161.28, 161.21, 140.33, 139.45, 131.83, 131.66, 131.24, 131.14, 130.15, 129.95, 129.35, 127.36, 59.84, 59.38, 51.24, 50.63, 35.35, 35.31, 35.29, 32.54, 32.37, 31.40, 31.24, 25.08, 25.05, 24.91, 23.87, 20.86, 20.35, 20.17. HRMS *m*/*z*: calcd for C_24_H_33_N_3_O [M + H]^+^: 380.2702; found, 380.2680. Mp 187.8–189.4 °C. The purity of the compound was checked by HPLC (*R*_t_ = 2.888 min) and was found to be 100.00% pure.

#### *N*-(1-(Cyclohexylmethyl)­azepan-4-yl)-*N*-phenyl-1*H*-pyrrole-2-carboxamide Hydrogen Chloride (**71**)

The title compound was prepared following the general procedure as a pale-yellow oil in 41% yield. ^1^H NMR (400 MHz, DMSO-*d*_6_) δ 10.91 (s, 1H), 10.16 (d, *J* = 56.6 Hz, 1H), 7.55–7.32 (m, 3H), 7.23 (dt, *J* = 6.8, 1.8 Hz, 2H), 6.46 (q, *J* = 2.4 Hz, 1H), 6.15–5.88 (m, 1H), 5.68–5.45 (m, 1H), 4.86–4.51 (m, 1H), 3.34–3.24 (m, 2H), 3.14 (td, *J* = 10.1, 5.7 Hz, 2H), 3.07 (td, *J* = 6.6, 3.5 Hz, 2H), 2.95 (dt, *J* = 13.9, 6.7 Hz, 1H), 2.79–2.67 (m, 1H), 2.14–1.88 (m, 7H), 1.90–1.70 (m, 6H), 1.63 (dd, *J* = 24.0, 13.1 Hz, 2H). ^13^C NMR (100 MHz, DMSO-*d*_6_) δ 164.19, 164.13, 141.66, 131.31, 131.21, 129.55, 128.52, 128.47, 122.42, 119.53, 119.48, 117.68, 110.30, 110.27, 61.14, 59.87, 51.14, 50.97, 32.56, 31.58, 30.89, 30.82, 27.32, 27.24, 21.13, 20.57, 18.63, 18.56. HRMS *m*/*z*: calcd for C_25_H_35_N_3_O [M + H]^+^: 394.2858; found, 394.2869. The purity of the compound was checked by HPLC (*R*_t_ = 2.565 min) and was found to be 99.15% pure.

#### *N*-(1-Benzylazepan-4-yl)-*N*-phenyl-1*H*-pyrrole-2-carboxamide Hydrogen Chloride (**72**)

The title compound was prepared following the general procedure as a pale solid in 42.2% yield. ^1^H NMR (400 MHz, DMSO-*d*_6_) δ 12.26 (s, 1H), 10.49 (s, 1H), 7.62 (m, *J* = 6.5, 4.2, 2.2 Hz, 3H), 7.54–7.36 (m, 6H), 7.36–7.20 (m, 6H), 7.16 (s, 1H), 6.84–6.62 (m, 1H), 6.29 (dt, *J* = 3.3, 2.3 Hz, 1H), 5.96 (d, *J* = 17.3 Hz, 2H), 4.80–4.43 (m, 2H), 4.38–4.13 (m, 3H), 3.37–3.32 (m, 1H), 3.19 (d, *J* = 15.0 Hz, 2H), 3.03–2.86 (m, 2H), 2.06 (d, *J* = 8.9 Hz, 2H), 1.99–1.84 (m, 4H), 1.75 (s, 2H). ^13^C NMR (100 MHz, DMSO-*d*_6_) δ 161.28, 161.21, 140.33, 139.45, 131.83, 131.66, 131.24, 131.14, 130.15, 129.95, 129.35, 127.36, 59.84, 59.38, 51.24, 50.63, 35.35, 35.31, 35.29, 32.54, 32.37, 31.40, 31.24, 25.08, 25.05, 24.91, 23.87, 20.86, 20.35, 20.17. HRMS *m*/*z*: calcd for C_25_H_29_N_3_O [M + H]^+^: 388.2389; found, 388.2365. Mp 192.4–193.8 °C. The purity of the compound was checked by HPLC (*R*_t_ = 3.077 min) and was found to be 100% pure.

#### *N*-(1-Phenethylazocan-5-yl)-*N*-phenyl-1*H*-pyrrole-2-carboxamide Hydrogen Chloride (**73**)

The title compound was prepared following the general procedure as a pale solid in 35% yield. ^1^H NMR (400 MHz, DMSO-*d*_6_) δ 11.34 (s, 1H), 10.11 (d, *J* = 121.3 Hz, 1H), 7.49 (q, *J* = 3.1 Hz, 3H), 7.36–7.20 (m, 7H), 6.75 (td, *J* = 2.7, 1.4 Hz, 1H), 5.73 (dt, *J* = 3.8, 2.5 Hz, 1H), 4.85–4.63 (m, 1H), 4.56–4.40 (m, 1H), 3.50 (d, *J* = 21.8 Hz, 4H), 3.28–3.19 (m, 2H), 3.18–3.06 (m, 1H), 3.02 (dt, *J* = 13.8, 4.8 Hz, 2H), 2.05 (q, *J* = 10.7, 9.4 Hz, 3H), 1.92 (s, 3H), 1.67 (d, *J* = 10.6 Hz, 1H). ^13^C NMR (100 MHz, DMSO-*d*_6_) δ 160.55, 160.45, 137.54, 137.49, 131.37, 131.22, 129.78, 129.26, 129.24, 129.08, 129.04, 128.99, 127.26, 125.63, 125.55, 121.72, 113.11, 109.01, 57.71, 56.48, 52.09, 51.53, 32.26, 31.86, 31.01, 30.17, 30.06, 21.45, 20.95. HRMS *m*/*z*: calcd for C_26_H_31_N_3_O [M + H]^+^: 402.2545; found, 402.2531. Mp 198.5–200.1 °C. The purity of the compound was checked by HPLC (*R*_t_ = 2.863 min) and was found to be 99.30% pure.

#### *N*-(1-Allylazocan-5-yl)-*N*-phenylthiophene-3-carboxamide Hydrochloride (**74**)

The title compound was prepared following the general procedure as a pale-yellow oil in 17.7% yield. ^1^H NMR (400 MHz, DMSO-*d*_6_) δ 10.19 (d, *J* = 77.2 Hz, 1H), 7.43–7.12 (m, 7H), 6.77 (m, *J* = 5.0, 3.5, 1.3 Hz, 1H), 6.07–5.89 (m, 1H), 5.59–5.38 (m, 2H), 4.79–4.52 (m, 1H), 3.71 (q, *J* = 5.4 Hz, 3H), 3.37 (m, *J* = 14.3, 9.7, 4.9 Hz, 1H), 3.28–3.18 (m, 1H), 3.00 (dt, *J* = 14.6, 7.2 Hz, 1H), 2.13–1.97 (m, 3H), 1.95–1.79 (m, 4H), 1.76 (t, *J* = 11.1 Hz, 1H). ^13^C NMR (100 MHz, DMSO-*d*_6_) δ 130.71, 130.47, 129.53, 129.29, 128.29, 125.61, 125.36, 51.44, 32.16, 30.86, 21.53, 20.85. HRMS *m*/*z*: calcd for C_21_H_26_N_2_OS [M + H]^+^: 355.1844; found, 355.1855. The purity of the compound was checked by HPLC (*R*_t_ = 2.733 min) and was found to be 96.59% pure.

#### *N*-(1-(Cyclopropylmethyl)­azocan-5-yl)-*N*-phenylthiophene-3-carboxamide Hydrogen Chloride (**75**)

The title compound was prepared following the general procedure as a white solid in 37.7% yield. ^1^H NMR (400 MHz, DMSO-*d*_6_) δ 10.74 (d, *J* = 35.4 Hz, 1H), 7.42–7.11 (m, 7H), 6.78 (d, *J* = 5.0 Hz, 1H), 4.75–4.50 (m, 1H), 3.44 (d, *J* = 12.3 Hz, 3H), 3.36–3.19 (m, 2H), 3.06 (dt, *J* = 13.6, 6.8 Hz, 1H), 2.95 (q, *J* = 6.1 Hz, 2H), 2.07–1.61 (m, 7H), 1.19–1.03 (m, 1H), 0.60 (h, *J* = 5.3, 4.7 Hz, 2H), 0.41 (t, *J* = 4.9 Hz, 2H). ^13^C NMR (100 MHz, DMSO-*d*_6_) δ 164.16, 164.12, 141.22, 140.96, 138.18, 138.13, 130.61, 130.49, 129.50, 129.26, 129.20, 128.43, 128.32, 128.27, 125.60, 60.37, 59.17, 56.91, 55.42, 50.47, 50.28, 32.43, 31.66, 31.17, 21.05, 20.45, 6.21, 6.16, 4.65, 4.63. HRMS *m*/*z*: calcd for C_22_H_28_N_2_OS [M + H]^+^: 369.2001; found, 369.2000. Mp 173.3–173.9 °C. The purity of the compound was checked by HPLC (*R*_t_ = 2.775 min) and was found to be 98.51% pure.

#### *N*-(1-(Cyclobutylmethyl)­azocan-5-yl)-*N*-phenylthiophene-3-carboxamide Hydrogen Chloride (**76**)

The title compound was prepared following the general procedure as a white solid in 40.0% yield. ^1^H NMR (400 MHz, DMSO-*d*_6_) δ 10.37 (d, *J* = 49.8 Hz, 1H), 7.38–7.30 (m, 3H), 7.28 (td, *J* = 4.8, 2.3 Hz, 1H), 7.21 (dt, *J* = 7.7, 1.8 Hz, 3H), 6.77 (d, *J* = 5.1 Hz, 1H), 4.61 (dt, *J* = 43.8, 10.1 Hz, 1H), 3.35–3.26 (m, 1H), 3.14 (d, *J* = 4.4 Hz, 1H), 3.08 (q, *J* = 6.3 Hz, 2H), 3.01–2.90 (m, 1H), 2.74 (p, *J* = 7.6 Hz, 1H), 2.03 (dm, *J* = 16.6, 7.9, 4.6 Hz, 6H), 1.96–1.62 (m, 9H). ^13^C NMR (100 MHz, DMSO-*d*_6_) δ 164.16, 141.22, 140.94, 138.17, 138.11, 130.63, 130.49, 129.50, 129.26, 128.42, 128.34, 128.28, 125.60, 60.97, 59.81, 56.86, 55.15, 50.93, 50.82, 32.35, 31.48, 30.89, 30.81, 27.33, 27.28, 21.09, 20.42, 18.63, 18.58. HRMS *m*/*z*: calcd for C_23_H_30_N_2_OS [M + H]^+^: 383.2157; found, 383.2146. Mp 186.0–187.5 °C. The purity of the compound was checked by HPLC (*R*_t_ = 2.863 min) and was found to be 96.12% pure.

#### *N*-(1-(Cyclopentylmethyl)­azocan-5-yl)-*N*-phenylthiophene-3-carboxamide Hydrogen Chloride (**77**)

The title compound was prepared following the general procedure as a white solid in 49.1% yield. ^1^H NMR (400 MHz, DMSO-*d*_6_) δ 9.92 (d, *J* = 36.0 Hz, 1H), 7.38–7.31 (m, 3H), 7.28 (dd, *J* = 5.1, 3.0 Hz, 1H), 7.21 (dt, *J* = 7.9, 1.9 Hz, 3H), 6.77 (dd, *J* = 5.0, 1.3 Hz, 1H), 4.62 (dt, *J* = 38.3, 10.3 Hz, 1H), 3.49–3.38 (m, 2H), 3.24 (dd, *J* = 6.6, 3.2 Hz, 2H), 3.04 (p, *J* = 6.7, 6.2 Hz, 3H), 2.19 (p, *J* = 7.8 Hz, 1H), 2.11–1.98 (m, 4H), 1.93 (dd, *J* = 12.2, 6.6 Hz, 2H), 1.87–1.76 (m, 3H), 1.64–1.56 (m, 2H), 1.54–1.44 (m, 2H), 1.23 (m, *J* = 7.3, 3.7 Hz, 2H). ^13^C NMR (100 MHz, DMSO-*d*_6_) δ 164.17, 138.16, 138.12, 130.62, 130.50, 129.51, 129.28, 128.42, 128.34, 128.29, 125.62, 60.67, 59.31, 51.09, 50.53, 35.32, 35.30, 32.50, 31.79, 31.36, 31.27, 25.05, 20.90, 20.16. HRMS *m*/*z*: calcd for C_24_H_32_N_2_OS [M + H]^+^: 397.2314; found, 397.2326. Mp 189.0–190.2 °C. The purity of the compound was checked by HPLC (*R*_t_ = 2.945 min) and was found to be 98.19% pure.

#### *N*-(1-(Cyclohexylmethyl)­azocan-5-yl)-*N*-phenylthiophene-3-carboxamide Hydrogen Chloride (**78**)

The title compound was prepared following the general procedure as a white solid in 20.3% yield. ^1^H NMR (400 MHz, DMSO-*d*_6_) δ 9.87 (d, *J* = 31.8 Hz, 1H), 7.34 (q, *J* = 7.9, 7.2 Hz, 3H), 7.28 (dd, *J* = 5.1, 3.1 Hz, 1H), 7.21 (d, *J* = 7.8 Hz, 3H), 6.77 (d, *J* = 5.0 Hz, 1H), 4.62 (dt, *J* = 36.7, 10.3 Hz, 1H), 3.27–3.18 (m, 1H), 3.08–2.97 (m, 2H), 2.88 (q, *J* = 5.8 Hz, 2H), 2.04 (t, *J* = 11.0 Hz, 4H), 1.92 (m, *J* = 10.3, 5.8, 4.8 Hz, 2H), 1.85–1.76 (m, 3H), 1.66 (m, *J* = 16.0, 14.6, 7.2 Hz, 6H), 1.30–1.05 (m, 4H), 0.93 (qt, *J* = 12.2, 8.8, 6.3 Hz, 2H). ^13^C NMR (100 MHz, DMSO-*d*_6_) δ 164.17, 138.15, 130.60, 130.51, 129.50, 129.27, 128.42, 128.33, 128.29, 125.61, 61.67, 60.20, 51.12, 50.57, 33.04, 32.90, 32.54, 31.91, 31.07, 30.94, 25.97, 25.45, 20.77, 20.00. HRMS *m*/*z*: calcd for C_25_H_34_N_2_OS [M + H]^+^: 411.2470; found, 411.2477. Mp 188.5–189.4 °C. The purity of the compound was checked by HPLC (*R*_t_ = 3.063 min) and was found to be 98.95% pure.

#### *N*-(1-Benzylazocan-5-yl)-*N*-phenylthiophene-3-carboxamide Hydrogen Chloride (**79**)

The title compound was prepared following the general procedure as a white solid in 48.1% yield. ^1^H NMR (400 MHz, DMSO-*d*_6_) δ 10.92 (d, *J* = 27.0 Hz, 1H), 7.71–7.64 (m, 2H), 7.44 (m, *J* = 6.6, 2.9 Hz, 3H), 7.34 (m, *J* = 6.6, 5.6, 4.6, 2.5 Hz, 3H), 7.28 (dd, *J* = 5.1, 3.0 Hz, 1H), 7.24–7.17 (m, 3H), 6.78 (dd, *J* = 5.0, 1.3 Hz, 1H), 4.64 (dt, *J* = 42.0, 10.2 Hz, 1H), 4.34–4.25 (m, 2H), 3.39–3.29 (m, 2H), 3.27–3.13 (m, 2H), 2.98 (dt, *J* = 13.3, 6.3 Hz, 1H), 2.16–1.86 (m, 7H), 1.84–1.65 (m, 2H). ^13^C NMR (100 MHz, DMSO-*d*_6_) δ 164.17, 164.13, 141.19, 140.95, 138.16, 138.11, 131.77, 131.59, 130.97, 130.61, 130.50, 129.84, 129.74, 129.50, 129.25, 129.19, 128.43, 128.34, 128.29, 125.60, 65.38, 59.17, 57.97, 56.81, 54.92, 50.49, 50.19, 32.48, 31.60, 20.89, 20.28, 15.64. HRMS *m*/*z*: calcd for C_25_H_28_N_2_OS [M + H]^+^: 405.2001; found, 405.2007. Mp 98.9–100.1 °C. The purity of the compound was checked by HPLC (*R*_t_ = 2.883 min) and was found to be 96.45% pure.

#### *N*-(1-Phenethylazocan-5-yl)-*N*-phenylthiophene-3-carboxamide Hydrogen Chloride (**80**)

The title compound was prepared following the general procedure as a white solid in 51.1% yield. ^1^H NMR (400 MHz, DMSO-*d*_6_) δ 10.70 (d, *J* = 84.5 Hz, 1H), 7.29 (m, *J* = 26.8, 23.9, 8.4, 4.8 Hz, 13H), 6.78 (d, *J* = 5.1 Hz, 1H), 4.65 (dt, *J* = 45.1, 9.9 Hz, 1H), 3.55–3.45 (m, 2H), 3.35–3.19 (m, 4H), 3.16–3.01 (m, 3H), 2.07 (m, *J* = 18.2, 11.5, 5.8 Hz, 4H), 1.91 (m, *J* = 21.9, 12.5, 5.1 Hz, 3H), 1.77 (t, *J* = 10.9 Hz, 1H). ^13^C NMR (100 MHz, DMSO-*d*_6_) δ 164.19, 164.13, 141.33, 140.97, 138.22, 138.14, 137.68, 137.64, 130.65, 130.46, 129.51, 129.27, 129.25, 129.23, 129.19, 129.06, 128.43, 128.34, 128.26, 127.22, 125.60, 65.37, 57.53, 57.20, 56.36, 55.28, 51.61, 51.28, 32.22, 31.26, 30.13, 30.04, 21.39, 20.83, 15.64. HRMS *m*/*z*: calcd for C_26_H_30_N_2_OS [M + H]^+^: 419.2157; found, 419.2159. Mp 76.0–77.5 °C. The purity of the compound was checked by HPLC (*R*_t_ = 2.972 min) and was found to be 98.34% pure.

#### *N*-(1-Sllylazocan-5-yl)-*N*-phenyl-1*H*-pyrrole-3-carboxamide Hydrogen Chloride (**81**)

The title compound was prepared following the general procedure as a white solid in 34.2% yield. ^1^H NMR (400 MHz, DMSO-*d*_6_) δ 10.92 (s, 1H), 10.61 (d, *J* = 75.4 Hz, 1H), 7.51–7.38 (m, 3H), 7.24 (dt, *J* = 7.2, 2.2 Hz, 2H), 6.46 (q, *J* = 2.5 Hz, 1H), 6.09–5.92 (m, 2H), 5.60 (p, *J* = 2.6 Hz, 1H), 5.55–5.41 (m, 2H), 4.70 (dt, *J* = 52.3, 9.9 Hz, 1H), 3.69 (dt, *J* = 11.8, 5.8 Hz, 2H), 3.37–3.29 (m, 1H), 3.27–3.09 (m, 2H), 2.97 (dt, *J* = 14.1, 7.0 Hz, 1H), 2.02 (t, *J* = 10.4 Hz, 3H), 1.89 (m, *J* = 17.6, 9.4, 4.1 Hz, 3H), 1.76 (m, *J* = 10.4, 4.6, 3.7 Hz, 1H), 1.64 (q, *J* = 10.9 Hz, 1H). ^13^C NMR (100 MHz, DMSO-*d*_6_) δ 164.20, 141.70, 141.43, 131.33, 131.18, 129.56, 128.55, 128.52, 128.47, 125.19, 124.84, 122.42, 119.54, 119.46, 117.67, 110.31, 110.27, 65.37, 59.25, 58.08, 56.01, 53.76, 51.46, 51.33, 32.42, 31.19, 21.42, 20.87. HRMS *m*/*z*: calcd for C_21_H_27_N_3_O [M + H]^+^: 338.2232; found, 338.2242. Mp 68.0–68.9 °C. The purity of the compound was checked by HPLC (*R*_t_ = 2.565 min) and was found to be 95.77% pure.

#### *N*-(1-(Cyclopropylmethyl)­azocan-5-yl)-*N*-phenyl-1*H*-pyrrole-3-carboxamide Hydrogen Chloride (**82**)

The title compound was prepared following the general procedure as a white solid in 19.2% yield. ^1^H NMR (400 MHz, DMSO-*d*_6_) δ 10.95 (s, 1H), 10.43 (d, *J* = 46.5 Hz, 1H), 7.43 (d, *J* = 5.5 Hz, 3H), 7.31–7.14 (m, 2H), 6.46 (q, *J* = 2.4 Hz, 1H), 6.04 (dd, *J* = 6.4, 2.9 Hz, 1H), 5.60 (d, *J* = 2.3 Hz, 1H), 4.70 (dt, *J* = 39.9, 10.2 Hz, 1H), 3.50–3.38 (m, 2H), 3.36–3.18 (m, 2H), 3.00 (m, *J* = 37.1, 11.7, 6.4 Hz, 4H), 2.15–1.80 (m, 7H), 1.66 (m, *J* = 43.6, 10.9, 9.2 Hz, 2H), 1.10 (m, *J* = 7.2, 2.9 Hz, 1H), 0.69–0.53 (m, 2H), 0.40 (q, *J* = 4.6 Hz, 2H). ^13^C NMR (100 MHz, DMSO-*d*_6_) δ 164.20, 164.15, 141.64, 141.47, 131.30, 131.22, 129.55, 128.52, 128.48, 122.42, 122.40, 119.52, 119.47, 117.67, 110.30, 110.27, 60.54, 59.23, 50.61, 50.52, 32.64, 31.74, 21.08, 20.58, 6.22, 6.16, 4.62. HRMS *m*/*z*: calcd for C_22_H_29_N_3_O [M + H]^+^: 352.2389; found, 352.2377. Mp 207.8–209.0 °C. The purity of the compound was checked by HPLC (*R*_t_ = 2.590 min) and was found to be 98.60% pure.

#### *N*-(1-(Cyclobutylmethyl)­azocan-5-yl)-*N*-phenyl-1*H*-pyrrole-3-carboxamide Hydrogen Chloride (**83**)

The title compound was prepared following the general procedure as a white solid in 36.9% yield. ^1^H NMR (400 MHz, DMSO-*d*_6_) δ 10.91 (s, 1H), 10.16 (d, *J* = 56.7 Hz, 1H), 7.47–7.37 (m, 3H), 7.23 (dt, *J* = 6.8, 1.8 Hz, 2H), 6.46 (q, *J* = 2.4 Hz, 1H), 6.04 (m, *J* = 6.6, 3.4, 1.9 Hz, 1H), 5.60 (p, *J* = 2.5 Hz, 1H), 4.68 (dt, *J* = 43.7, 10.2 Hz, 1H), 3.29 (d, *J* = 12.7 Hz, 1H), 3.18–3.03 (m, 4H), 2.95 (dt, *J* = 13.8, 6.7 Hz, 1H), 2.79–2.66 (m, 1H), 2.12–1.95 (m, 5H), 1.96–1.84 (m, 4H), 1.79 (m, *J* = 13.5, 6.5, 4.0 Hz, 3H), 1.71–1.52 (m, 2H).^13^C NMR (100 MHz, DMSO-*d*_6_) δ 164.19, 164.13, 141.66, 131.31, 131.21, 129.55, 128.52, 128.47, 122.42, 119.53, 119.48, 117.68, 110.30, 110.27, 61.14, 59.87, 51.14, 50.97, 32.56, 31.58, 30.89, 30.82, 27.32, 27.24, 21.13, 20.57, 18.63, 18.56. HRMS *m*/*z*: calcd for C_23_H_31_N_3_O: [M + H]^+^: 366.2545; found, 366.2546. Mp 218.2–218.6 °C. The purity of the compound was checked by HPLC (*R*_t_ = 2.645 min) and was found to be 100% pure.

#### *N*-(1-(Cyclopentylmethyl)­azocan-5-yl)-*N*-phenyl-1*H*-pyrrole-3-carboxamide Hydrogen Chloride (**84**)

The title compound was prepared following the general procedure as a white solid in 26.4% yield. ^1^H NMR (400 MHz, DMSO-*d*_6_) δ 10.90 (s, 1H), 9.85 (d, *J* = 37.0 Hz, 1H), 7.48–7.39 (m, 3H), 7.24 (dt, *J* = 7.0, 1.8 Hz, 2H), 6.50–6.43 (m, 1H), 6.05 (td, *J* = 3.2, 1.5 Hz, 1H), 5.60 (td, *J* = 2.7, 1.4 Hz, 1H), 4.69 (dt, *J* = 34.8, 10.2 Hz, 1H), 3.23 (d, *J* = 5.4 Hz, 2H), 3.03 (m, *J* = 12.3, 6.1, 3.3 Hz, 4H), 2.26–2.12 (m, 1H), 2.05–1.88 (m, 6H), 1.87–1.75 (m, 3H), 1.70–1.55 (m, 4H), 1.51 (m, *J* = 6.5, 5.3, 2.3 Hz, 2H), 1.29–1.16 (m, 2H). ^13^C NMR (100 MHz, DMSO-*d*_6_) δ 162.09, 129.17, 129.11, 127.44, 126.37, 120.31, 117.41, 117.38, 116.39, 115.57, 108.18, 108.16, 63.26, 58.74, 57.26, 49.14, 48.56, 46.94, 33.23, 33.19, 30.61, 29.82, 29.26, 29.15, 22.93, 18.82, 18.19, 13.51. HRMS *m*/*z*: calcd for C_24_H_33_N_3_O [M + H]^+^: 380.2702; found, 380.2680. Mp 206.6–207.4 °C. The purity of the compound was checked by HPLC (*R*_t_ = 2.700 min) and was found to be 97.90% pure.

#### *N*-(1-(Cyclohexylmethyl)­azocan-5-yl)-*N*-phenyl-1*H*-pyrrole-3-carboxamide Hydrogen Chloride (**85**)

The title compound was prepared following the general procedure as a white solid in 24.4%yield. ^1^H NMR (400 MHz, DMSO-*d*_6_) δ 10.95 (s, 1H), 9.91 (d, *J* = 33.9 Hz, 1H), 7.43 (dd, *J* = 5.2, 1.9 Hz, 3H), 7.29–7.18 (m, 2H), 6.46 (q, *J* = 2.4 Hz, 1H), 6.04 (dd, *J* = 4.9, 2.8 Hz, 1H), 5.60 (t, *J* = 2.2 Hz, 1H), 4.81–4.60 (m, 1H), 3.29–3.12 (m, 2H), 3.00 (dt, *J* = 13.5, 6.3 Hz, 1H), 2.87 (dt, *J* = 12.4, 6.1 Hz, 2H), 1.97 (pd, *J* = 17.5, 15.9, 9.6 Hz, 7H), 1.81 (dd, *J* = 11.2, 5.0 Hz, 2H), 1.75–1.48 (m, 6H), 1.32–1.04 (m, 3H), 1.01–0.84 (m, 2H). ^13^C NMR (100 MHz, DMSO-*d*_6_) δ 164.21, 131.28, 131.24, 129.57, 128.55, 122.45, 119.46, 117.72, 110.29, 61.79, 60.15, 51.24, 50.57, 33.04, 32.87, 32.77, 32.04, 31.01, 30.86, 25.96, 25.46, 25.43, 20.76, 20.11. HRMS *m*/*z*: calcd for C_25_H_35_N_3_O [M + H]^+^: 394.2858; found, 394.2869. Mp 190.0–191.7 °C. The purity of the compound was checked by HPLC (*R*_t_ = 2.773 min) and was found to be 97.52% pure.

#### *N*-(1-Benzylazocan-5-yl)-*N*-phenyl-1*H*-pyrrole-3-carboxamide Hydrogen Chloride (**86**)

The title compound was prepared following the general procedure as a white solid in 37.7% yield. ^1^H NMR (400 MHz, DMSO-*d*_6_) δ 10.95 (s, 1H), 10.73 (d, *J* = 33.6 Hz, 1H), 7.65 (dt, *J* = 5.8, 2.1 Hz, 2H), 7.50–7.36 (m, 7H), 7.24 (m, *J* = 7.1, 3.2, 1.8 Hz, 2H), 6.46 (td, *J* = 2.6, 2.0 Hz, 1H), 6.08–6.01 (m, 1H), 5.64–5.57 (m, 1H), 4.83–4.60 (m, 1H), 4.28 (dd, *J* = 8.7, 5.5 Hz, 2H), 3.33 (d, *J* = 11.2 Hz, 1H), 3.27–3.11 (m, 2H), 2.97 (dt, *J* = 13.7, 6.7 Hz, 1H), 2.13–2.00 (m, 3H), 1.98–1.84 (m, 4H), 1.65 (dd, *J* = 18.1, 9.2 Hz, 2H). ^13^C NMR (100 MHz, DMSO-*d*_6_) δ 164.28, 164.23, 131.72, 131.52, 131.26, 131.20, 130.83, 130.79, 129.95, 129.85, 129.59, 129.32, 129.26, 128.57, 122.52, 119.42, 119.37, 117.74, 110.29, 65.39, 59.43, 58.05, 50.53, 49.06, 32.66, 31.58, 20.92, 20.43, 15.61. HRMS *m*/*z*: calcd for C_25_H_29_N_3_O [M + H]^+^: 388.2389; found, 388.2365. Mp 186.6–187.8 °C. The purity of the compound was checked by HPLC (*R*_t_ = 2.677 min) and was found to be 97.56% pure.

#### *N*-(1-Phenethylazocan-5-yl)-*N*-phenyl-1*H*-pyrrole-3-carboxamide Hydrogen Chloride (**87**)

The title compound was prepared following the general procedure as a white solid in 33.4% yield. ^1^H NMR (400 MHz, DMSO-*d*_6_) δ 10.97 (s, 1H), 10.55 (d, *J* = 92.9 Hz, 1H), 7.44 (m, *J* = 3.8, 3.1, 1.8 Hz, 3H), 7.37–7.16 (m, 8H), 6.47 (q, *J* = 2.4 Hz, 1H), 6.05 (m, *J* = 7.7, 3.3, 1.7 Hz, 1H), 5.61 (td, *J* = 2.6, 1.6 Hz, 1H), 4.83–4.61 (m, 1H), 3.48 (t, *J* = 11.7 Hz, 2H), 3.23 (dd, *J* = 15.0, 10.2, 5.0 Hz, 3H), 3.16–2.99 (m, 4H), 2.03 (m, *J* = 11.4, 9.9, 5.7 Hz, 3H), 1.98–1.85 (m, 3H), 1.84–1.73 (m, 1H), 1.65 (q, *J* = 10.6 Hz, 1H). ^13^C NMR (100 MHz, DMSO-*d*_6_) δ 164.27, 164.17, 137.58, 137.55, 131.32, 131.19, 129.60, 129.27, 129.25, 129.07, 128.60, 128.53, 127.25, 122.47, 119.49, 119.42, 117.72, 110.33, 110.29, 65.39, 57.70, 56.38, 51.81, 51.38, 49.06, 32.41, 31.28, 30.15, 30.05, 21.40, 20.94, 15.63. HRMS *m*/*z*: calcd for C_26_H_31_N_3_O [M + H]^+^: 402.2545; found, 402.2522. Mp 181.3–182.8 °C. The purity of the compound was checked by HPLC (*R*_t_ = 2.722 min) and was found to be 96.66% pure.

### Biological Evaluation Drugs

Morphine (morphine sulfate pentahydrate salt) and fentanyl was purchased from Mallinckrodt (St. Louis, MO) or provided by the National Institute of Drug Abuse (NIDA). Naloxone -*d*_5_ was purchased Cerilliant Corp. while all other reagents were purchased from Fisher Scientific). All drugs and test compounds were dissolved in pyrogen-free isotonic saline (Baxter Healthcare, Deerfield, IL) or sterile-filtered distilled/deionized water. All other reagents and radioligands were purchased from either Sigma-Aldrich or Thermo Fisher. Several *in vitro* metabolism studies reported in this manuscript involved human subject samples. These studies were conducted through Contract Research Organizations (CROs). They have provided several documents related to these samples, including Ethics Policy Statement, Current Next of Kin Authorization, and Certification Donor Consent. According to these documents, the sample collection was approved by their local ethics committee (ID 10851), and all participants gave informed consent.

### Animals

Male Swiss-Webster mice (23–35 g, 6–8 weeks, Harlan Laboratories, Indianapolis, IN) were housed five to a cage in animal care quarters maintained at 22 °C on a 12 h light/dark cycle with food and water available ad libitum. The mice were maintained on a 12 h/12 h light–dark cycle (0600-1800 lights on) for the duration of the experiment and were tested during the light segment of this cycle. Mice arrived at the vivarium housed 4/cage and, following 1-week habituation, were separated into individual cages. Mice were allowed to acclimate to individual caging for at least 24 hand then were randomly assigned to the various treatment conditions before the start of studies. Experimenters were blinded to these treatment conditions during the duration of the experiment and data analysis. No adverse events occurred during the experiment, and no mice were excluded from data analysis. Protocols and procedures (Animal Welfare Assurance Number D16-00180) were approved by the Institutional Animal Care and Use Committee (IACUC) at the Virginia Commonwealth University Medical Center and complied with the recommendations of the IASP (International Association for the Study of Pain).

### *In Vitro* Competitive Radioligand Binding Assay and Functional Assay

Competition binding and functional assays were performed using opioid receptors expressed in Chinese hamster ovary (CHO), as previously described.^25^ [^3^H] Naloxone, [^3^H] NTI, and [^3^H]­norBNI (or [^3^H]­DPN) were applied to label the μ, δ, and κ opioid receptors, respectively. In this assay, 20 μg of membrane protein was incubated with 1.4 nM the corresponding radioligand in the presence of different concentrations of test compounds in TME buffer (50 mM Tris, 3 mM MgCl_2_, and 0.2 mM EGTA, pH 7.7) for 1.5 h at 30 °C. After incubation, the bound radioactive ligand was separated from free radioligand by filtration through GF/B glass fiber filters and rinsed three times with ice-cold wash buffer (50 mM Tris-HCl, pH 7.2) using a Brandel harvester. The results were determined by utilizing a scintillation counter. Specific binding was determined as the difference in binding obtained in the absence and presence of 5 μM naltrexone. The IC_50_ values were determined and converted to *K*_i_ values using the Cheng–Prusoff equation. Functional assays were conducted in the same cell membranes used for the receptor binding assays. Membrane proteins (10 μg) were incubated with varying concentrations of drugs, GDP (20 μM), and 0.1 nM ^35^S-GTP­[γS] in assay buffer for 1.5 h at 30 °C. Nonspecific binding was determined with 20 μM unlabeled GTP­[γS]. DAMGO (3 μM), U50,488H (5 μM), and SNC80 (5 μM) were included in the assay for a maximally effective concentration of a full agonist for the μ, κ, and δ opioid receptors, respectively.

### Calcium Mobilization Assay

mMOR-CHO cells were cultured with DMEM/F-12 supplemented with 10% FBS at 37 °C and 5% CO_2_. The cells were transfected with Gqi4 cDNA using lipofectamine 2000 medium OPTI according to the manufacturer’s recommended procedure. Then, the cells were incubated for 24 h before being plated to a clear bottom, black 96-well assay plate at 20,000 cells/well in cell growth media. Cells were ready for calcium mobilization assay after 44–48 h incubation. 50 μL of loading buffer was added to each well in the assay plate, followed by 1 h incubation. The positive control and varying concentrations of the testing compound were added to a source plate (for antagonist measurement, 20 μL of the testing compound was then added to each well and incubated for another 15 min). Before the measurement, the loading buffer was decanted, and 80 μL/well of the washing buffer was added to the 96-well plate. Subsequently, the assay plates were read on a FlexStation3 microplate reader at 494/516 ex/em. The changes in fluorescence were monitored, and peak height values were obtained using SoftMaxPro software (Molecular Devices). Nonlinear regression curves and IC_50_ values were generated using GraphPad Prism 8.0. All concentrations were tested in triplicate, and all experiments were repeated at least three times.^43^

### Warm-Water Immersion Assay

The antinociceptive effect of synthesized compounds was determined using the warm-water tail immersion assay.^73^ 6–8-week 25–35 g male Swiss Webster mice were housed in cages (five maximal per cage) in animal care quarters and maintained at 22 ± 2 °C on a 12 h light-dark cycle. Food (standard chow) and water were available ad libitum. The mice were brought to the laboratory (22 ± 2 °C, 12 h light-dark cycle) and allowed 18 h to recover from the transport. The tail-flick test was performed using a water bath with the temperature maintained at 56 ± 0.1 °C. Each mouse was gently wrapped in a cloth with only the tail exposed. Baseline latency was measured before s.c. injection of the compounds. The distal one-third of the tail was immersed perpendicularly in water, and the mouse rapidly flicked his tail from the bath at the first sign of discomfort. The duration of time the tail remained in the water bath was counted as the baseline latency. Untreated mice with baseline latency reaction times ranging from 2 to 4 s were used. Test latency was obtained 20 min later after the agonist injection. A 10 s maximum cutoff latency was used to prevent any tissue damage. Antinociception was quantified as the percentage of maximal possible effect (% MPE), which was calculated as % MPE = [(test latency – control latency)/(10 – control latency)] × 100. The % MPE value was calculated for each mouse using six mice per compound. If the compound was evaluated for its antagonizing effects against morphine or fentanyl, the compound was s.c. injected 5 min prior to the agonist administration. AD_50_ values were calculated using the least-squares linear regression analysis followed by calculation of 95% confidence interval by the Bliss method.

### *In Vivo* BBB Penetration Studies

Following our previously reported protocol,^47,74^ Swiss Webster mice (three mice each time point) were given compound **53** (10 mg/kg, s.c.). At 5-, 10-, 30-, and 60 min time points post administration, the mice were decapitated, and whole brain and blood samples were collected. Brain samples were washed with saline to ensure removal of any blood on the isolated brains. They were then immersed in 300 μL saline. Blood samples were centrifuged for 10 min at 15,000*g* at 4 °C following which plasma was collected. Brain and plasma samples were stored at −80 °C until further analysis.^75^

### UPLC-MS/MS Analysis

The identification and quantification of compound **53** in mouse plasma and brain were performed using a modification of a previously described method with naloxone-*d*_5_ as the internal standard.^2^ Prior to extraction, brain tissues were homogenized with deionized water at a 1:3 (w/w) ratio using an Omni Bead Ruptor (Omni International Inc., Kennesaw, GA). Each analytical run included seven-point calibration curves (10–1000 ng/mL or ng/g) for compound **53**, quality control samples at 30, 300, and 750 ng/mL or ng/g, as well as negative and blank controls, all prepared in plasma or brain homogenate. After mixing, 100 μL of 5 M ammonium hydroxide and 2 mL of a 25:75 methylene chloride-diethyl ether mixture were added. Samples were vortexed for 2 min and centrifuged at 3000 rpm for 5 min. The organic layer was evaporated under nitrogen and reconstituted with 100 μL of mobile phase before LC-MS/MS analysis. Chromatography was performed on a Sciex ExionLC 2.0+ system coupled to a Sciex 6500 QTRAP with an IonDrive Turbo V source (Sciex, Ontario, Canada), using a Zorbax Eclipse column (4.6 × 75 mm, 3.5 μm; Agilent, USA) and an isocratic mobile phase of 10 mM ammonium formate:methanol (50:50, v/v) at 0.6 mL/min. Source conditions included a temperature of 600 °C, curtain gas at 30 mL/min, ion spray voltage of 5000 V, and ion source gases 1 and 2 at 50 and 30 mL/min, respectively. Data were acquired in positive-ion mode using multiple reaction monitoring (MRM) with the following transitions (*m*/*z*), and collision energy (eV) in parentheses: Compd. **53**, 339 > 152 (24) and 339 > 140 (49). Total run time was 4 min. Quantification was performed using linear regression of analyte-to-ISTD peak area ratios from the calibration curves.

### *In Vitro* Metabolism

Metabolic stability, expressed as percent of the parent compound remaining, was calculated by comparing the peak area of the compound at the time point relative to that at time-0. The half-life (*T*_1/2_) was estimated from the slope of the initial linear range of the logarithmic curve of compound remaining (%) vs time, assuming the first-order kinetics.^76,77^

The apparent intrinsic clearance (CL_int_, in μL/min/pmol, μL/min/mg or μL/min/Mcell) was calculated according to the following formula:

$${CL}_{\mathrm{int}}=\frac{0.693}{T_{1/2}\times \left(\mathrm{mg protein}/\mu \mathrm{L or million cells}/\mu \mathrm{L or pmol CYP isoyme}/\mu L\right)}$$

### *In Vitro* Absorption

The apparent permeability coefficient (*P*_app_) of the test compound was calculated as follows:

$$P_{app}\;\left(\mathrm{cm}/s\right)=\frac{V_{R}\times C_{R,end}}{\Delta t}\times \frac{1}{A\times \left(C_{D,\mathrm{mid}}-C_{R,\mathrm{mid}}\right)}$$

where *V*_R_ is the volume of the receiver chamber. *C*_R,end_ is the concentration of the test compound in the receiver chamber at the end time point, Δ*t* is the incubation time and *A* is the surface area of the cell monolayer. *C*_D,mid_ is the calculated midpoint concentration of the test compound in the donor side, which is the mean value of the donor concentration at time 0 min and the donor concentration at the end time point. *C*_R,mid_ is the midpoint concentration of the test compound in the receiver side, which is one-half of the receiver concentration at the end time point. Concentrations of the test compound were expressed as peak areas of the test compound.

Recovery of the Test Compound from the Permeability Assay. The recovery of the test compound was calculated as follows:

$$\mathrm{Recovery}\;\left(\%\right)=\frac{V_{D}\times C_{D,\mathrm{end}}+V_{R}\times C_{R,\mathrm{end}}}{V_{D}\times C_{D0}}\times 100$$

where *V*_D_ and *V*_R_ are the volumes of the donor and receiver chambers, respectively. *C*_D,end_ is the concentration of the test compound in the donor sample at the end time point. *C*_R,end_ is the concentration of the test compound in the receiver sample at the end time point. *C*_D0_ is the concentration of the test compound in the donor sample at time zero. Concentrations of the test compound are expressed as peak areas of the test compound.

Fluorescein assessment for Permeability assays. Fluorescein was used as the cell monolayer integrity marker. Fluorescein permeability assessment (in the A–B direction at pH 7.4 on both sides) was performed after the permeability assay for the test compound. The cell monolayer that had a fluorescein permeability of less than 1.5 × 10^–6^ cm/s for Caco-2 and MDR1-MDCKII cells and 2.5 × 10^–6^ cm/s for MDCKII cells was considered intact, and the permeability result of the test compound from intact cell monolayer is reported.

Efflux Transporter Substrate Assessment. The Efflux Ratio (ER) was calculated as follows:

$$\mathrm{ER}=\frac{P_{\mathrm{app}}\left(B-A\right)}{P_{\mathrm{app}}\left(A-B\right)}$$

Where *P*_app_(B–A) is the apparent permeability coefficient in the B to A direction, and *P*_app_(A–B) is the apparent permeability coefficient in the A to B direction. A compound is considered as a substrate of an efflux transporter if the Efflux Ratio ≥ 2; and the transporter selective inhibitor inhibits the Efflux Ratio more than 50%.

### Measurement of Respiration

6–8 week 25–35 g male Swiss Webster mice were housed in cages (five maximal per cage) in animal care quarters and were maintained at 22 ± 2 °C on a reversed 12 h dark–light cycle. All experiments were conducted in the dark (active) phase. Respiration was measured using WBP chambers (EMKA Technologies, France) in freely moving mice. The chambers were supplied with an air mixture containing 5% CO_2_. A 10 min baseline respiration period was recorded prior to any administration. The rate and depth of respiration were recorded and averaged over 1- or 5 min periods. Tidal volume was calculated from the raw inspiration data and expiration data. Minute volume was then calculated as rate x tidal volume. The first compound was administered s.c., and respiration was recorded for 5 min. Then, respiration was recorded for a period of 30 min after the second injection.

### Statistical Analysis

One-way ANOVA followed by the posthoc Dunnett test were performed to assess significance using Prism 6.0 software (GraphPad Software, San Diego, CA).

### Molecular Docking Studies

A docking study was then conducted to elucidate the binding mode of compound **53** in the inactive mu-opioid receptor (MOR) crystal structure, aiming to provide deeper insights into its mechanism of action. The compound **53** was initially sketched using Sybyl X2.1, with Gasteiger–Hückel charges assigned, followed by energy minimization (100,000 iterations) to a gradient of 0.05 kcal/(mol·Å) using the Tripos Force Field. The X-ray crystal structure of the antagonist-bound MOR (PDB ID: 4DKL)^65^ was retrieved from the Protein Data Bank (http://www.rcsb.org). To prepare the receptor for docking, hydrogen atoms were added, water molecules and bound ligands were removed, and missing residues in the intracellular loop 3 (ICL-3) region were modeled using Sybyl 8.0 (Tripos, MO, USA). Molecular docking was performed using the genetic algorithm-based docking program GOLD 2020.^78^ Consistent with known opioid ligands, the carboxylate group of Asp147 (D147) in the orthosteric site formed an ionic interaction with the protonated nitrogen atom of the ligand’s amino group. The binding site was defined as atoms within 10 Å of the γ-carbon atom of D147, with a distance constraint applied between the quaternary amine of the ligand and the carboxylate group of D147. The docking results were analyzed based on the highest CHEM-PLP scores, with molecular interactions visualized using the PyMOL Molecular Graphics System.

## Supplementary Material







## Notes

Molecular formula strings and some data (CSV)
3D PDB docking study (PDB)
Spectra data for target compounds (^1^H, ^13^C NMR, and Purity of final compounds HPLC graph); Effects of Compound **53** on ventilation in mice (PDF)

## Abbreviations Used

BBB: blood-brain barrier
CHO: Chinese hamster ovary
CL: confidence level
CNS: central nervous system
DAMGO: [d-Ala2-MePhe4-Gly­(ol)-5] enkephalin
DOR: δ opioid receptor
GPCR: G-protein-coupled receptor
HPLC: high-performance liquid chromatography
HRMS: high-resolution mass spectroscopy
KOR: κ opioid receptor
MOR: μ opioid receptor
NIDA: National Institute on Drug Abuse
NIH: National Institutes of Health
NLX: naloxone
NTX: naltrexone
OUD: opioid use disorder
% MPE: percentage maximum possible effect
SAR: structure–activity relationship
WWTI: warm-water tail immersion
β-FNA: β-funaltrexamine
