# Supplementary material for: Design, Syntheses, and Pharmacological Evaluations of Core Ring Expanded Fentanyl Analogues as Potential Counteracting Agents Against Fentanyl Induced Respiratory Depression
Source: J Med Chem. 2025 Nov 3;68(21):22170–95. doi: 10.1021/acs.jmedchem.5c00528 (PMC12621185; doi:10.1021/acs.jmedchem.5c00528)

Supporting Information

Design, Syntheses, and Pharmacological Evaluations of Core Ring Expanded Fentanyl Analogues as Potential Counteracting Agents Against Fentanyl Induced Respiratory Depression

Abeje A. Silte[a], Ennian Li[a], Balaji S. Kale[a], Logan Neel[a], Neha Upadhyay[a], Rachael Flammia[a], Rui Lyu[a], Celsey M. St. Onge[a], Ahmed Reda[a], Samuel Woodard[b], James C. Gillespie[b], Daniel Kim[b], Dana E. Selley[b], William L. Dewey[b], Piyusha P. Pagare[a], Yan Zhang*[a,b,c,d]

[a] Department of Medicinal Chemistry, School of Pharmacy, Virginia Commonwealth University, 800 East Leigh Street, Richmond, Virginia 23298, United States

[b] Department of Pharmacology and Toxicology, School of Medicine, Virginia Commonwealth University, 410 North 12th Street, Richmond, Virginia 23298, United States

[c] Center for Drug Discovery, Virginia Commonwealth University, 800 East Leigh Street, Richmond, Virginia 23298, United States

[d] Institute for Drug and Alcohol Studies, Virginia Commonwealth University, 203 East Cary Street, Richmond, Virginia 23298, United States

* Corresponding author: Tel: +1 (804) 828-0021. E-mail address: yzhang2@vcu.edu (Y. Zhang).

Table of Contents

1. *Figure S1. Predominant configuration of compound 4* 2
2. *Figure S2. Calcium flux assay* 5
3. *Figure S3. Respiratory depression of compound 53* 6
4. *^{1}H and ^{13}C NMR spectra of novel intermediates and final compounds* 7
5. *Table S1. Purity data of final compounds* 104
6. *HPLC chromatograms of final compounds.* 108

1. **Figure S1**. Predominant configuration of compound **4**

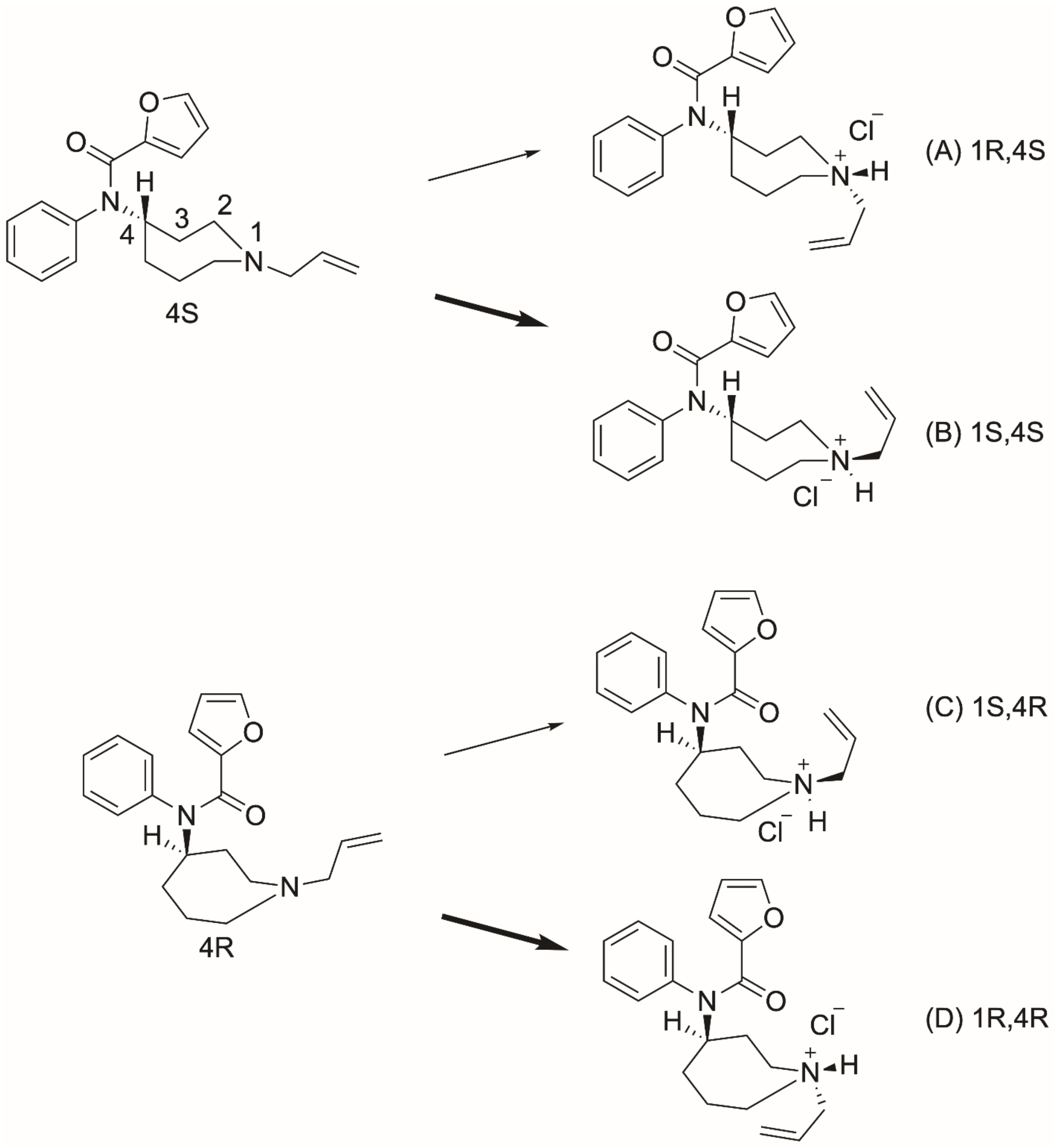

While enantiomers are indistinguishable by standard NMR spectroscopy, the formation of hydrogen chloride salts for the final compounds leads to the appearance of distinct multiple peaks in the NMR spectra. This observation indicates a structural differentiation, consistent with the formation of diastereomers. As Van Bever et al. observed[1], the spectral data from a diastereoisomeric mixture didn't display enough unique characteristics for an unambiguous structural assignment. Further study of the potent compounds will be conducted as part of the ongoing project. We believe the cycloheptane compound exists primarily as two interconverting chair conformations, undergoing a rapid ring flip[2]. As showed in **Figure S1**, we used compound **4** as an example to illustrate the predominant configuration. The bulky substituent prefers the equatorial position (as in structures B_ (1S, 4S) and D_ (1R, 4R)) because this configuration experiences less steric strain and is therefore more stable (lower in energy) than when the bulky group

is axial (A_ (1R, 4S) and C_ (1S, 4R)). This explains the two sets of peaks observed in the NMR spectrum.

(1) Van Bever, W. F. M.; Niemegeers, C. J. E.; Janssen, P. A. J. Synthetic Analgesics. Synthesis and Pharmacology of the Diastereoisomers of N-[3-Methyl-1-(2-Phenylethyl)-4-Piperidyl]-N-Phenylpropanamide and N-[3-Methyl-1-(1-Methyl-2-Phenylethyl)-4-Piperidyl]-N-Phenylpropanamide. *J. Med. Chem.* **1974**, *17* (10), 1047–1051. https://doi.org/10.1021/jm00256a003.

(2) Jahn, M. K.; Dewald, D. A.; Vallejo-López, M.; Cocinero, E. J.; Lesarri, A.; Zou, W.; Cremer, D.; Grabow, J.-U. Pseudorotational Landscape of Seven-Membered Rings: The Most Stable Chair and Twist-Boat Conformers of ε-Caprolactone. *Chemistry – A European Journal* **2014**, *20* (43), 14084–14089. https://doi.org/10.1002/chem.201403379.

Figure S2. Calcium flux assay

Calcium flux assay of compounds **16**, **46**, **53** and **69** in Gαqi4-transfected mMOR-CHO cells. Compound **16**, **46**, **53** and **69** exhibited no apparent agonism to increase the intracellular calcium level.

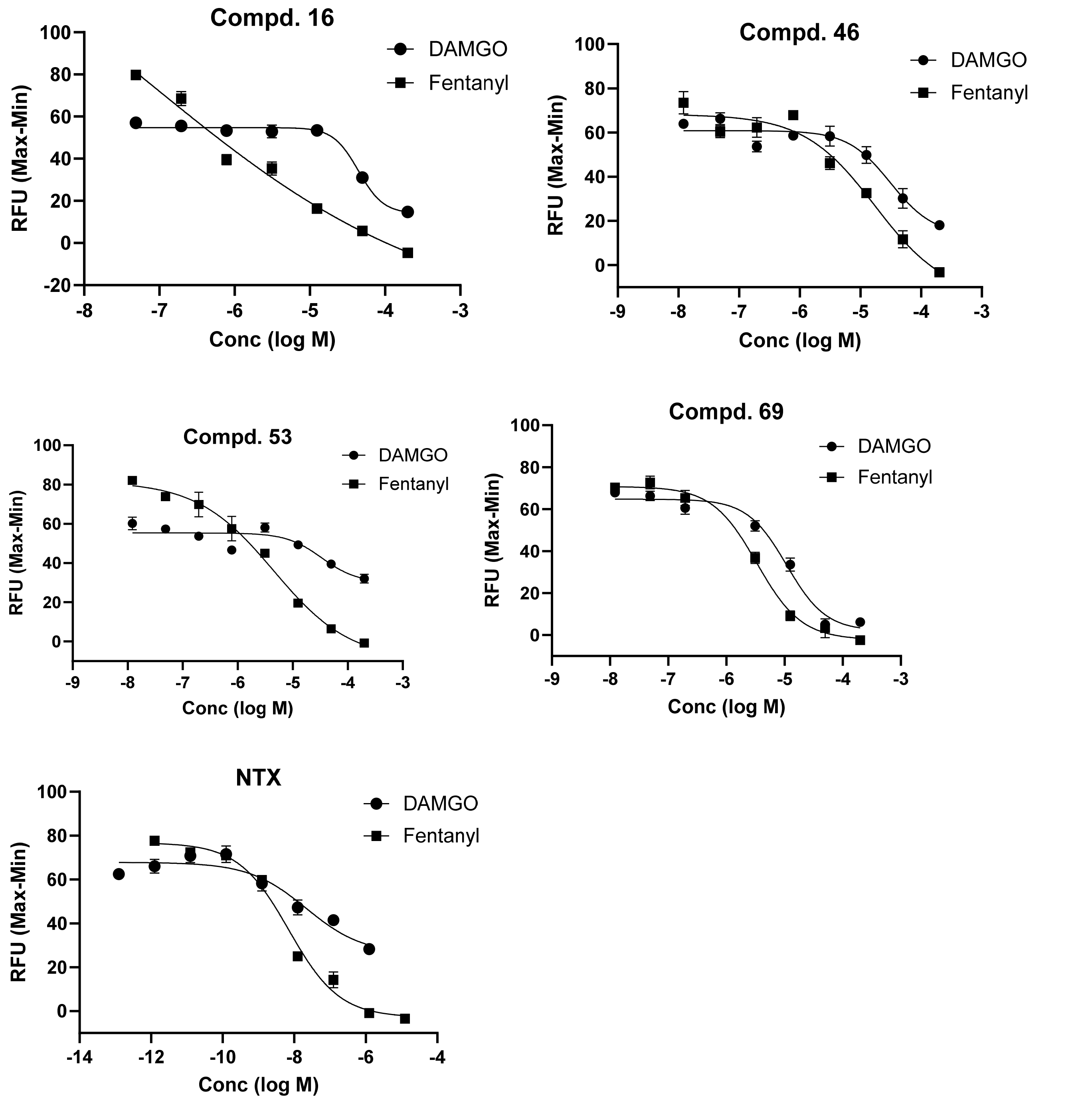

2. **Figure S3.** Respiratory depression of compound **53**

Effects of Compound **53** on ventilation in mice. **(A)** Minute volume; **(B)** Respiratory rate and **(C)** Tidal volume. Error bars represent the standard error of normalized mean values within individual 5 min bins. Closed symbols indicate significant differences compared to the saline (SAL + SAL)-treated controls at individual timepoints ($p \leq 0.05$) via one-way ANOVA.

A.

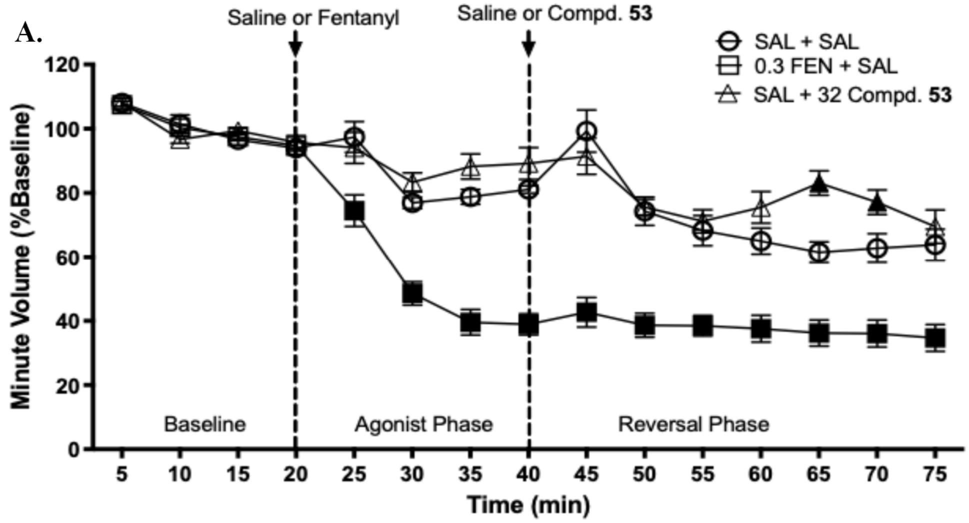

B.

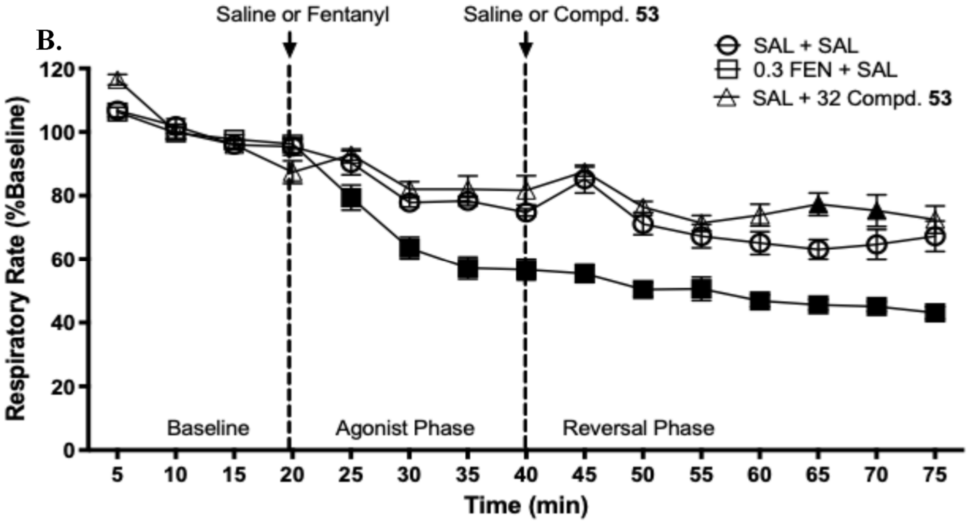

C.

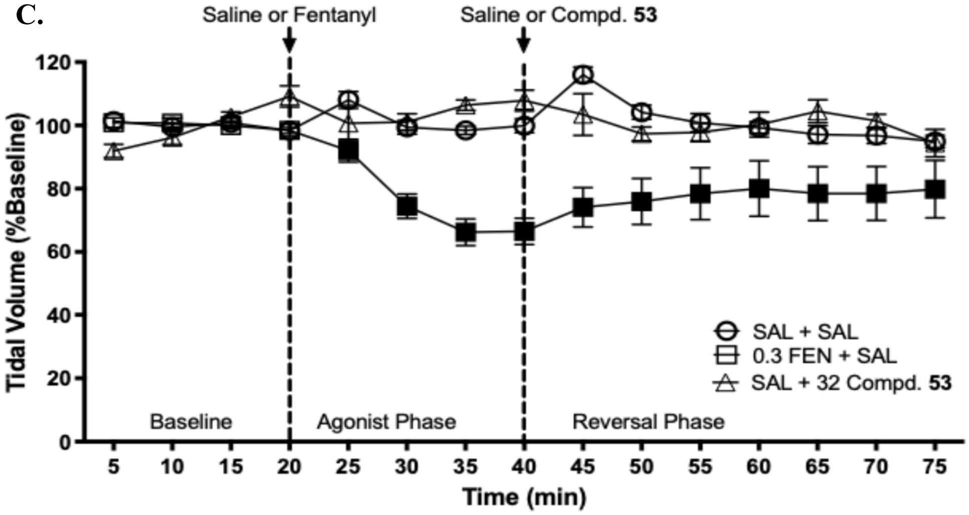

3. ^{1}H and ^{13}C NMR spectra of novel intermediates and final compounds

tert-butyl 4-(phenylamino)azepane-1-carboxylate **(1a)**

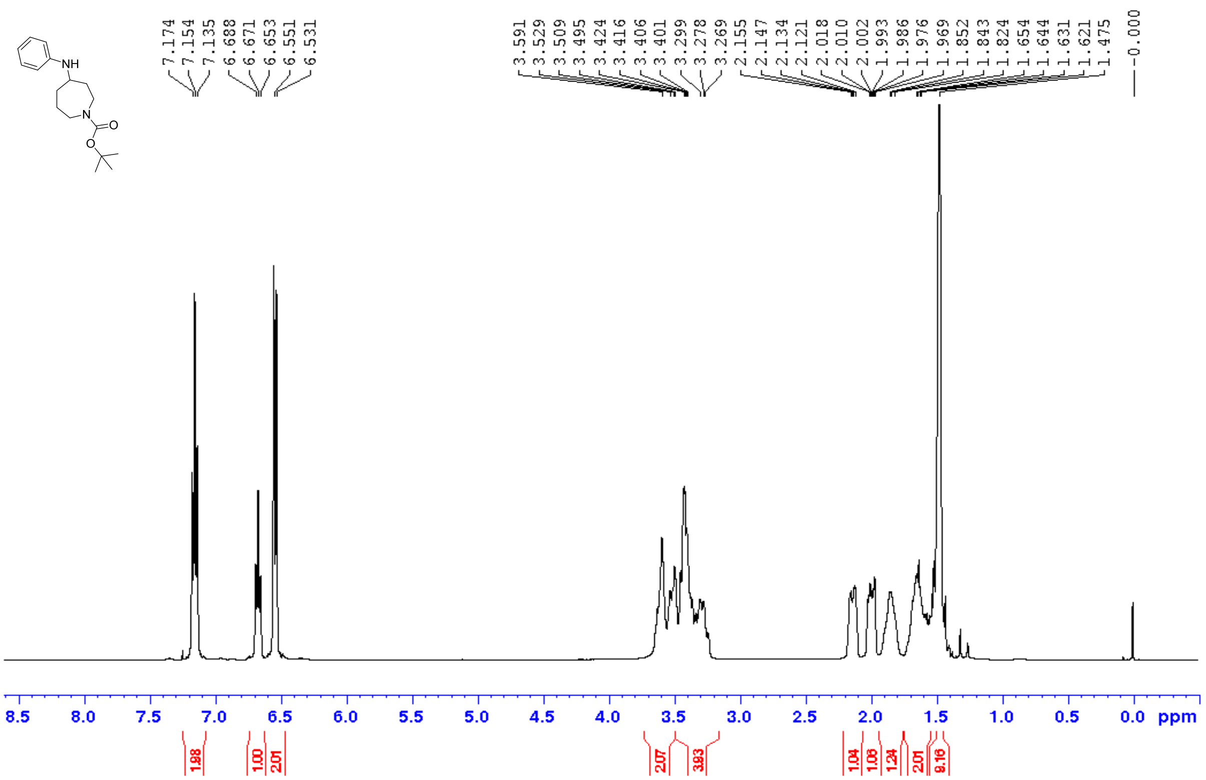

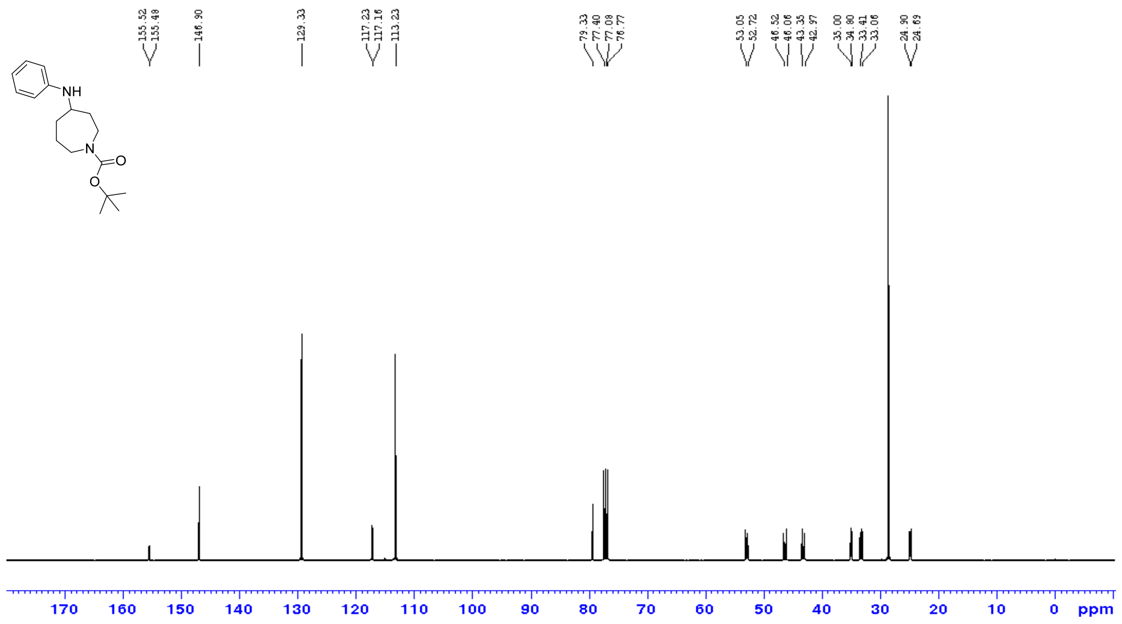

tert-butyl 5-(phenylamino)cyclooctane-1-carboxylate (1a’)

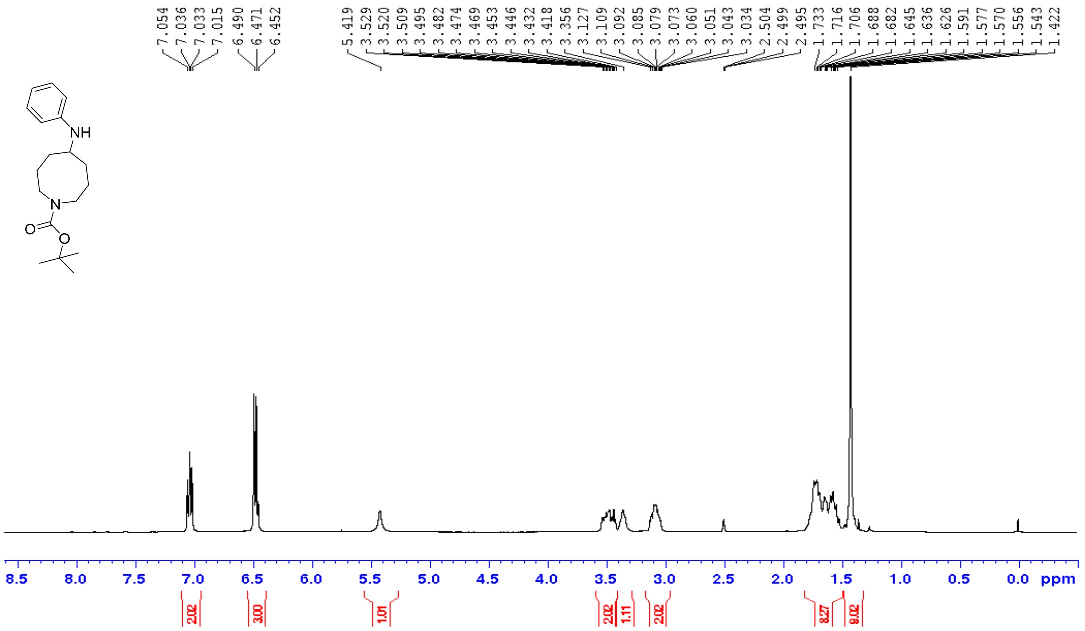

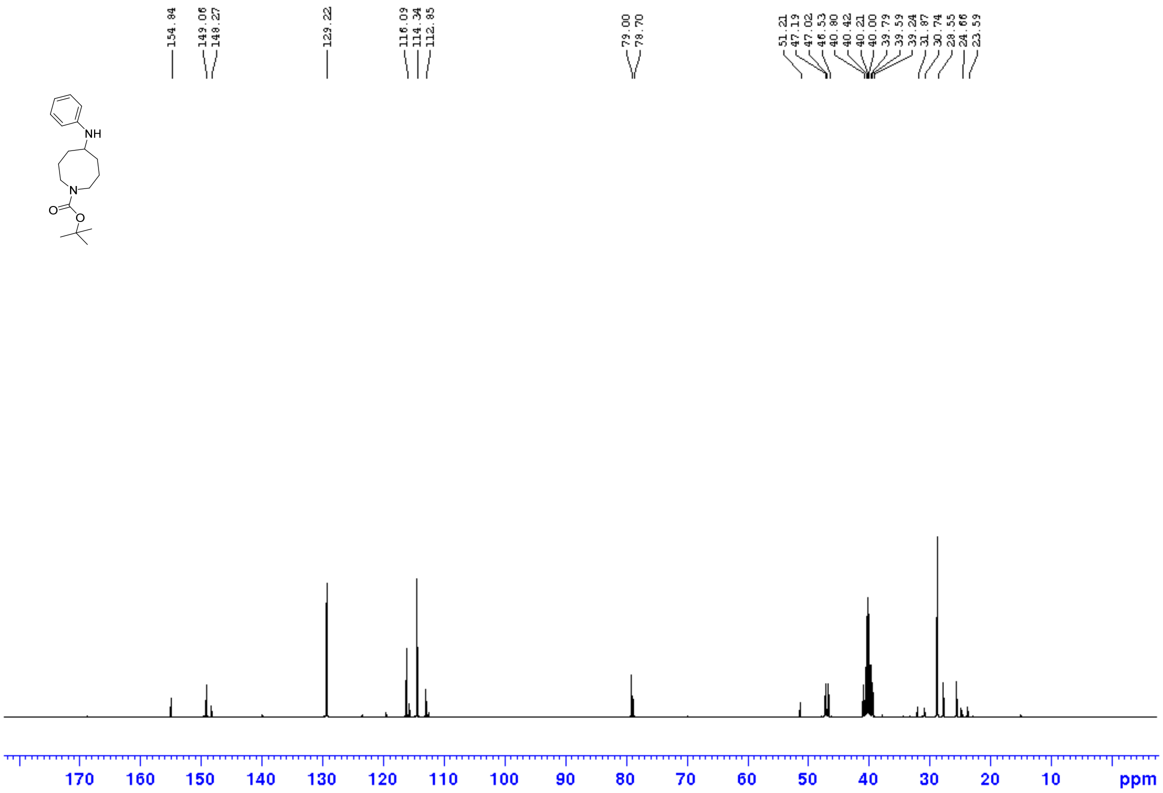

tert-butyl 4-(N-phenylfuran-2-carboxamido)azepane-1-carboxylate **(2a)**

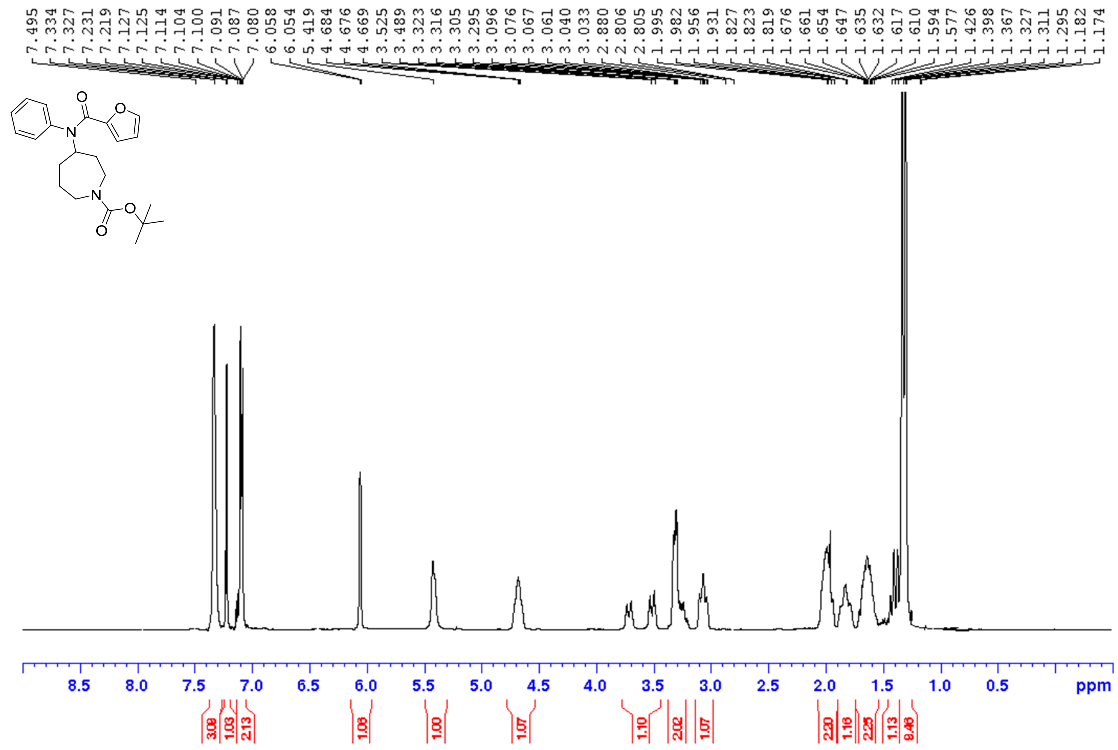

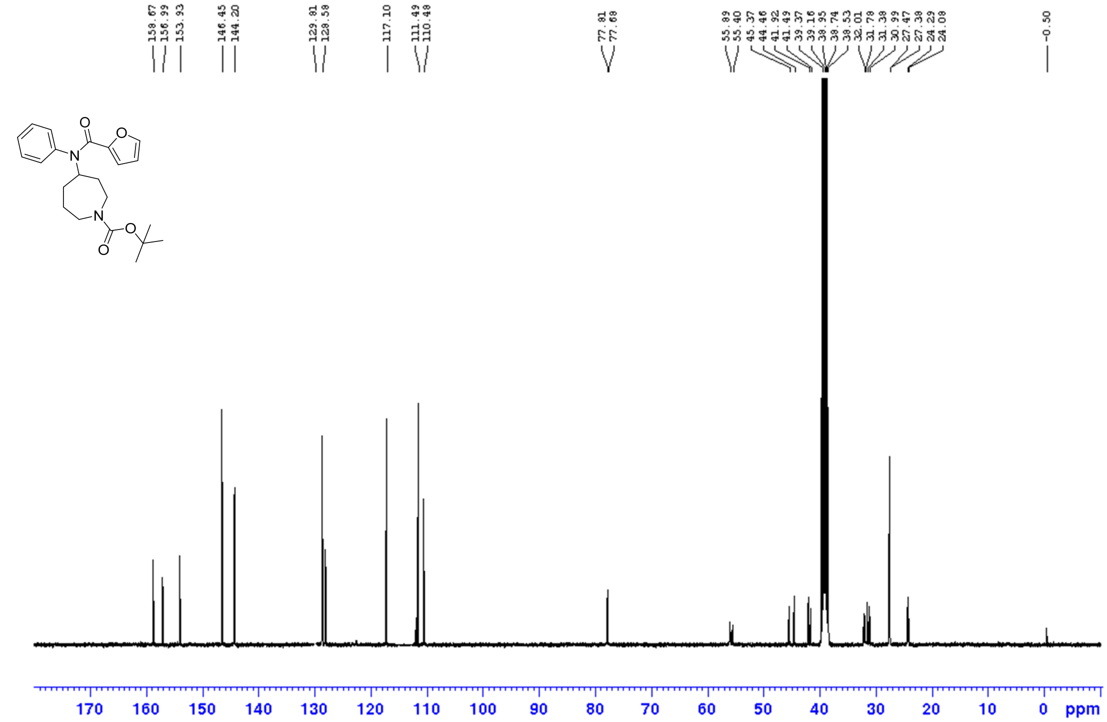

tert-butyl 4-(N-phenylthiophene-2-carboxamido)azepane-1-carboxylate**(2b)**

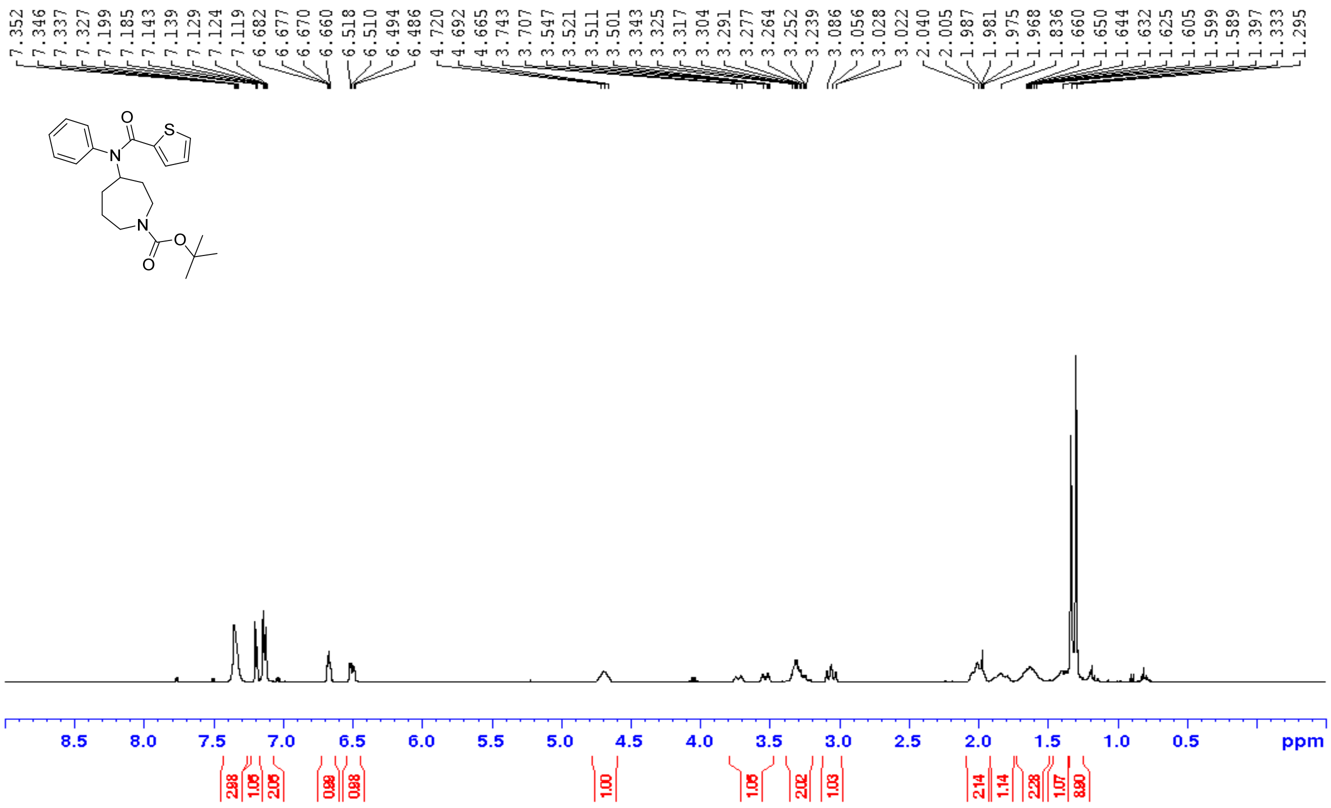

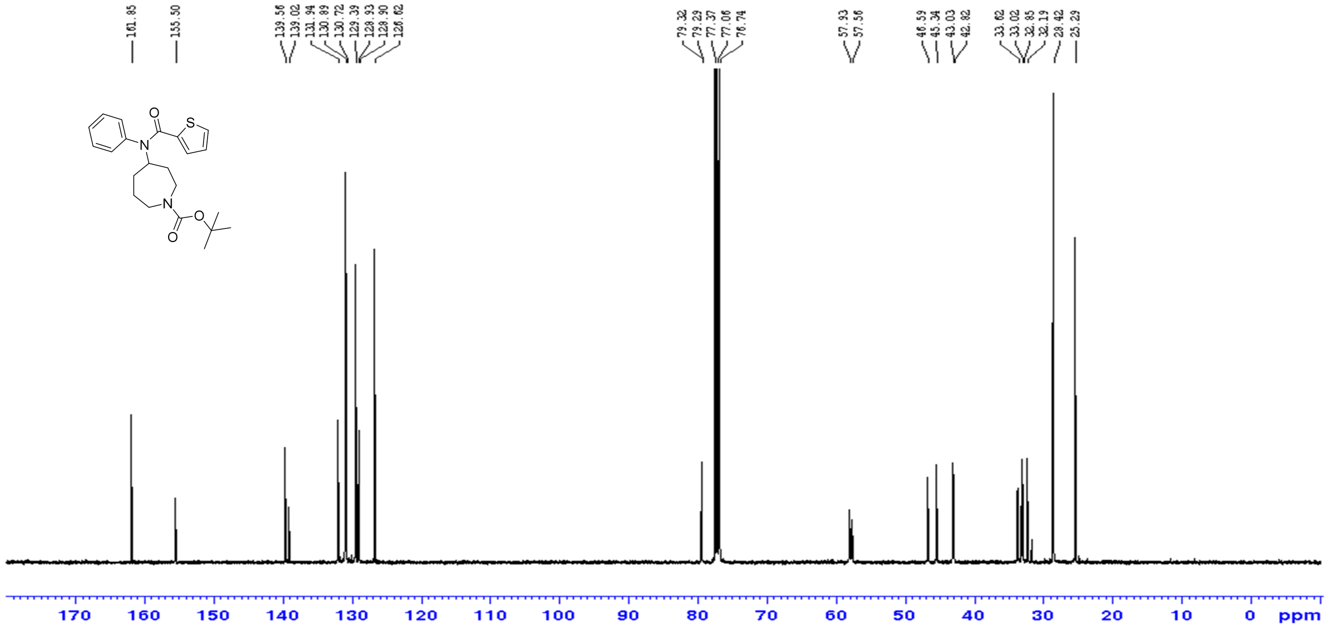

tert-butyl 4-(N-phenylfuran-3-carboxamido)azepane-1-carboxylate**(2c)**

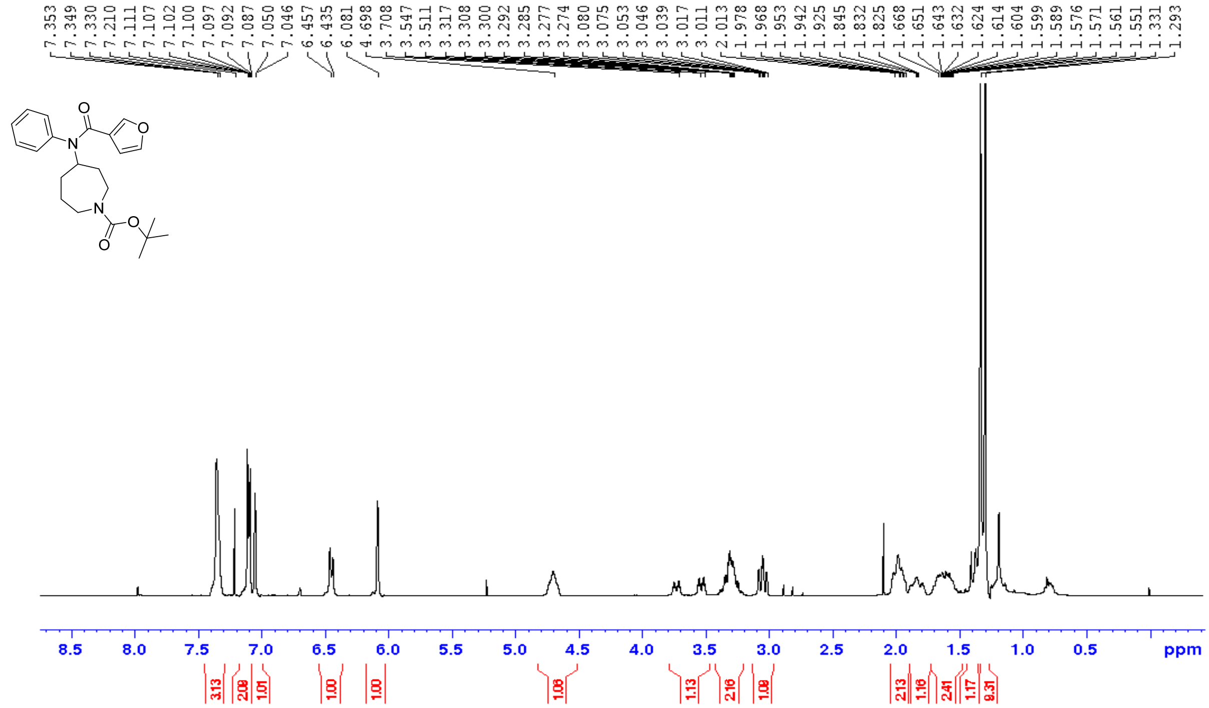

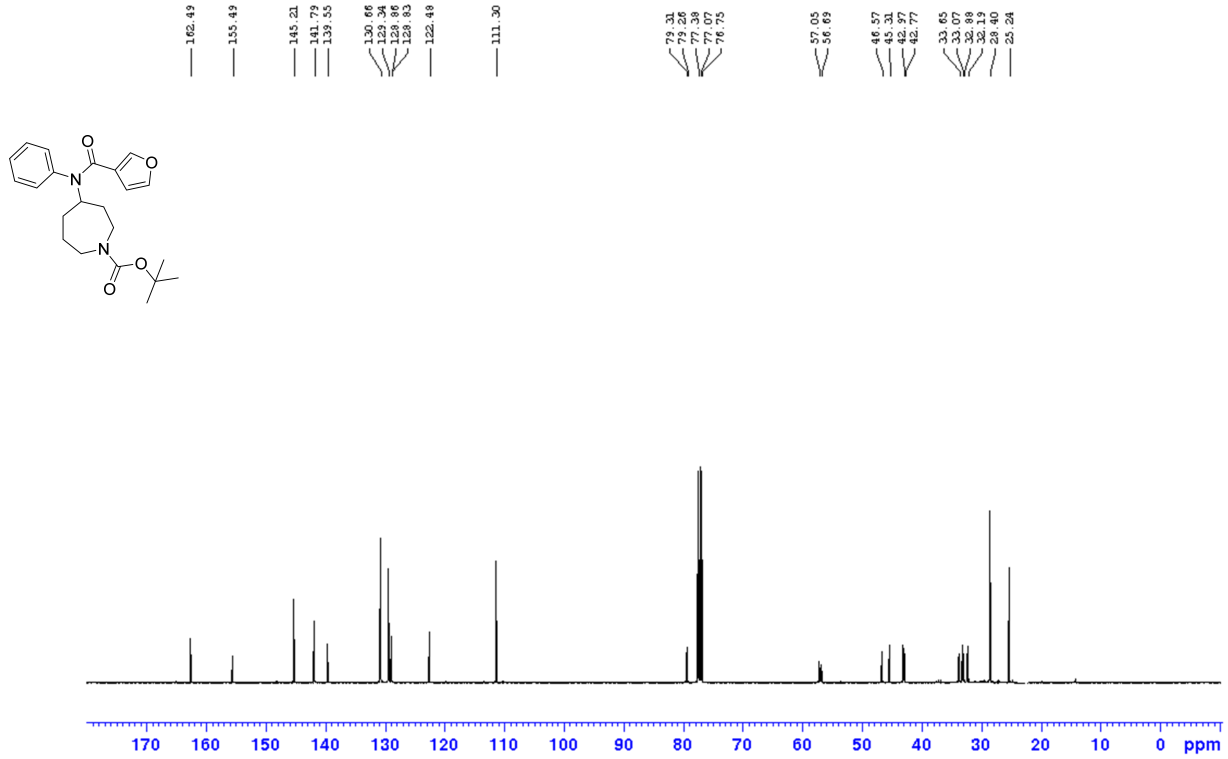

tert-butyl 4-(N-phenyl-1H-pyrrole-2-carboxamido)azepane-1-carboxylate **(2d)**

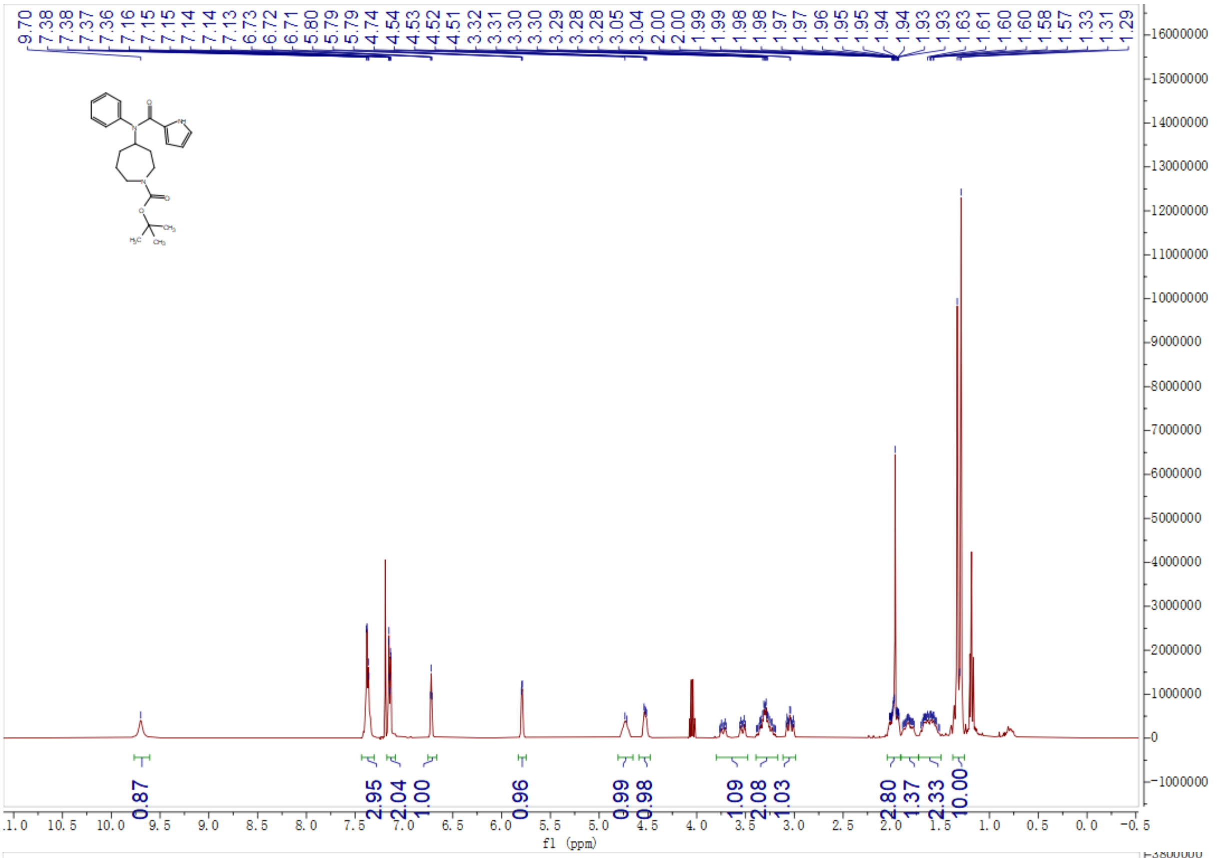

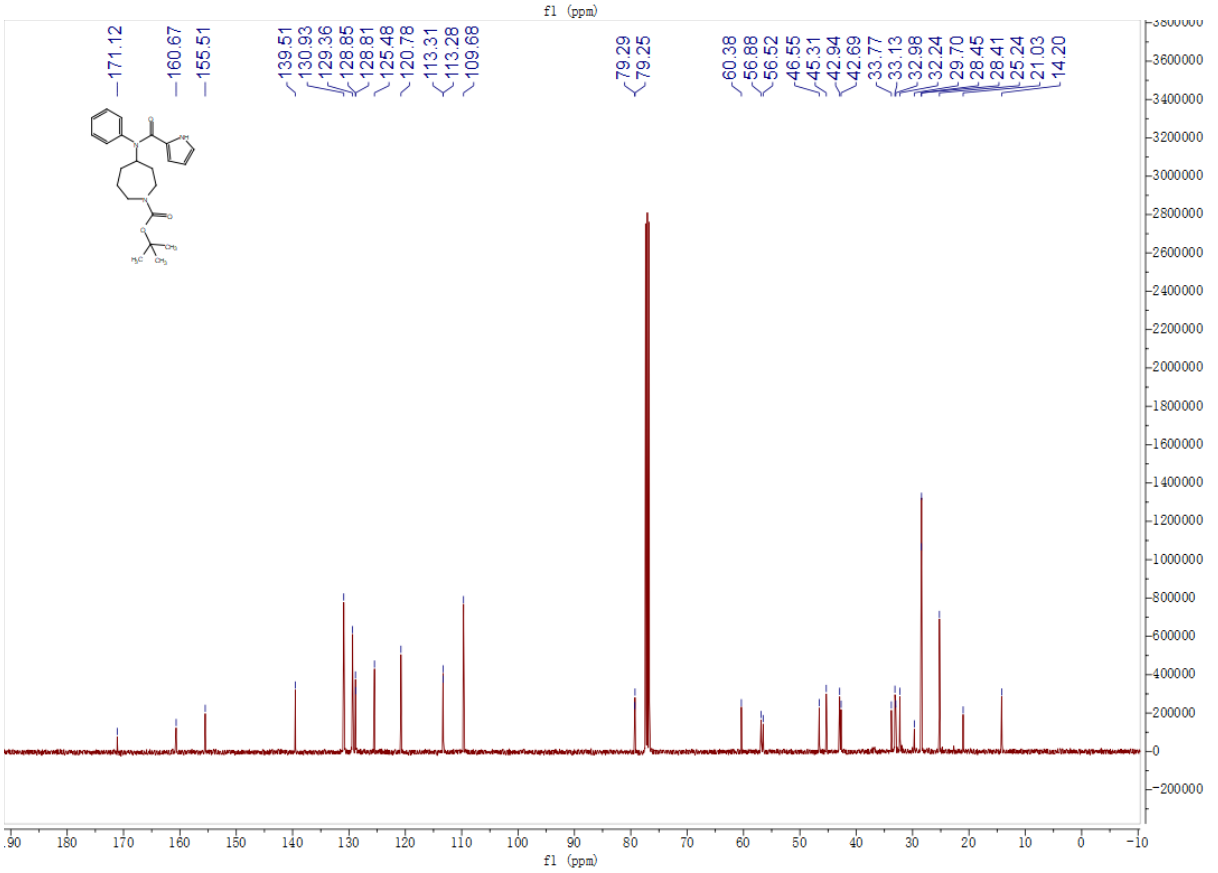

tert-butyl 4-(N-phenyl-1H-pyrrole-3-carboxamido)azepane-1-carboxylate**(2e)**

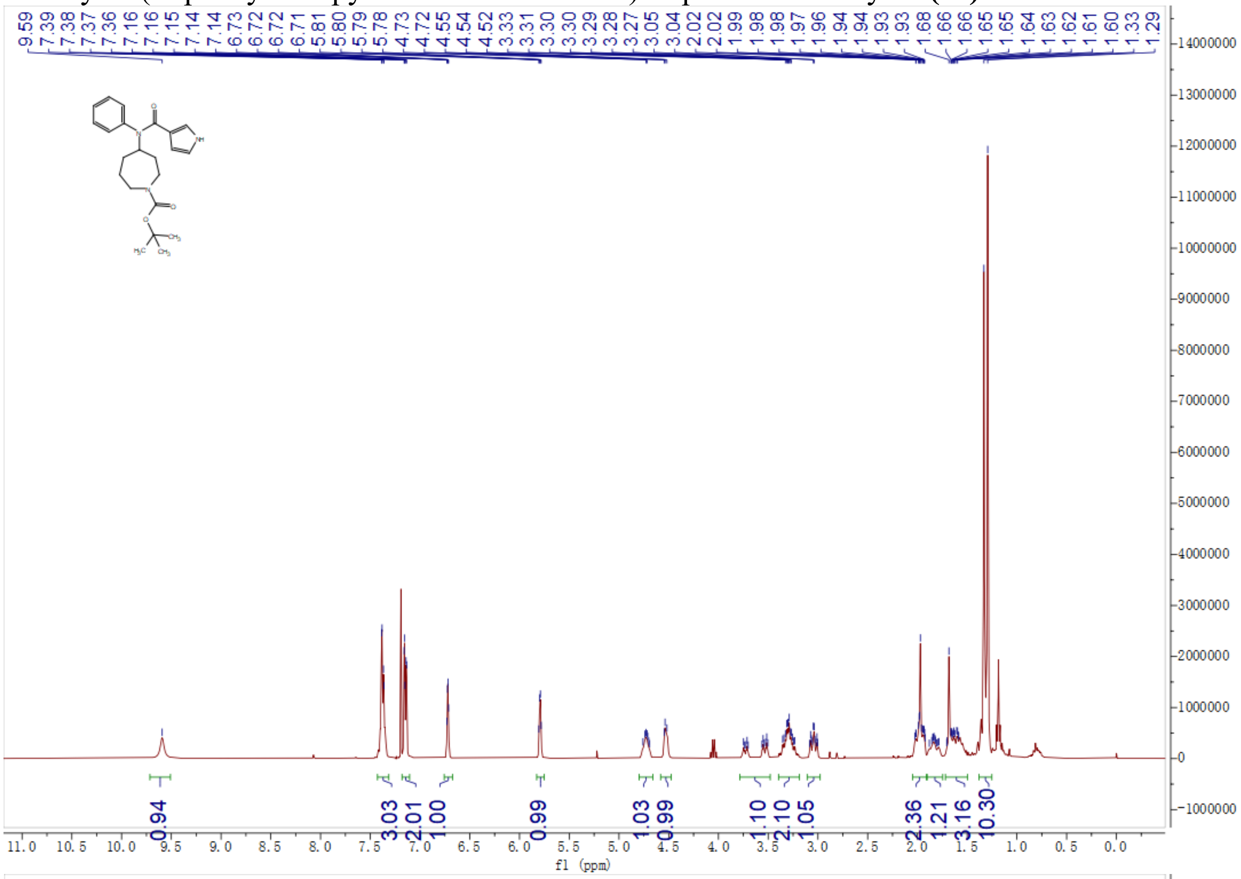

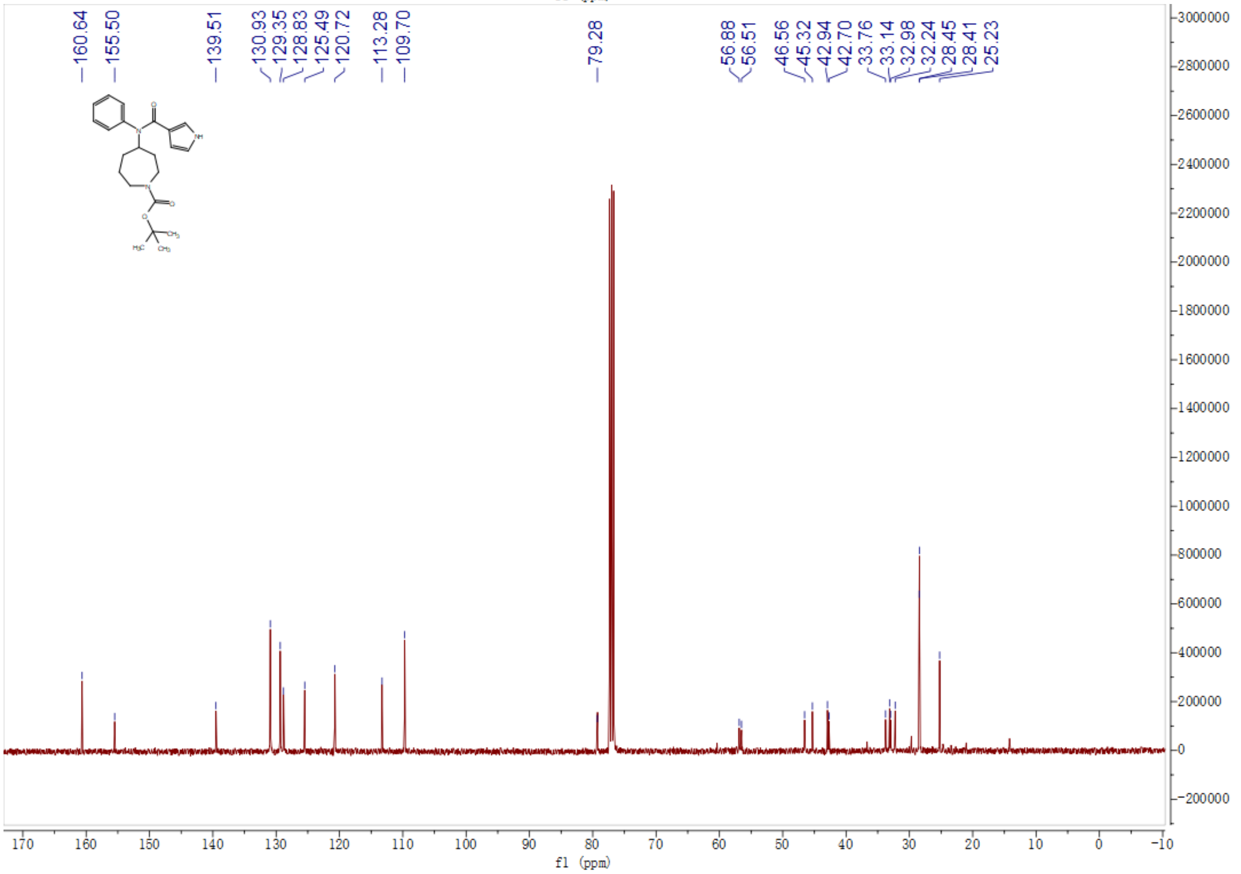

tert-butyl 4-(N-phenylthiophene-3-carboxamido)azepane-1-carboxylate(**2f**)

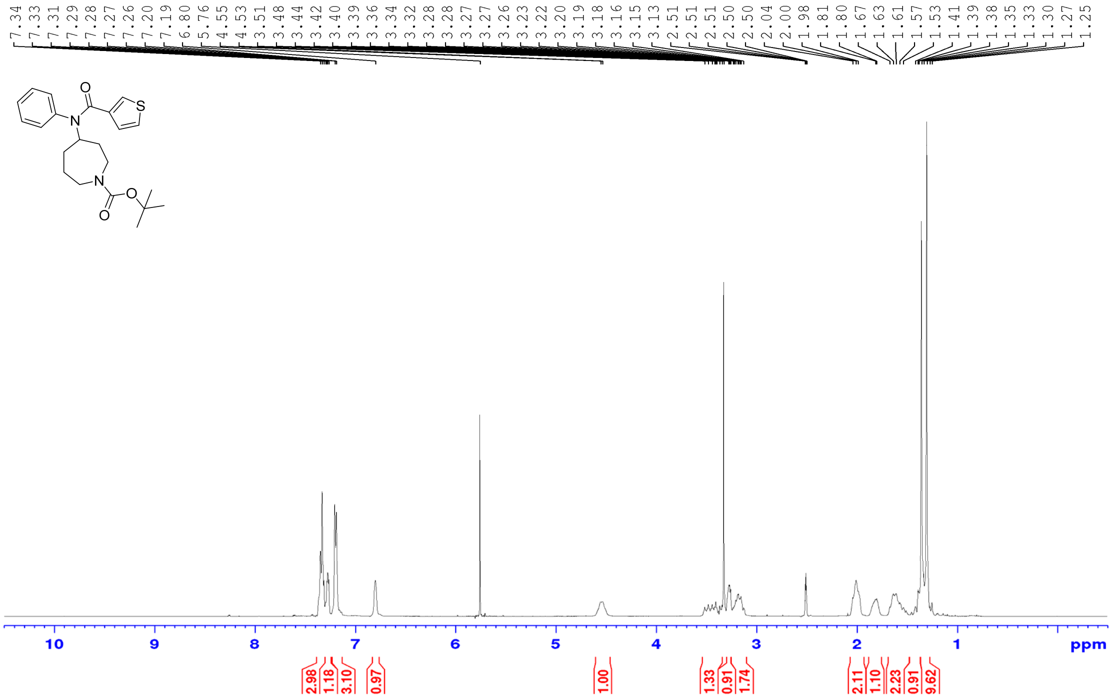

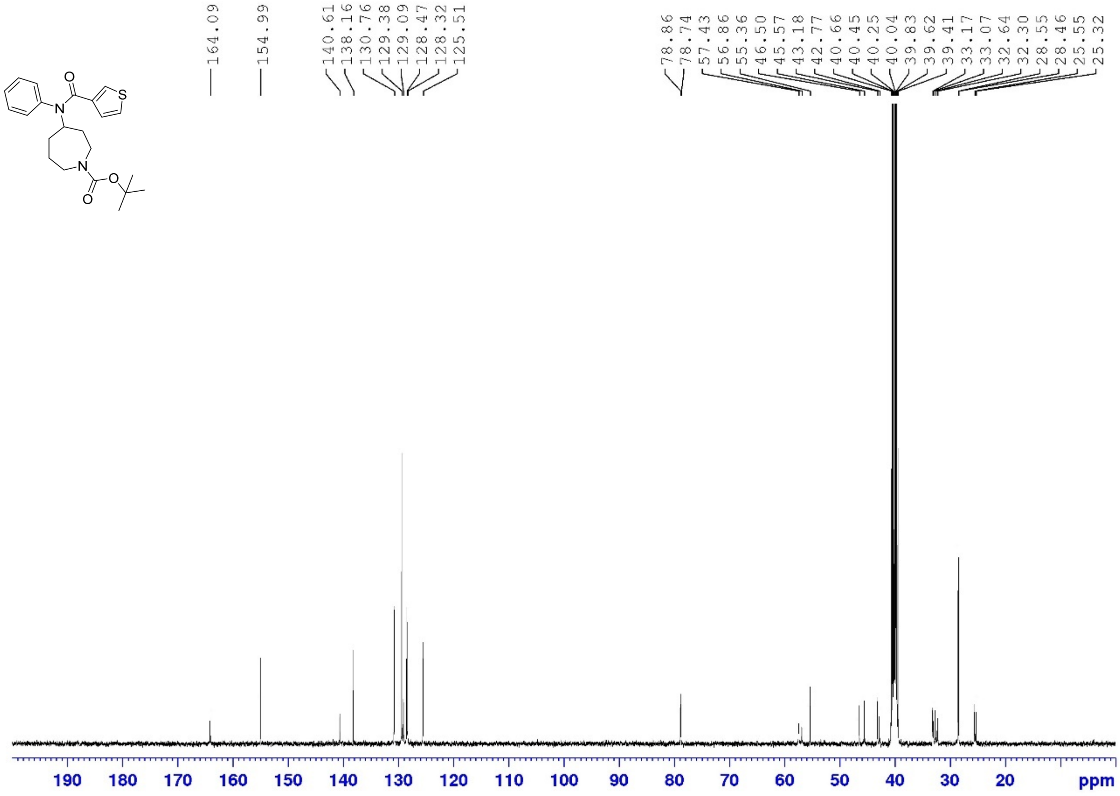

tert-butyl 5-(N-phenylfuran-2-carboxamido)azocane-1-carboxylate **(3a)**

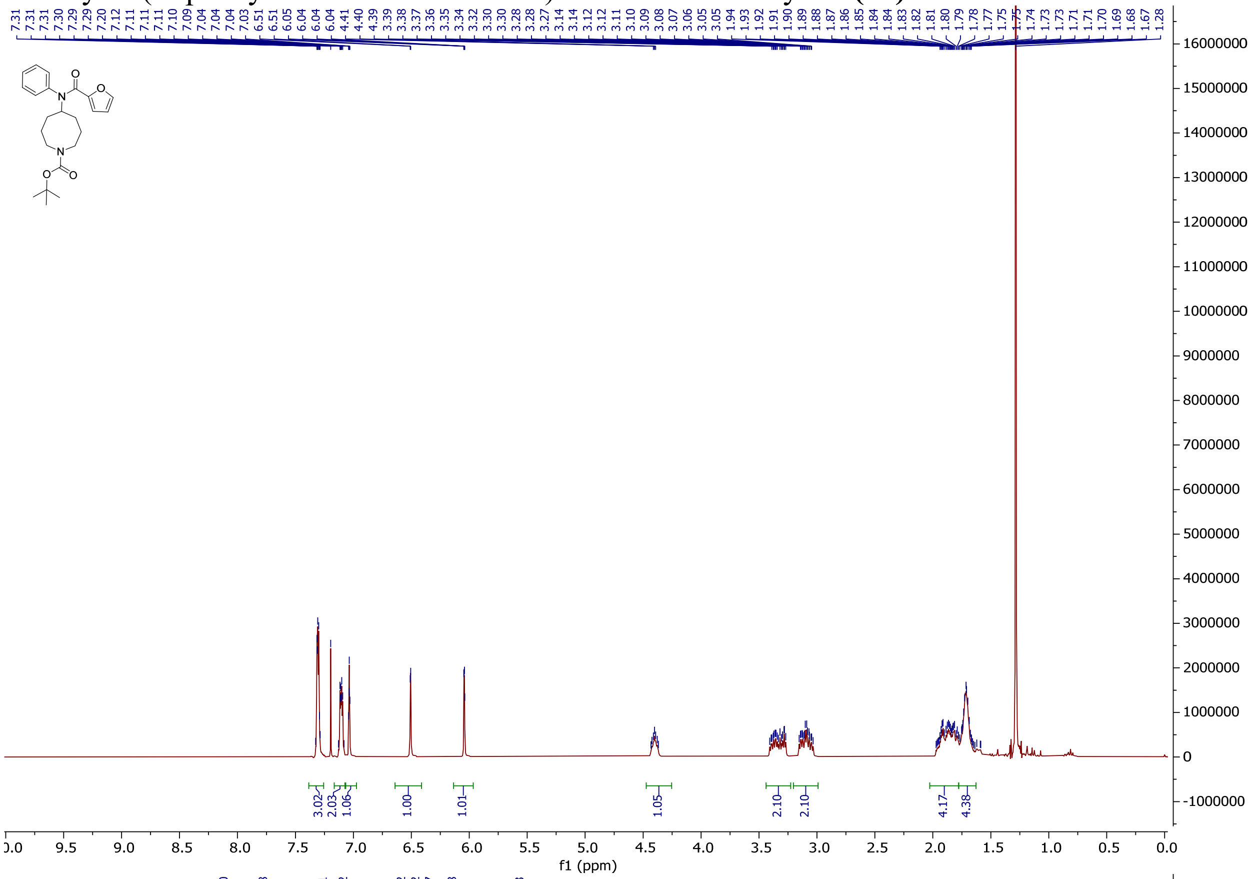

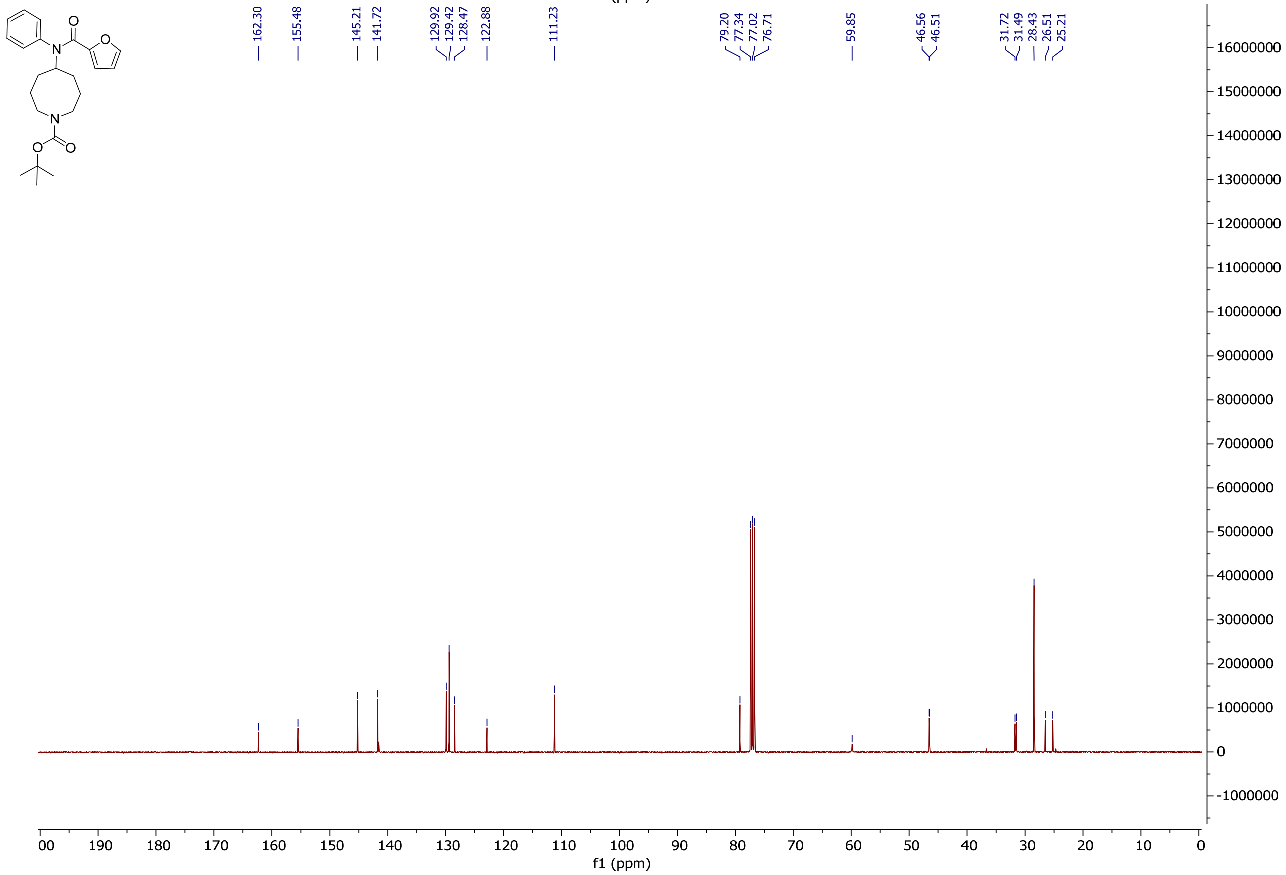

tert-butyl 5-(N-phenylfuran-3-carboxamido)azocane-1-carboxylate **(3b)**

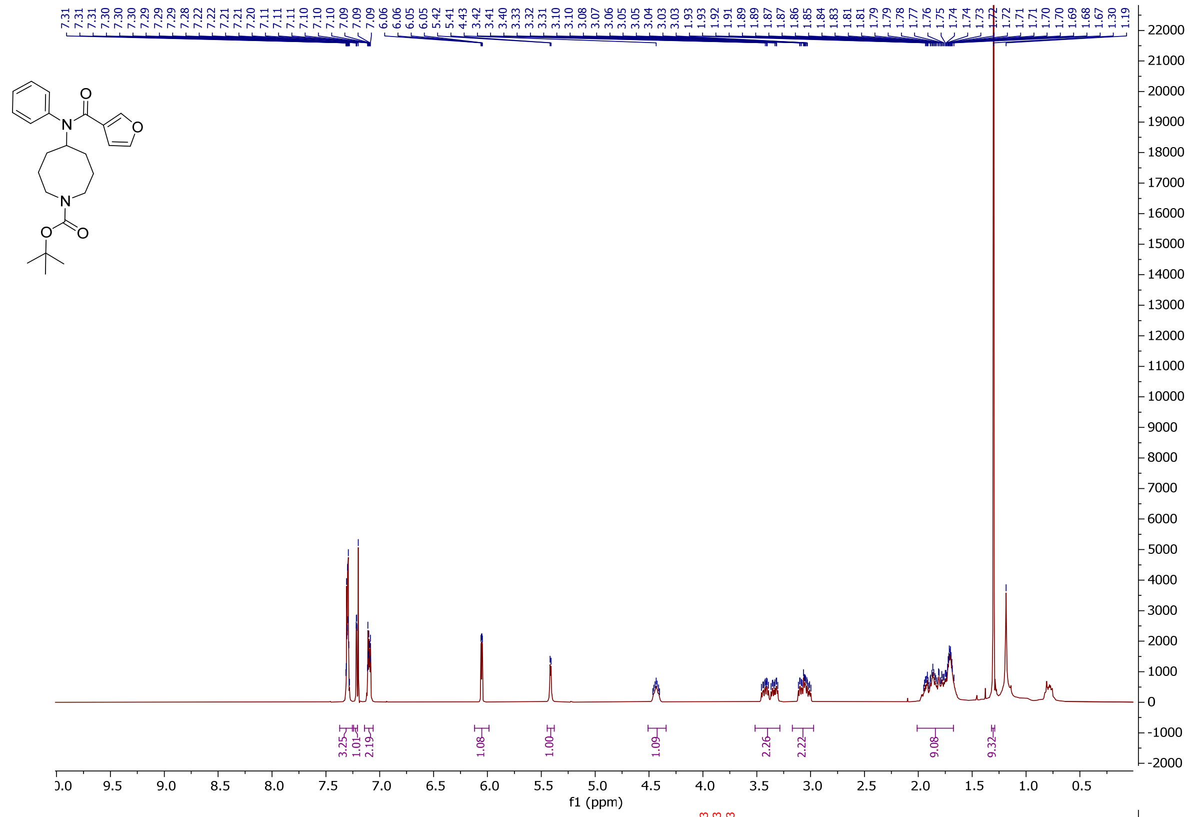

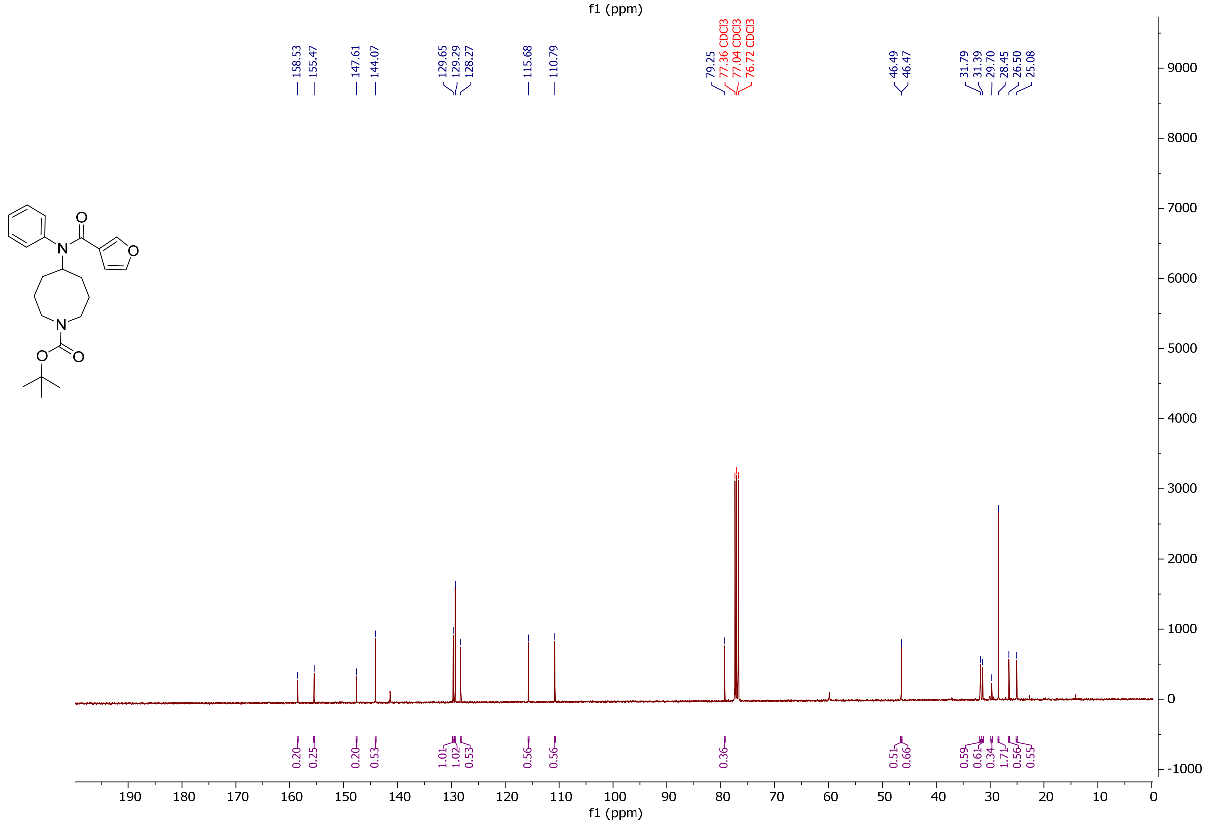

tert-butyl 5-(N-phenylthiophene-2-carboxamido)azocane-1-carboxylate **(3c)**

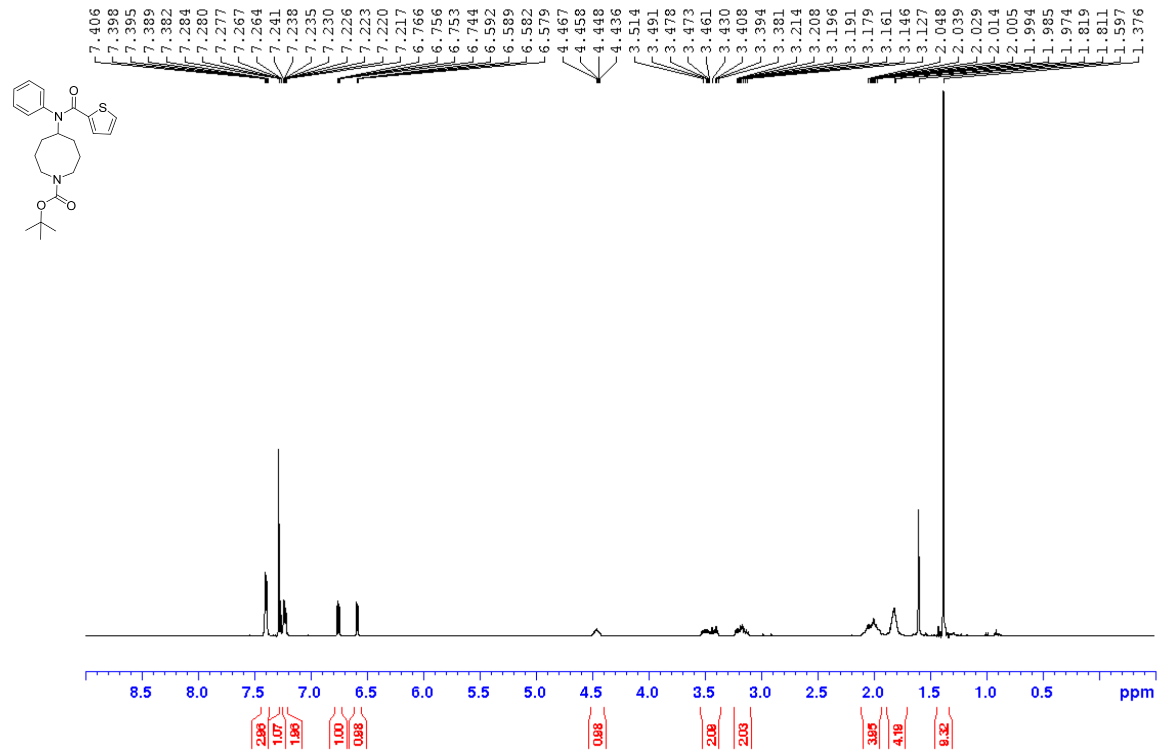

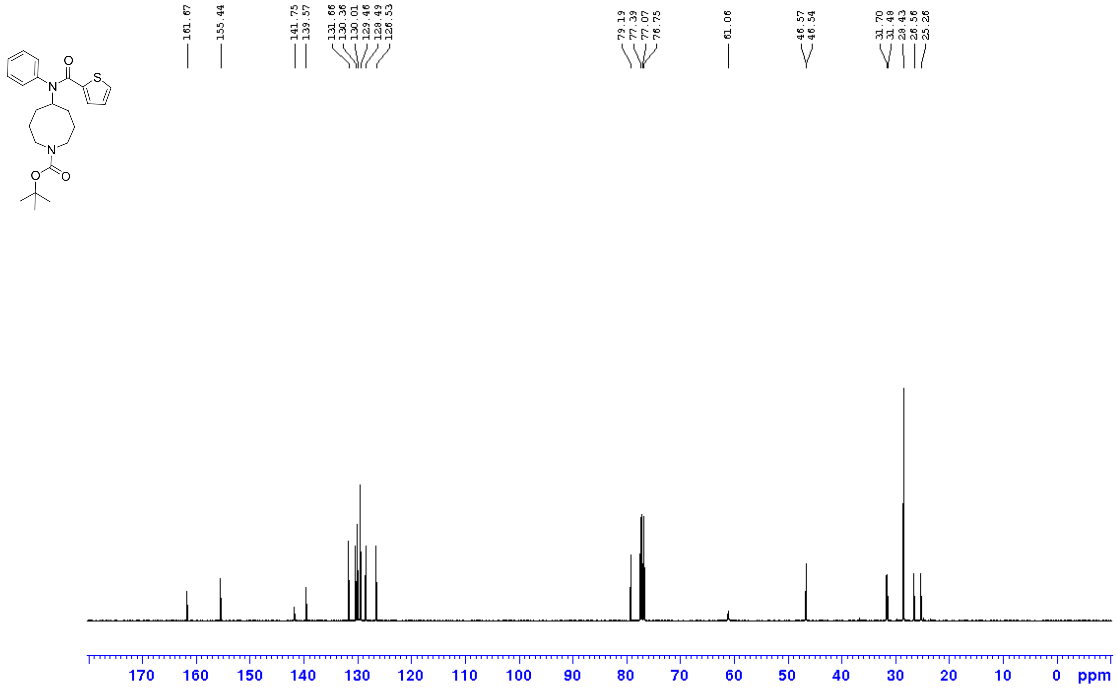

tert-butyl 5-(N-phenyl-1H-pyrrole-2-carboxamido)azocane-1-carboxylate **(3d)**

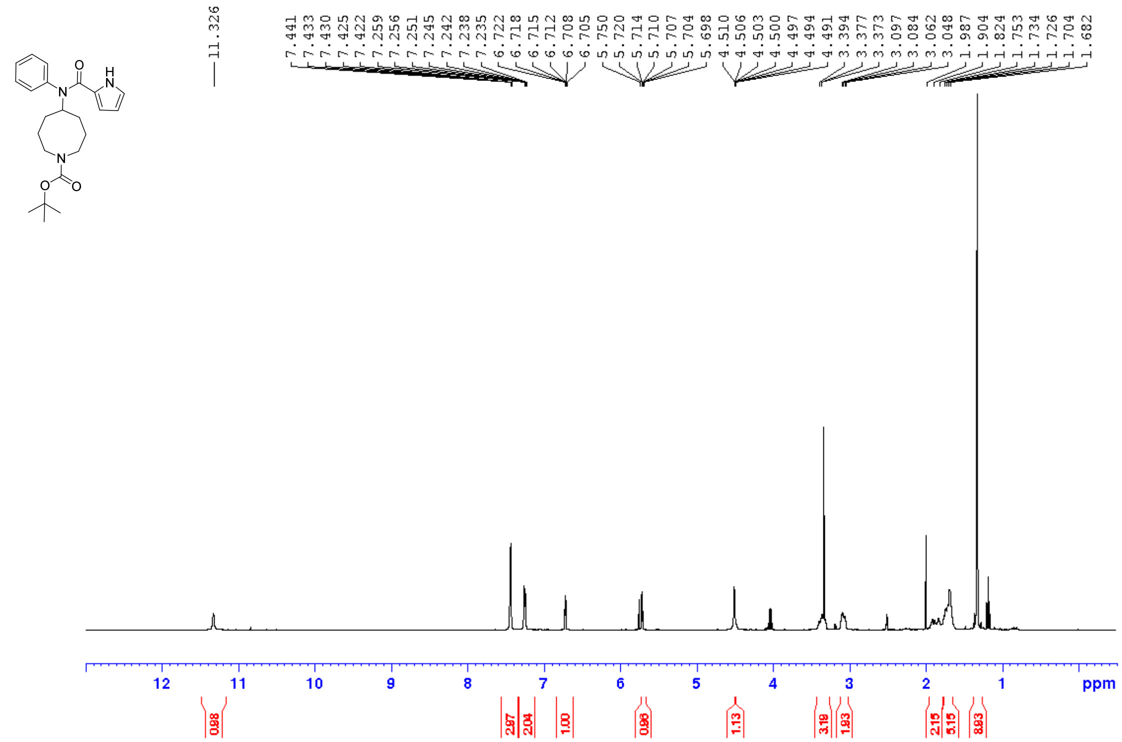

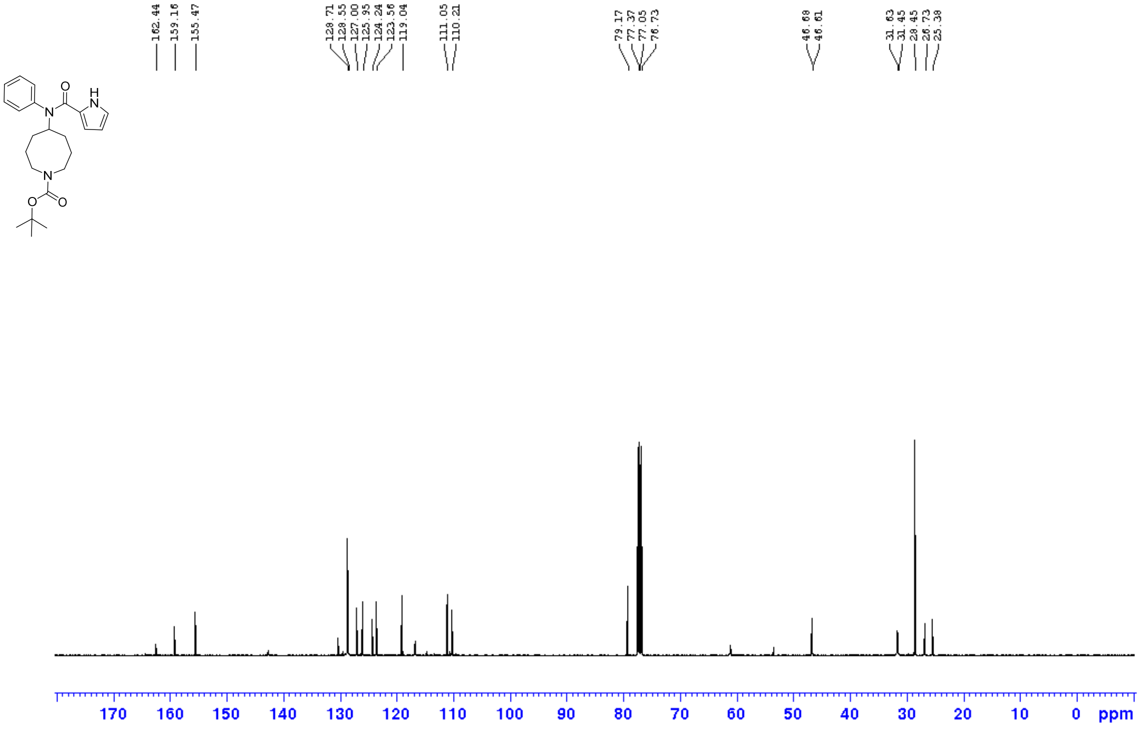

tert-butyl 5-(N-phenylthiophene-3-carboxamido)azocane-1-carboxylate **(3e)**

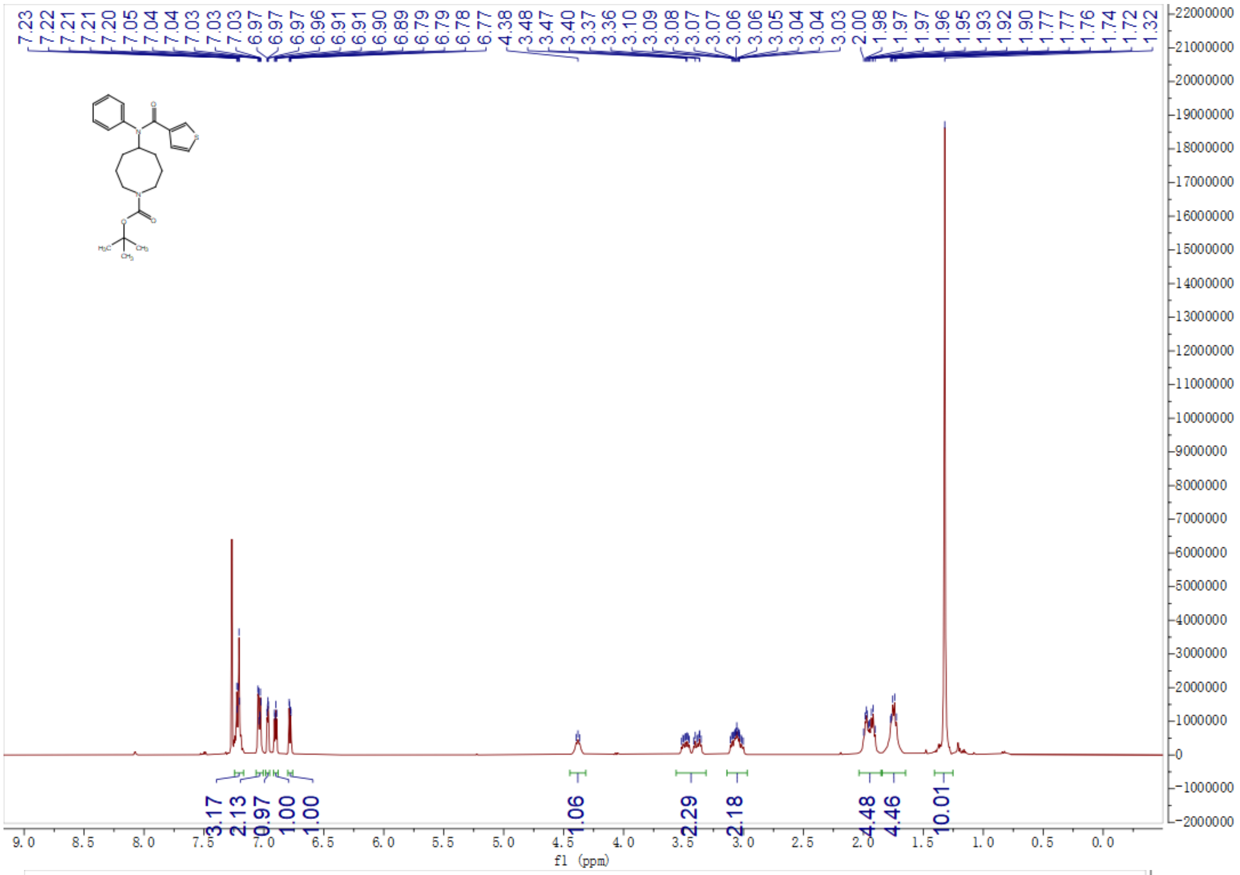

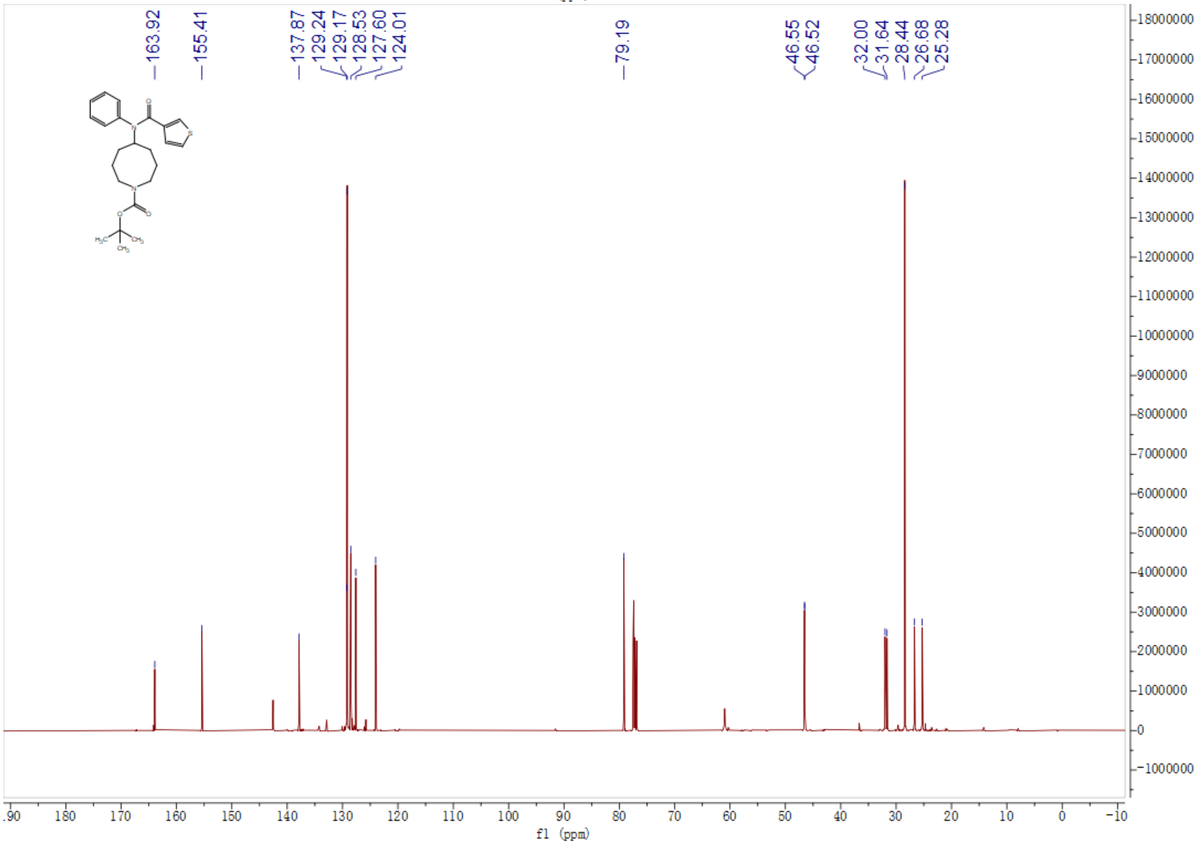

tert-butyl 5-(N-phenyl-1H-pyrrole-3-carboxamido)azocane-1-carboxylate**(3f)**

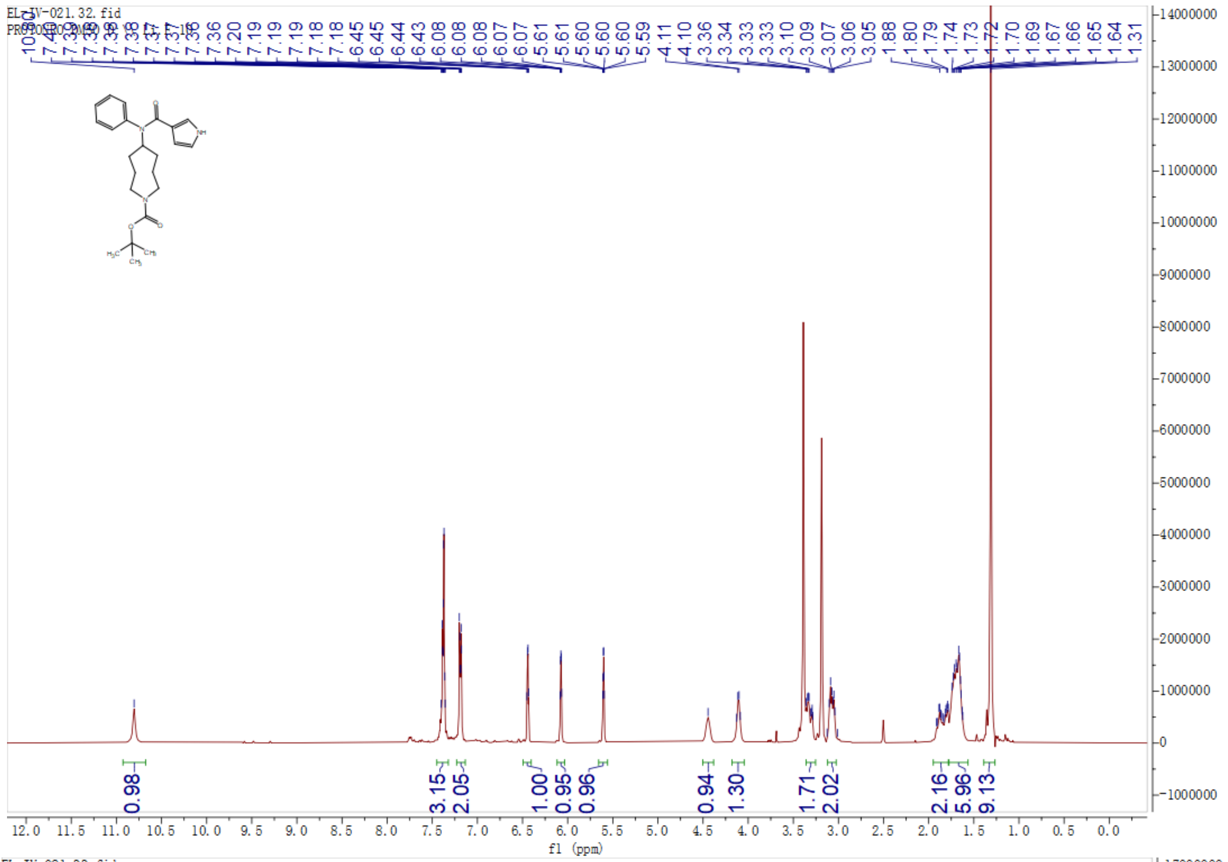

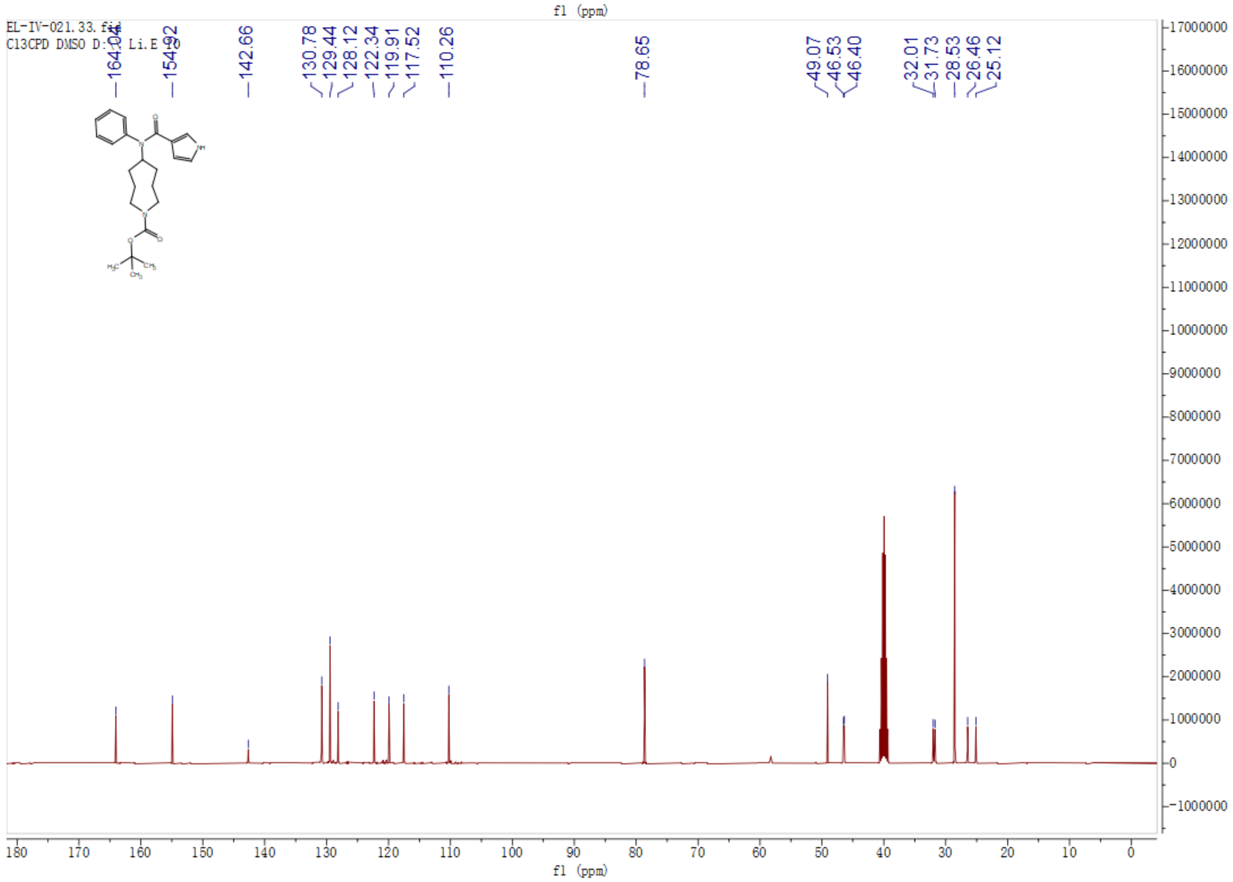

N-(1-allylazepan-4-yl)-N-phenylfuran-2-carboxamide hydrogen chloride **(4)**

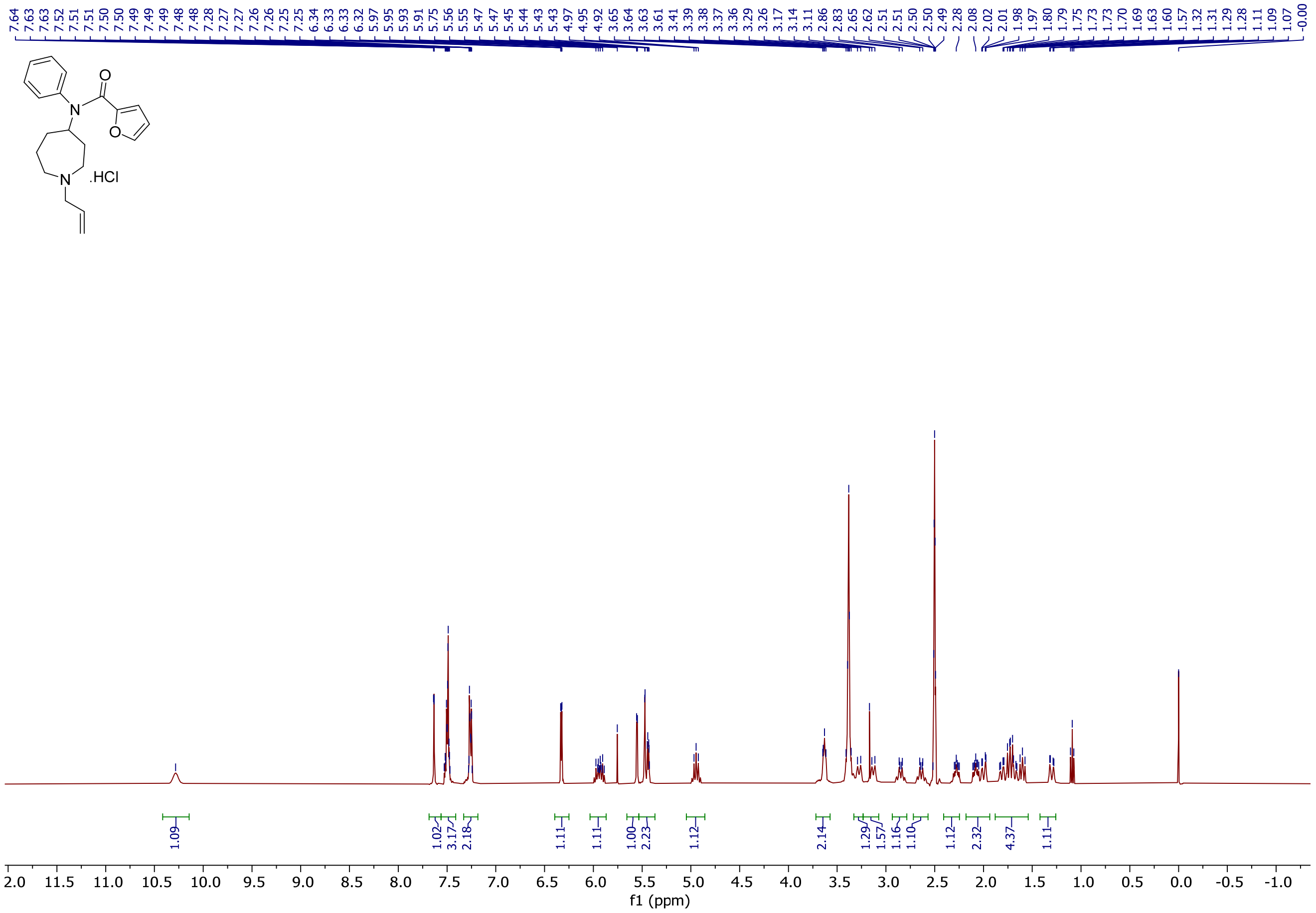

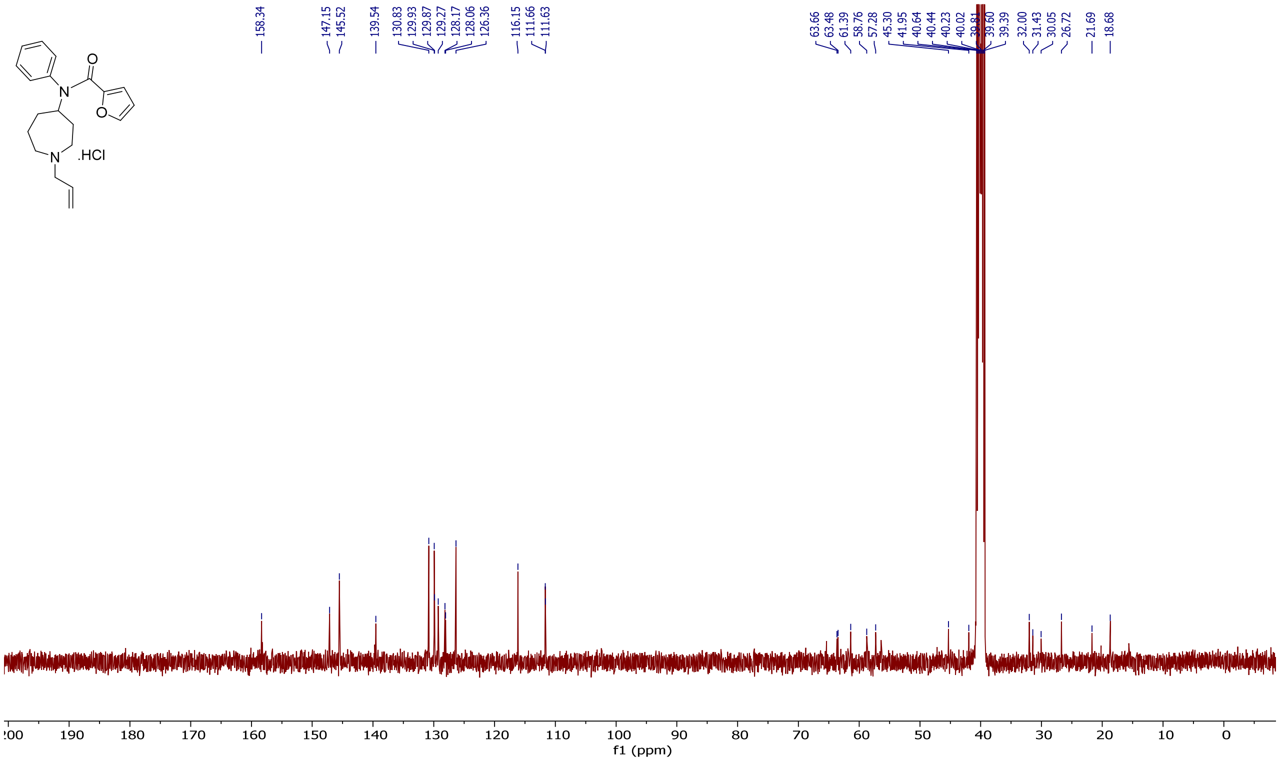

N-(1-(cyclopropylmethyl)azepan-4-yl)-N-phenylfuran-2-carboxamide hydrogen chloride **(5)**

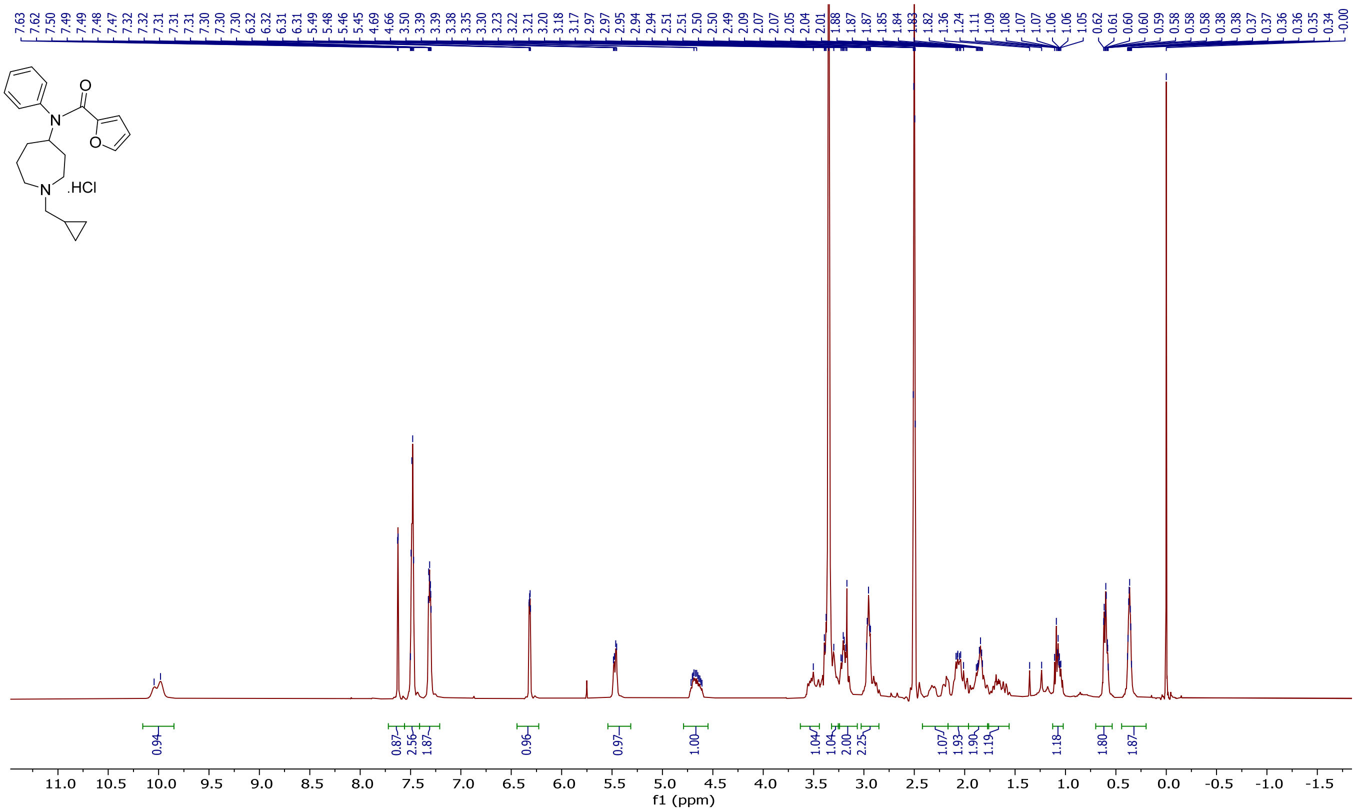

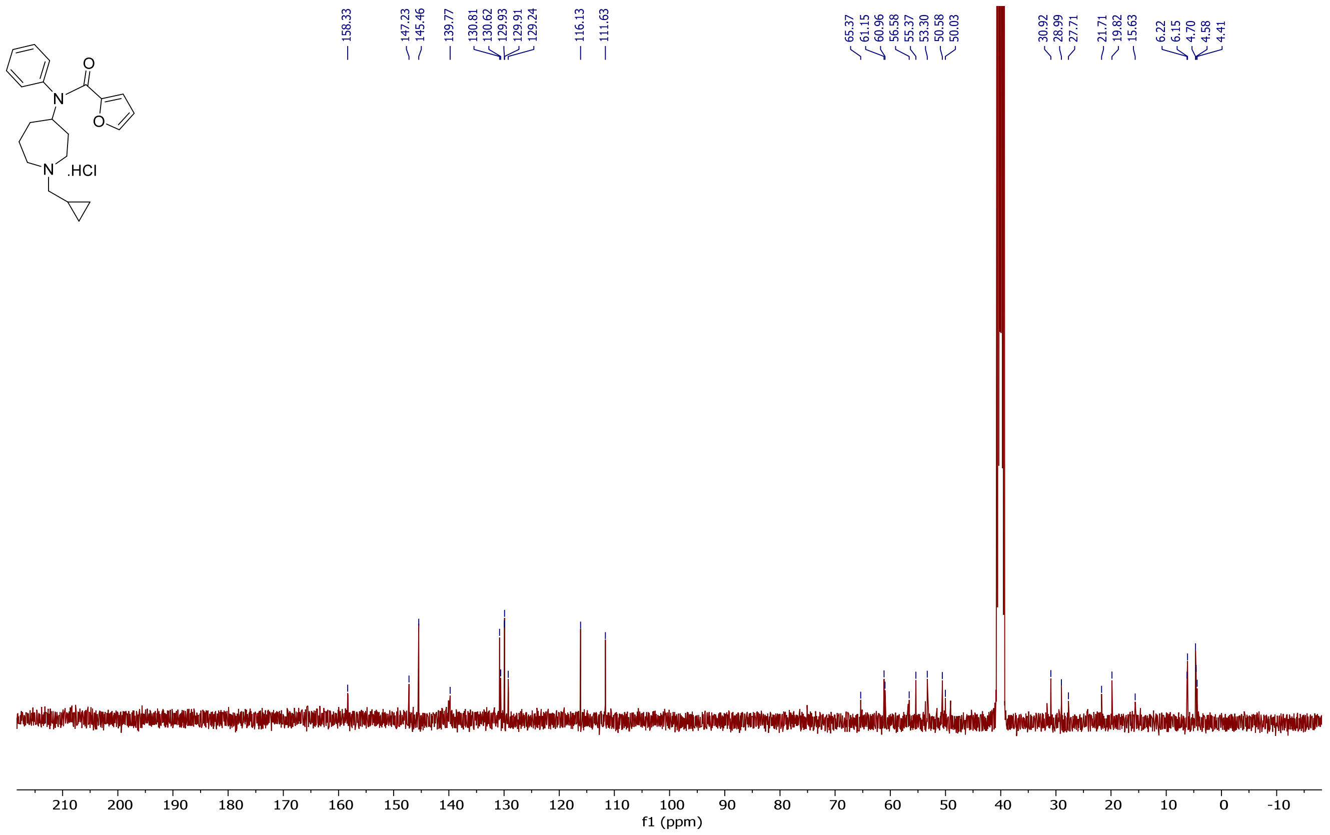

N-(1-(cyclobutylmethyl)azepan-4-yl)-N-phenylfuran-2-carboxamide hydrogen chloride **(6)**

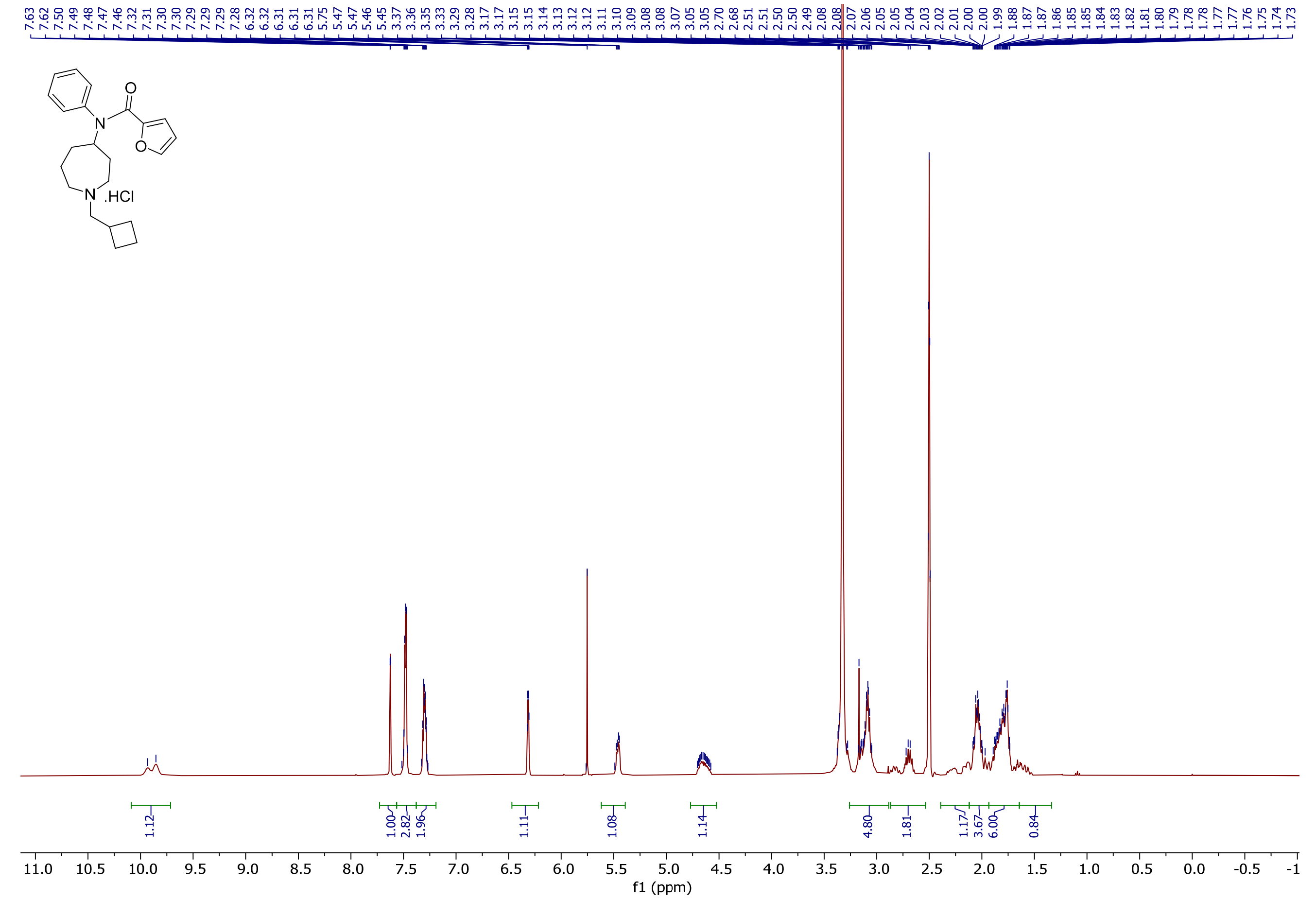

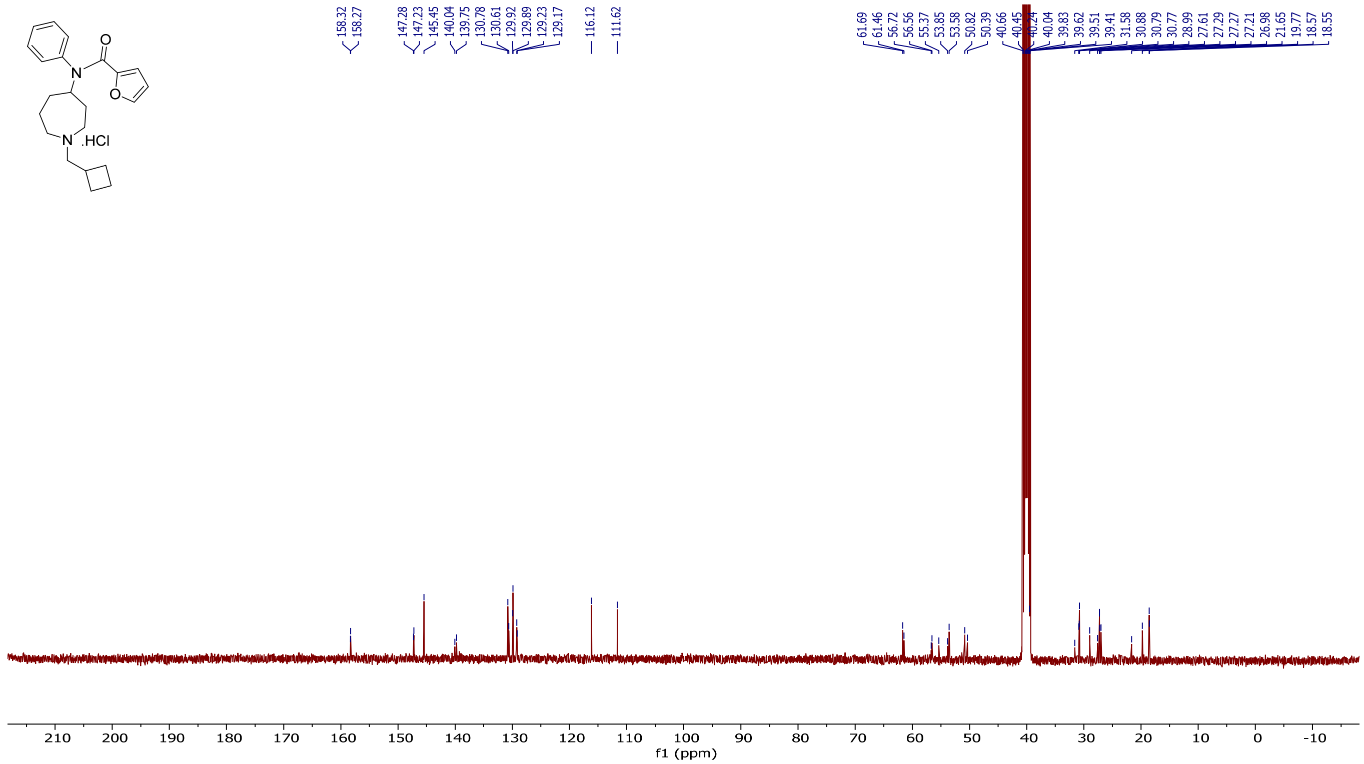

N-(1-(cyclopentylmethyl)azepan-4-yl)-N-phenylfuran-2-carboxamide hydrogen chloride **(7)**

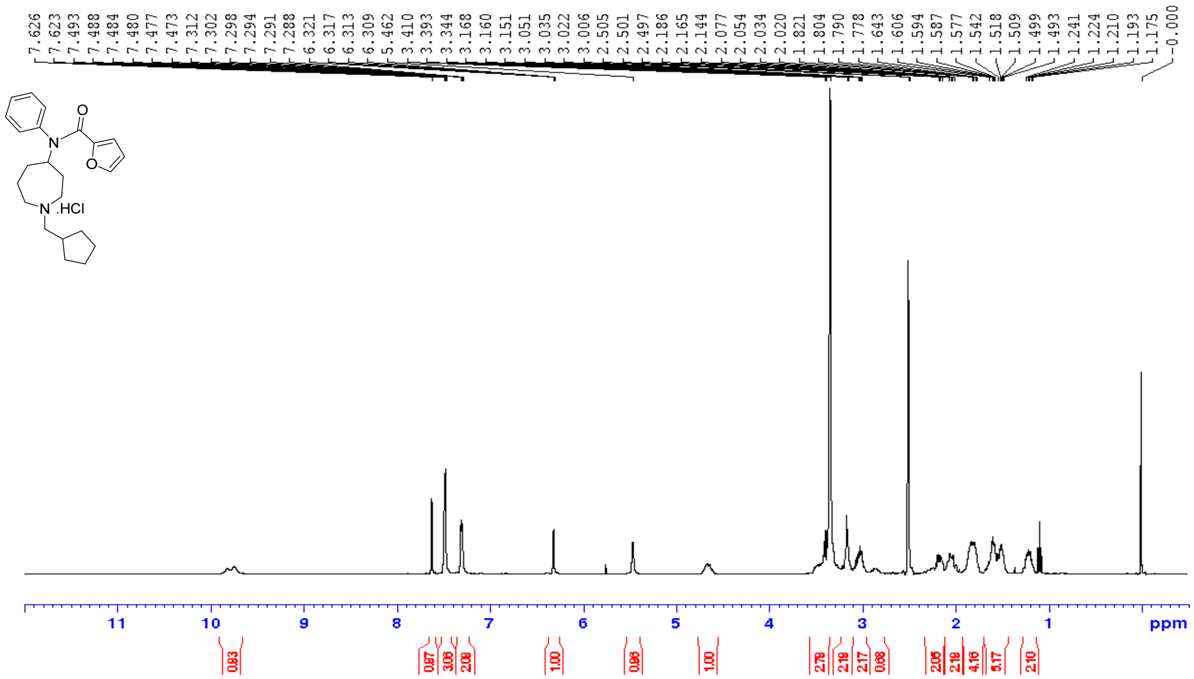

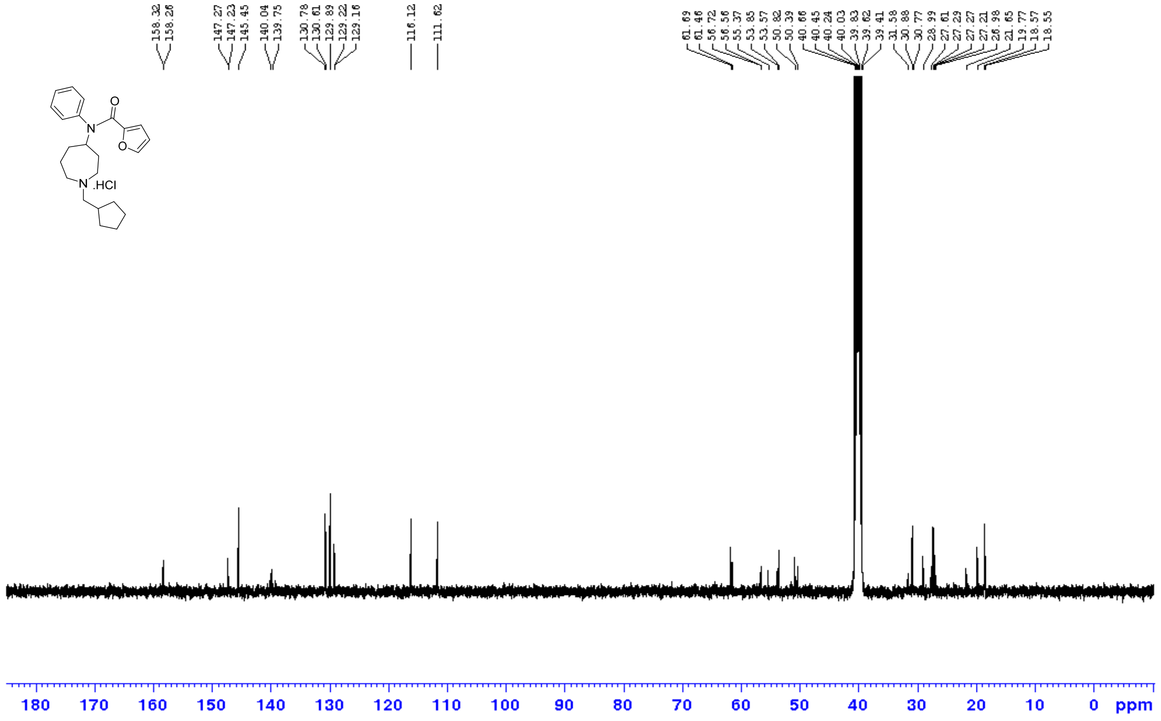

N-(1-(cyclohexylmethyl)azepan-4-yl)-N-phenylfuran-2-carboxamide hydrogen chloride **(8)**

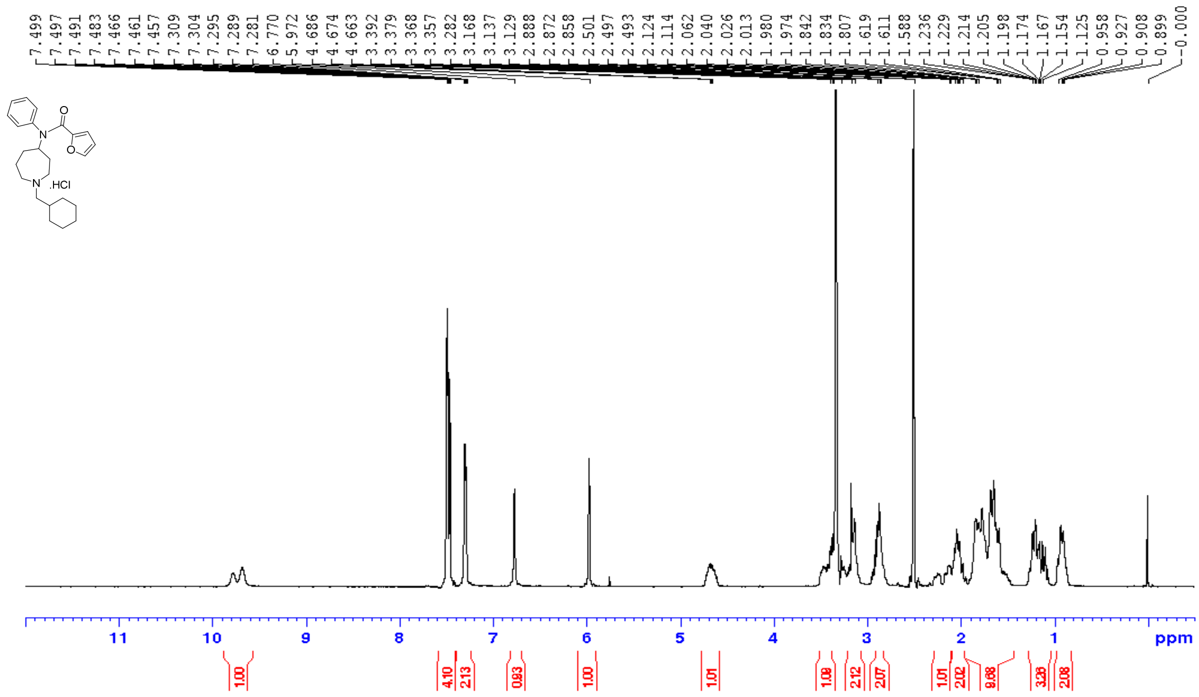

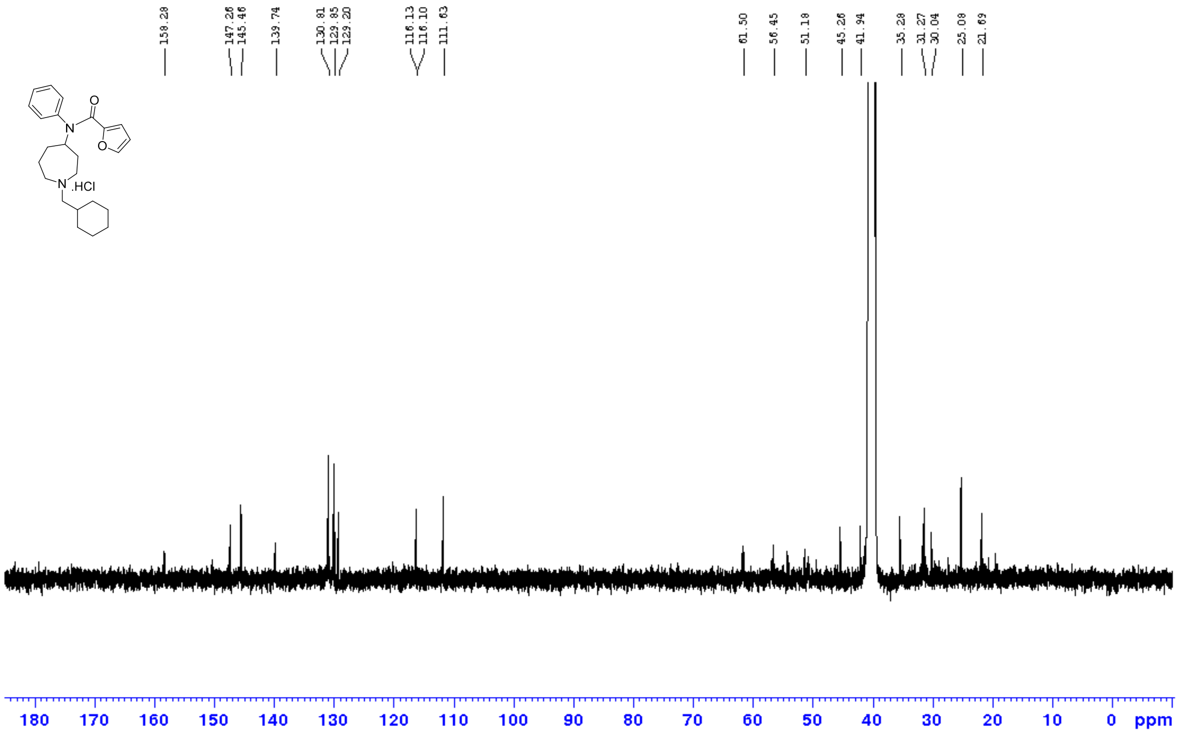

N-(1-benzylazepan-4-yl)-N-phenylfuran-2-carboxamide hydrogen chloride (**9**)

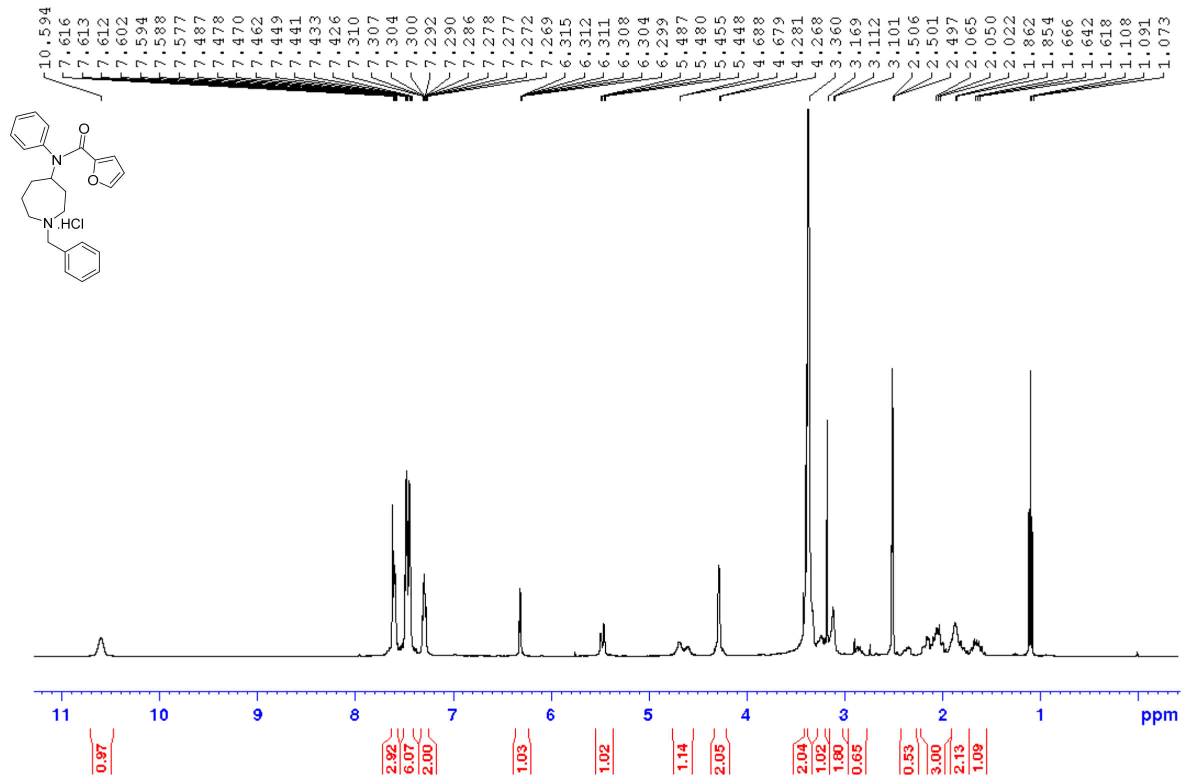

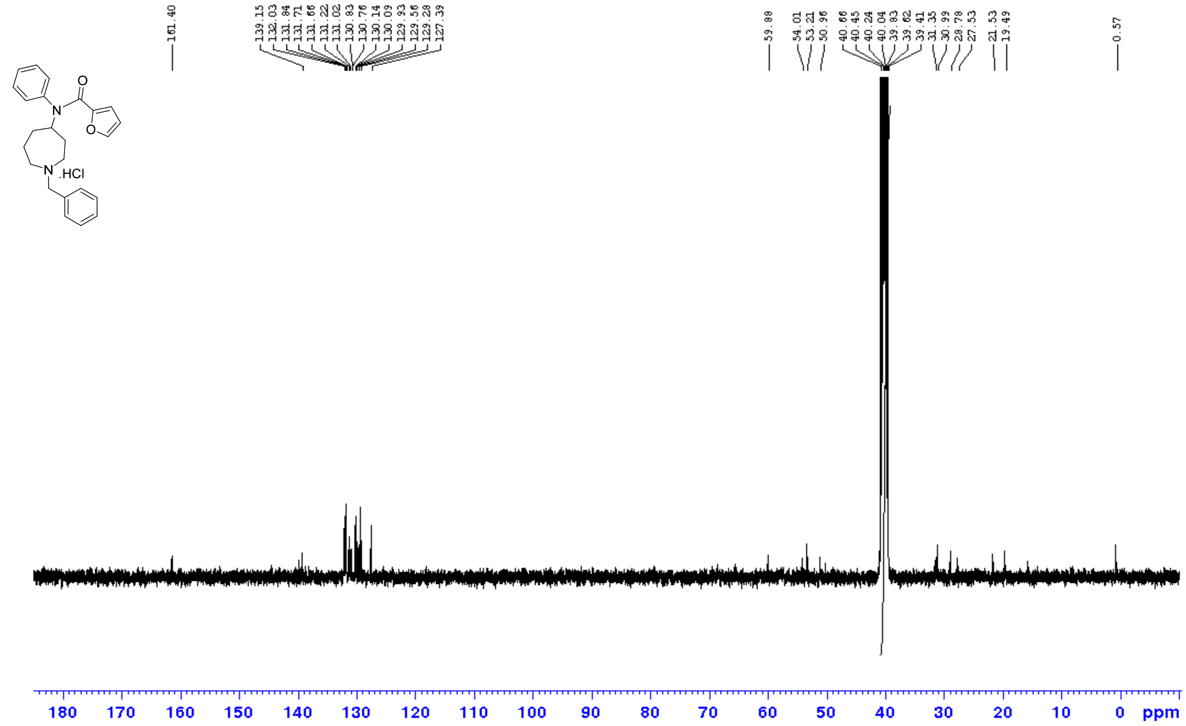

N-(1-phenethylazepan-4-yl)-N-phenylfuran-2-carboxamide hydrogen chloride (**10**)

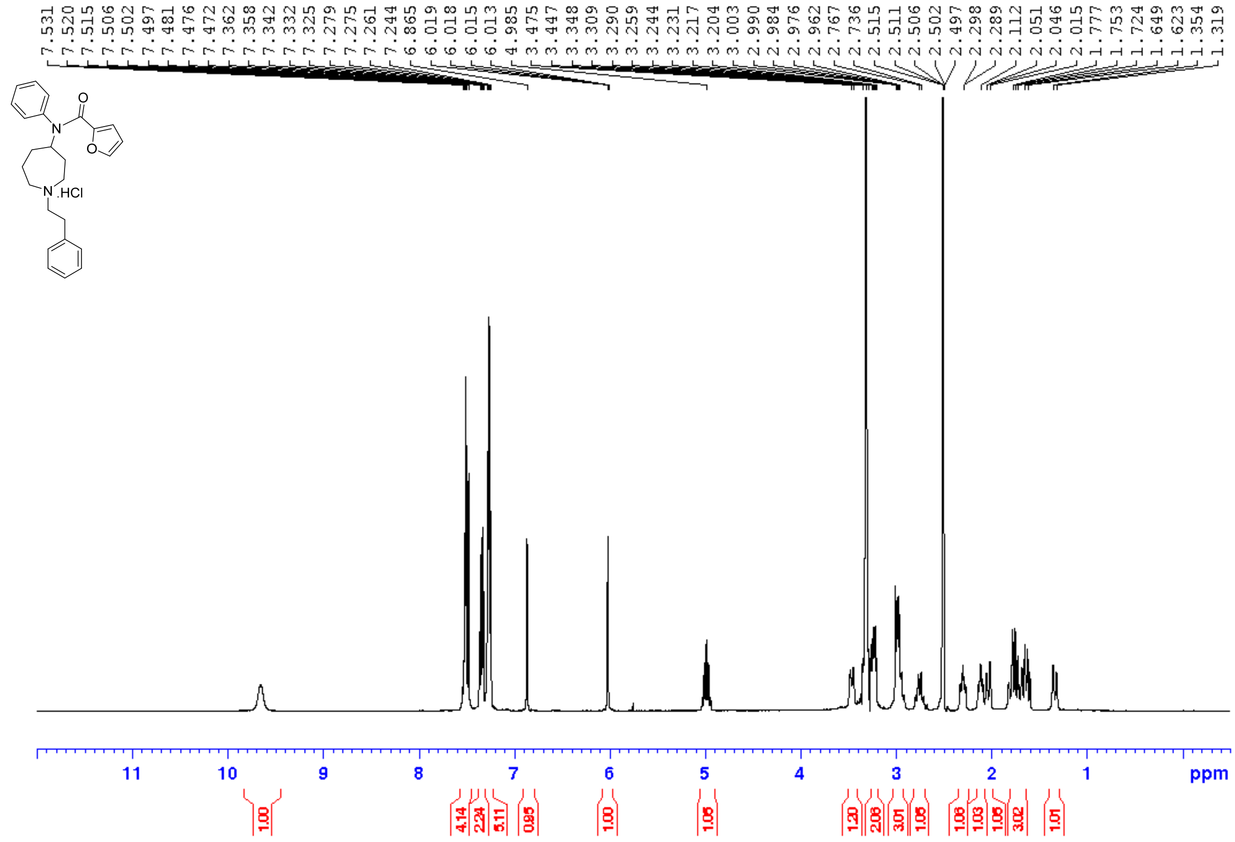

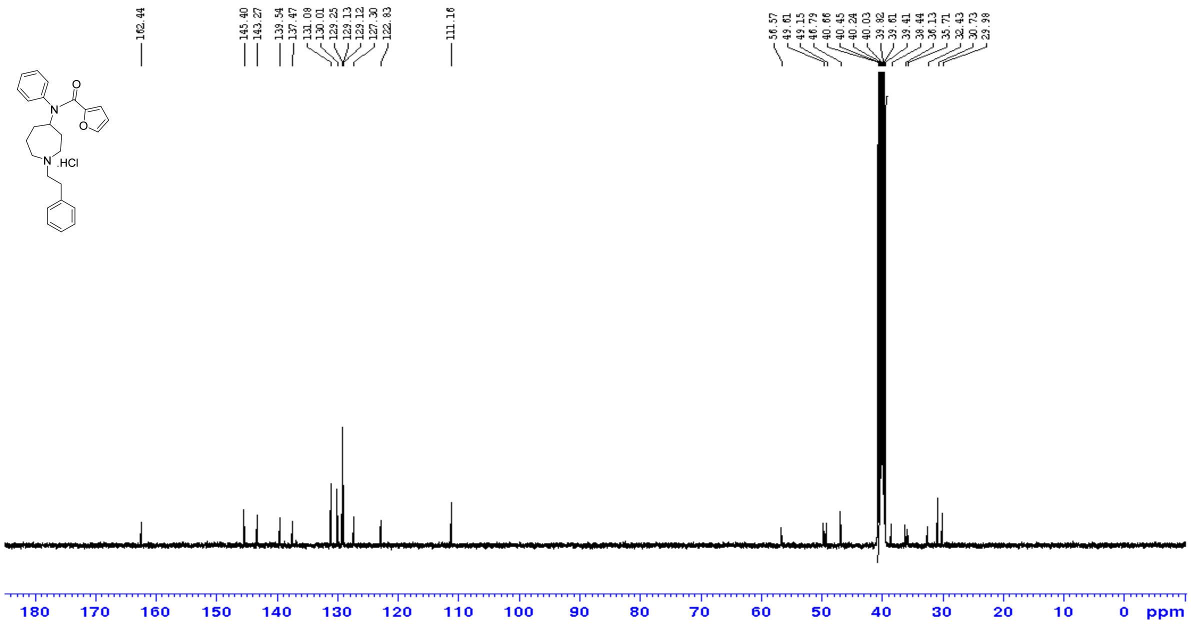

N-(1-allylazepan-4-yl)-N-phenylthiophene-2-carboxamide hydrogen chloride **(11)**

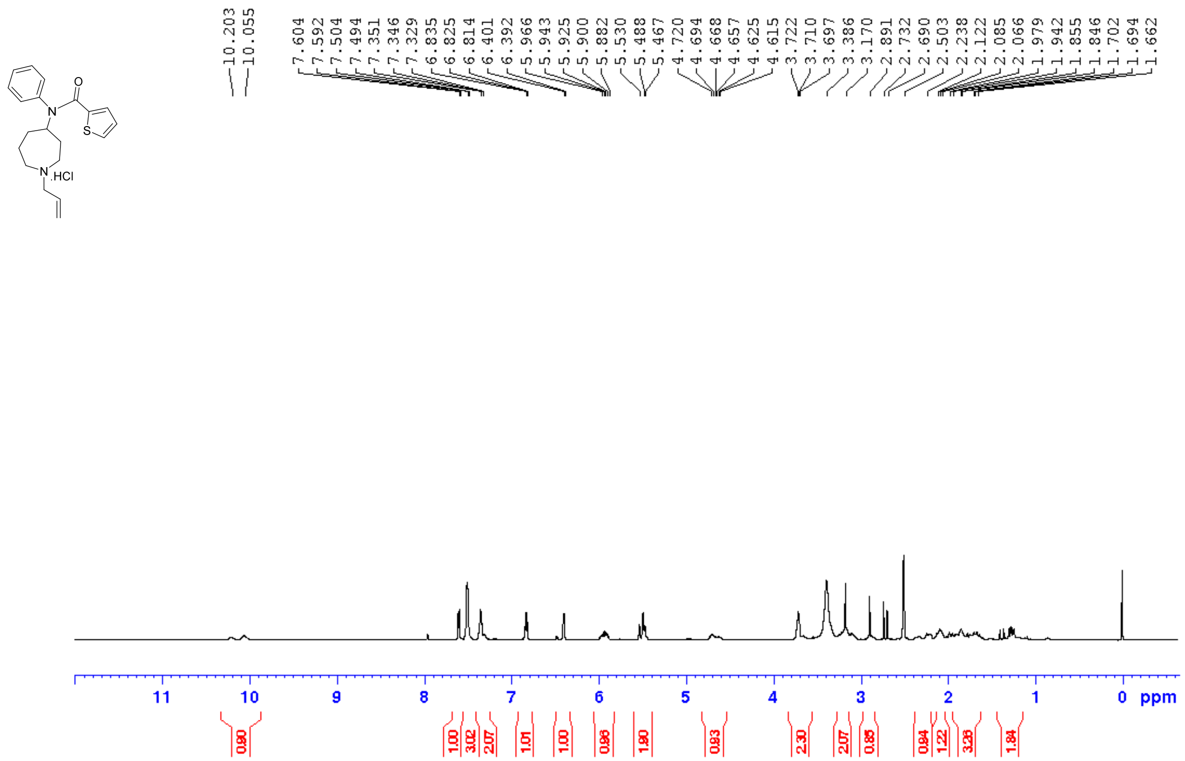

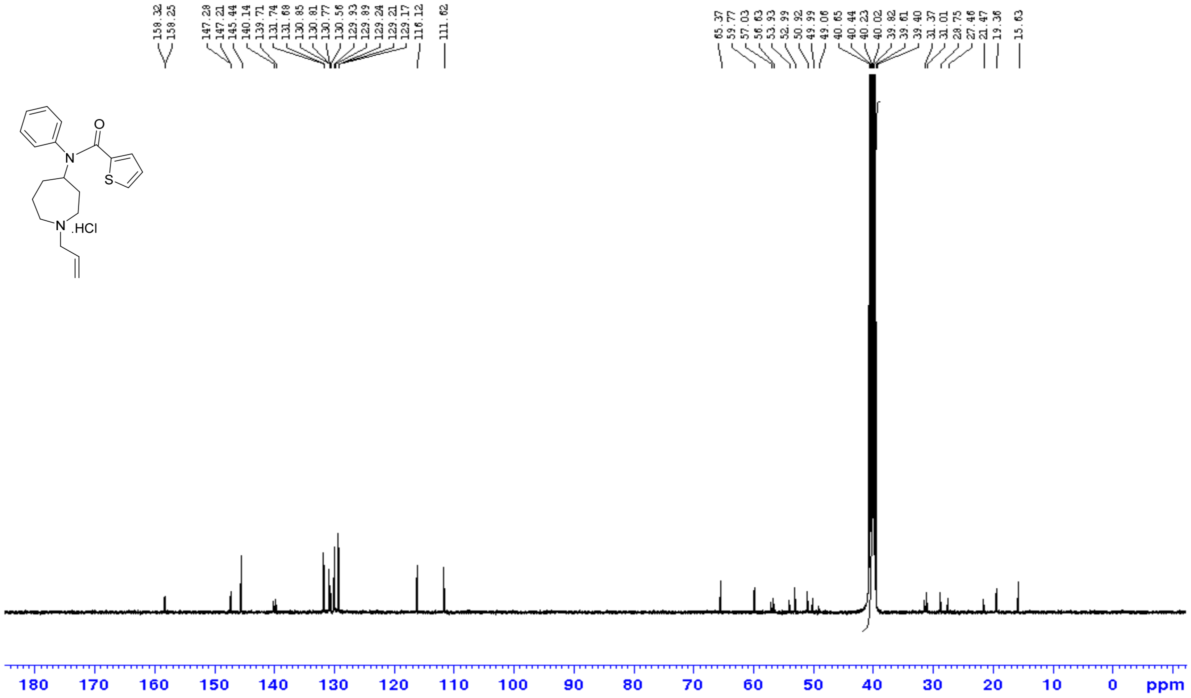

N-(1-(cyclopropylmethyl)azepan-4-yl)-N-phenylthiophene-2-carboxamide hydrogen chloride **(12)**

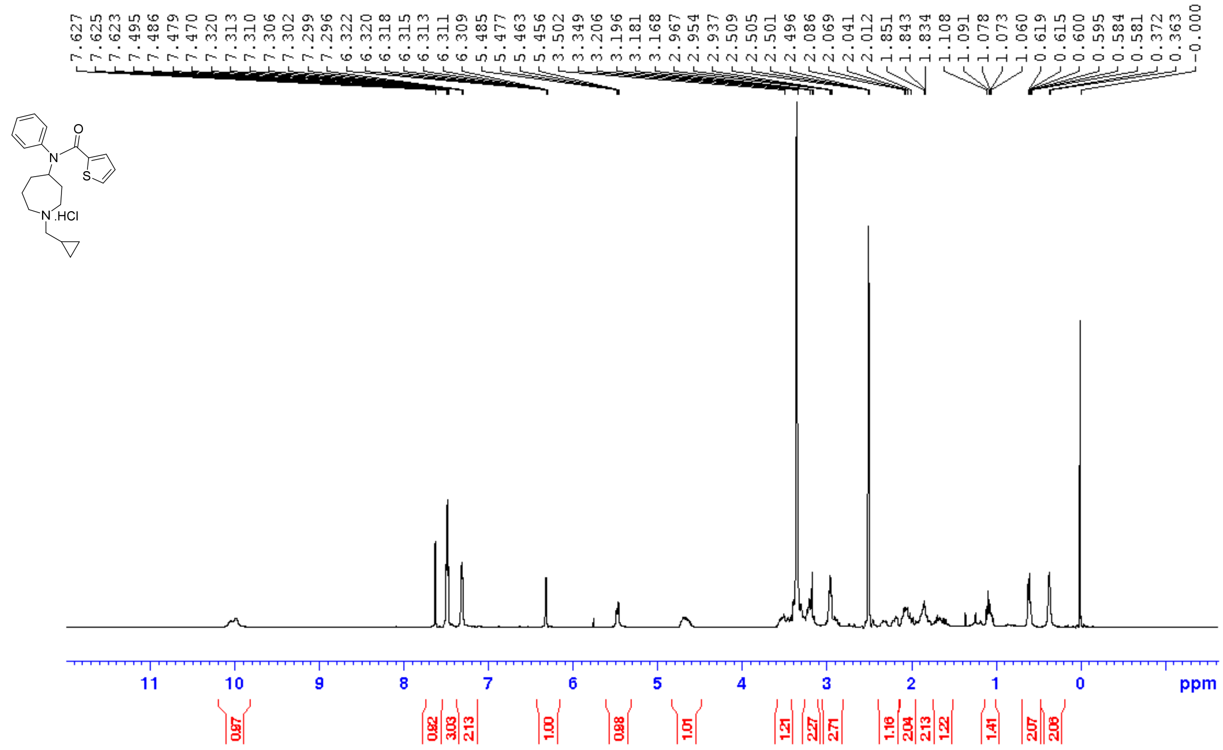

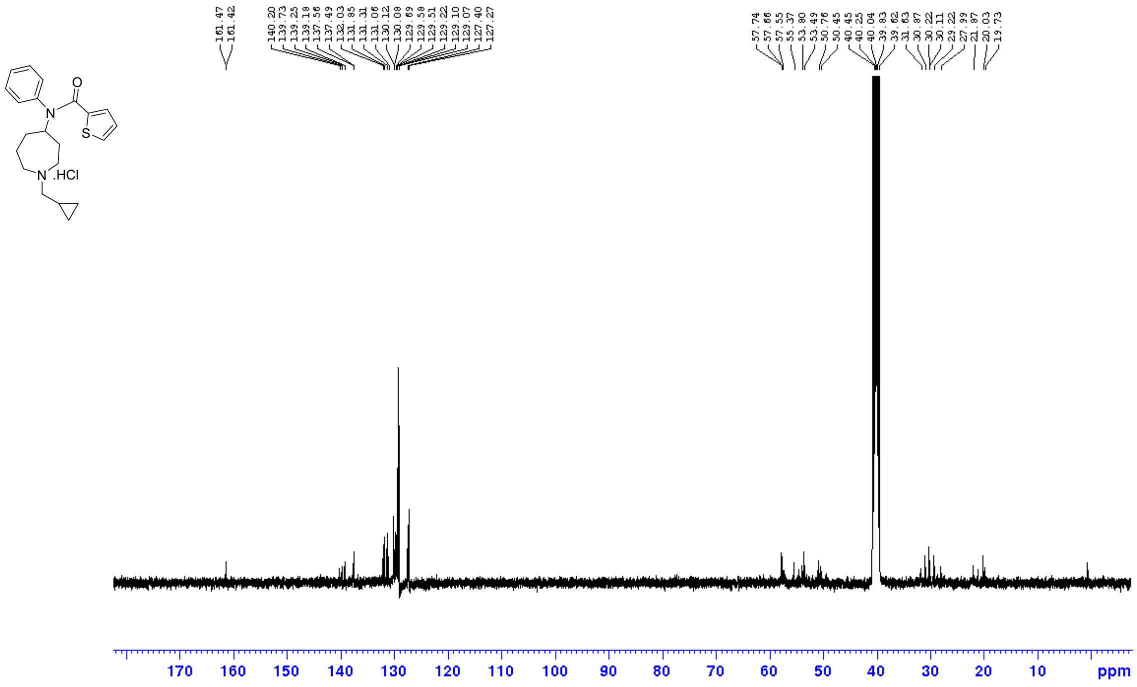

N-(1-(cyclobutylmethyl)azepan-4-yl)-N-phenylthiophene-2-carboxamide hydrogen chloride **(13)**

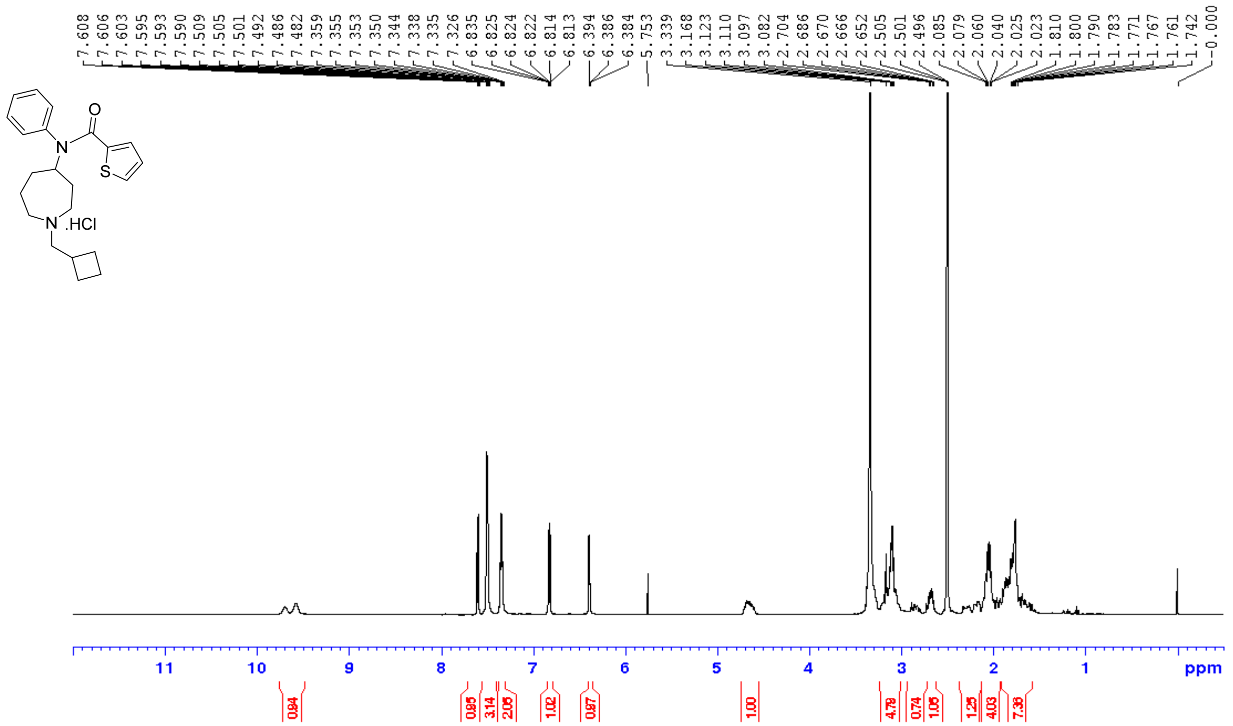

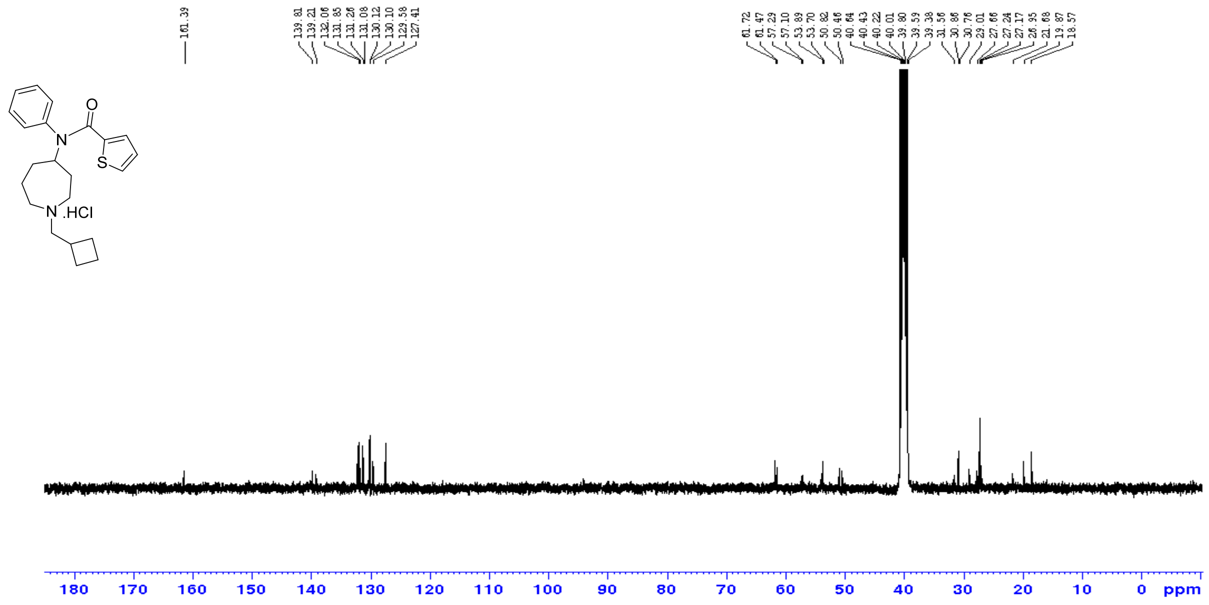

N-(1-(cyclopentylmethyl)azepan-4-yl)-N-phenylthiophene-2-carboxamide hydrogen chloride **(14)**

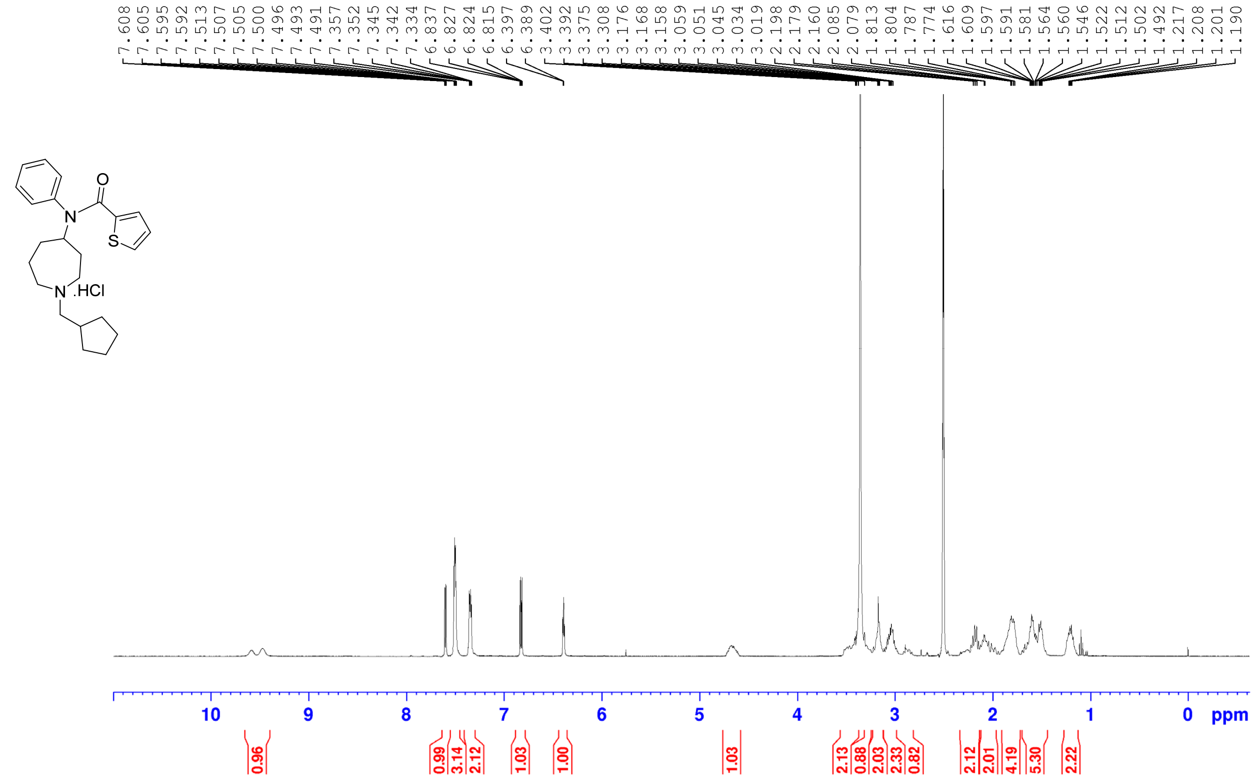

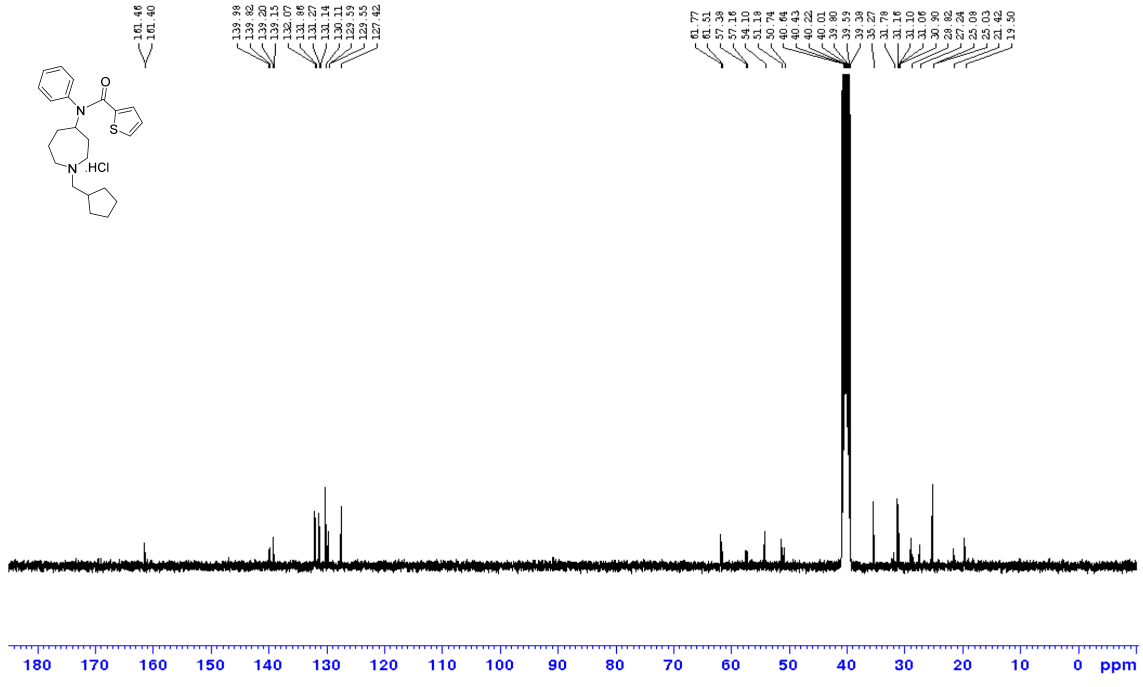

N-(1-(cyclohexylmethyl)azepan-4-yl)-N-phenylthiophene-2-carboxamide hydrogen chloride **(15)**

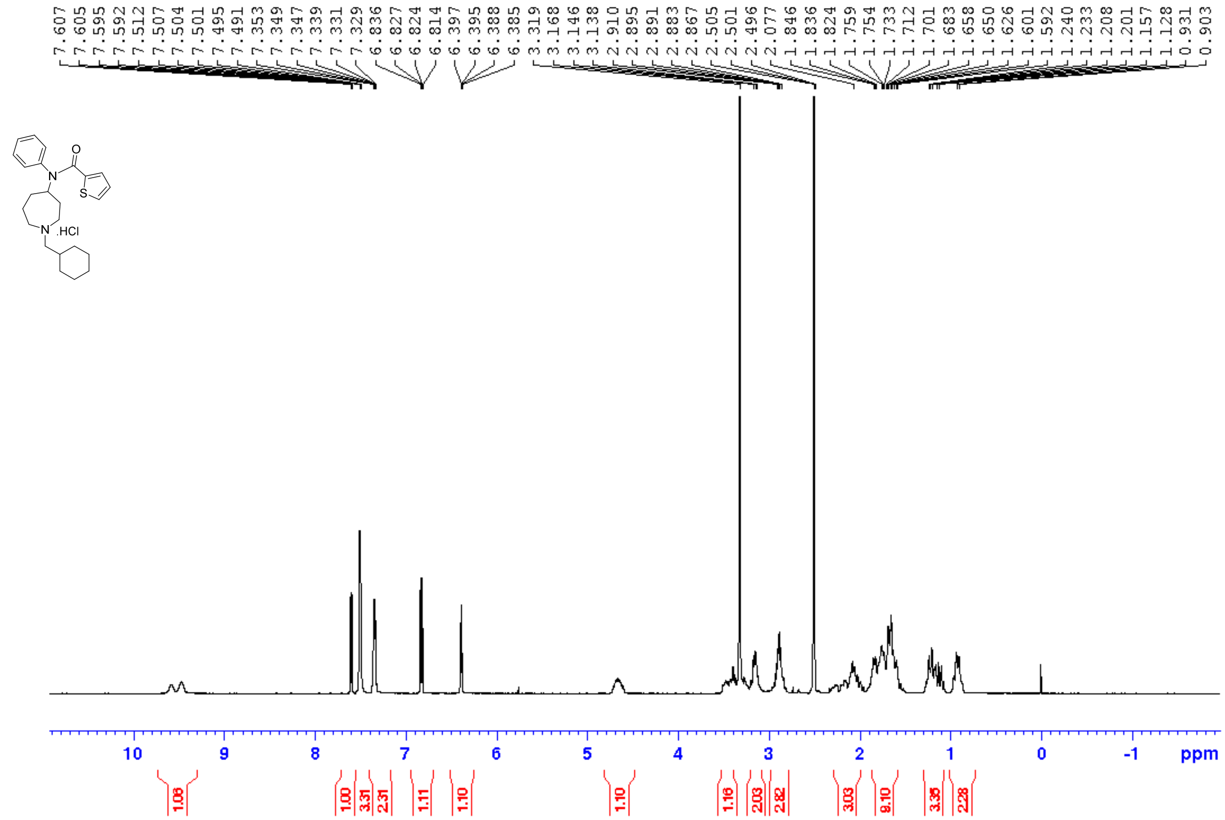

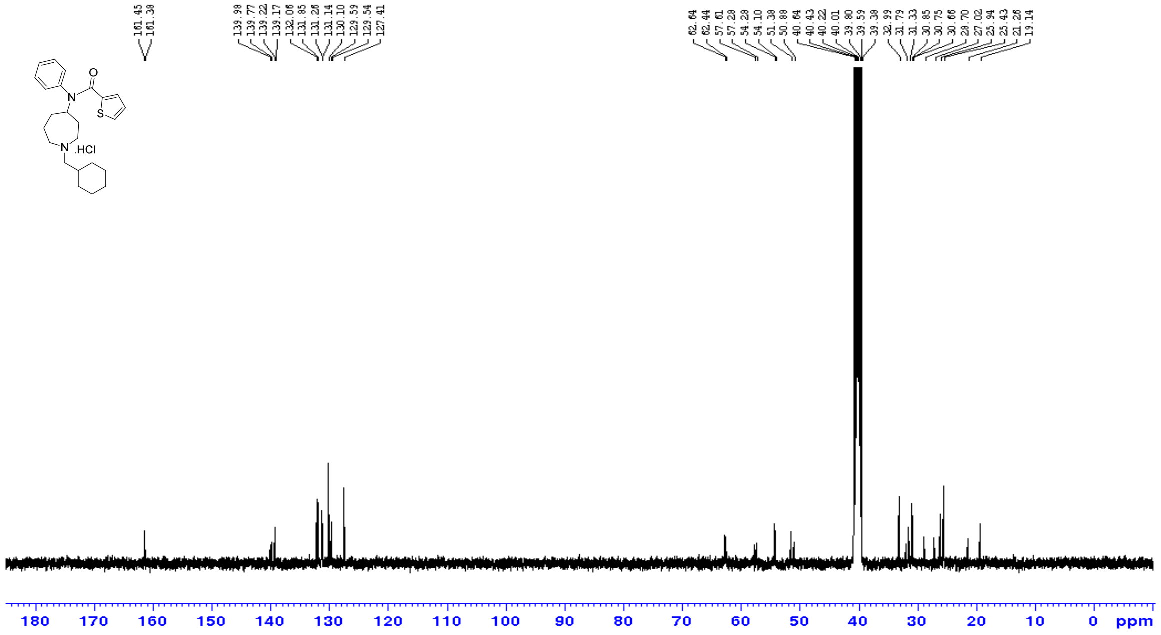

N-(1-benzylazepan-4-yl)-N-phenylthiophene-2-carboxamide hydrogen chloride **(16)**

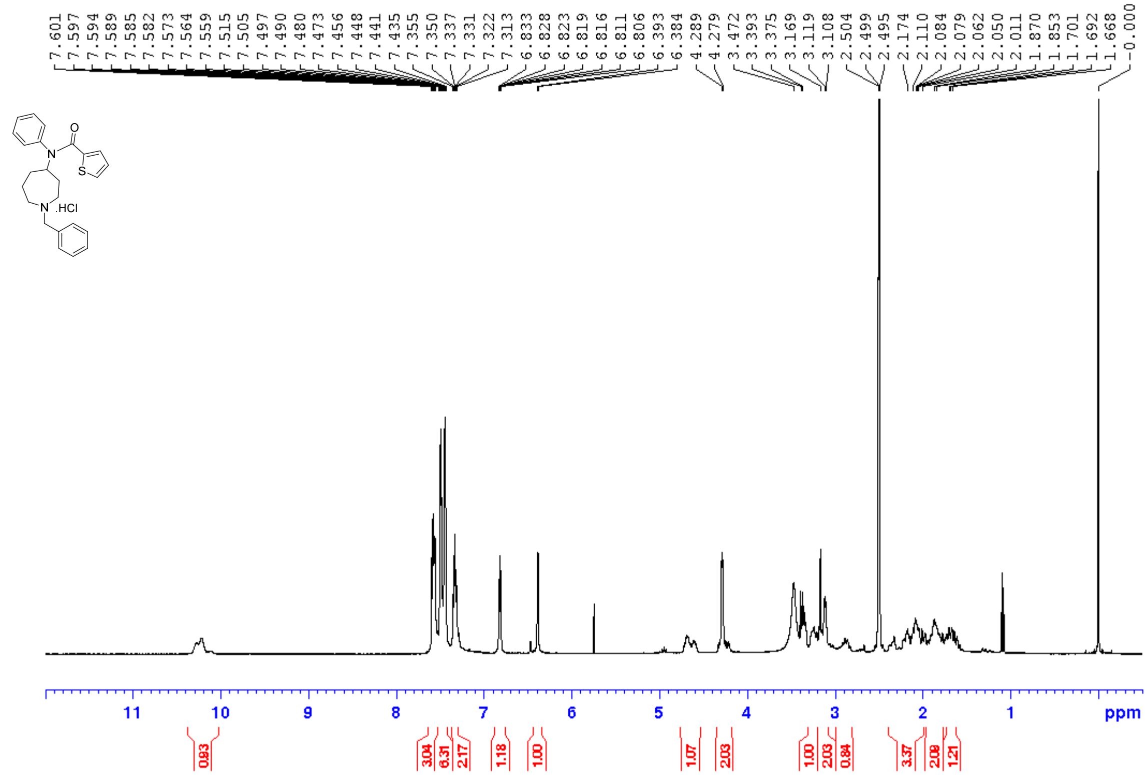

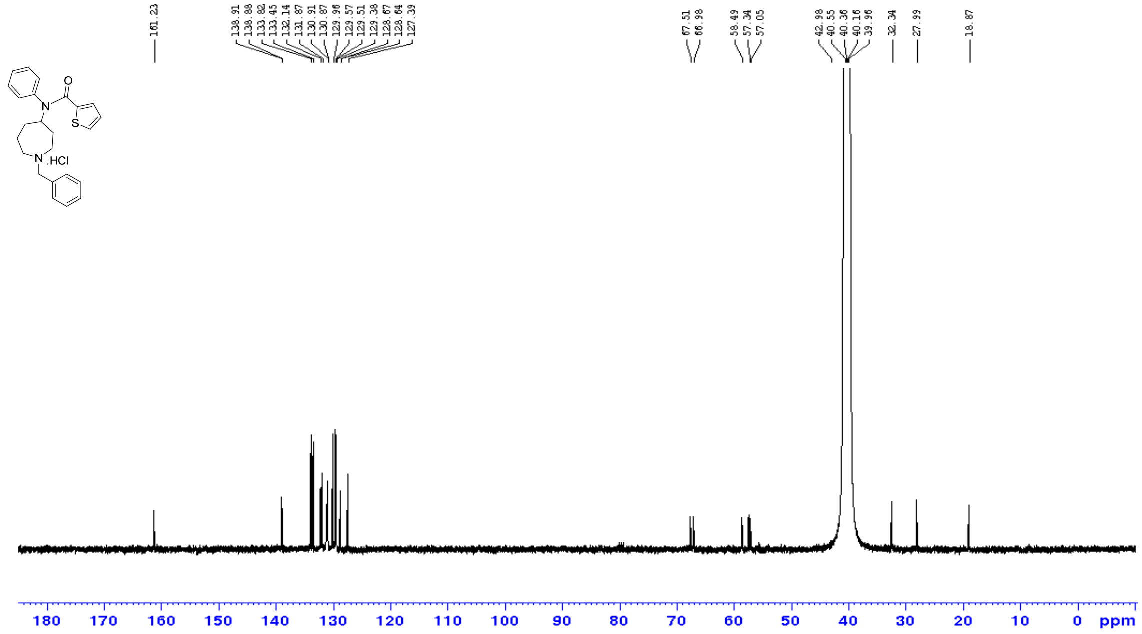

N-(1-phenethylazepan-4-yl)-N-phenylthiophene-2-carboxamide hydrogen chloride **(17)**

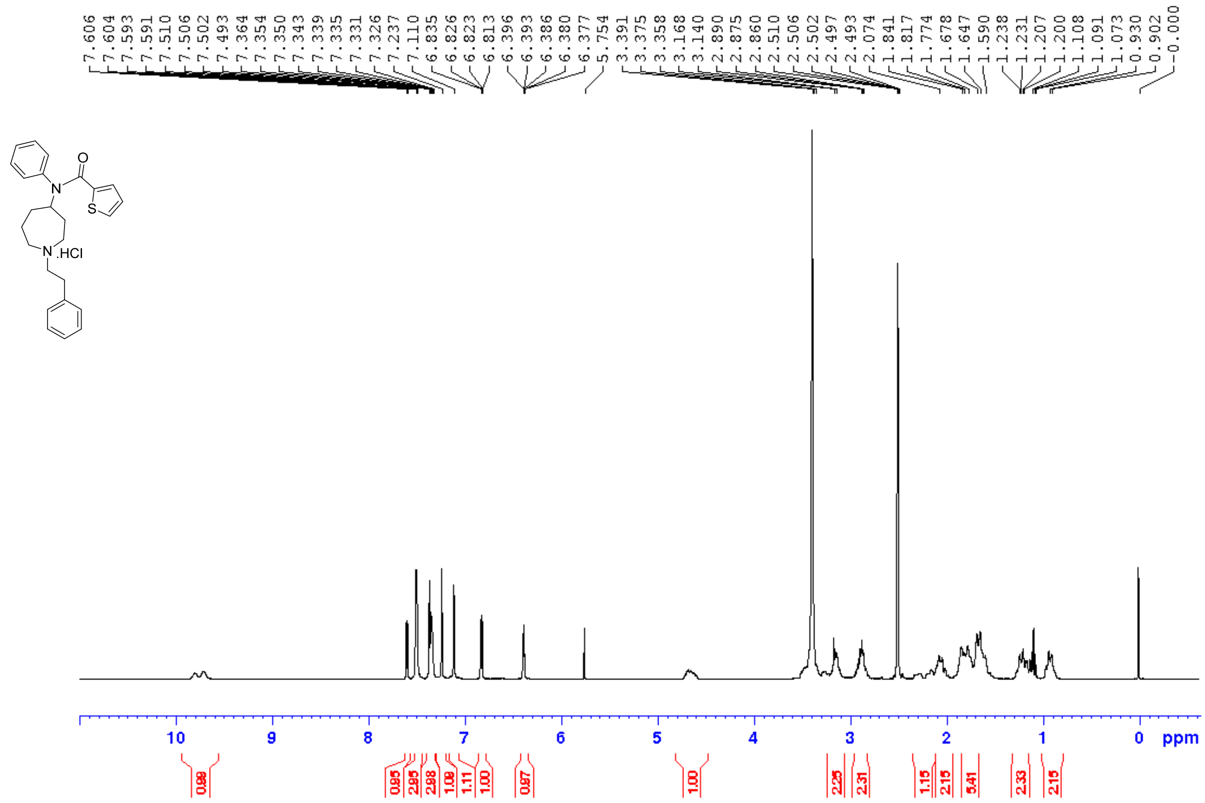

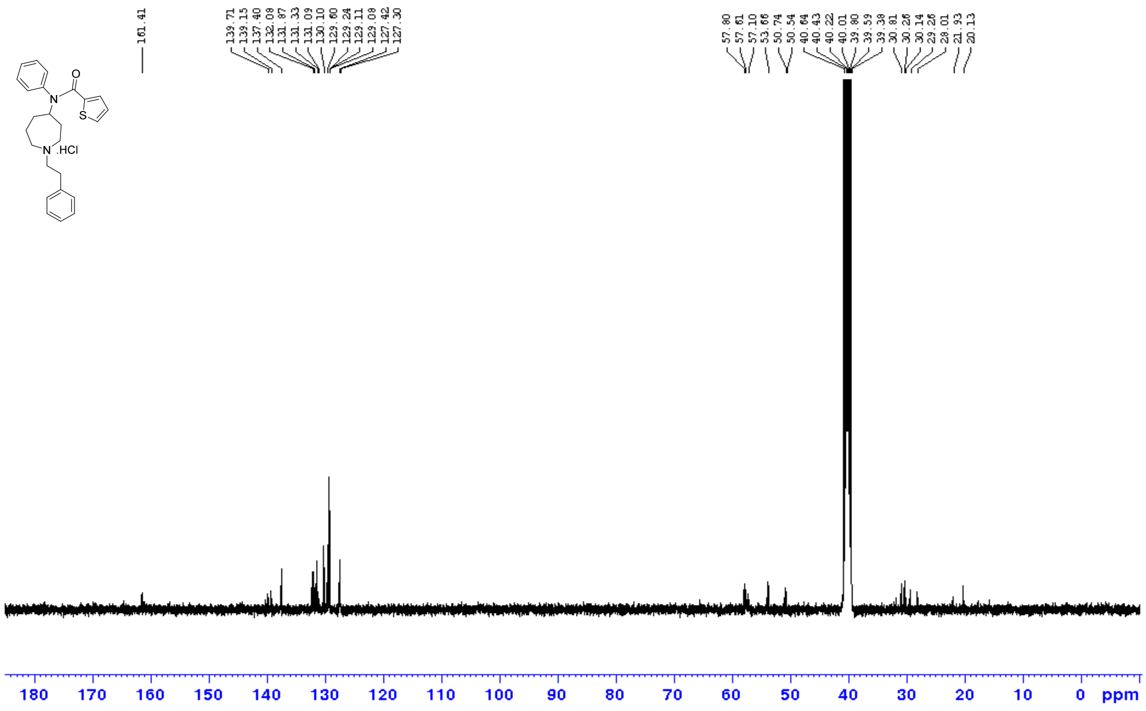

N-(1-allylazepan-4-yl)-N-phenylfuran-3-carboxamide hydrogen chloride **(18)**

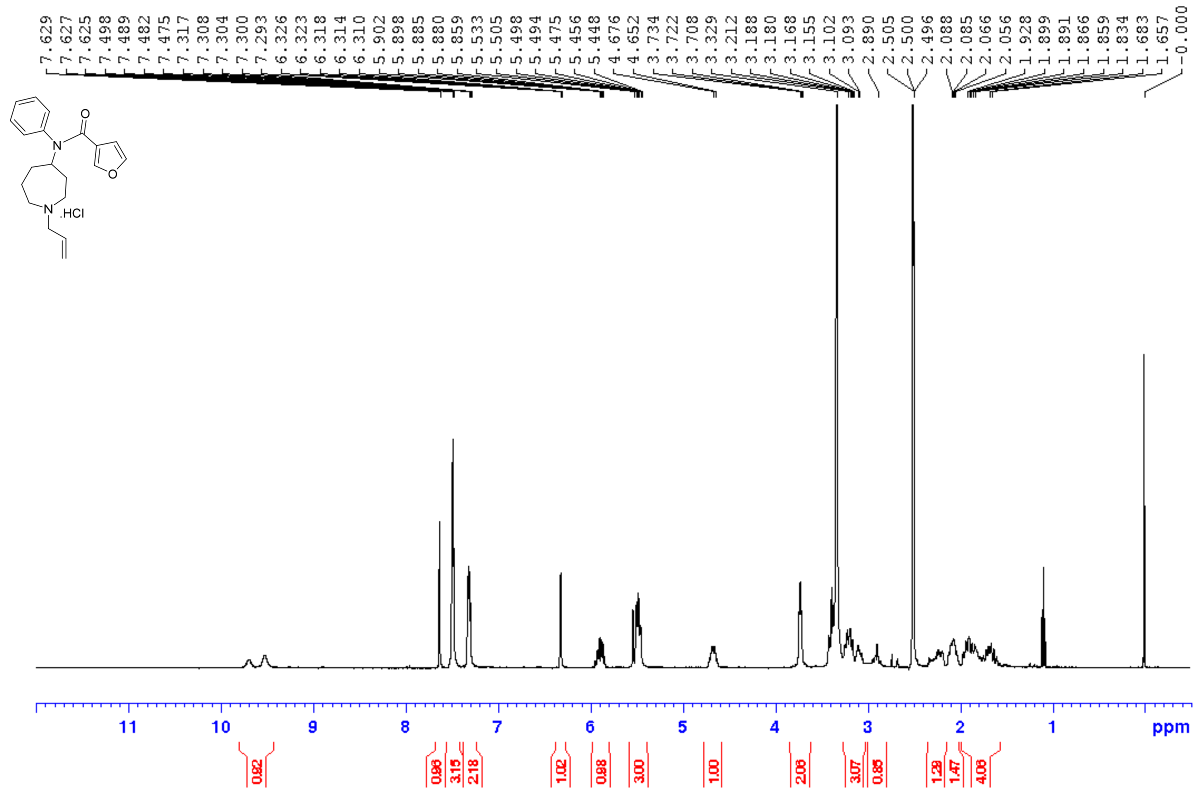

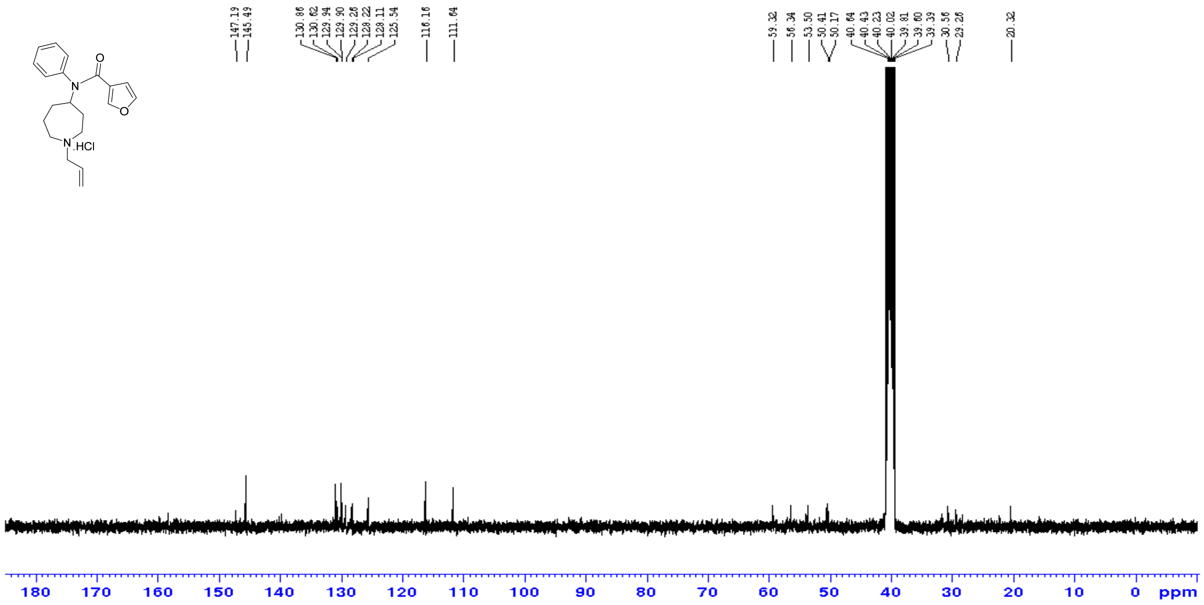

N-(1-(cyclopropylmethyl)azepan-4-yl)-N-phenylfuran-3-carboxamide hydrogen chloride **(19)**

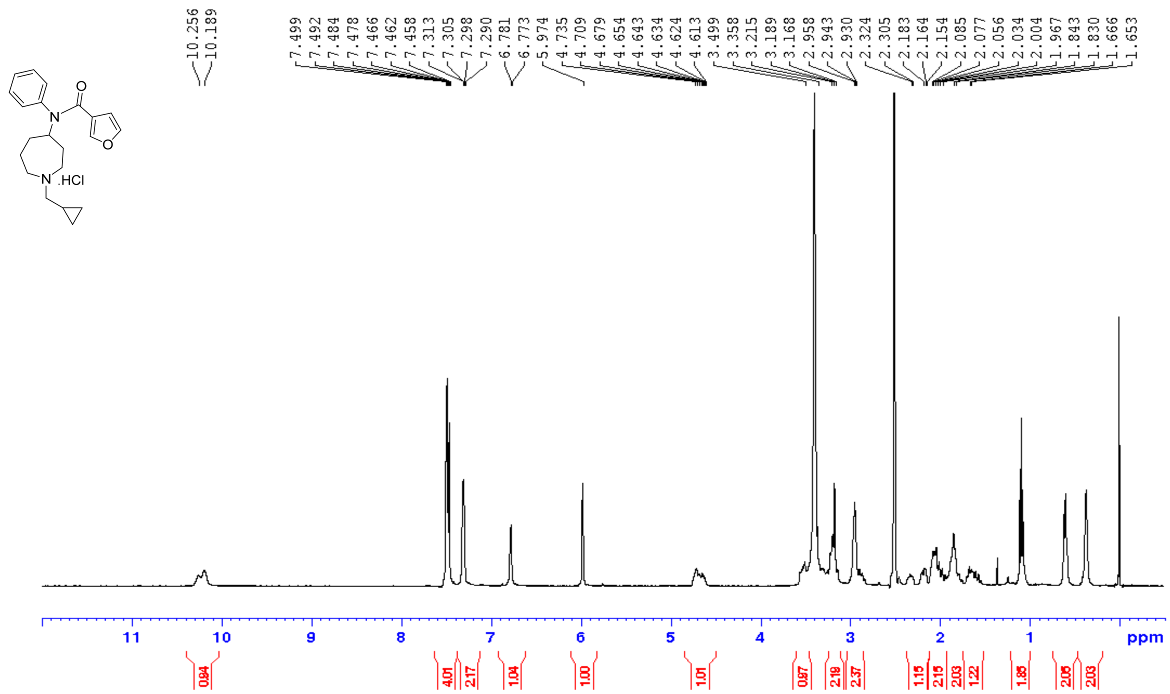

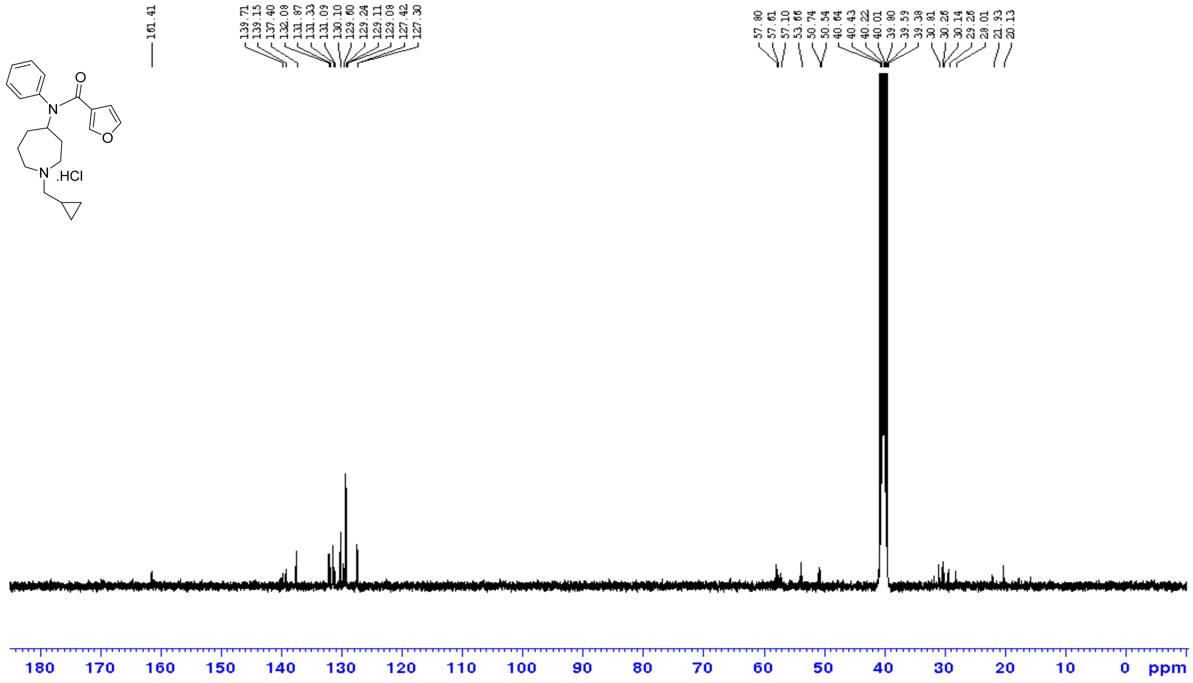

N-(1-(cyclobutylmethyl)azepan-4-yl)-N-phenylfuran-3-carboxamide hydrogen chloride **(20)**

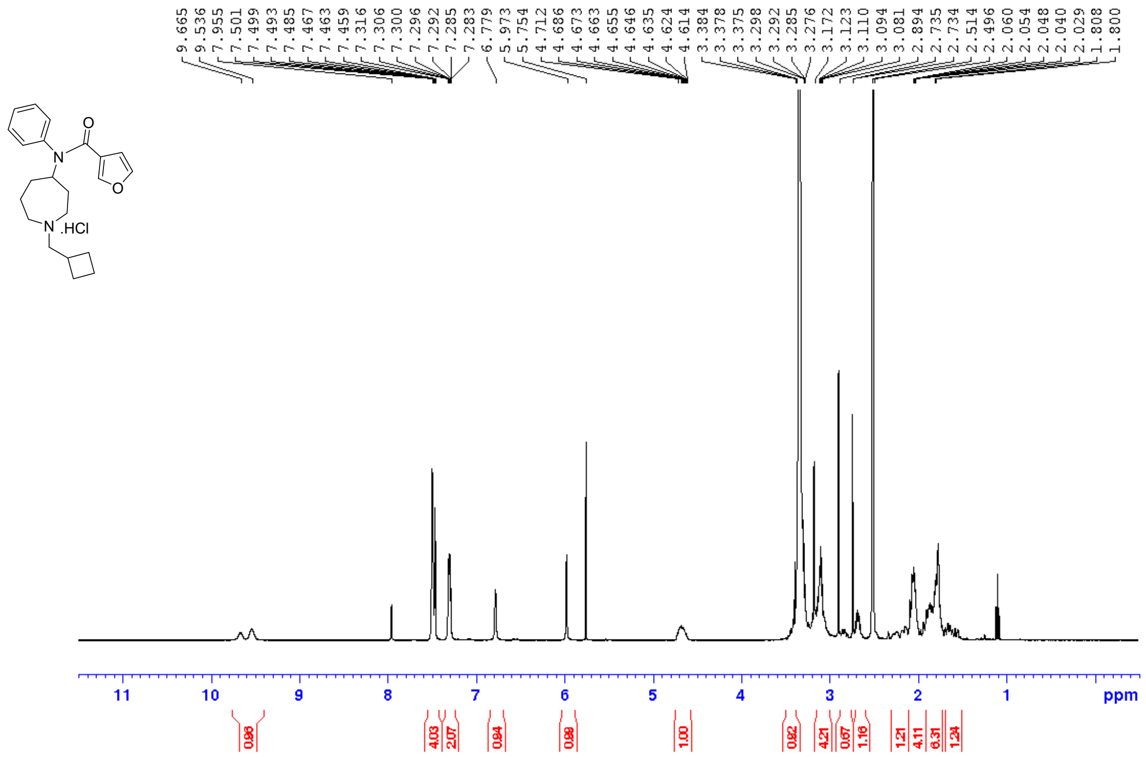

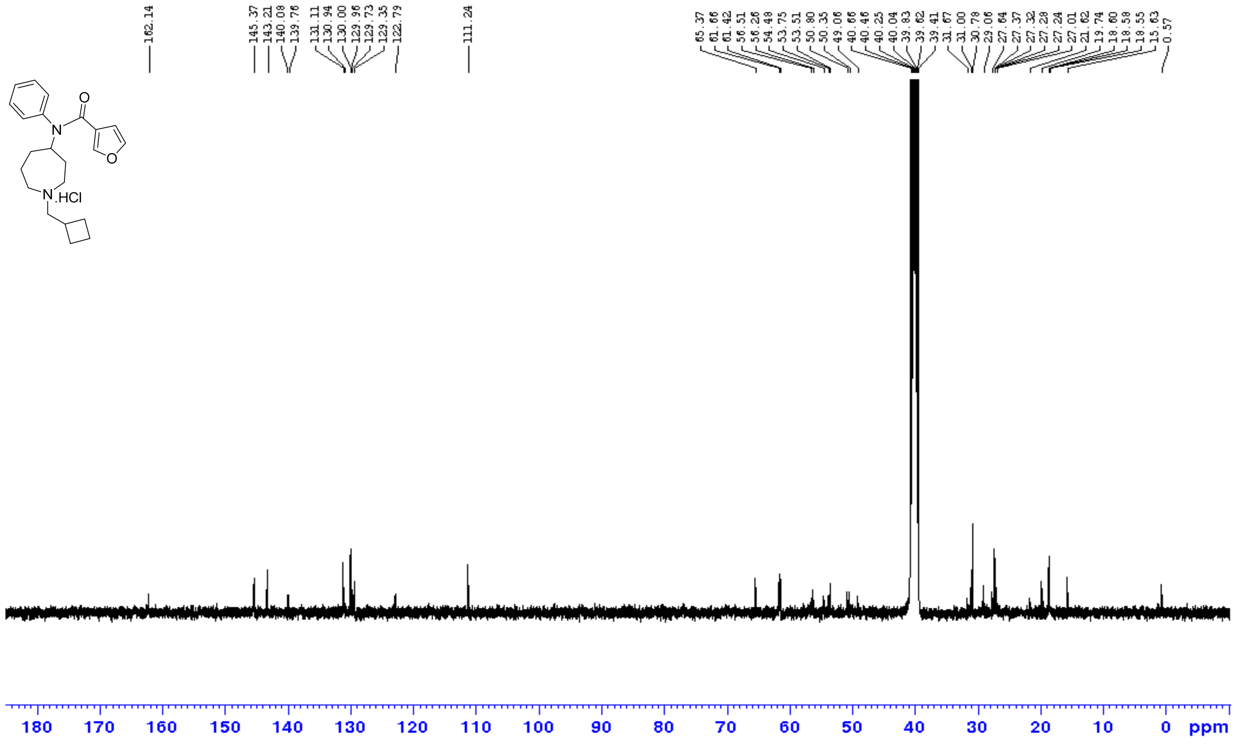

N-(1-(cyclopentylmethyl)azepan-4-yl)-N-phenylfuran-3-carboxamide hydrogen chloride **(21)**

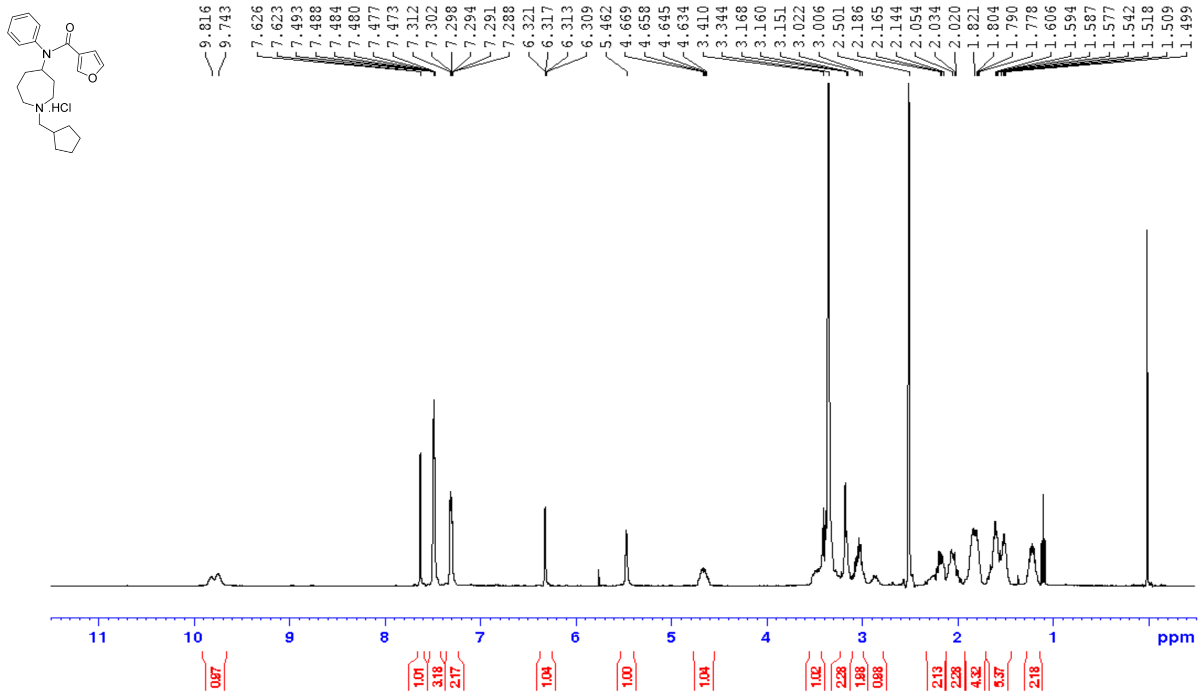

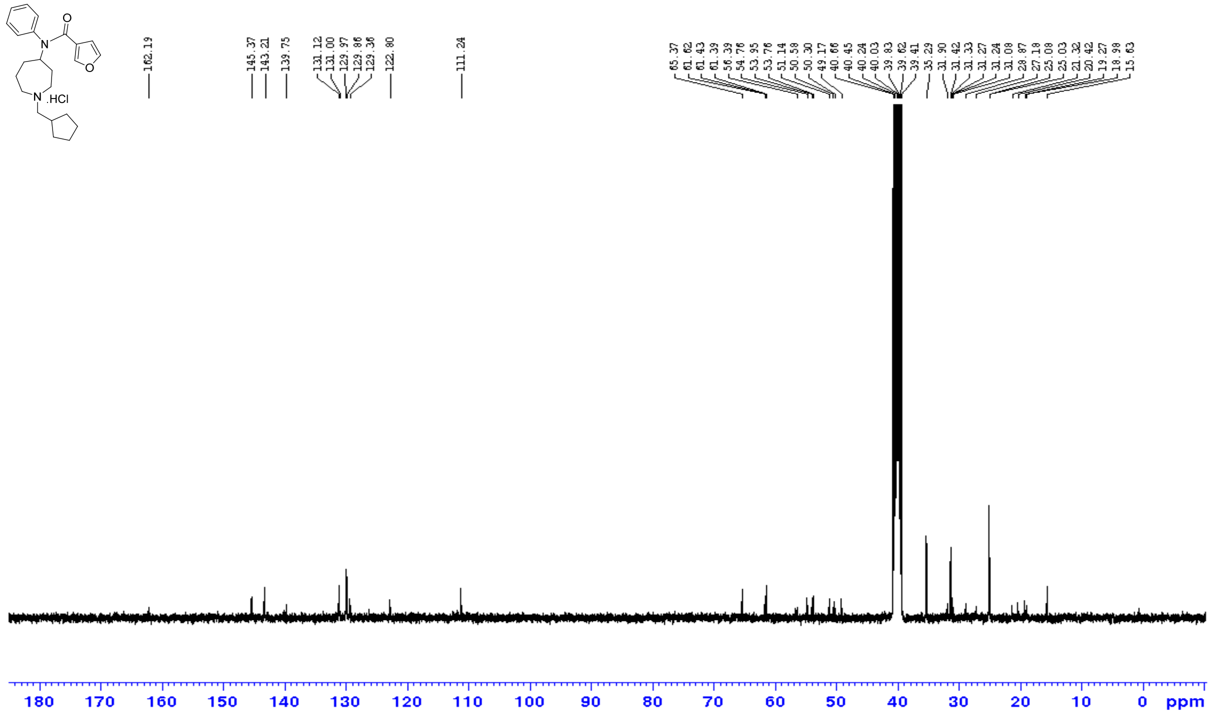

N-(1-(cyclohexylmethyl)azepan-4-yl)-N-phenylfuran-3-carboxamide hydrogen chloride **(22)**

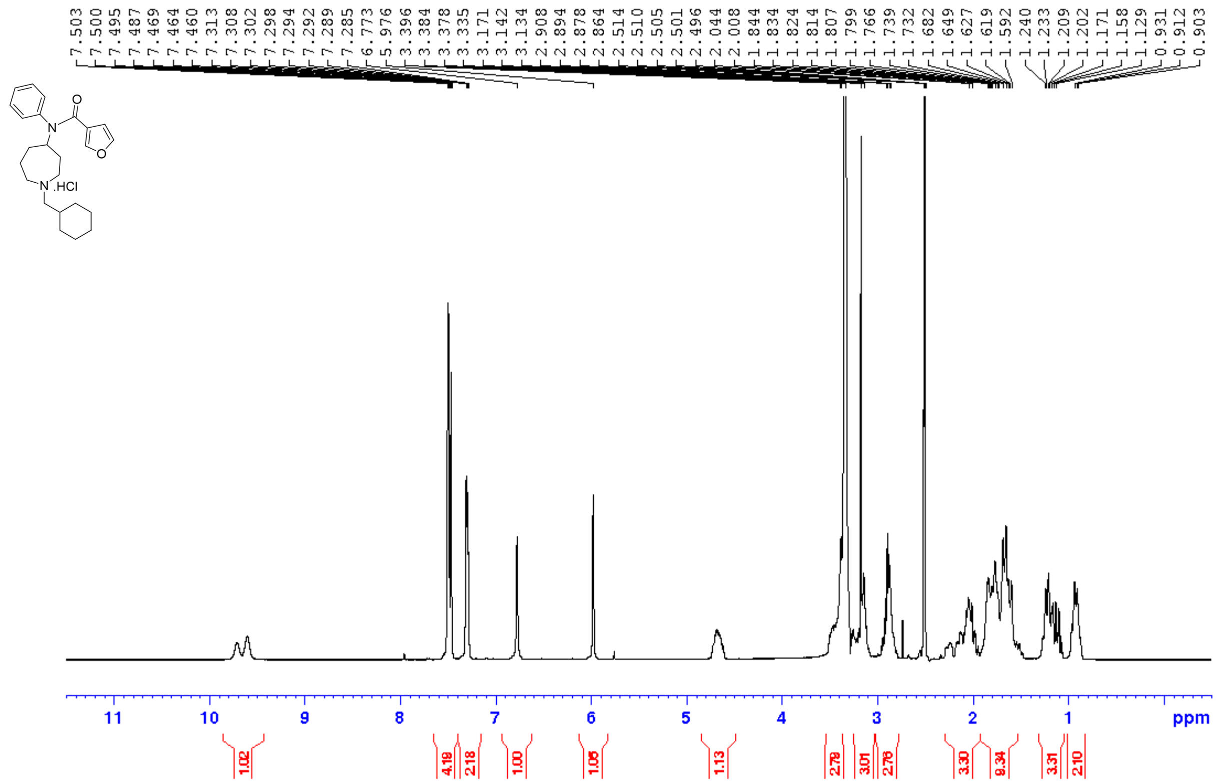

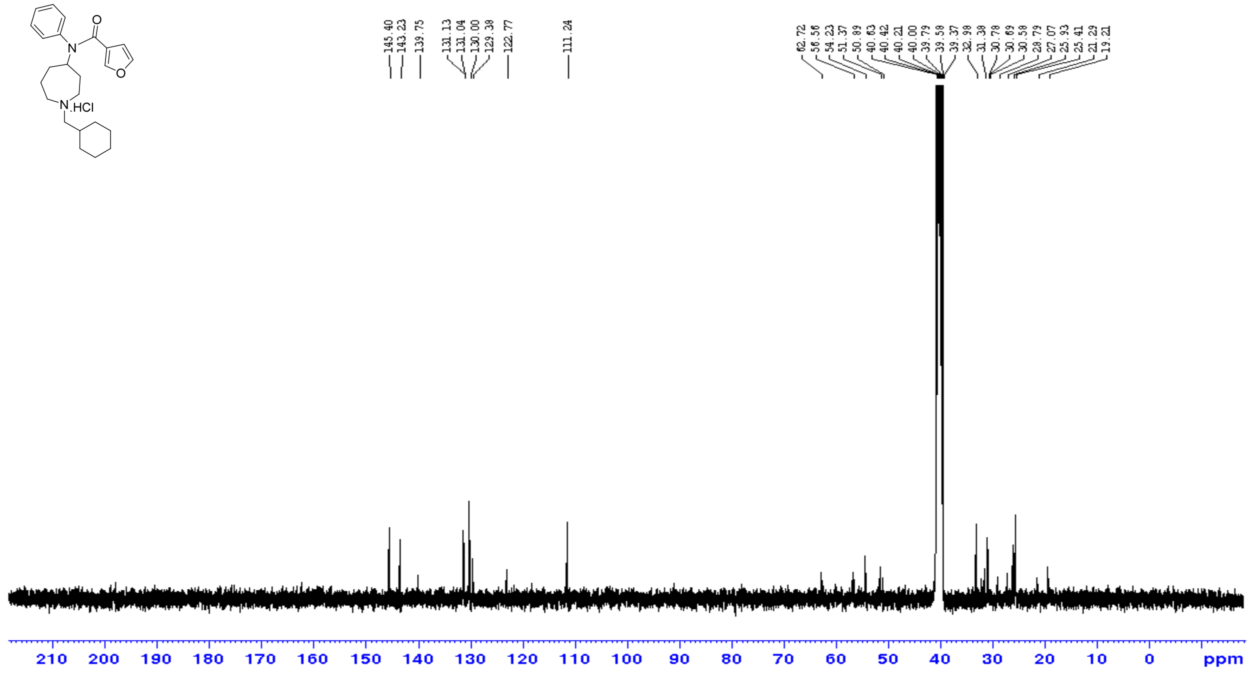

N-(1-benzylazepan-4-yl)-N-phenylfuran-3-carboxamide hydrogen chloride **(23)**

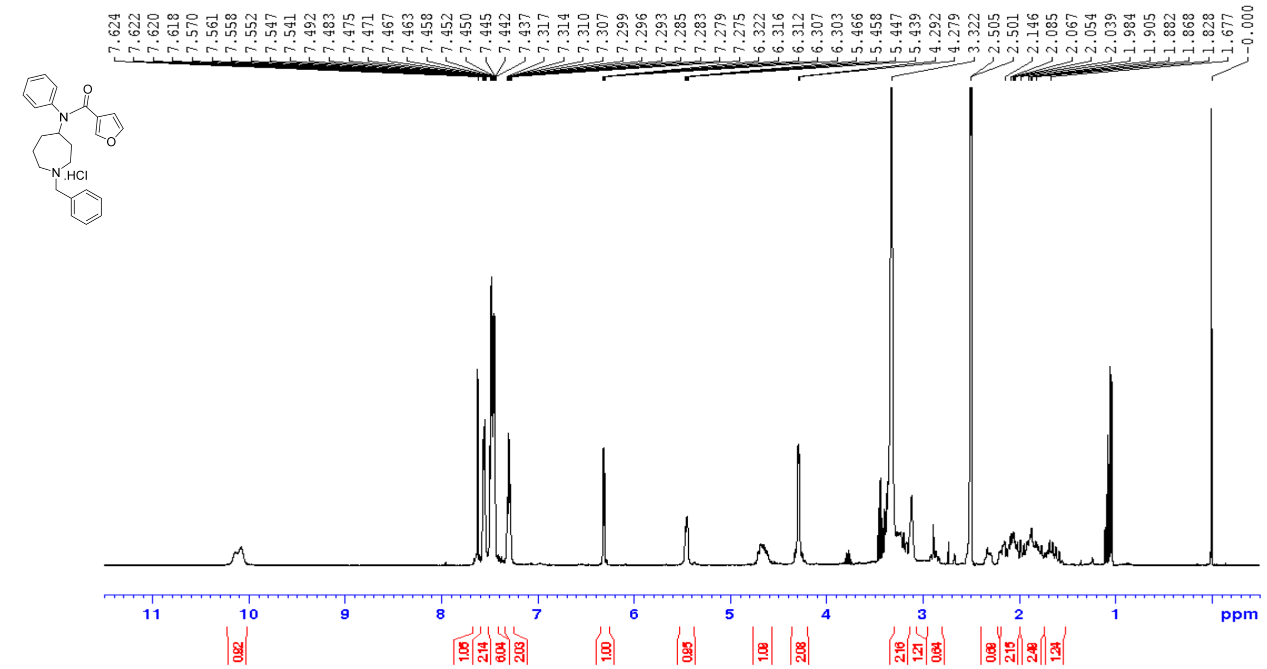

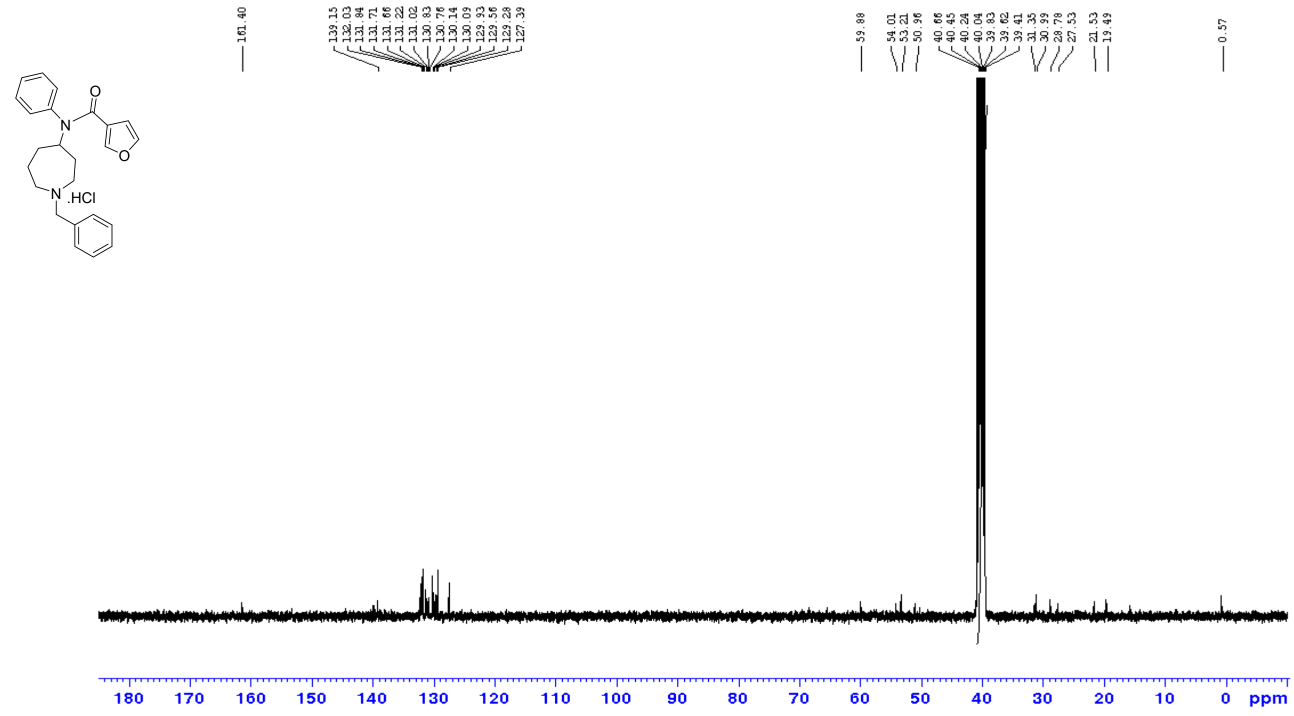

N-(1-phenethylazepan-4-yl)-N-phenylfuran-3-carboxamide hydrogen chloride **(24)**

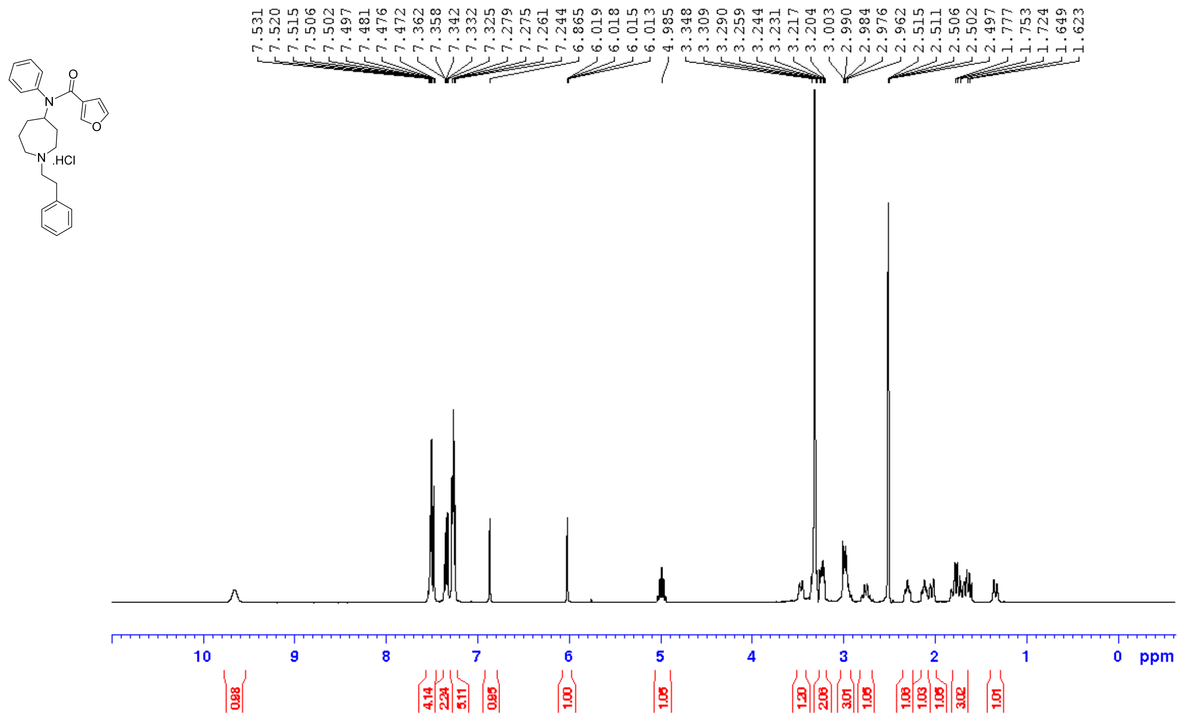

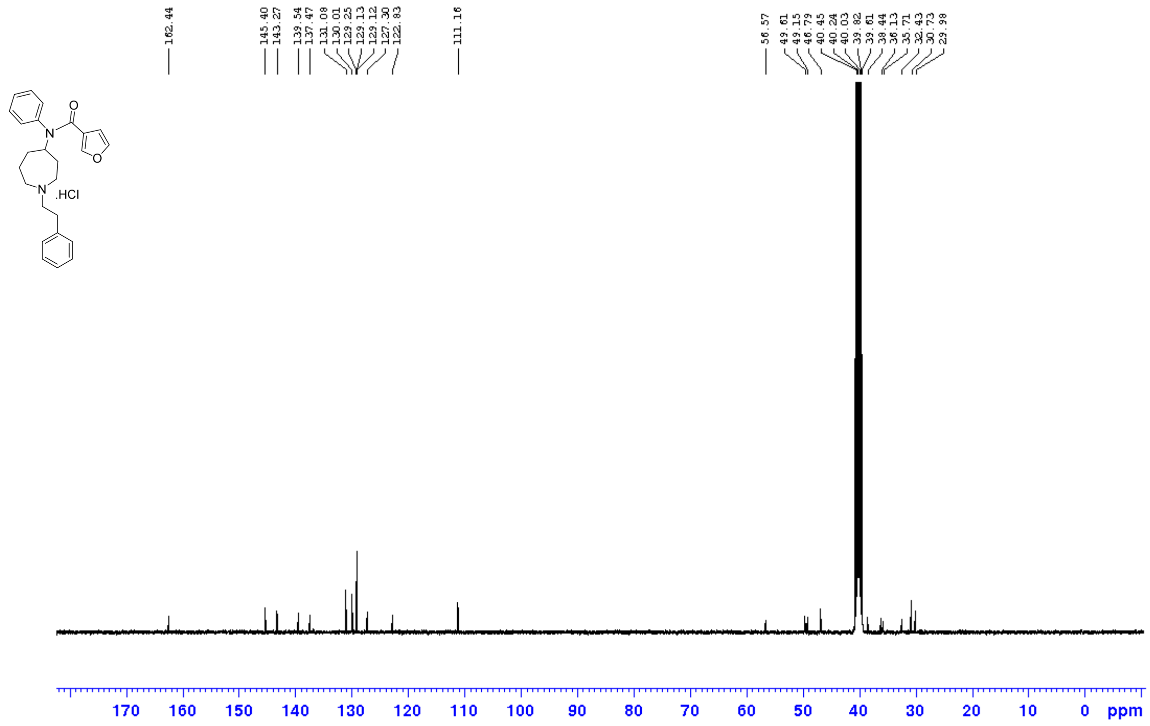

N-(1-allylazepan-4-yl)-N-phenyl-1H-pyrrole-2-carboxamide hydrochloride **(25)**

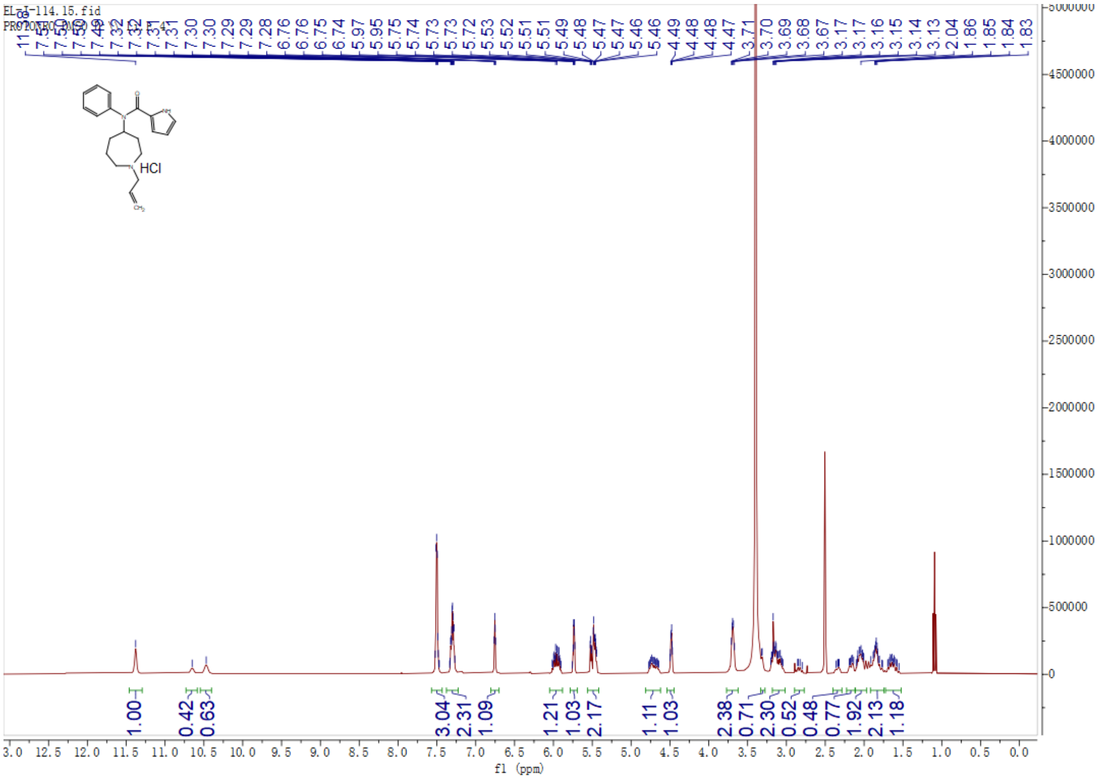

HCl
1.00
0.42
0.63
3.04
2.31
1.09
1.21
1.03
2.17
1.11
1.03
2.38
0.71
2.30
0.52
0.48
0.77
1.92
2.13
1.18
f1 (ppm)

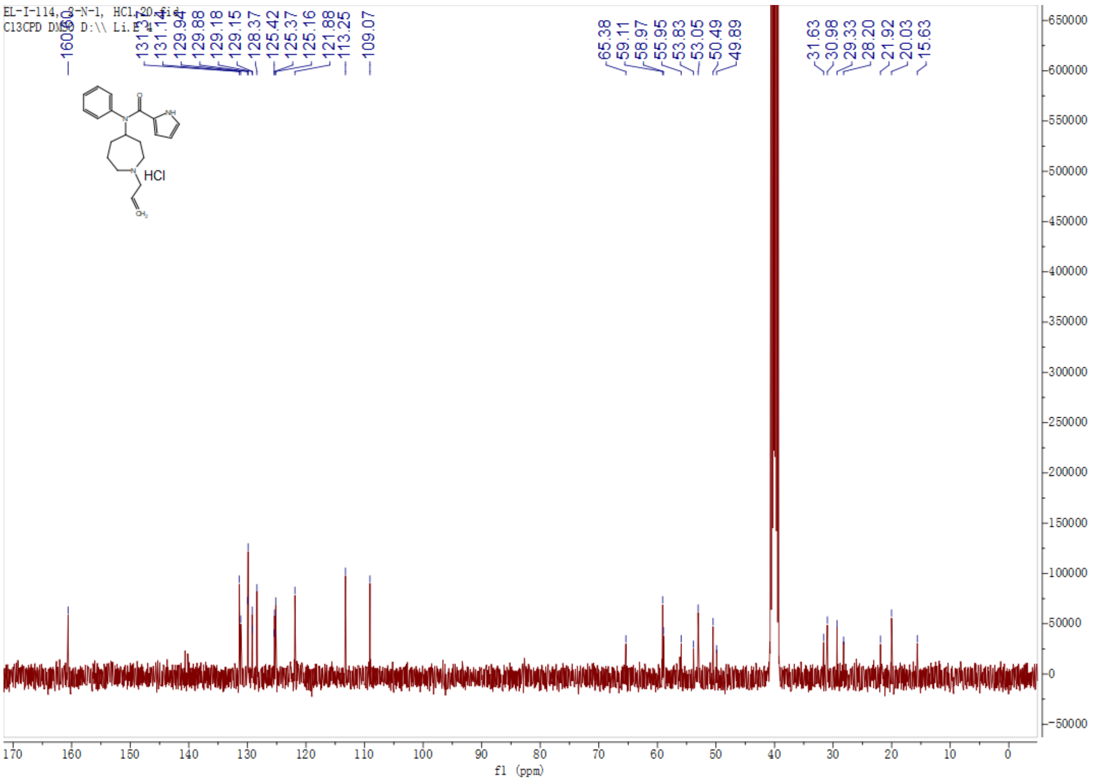

HCl
131.14
129.94
129.88
129.18
129.15
128.37
125.42
125.37
125.16
121.88
113.25
109.07
65.38
59.11
58.97
55.95
53.83
53.05
50.49
49.89
31.63
30.98
29.33
28.20
21.92
20.03
15.63
f1 (ppm)

N-(1-(cyclopropylmethyl)azepan-4-yl)-N-phenyl-1H-pyrrole-2-carboxamide hydrochloride **(26)**

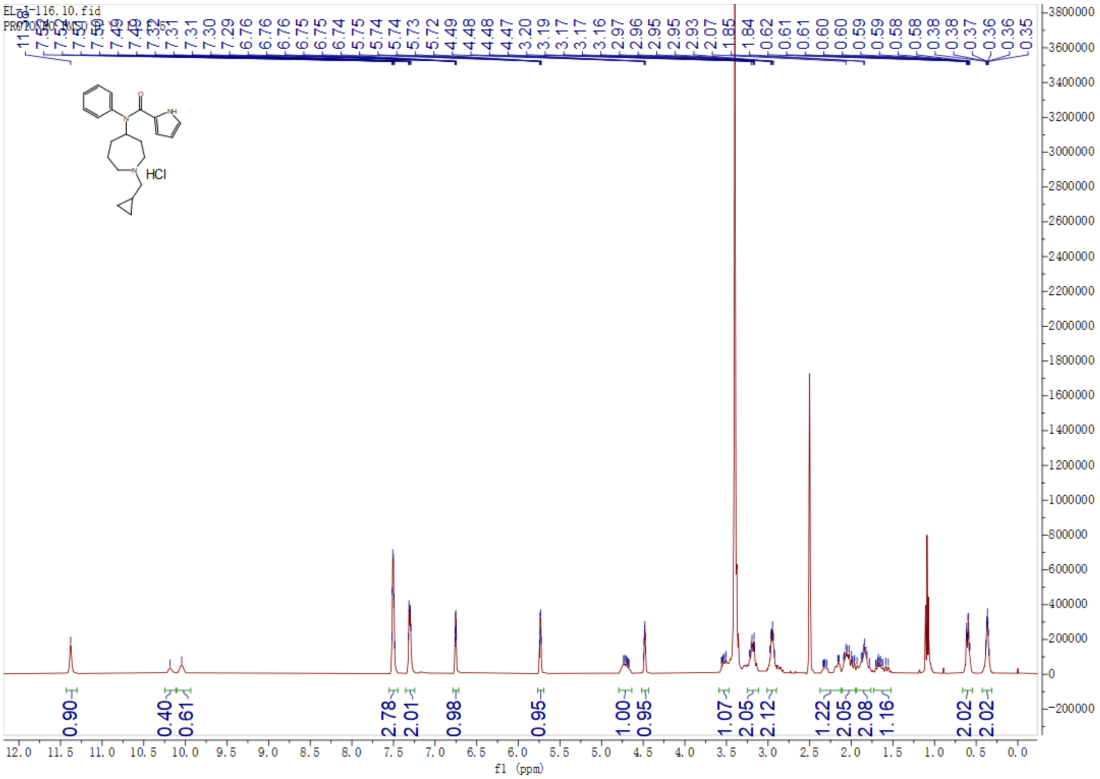

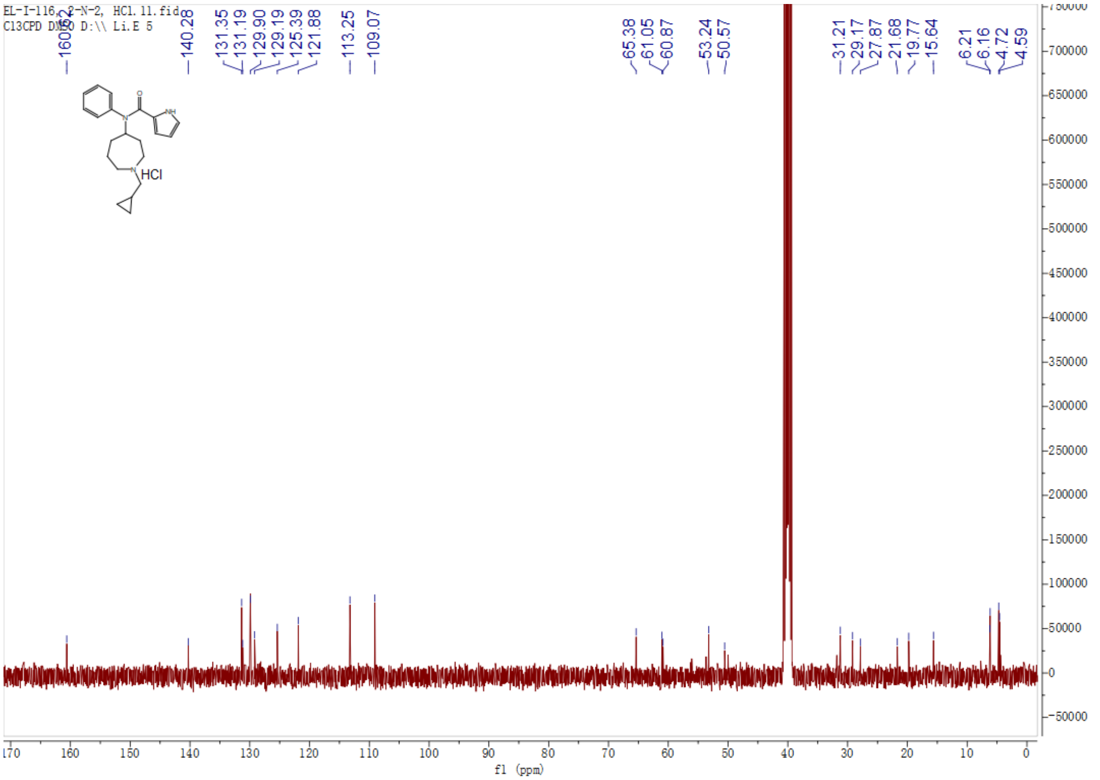

N-(1-(cyclobutylmethyl)azepan-4-yl)-N-phenyl-1H-pyrrole-2-carboxamide hydrochloride **(27)**

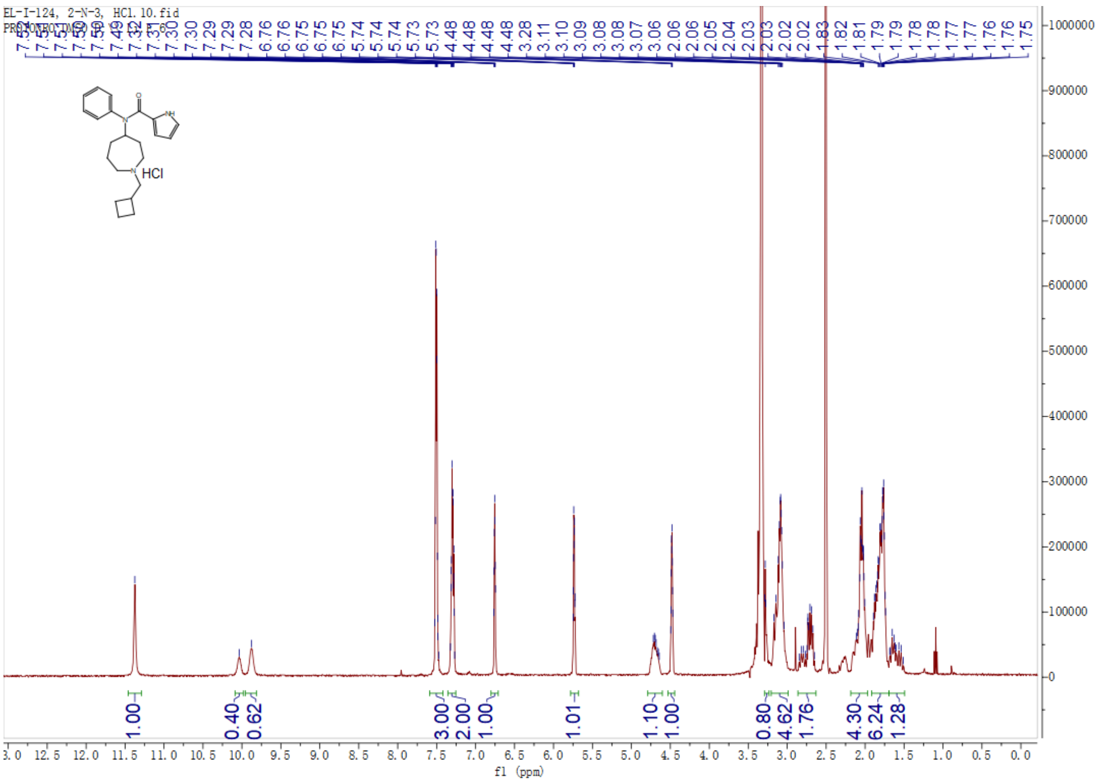

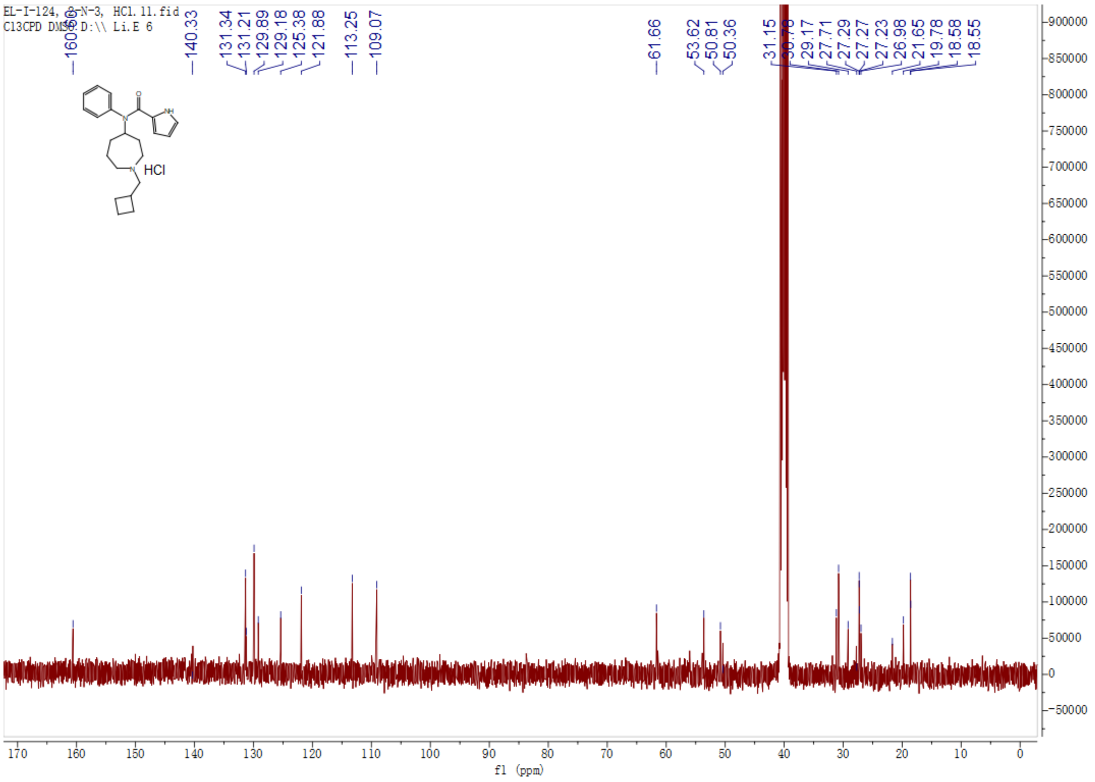

N-(1-(cyclopentylmethyl)azepan-4-yl)-N-phenyl-1H-pyrrole-2-carboxamide hydrochloride **(28)**

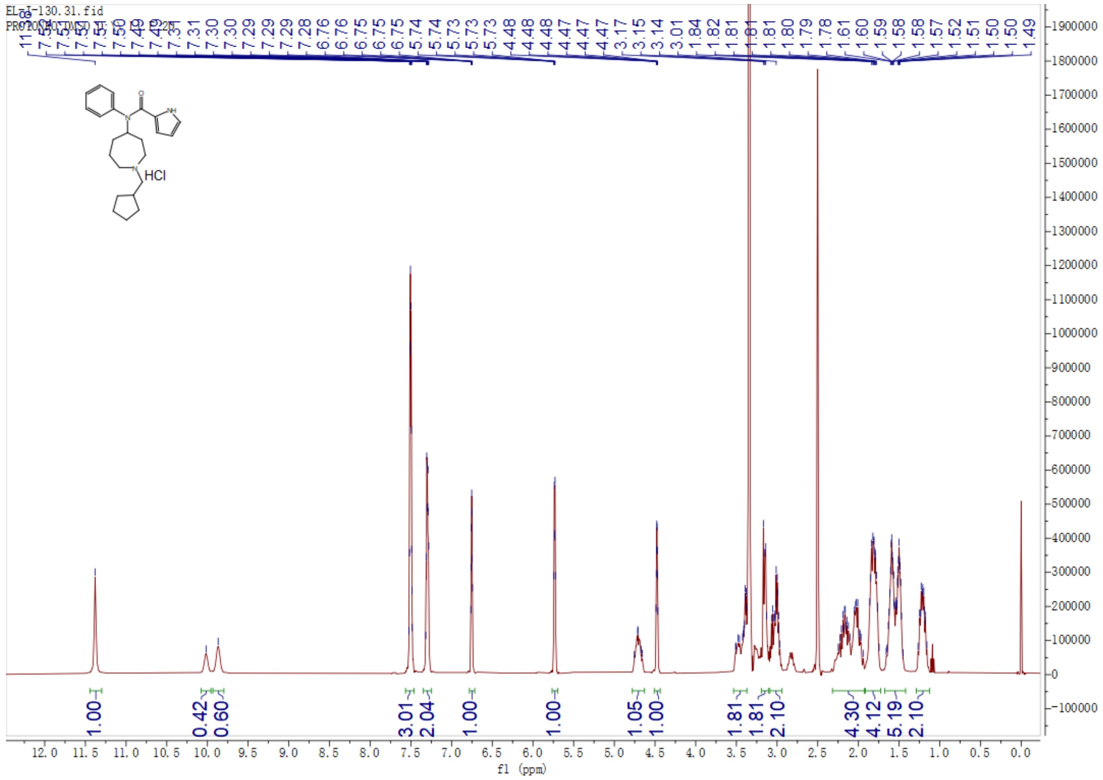

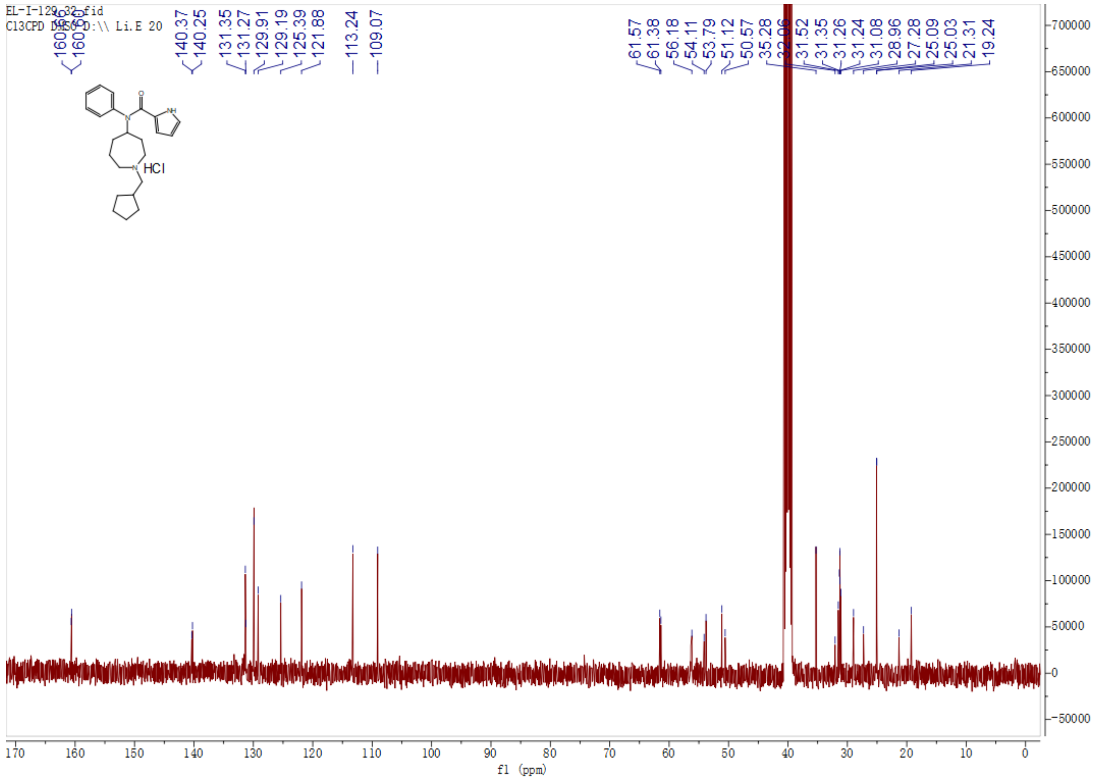

N-(1-(cyclopentylmethyl)azepan-4-yl)-N-phenyl-1H-pyrrole-2-carboxamide hydrochloride **(29)**

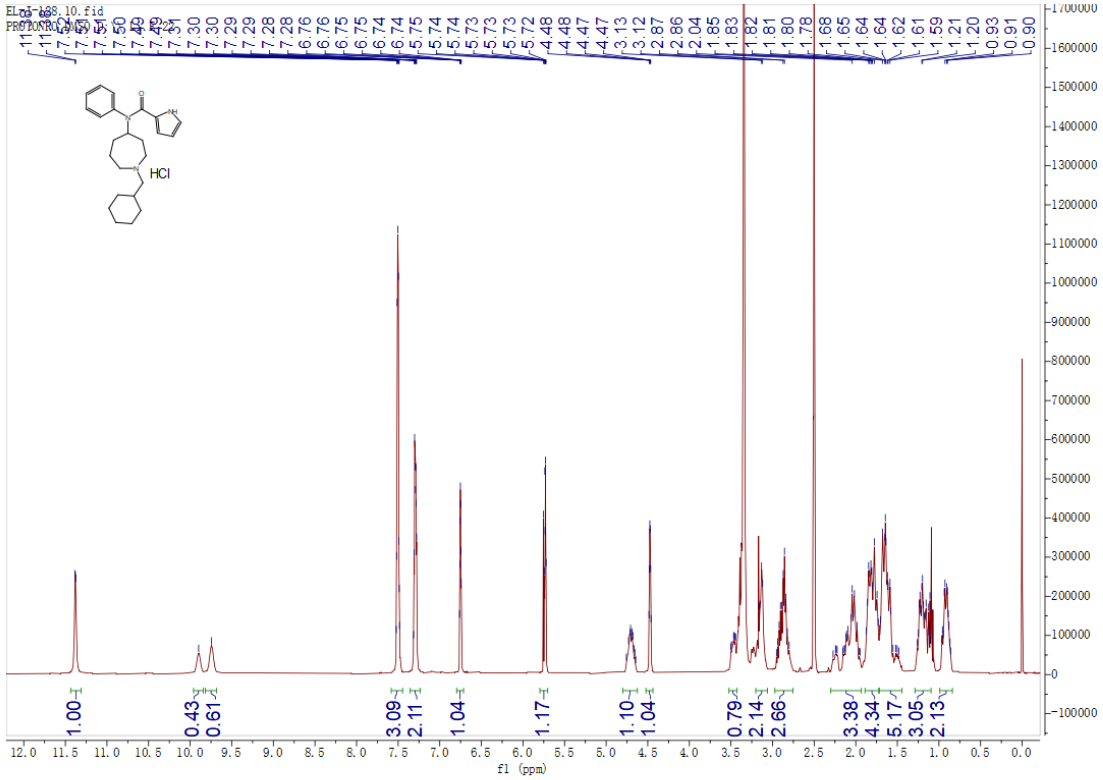

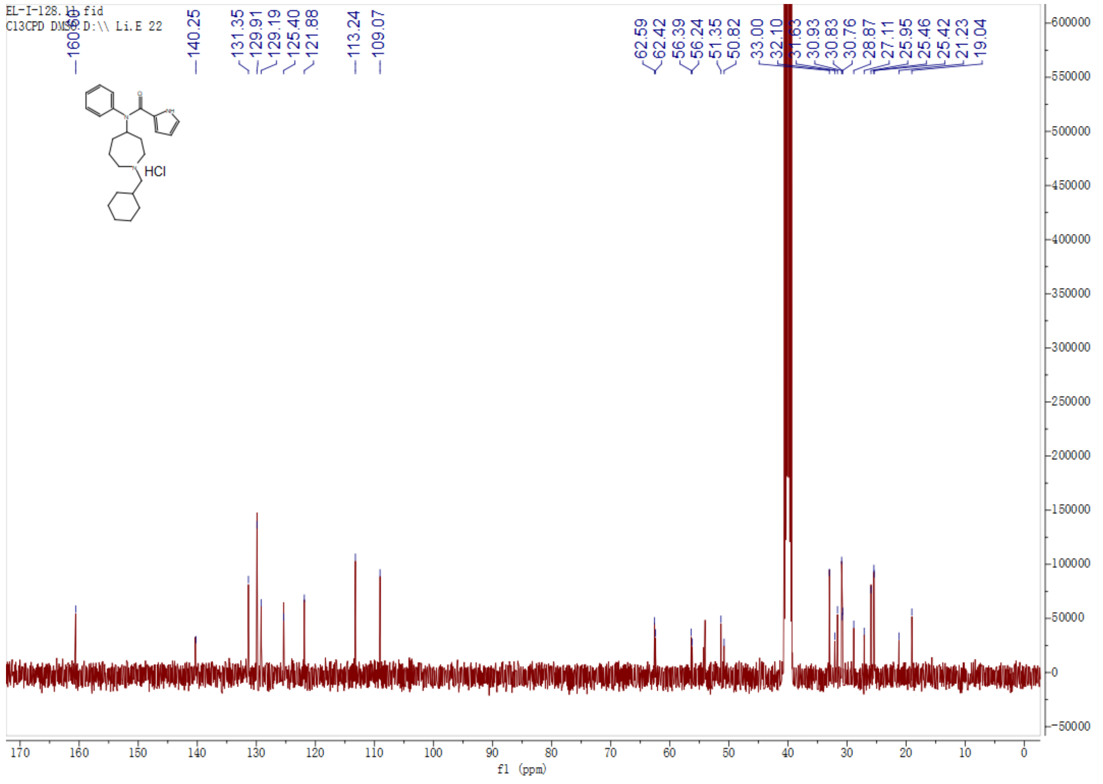

N-(1-benzylazepan-4-yl)-N-phenyl-1H-pyrrole-2-carboxamide hydrochloride **(30)**

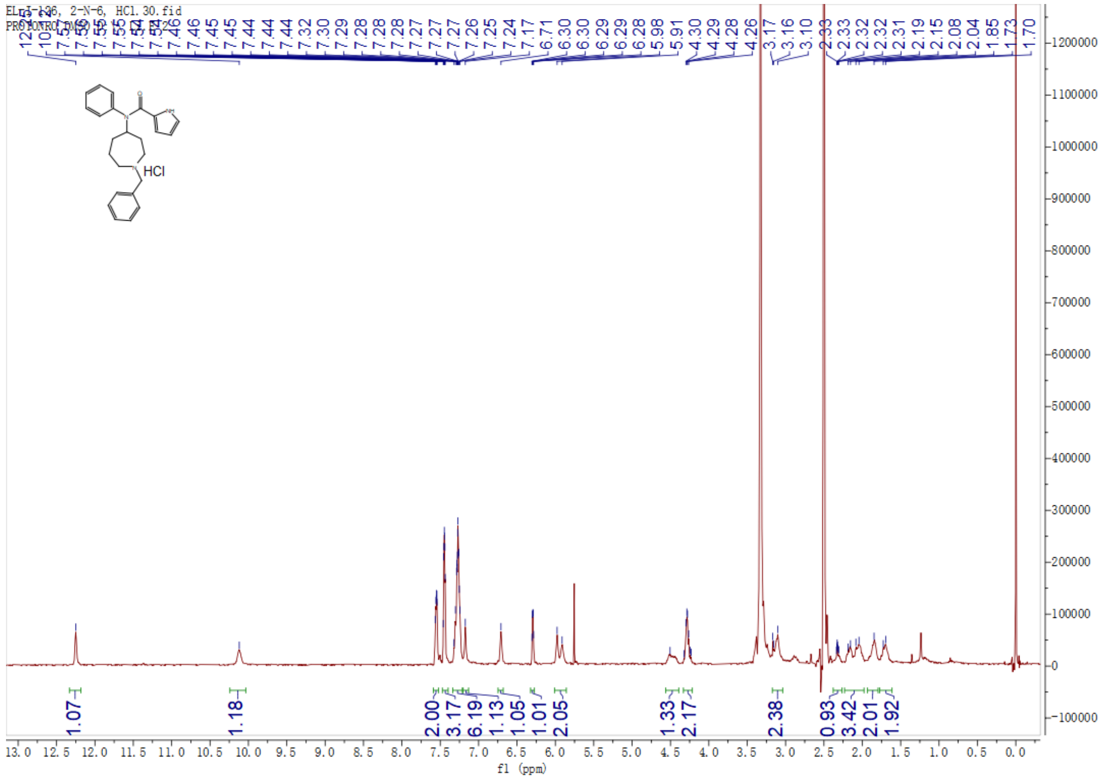

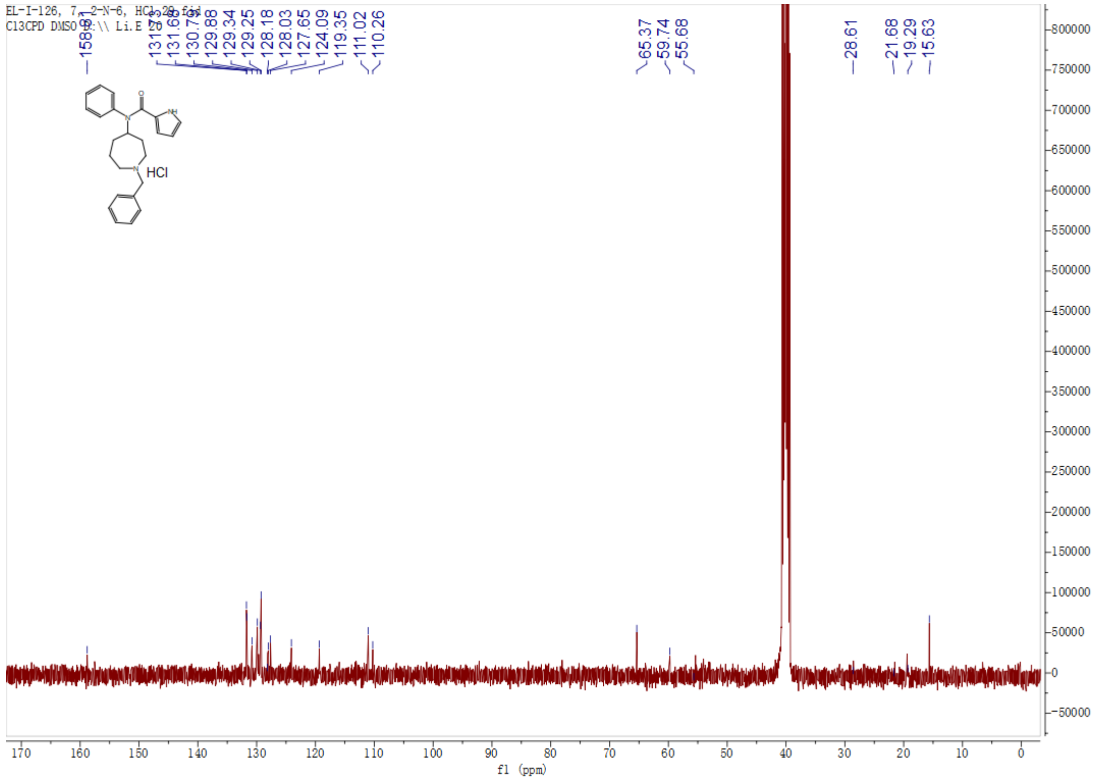

N-(1-benzylazepan-4-yl)-N-phenyl-1H-pyrrole-2-carboxamide hydrochloride **(31)**

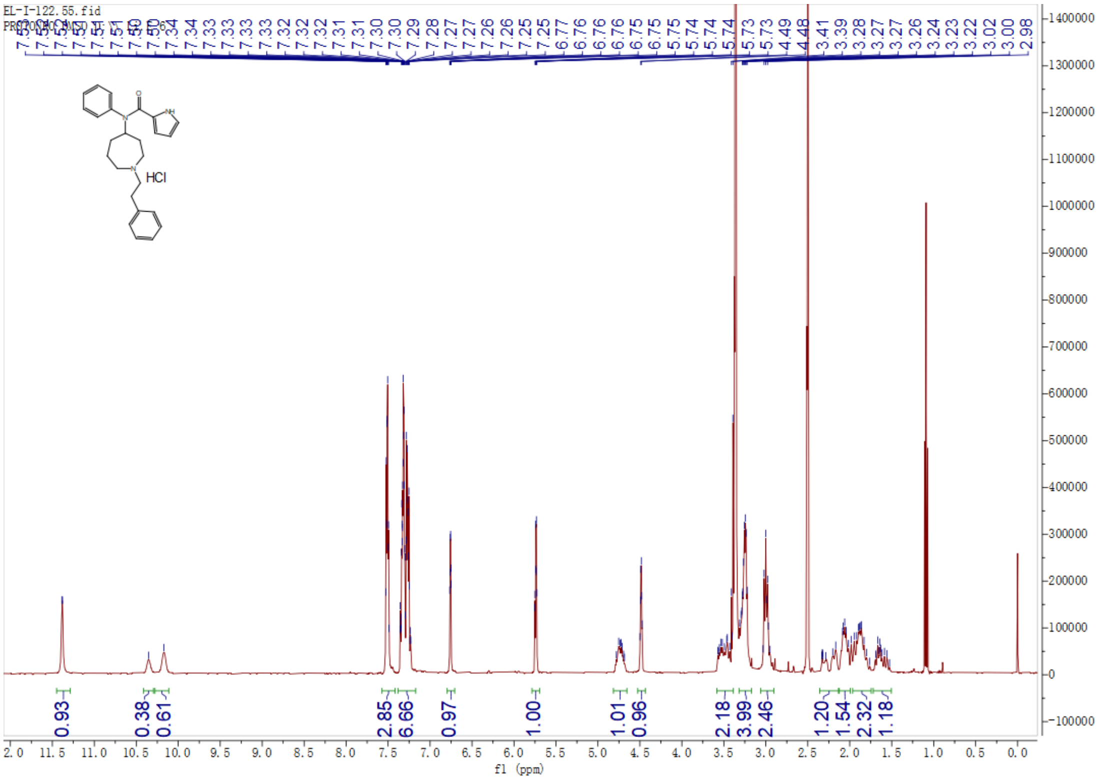

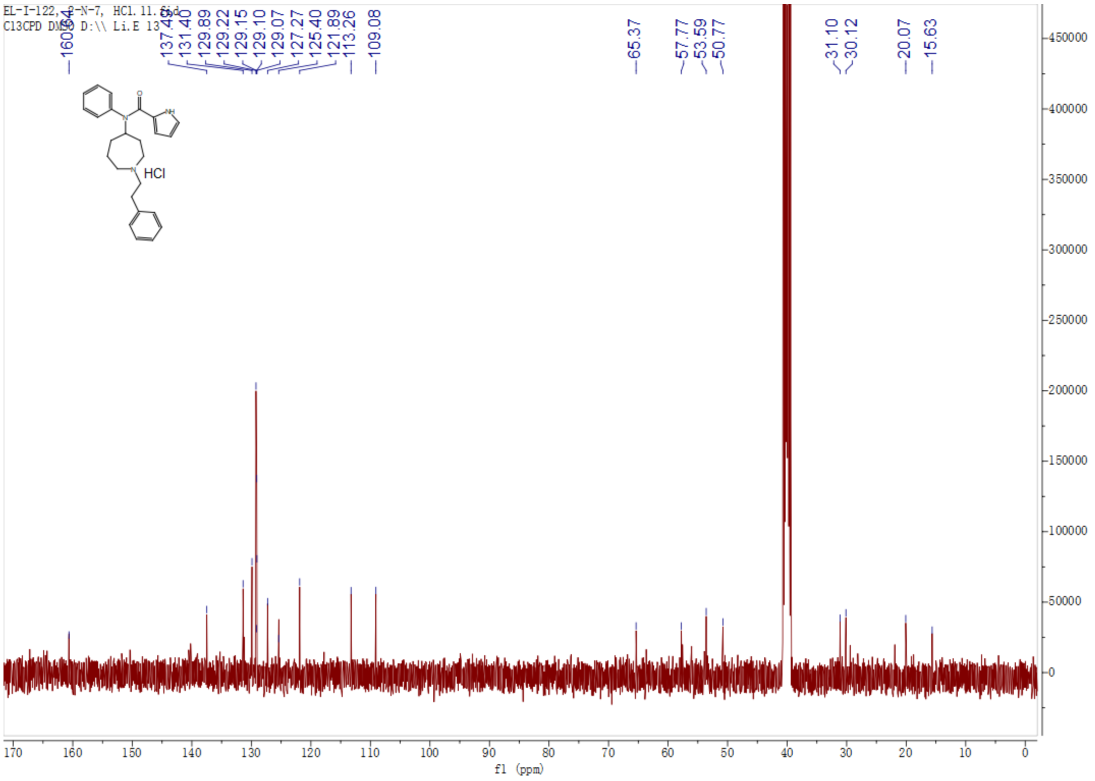

N-(1-allylazepan-4-yl)-N-phenyl-1H-pyrrole-3-carboxamide hydrochloride **(32)**

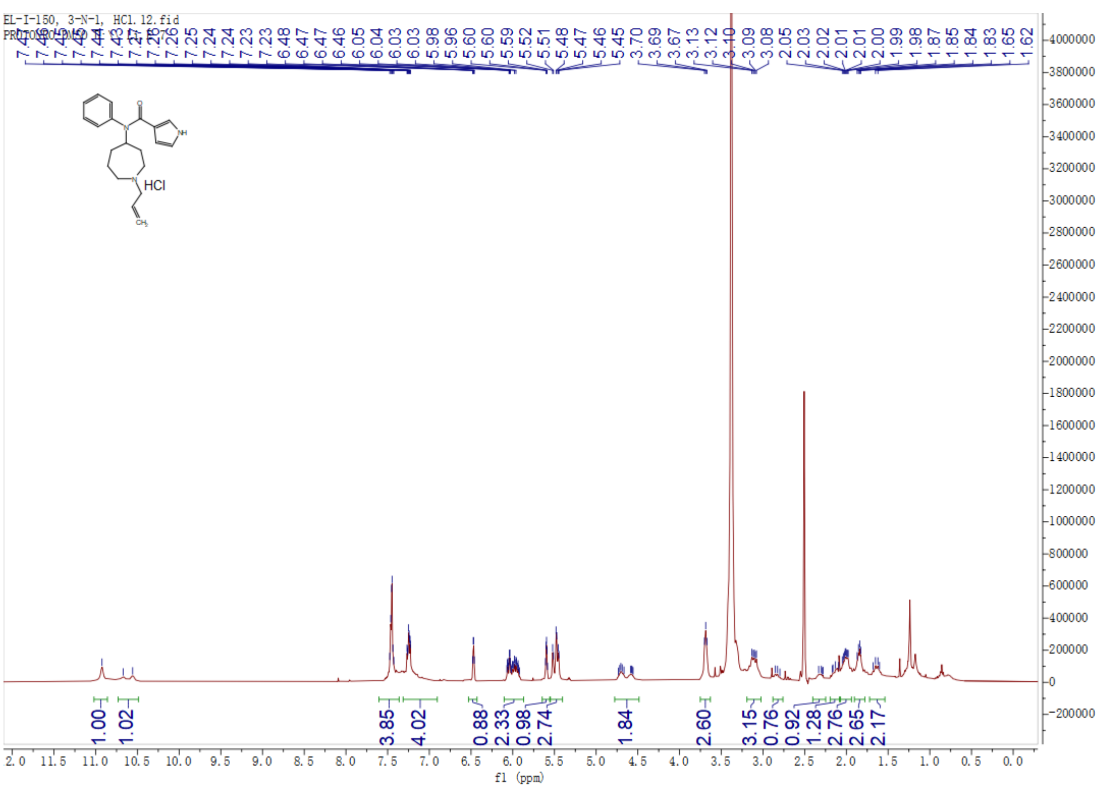

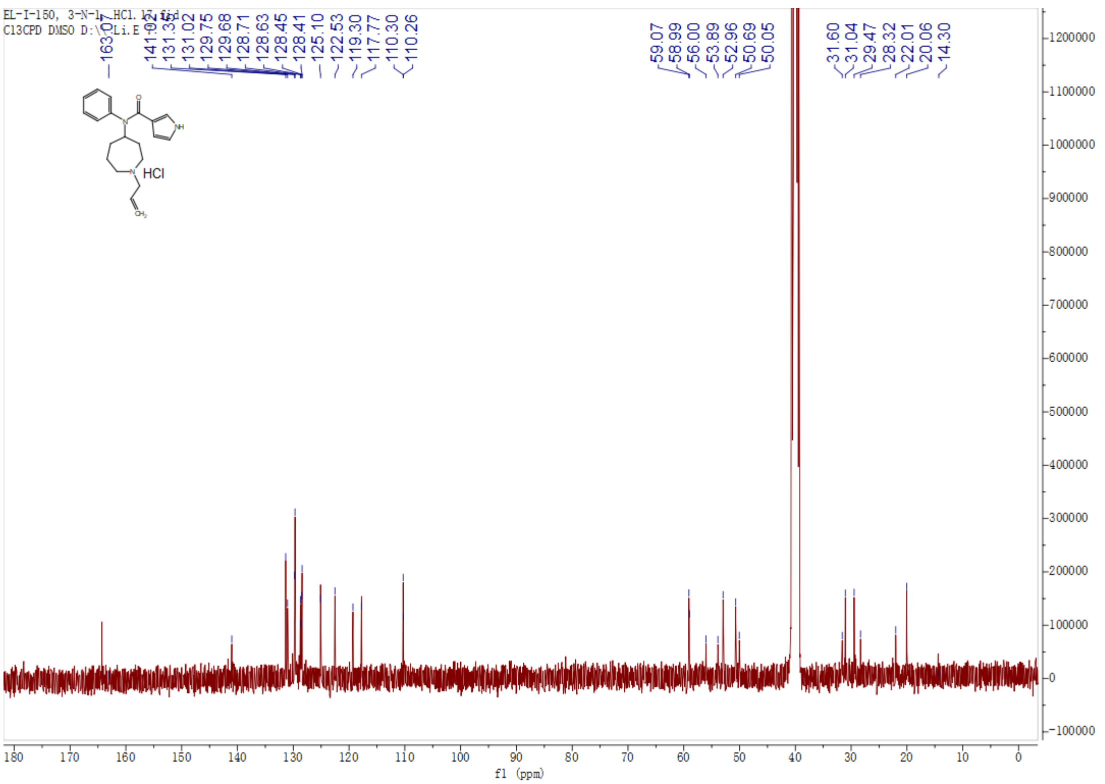

N-(1-(cyclopropylmethyl)azepan-4-yl)-N-phenyl-1H-pyrrole-3-carboxamide **(33)**

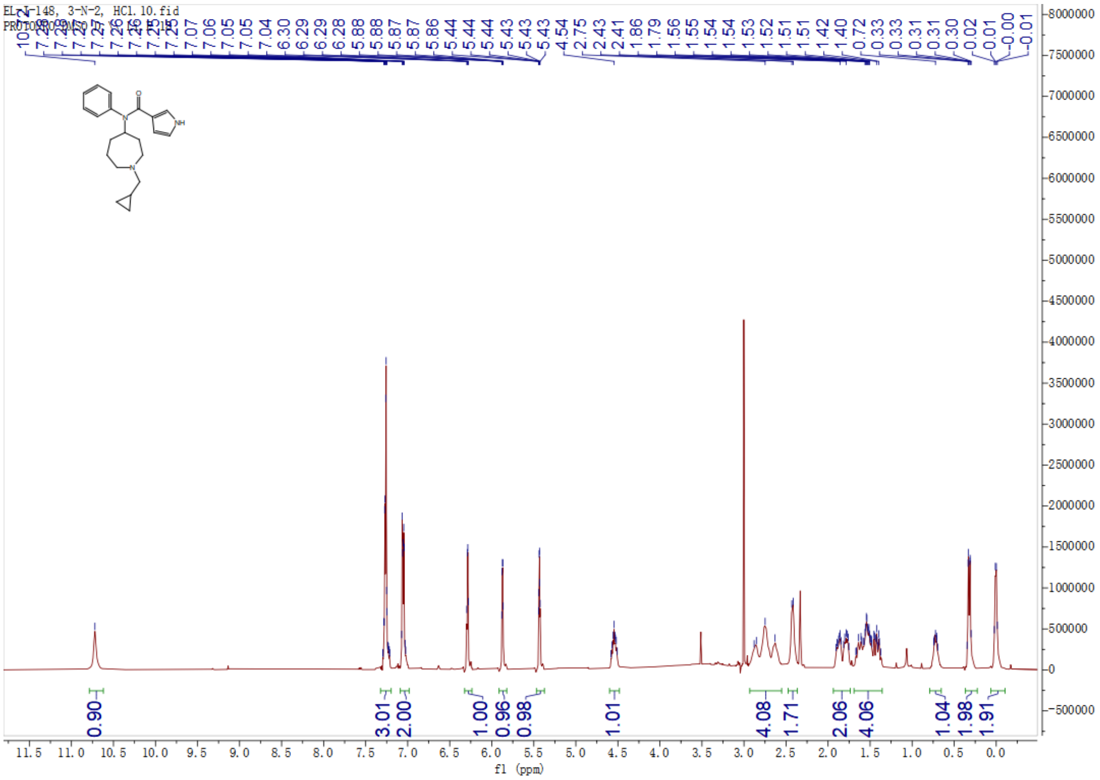

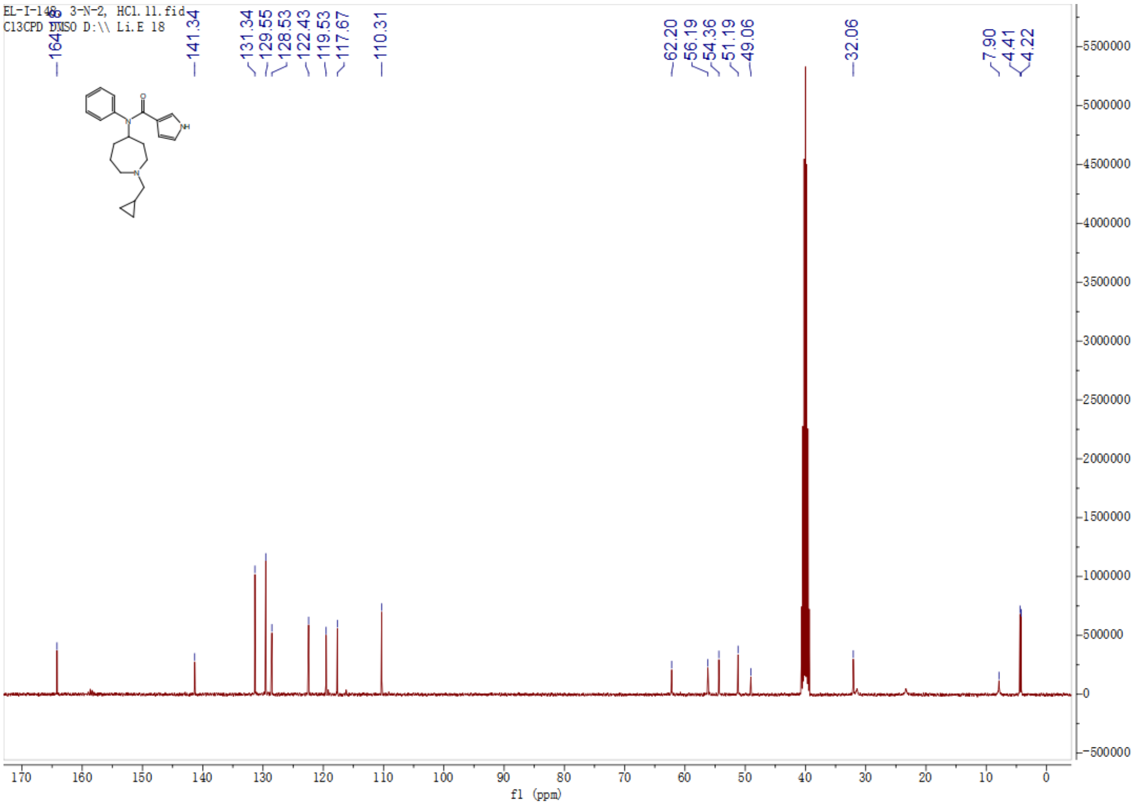

N-(1-(cyclobutylmethyl)azepan-4-yl)-N-phenyl-1H-pyrrole-3-carboxamide hydrochloride **(34)**

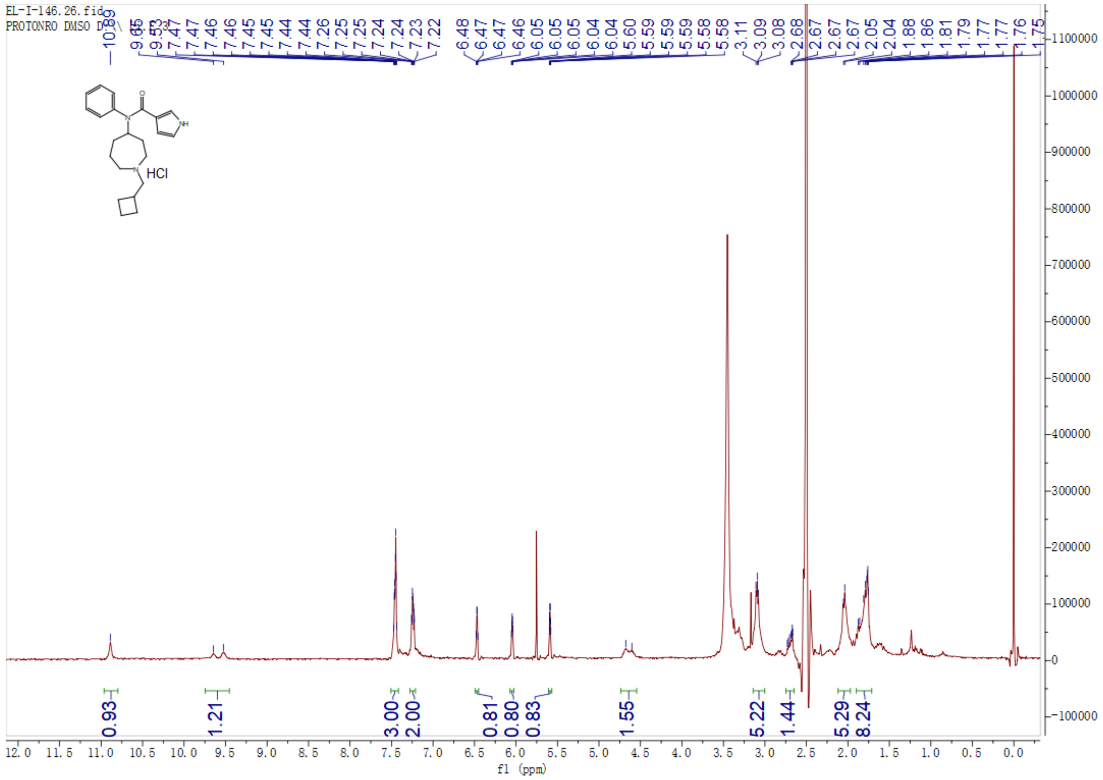

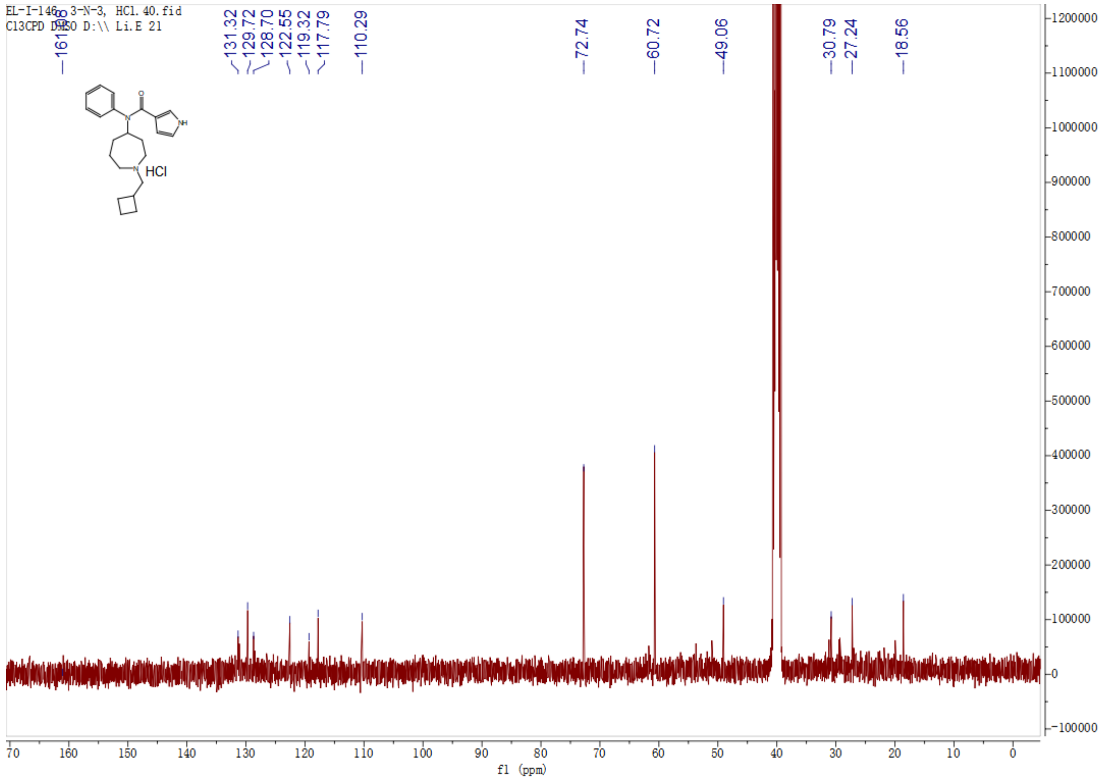

N-(1-(cyclopentylmethyl)azepan-4-yl)-N-phenyl-1H-pyrrole-3-carboxamide hydrochloride **(35)**

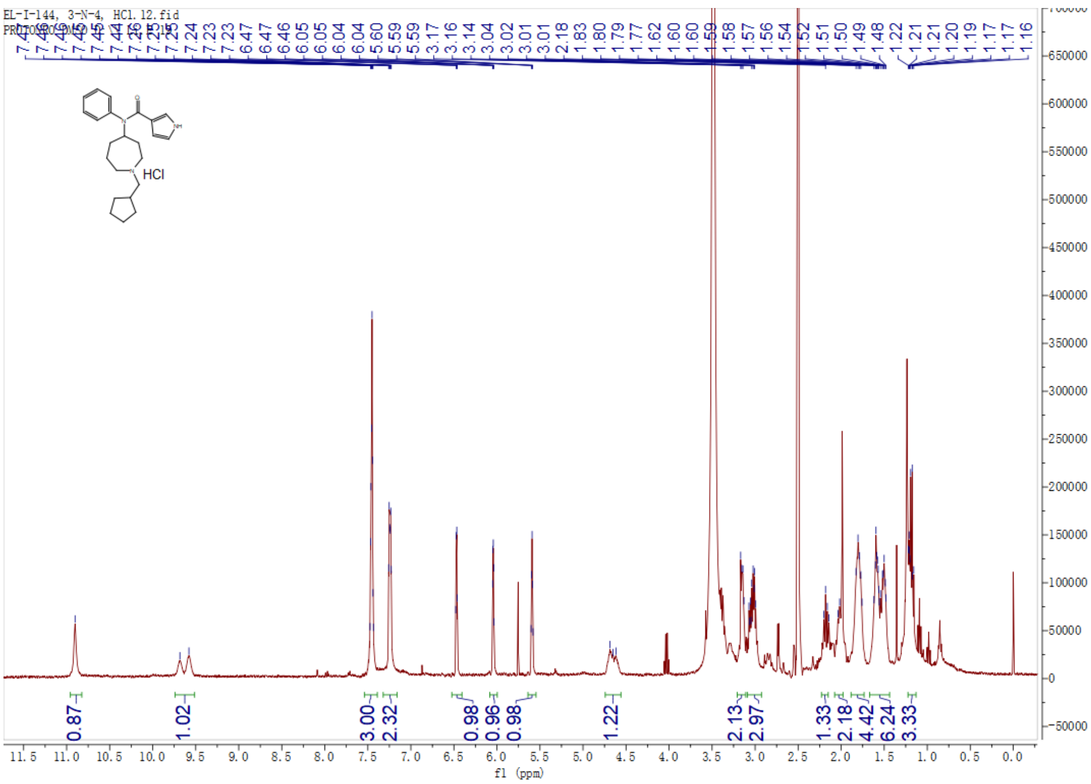

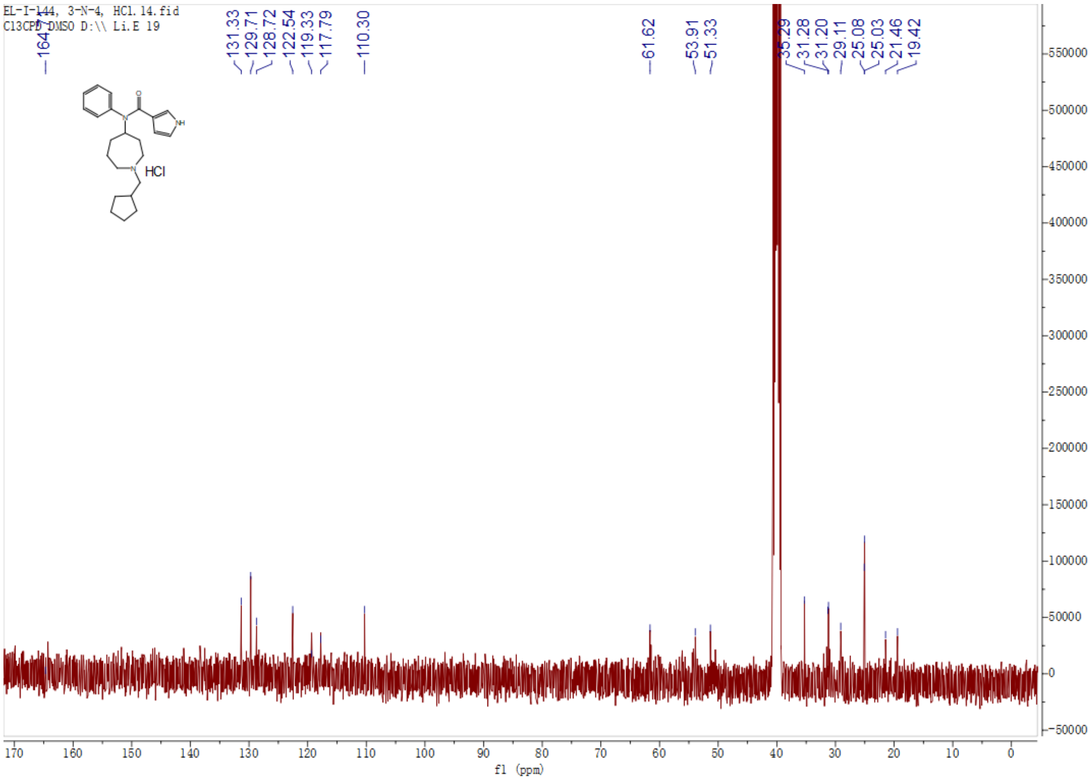

N-(1-(cyclohexylmethyl)azepan-4-yl)-N-phenyl-1H-pyrrole-3-carboxamide hydrochloride **(36)**

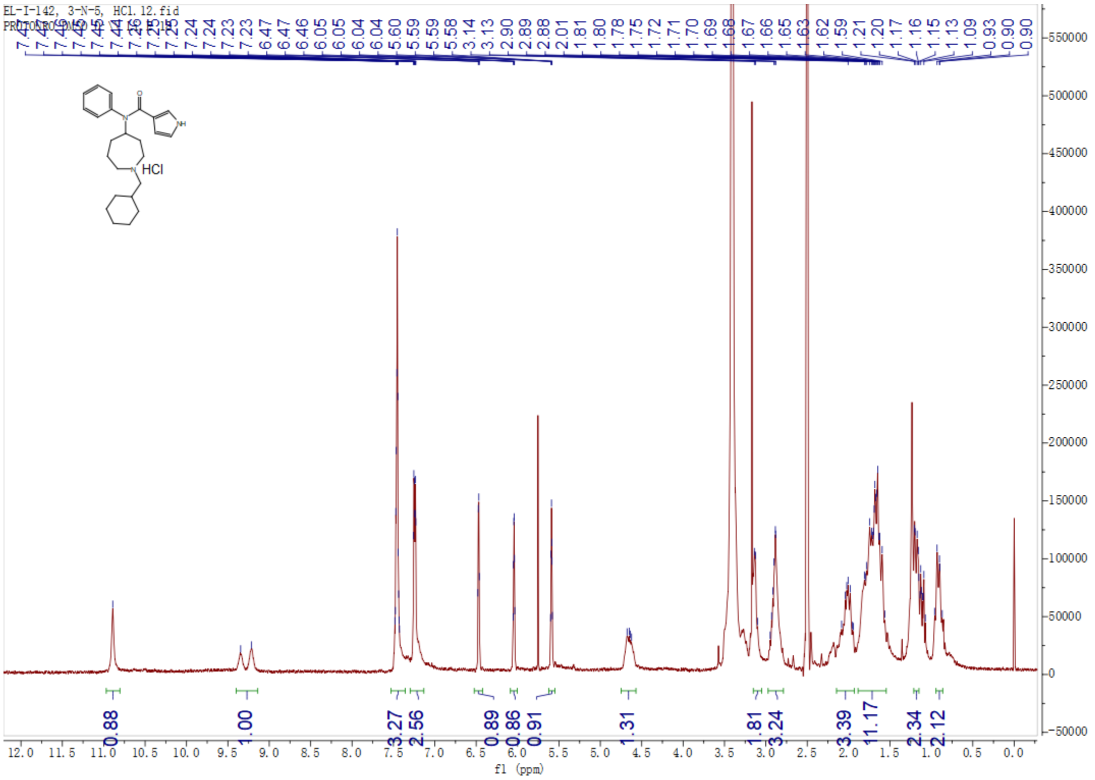

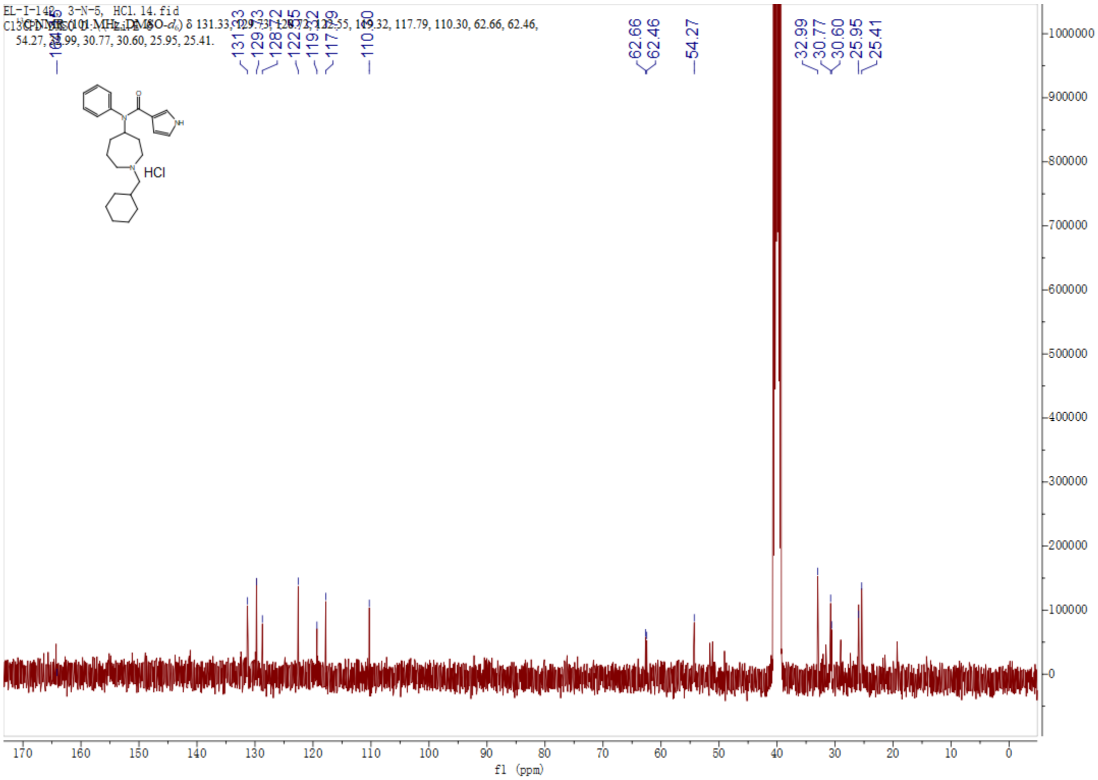

N-(1-benzylazepan-4-yl)-N-phenyl-1H-pyrrole-3-carboxamide hydrochloride **(37)**

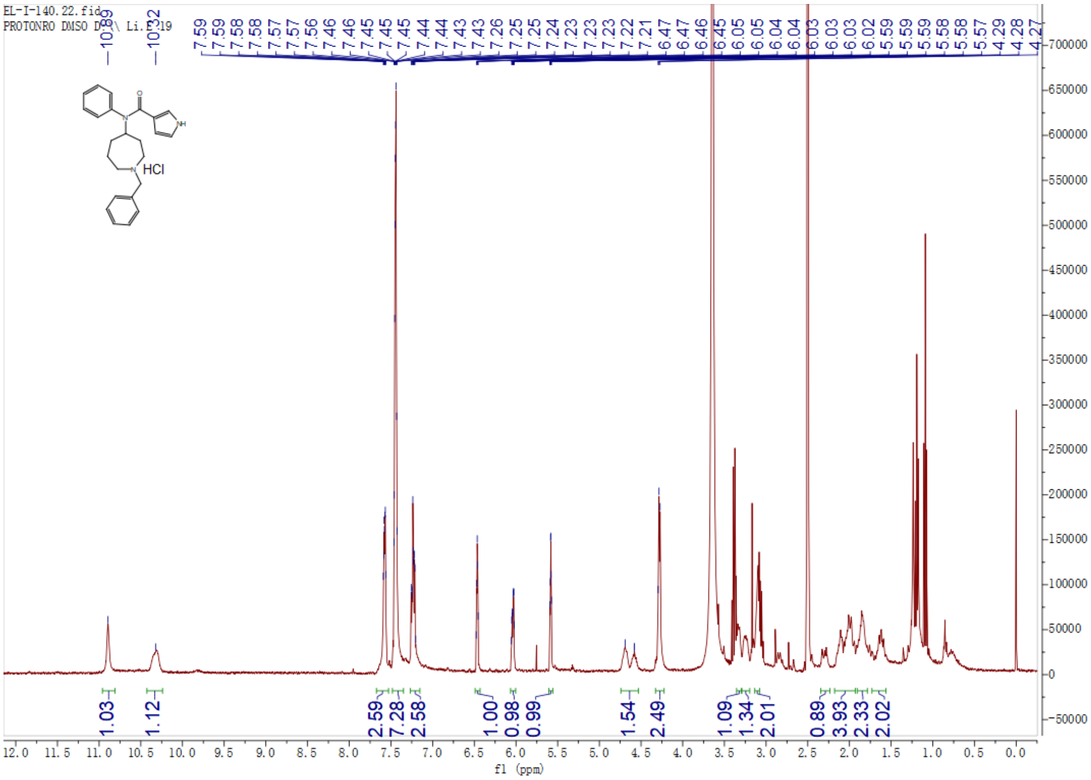

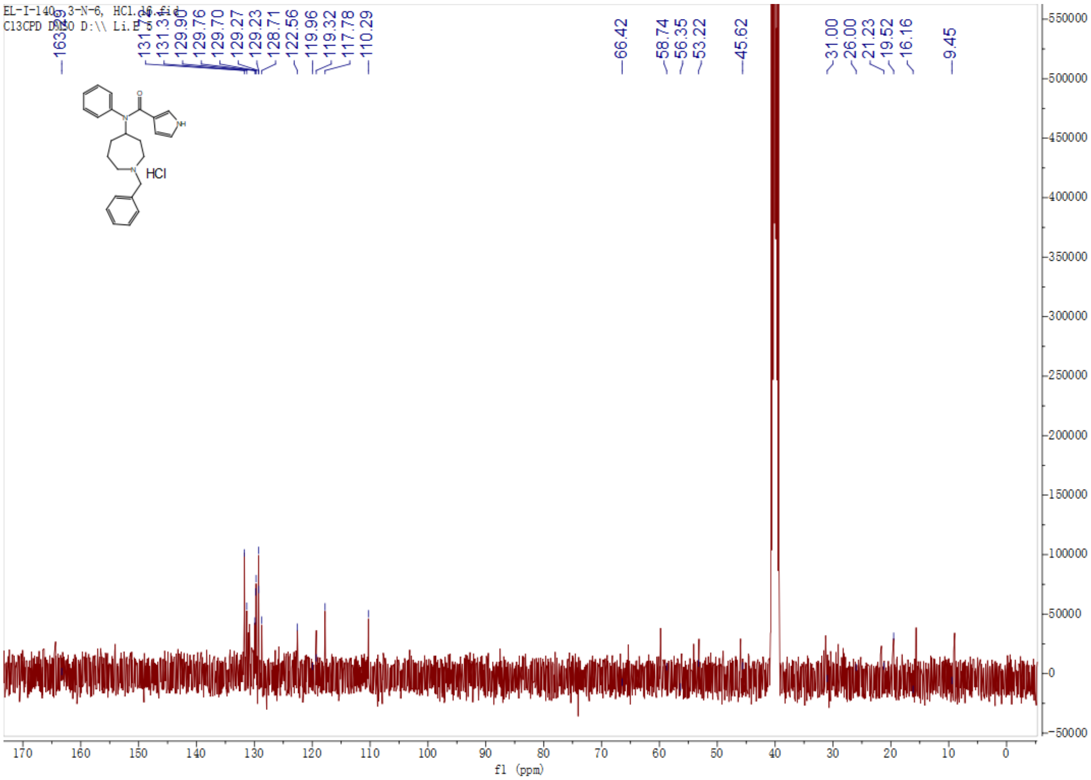

N-(1-phenethylazepan-4-yl)-N-phenyl-1H-pyrrole-3-carboxamide hydrochloride **(38)**

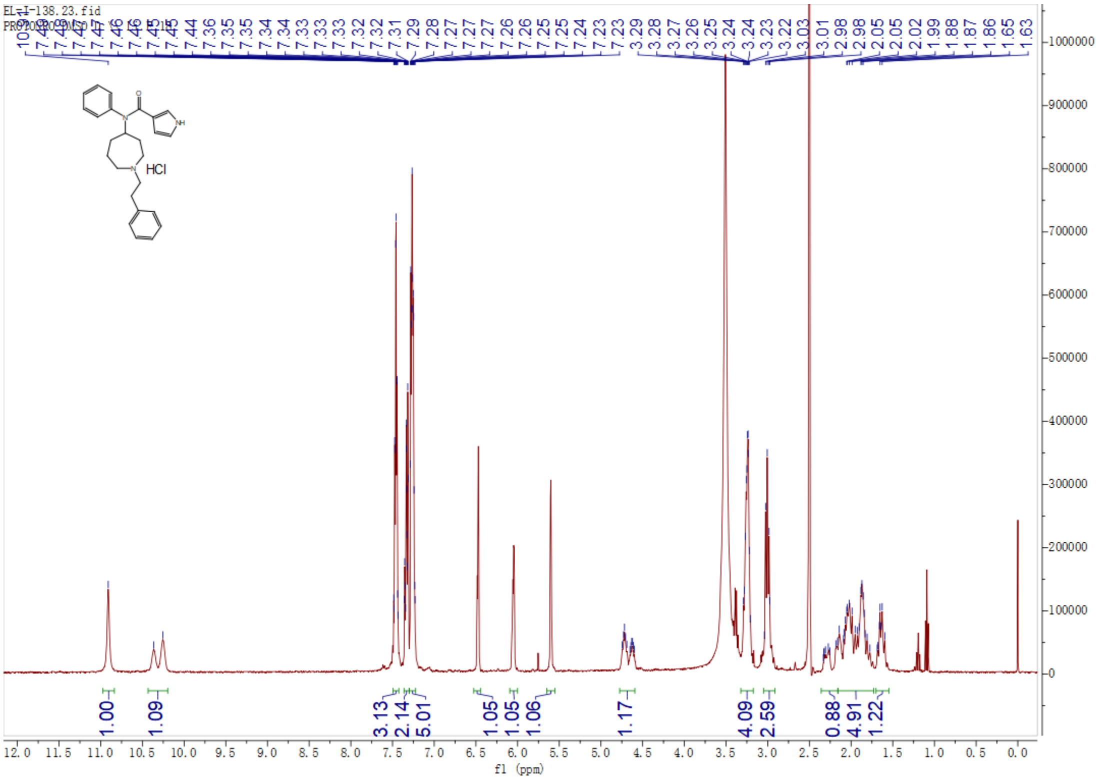

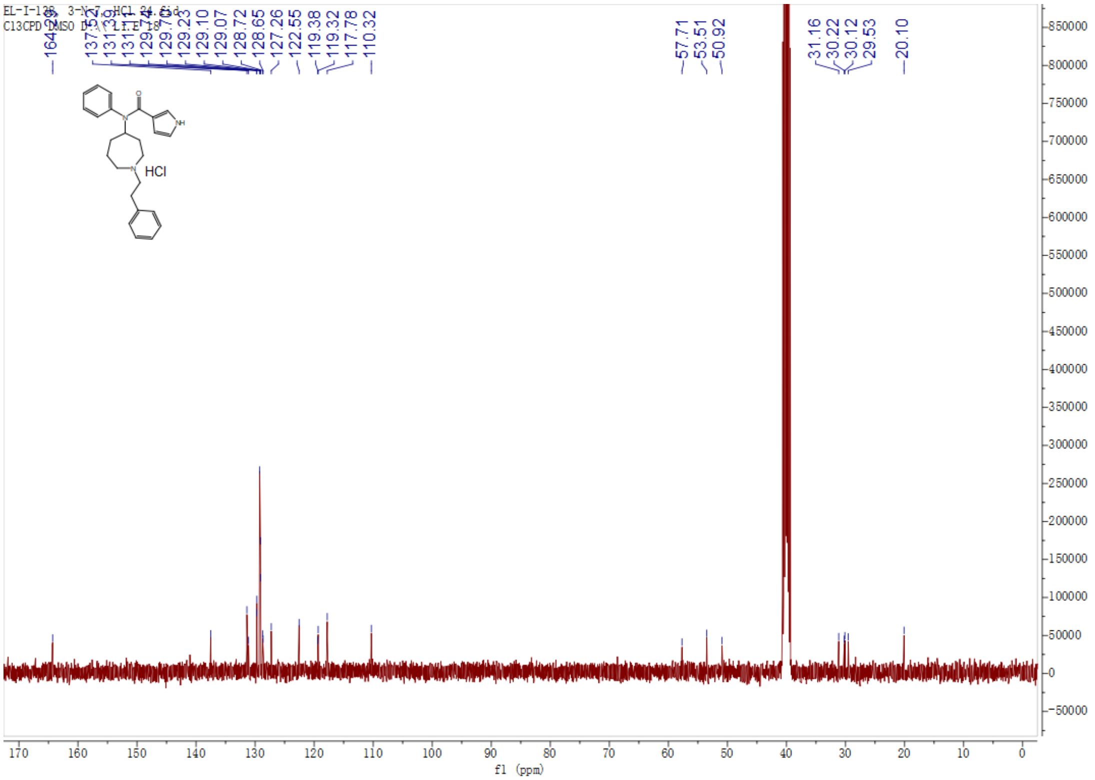

N-(1-allylazepan-4-yl)-N-phenylthiophene-3-carboxamide hydrochloride **(39)**

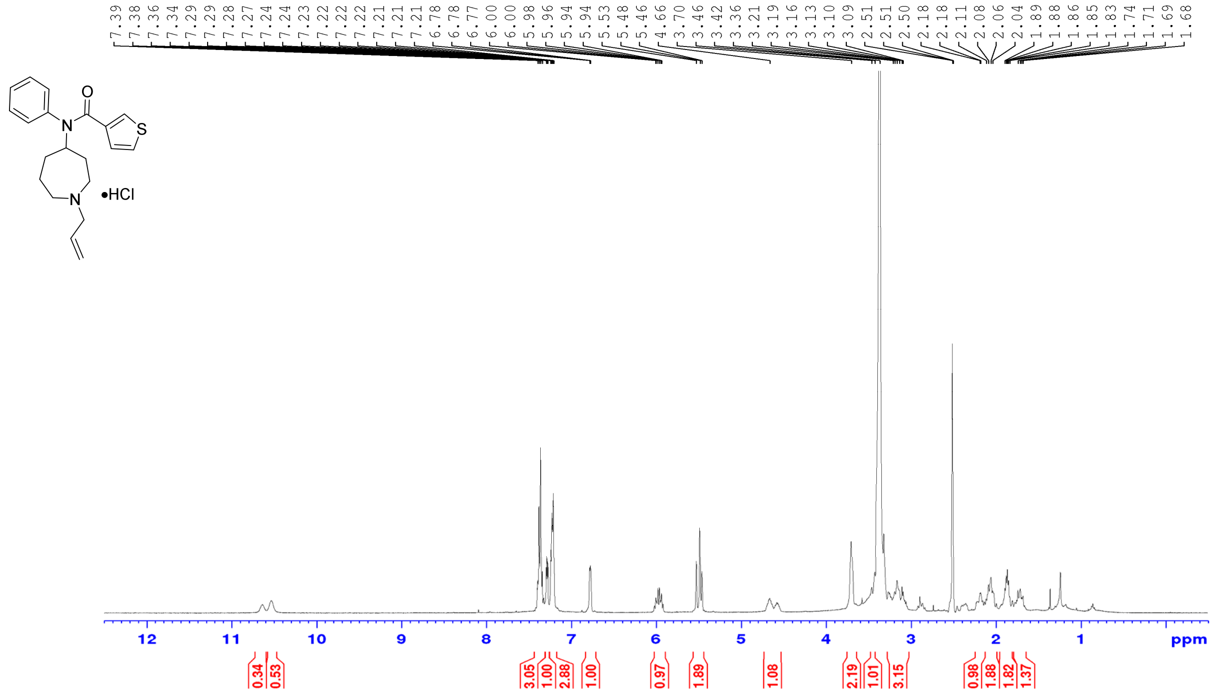

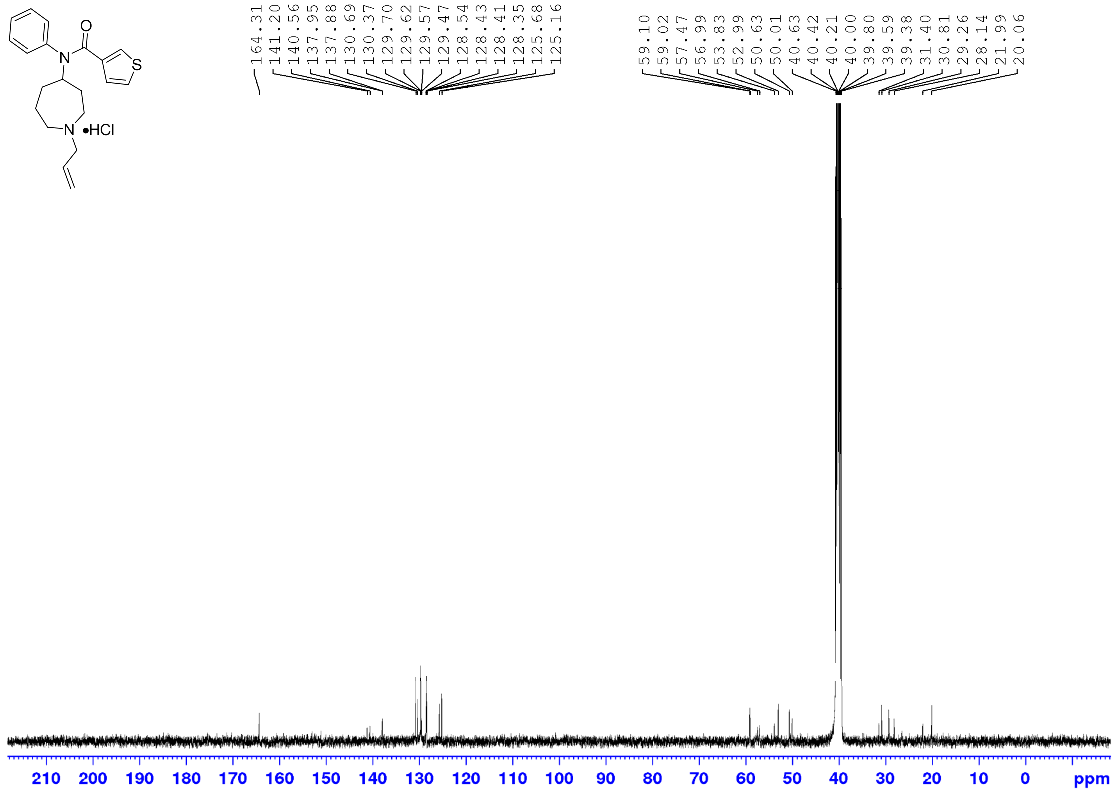

N-(1-(cyclopropylmethyl)azepan-4-yl)-N-phenylthiophene-3-carboxamide hydrochloride **(40)**

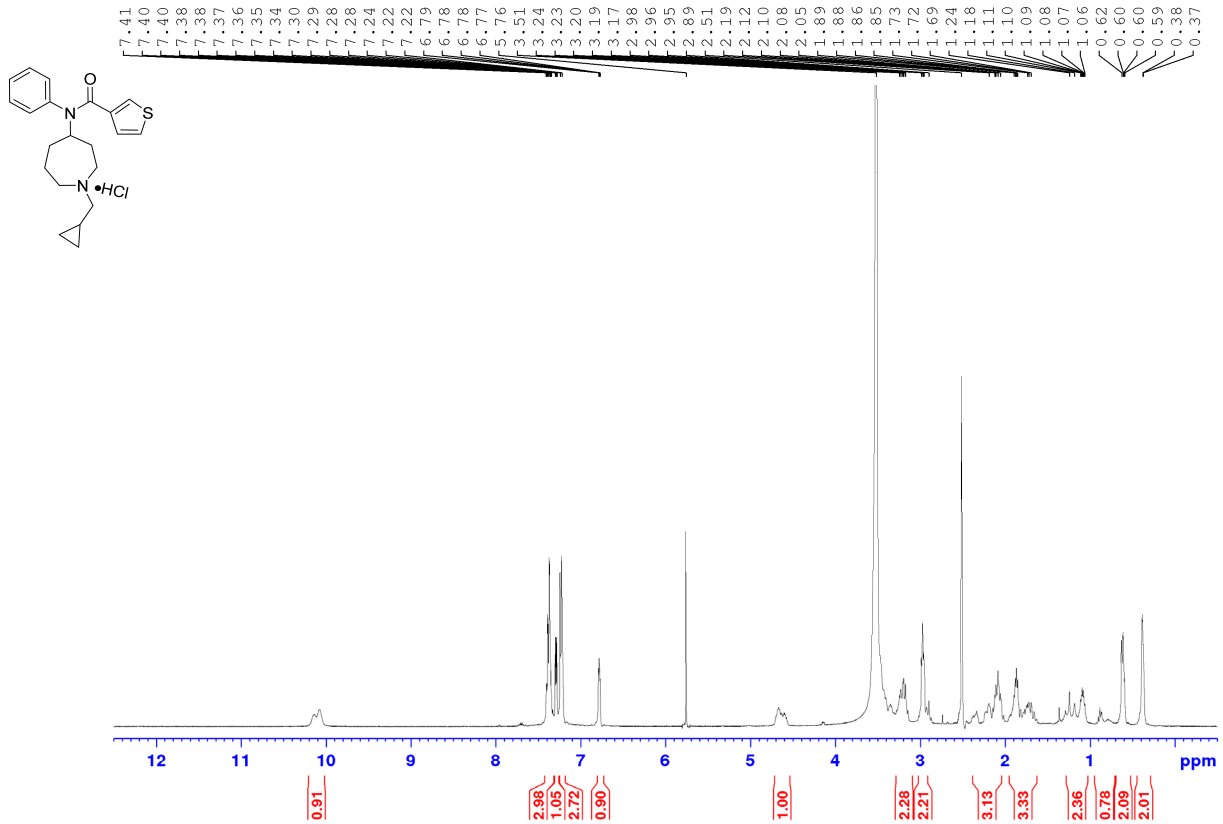

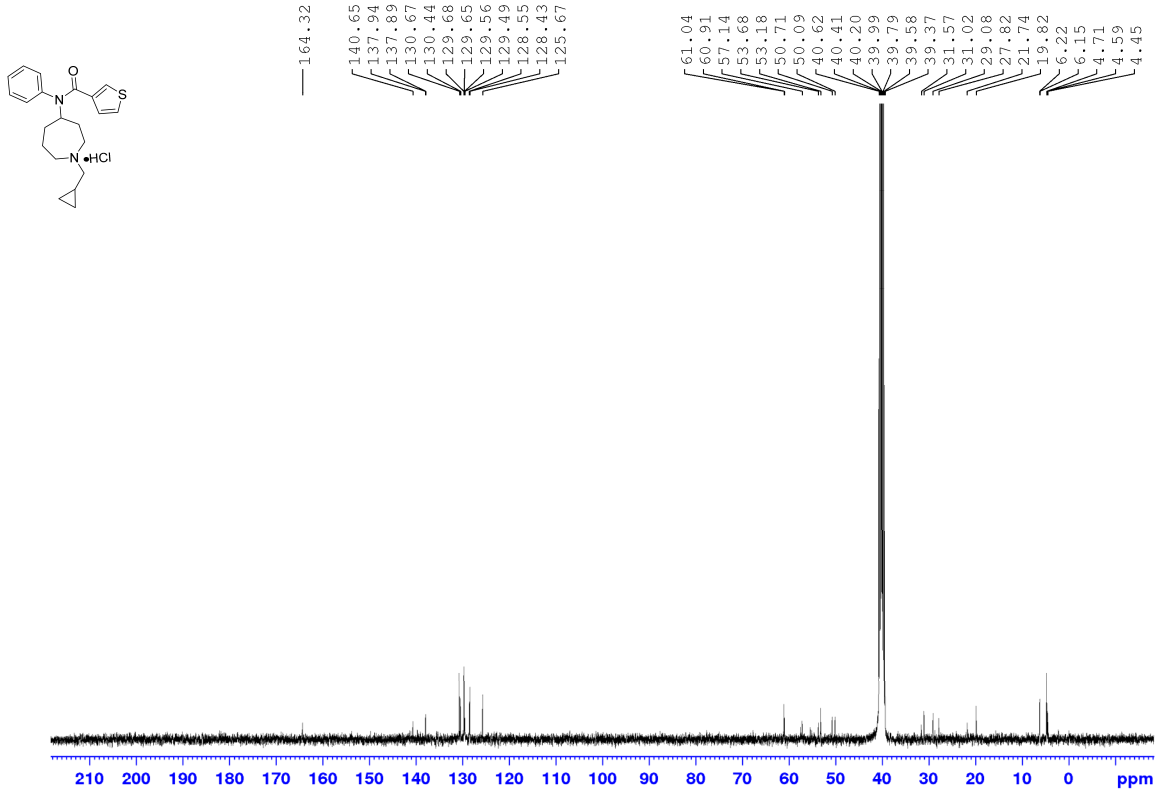

N-(1-(cyclobutylmethyl)azepan-4-yl)-N-phenylthiophene-3-carboxamide hydrochloride **(41)**

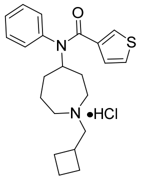

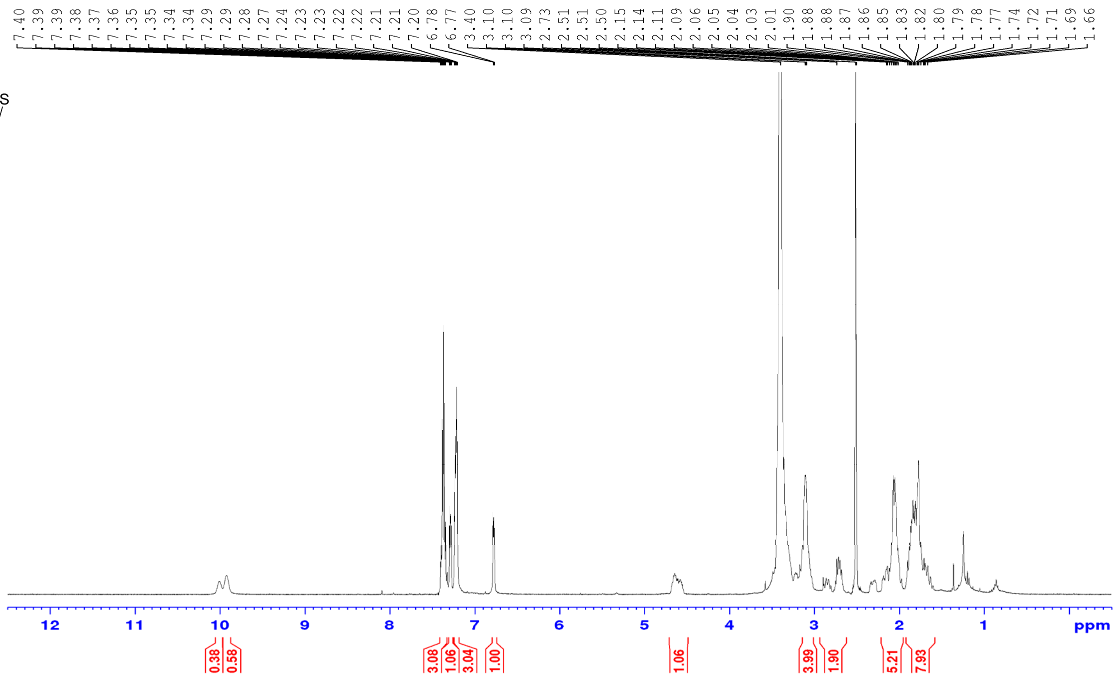

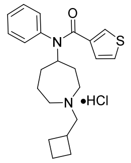

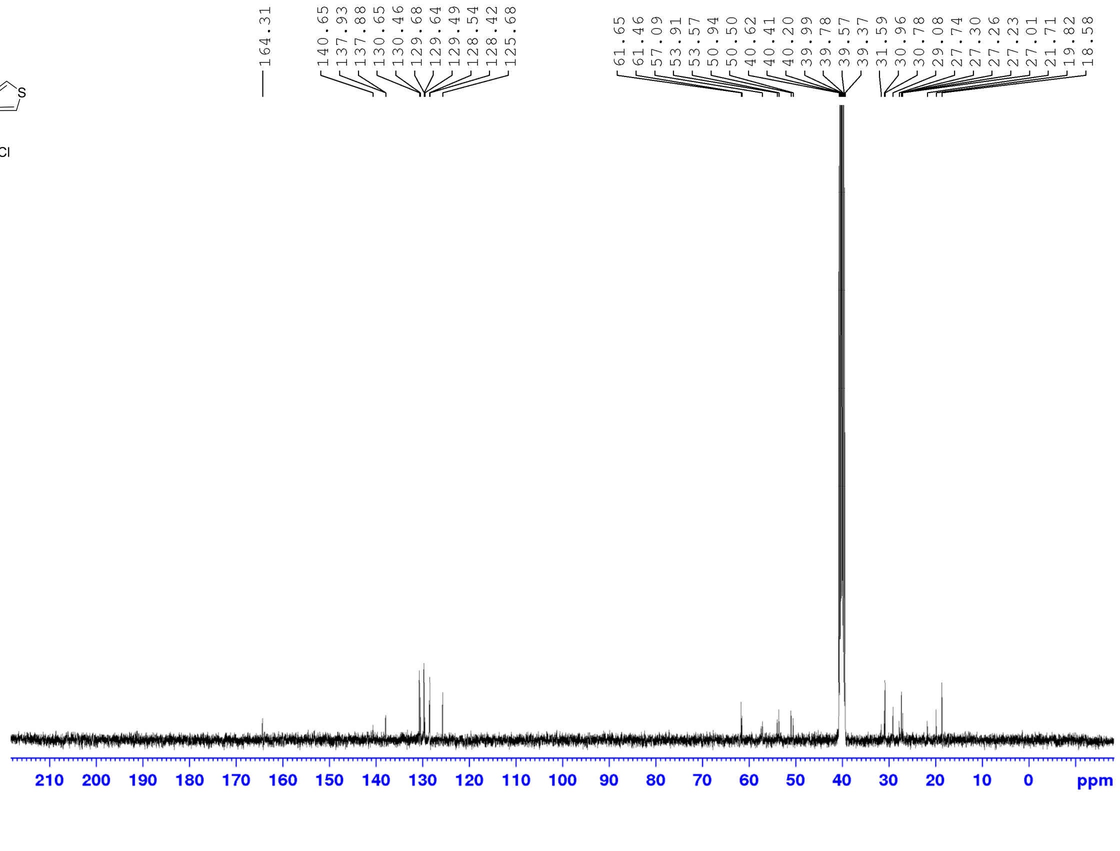

N-(1-(cyclopentylmethyl)azepan-4-yl)-N-phenylthiophene-3-carboxamide hydrochloride **(42)**

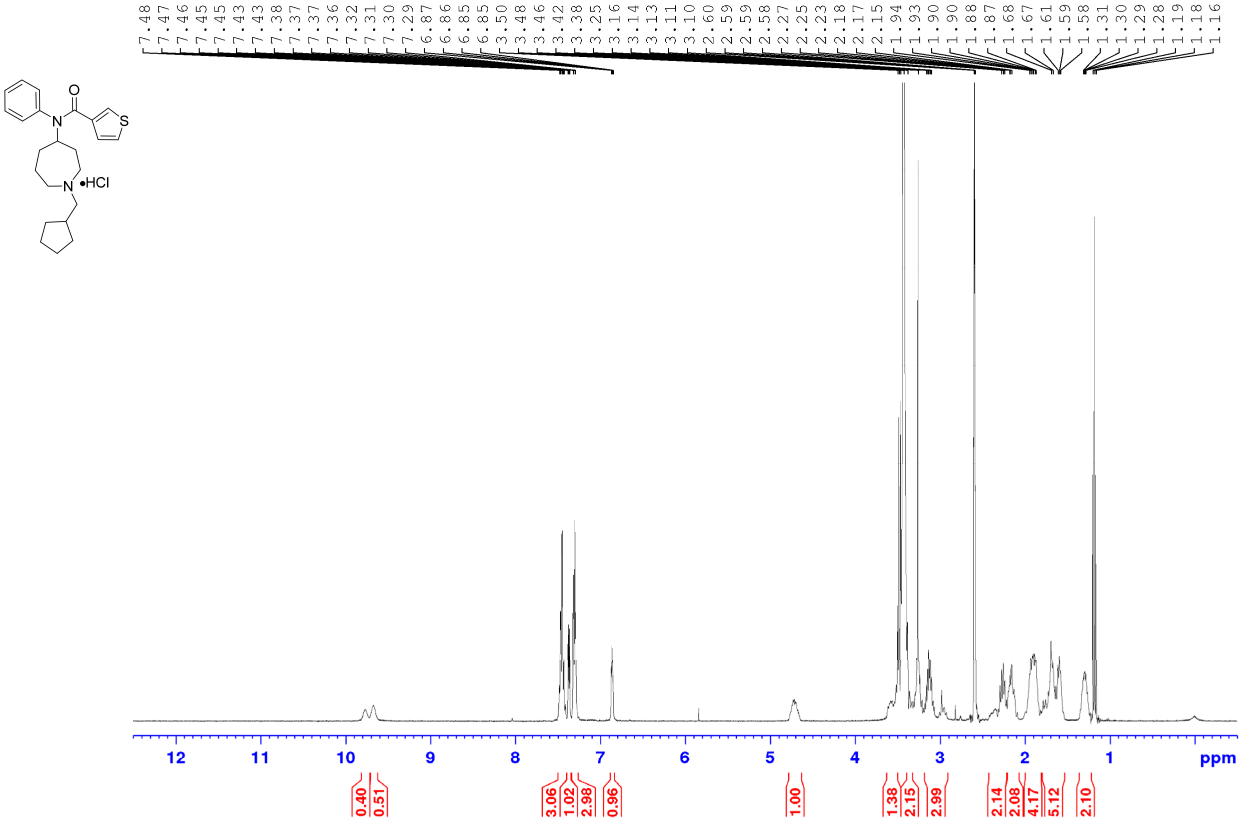

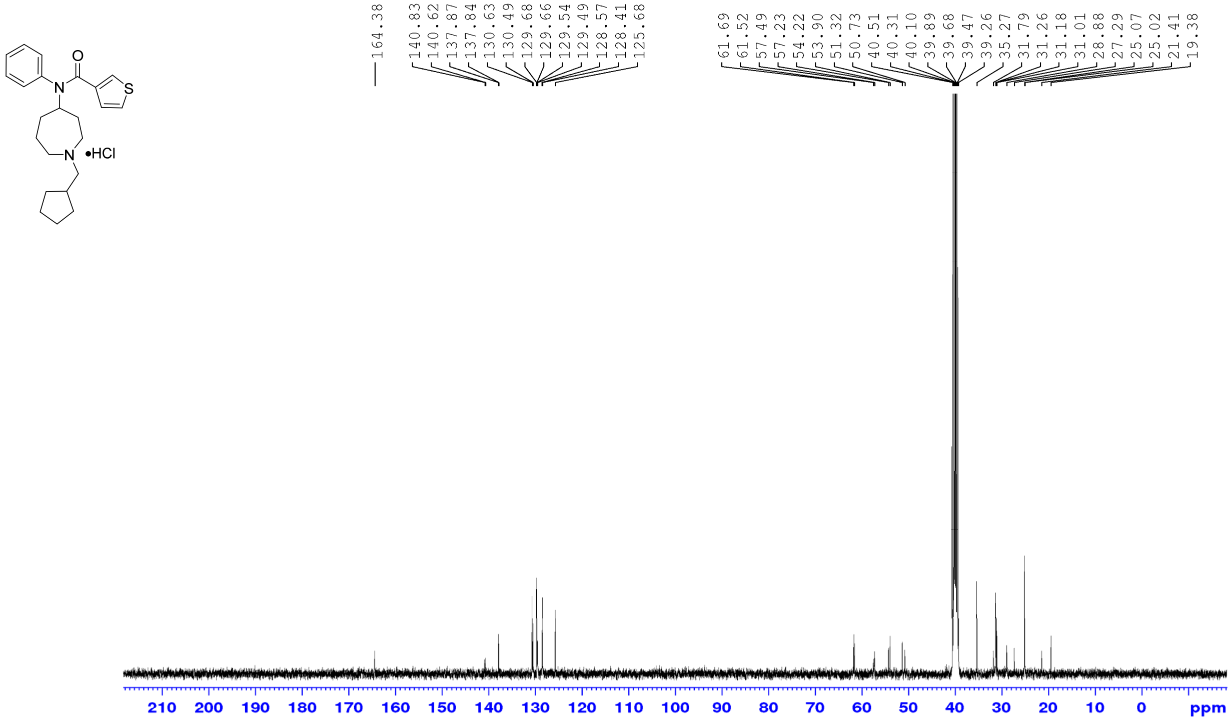

N-(1-(cyclohexylmethyl)azepan-4-yl)-N-phenylthiophene-3-carboxamide hydrochloride **(43)**

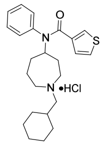

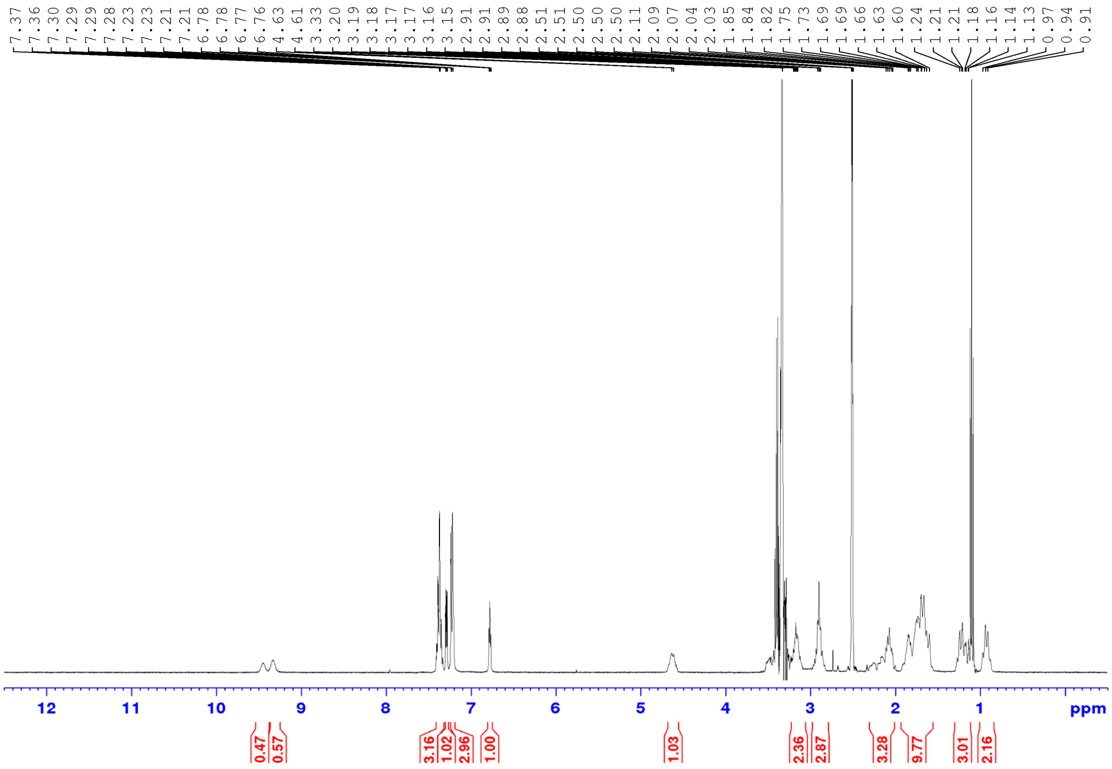

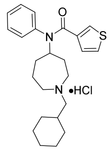

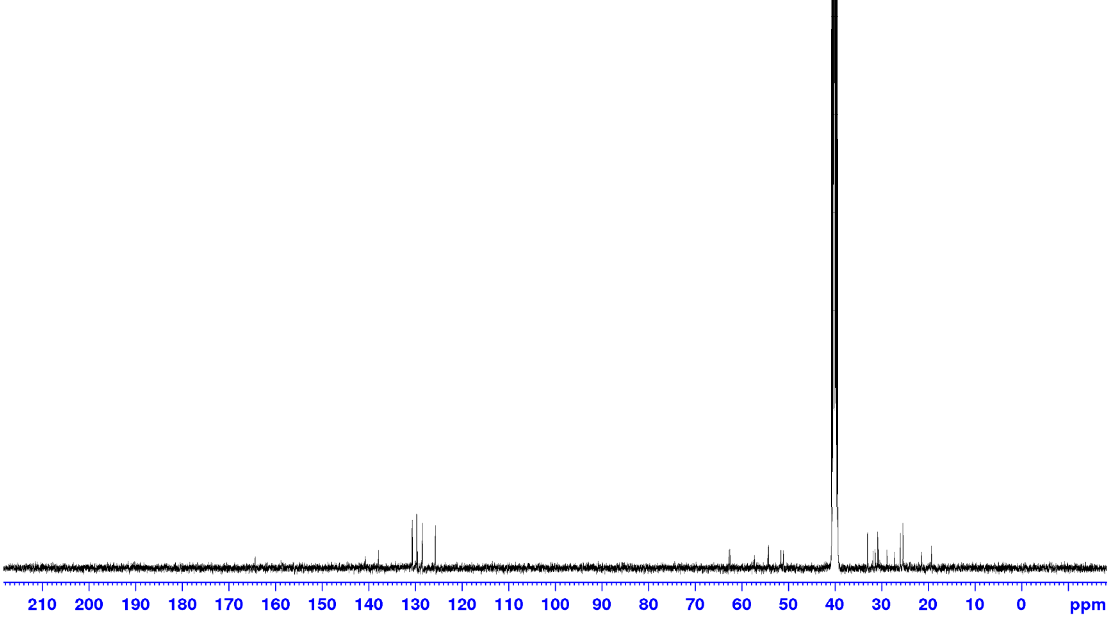

N-(1-benzylazepan-4-yl)-N-phenylthiophene-3-carboxamide hydrochloride **(44)**

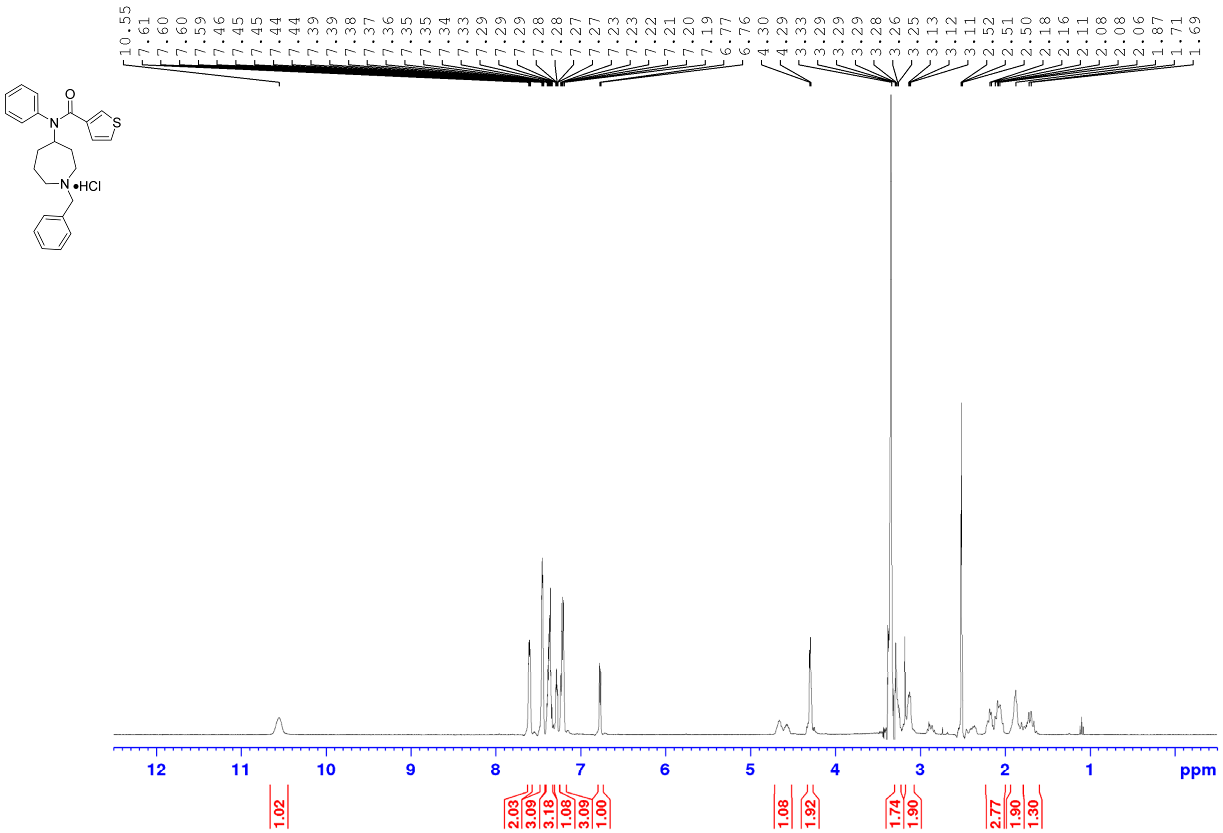

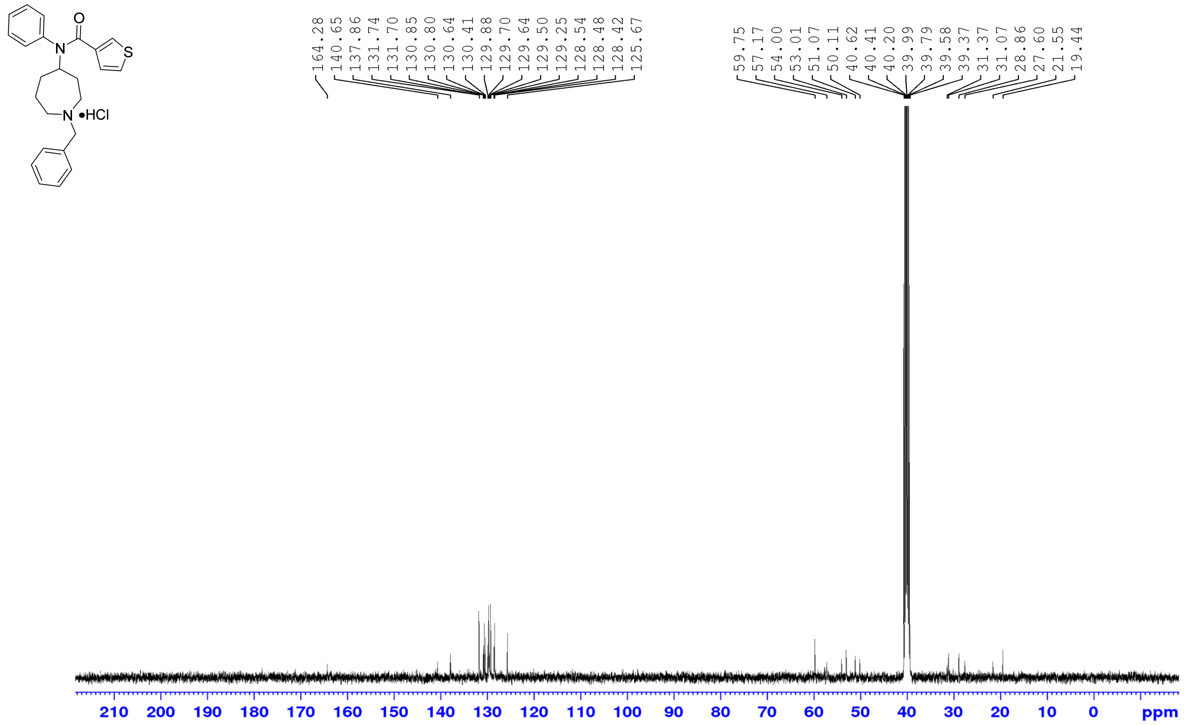

N-(1-phenethylazepan-4-yl)-N-phenylthiophene-3-carboxamide hydrochloride **(45)**

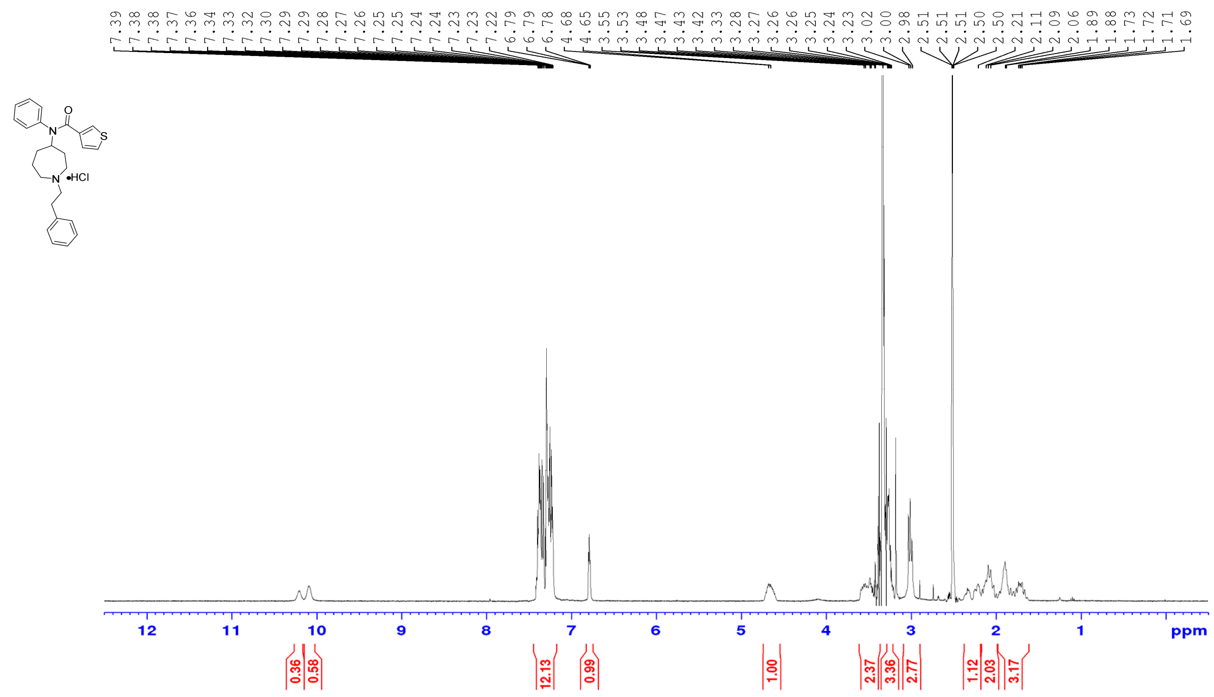

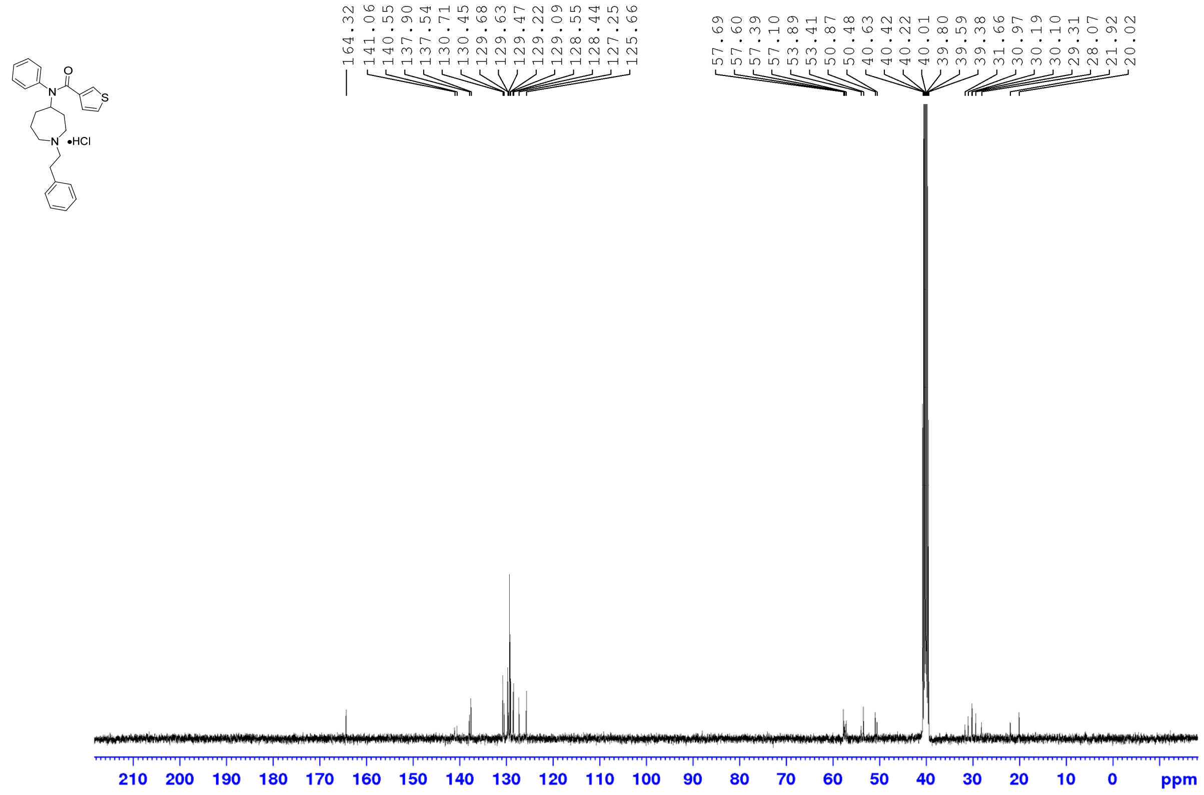

N-(1-allylazepan-4-yl)-N-phenylfuran-2-carboxamide hydrogen chloride **(46)**

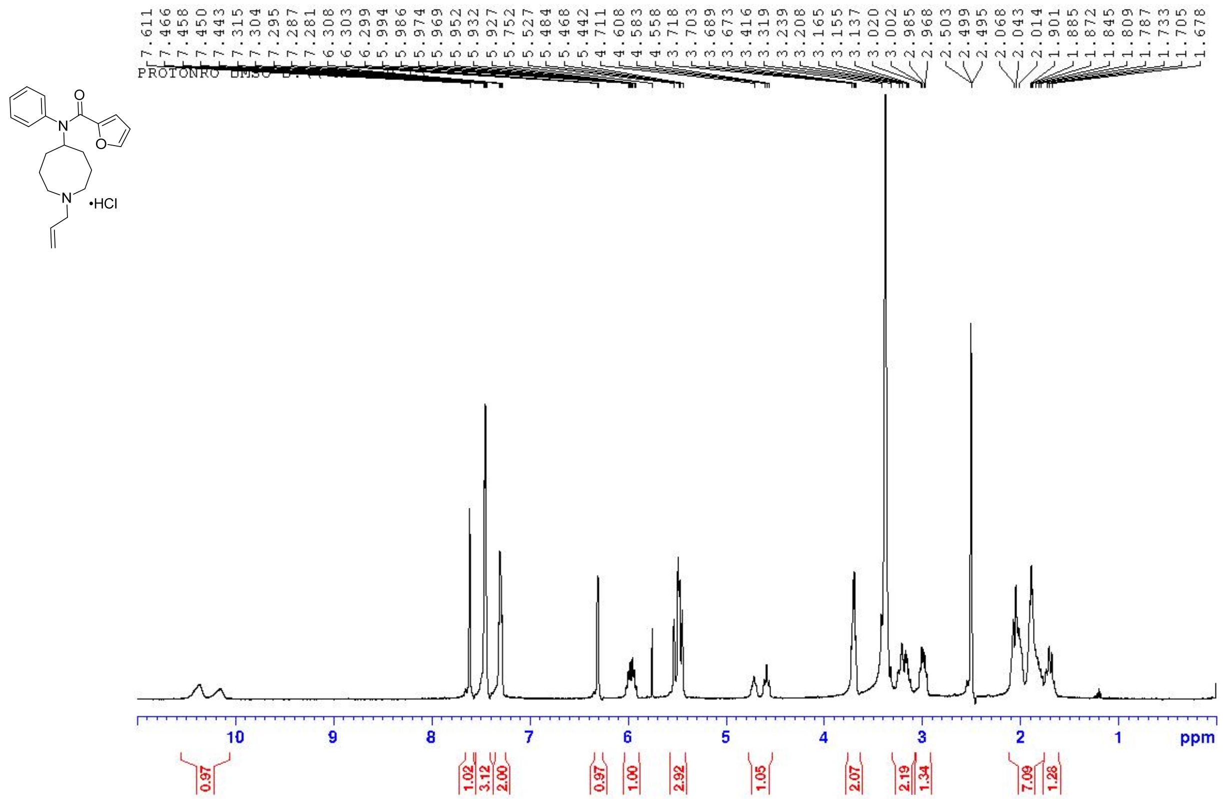

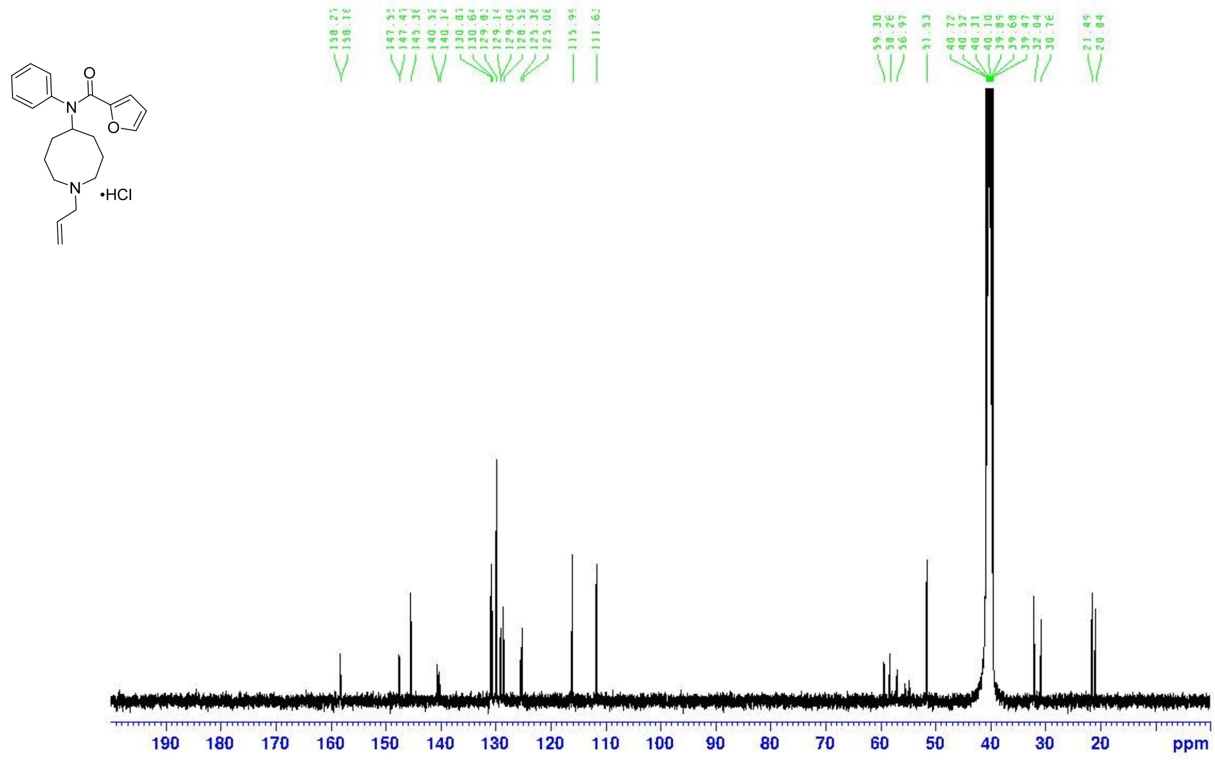

N-(1-(cyclopropylmethyl)azepan-4-yl)-N-phenylfuran-2-carboxamide hydrogen chloride (**47**)

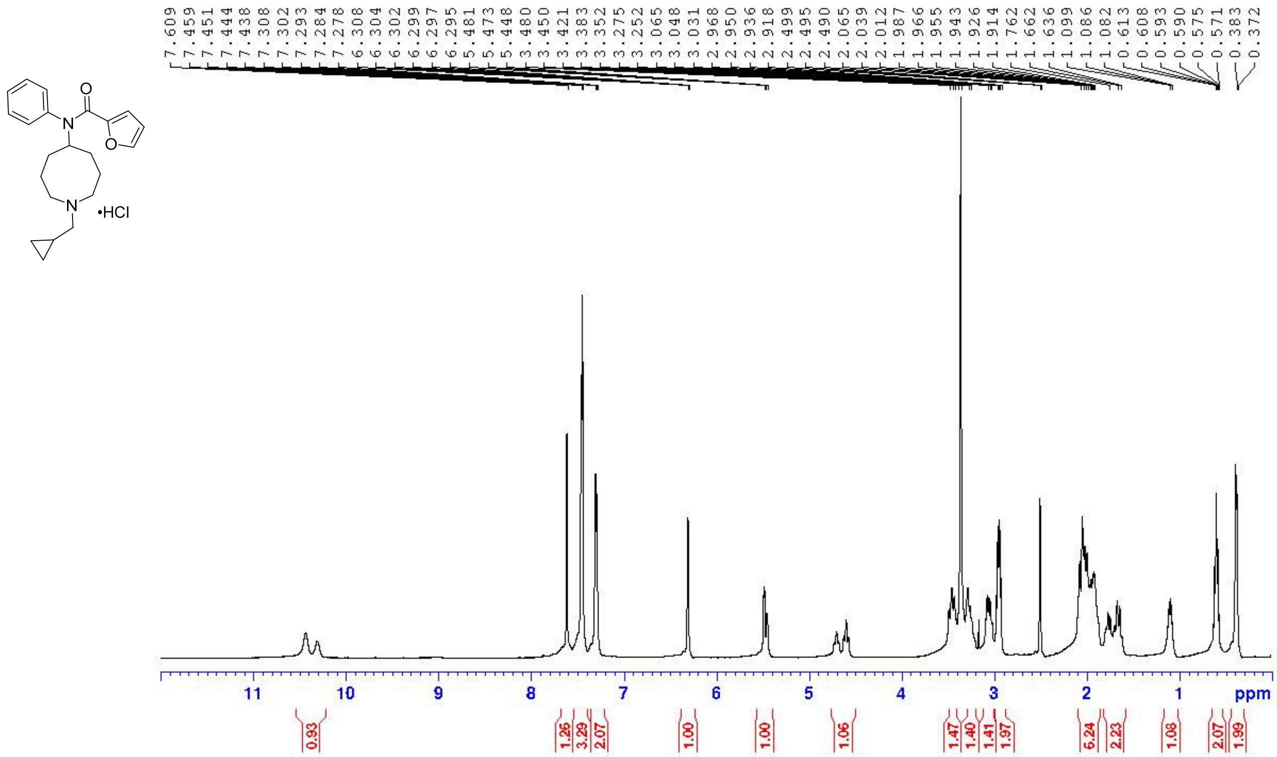

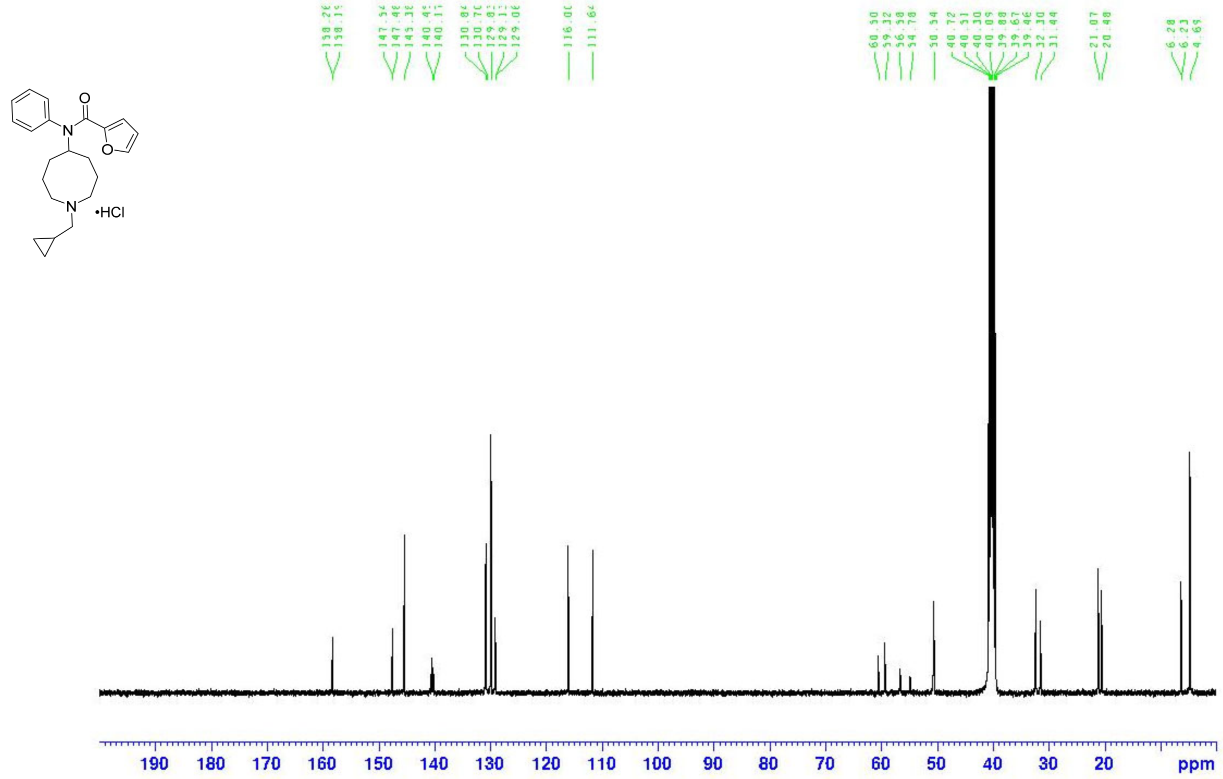

N-(1-(cyclobutylmethyl)azepan-4-yl)-N-phenylfuran-2-carboxamide hydrogen chloride (**48)**

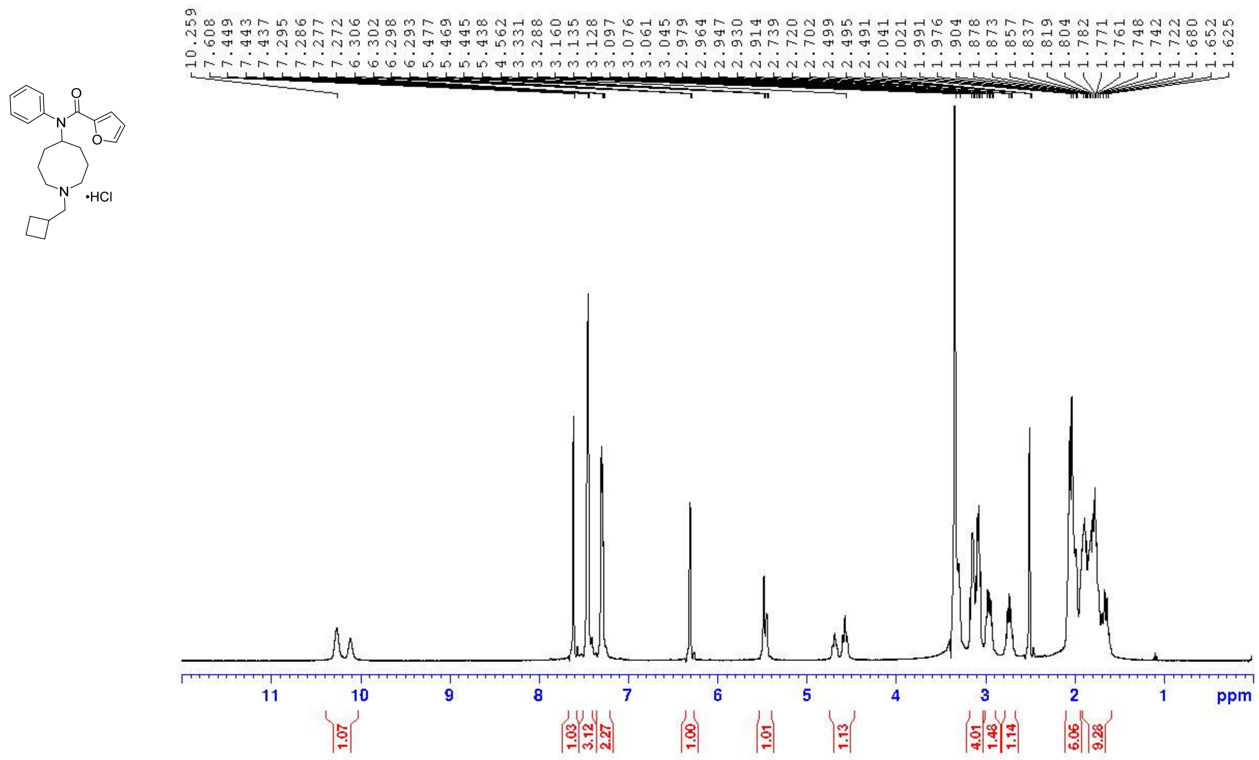

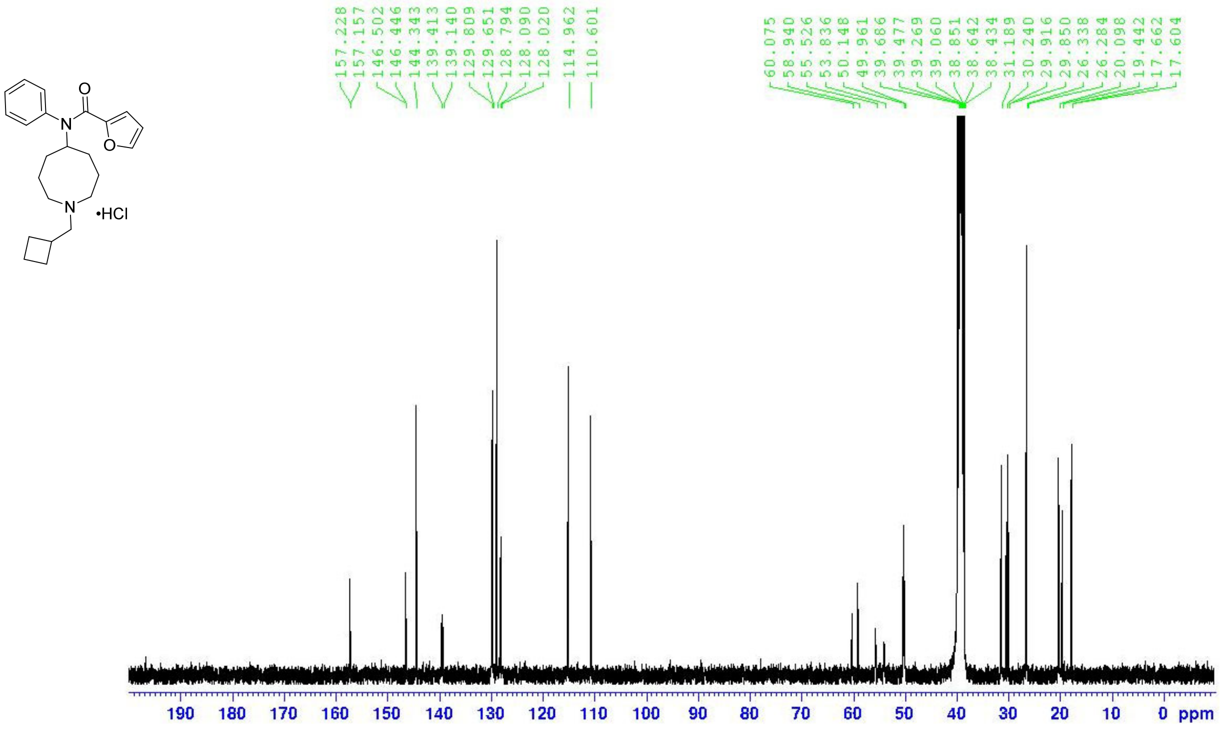

N-(1-(cyclopentylmethyl)azepan-4-yl)-N-phenylfuran-2-carboxamide hydrogen chloride **(49)**

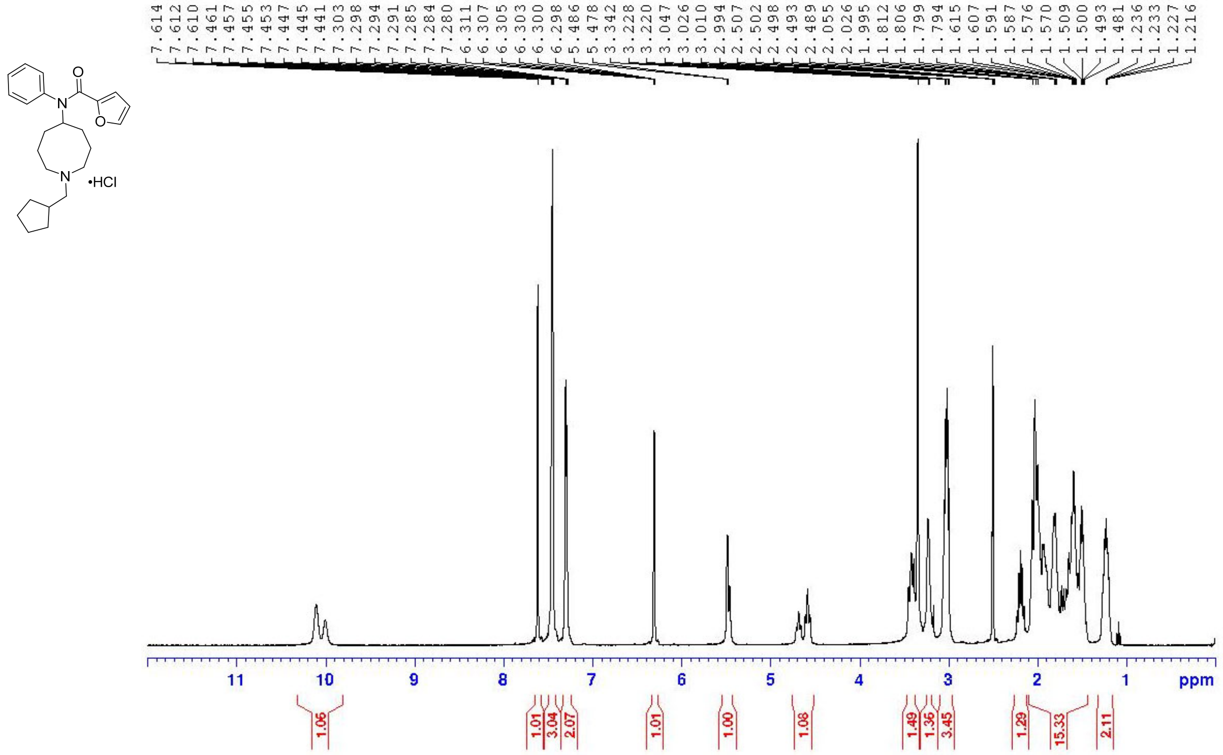

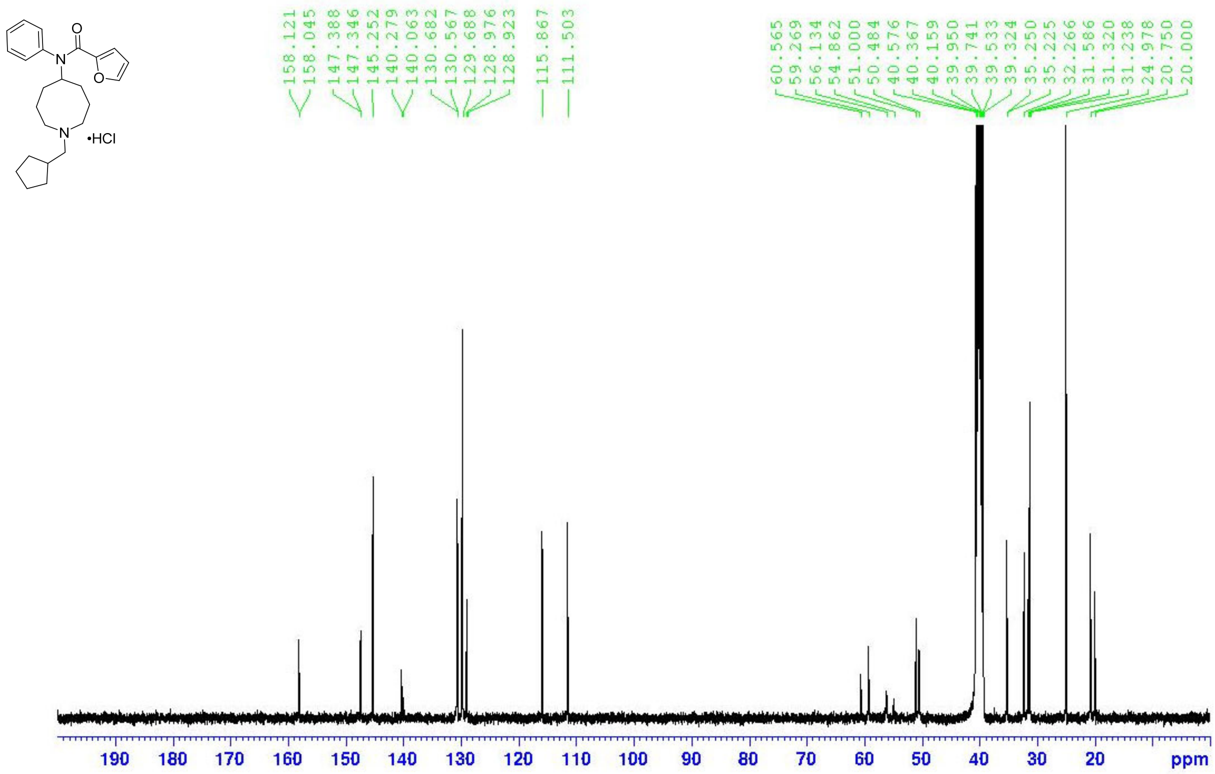

N-(1-(cyclohexylmethyl)azepan-4-yl)-N-phenylfuran-2-carboxamide hydrogen chloride **(50)**

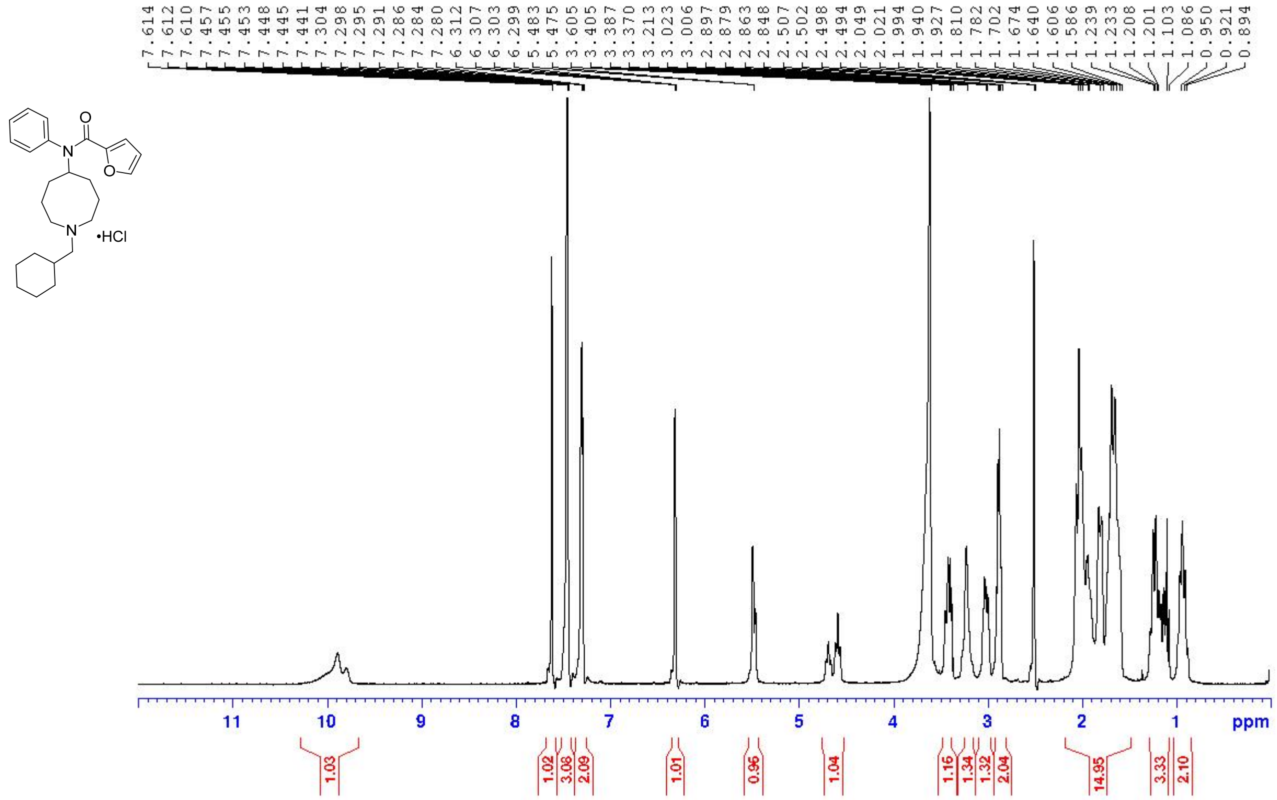

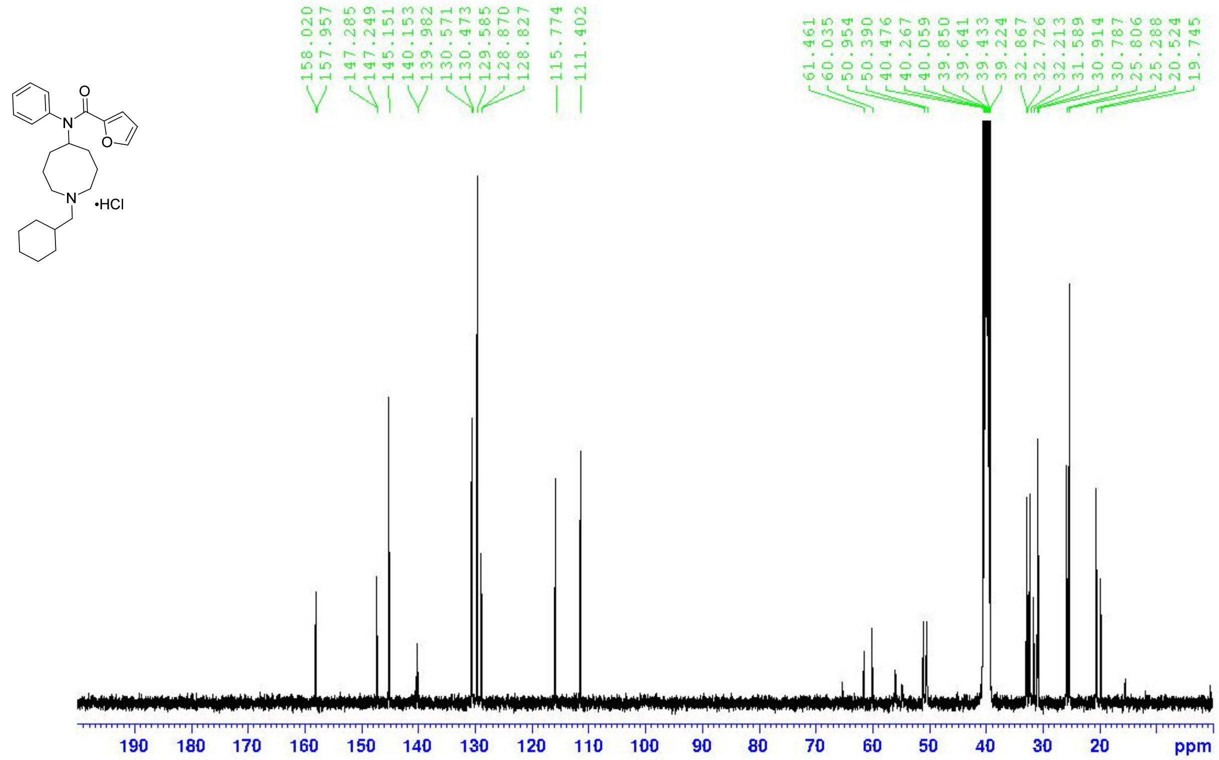

N-(1-benzylazepan-4-yl)-N-phenylfuran-2-carboxamide hydrogen chloride ((**51**)

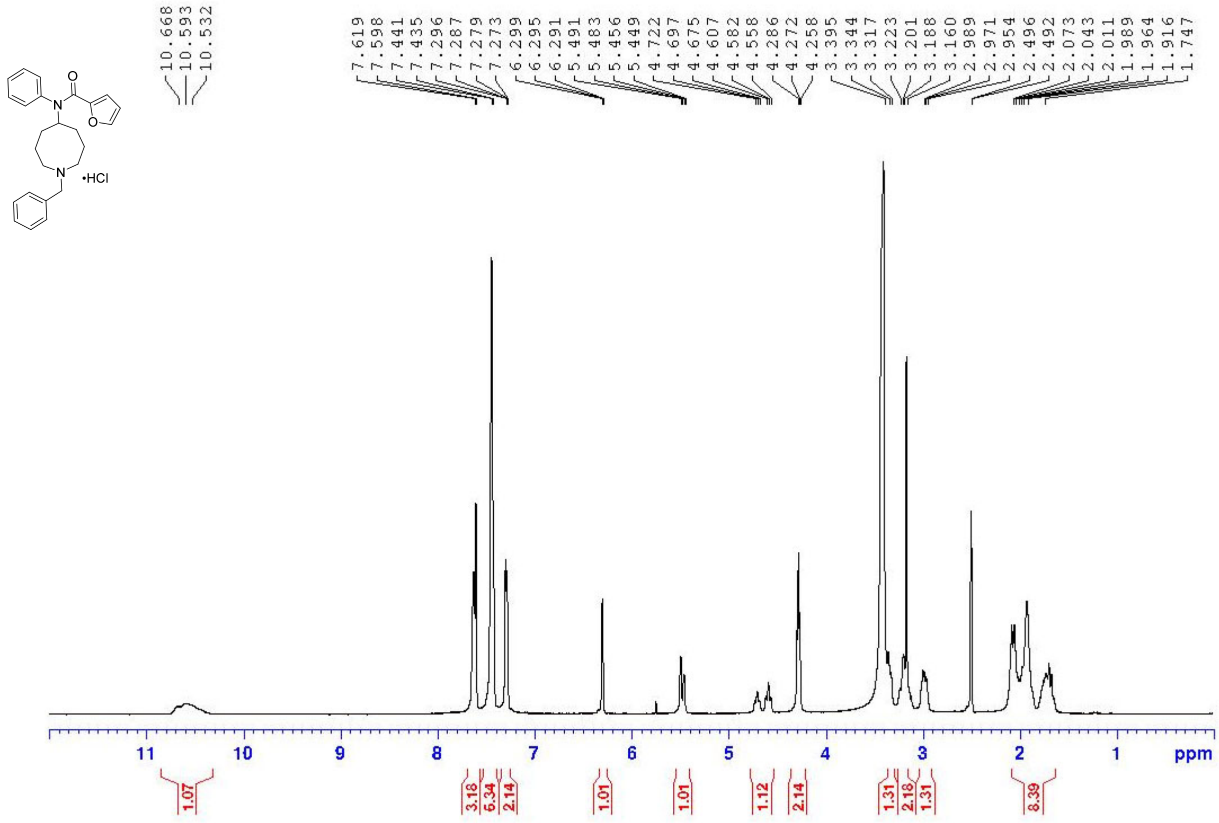

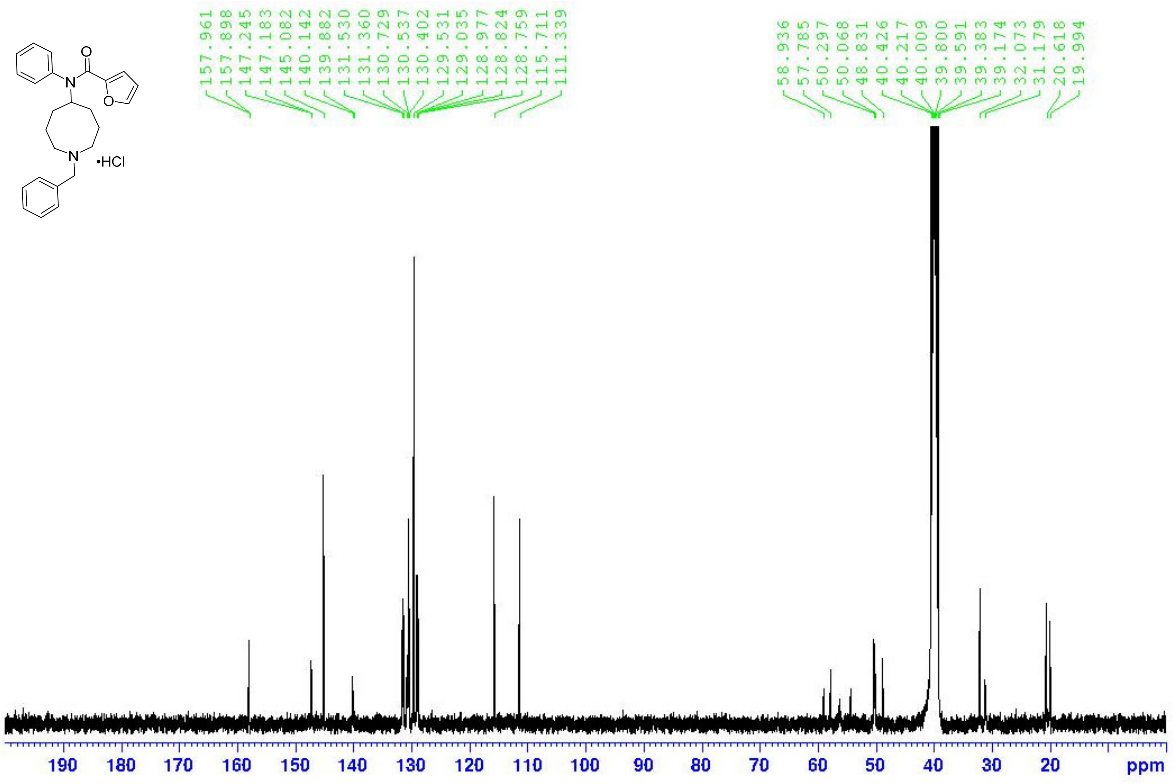

N-(1-phenethylazepan-4-yl)-N-phenylfuran-2-carboxamide hydrogen chloride (**52**)

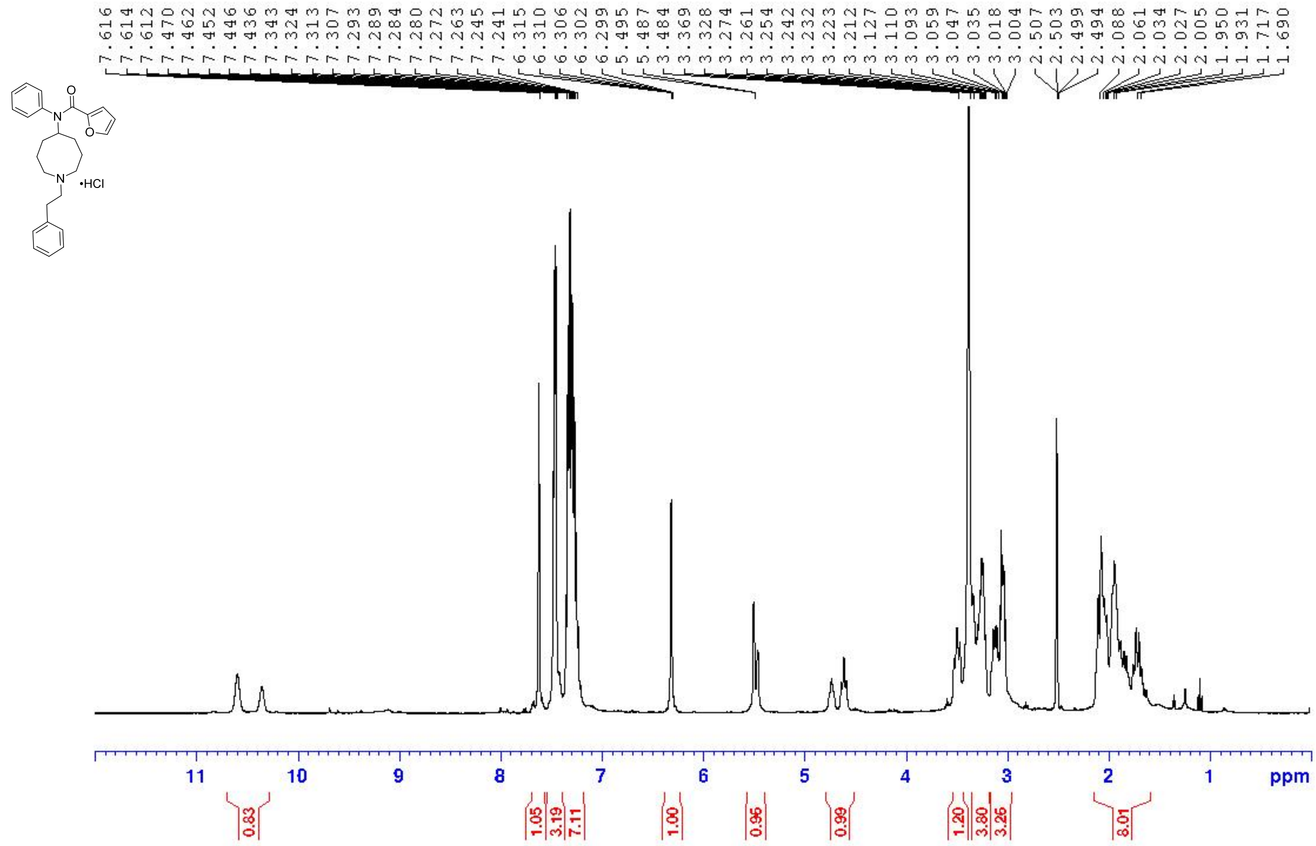

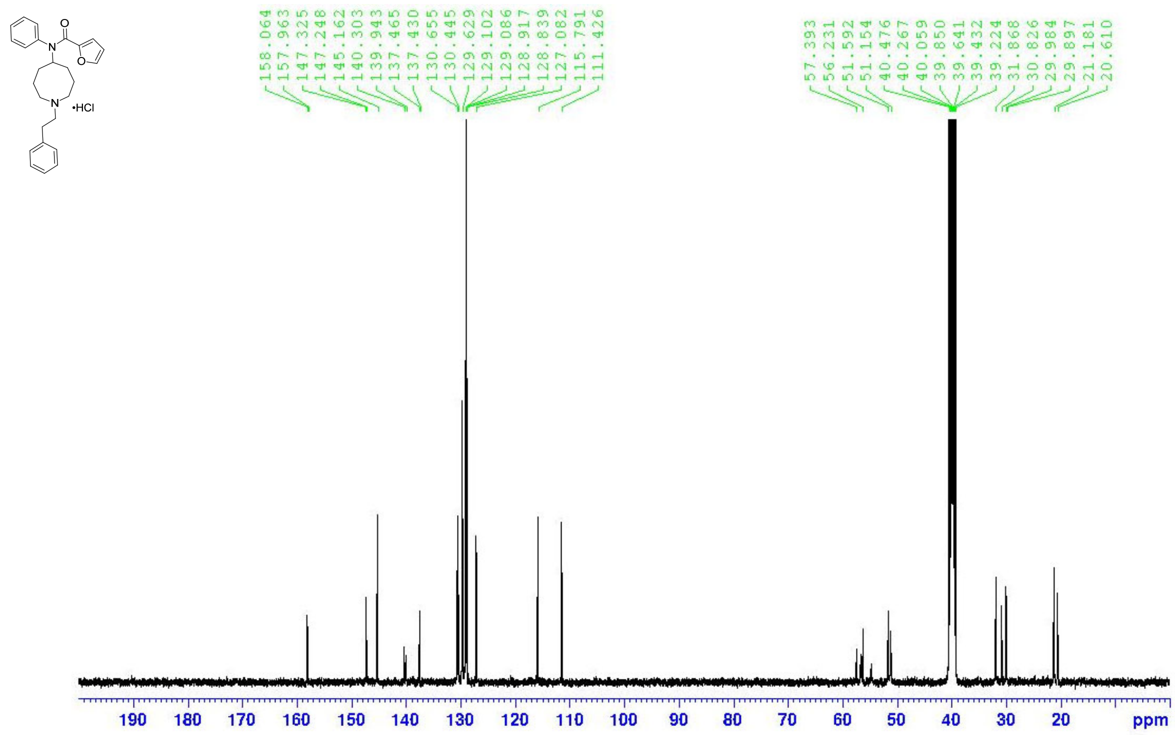

N-(1-allylazocan-5-yl)-N-phenylfuran-3-carboxamide hydrochloride **(53)**

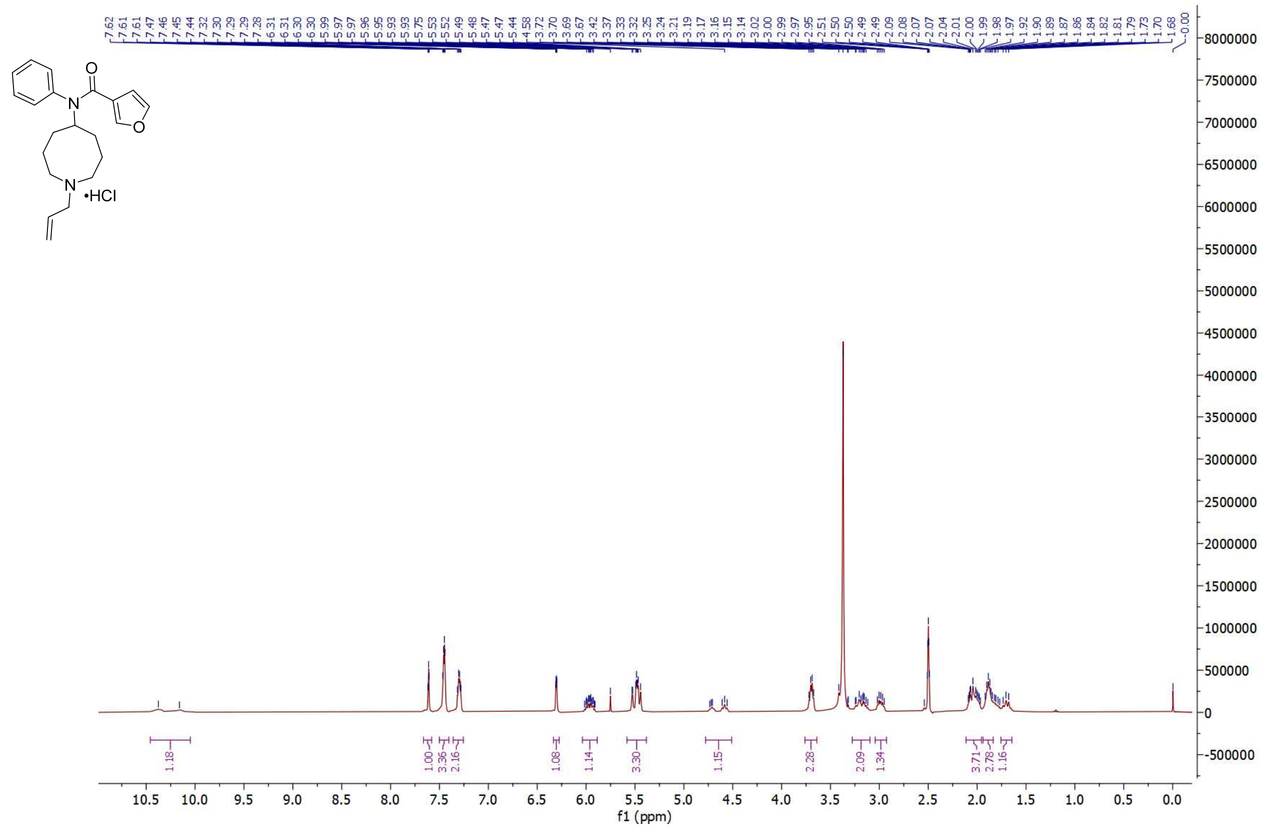

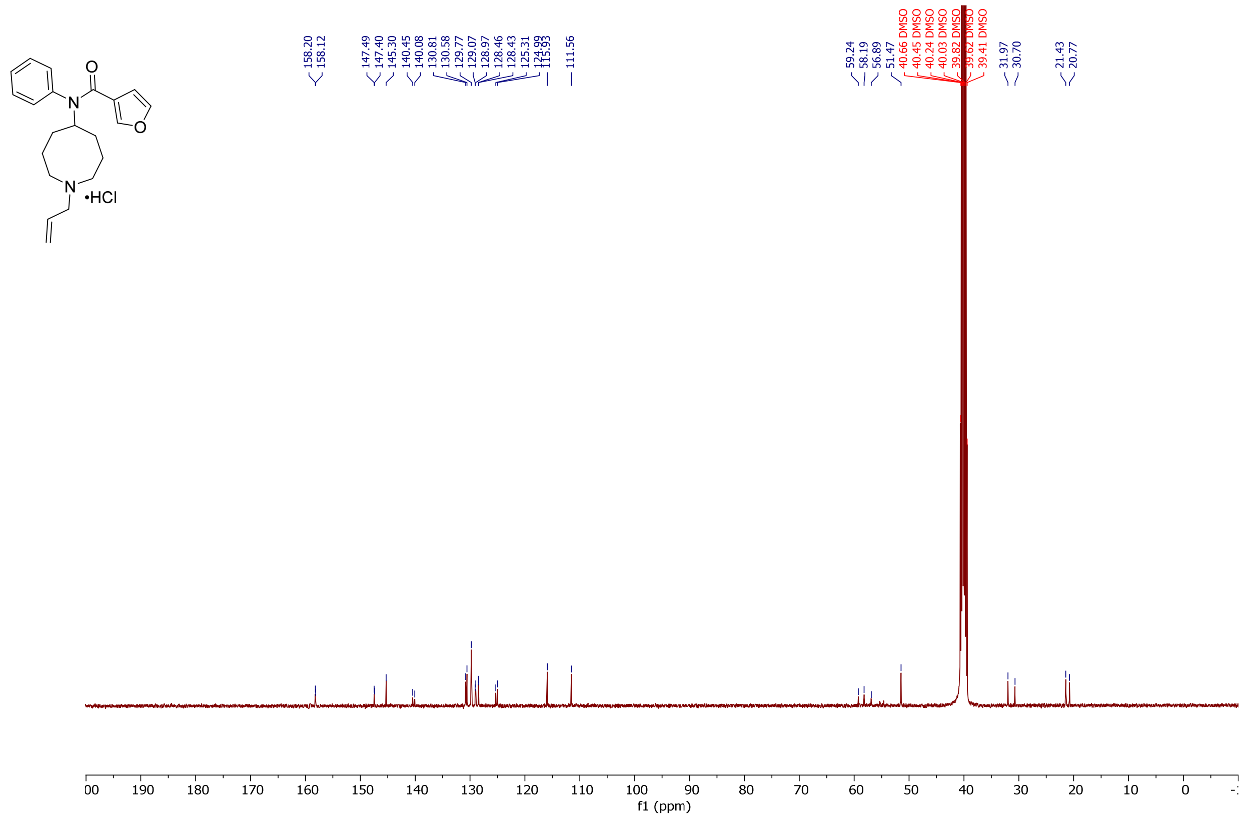

N-(1-(cyclopropylmethyl)azocan-5-yl)-N-phenylfuran-3-carboxamide hydrochloride **(54)**

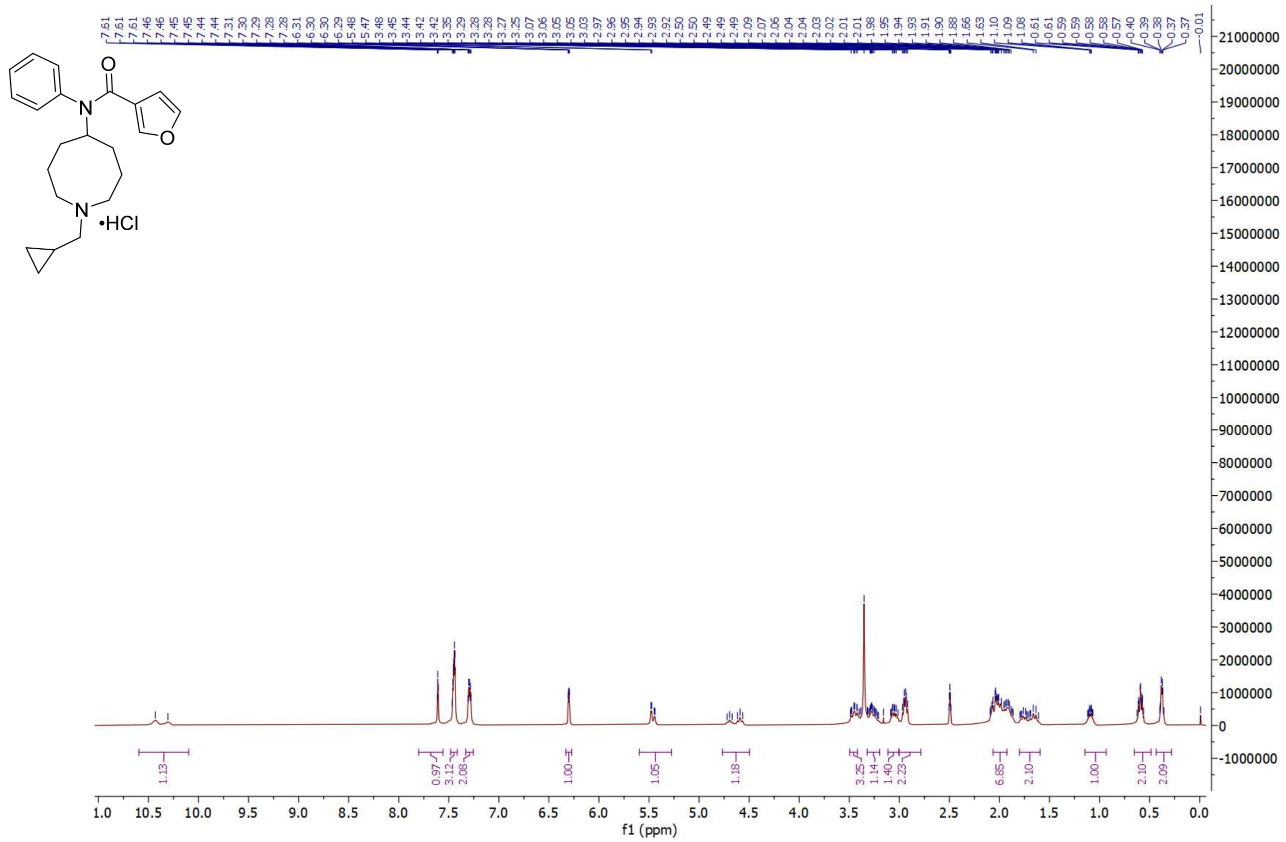

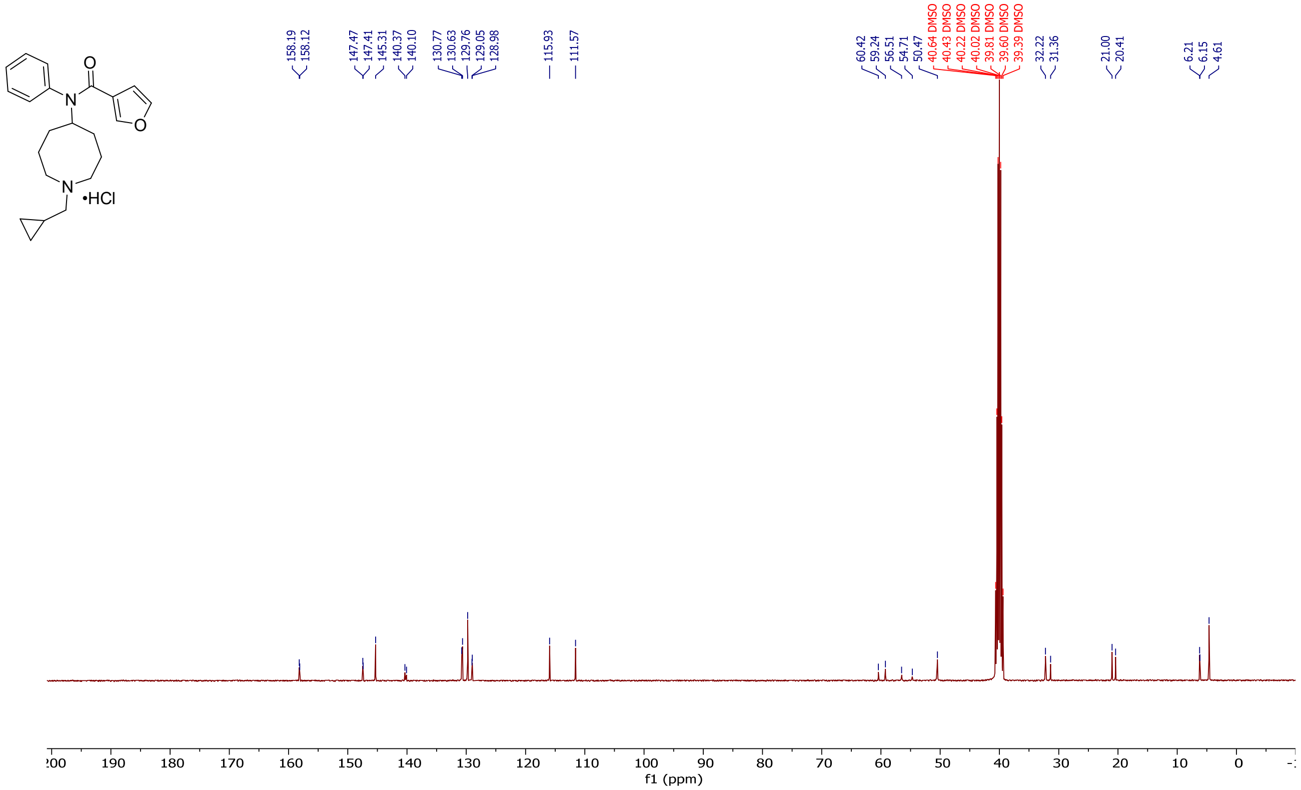

N-(1-(cyclobutylmethyl)azocan-5-yl)-N-phenylfuran-3-carboxamide hydrochloride **(55)**

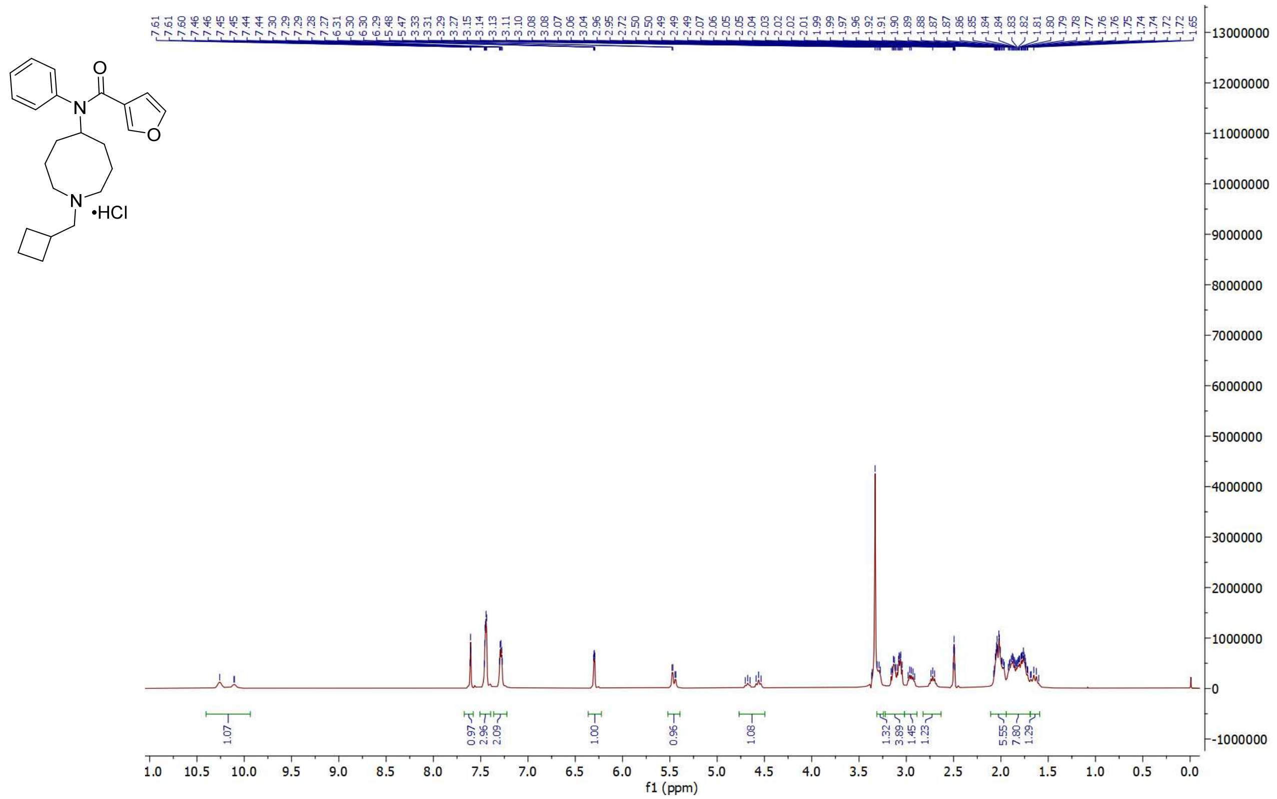

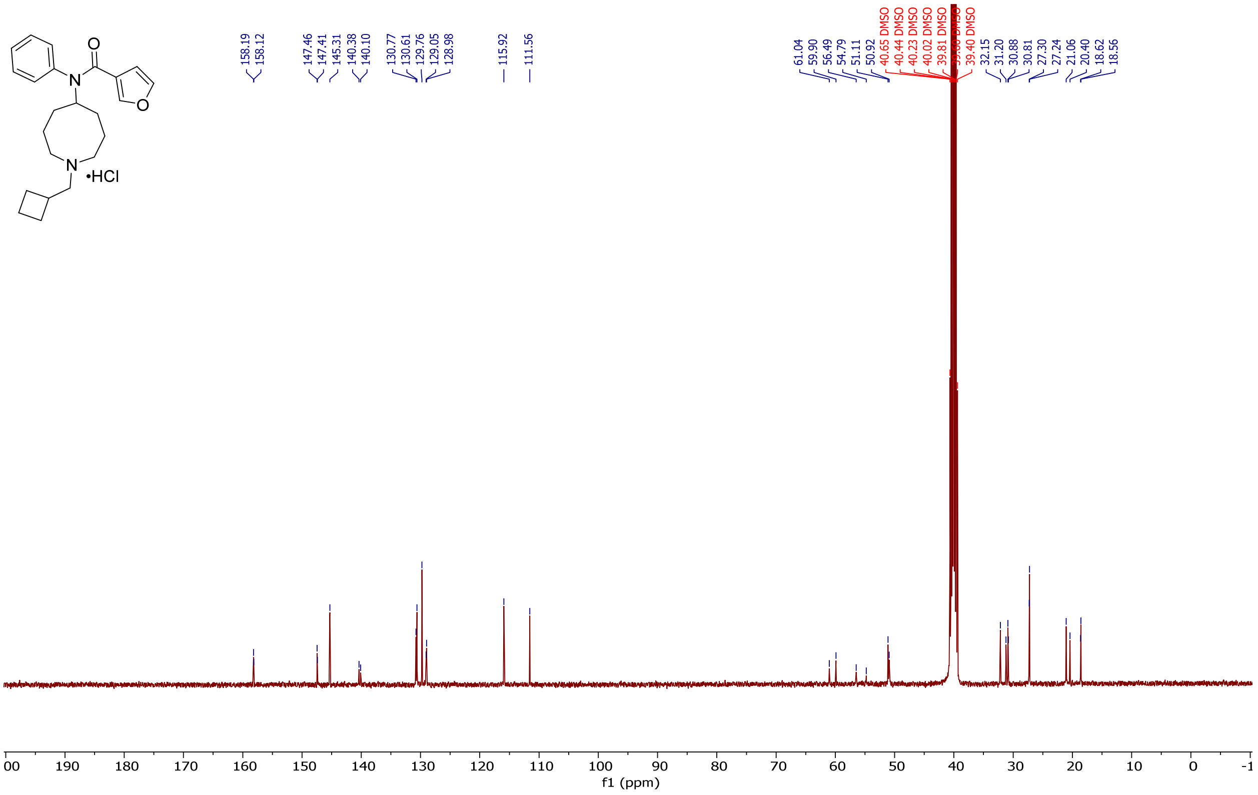

N-(1-(cyclopentylmethyl)azocan-5-yl)-N-phenylfuran-3-carboxamide hydrochloride **(56)**

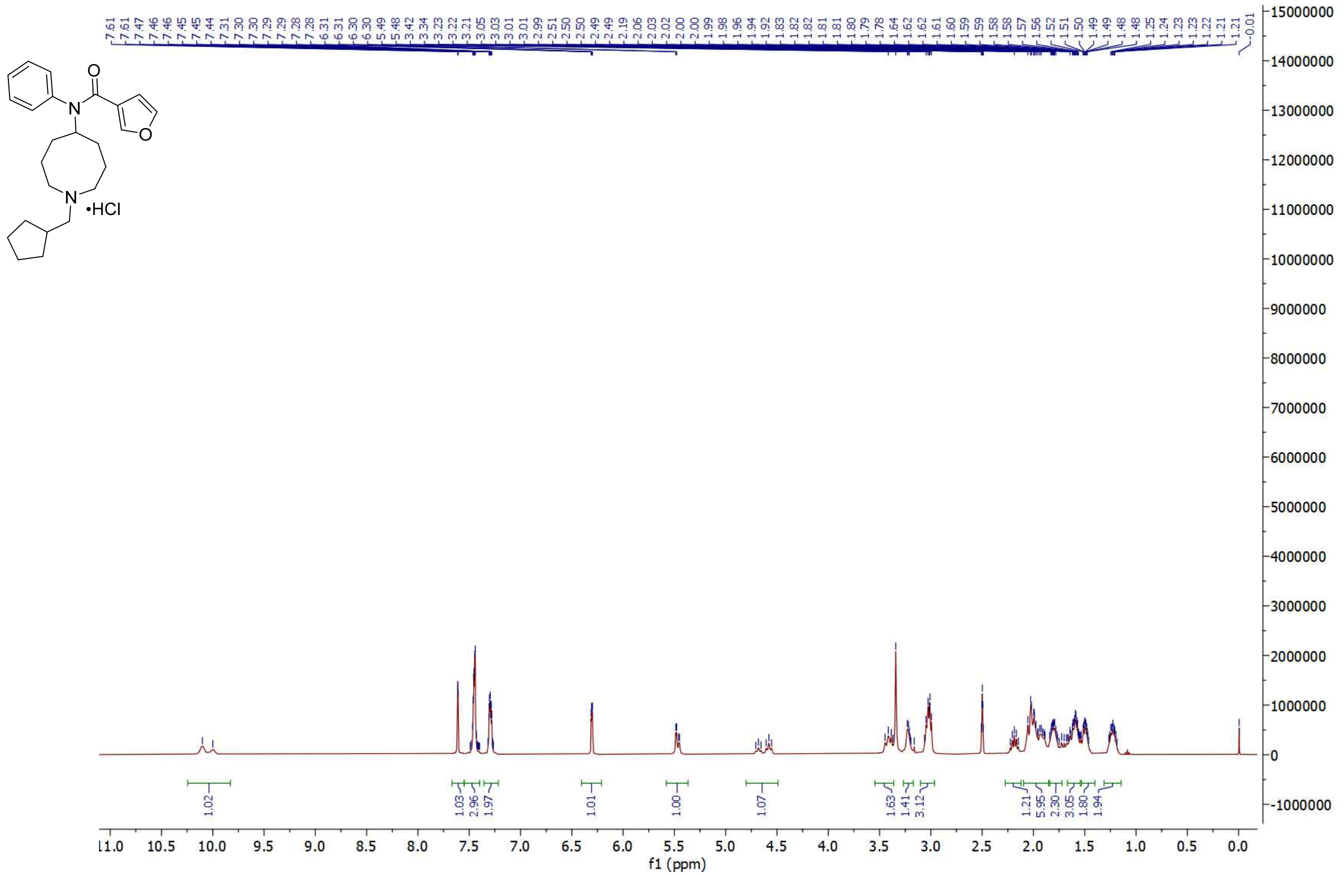

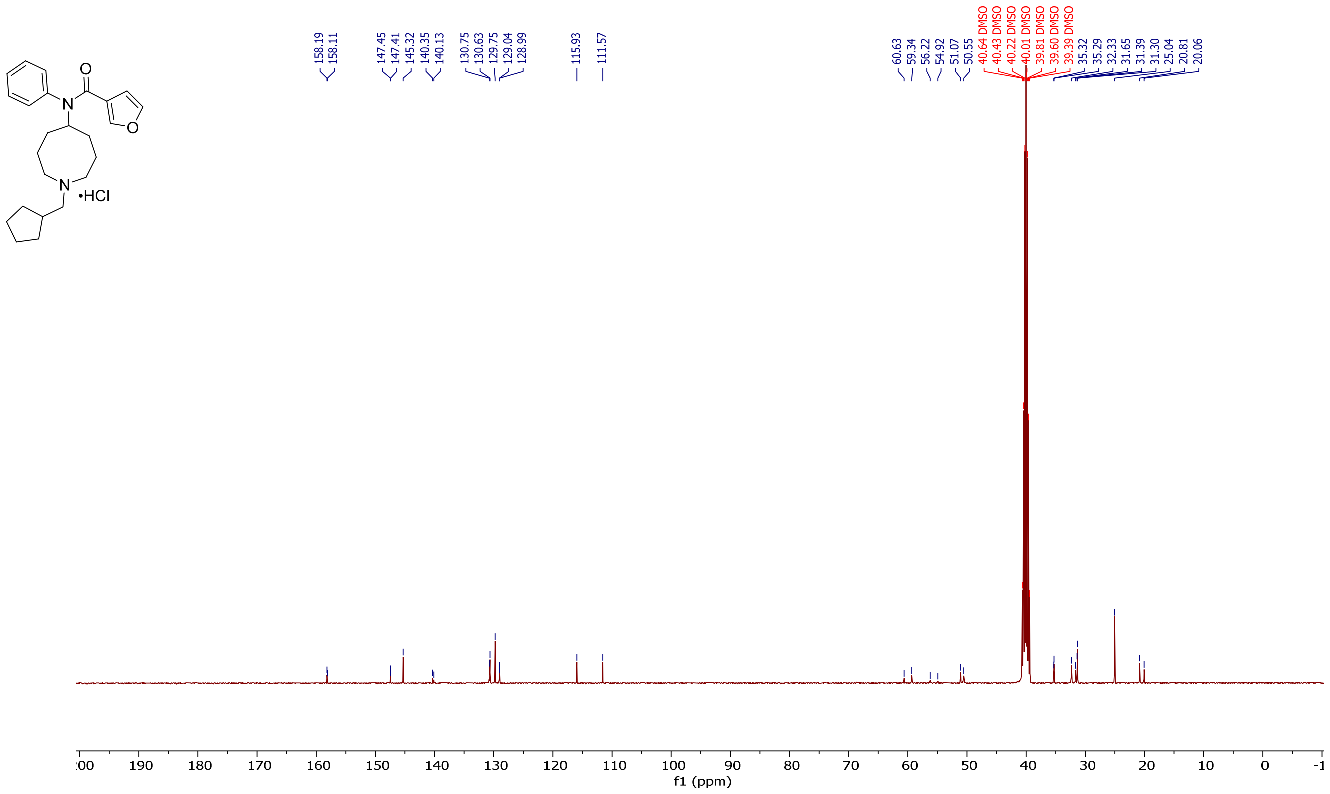

N-(1-(cyclohexylmethyl)azocan-5-yl)-N-phenylfuran-3-carboxamide hydrochloride **(57)**

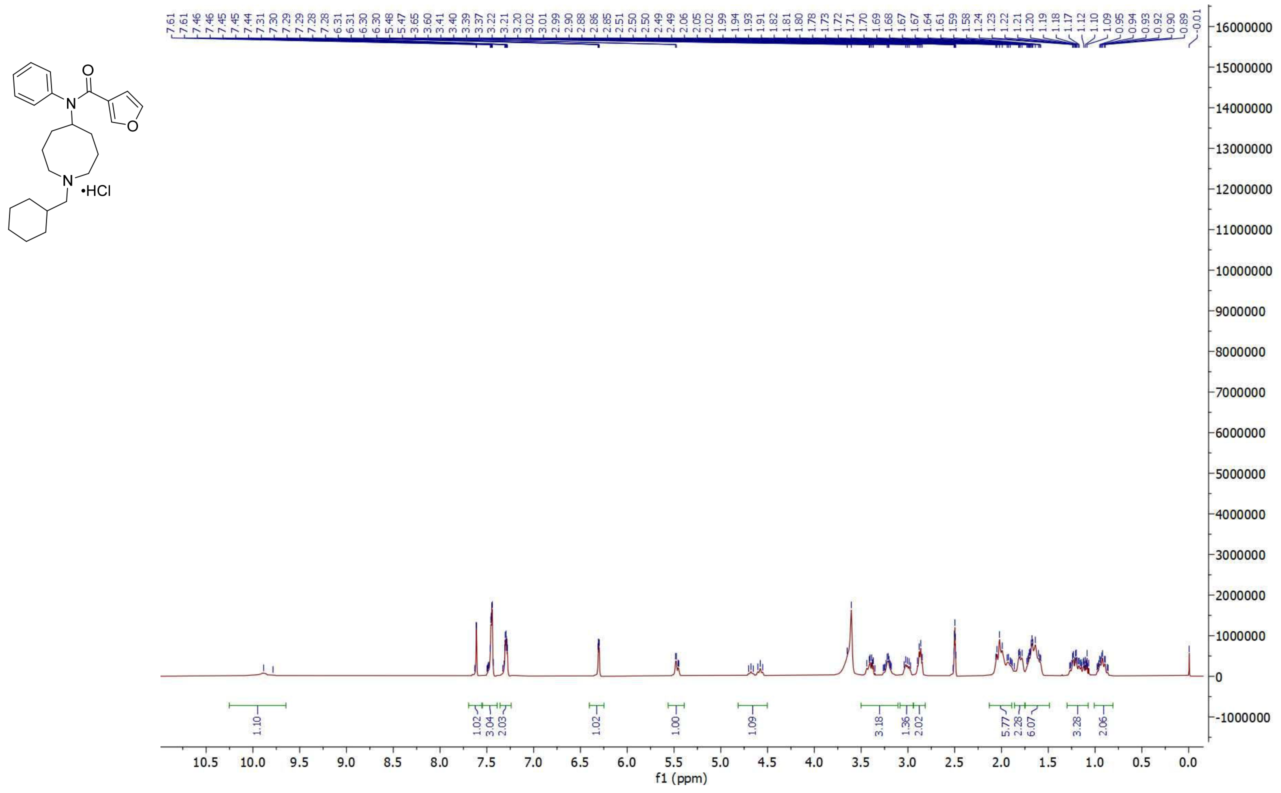

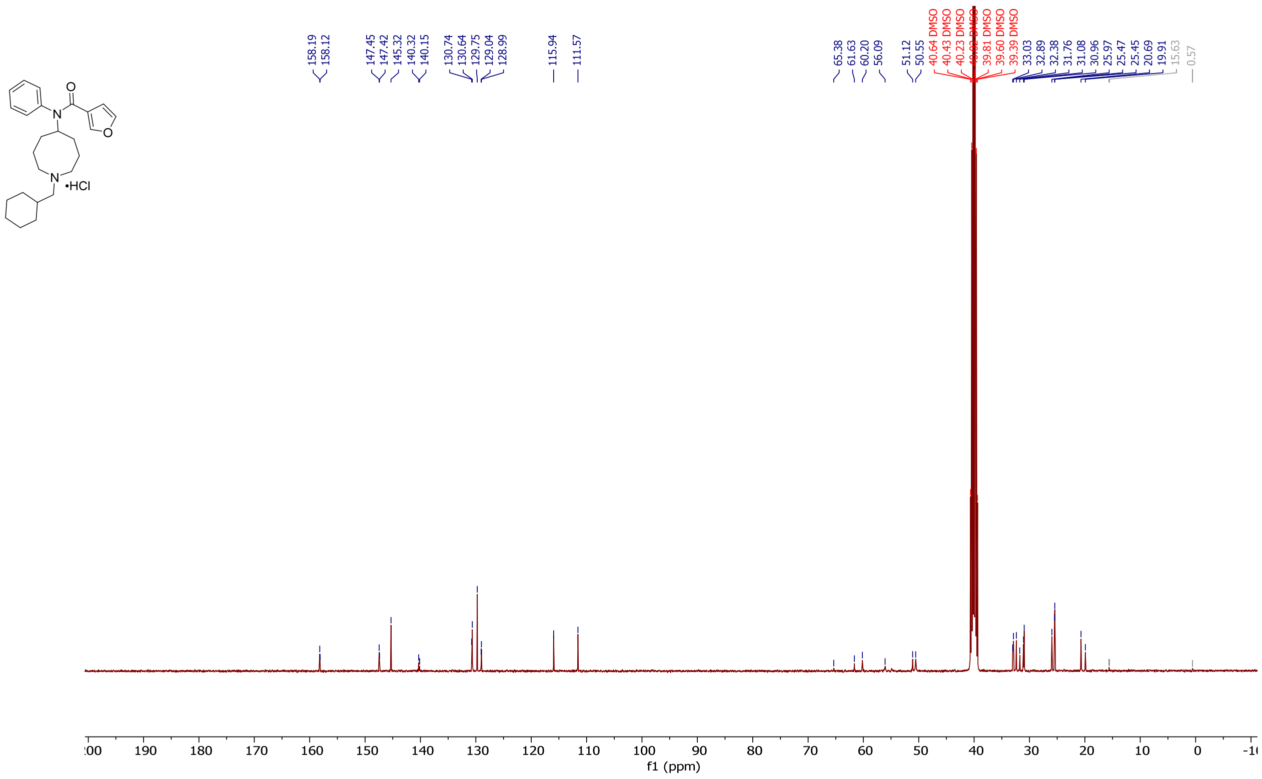

N-(1-benzylazocan-5-yl)-N-phenylfuran-3-carboxamide hydrochloride **(58)**

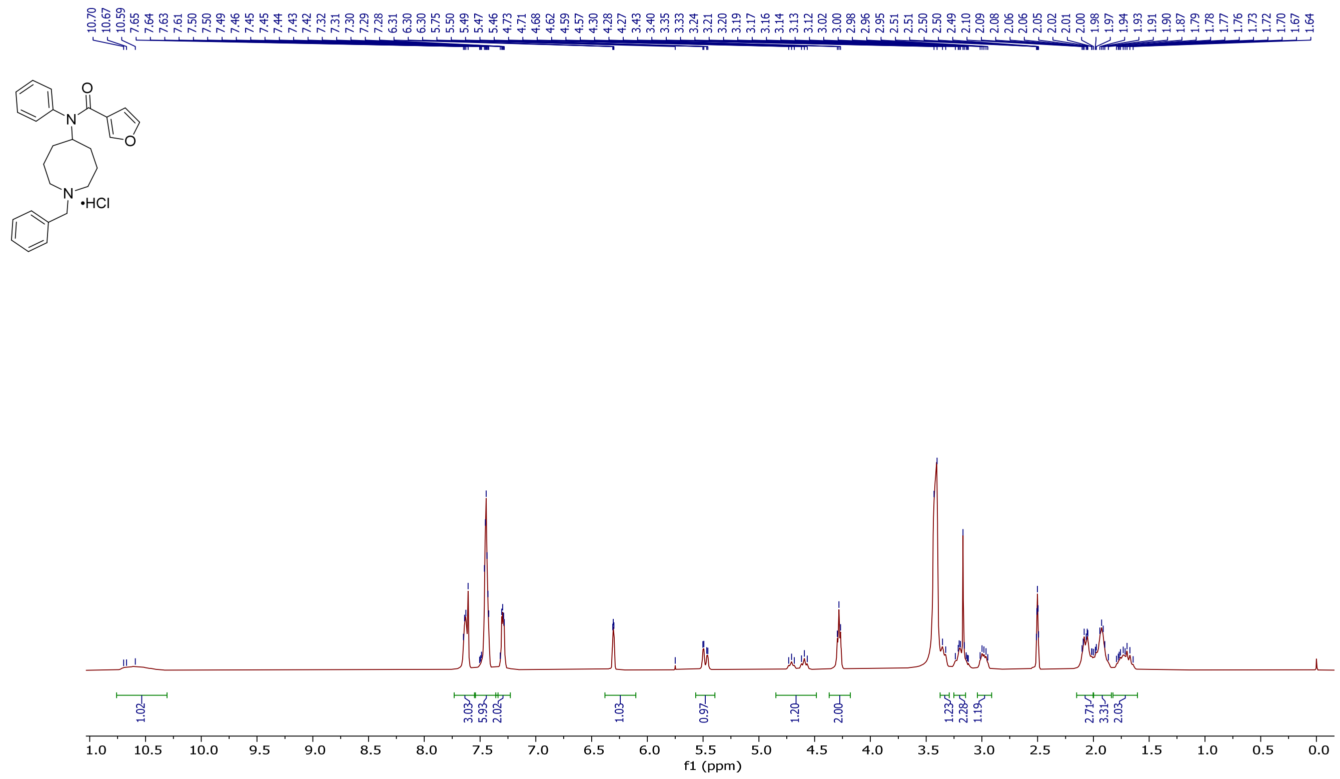

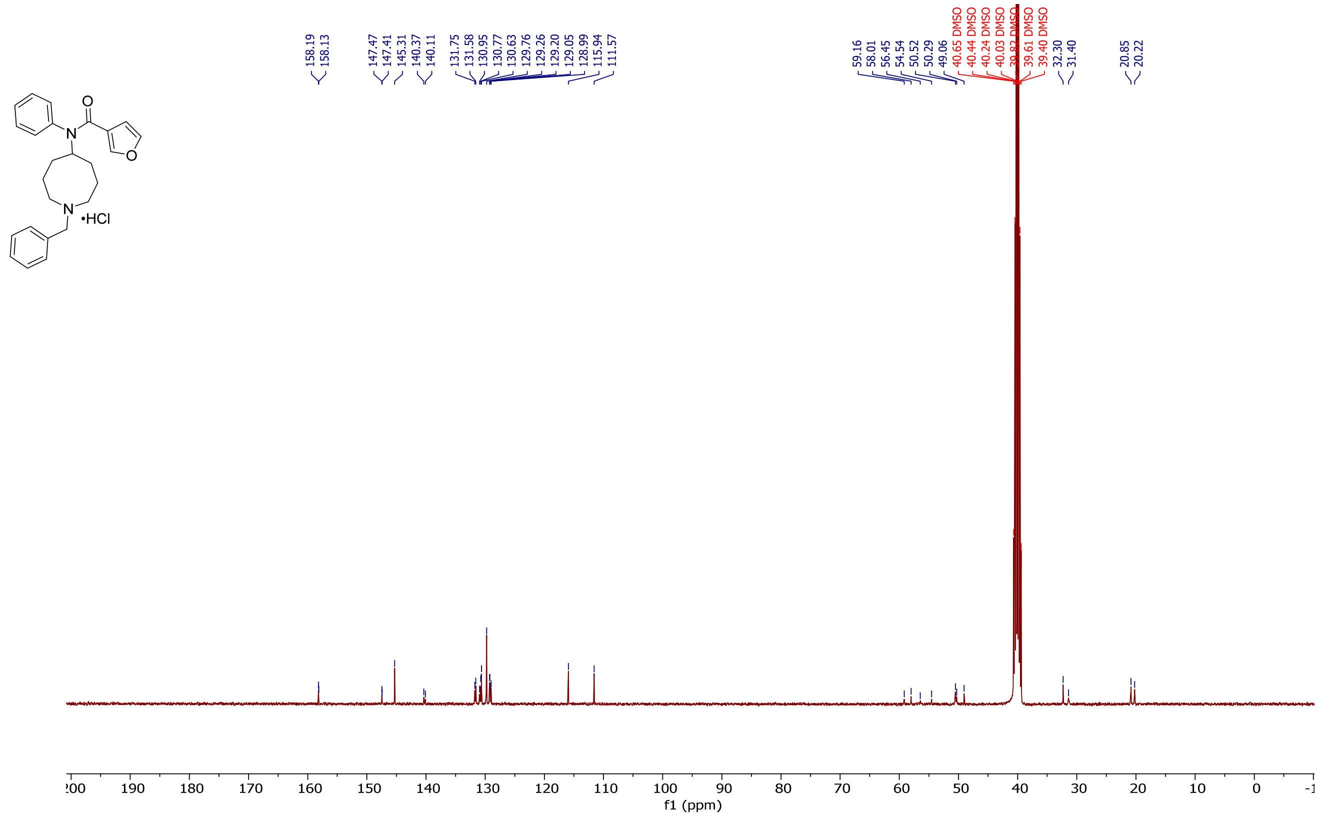

N-(1-phenethylazocan-5-yl)-N-phenylfuran-3-carboxamide hydrochloride **(59)**

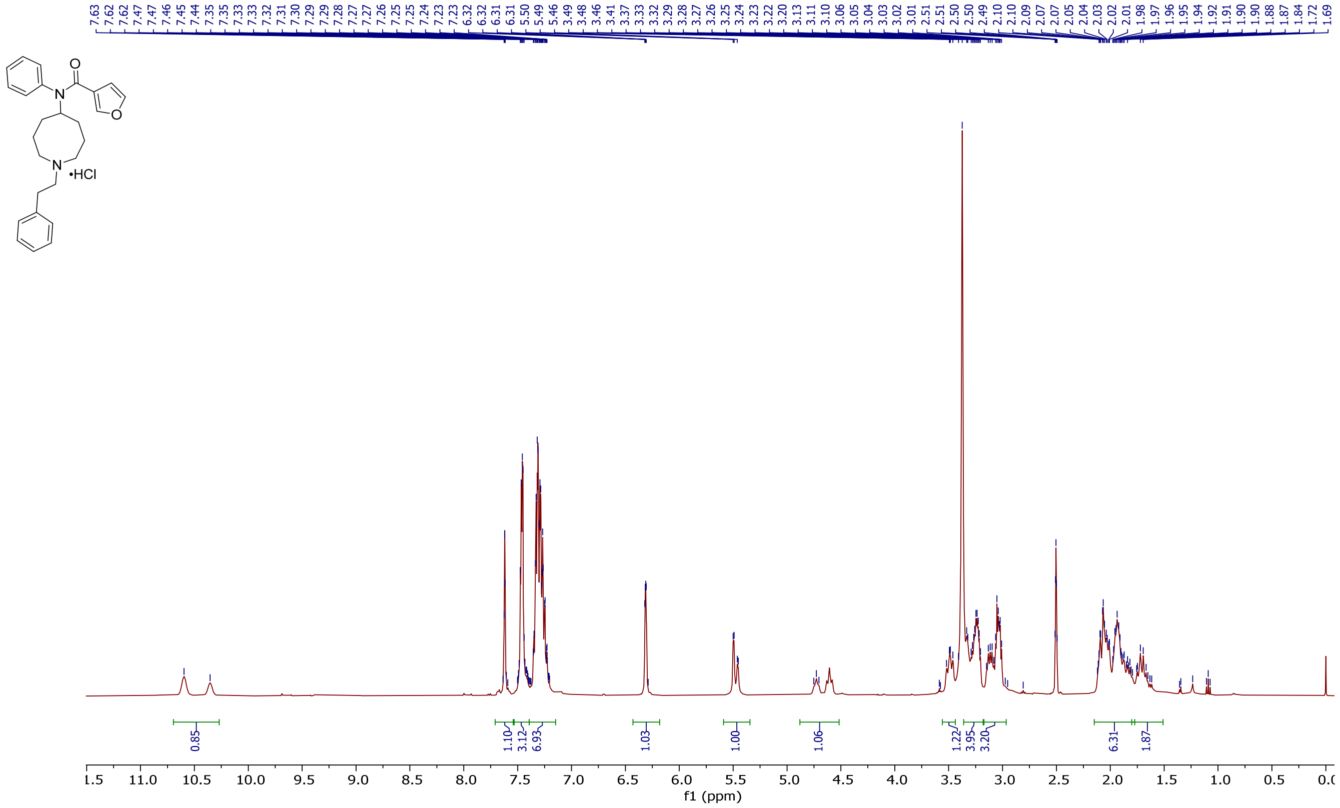

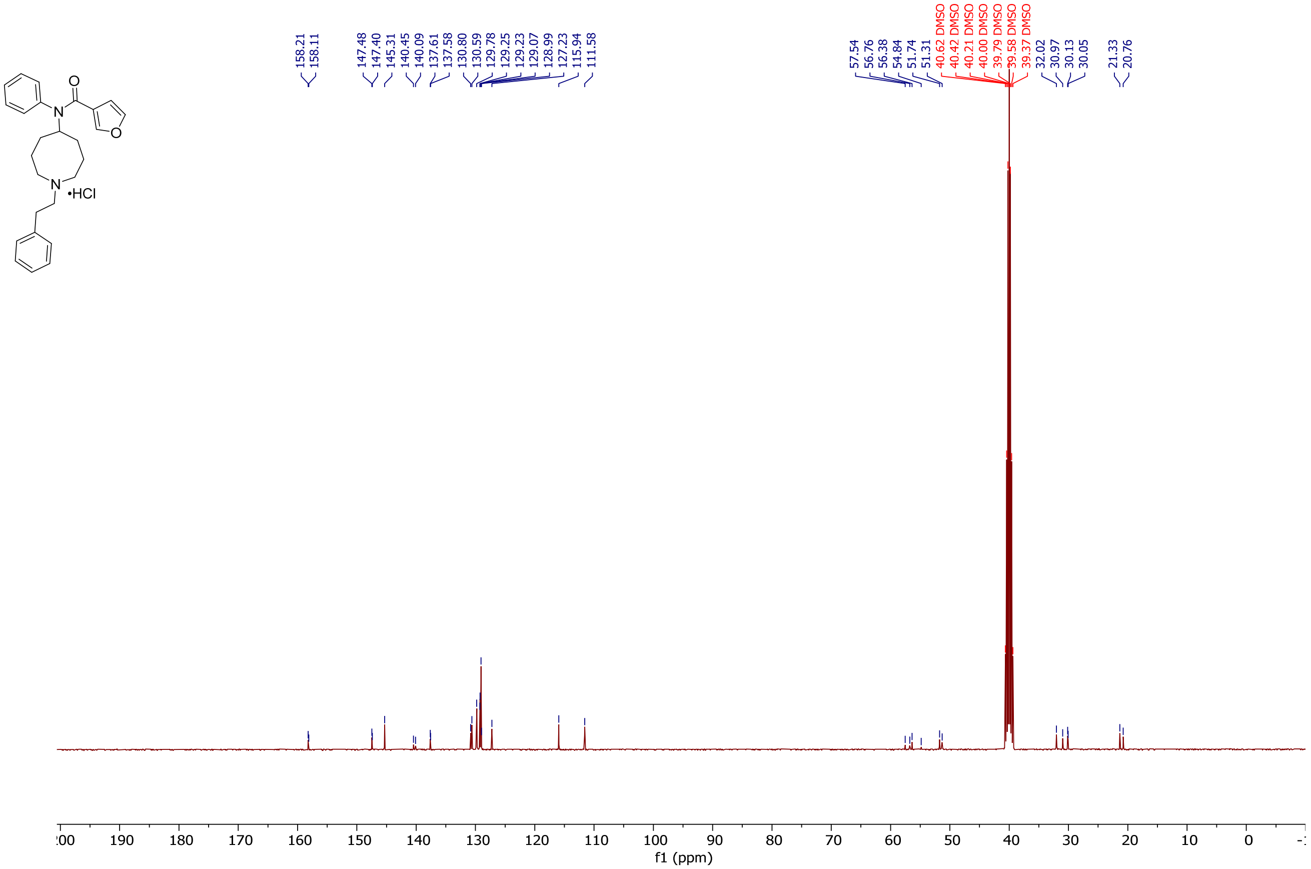

N-(1-allylazepan-4-yl)-N-phenylthiophene-2-carboxamide hydrogen chloride **(60)**

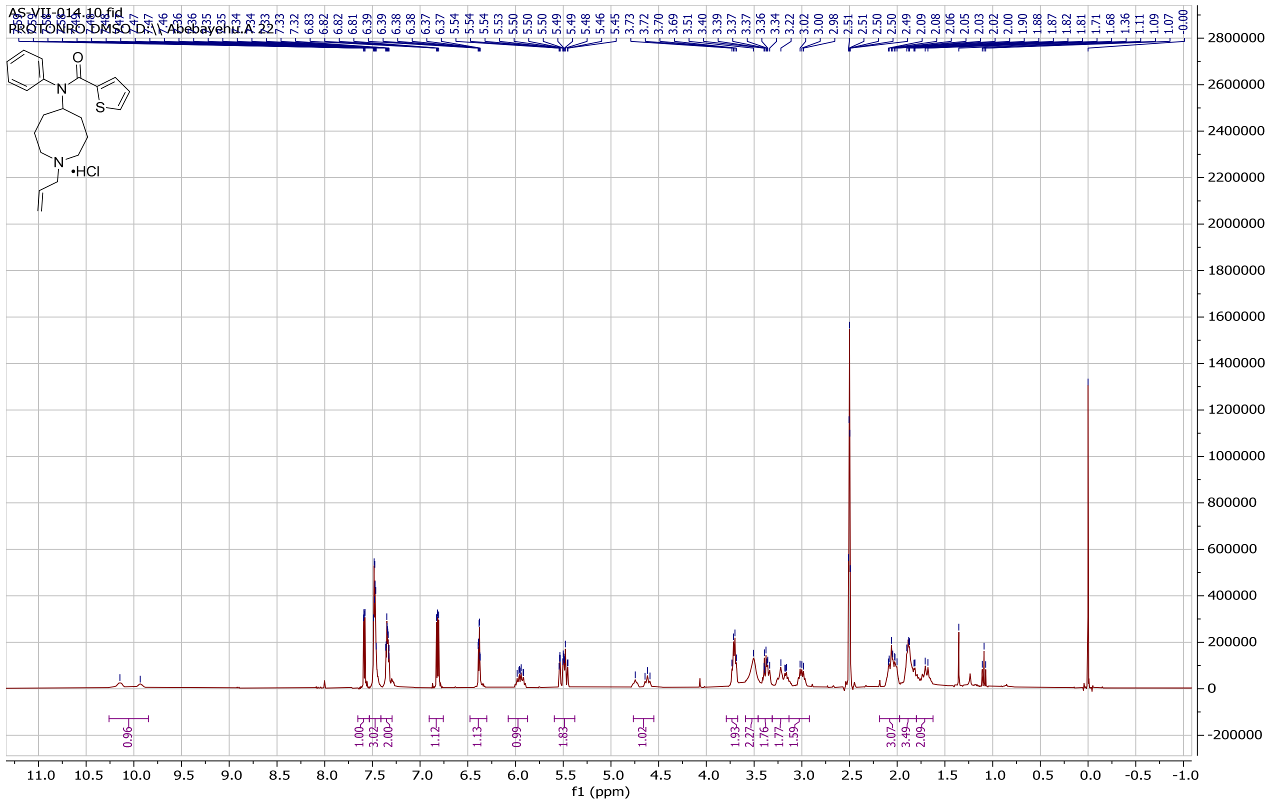

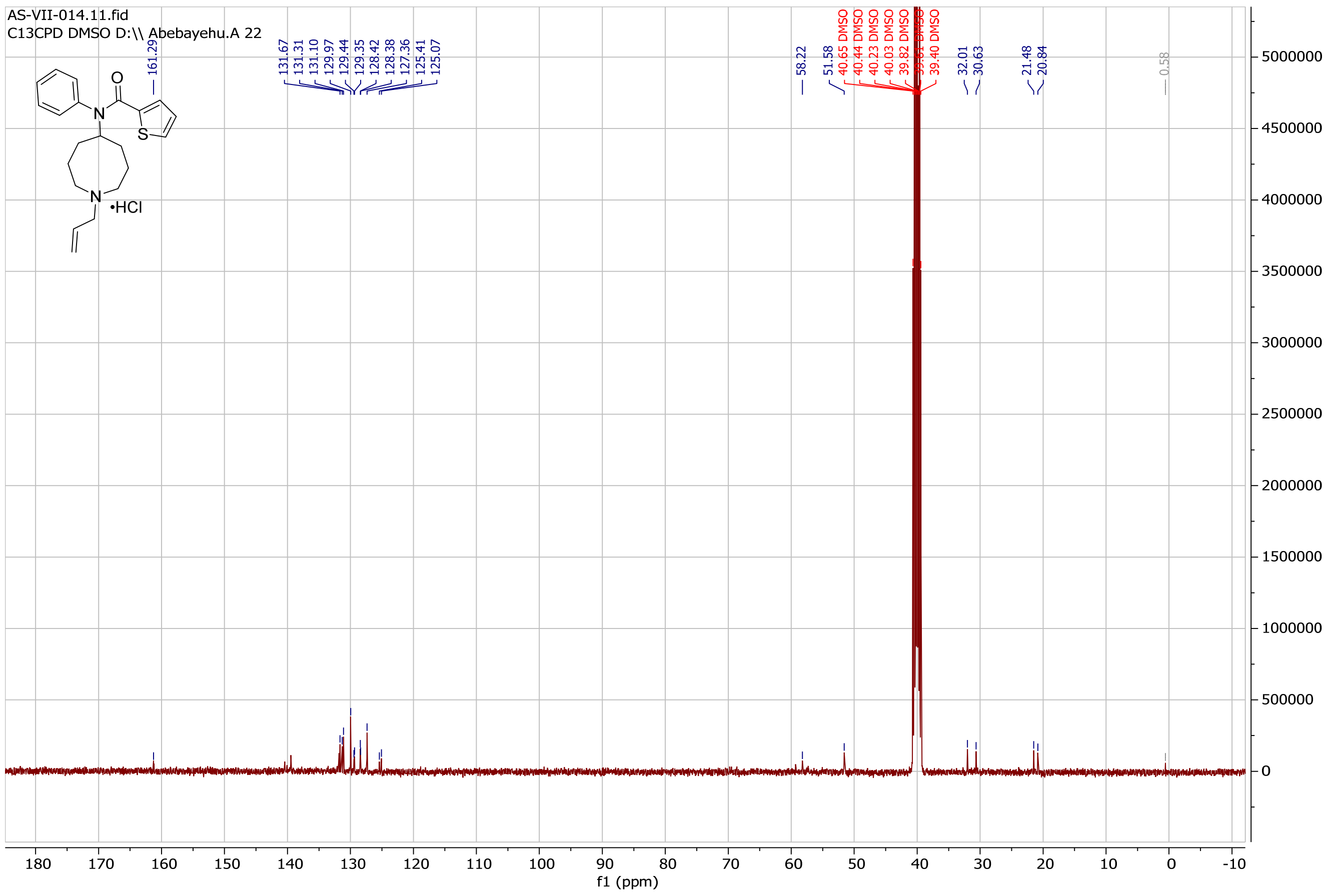

N-(1-(cyclopropylmethyl)azepan-4-yl)-N-phenylthiophene-2-carboxamide hydrogen chloride **(61)**

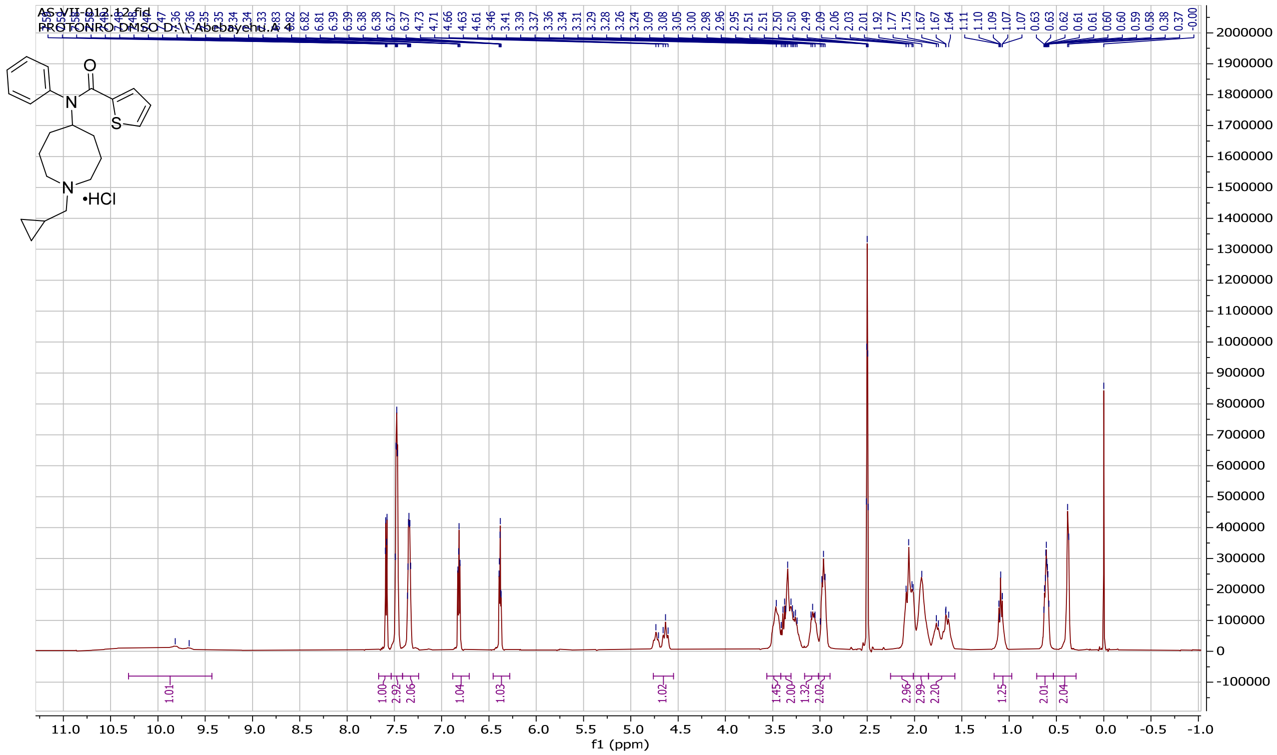

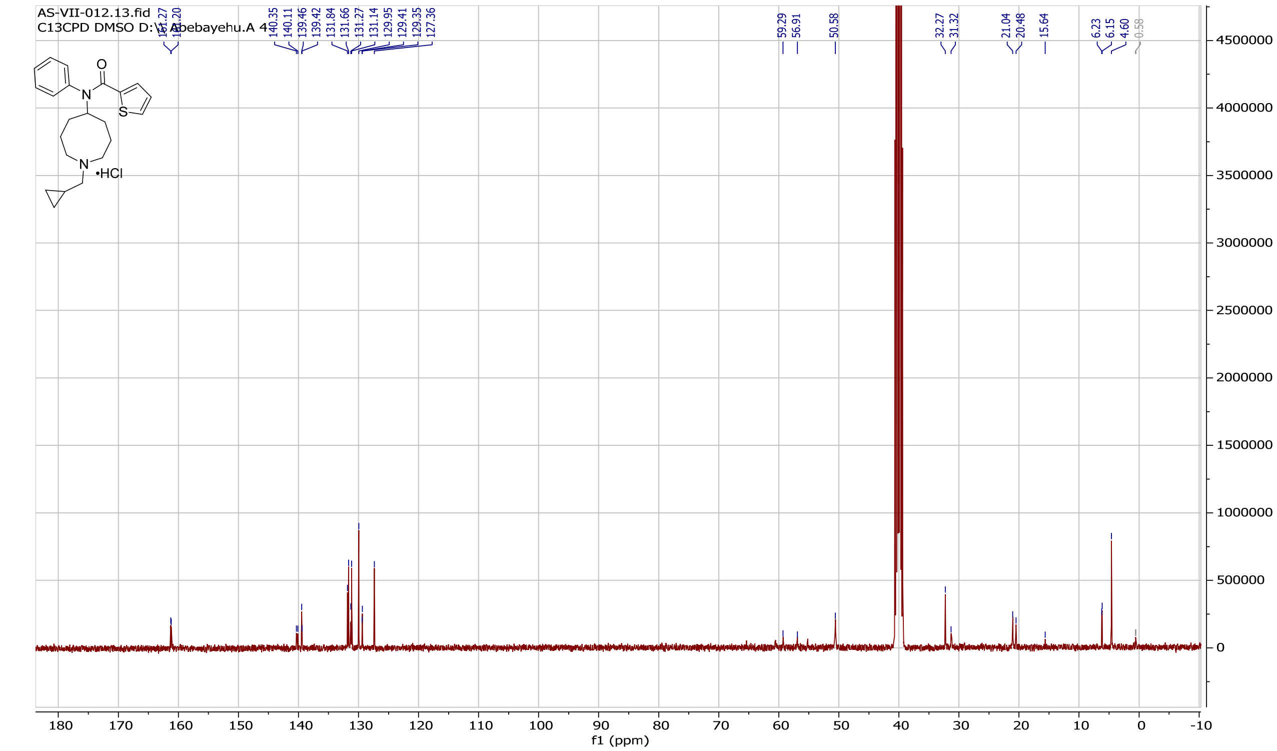

N-(1-(cyclobutylmethyl)azepan-4-yl)-N-phenylthiophene-2-carboxamide hydrogen chloride **(62)**

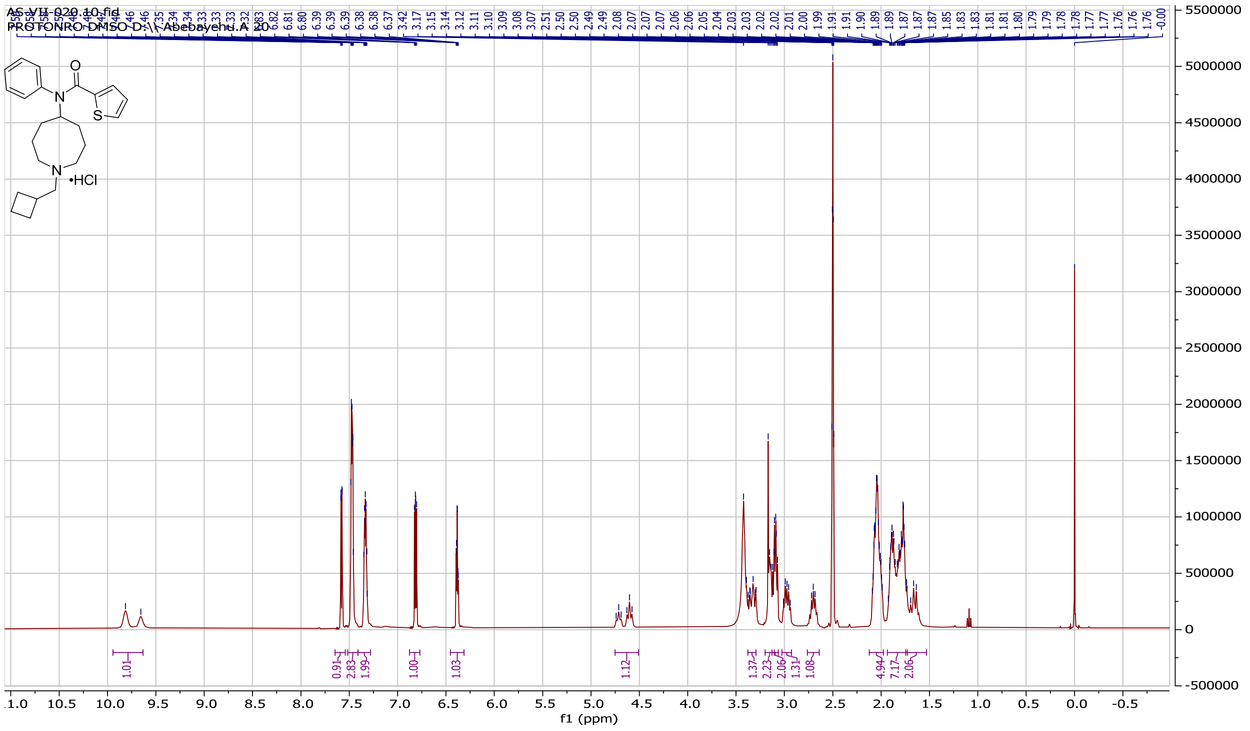

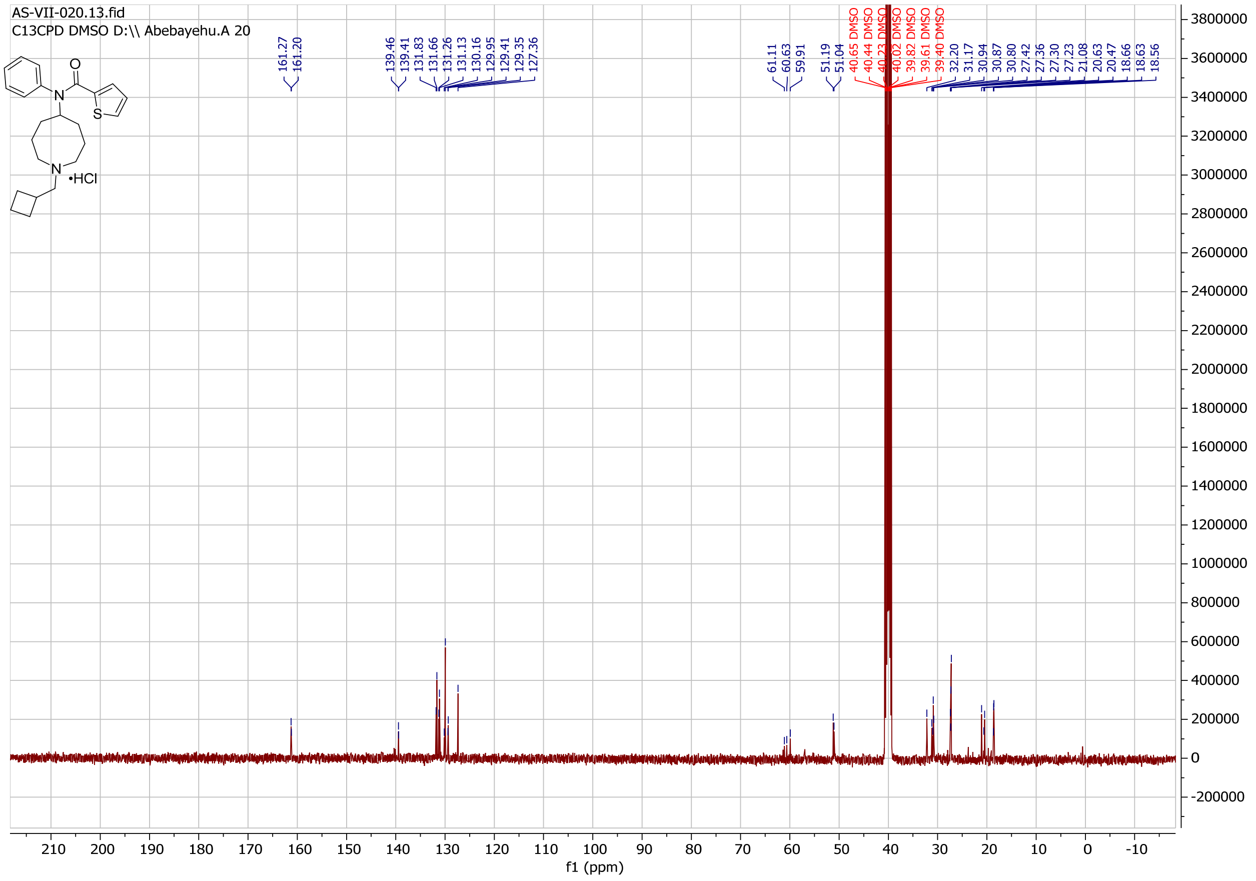

N-(1-(cyclopentylmethyl)azepan-4-yl)-N-phenylthiophene-2-carboxamide hydrogen chloride **(63)**

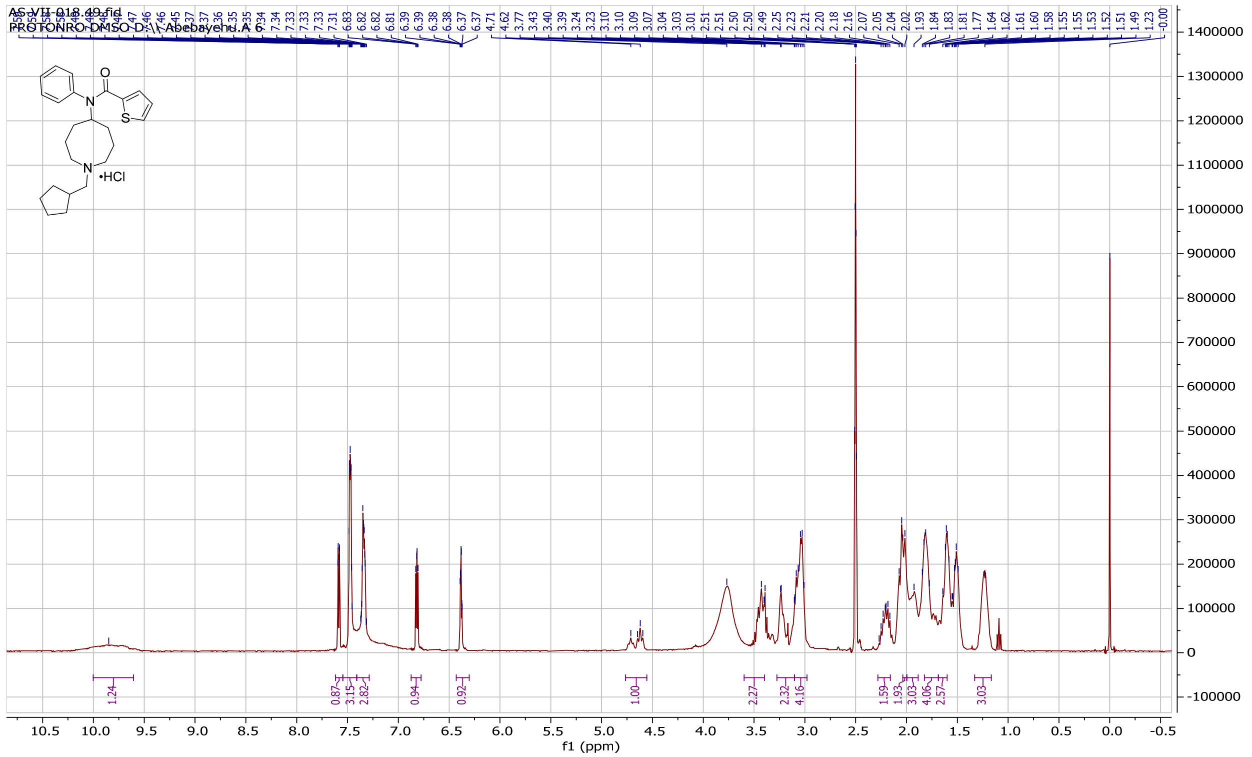

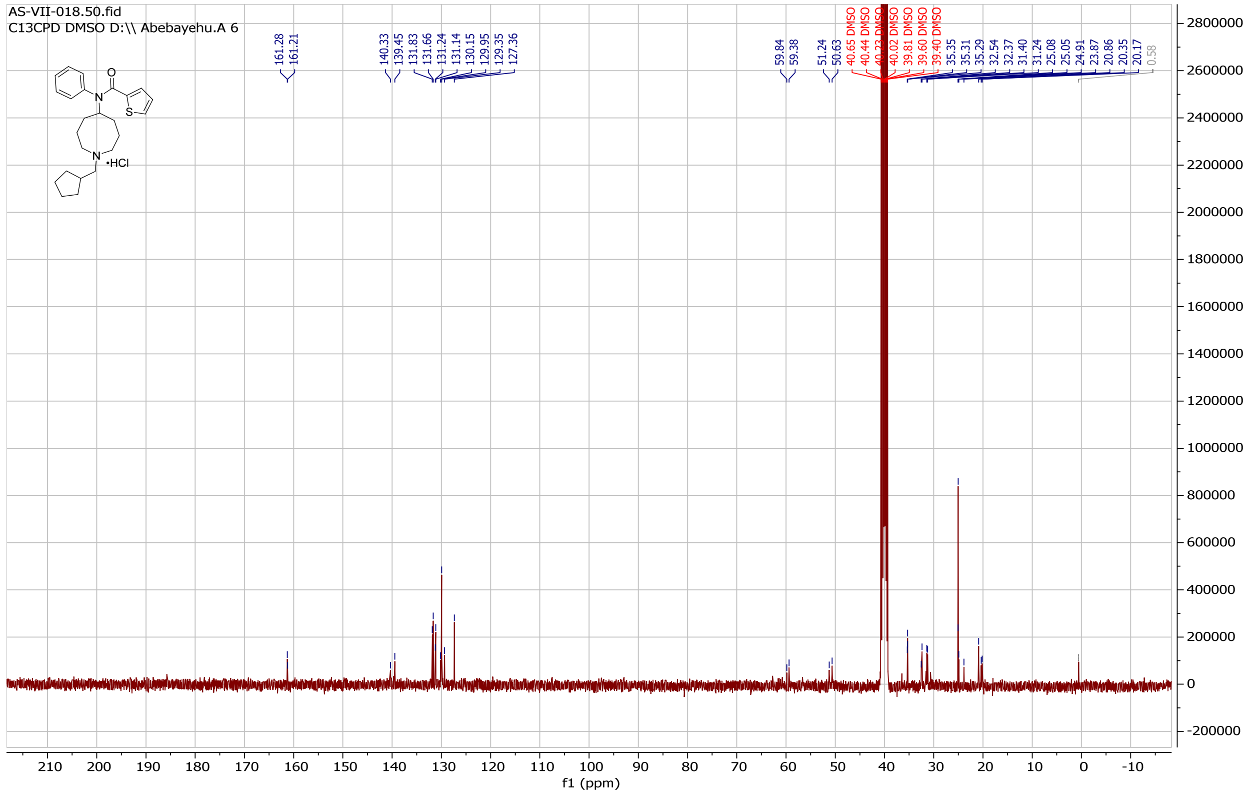

N-(1-(cyclohexylmethyl)azepan-4-yl)-N-phenylthiophene-2-carboxamide hydrogen chloride **(64)**

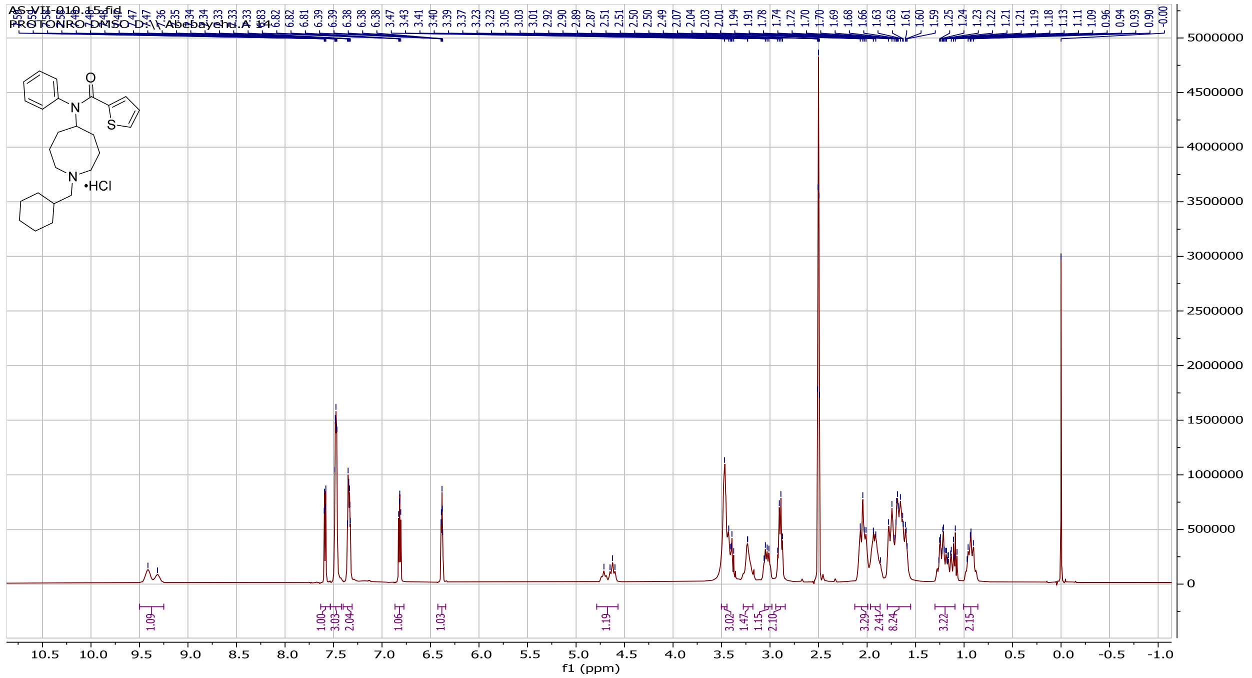

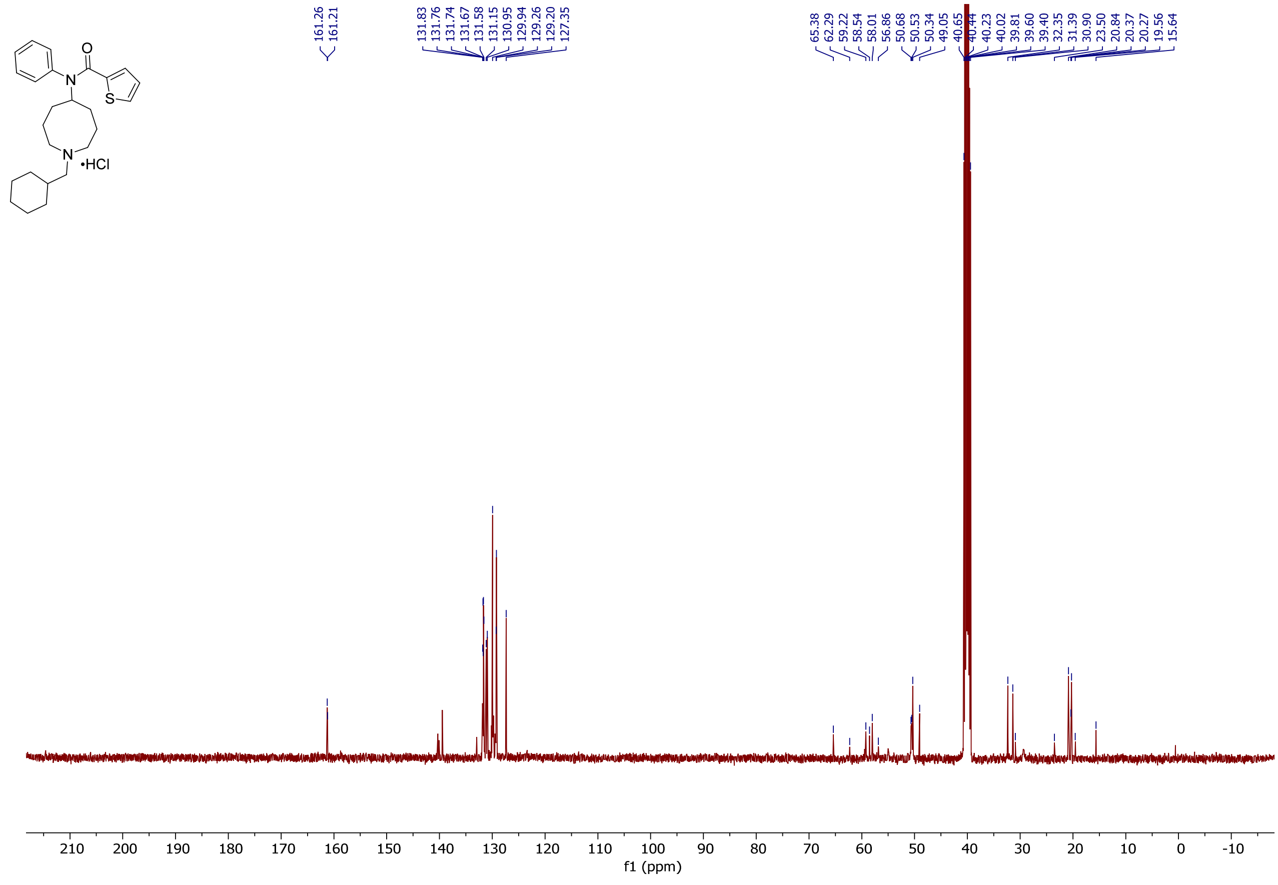

N-(1-benzylazepan-4-yl)-N-phenylthiophene-2-carboxamide hydrogen chloride **(65)**

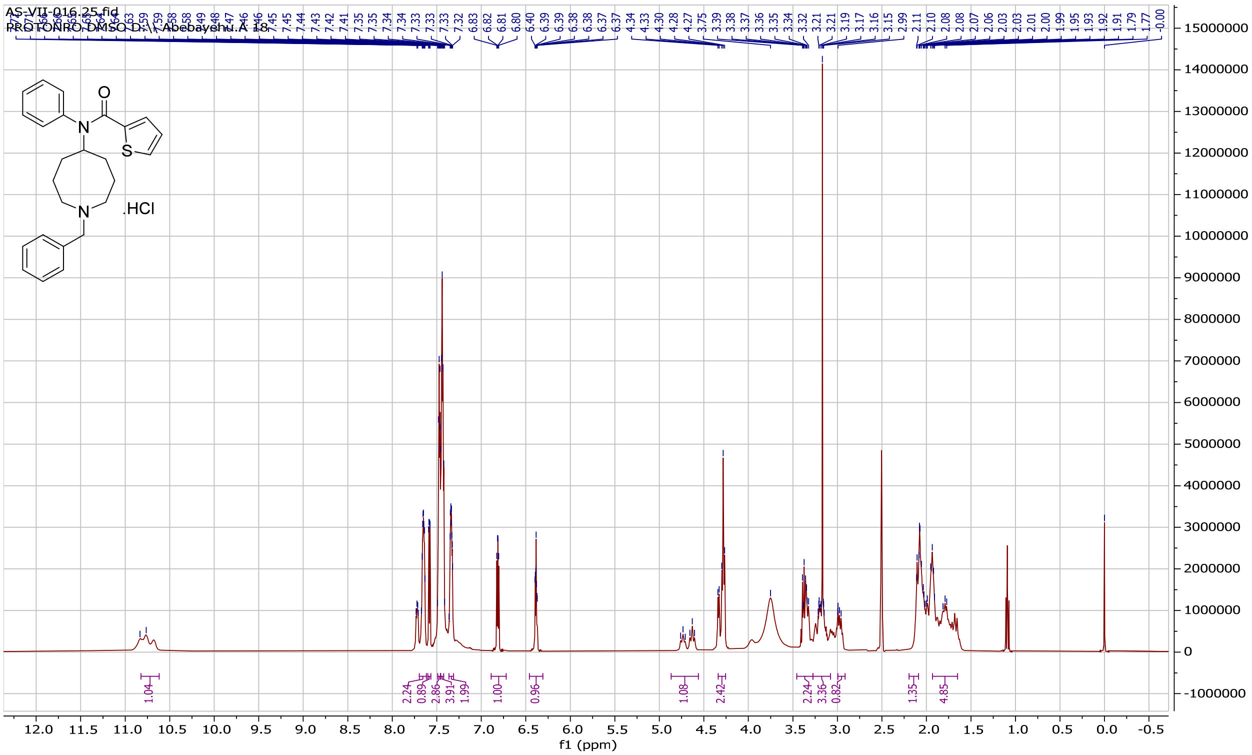

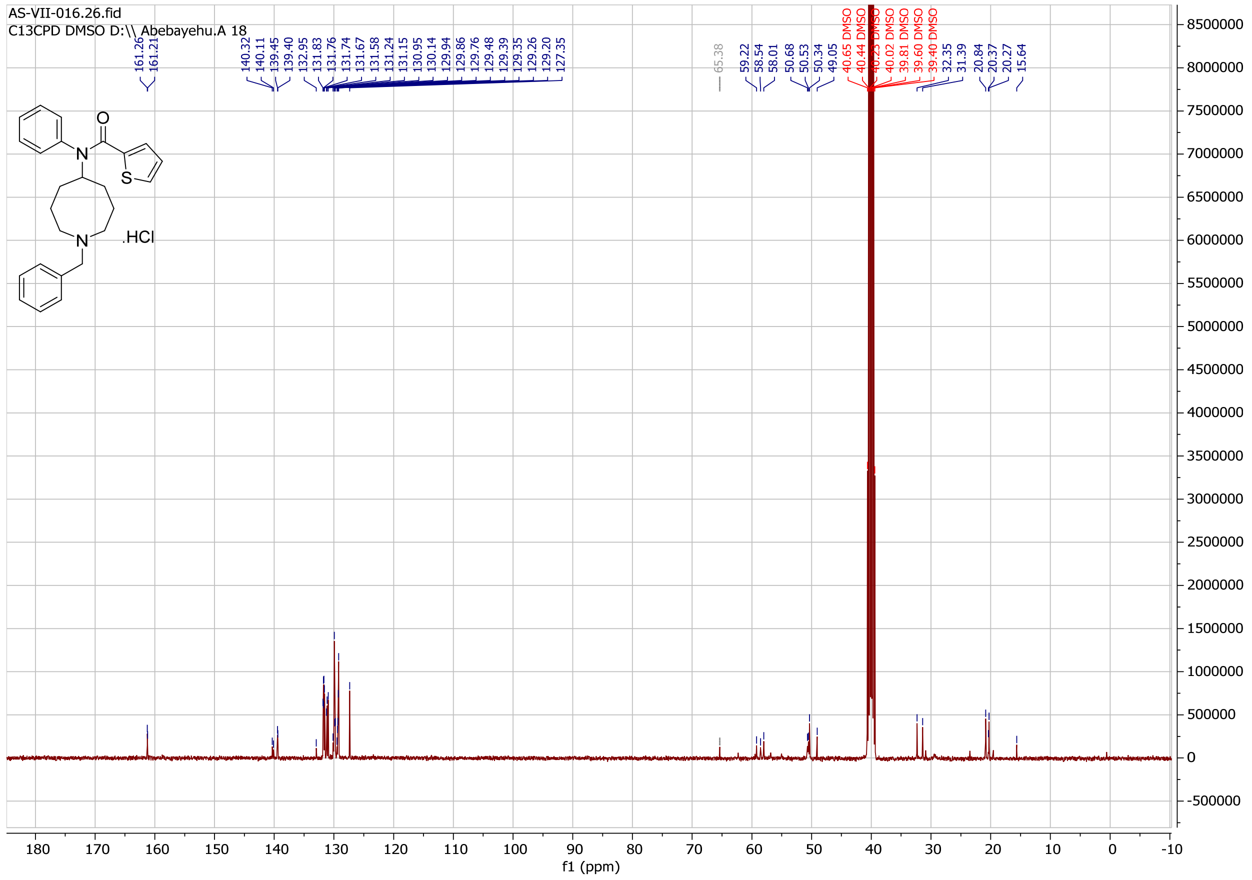

N-(1-phenethylazepan-4-yl)-N-phenylthiophene-2-carboxamide hydrogen chloride **(66)**

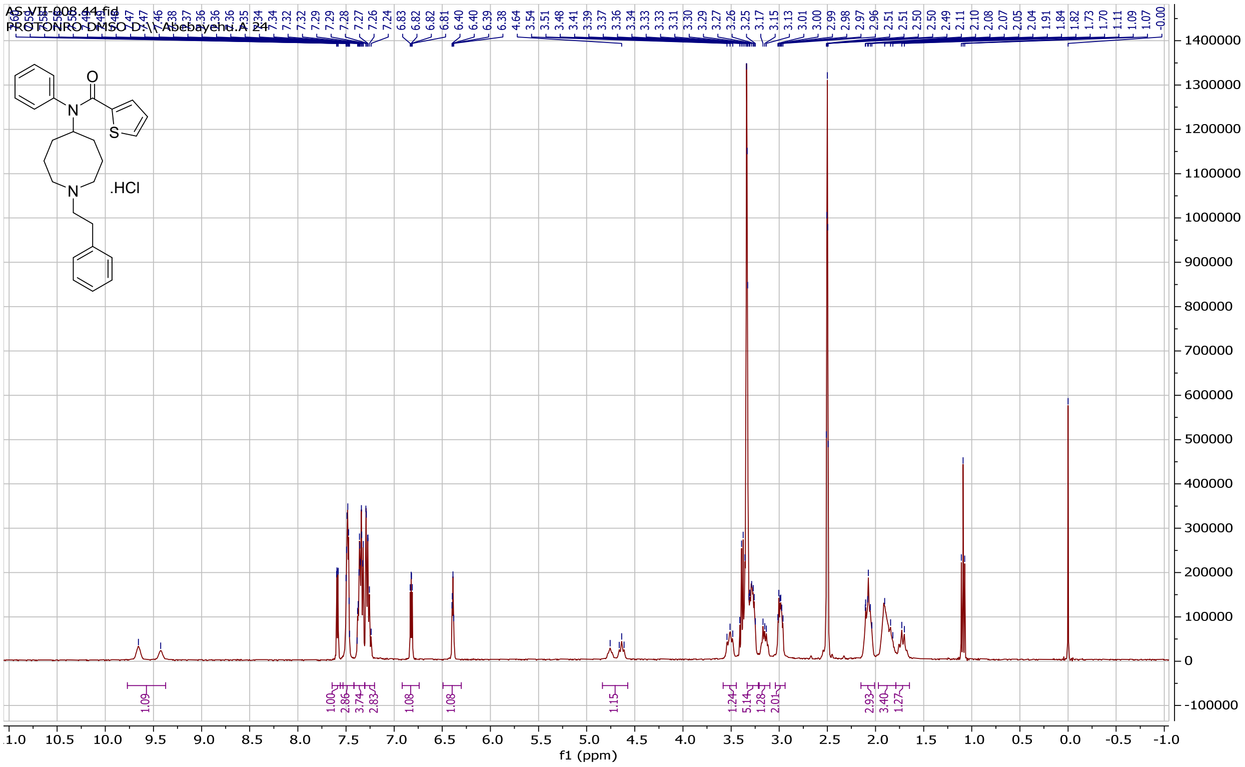

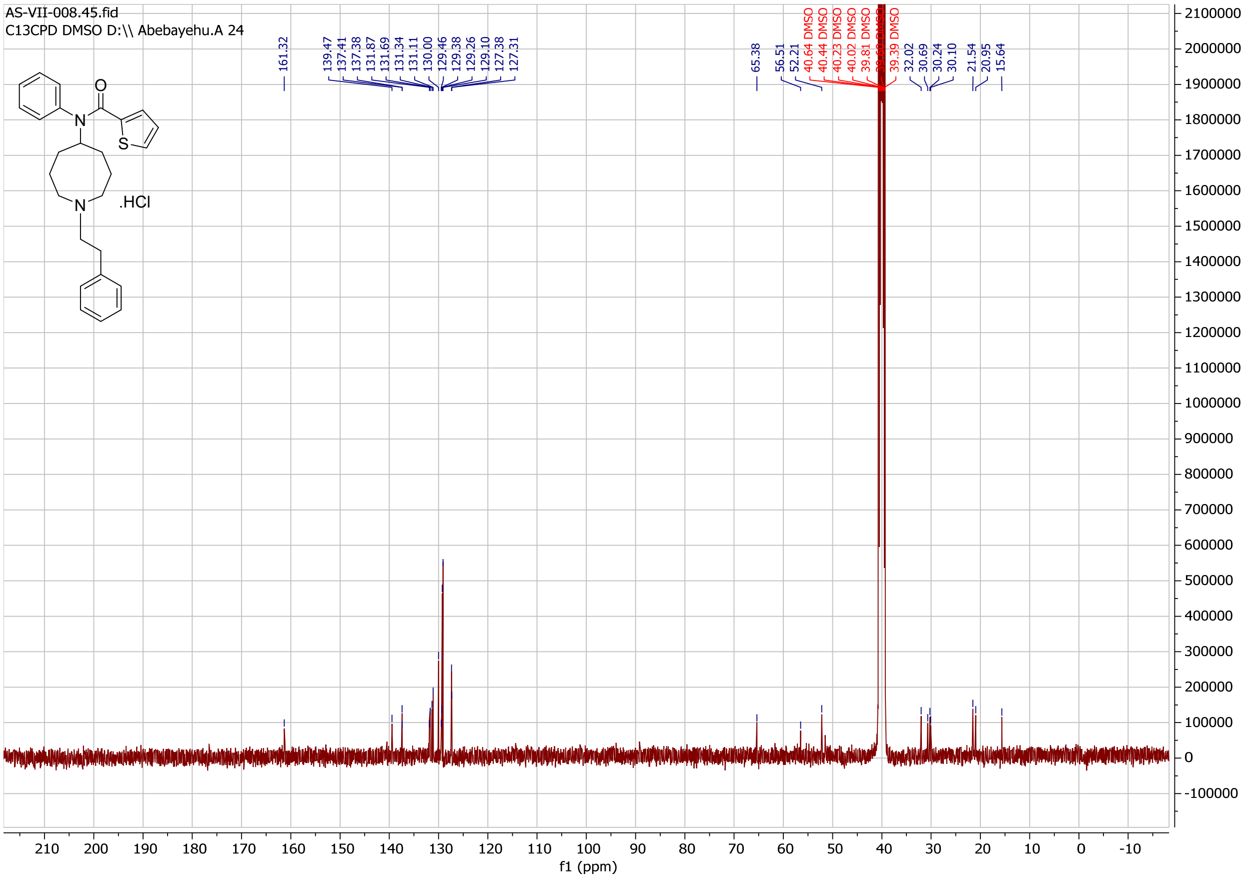

N-(1-allylazepan-4-yl)-N-phenyl-1H-pyrrole-2-carboxamide hydrogen chloride **(67)**

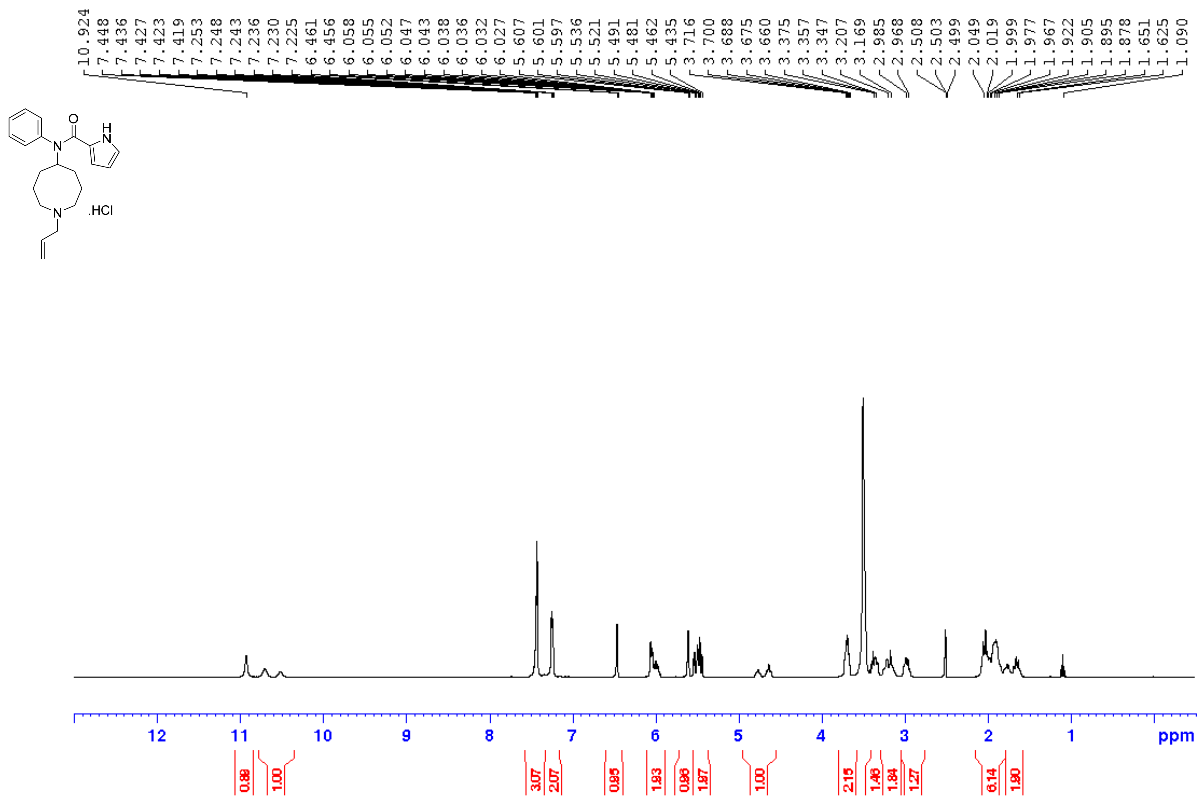

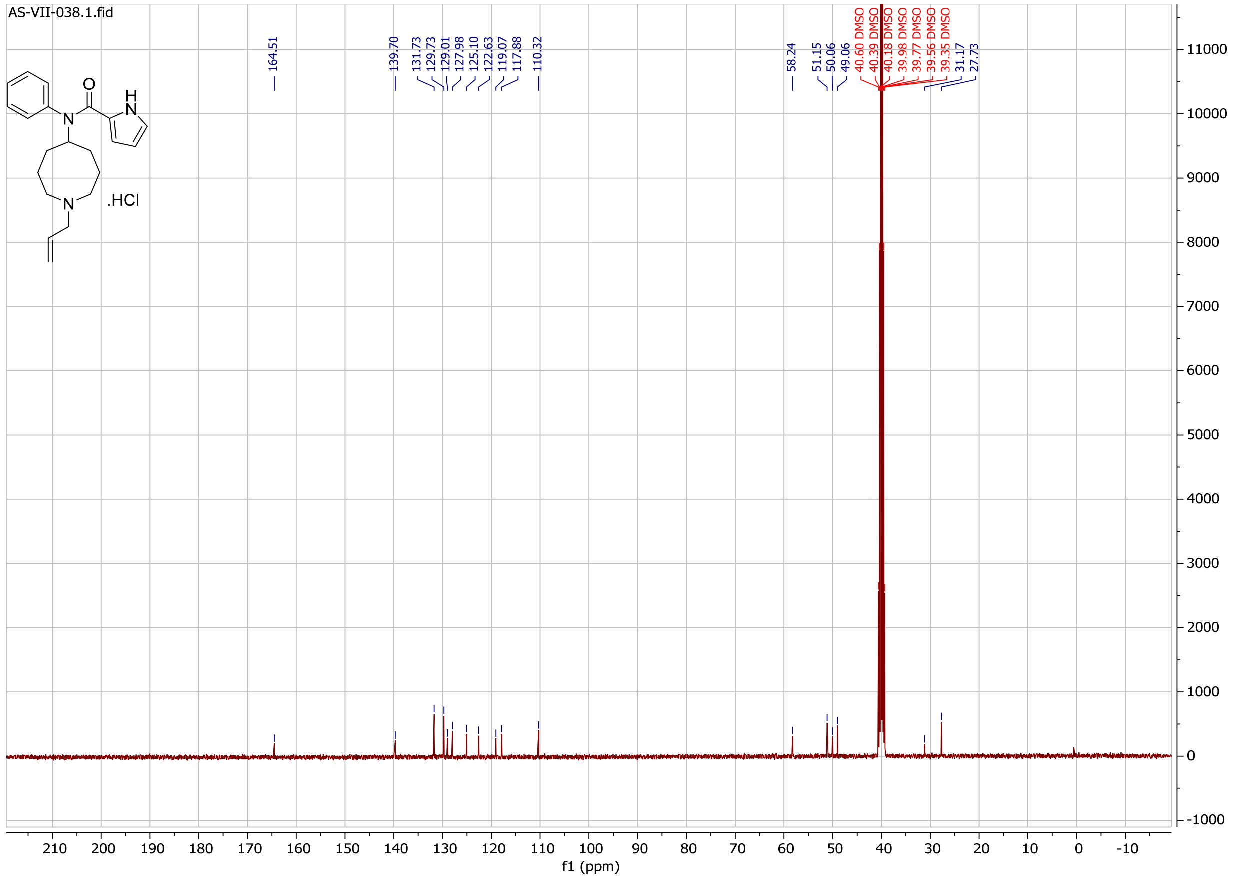

N-(1-(cyclopropylmethyl)azepan-4-yl)-N-phenyl-1H-pyrrole-2-carboxamide hydrogen chloride **(68)**

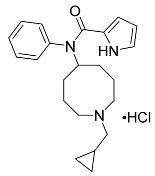

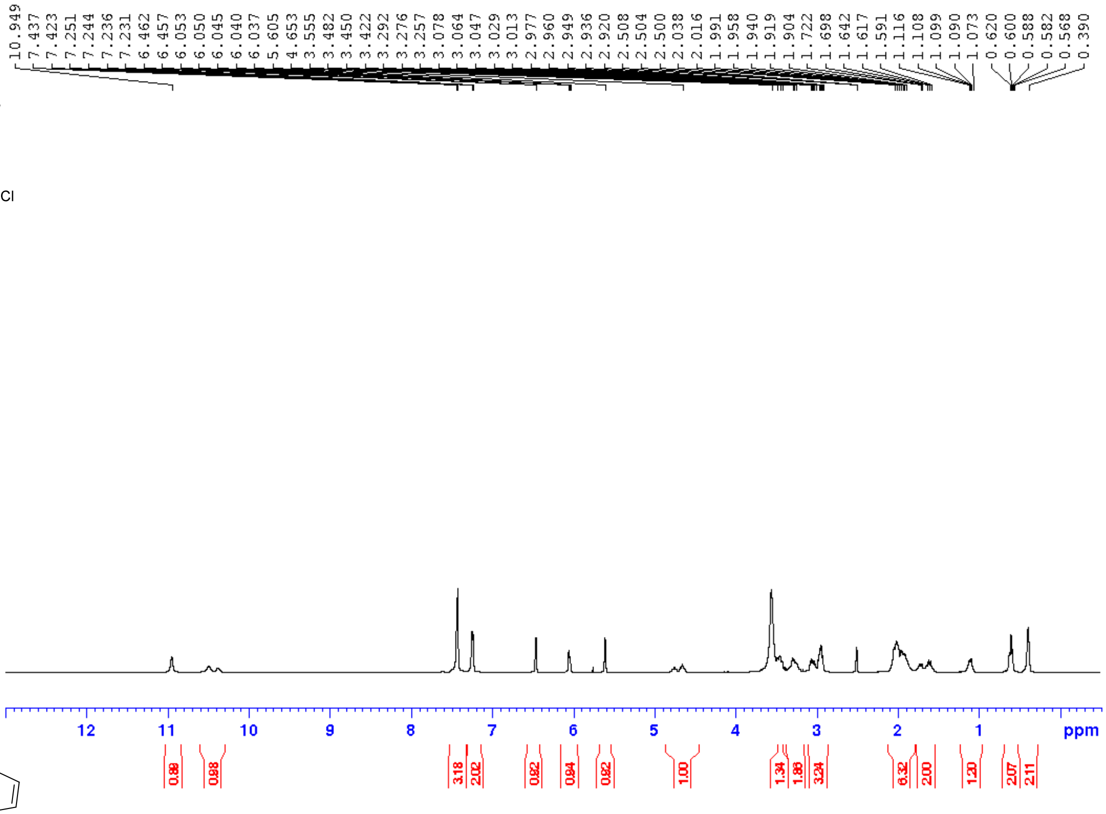

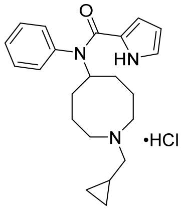

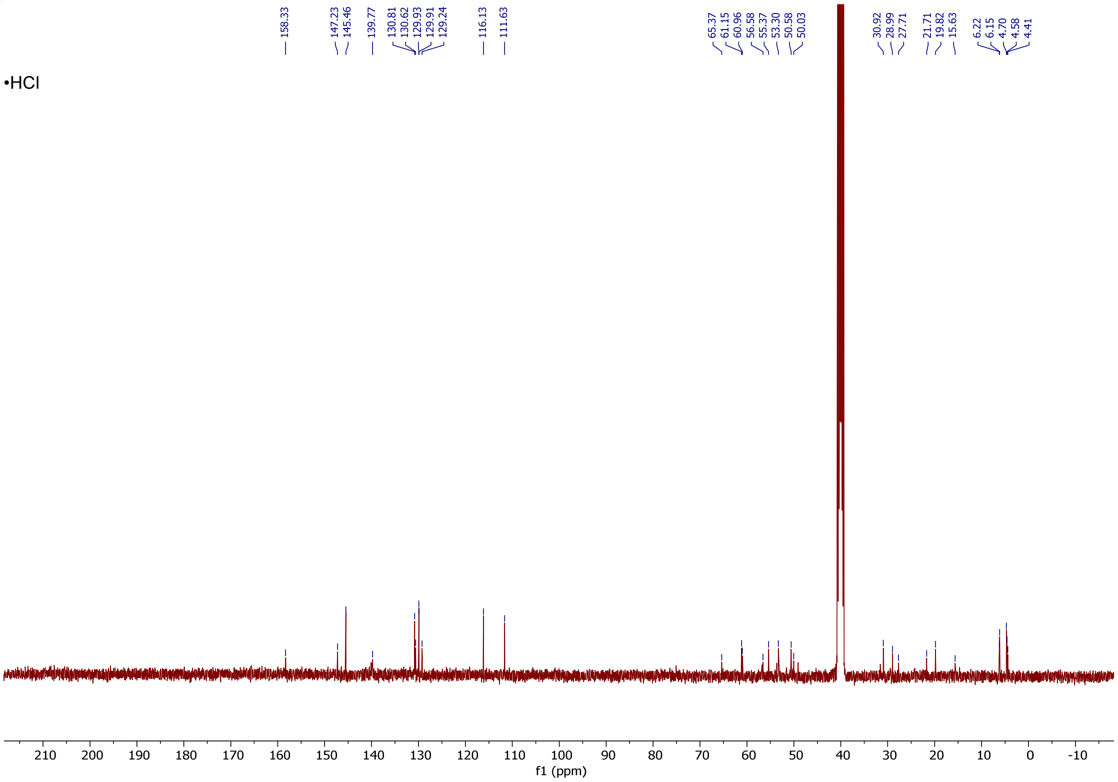

N-(1-(cyclobutylmethyl)azepan-4-yl)-N-phenyl-1H-pyrrole-2-carboxamide hydrogen chloride **(69)**

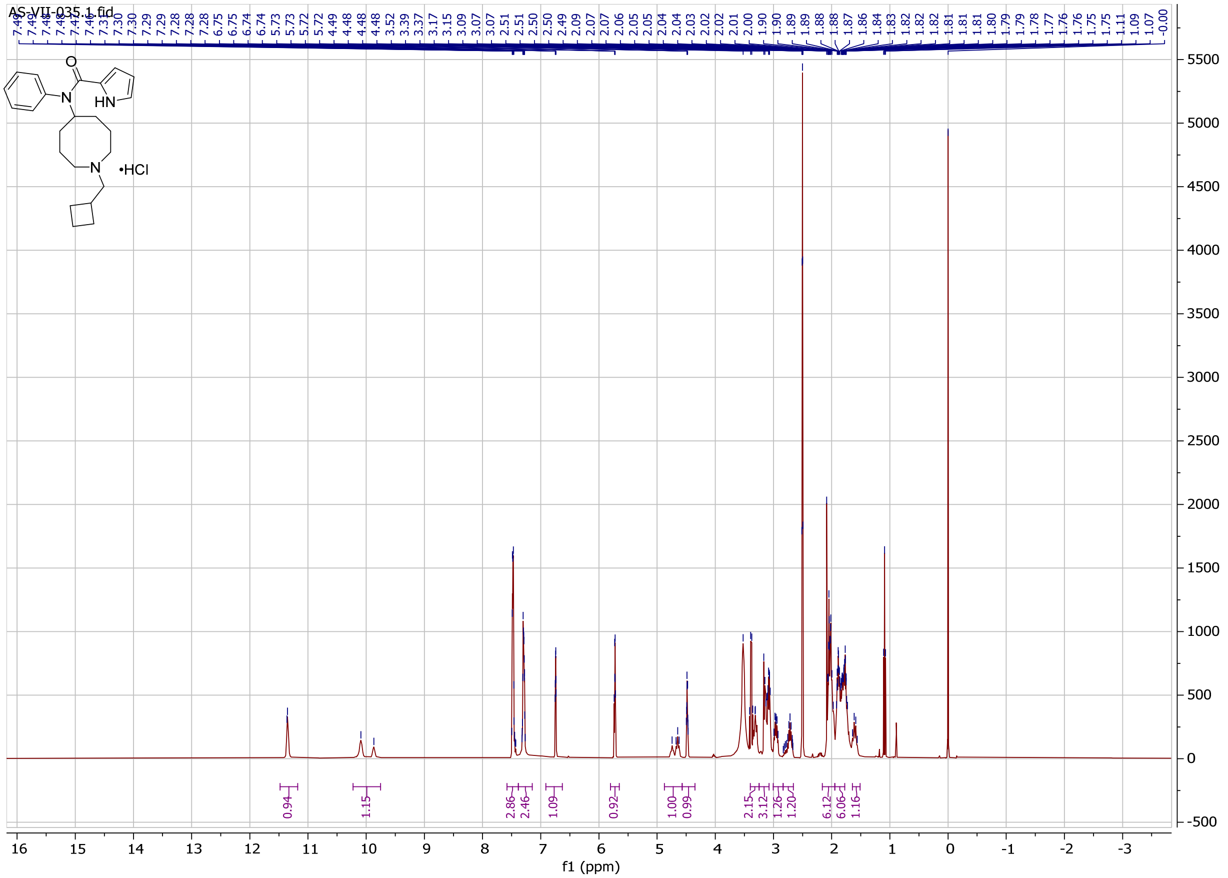

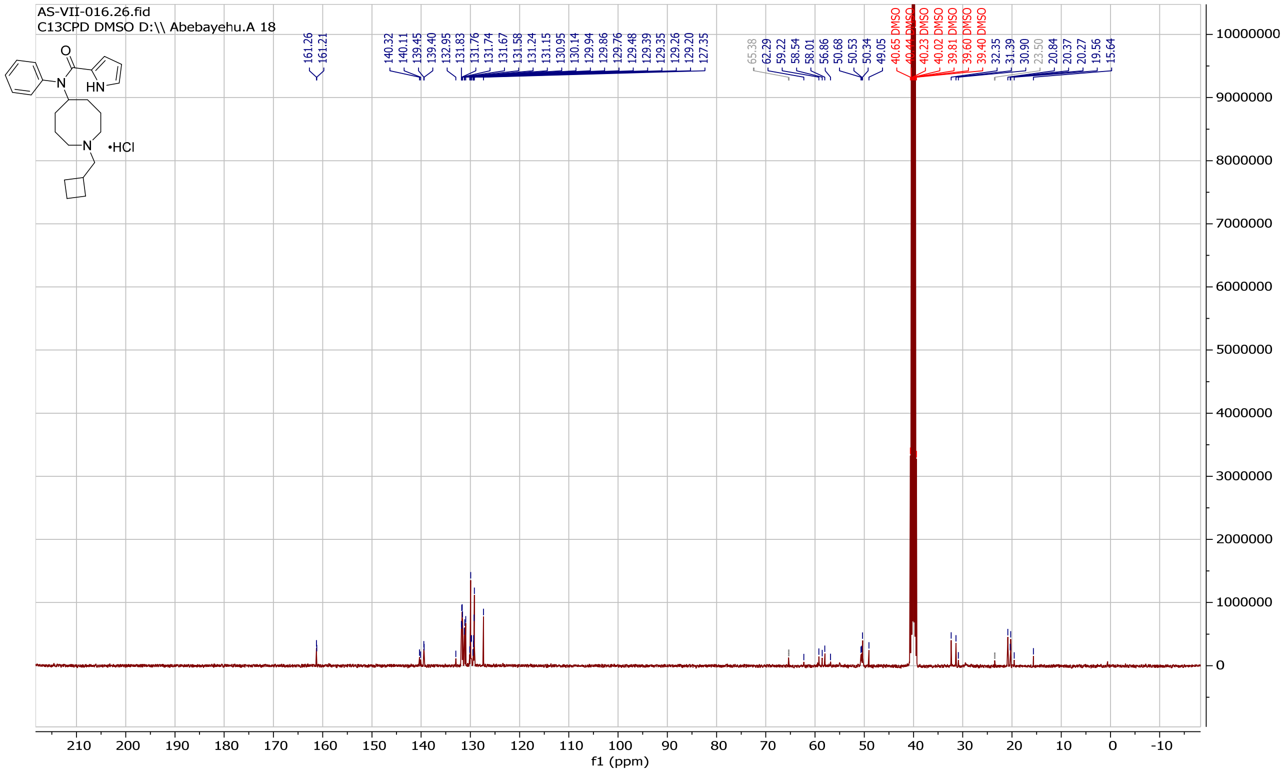

N-(1-(cyclopentylmethyl)azepan-4-yl)-N-phenyl-1H-pyrrole-2-carboxamide hydrogen chloride **(70)**

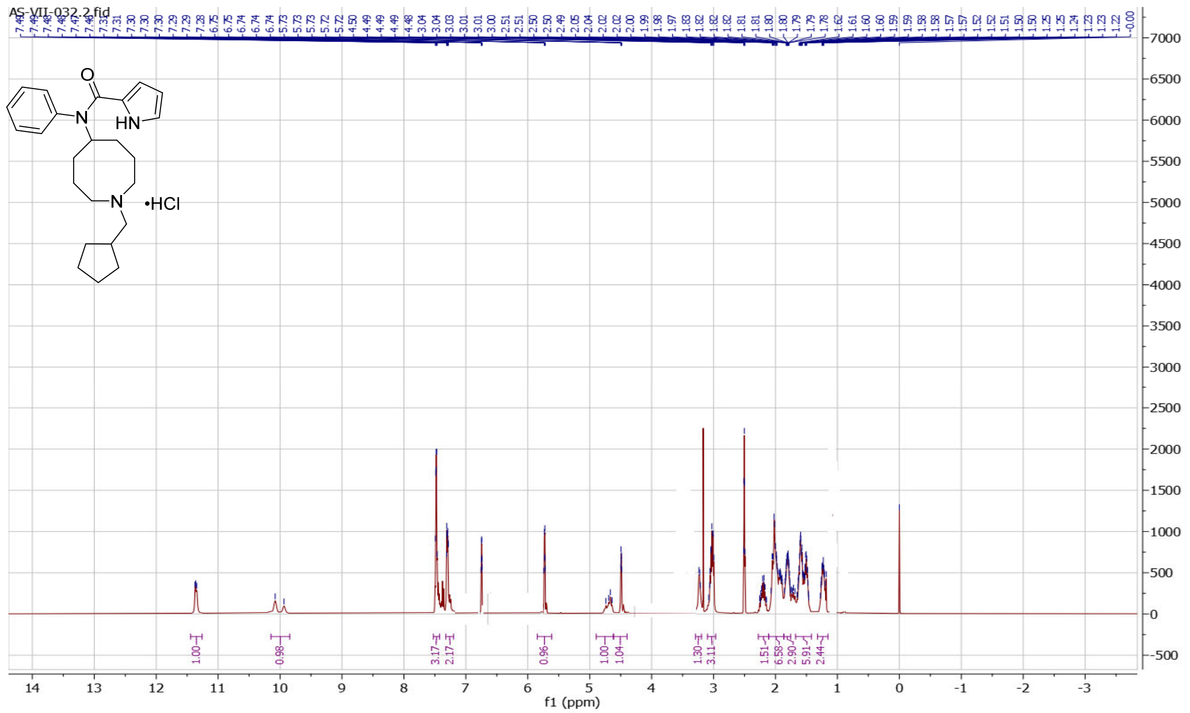

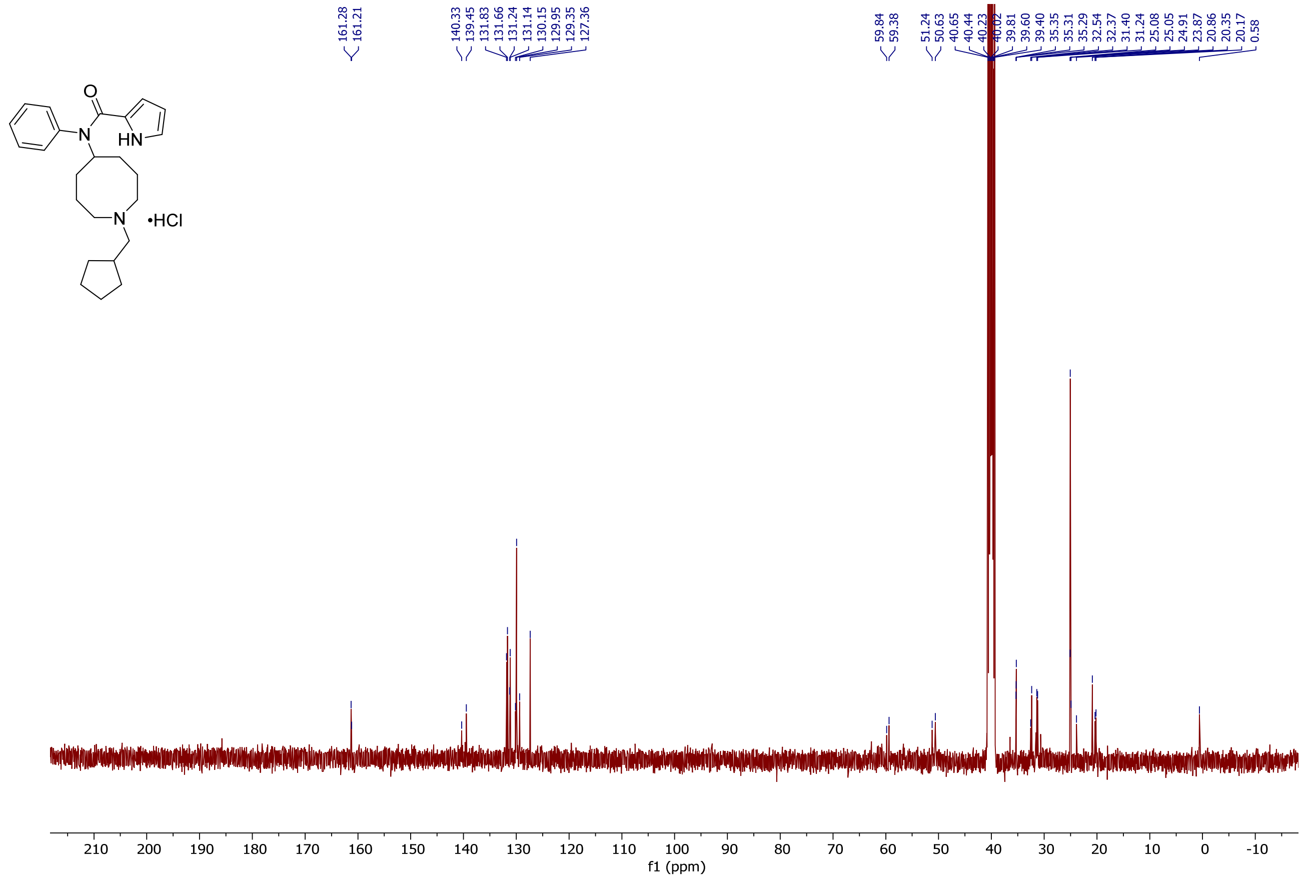

N-(1-(cyclopentylmethyl)azepan-4-yl)-N-phenyl-1H-pyrrole-2-carboxamide hydrogen chloride **(71)**

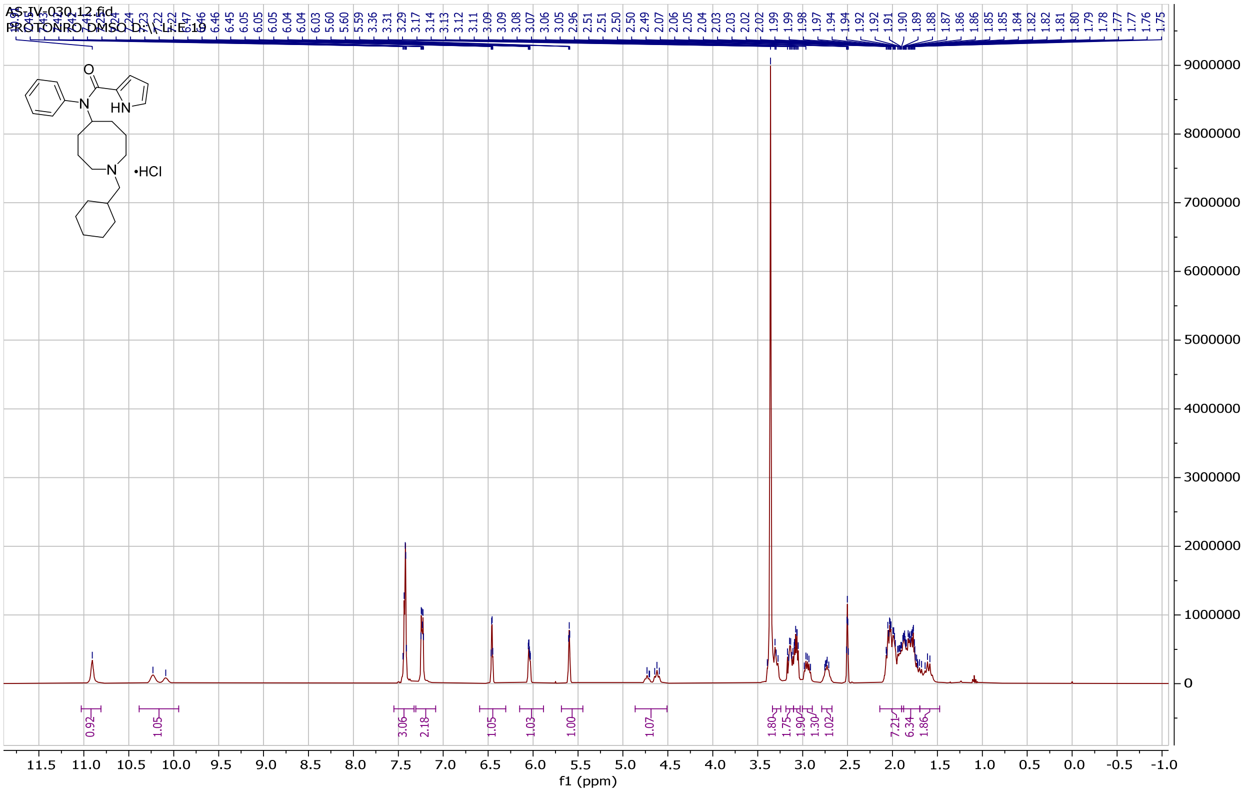

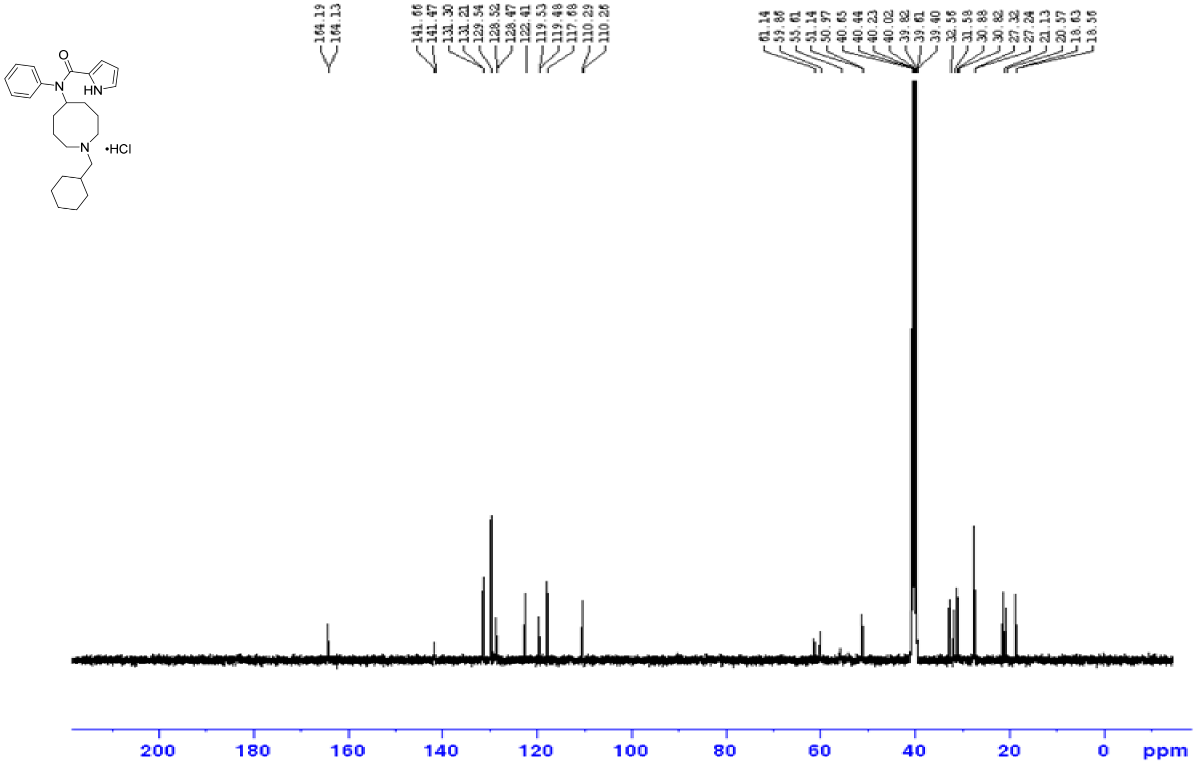

N-(1-benzylazepan-4-yl)-N-phenyl-1H-pyrrole-2-carboxamide hydrogen chloride **(72)**

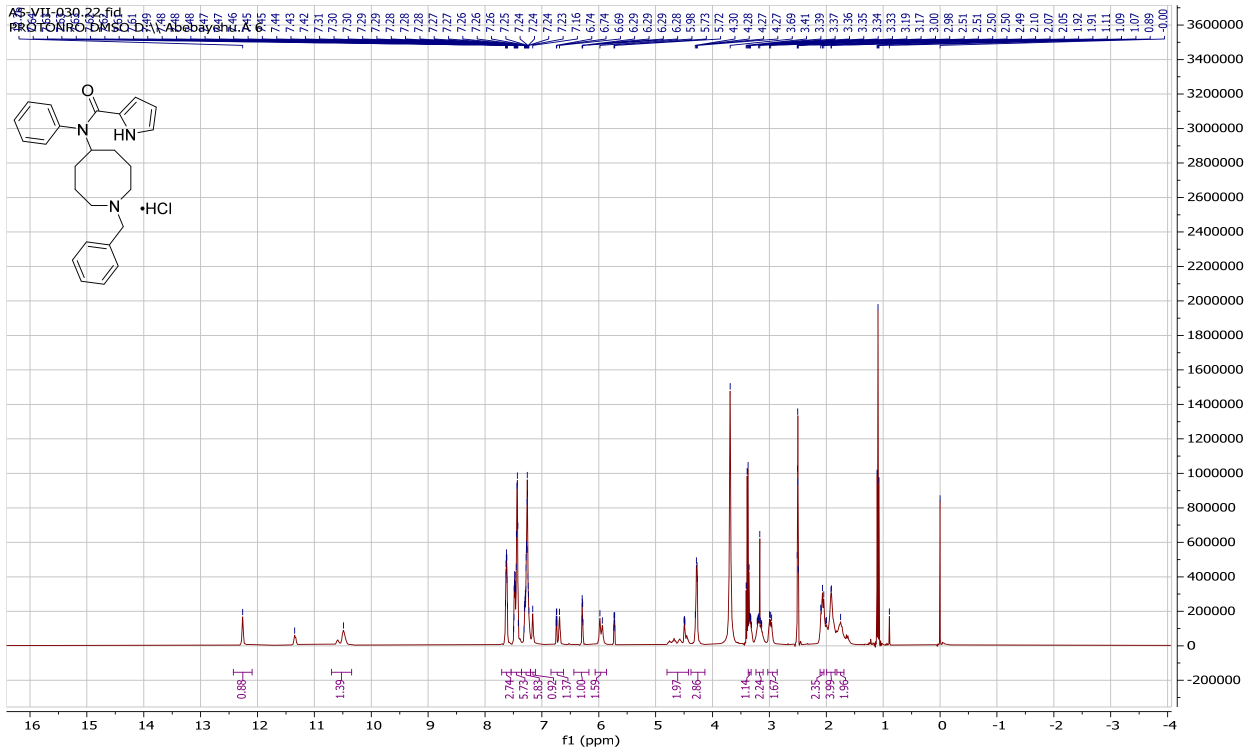

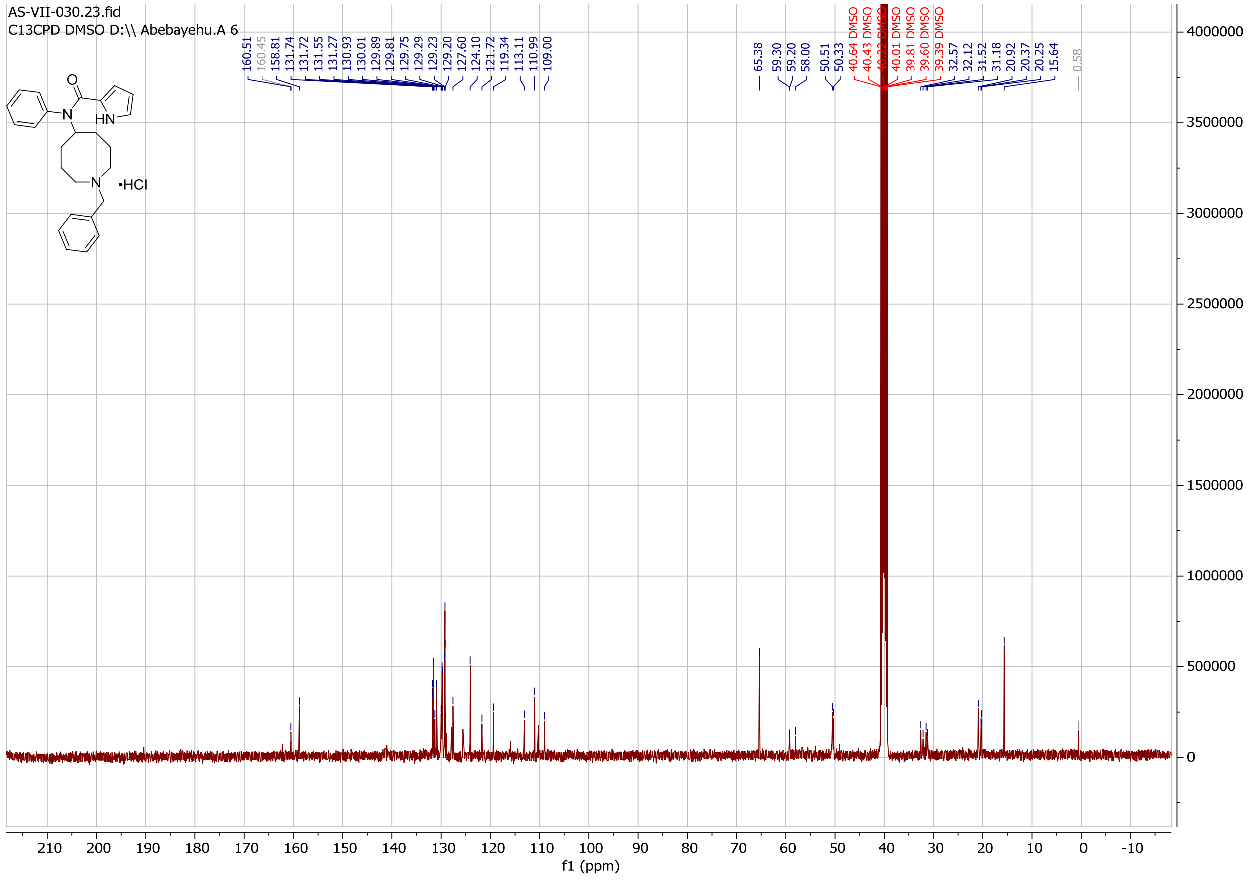

N-(1-(cyclopentylmethyl)azepan-4-yl)-N-phenyl-1H-pyrrole-2-carboxamide hydrogen chloride **(73)**

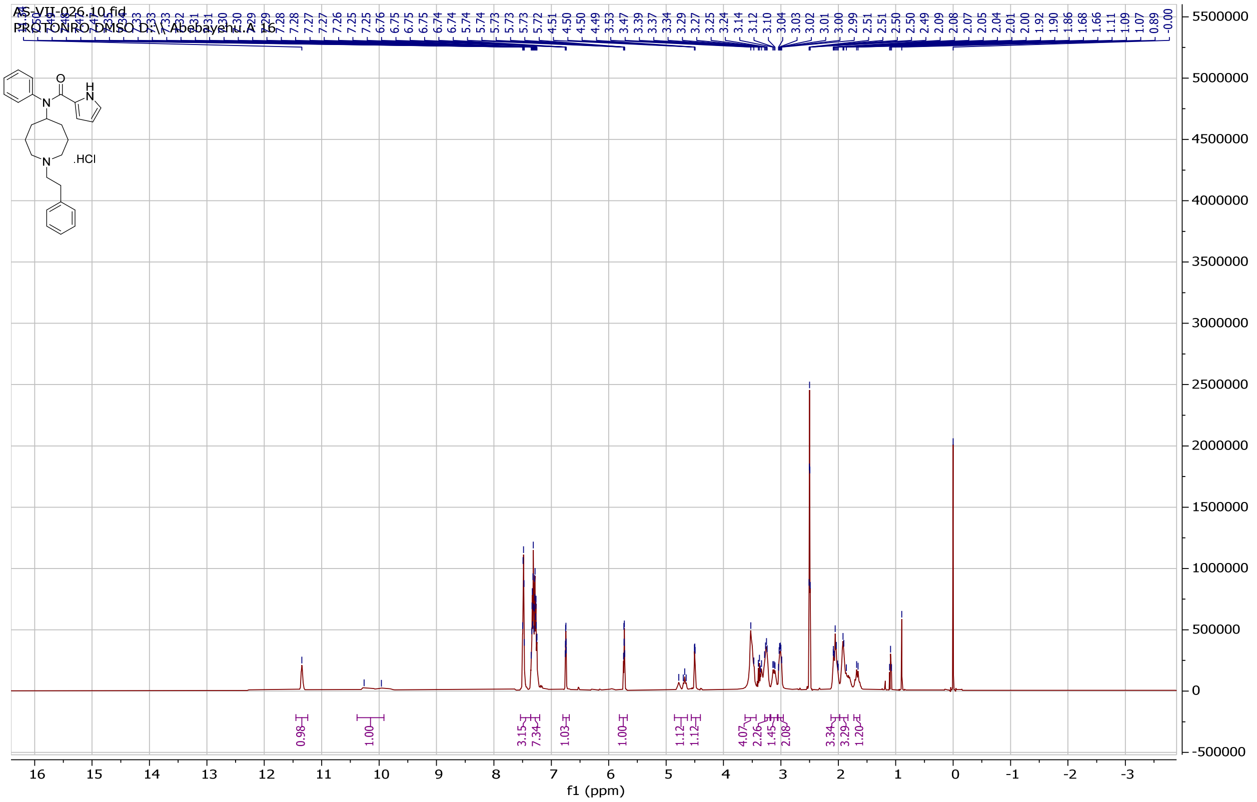

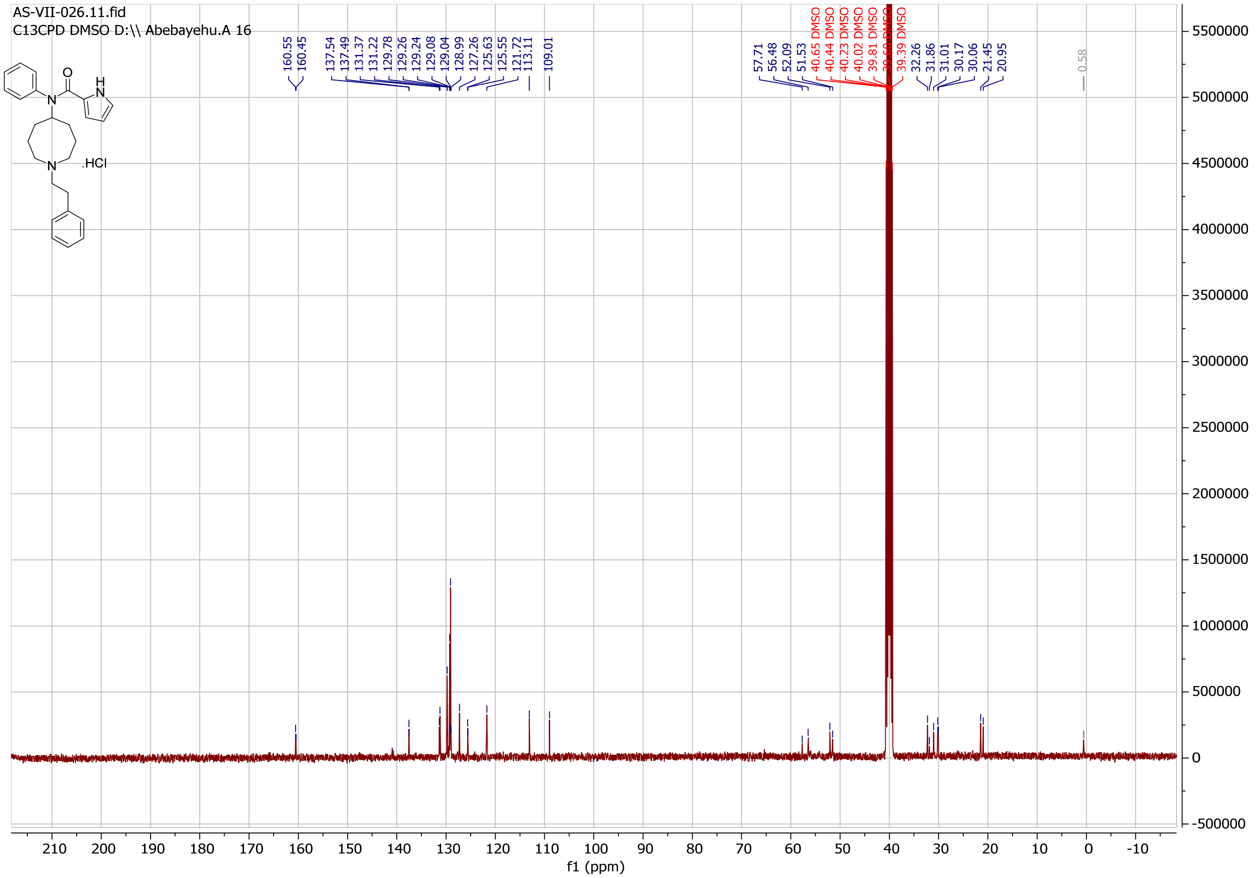

N-(1-allylazocan-5-yl)-N-phenylthiophene-3-carboxamide hydrogen chloride **(74)**

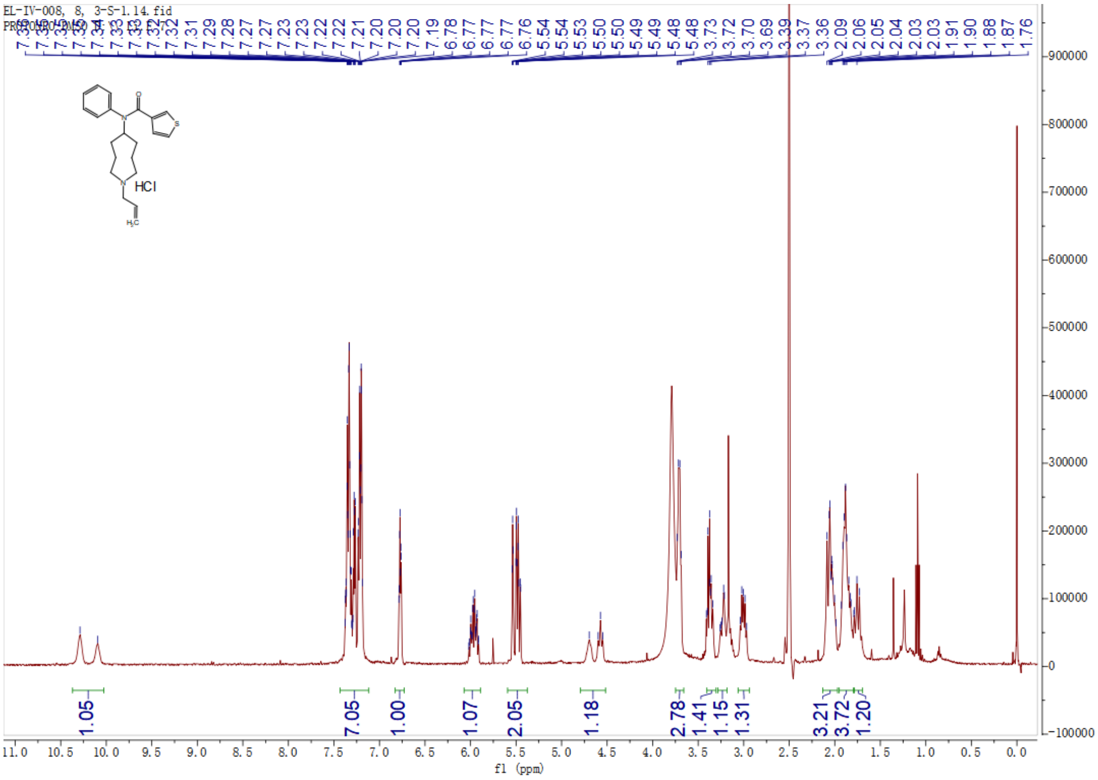

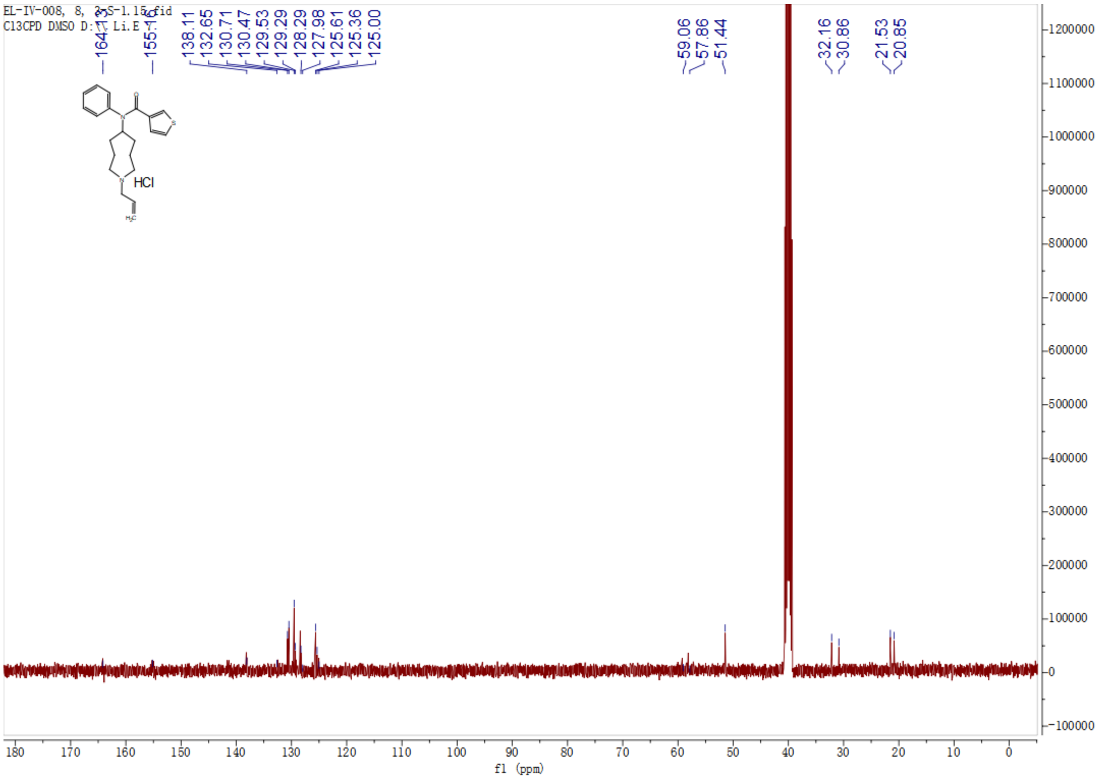

N-(1-(cyclopropylmethyl)azocan-5-yl)-N-phenylthiophene-3-carboxamide hydrogen chloride **(75)**

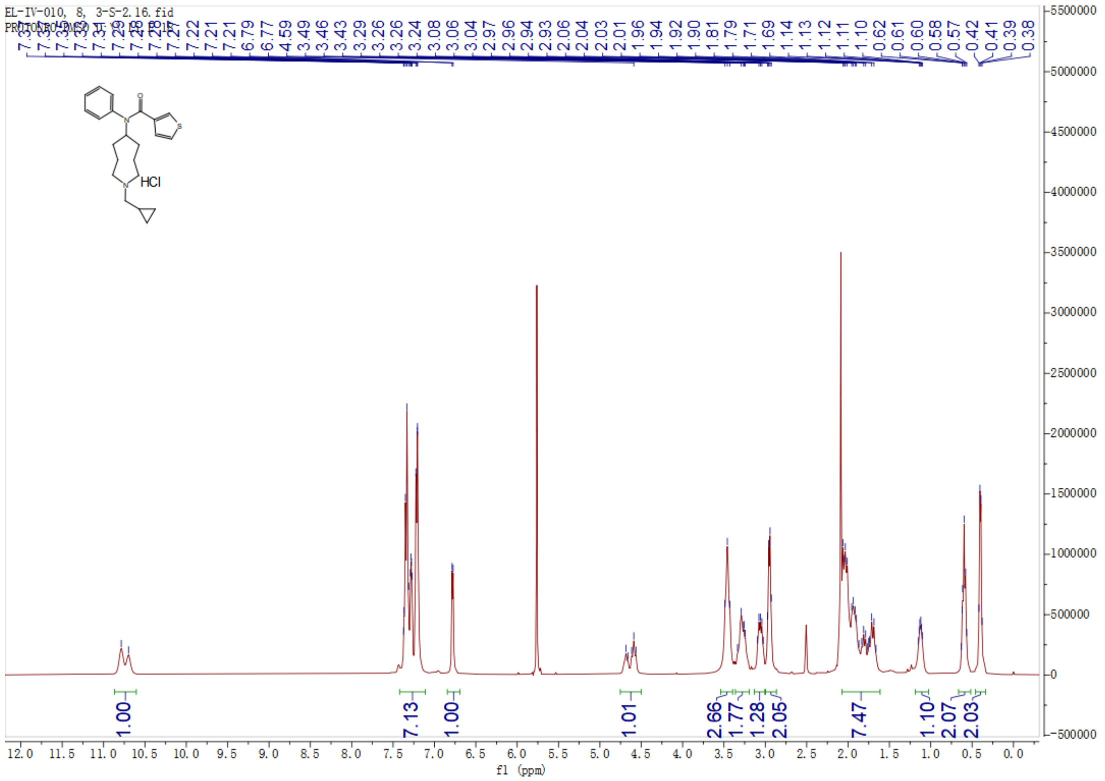

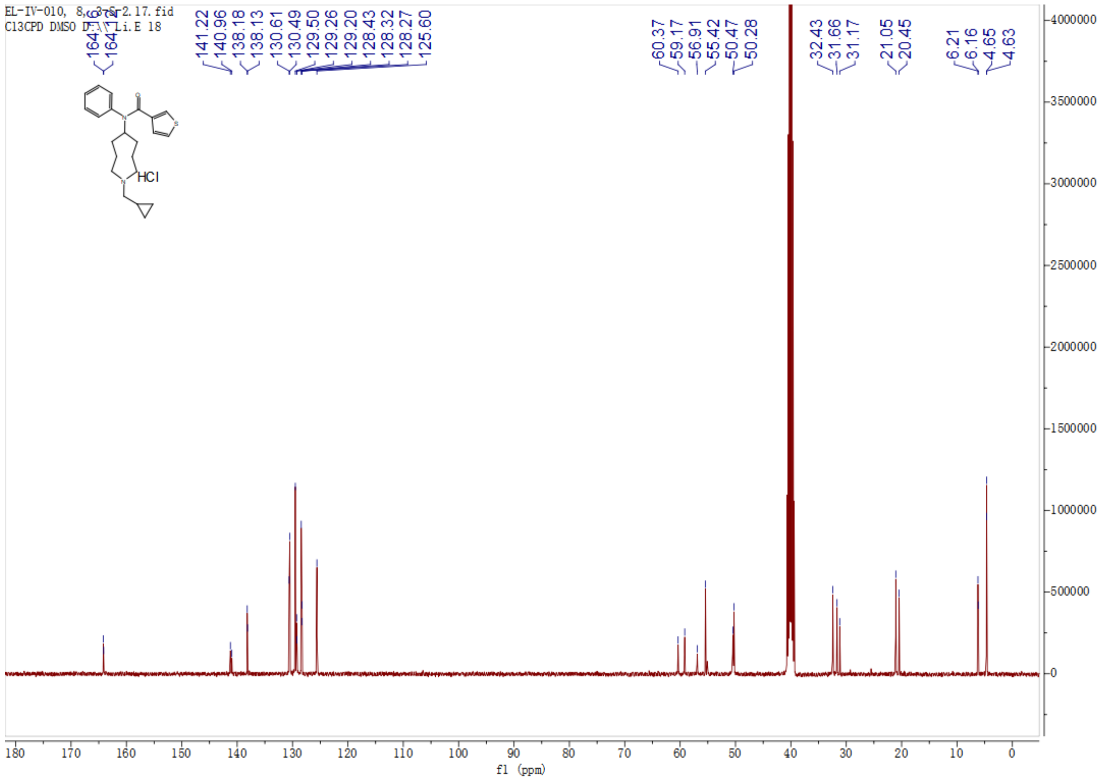

N-(1-(cyclobutylmethyl)azocan-5-yl)-N-phenylthiophene-3-carboxamide hydrogen chloride **(76)**

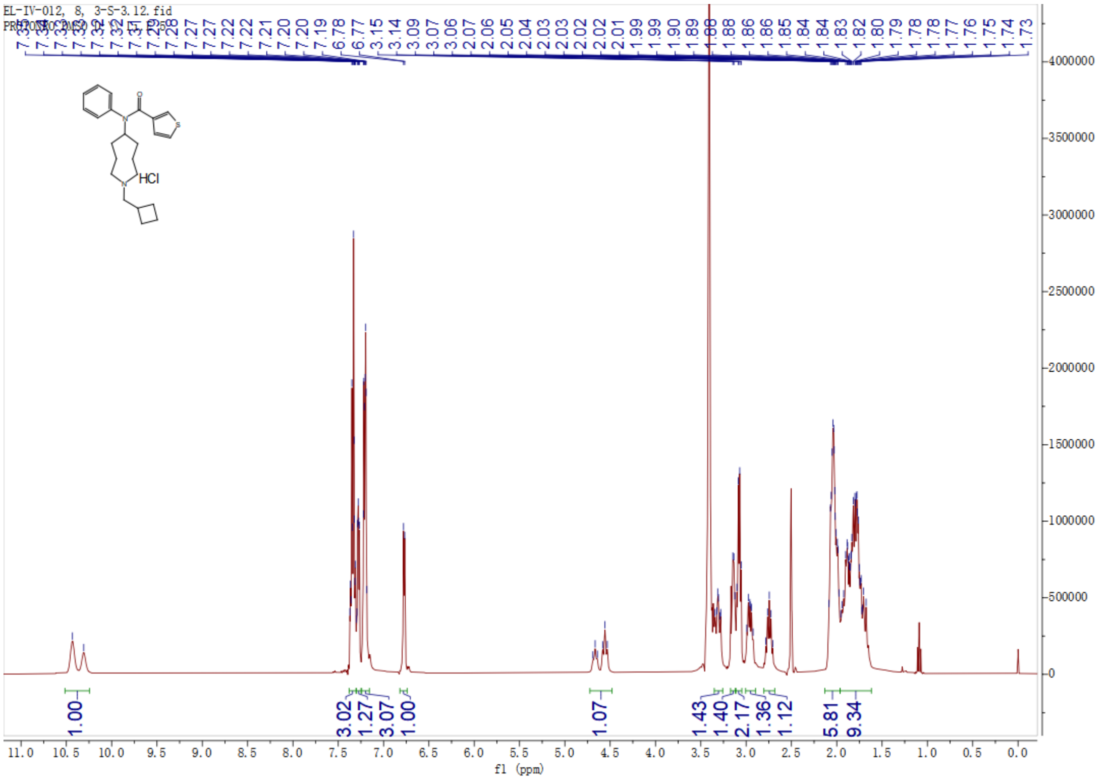

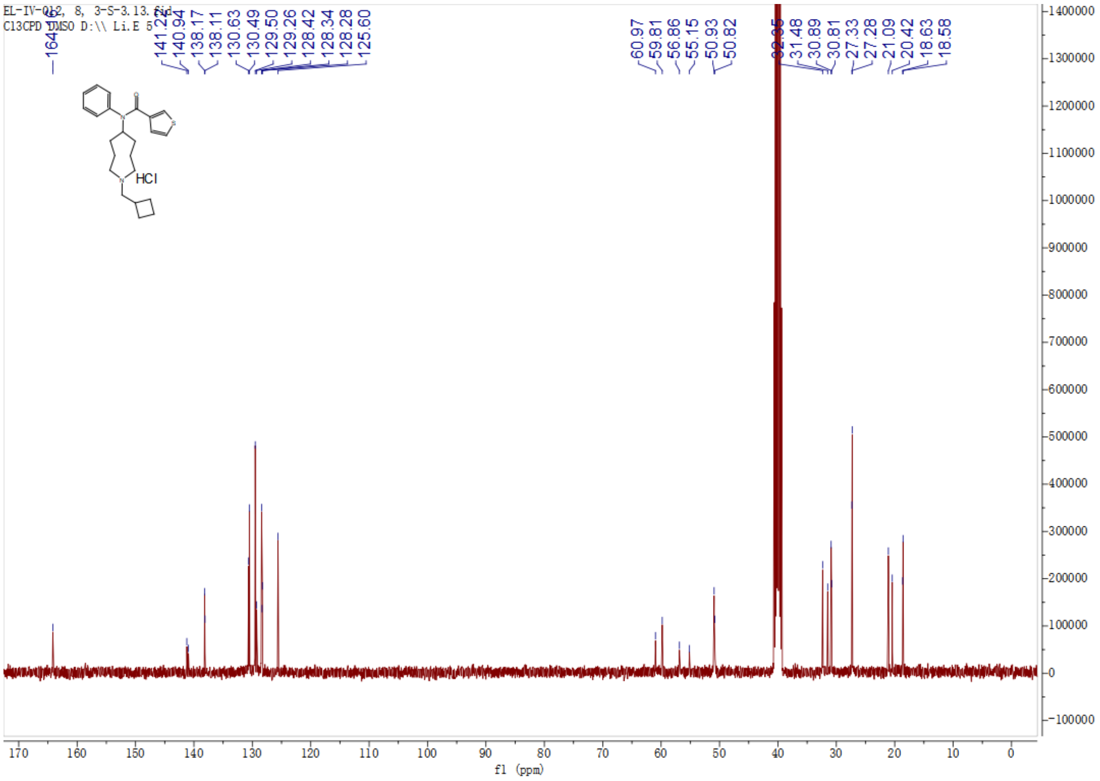

N-(1-(cyclopentylmethyl)azocan-5-yl)-N-phenylthiophene-3-carboxamide hydrogen chloride **(77)**

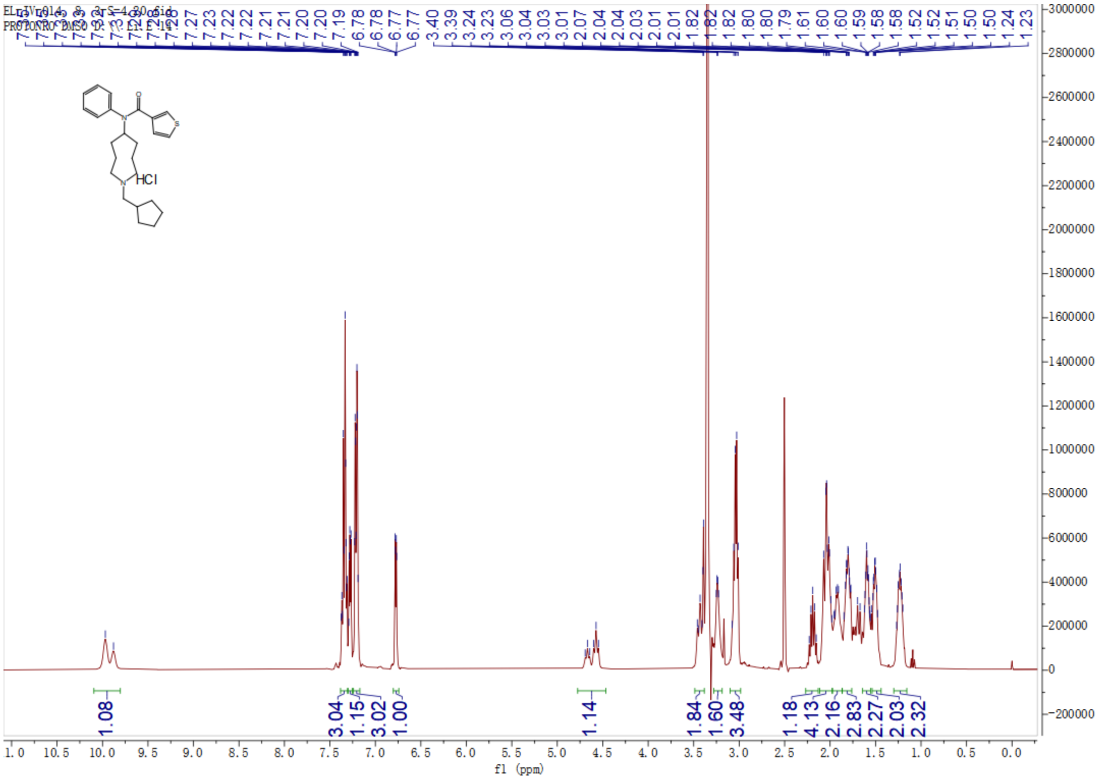

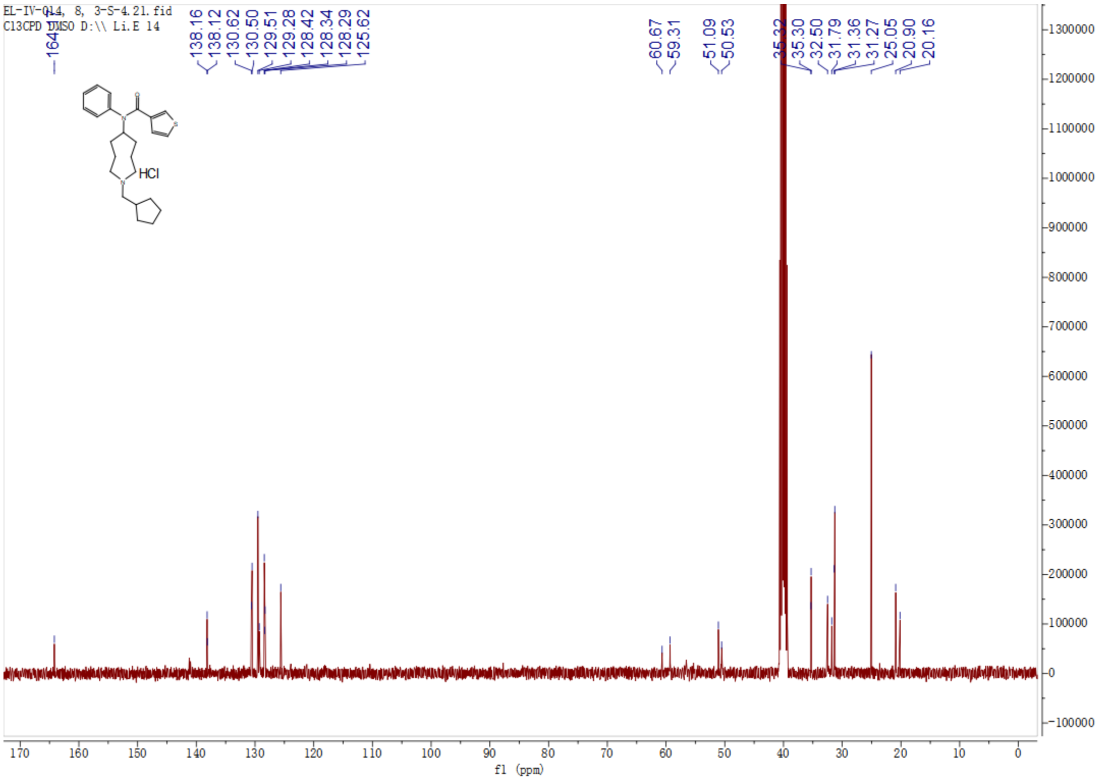

N-(1-(cyclohexylmethyl)azocan-5-yl)-N-phenylthiophene-3-carboxamide hydrogen chloride **(78)**

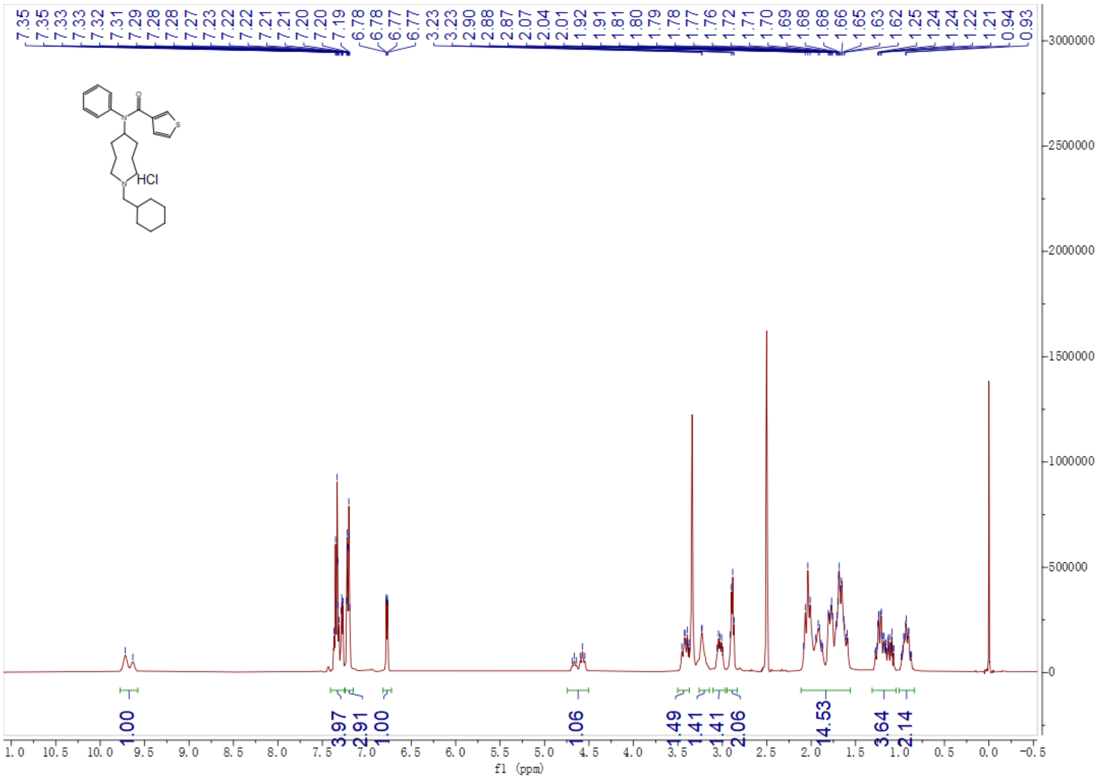

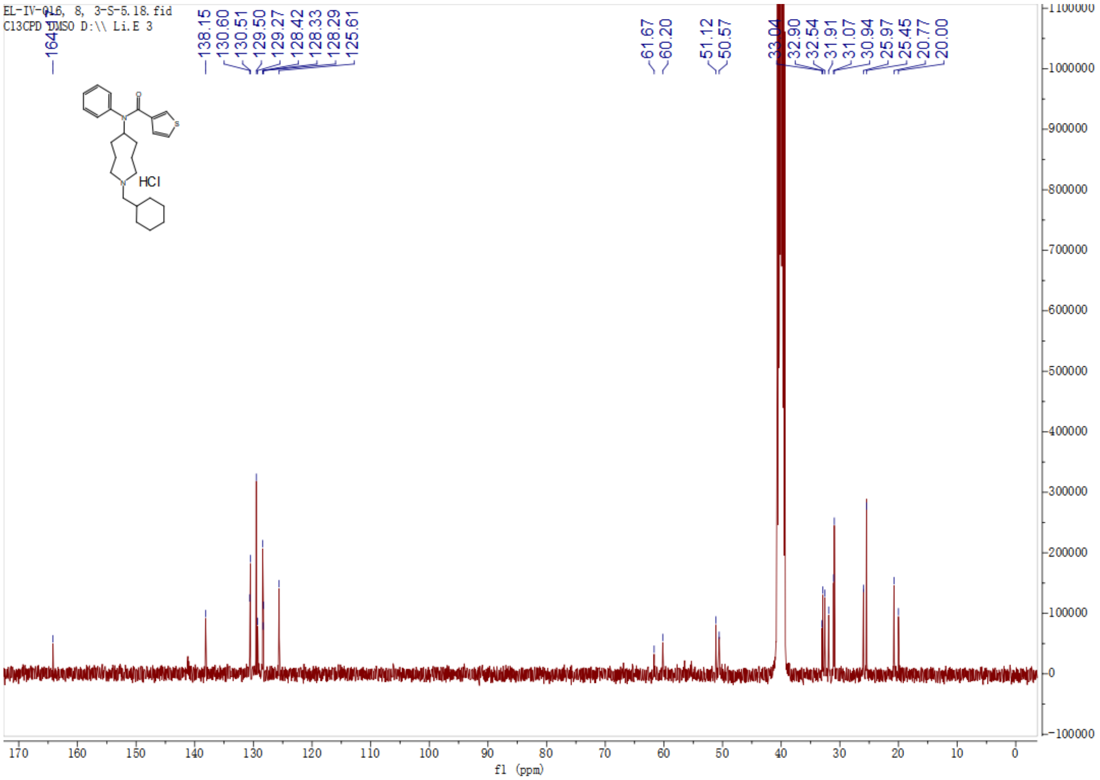

N-(1-benzylazocan-5-yl)-N-phenylthiophene-3-carboxamide hydrogen chloride **(79)**

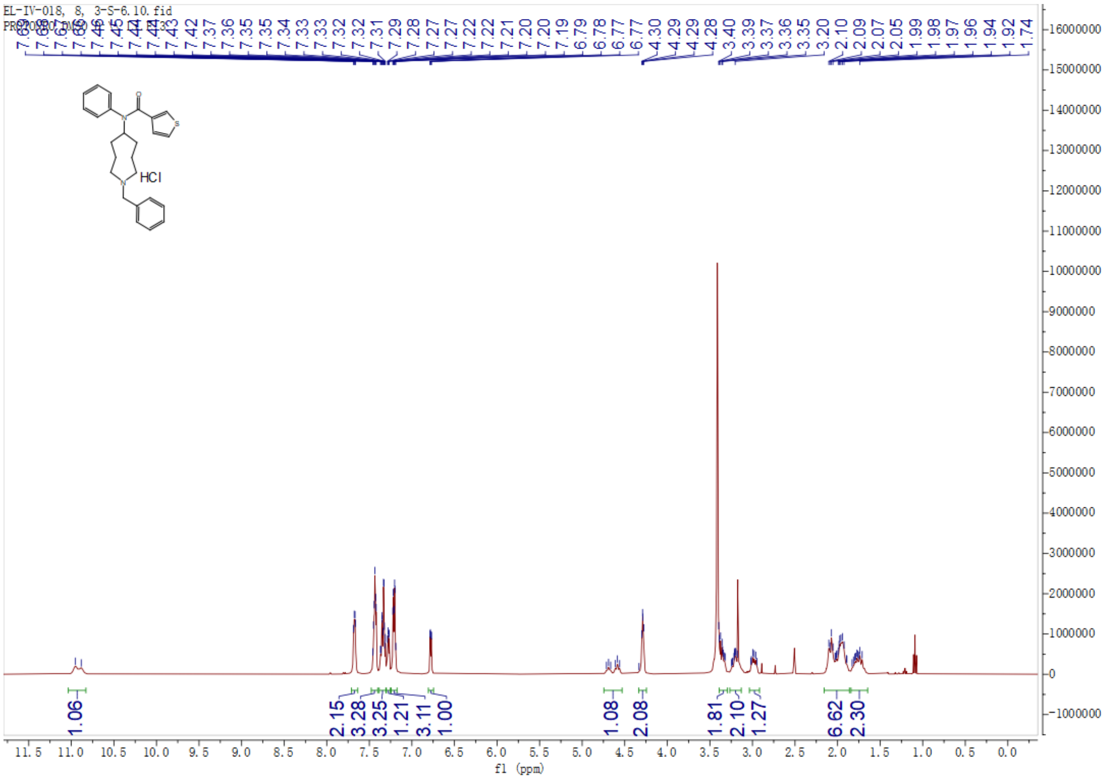

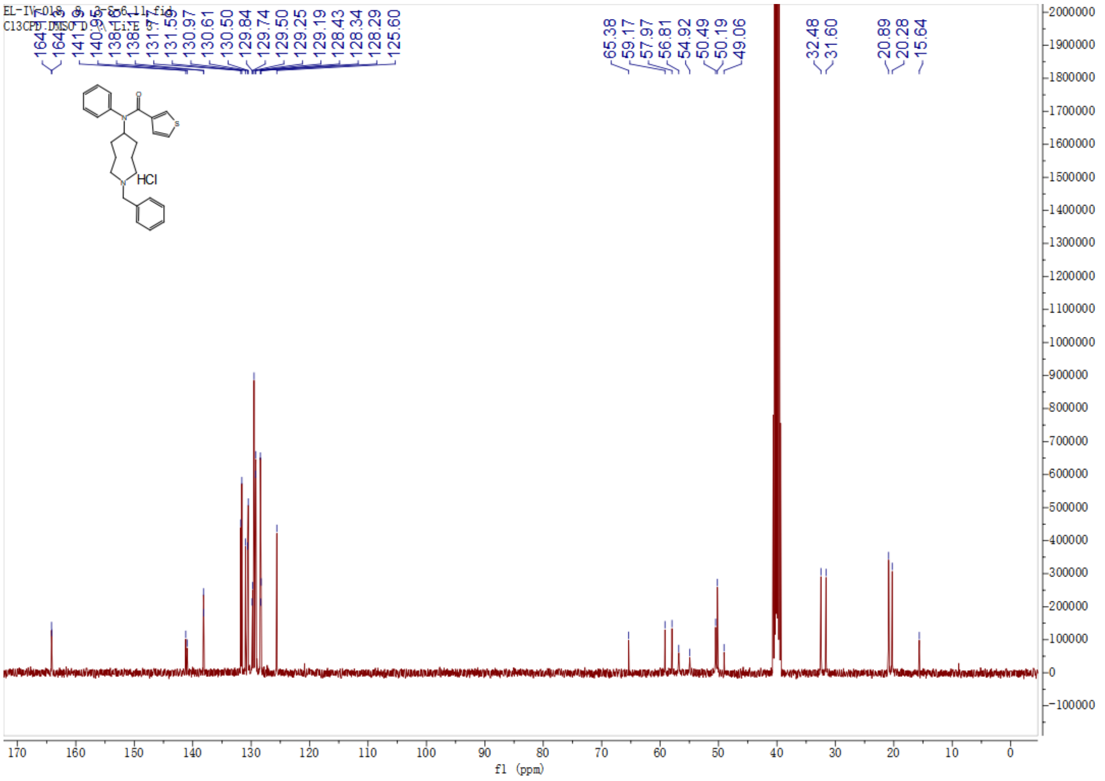

N-(1-phenethylazocan-5-yl)-N-phenylthiophene-3-carboxamide hydrogen chloride **(80)**

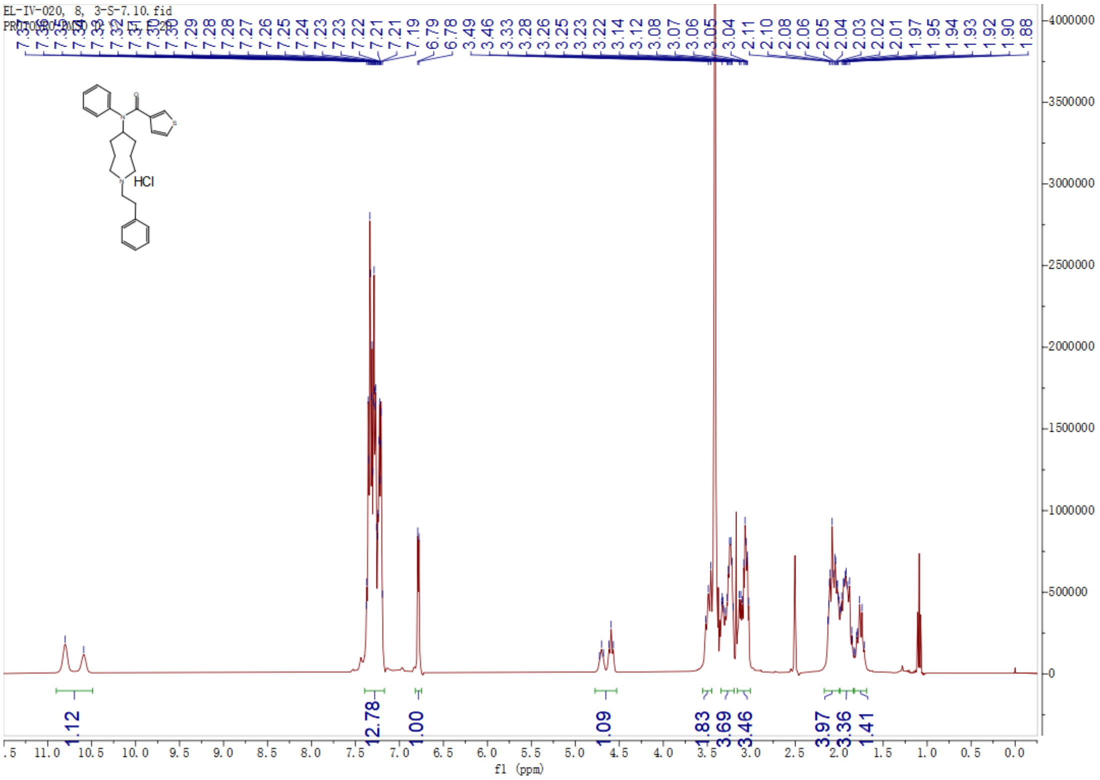

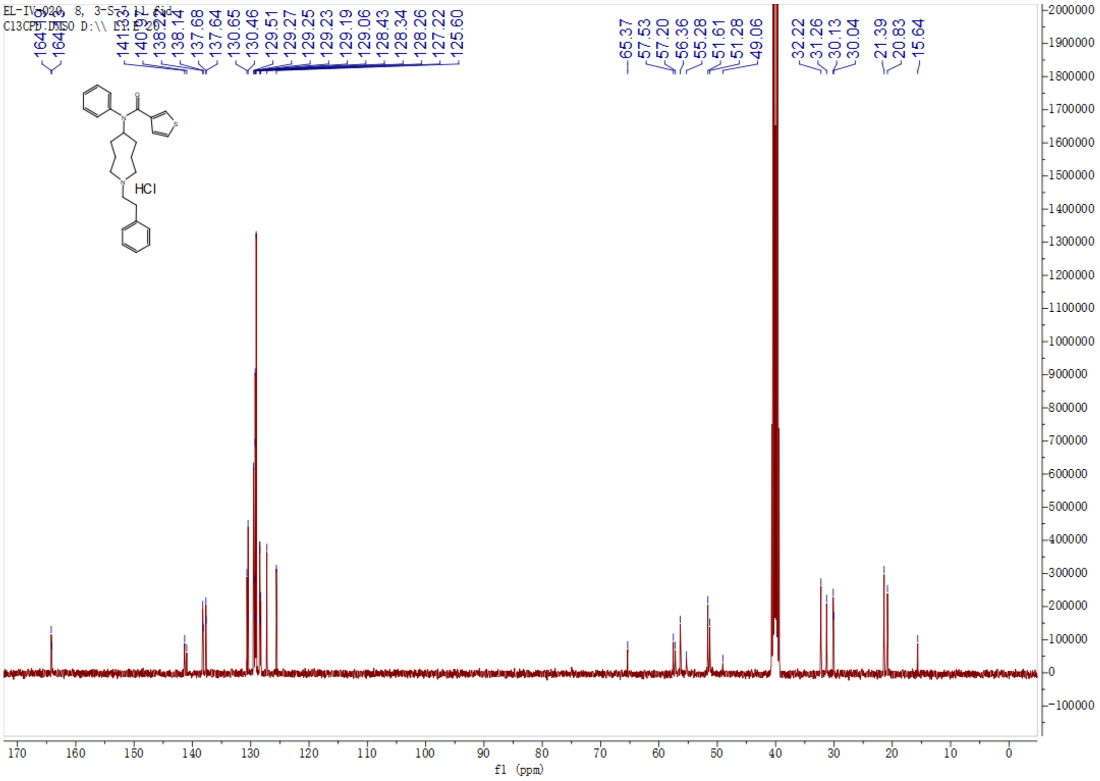

N-(1-allylazocan-5-yl)-N-phenyl-1H-pyrrole-3-carboxamide hydrogen chloride **(81)**

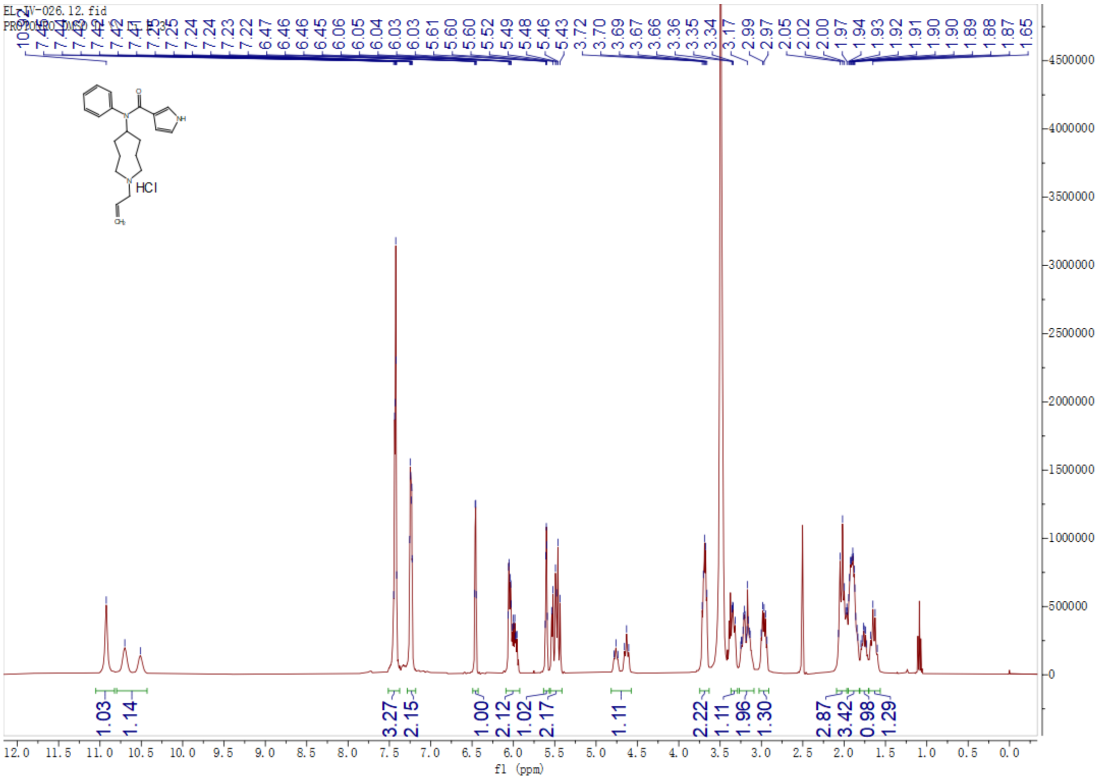

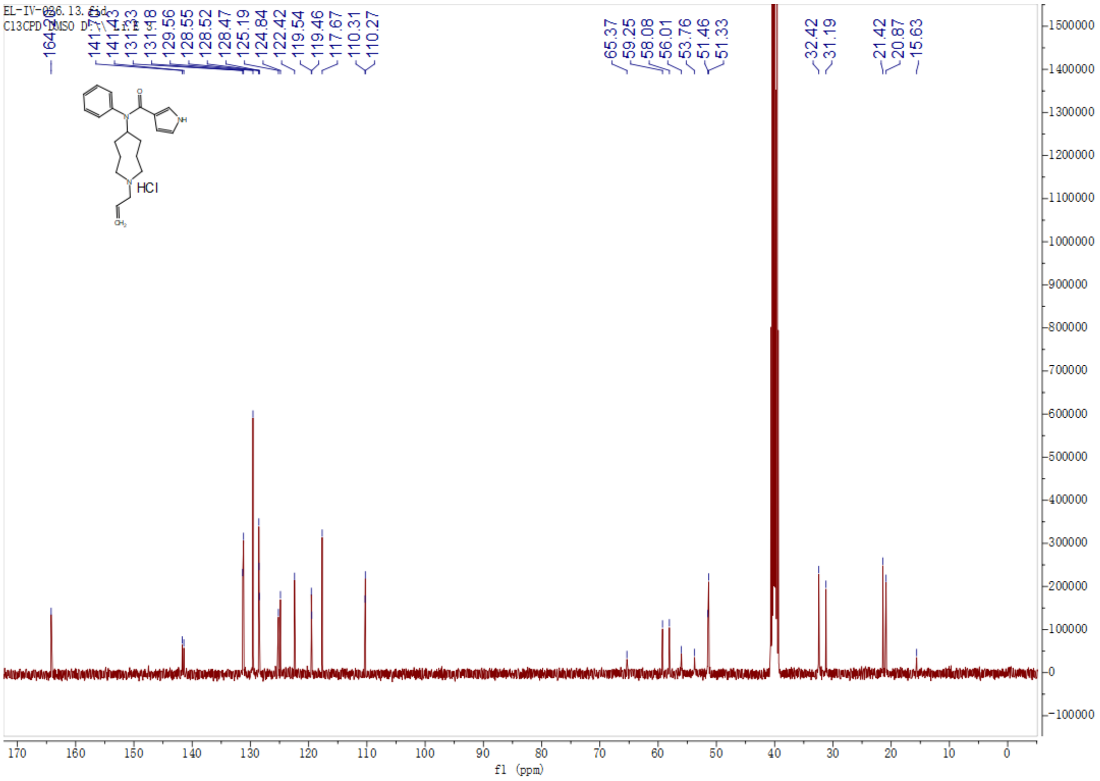

N-(1-(cyclopropylmethyl)azocan-5-yl)-N-phenyl-1H-pyrrole-3-carboxamide hydrogen chloride **(82)**

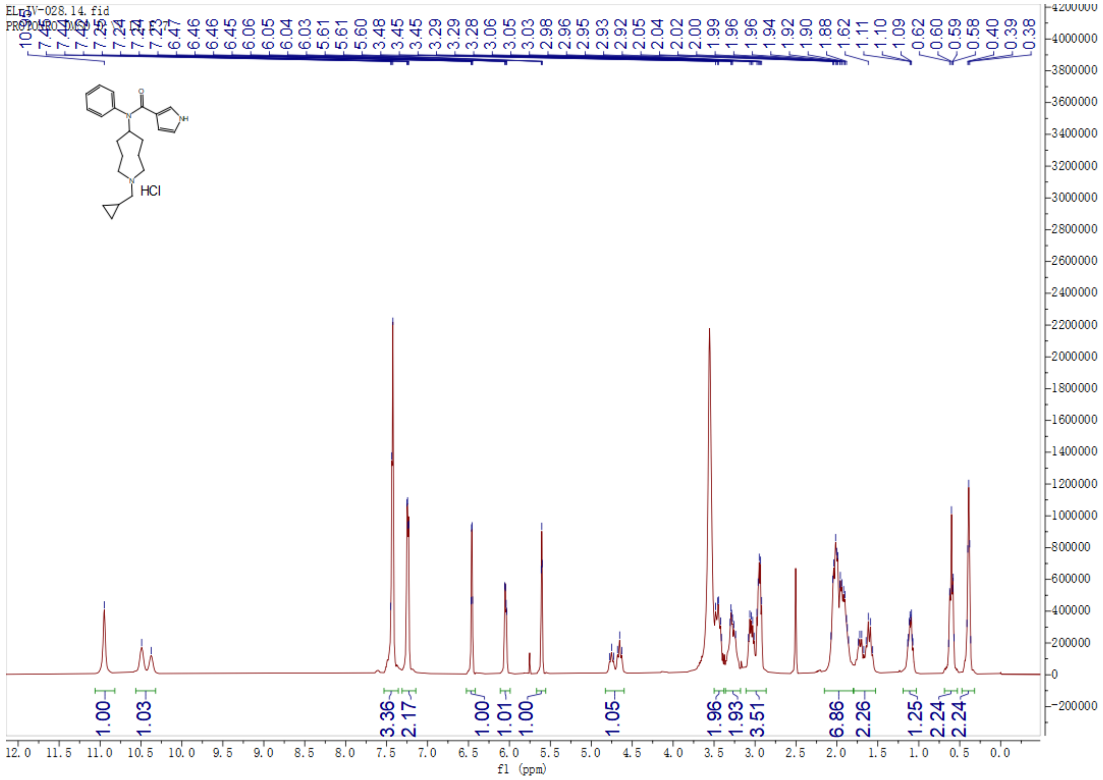

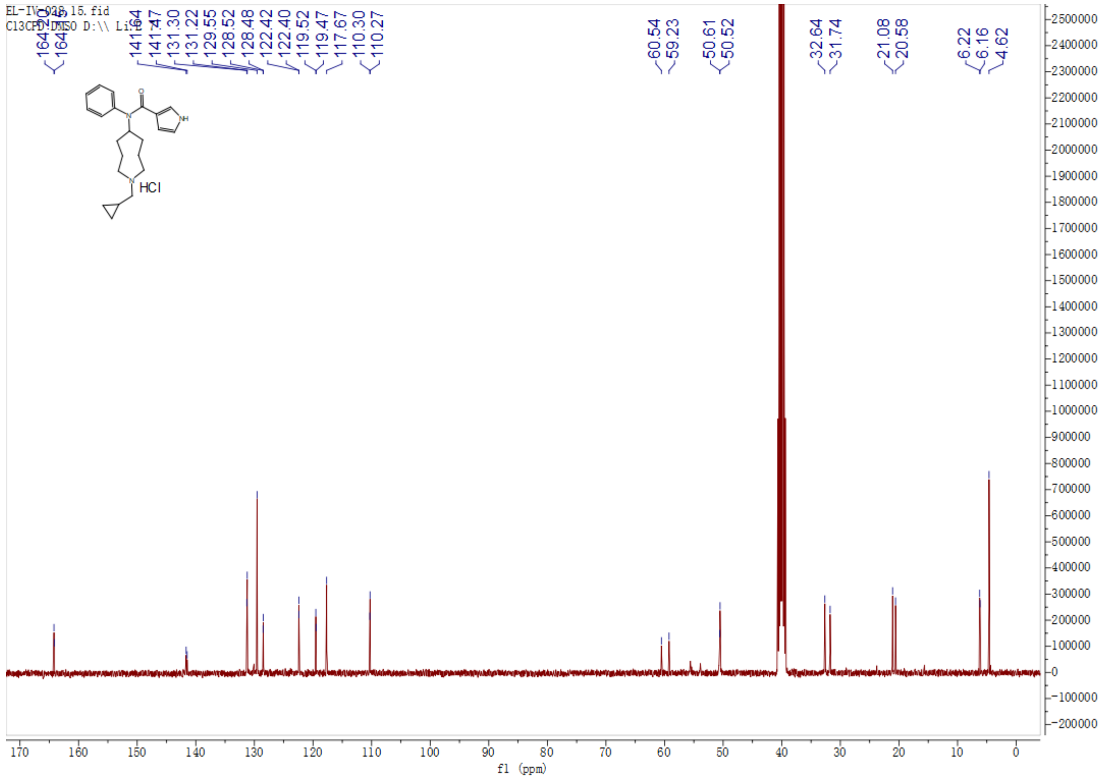

N-(1-(cyclobutylmethyl)azocan-5-yl)-N-phenyl-1H-pyrrole-3-carboxamide hydrogen chloride **(83)**

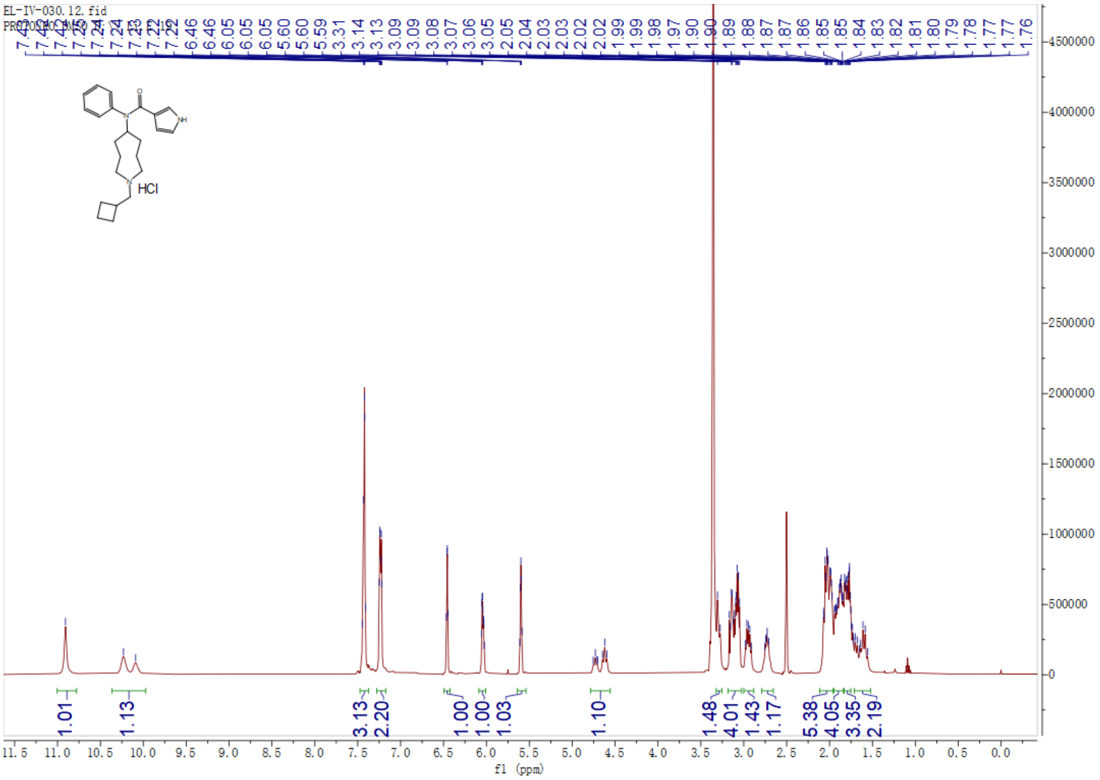

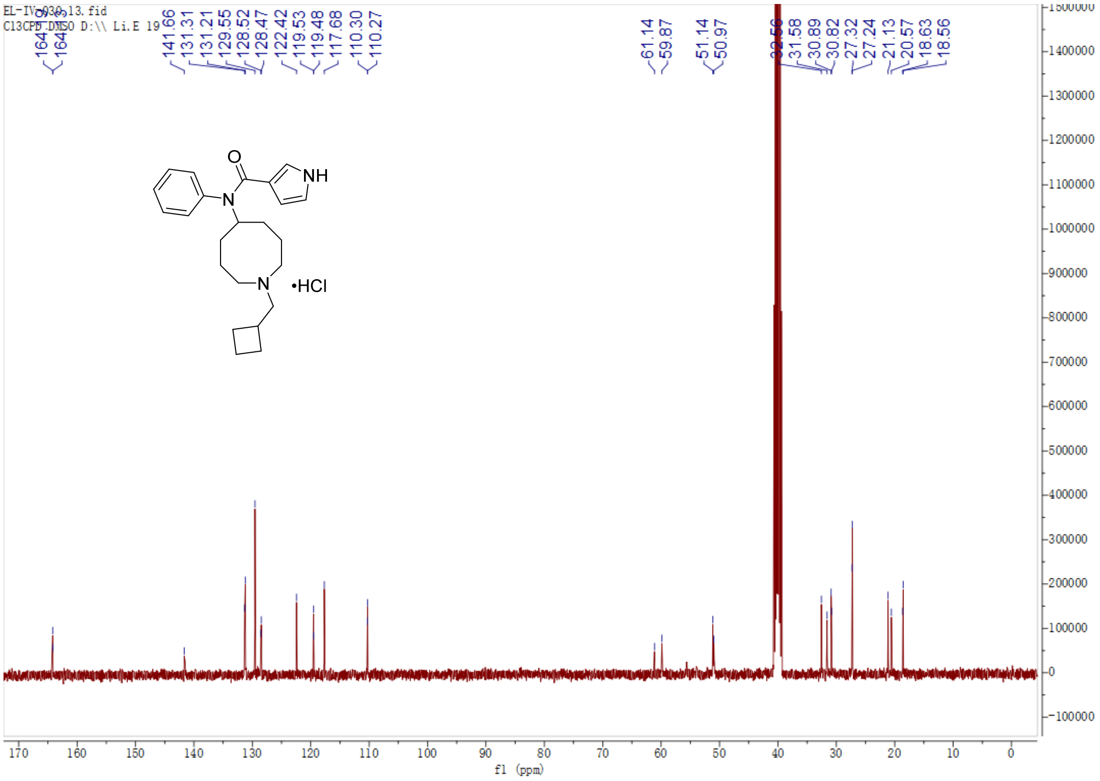

N-(1-(cyclopentylmethyl)azocan-5-yl)-N-phenyl-1H-pyrrole-3-carboxamide hydrogen chloride **(84)**

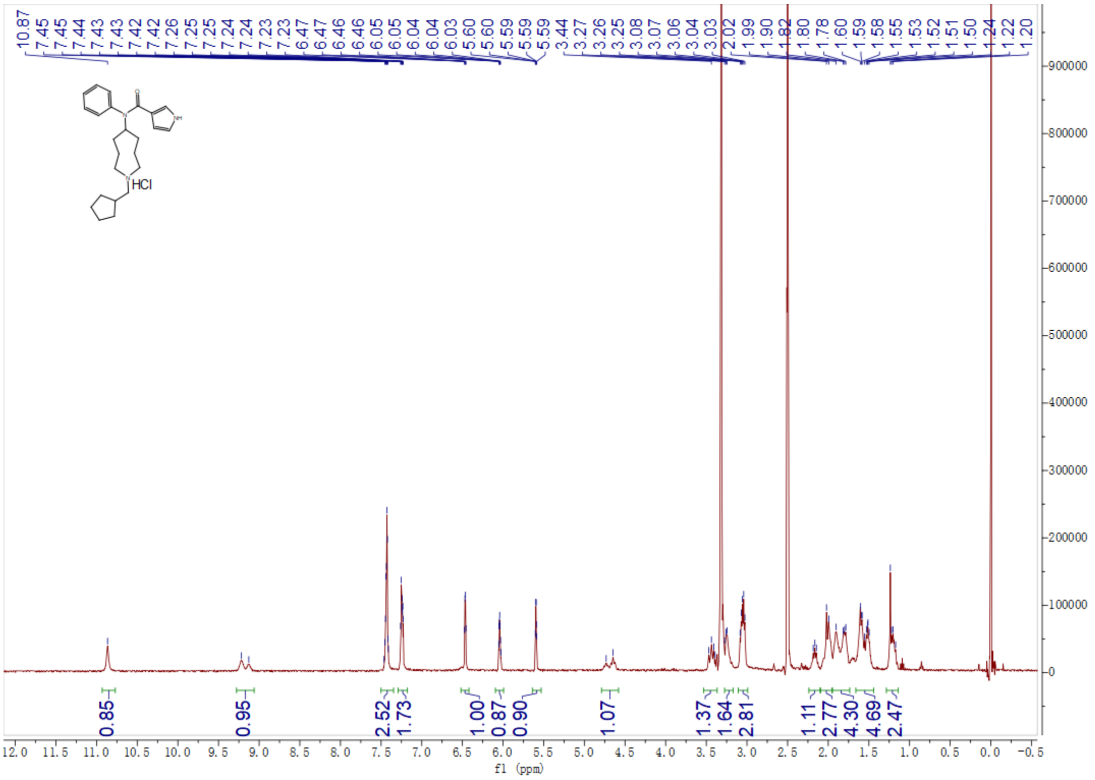

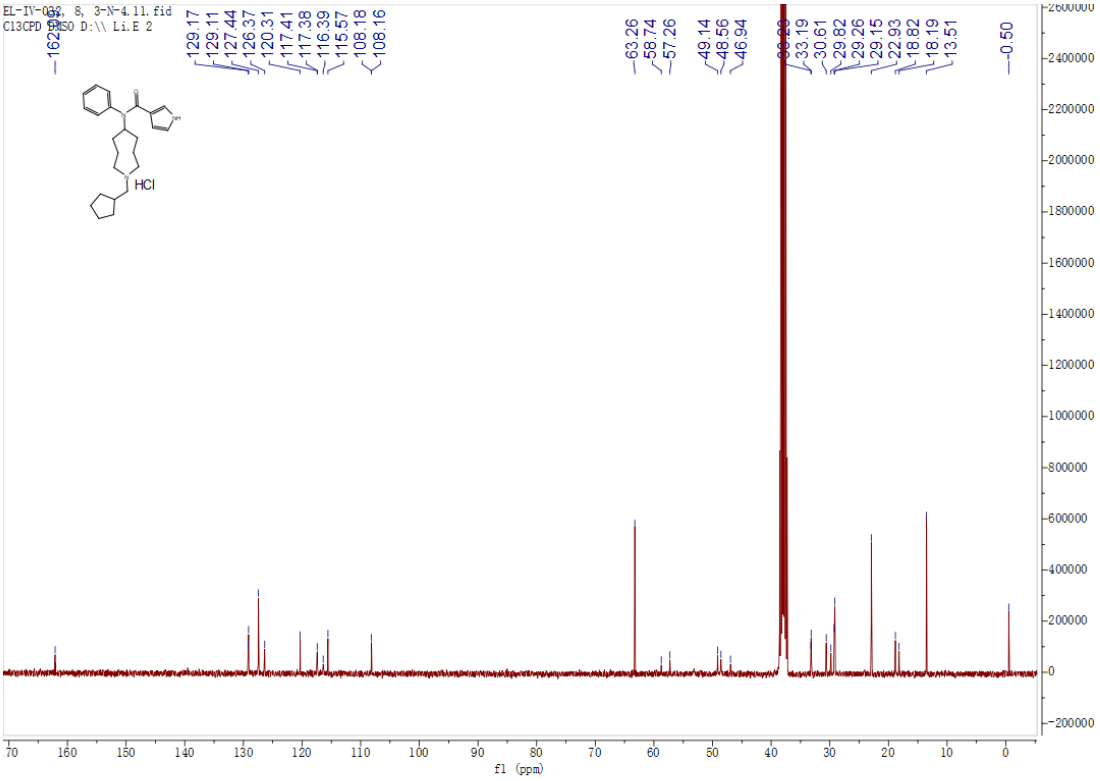

N-(1-(cyclohexylmethyl)azocan-5-yl)-N-phenyl-1H-pyrrole-3-carboxamide hydrogen chloride **(85)**

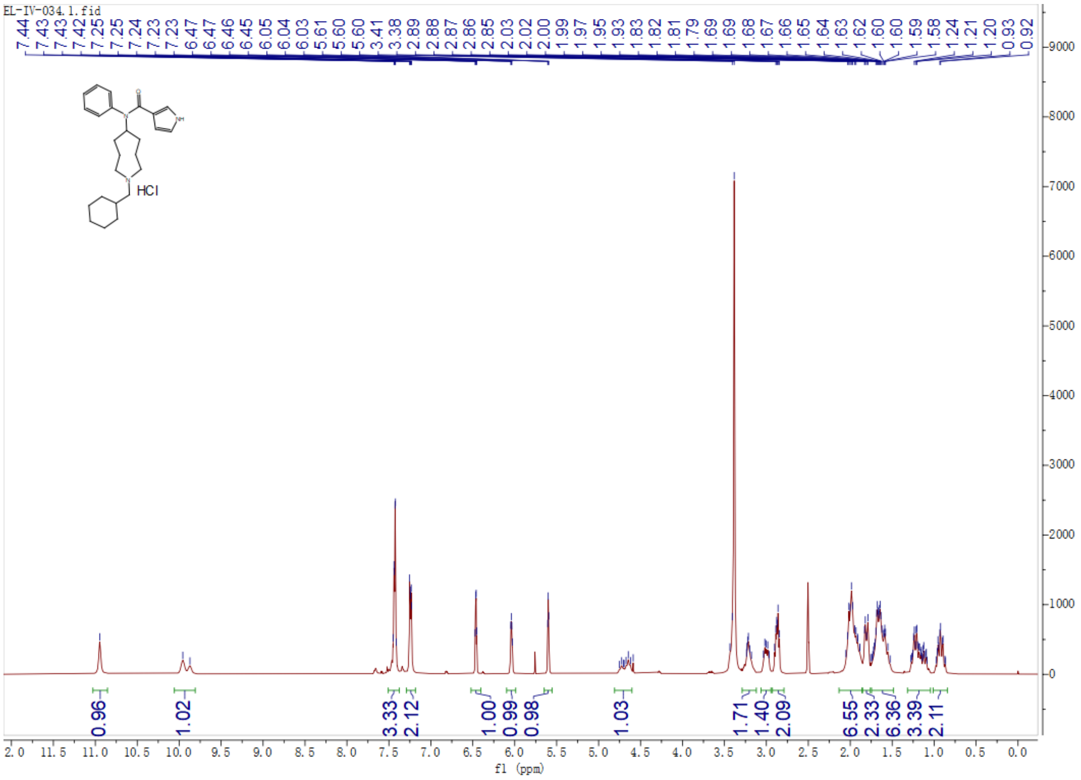

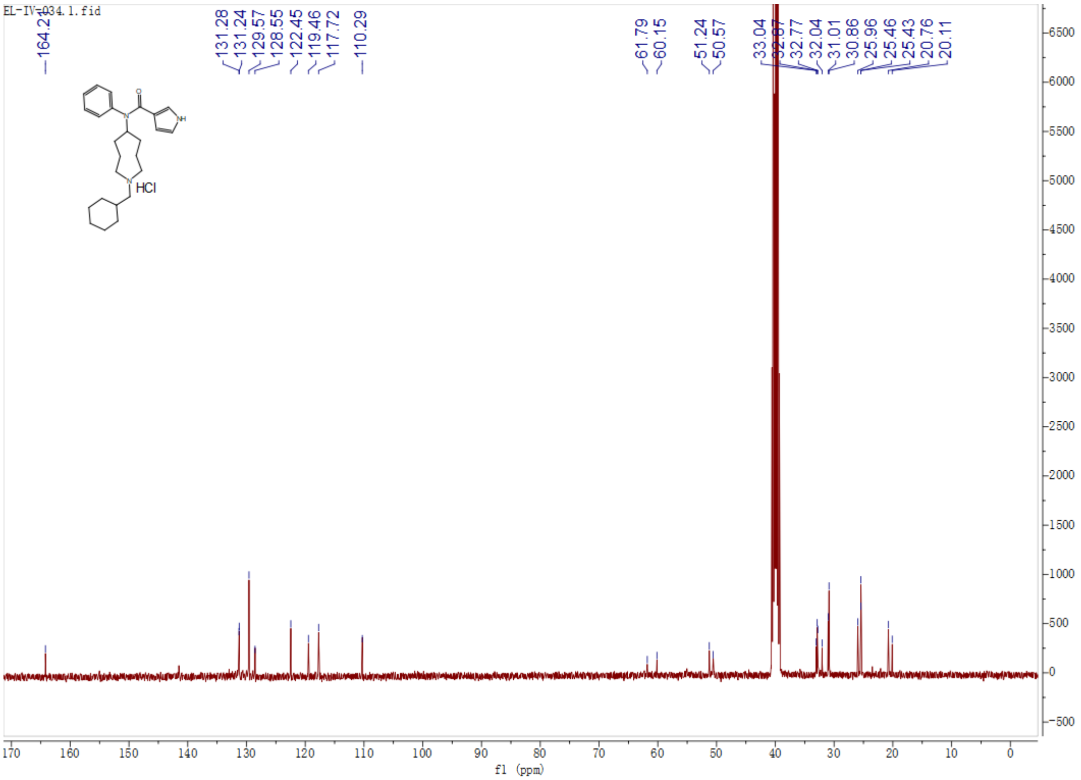

N-(1-benzylazocan-5-yl)-N-phenyl-1H-pyrrole-3-carboxamide hydrogen chloride **(86)**

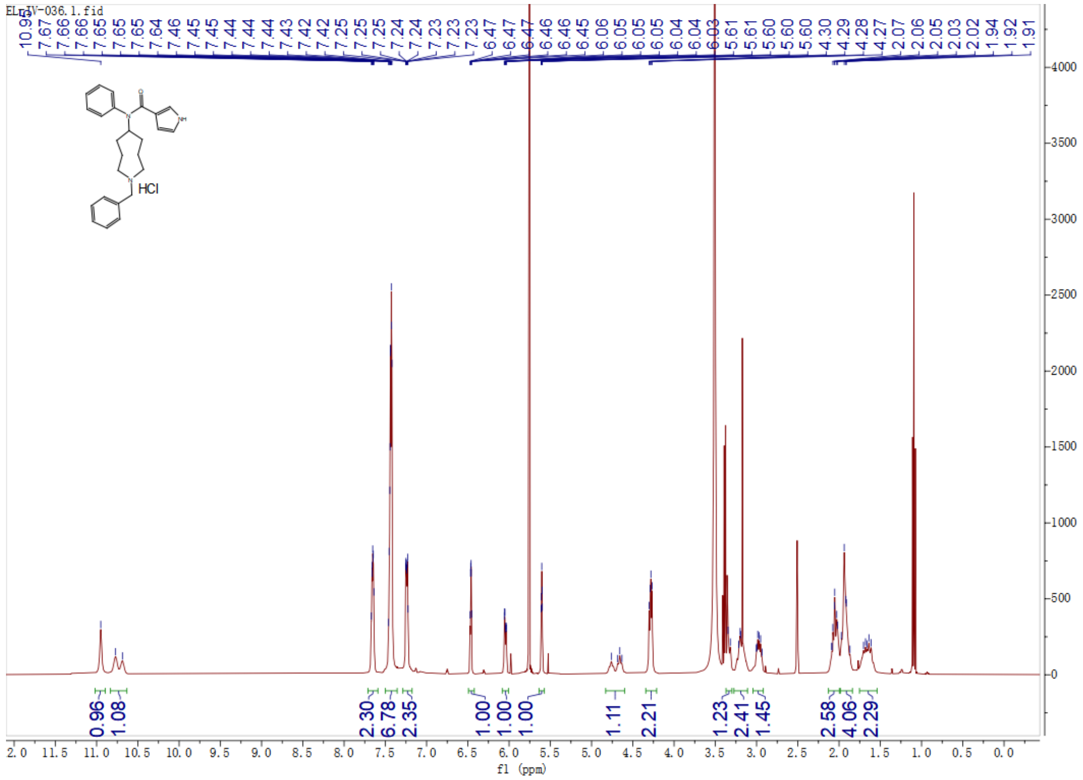

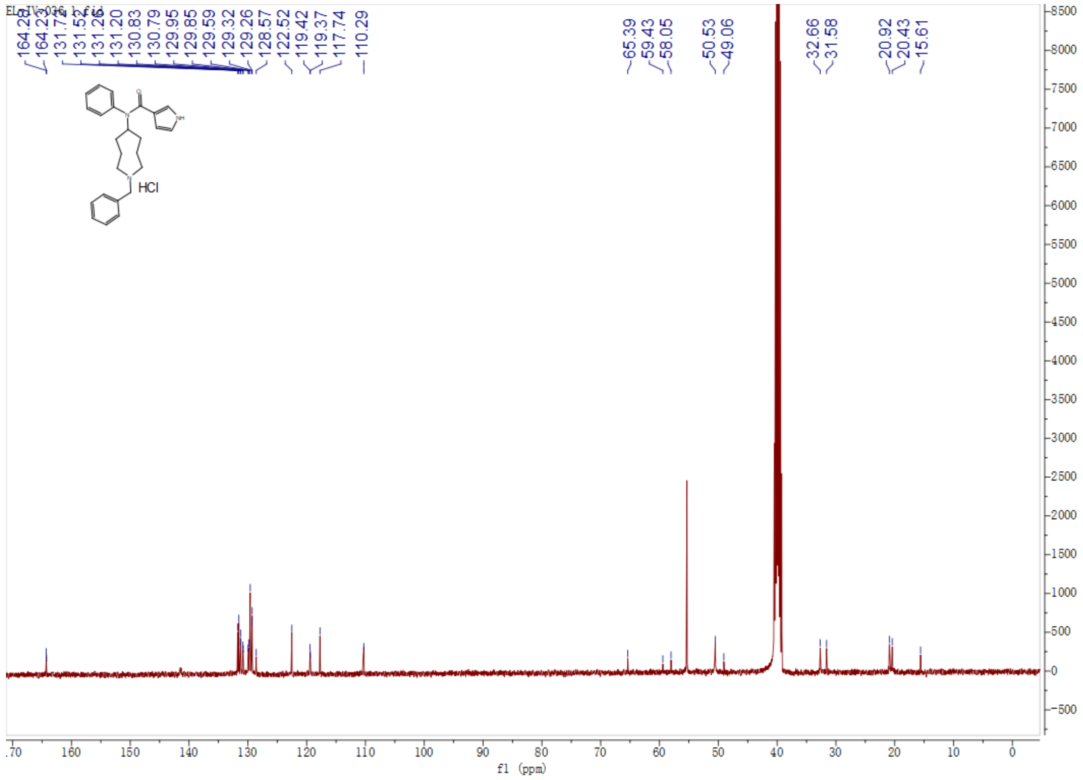

N-(1-phenethylazocan-5-yl)-N-phenyl-1H-pyrrole-3-carboxamide hydrogen chloride **(87)**

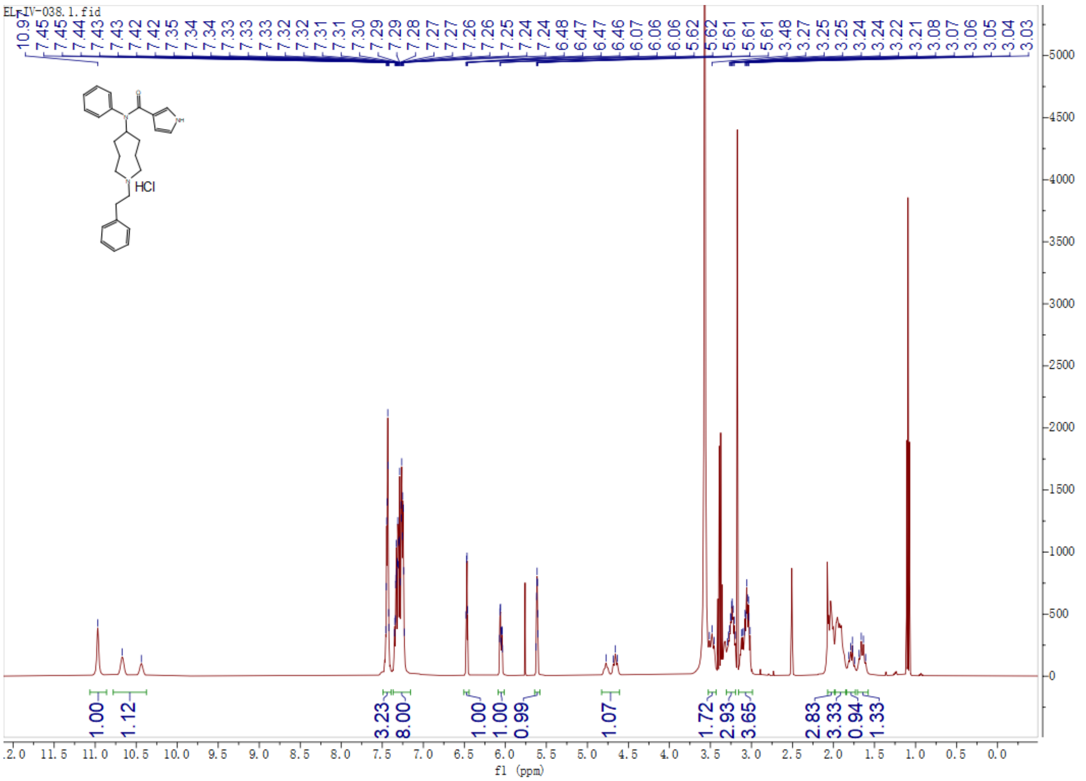

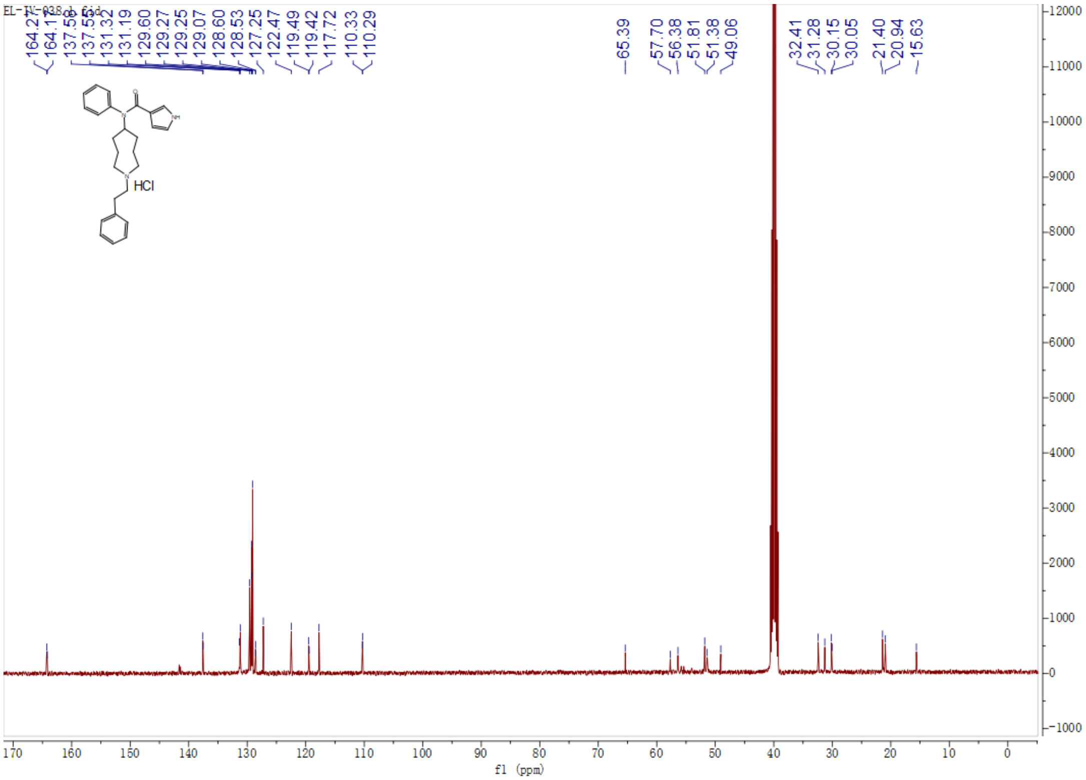

4. **Table S1.** Purity data of final compounds

HPLC System: Waters Arc HPLC
Column: XBridge™ C_{18} 3.5 µm (4.6 x 50 mm)
Sample Concentration: 0.25 mg/mL
Injection Solvent: Acetonitrile
Injection Volume: 5 µL
Isocratic Mobile Phase:
30% Mobile Phase A – 0.1% Trifluoroacetic acid in water
70% Mobile Phase B – Acetonitrile
Flow Rate: 0.2 mL/min
Single Wavelength: 210 nm
Run time: 10 min

**Compound No.**	**Retention Time (min)**	**Purity (%)**
4	2.658	100.00
5	2.672	99.70
6	2.740	99.66
7	2.803	98.92
8	2.918	99.26
9	2.772	99.58
10	2.878	97.77
11	2.732	96.91
12	2.663	99.16
13	2.730	99.20
14	2.790	98.87
15	2.918	99.36
16 [a]	2.877	96.28
17	2.875	97.75
18	2.657	98.94
19	2.662	98.33
20	2.772	99.70
21	2.790	97.82
22	2.925	99.13
23	2.765	99.79
24	2.873	97.95
25	2.670	100.00
26	2.718	99.08
27	2.775	99.24
28	2.840	100.00
29	2.938	99.46
30	2.842	99.14
31	2.878	99.72
32 [a,b]	2.545	100.00
33 [a,b]	2.580	99.72
34 [a,b]	2.633	97.16

35 [a,b]	2.672	97.52
36 [a,b]	2.735	97.49
37 [a]	2.637	97.24
38 [a]	2.677	100.00
39	2.708	98.14
40	2.750	99.37
41	2.823	98.78
42	2.913	100.00
43	3.00	100.00
44 [a]	2.842	99.79
45	2.923	100.00
46	2.653	98.50
47	2.687	98.63
48	2.755	99.77
49	2.825	100.00
50	2.915	99.37
51	2.772	99.74
52	2.853	98.27
53	2.665	97.77
54	2.697	97.85
55	2.770	100.00
56	2.838	100.00
57	2.928	99.27
58	2.780	99.46
59	2.852	98.34
60	2.878	99.90
61	2.803	99.53
62	2.908	98.96
63	3.067	99.56
64	3.118	97.30
65	2.875	99.88
66	3.018	97.10
67 [a]	2.562	99.55
68 [a]	2.860	98.90
69 [a]	2.630	97.59
70 [a]	2.888	100.00
71 [a]	2.565	99.15
72 [a]	3.077	100.00
73 [a]	2.863	99.30
74	2.733	96.59
75	2.775	98.51
76	2.863	96.12
77	2.945	98.19
78	3.063	98.95
79	2.883	96.45
80	2.972	98.34
81 [a]	2.565	95.77

82 [a]	2.590	98.60
83 [a]	2.645	100.00
84 [a]	2.700	97.90
85 [a]	2.773	97.52
86 [a]	2.677	97.56
87 [a]	2.722	96.66
[a] 1 drop of H_2O added to injection solvent to ensure homogenous solution.		
[b] tested as the free base. Compounds **32-36** exhibited instability in their HCl salt forms under HPLC conditions. Therefore, the free base forms of these compounds were used for analytic purposed.		

6. HPLC chromatograms of final compounds.

N-(1-allylazepan-4-yl)-N-phenylfuran-2-carboxamide hydrogen chloride **(4)**

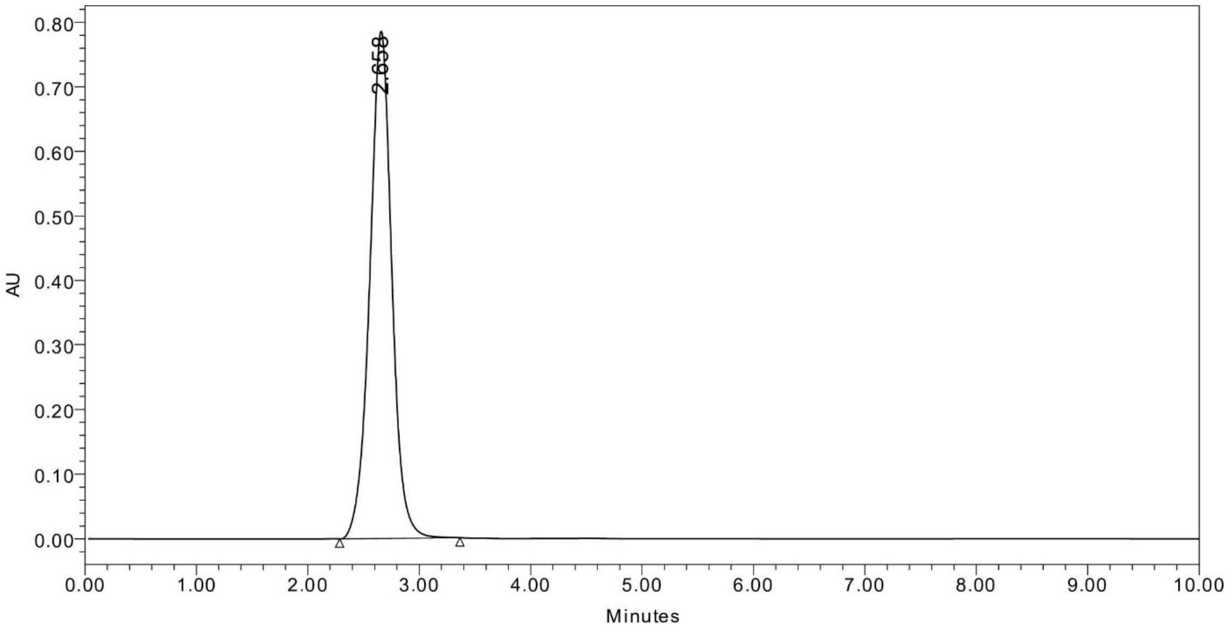

N-(1-(cyclopropylmethyl)azepan-4-yl)-N-phenylfuran-2-carboxamide hydrogen chloride (**5**)

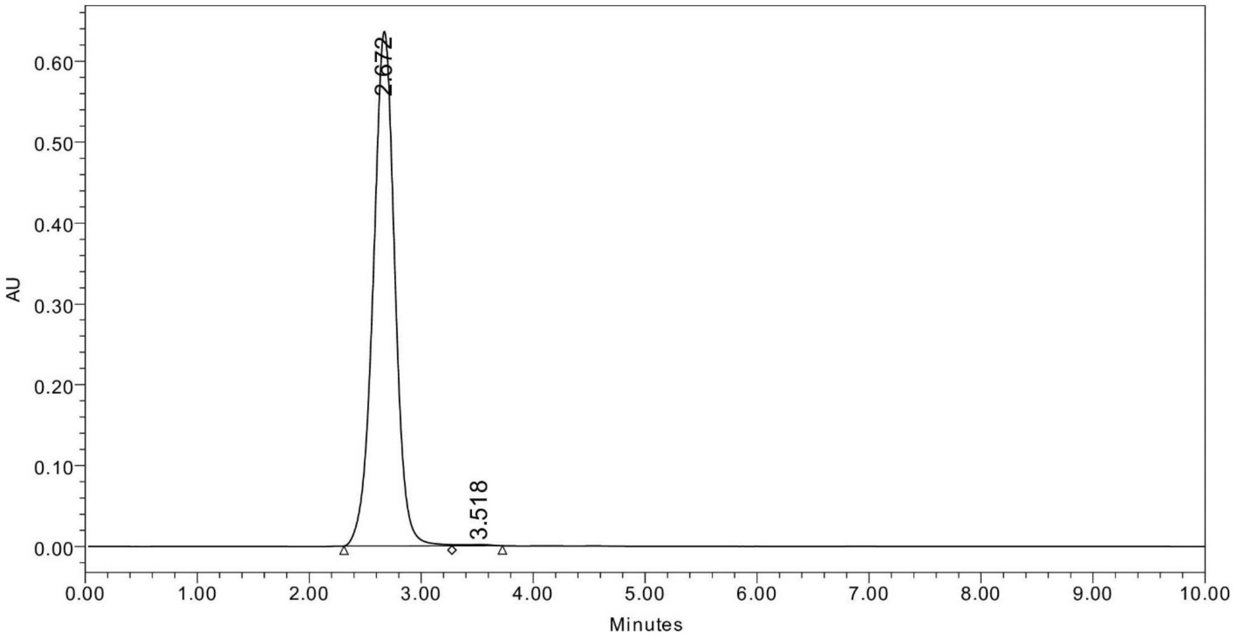

N-(1-(cyclobutylmethyl)azepan-4-yl)-N-phenylfuran-2-carboxamide hydrogen chloride **(6)**

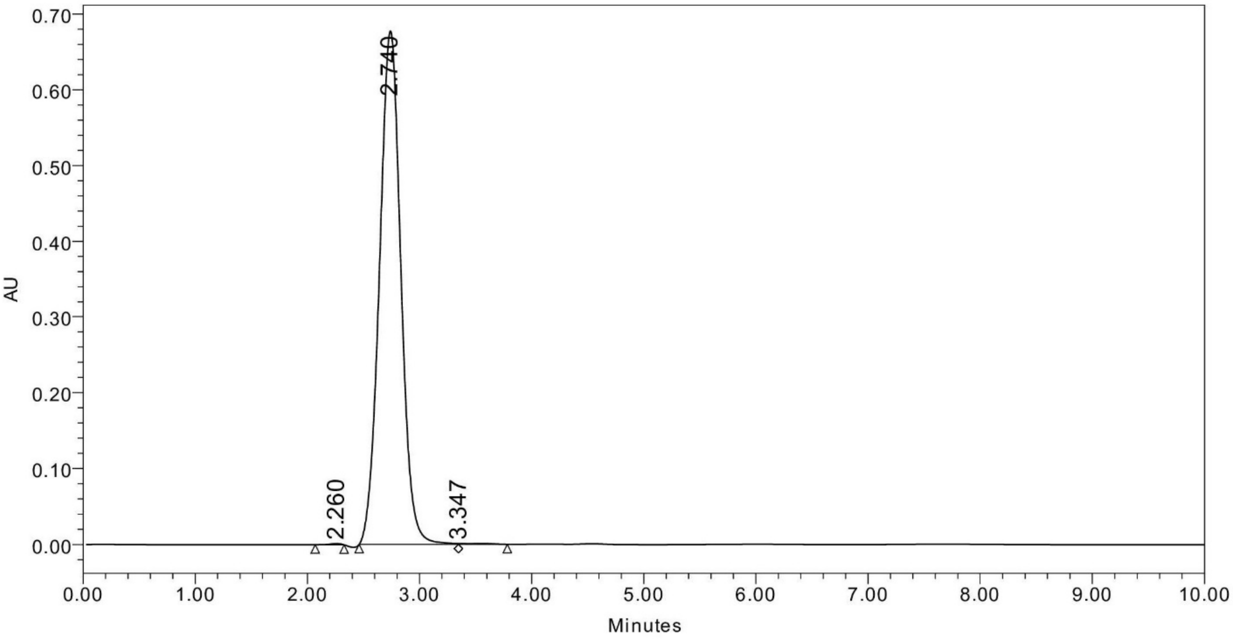

N-(1-(cyclopentylmethyl)azepan-4-yl)-N-phenylfuran-2-carboxamide hydrogen chloride **(7)**

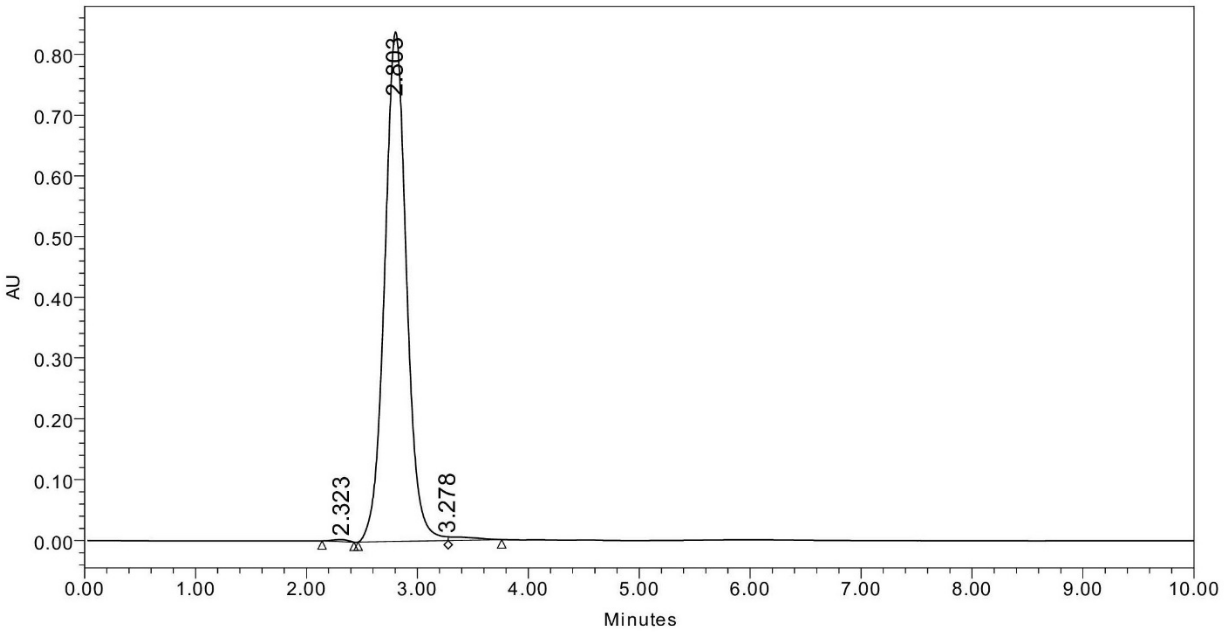

N-(1-(cyclohexylmethyl)azepan-4-yl)-N-phenylfuran-2-carboxamide hydrogen chloride **(8)**

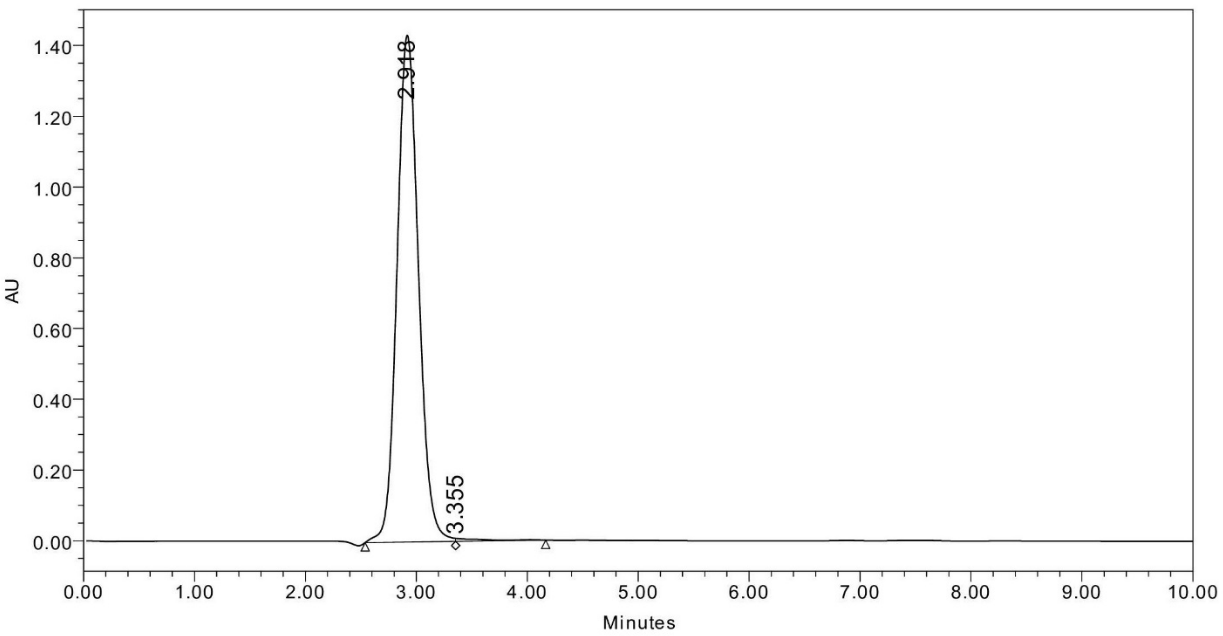

N-(1-benzylazepan-4-yl)-N-phenylfuran-2-carboxamide hydrogen chloride **(9)**

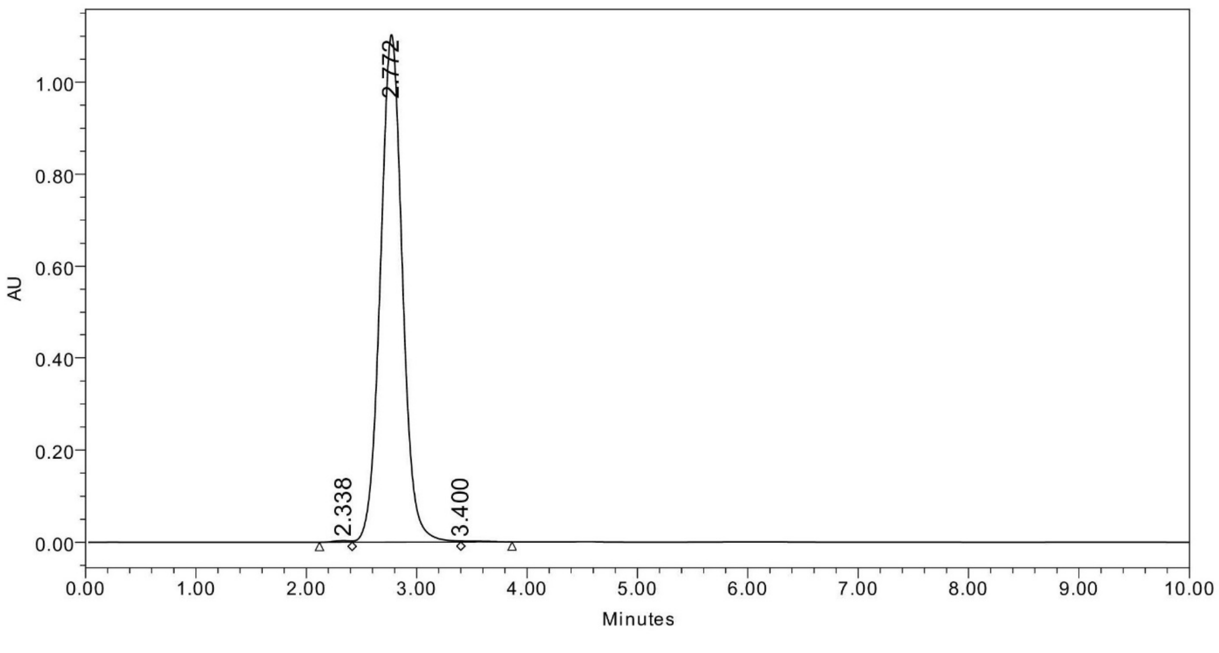

N-(1-phenethylazepan-4-yl)-N-phenylfuran-2-carboxamide hydrogen chloride (**10**)

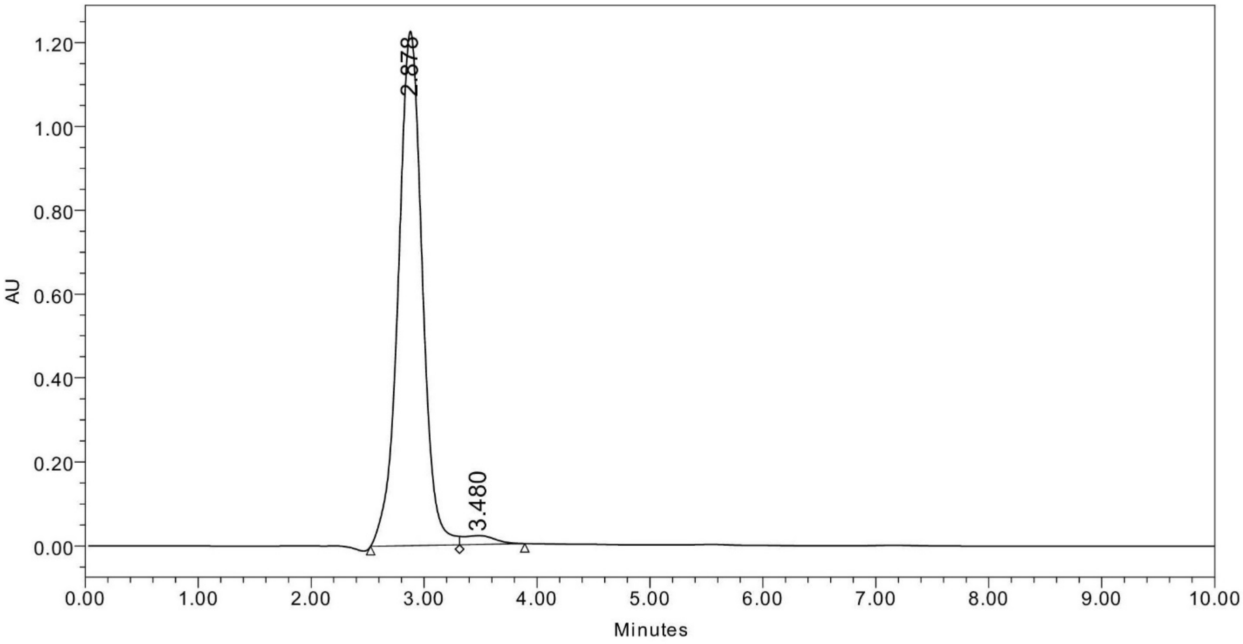

N-(1-allylazepan-4-yl)-N-phenylthiophene-2-carboxamide hydrogen chloride **(11)**

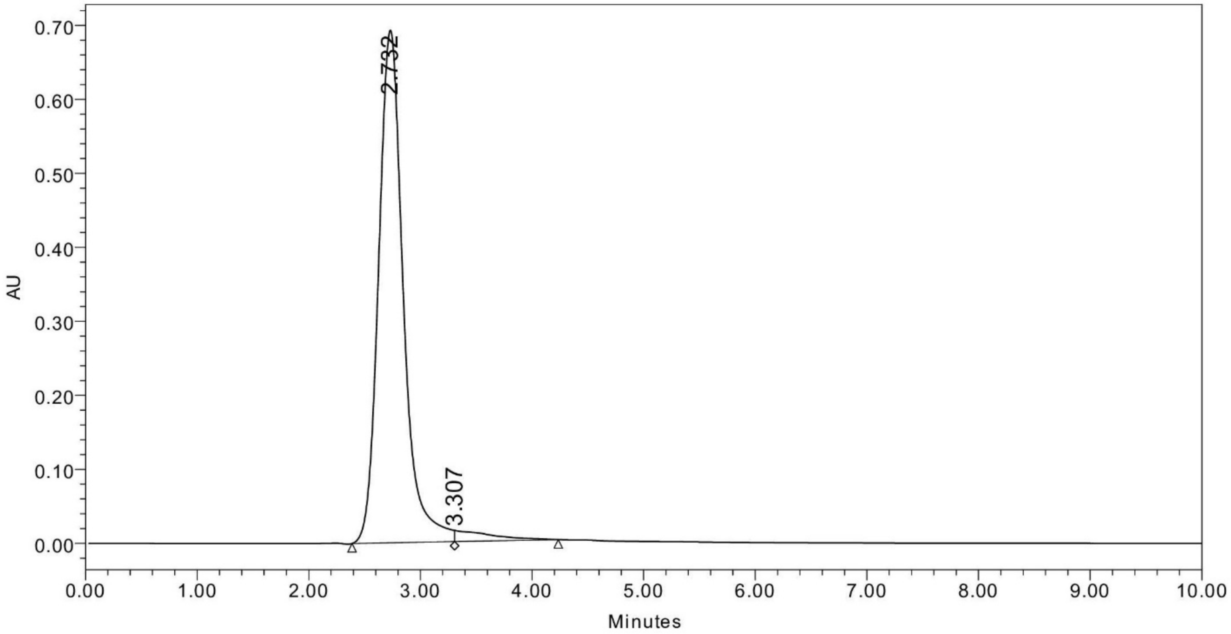

N-(1-(cyclopropylmethyl)azepan-4-yl)-N-phenylthiophene-2-carboxamide hydrogen chloride **(12)**

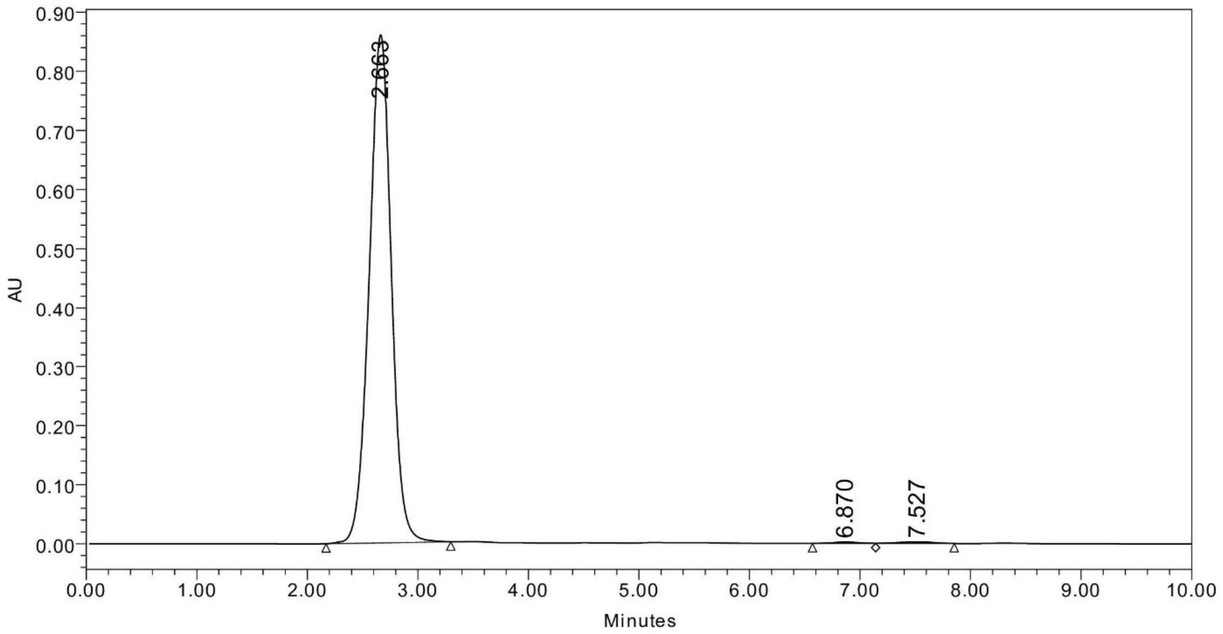

N-(1-(cyclobutylmethyl)azepan-4-yl)-N-phenylthiophene-2-carboxamide hydrogen chloride **(13)**

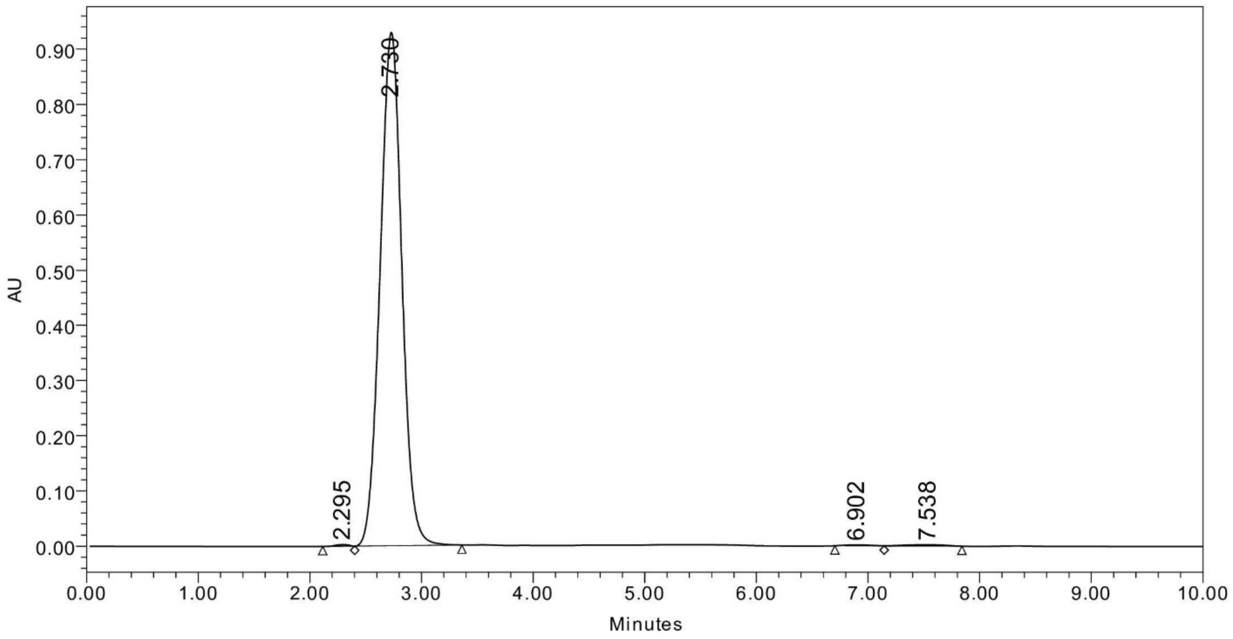

N-(1-(cyclopentylmethyl)azepan-4-yl)-N-phenylthiophene-2-carboxamide hydrogen chloride **(14)**

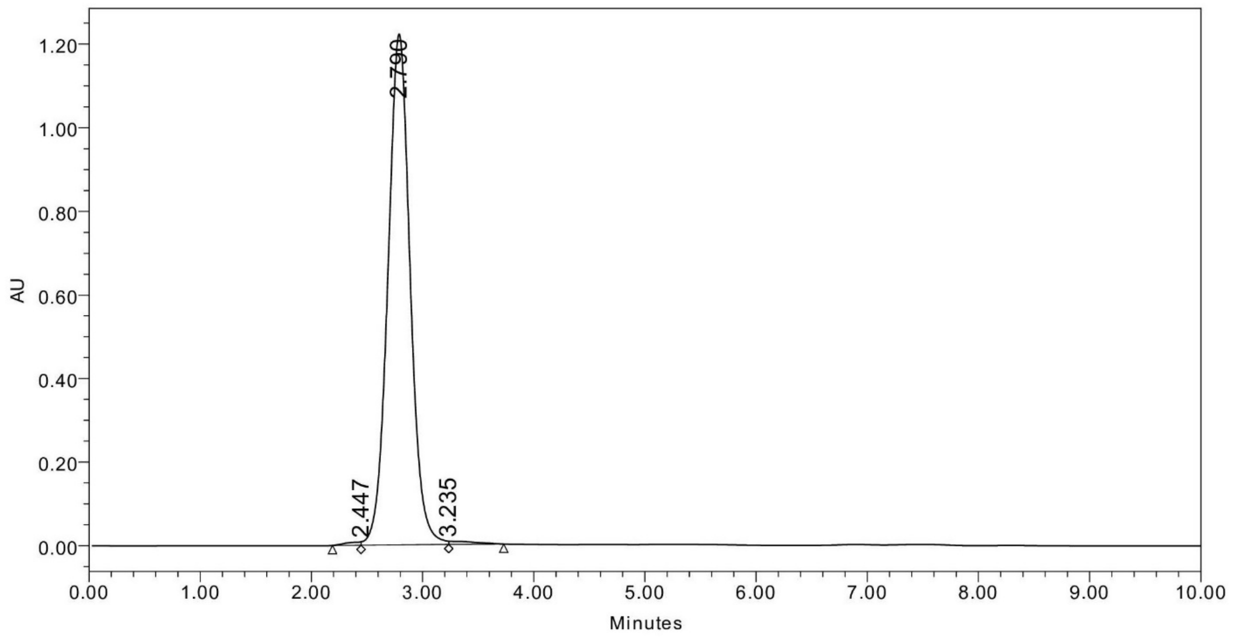

N-(1-(cyclohexylmethyl)azepan-4-yl)-N-phenylthiophene-2-carboxamide hydrogen chloride **(15)**

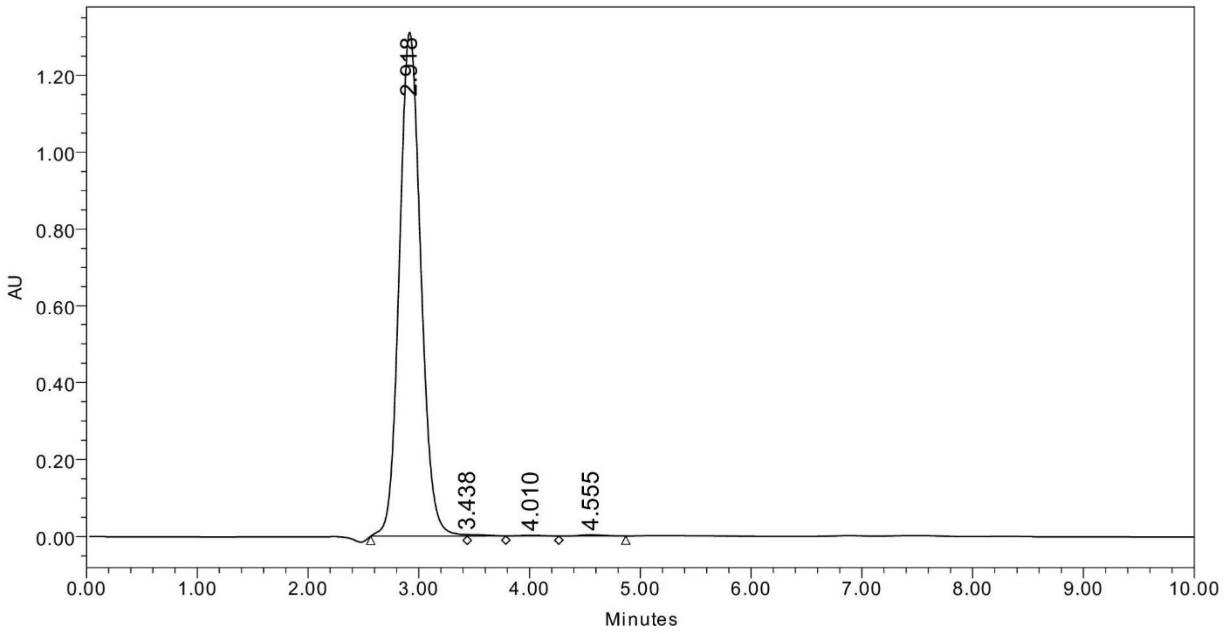

N-(1-benzylazepan-4-yl)-N-phenylthiophene-2-carboxamide hydrogen chloride **(16)**

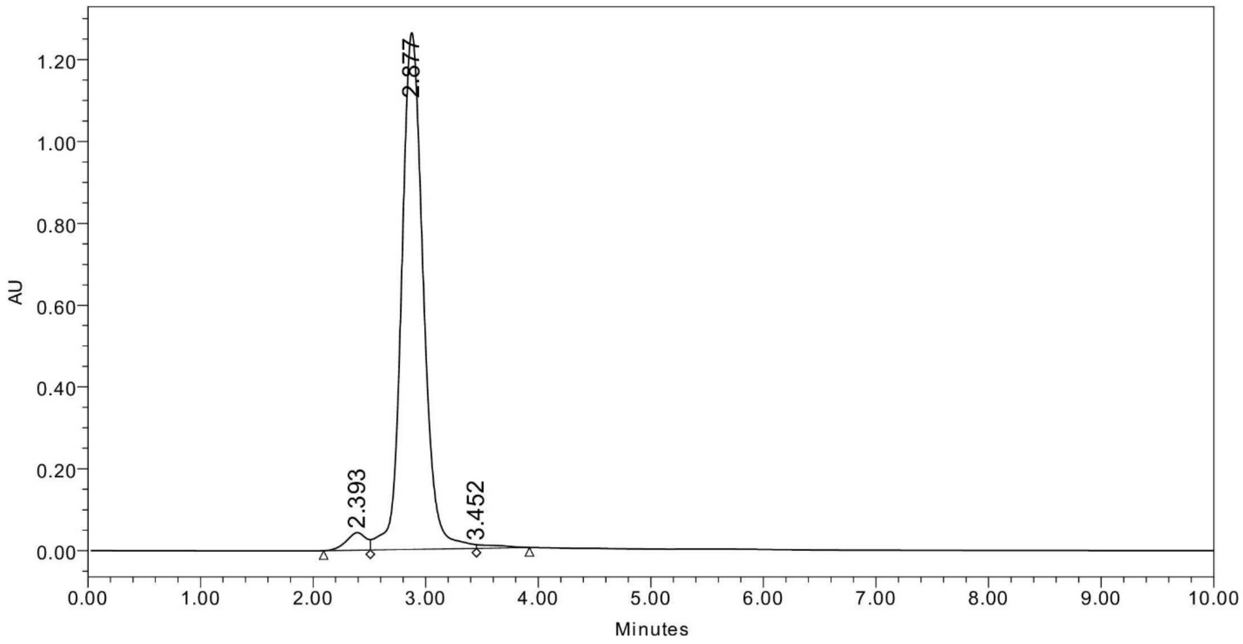

N-(1-phenethylazepan-4-yl)-N-phenylthiophene-2-carboxamide hydrogen chloride **(17)**

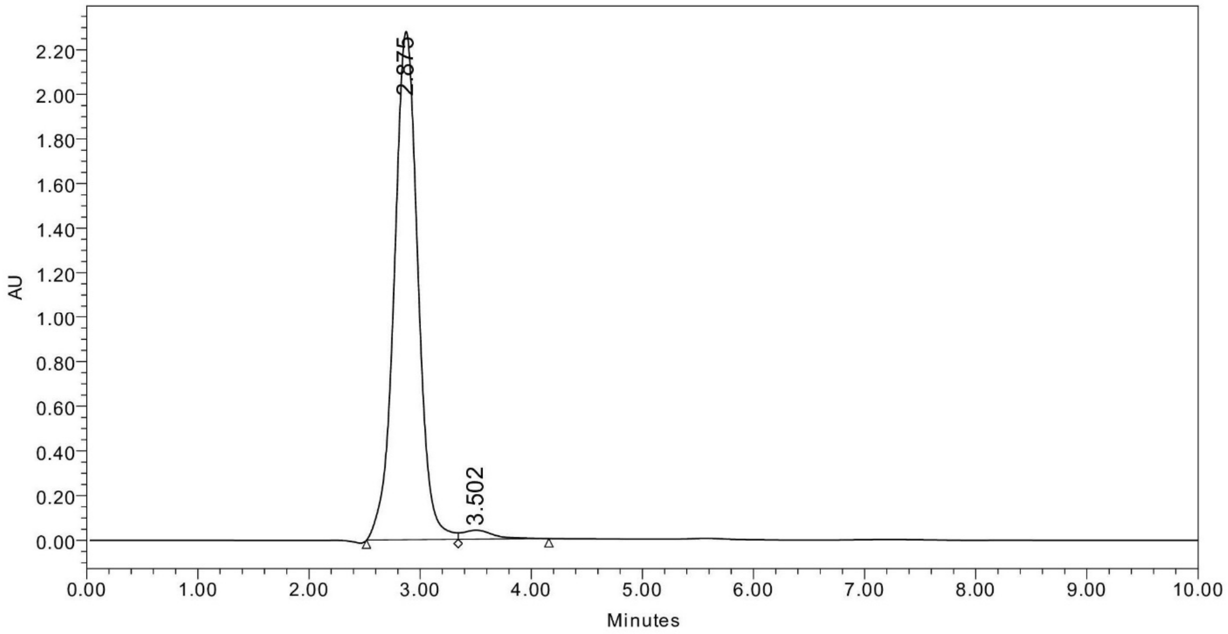

N-(1-allylazepan-4-yl)-N-phenylfuran-3-carboxamide hydrogen chloride **(18)**

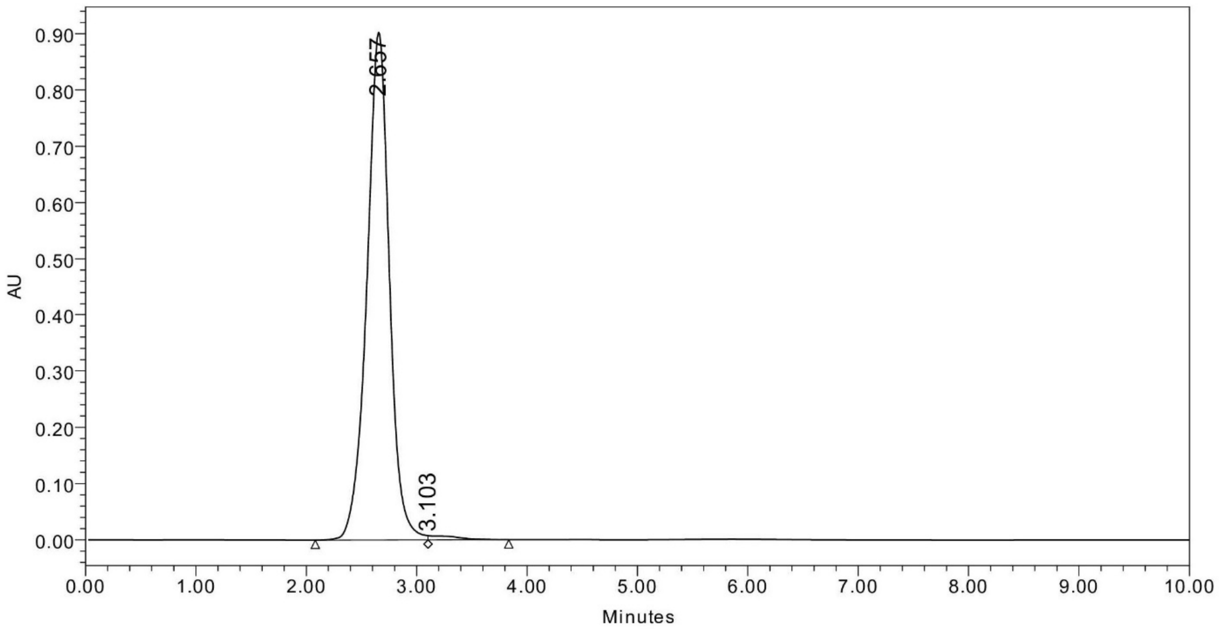

N-(1-(cyclopropylmethyl)azepan-4-yl)-N-phenylfuran-3-carboxamide hydrogen chloride **(19)**

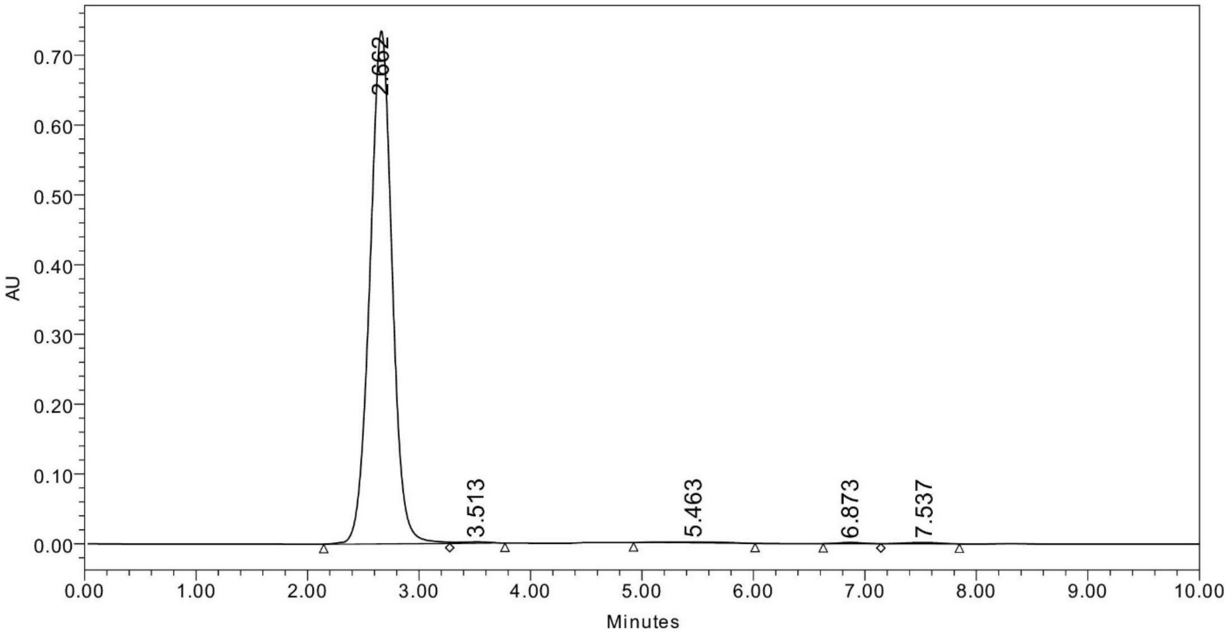

N-(1-(cyclobutylmethyl)azepan-4-yl)-N-phenylfuran-3-carboxamide hydrogen chloride **(20)**

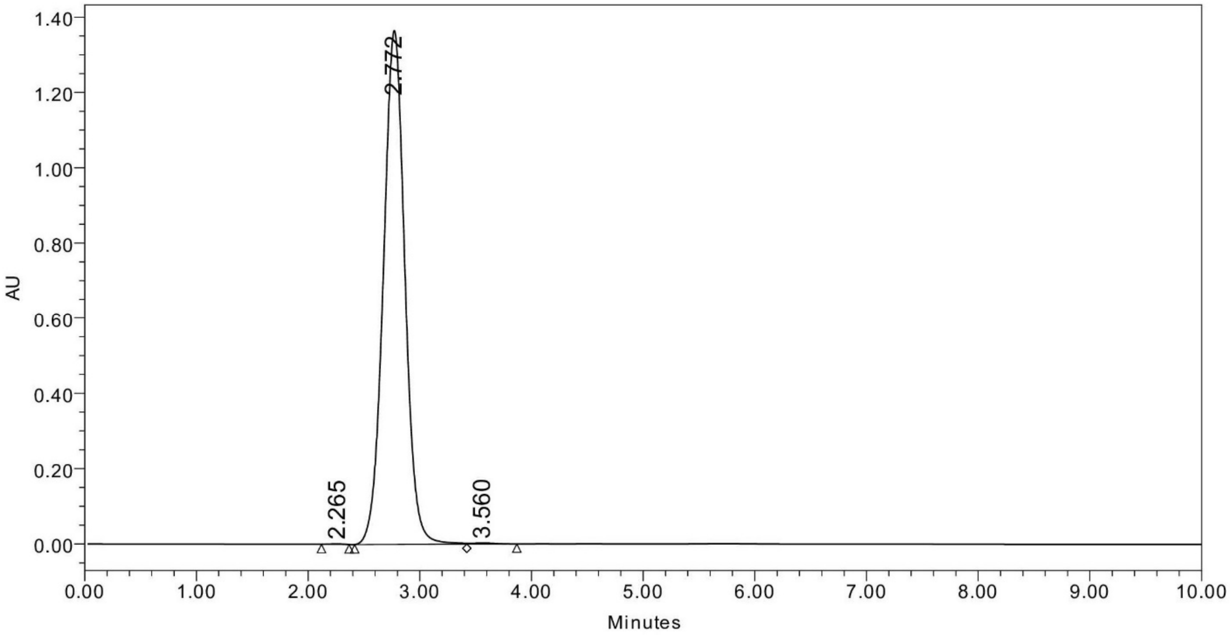

N-(1-(cyclopentylmethyl)azepan-4-yl)-N-phenylfuran-3-carboxamide hydrogen chloride **(21)**

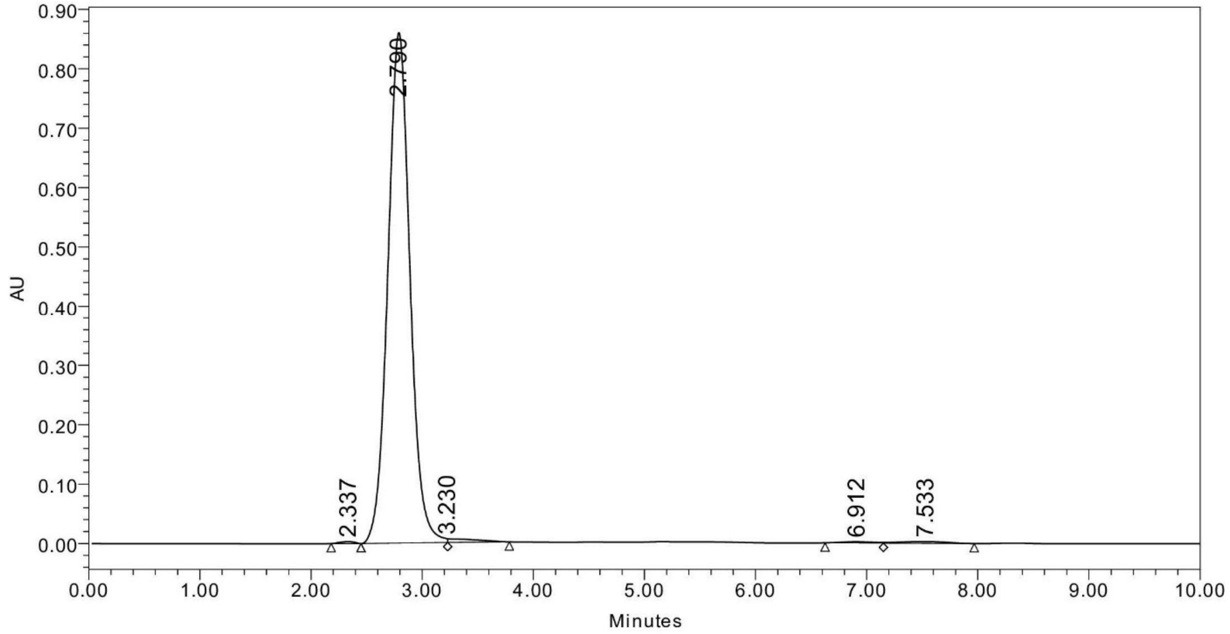

N-(1-(cyclohexylmethyl)azepan-4-yl)-N-phenylfuran-3-carboxamide hydrogen chloride **(22)**

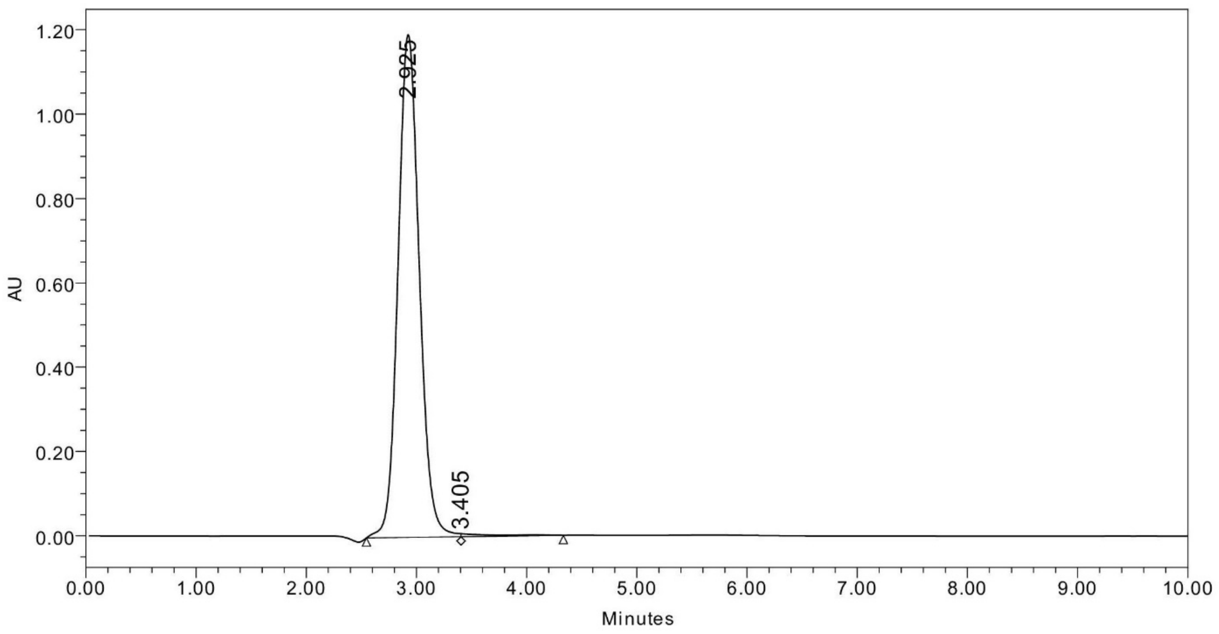

N-(1-benzylazepan-4-yl)-N-phenylfuran-3-carboxamide hydrogen chloride **(23)**

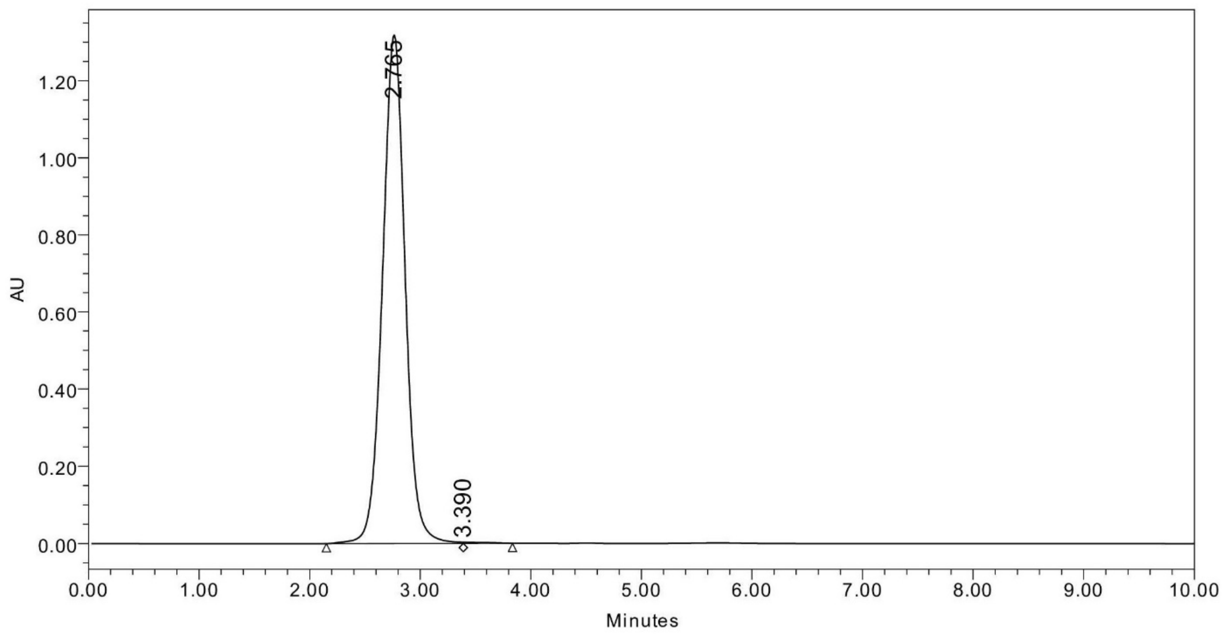

N-(1-phenethylazepan-4-yl)-N-phenylfuran-3-carboxamide hydrogen chloride **(24)**

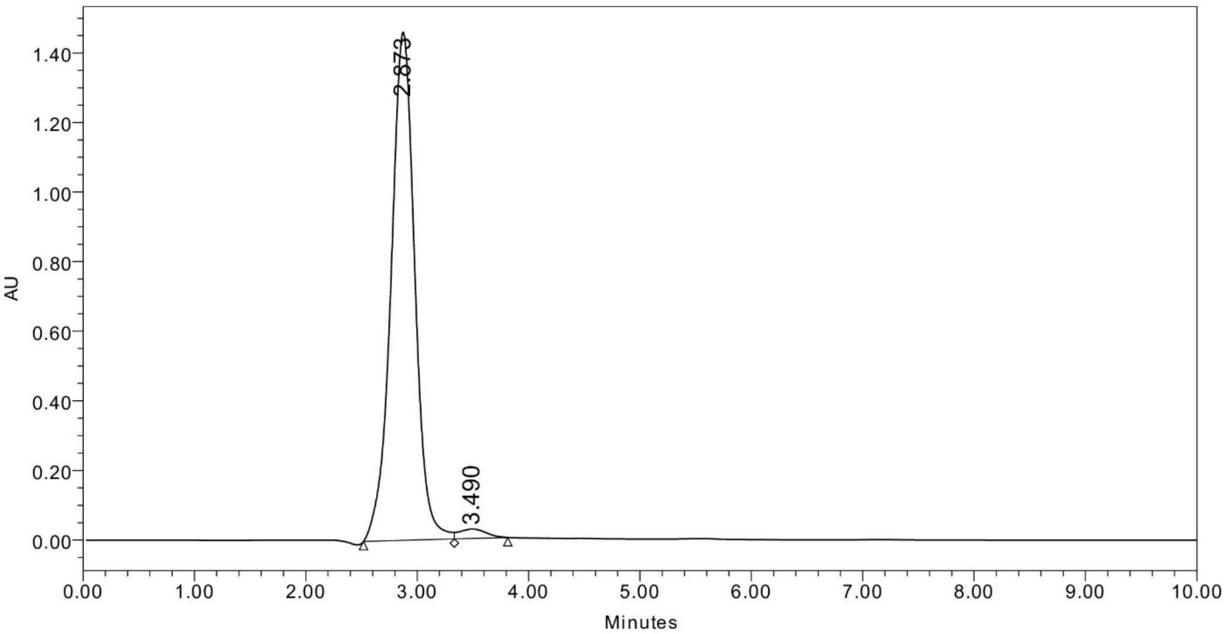

N-(1-allylazepan-4-yl)-N-phenyl-1H-pyrrole-2-carboxamide hydrochloride **(25)**

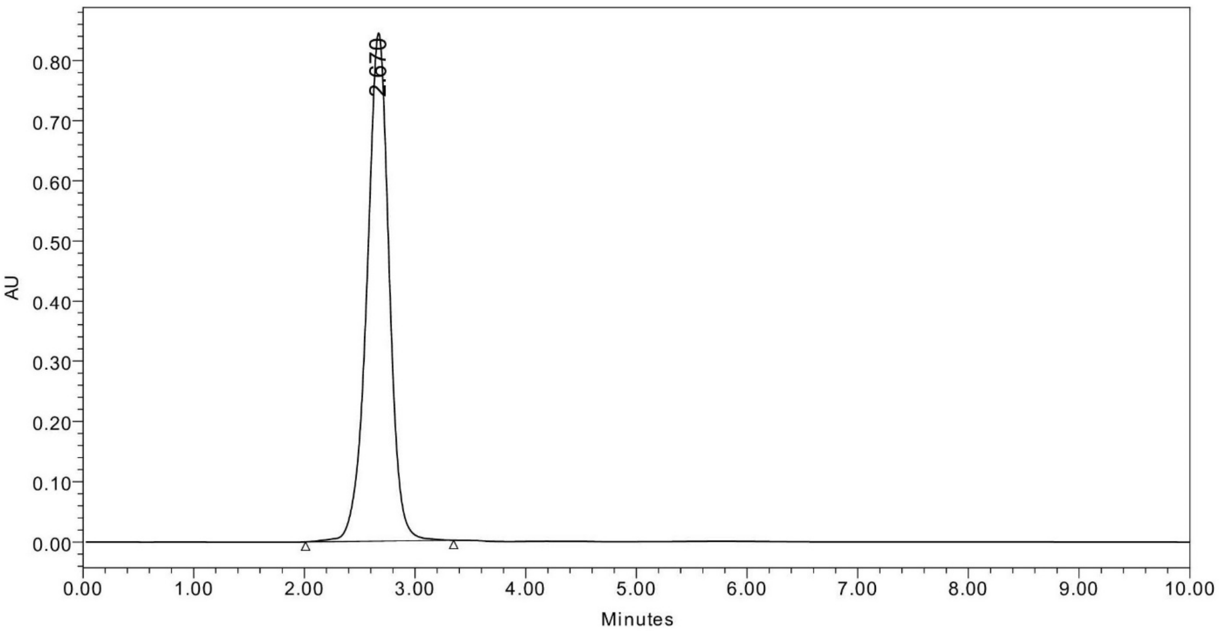

N-(1-(cyclopropylmethyl)azepan-4-yl)-N-phenyl-1H-pyrrole-2-carboxamide hydrochloride **(26)**

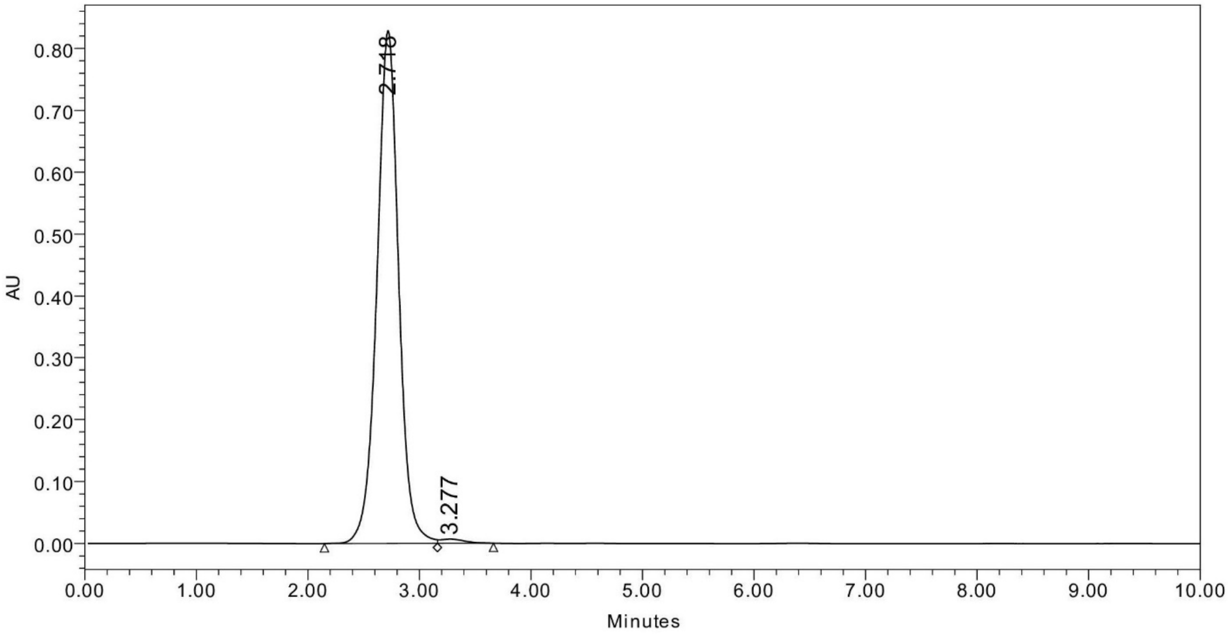

N-(1-(cyclobutylmethyl)azepan-4-yl)-N-phenyl-1H-pyrrole-2-carboxamide hydrochloride **(27)**

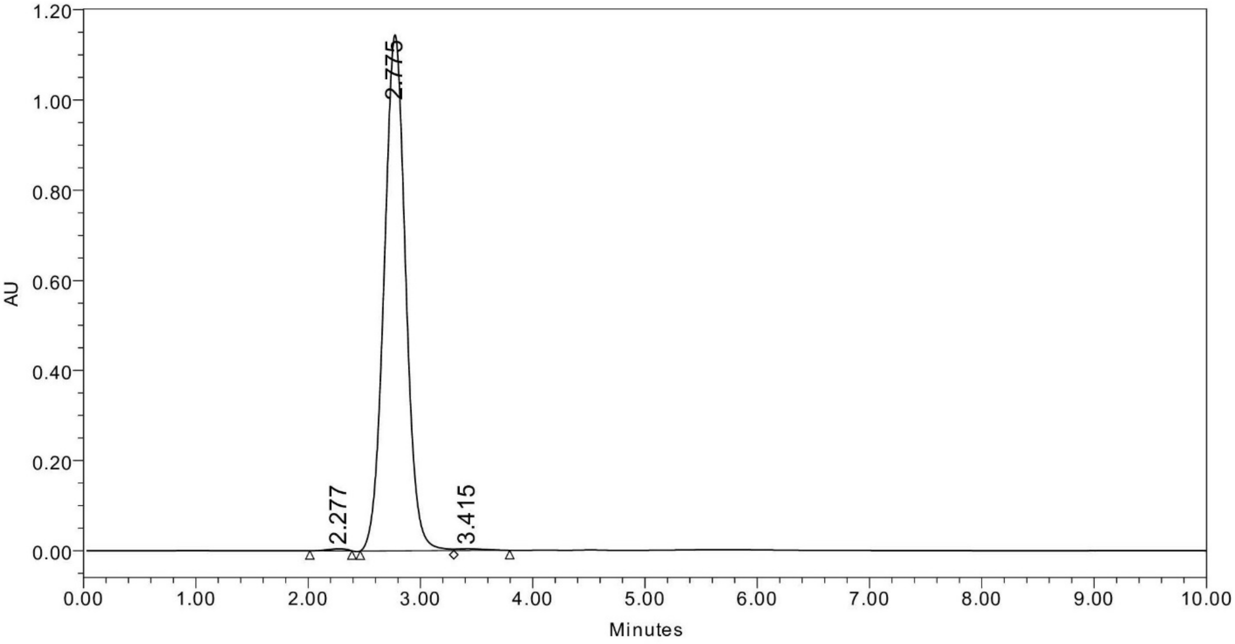

N-(1-(cyclopentylmethyl)azepan-4-yl)-N-phenyl-1H-pyrrole-2-carboxamide hydrochloride **(28)**

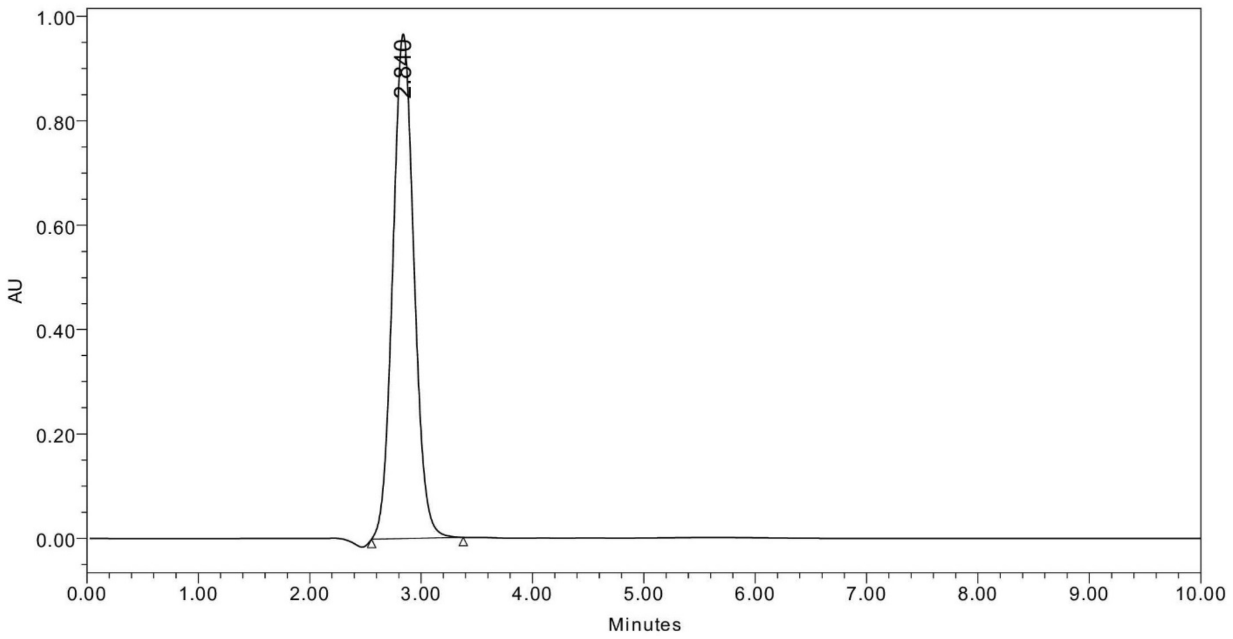

N-(1-(cyclohexylmethyl)azepan-4-yl)-N-phenyl-1H-pyrrole-2-carboxamide hydrochloride **(29)**

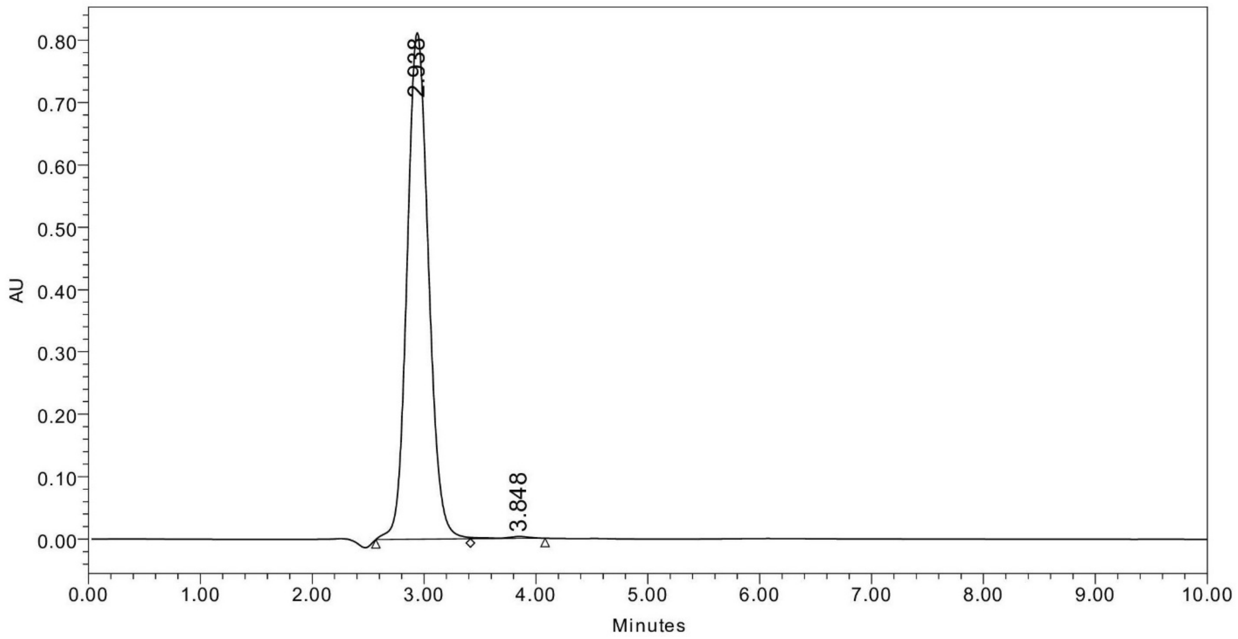

N-(1-benzylazepan-4-yl)-N-phenyl-1H-pyrrole-2-carboxamide hydrochloride **(30)**

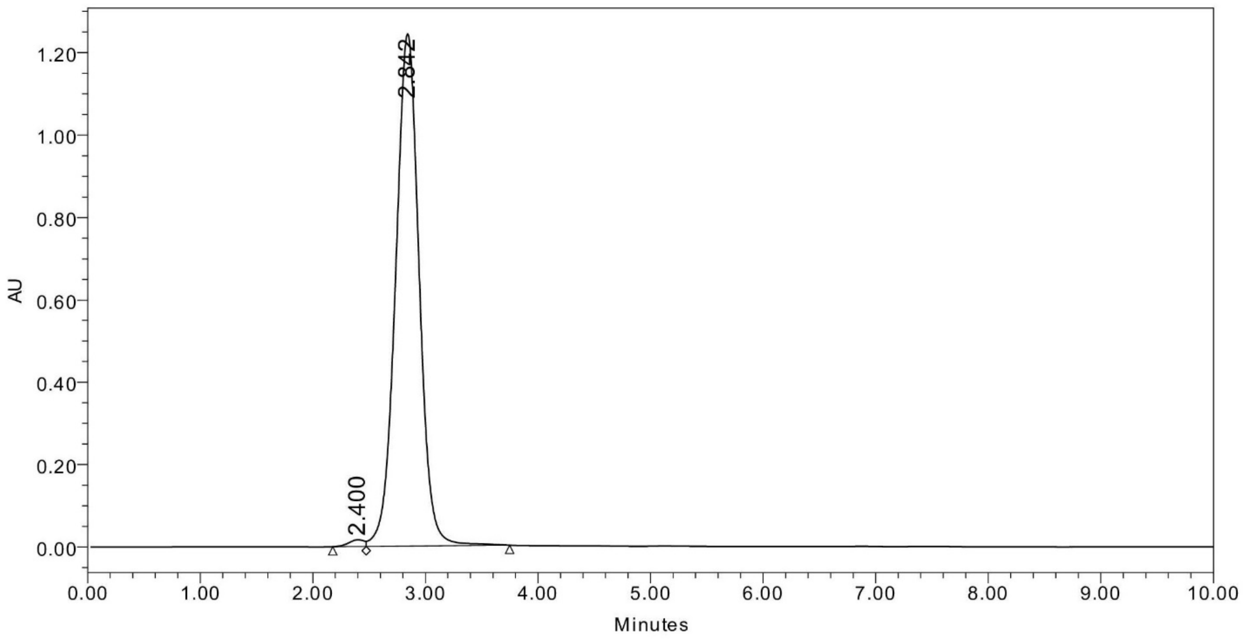

N-(1-benzylazepan-4-yl)-N-phenyl-1H-pyrrole-2-carboxamide hydrochloride **(31)**

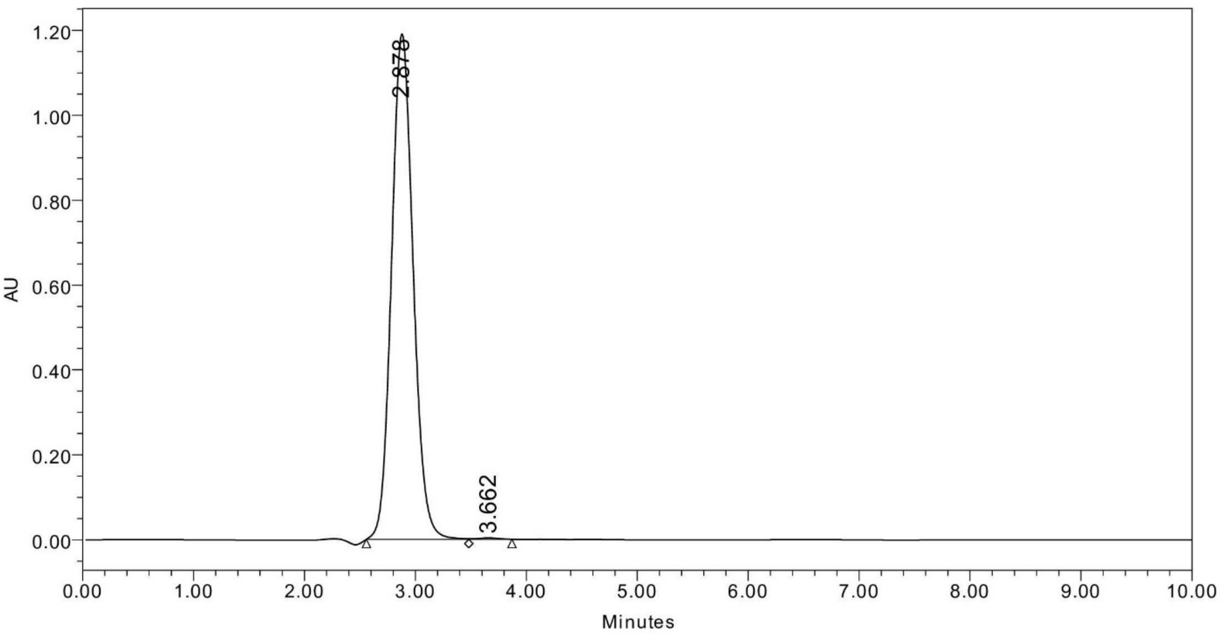

N-(1-allylazepan-4-yl)-N-phenyl-1H-pyrrole-3-carboxamide hydrochloride **(32)**

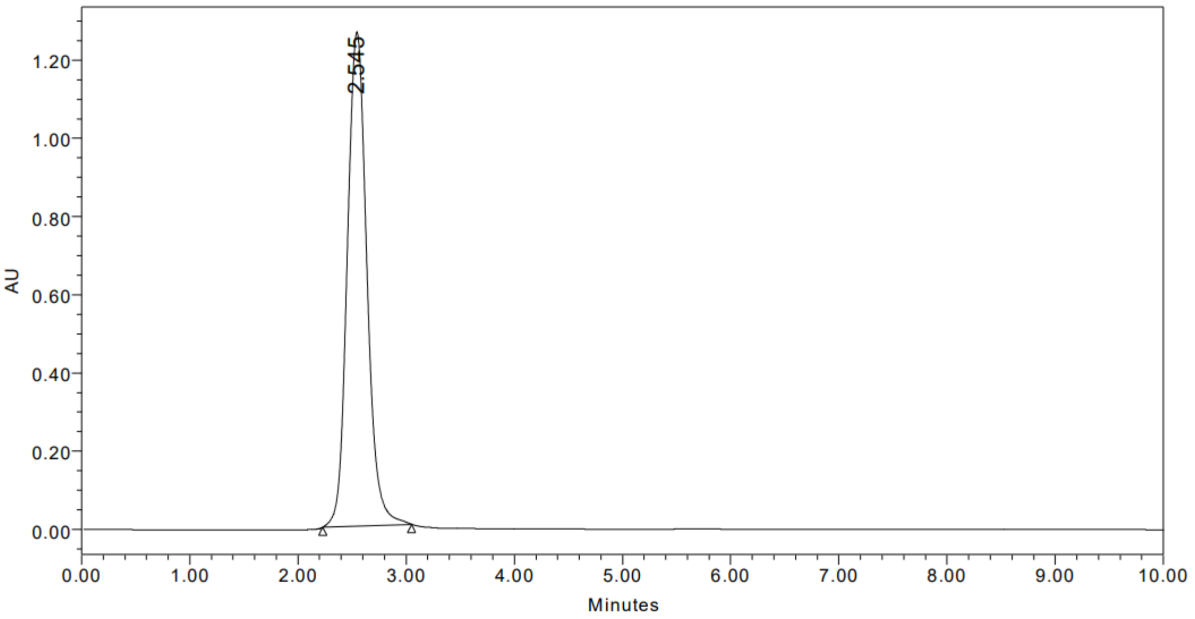

N-(1-(cyclopropylmethyl)azepan-4-yl)-N-phenyl-1H-pyrrole-3-carboxamide **(33)**

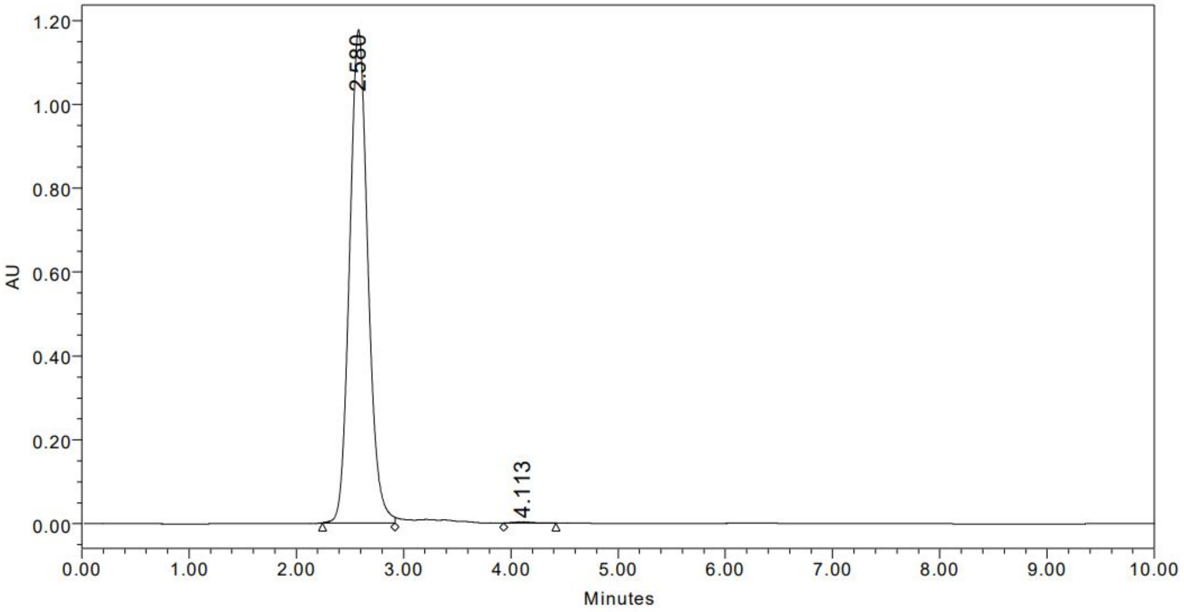

N-(1-(cyclobutylmethyl)azepan-4-yl)-N-phenyl-1H-pyrrole-3-carboxamide hydrochloride **(34)**

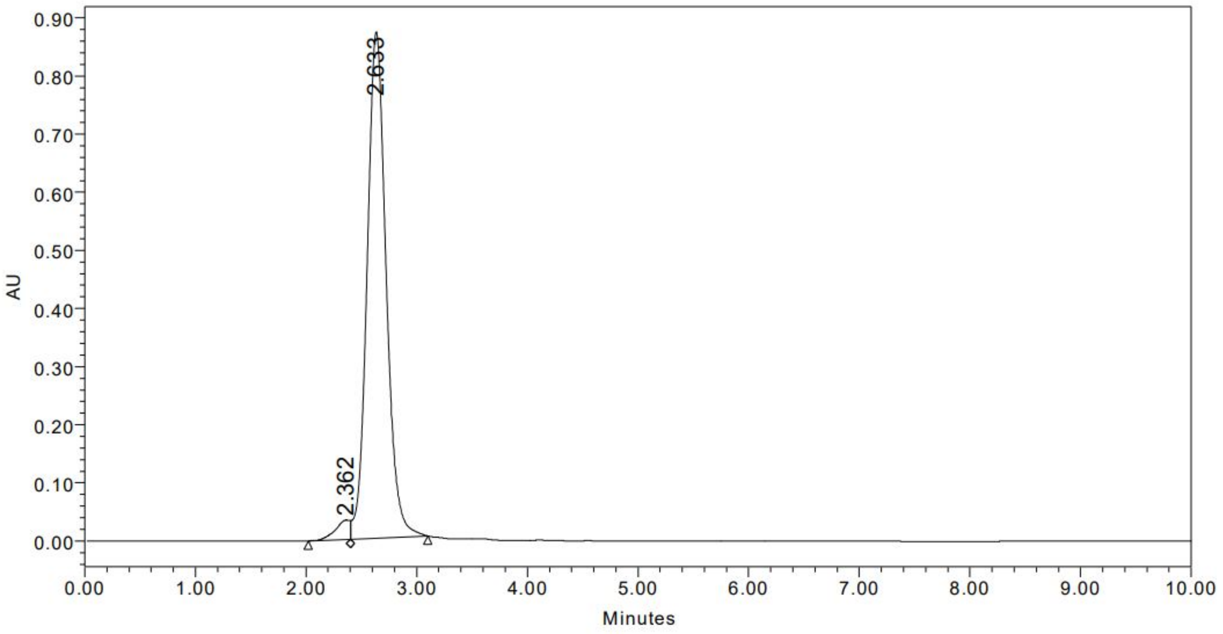

N-(1-(cyclopentylmethyl)azepan-4-yl)-N-phenyl-1H-pyrrole-3-carboxamide hydrochloride **(35)**

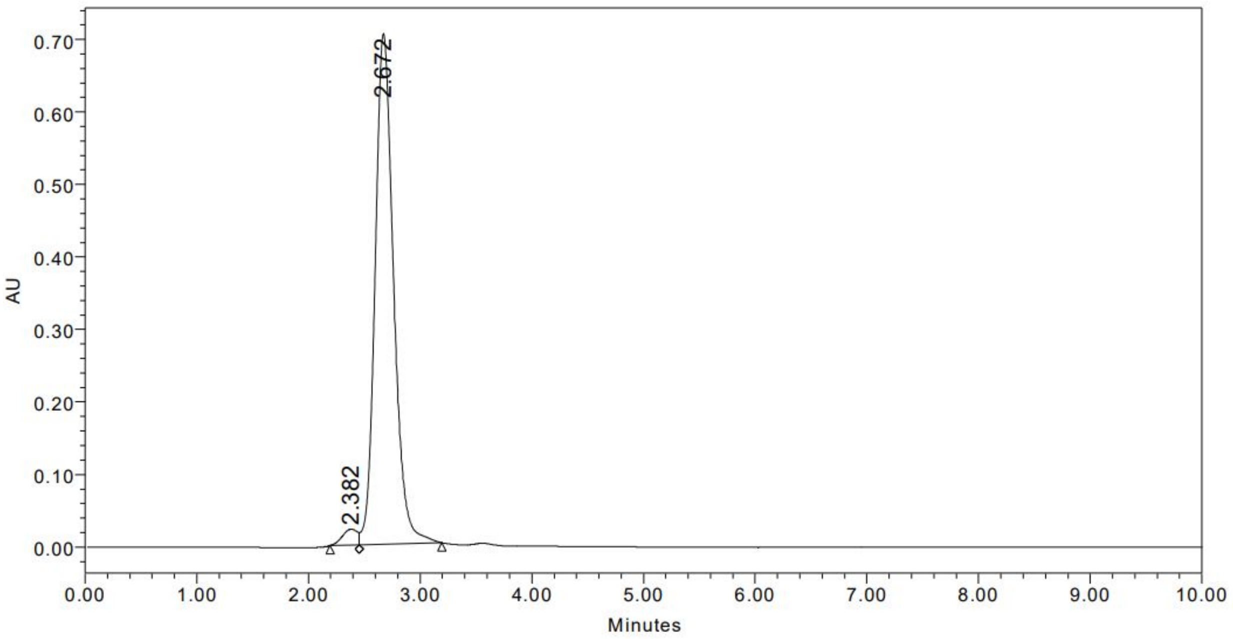

N-(1-(cyclohexylmethyl)azepan-4-yl)-N-phenyl-1H-pyrrole-3-carboxamide hydrochloride **(36)**

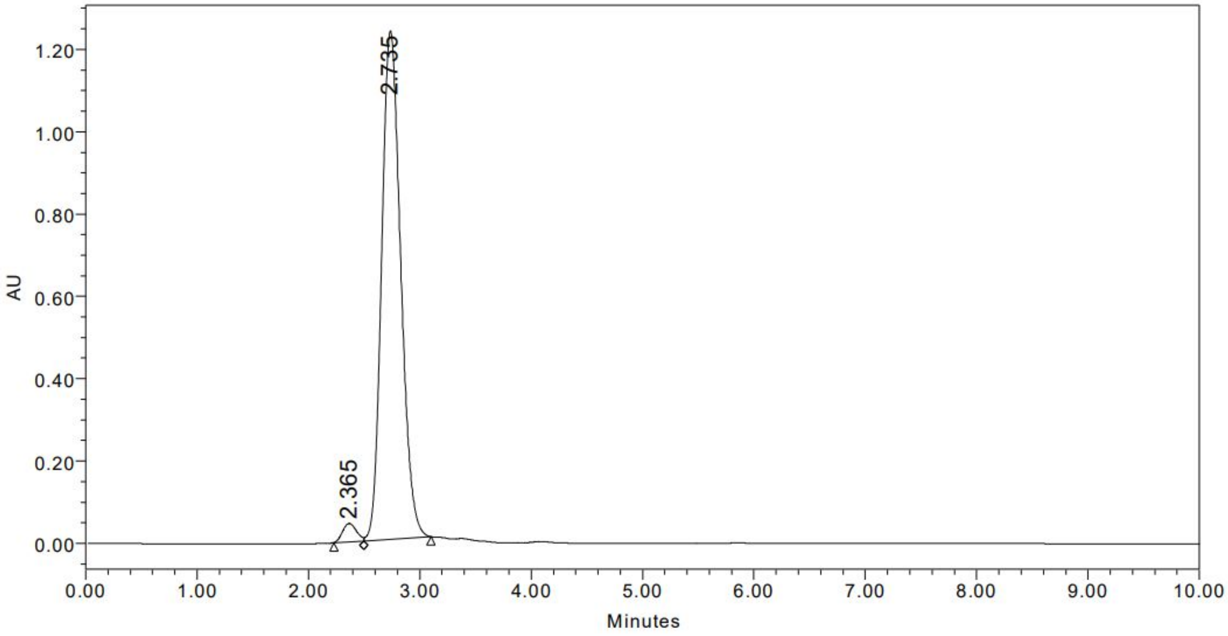

N-(1-benzylazepan-4-yl)-N-phenyl-1H-pyrrole-3-carboxamide hydrochloride **(37)**

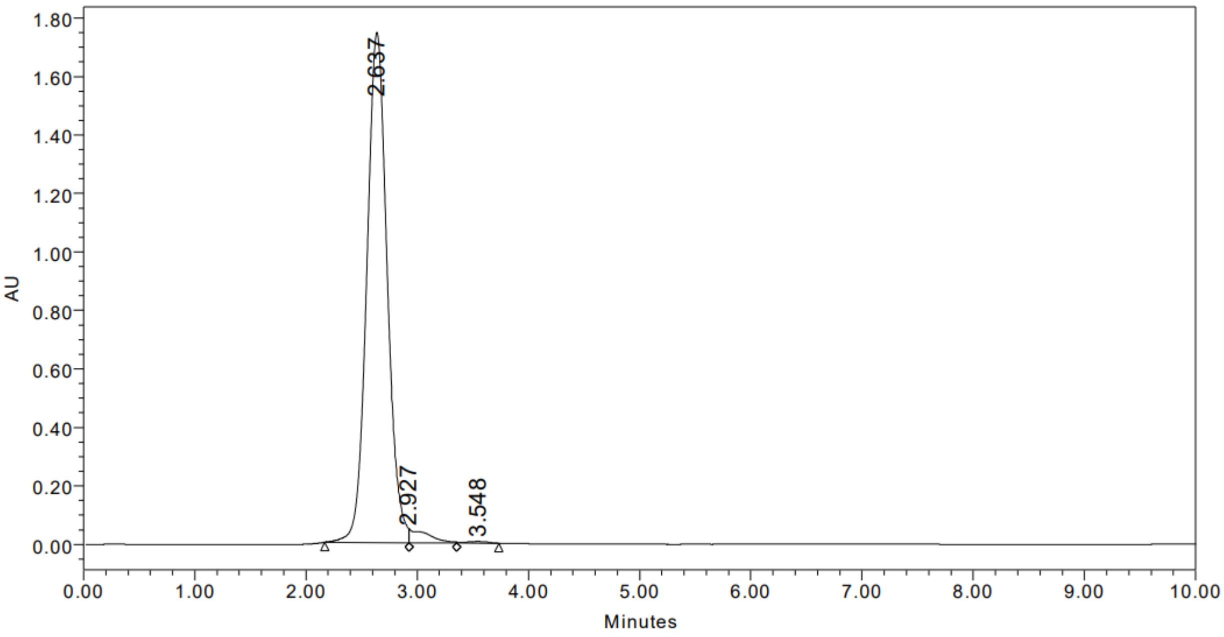

N-(1-phenethylazepan-4-yl)-N-phenyl-1H-pyrrole-3-carboxamide hydrochloride **(38)**

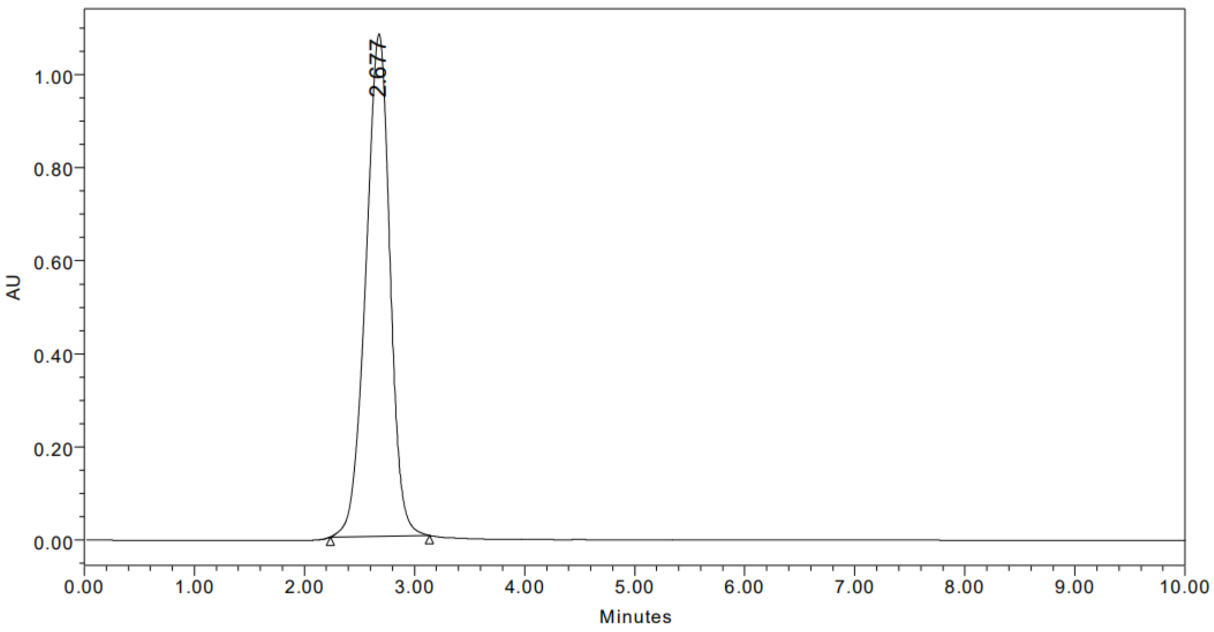

N-(1-allylazepan-4-yl)-N-phenylthiophene-3-carboxamide hydrochloride **(39)**

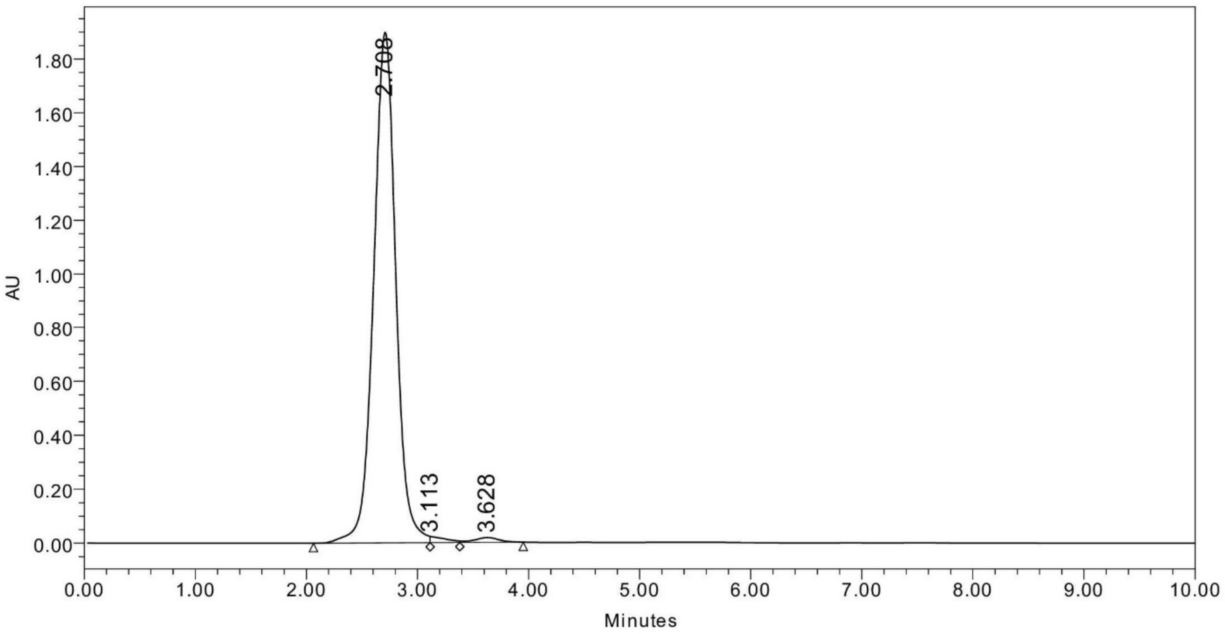

N-(1-(cyclopropylmethyl)azepan-4-yl)-N-phenylthiophene-3-carboxamide hydrochloride **(40)**

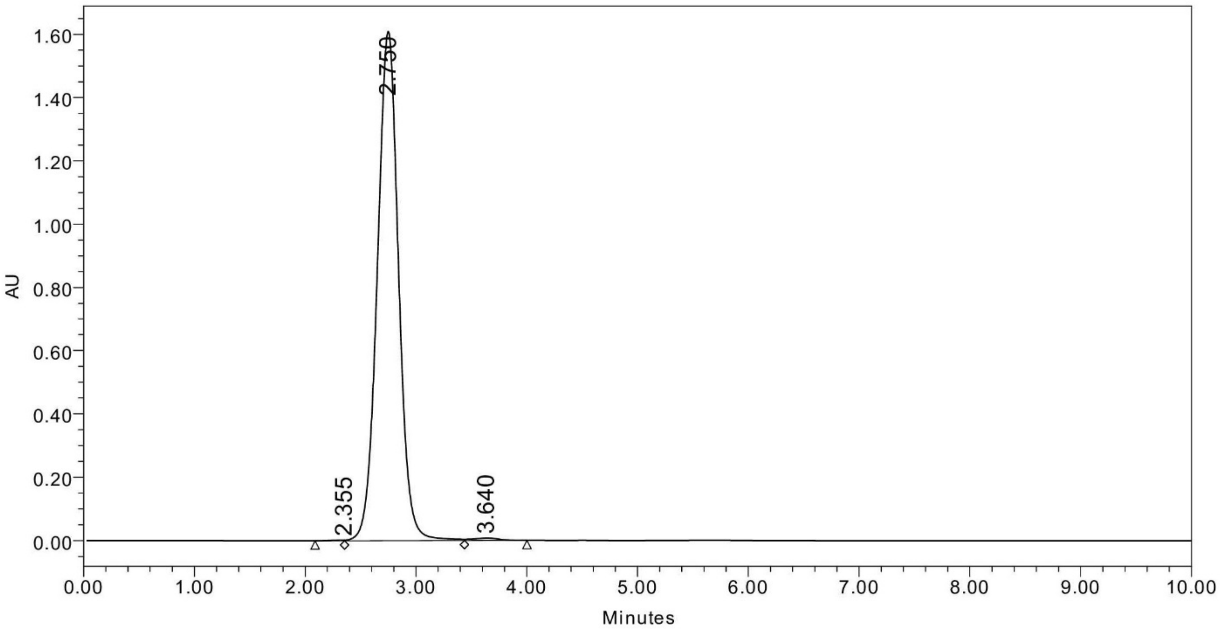

N-(1-(cyclobutylmethyl)azepan-4-yl)-N-phenylthiophene-3-carboxamide hydrochloride **(41)**

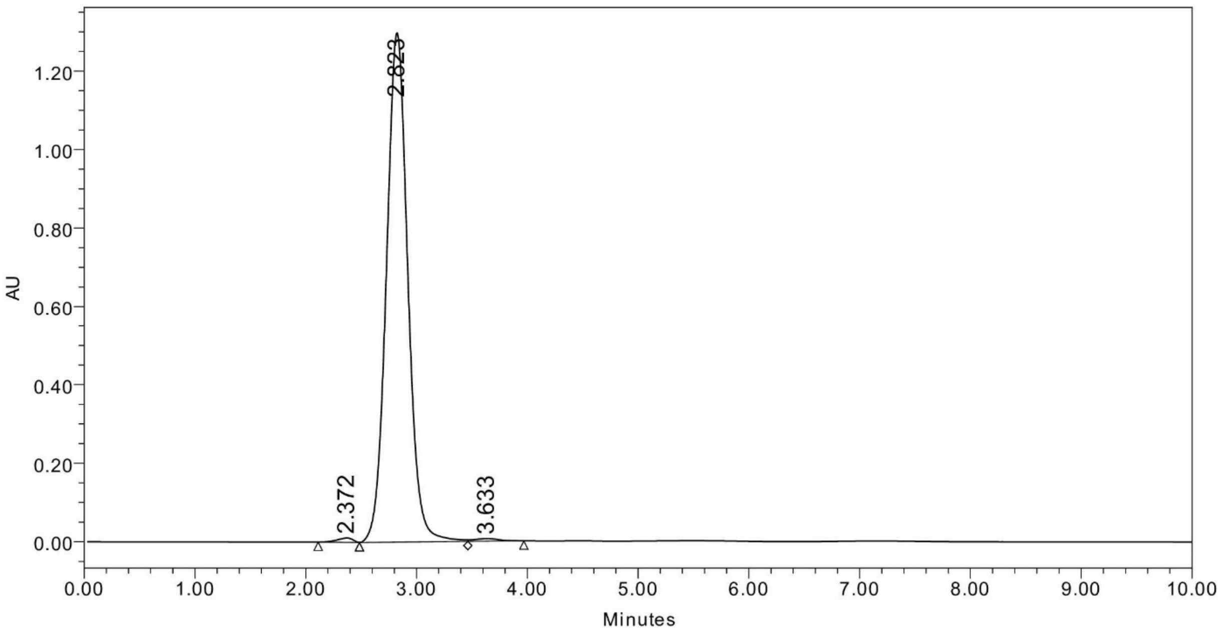

N-(1-(cyclopentylmethyl)azepan-4-yl)-N-phenylthiophene-3-carboxamide hydrochloride **(42)**

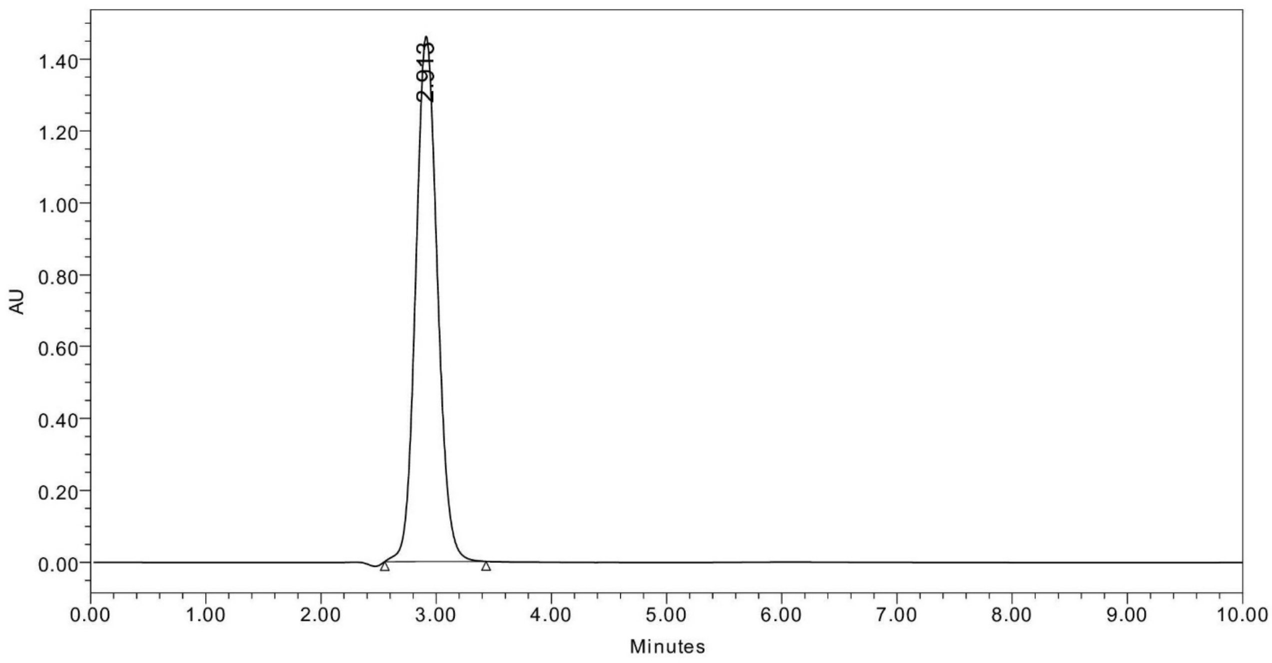

N-(1-(cyclohexylmethyl)azepan-4-yl)-N-phenylthiophene-3-carboxamide hydrochloride**(43)**

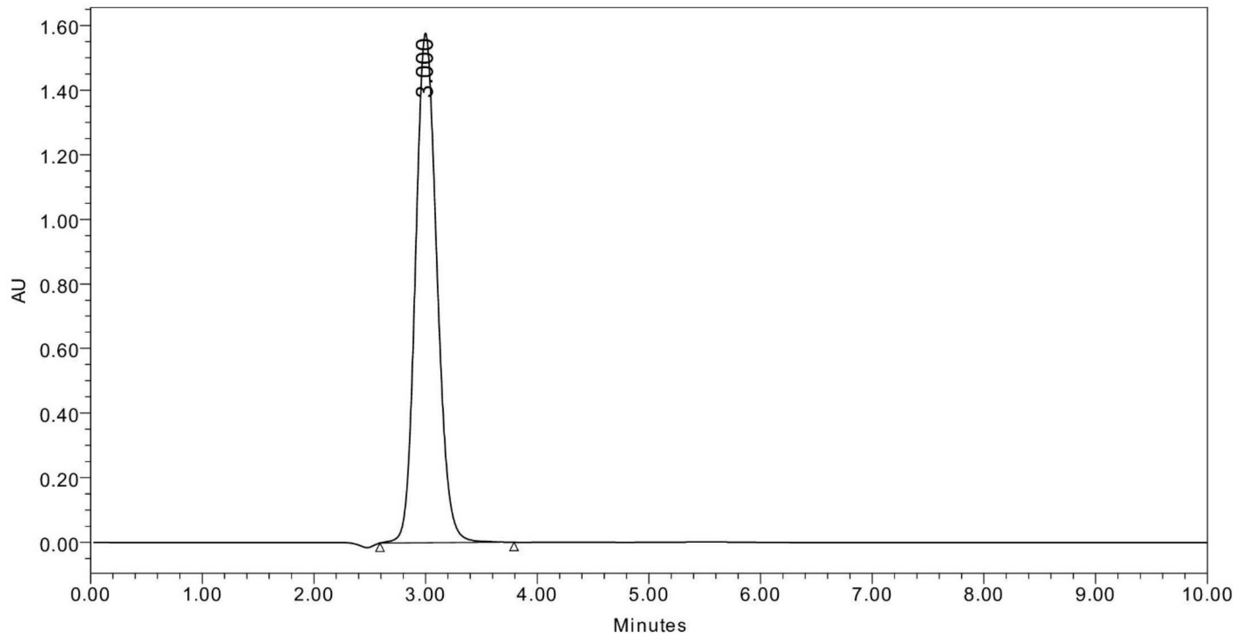

N-(1-benzylazepan-4-yl)-N-phenylthiophene-3-carboxamide hydrochloride **(44)**

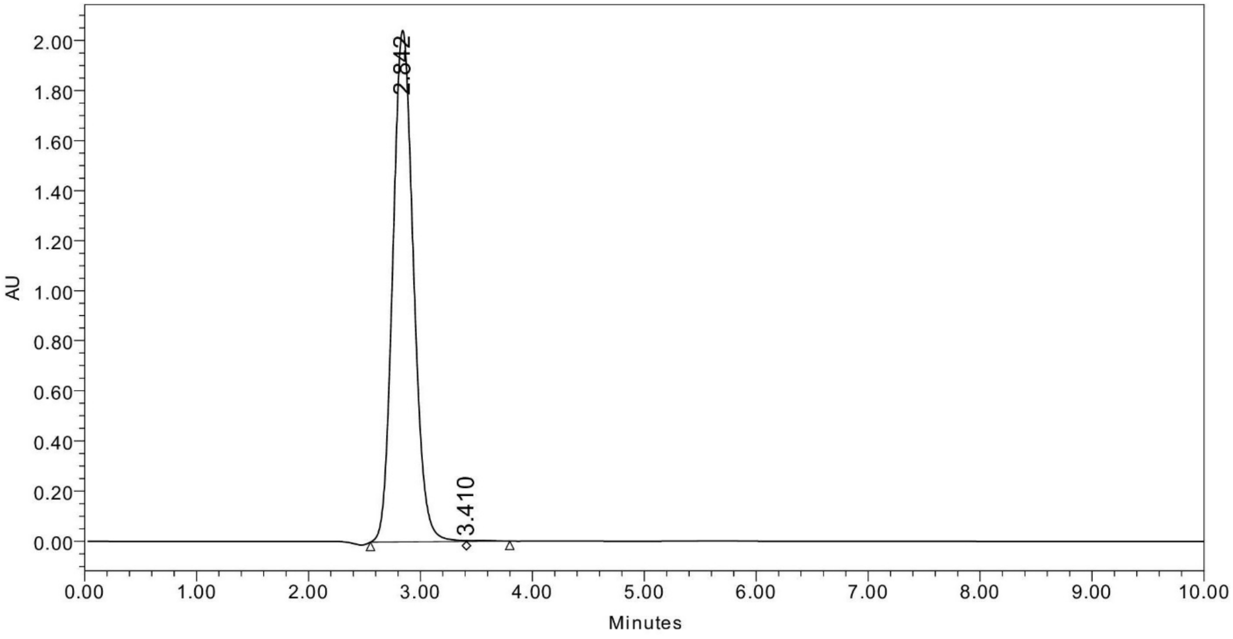

N-(1-phenethylazepan-4-yl)-N-phenylthiophene-3-carboxamide hydrochloride **(45)**

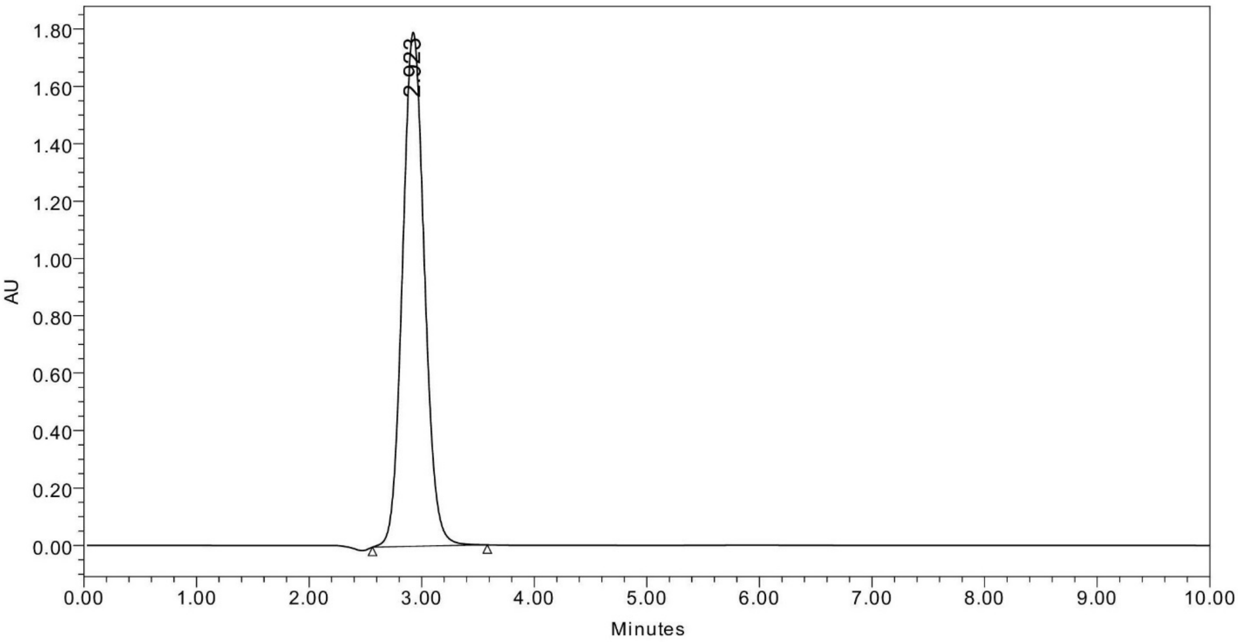

N-(1-allylazepan-4-yl)-N-phenylfuran-2-carboxamide hydrogen chloride **(46)**

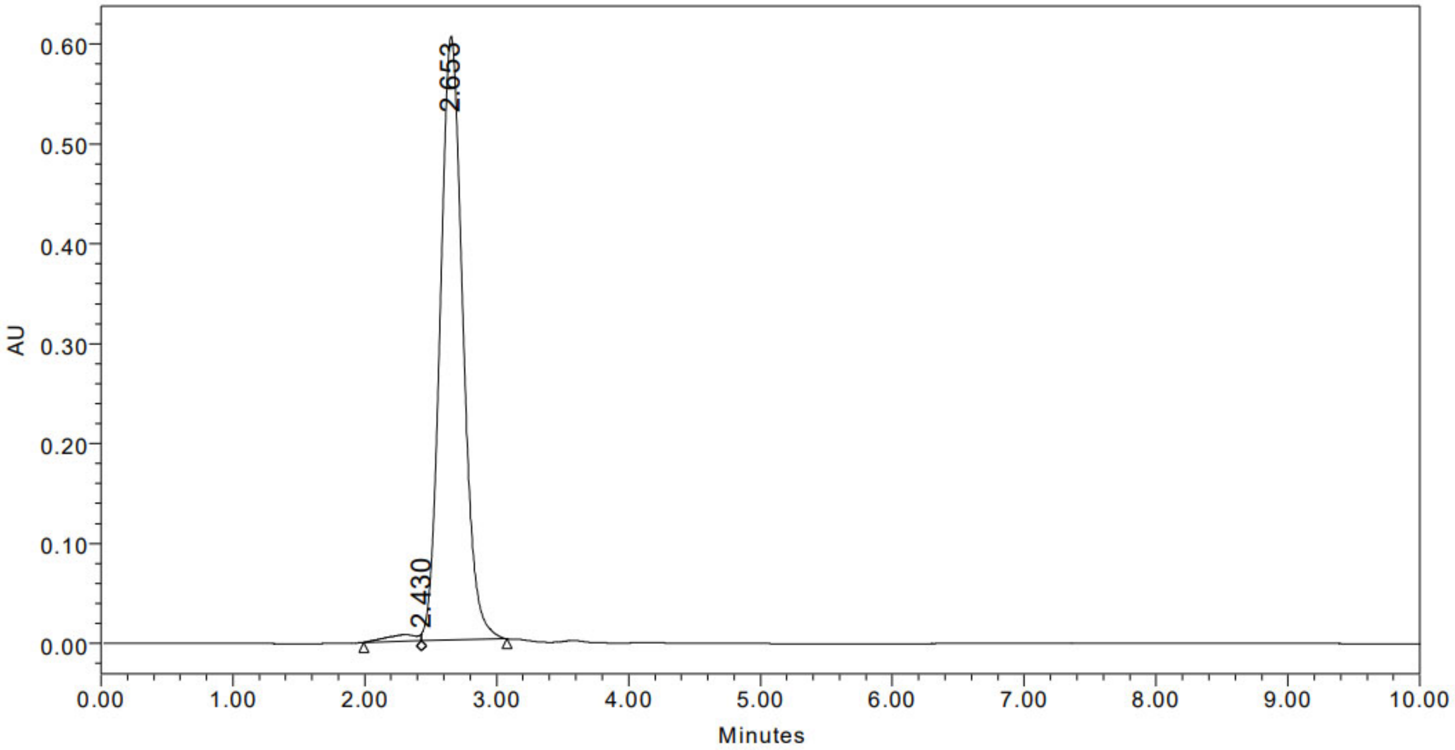

N-(1-(cyclopropylmethyl)azepan-4-yl)-N-phenylfuran-2-carboxamide hydrogen chloride **(47)**

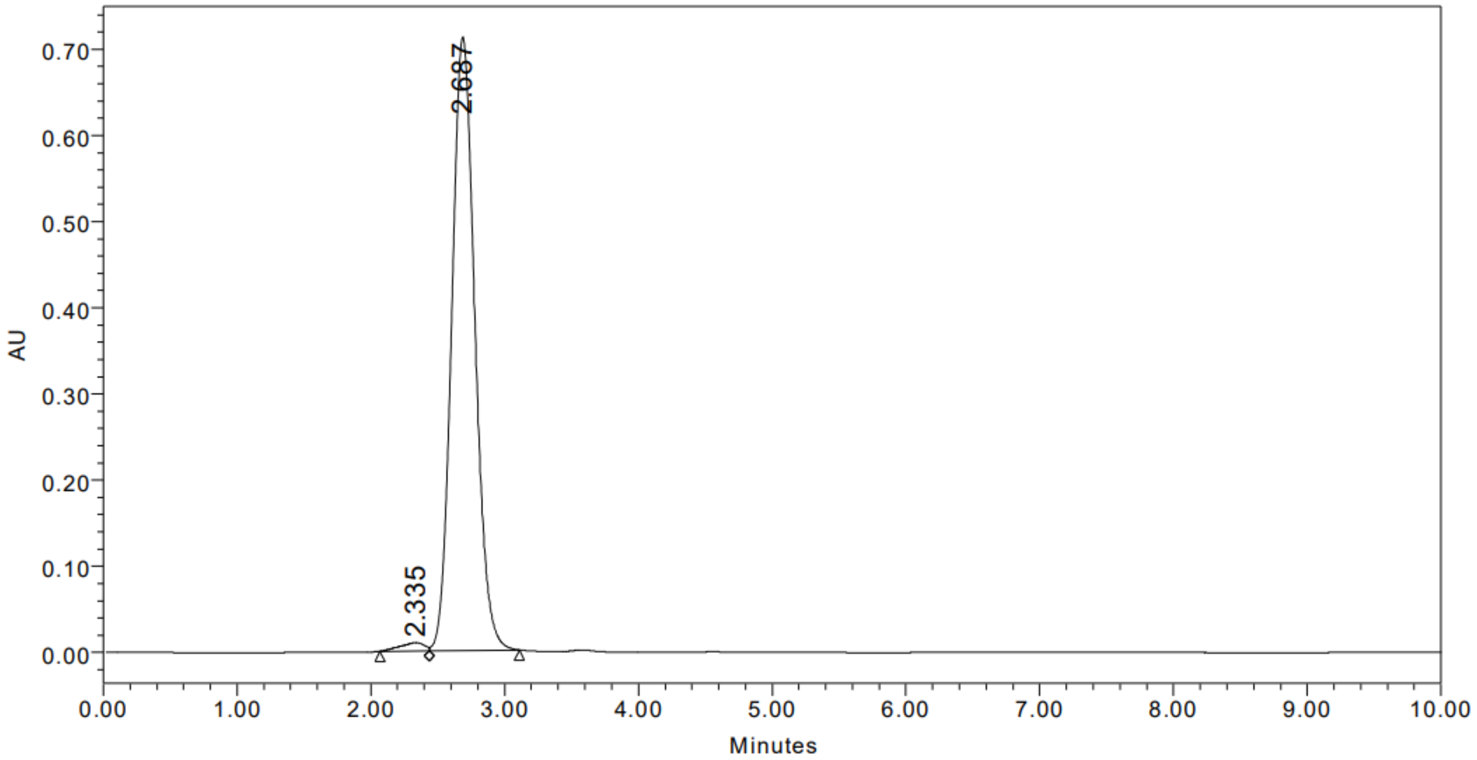

N-(1-(cyclobutylmethyl)azepan-4-yl)-N-phenylfuran-2-carboxamide hydrogen chloride **(48)**

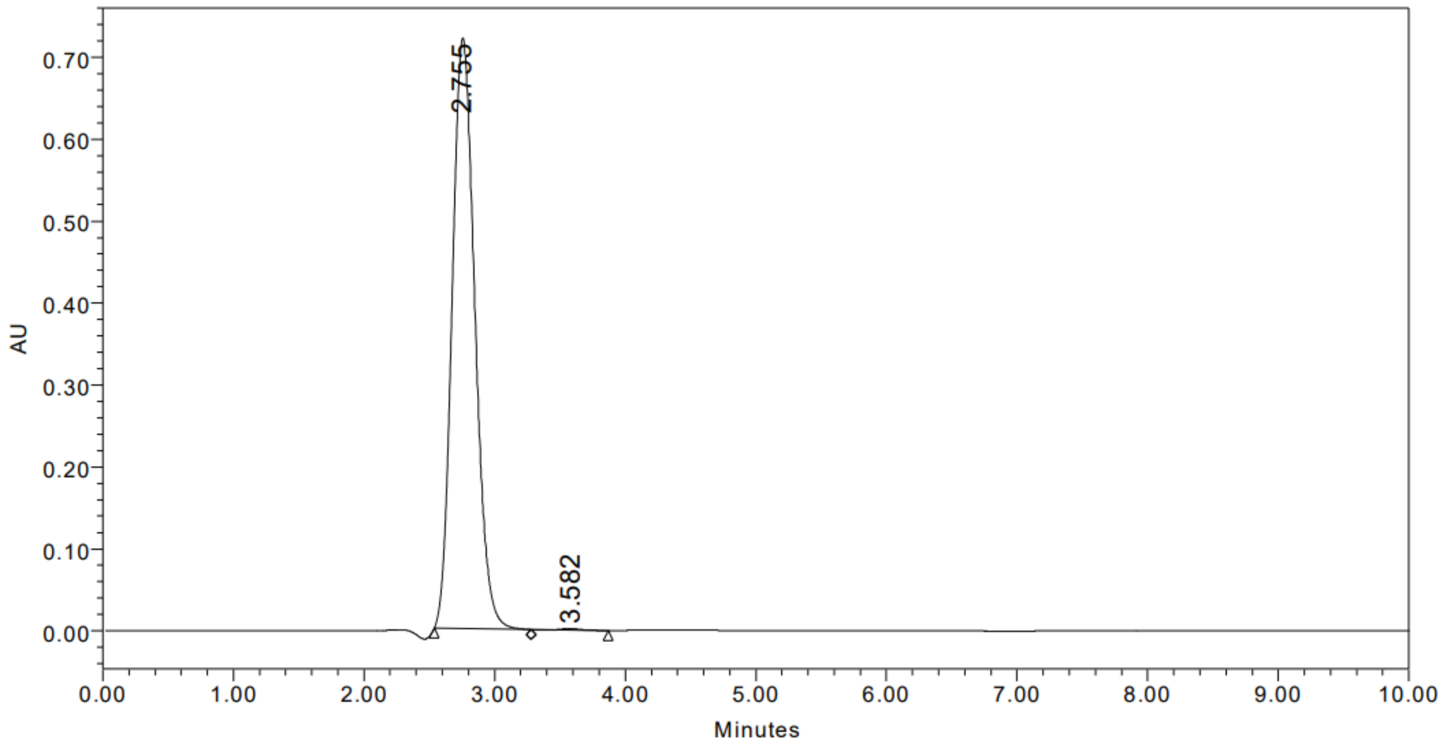

N-(1-(cyclopentylmethyl)azepan-4-yl)-N-phenylfuran-2-carboxamide hydrogen chloride **(49)**

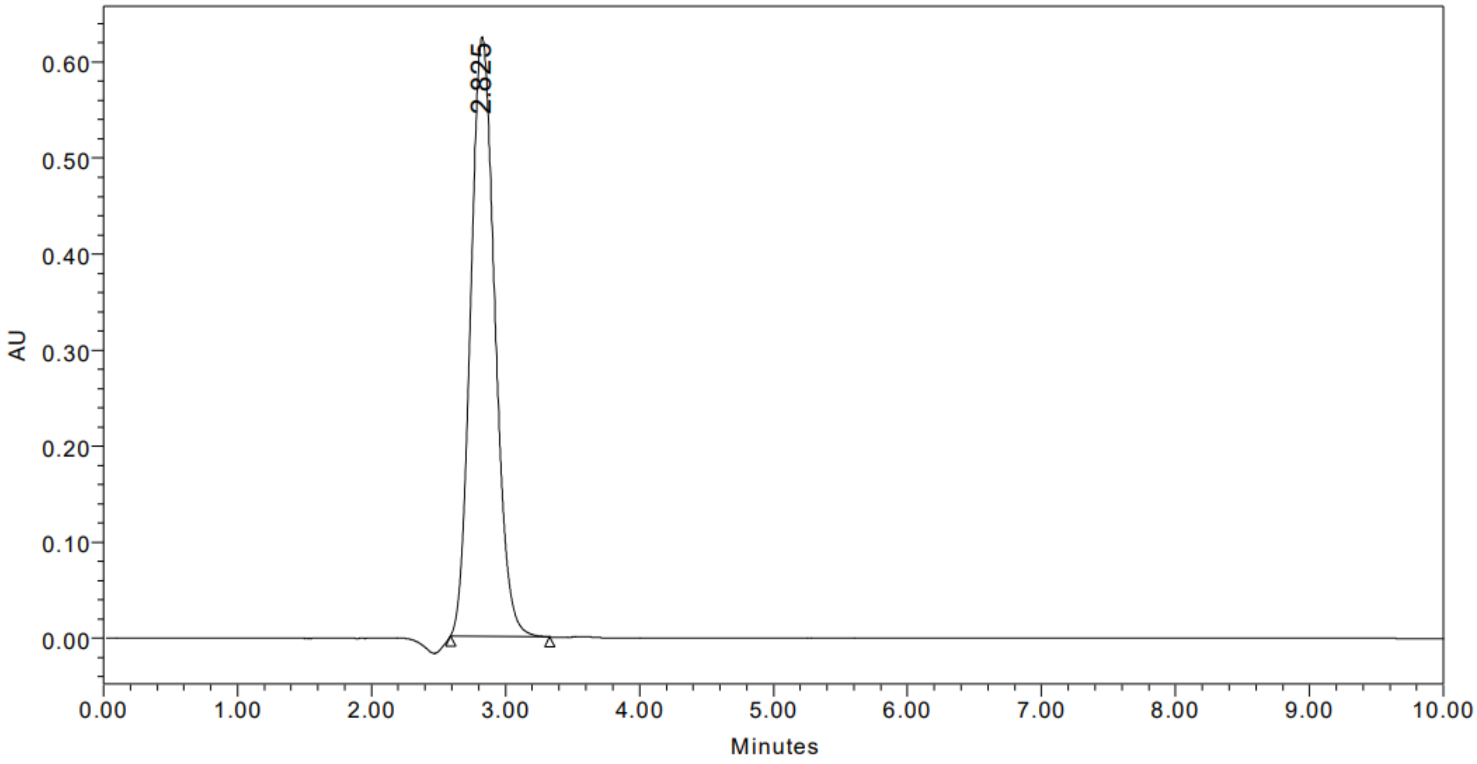

N-(1-(cyclohexylmethyl)azepan-4-yl)-N-phenylfuran-2-carboxamide hydrogen chloride **(50)**

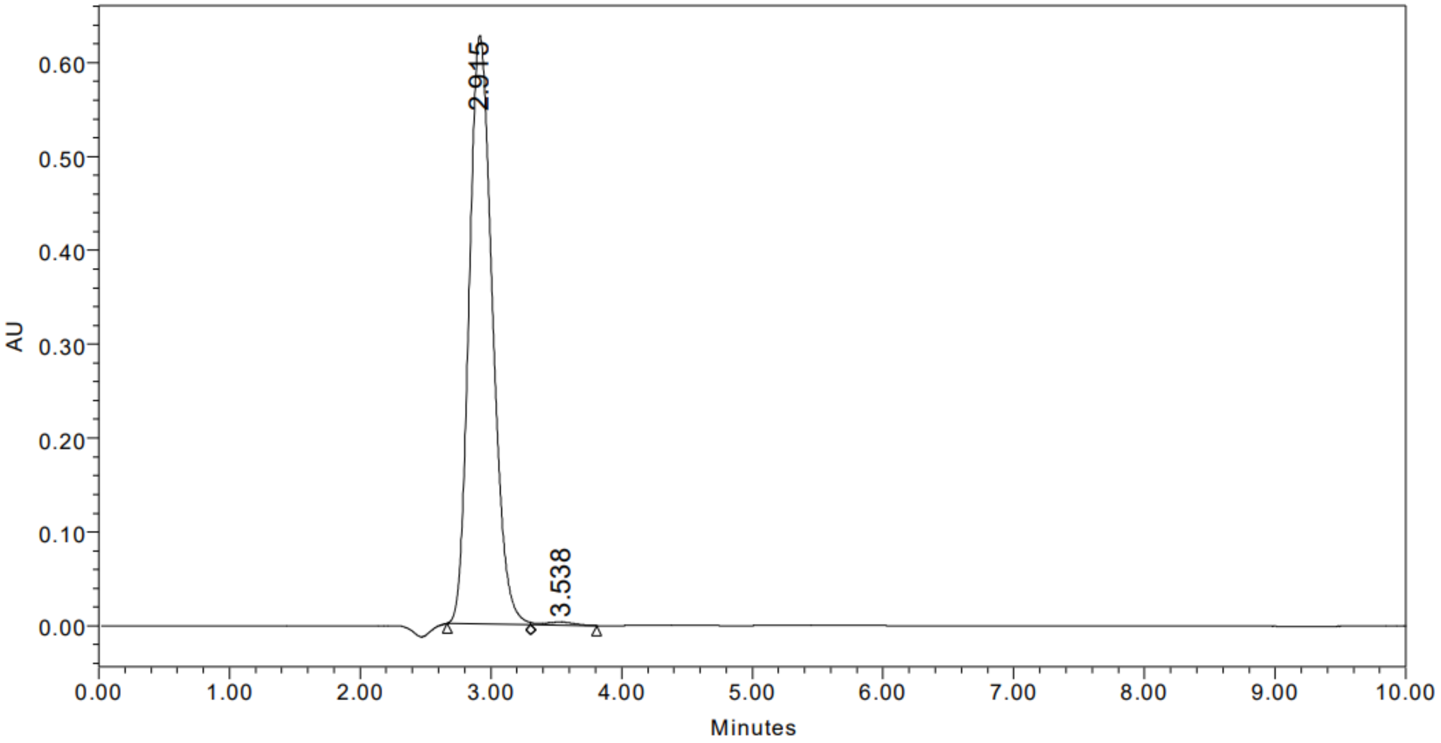

N-(1-benzylazepan-4-yl)-N-phenylfuran-2-carboxamide hydrogen chloride **(51)**

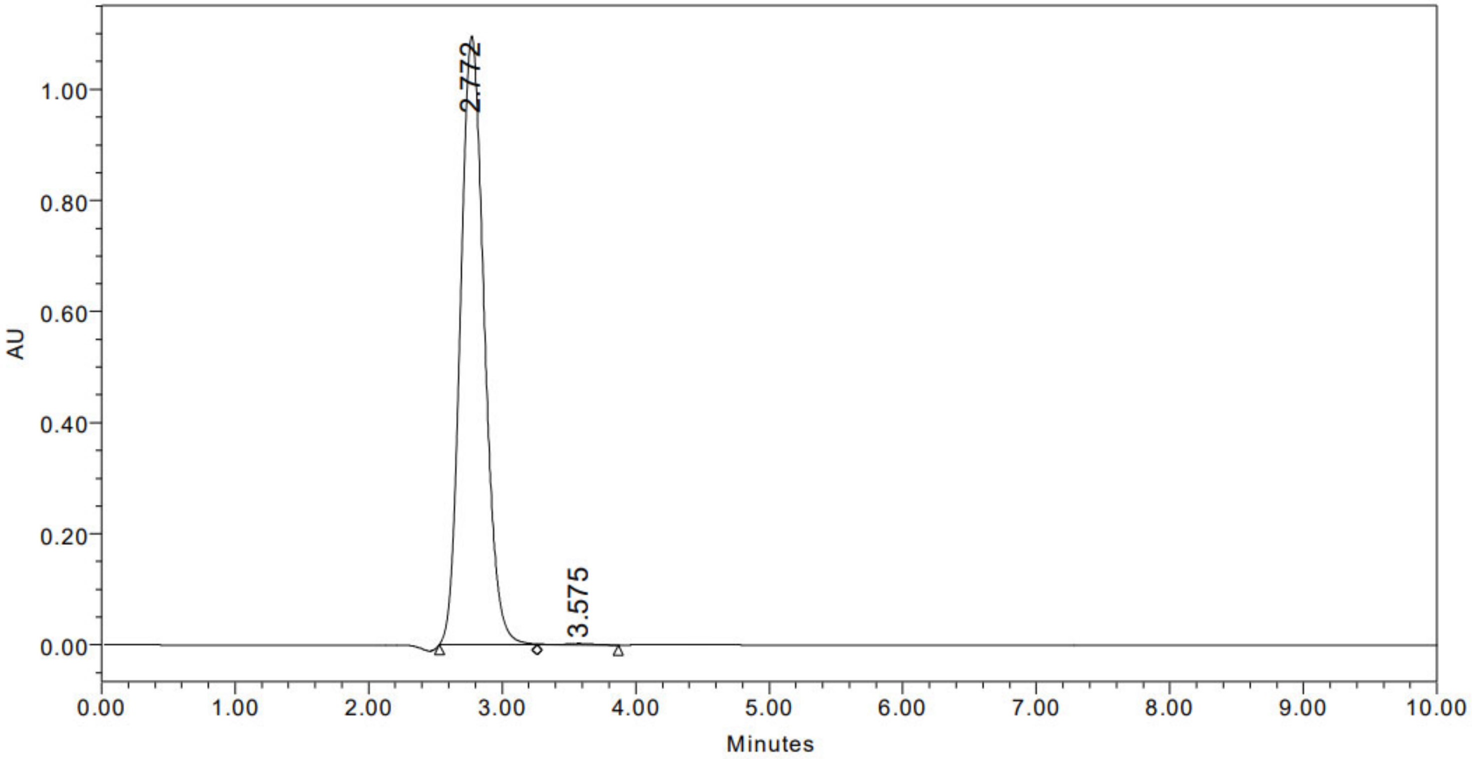

N-(1-phenethylazepan-4-yl)-N-phenylfuran-2-carboxamide hydrogen chloride **(52)**

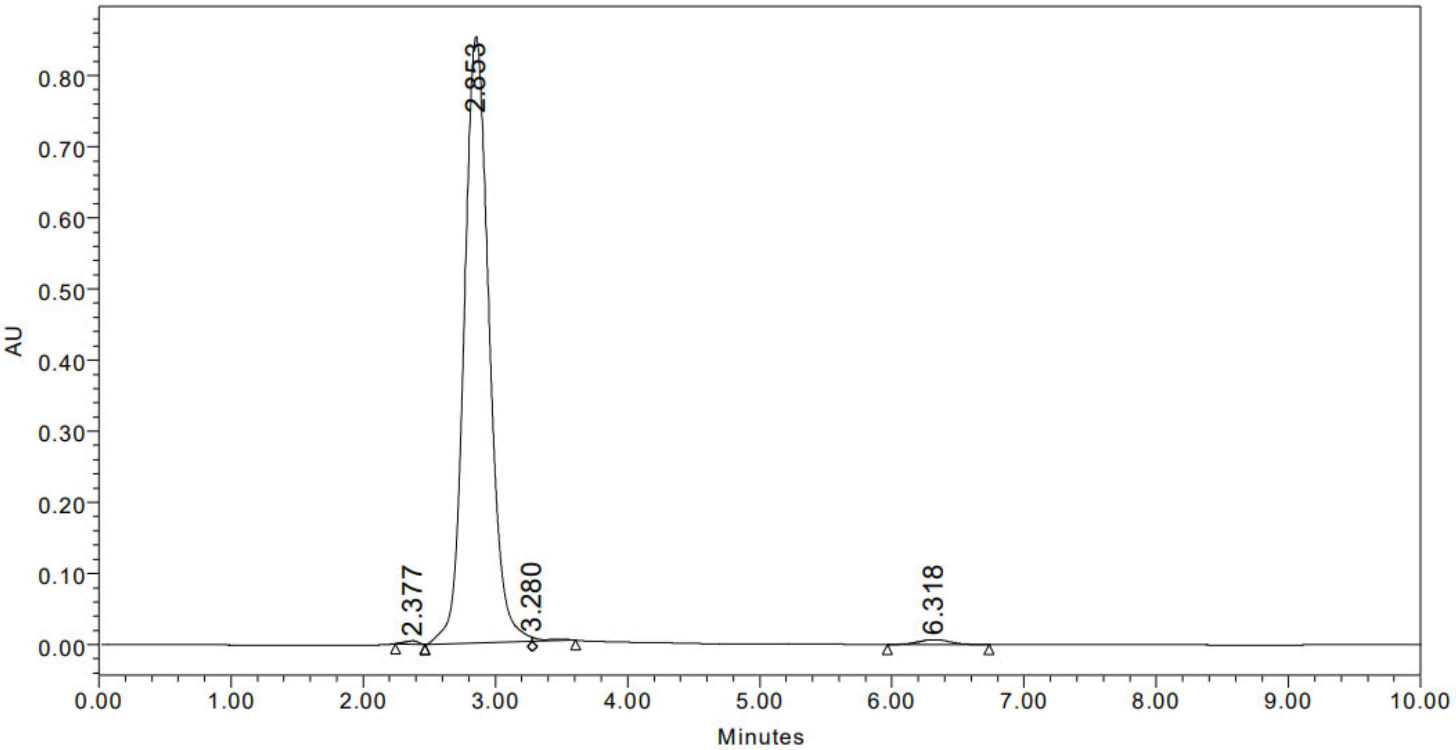

N-(1-allylazocan-5-yl)-N-phenylfuran-3-carboxamide hydrochloride **(53)**

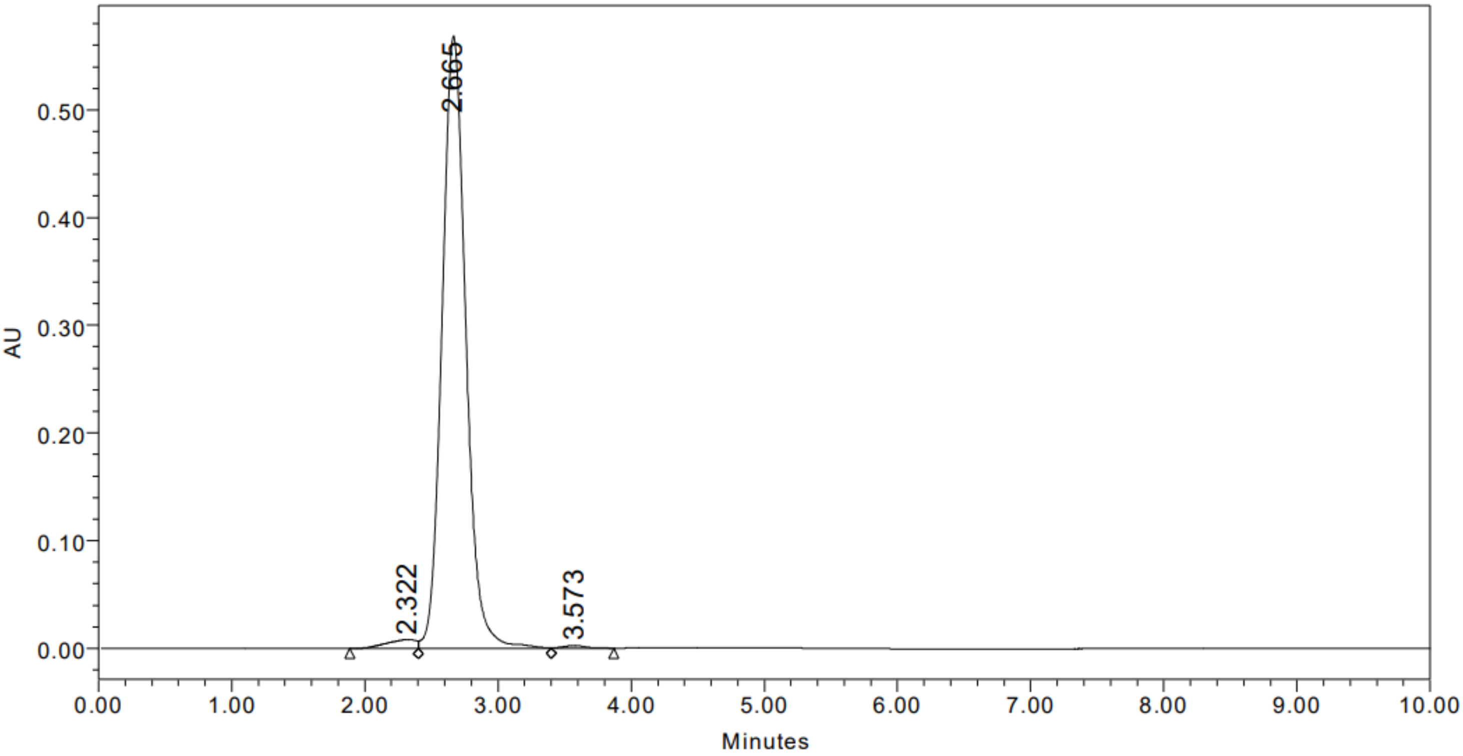

N-(1-(cyclopropylmethyl)azocan-5-yl)-N-phenylfuran-3-carboxamide hydrochloride **(54)**

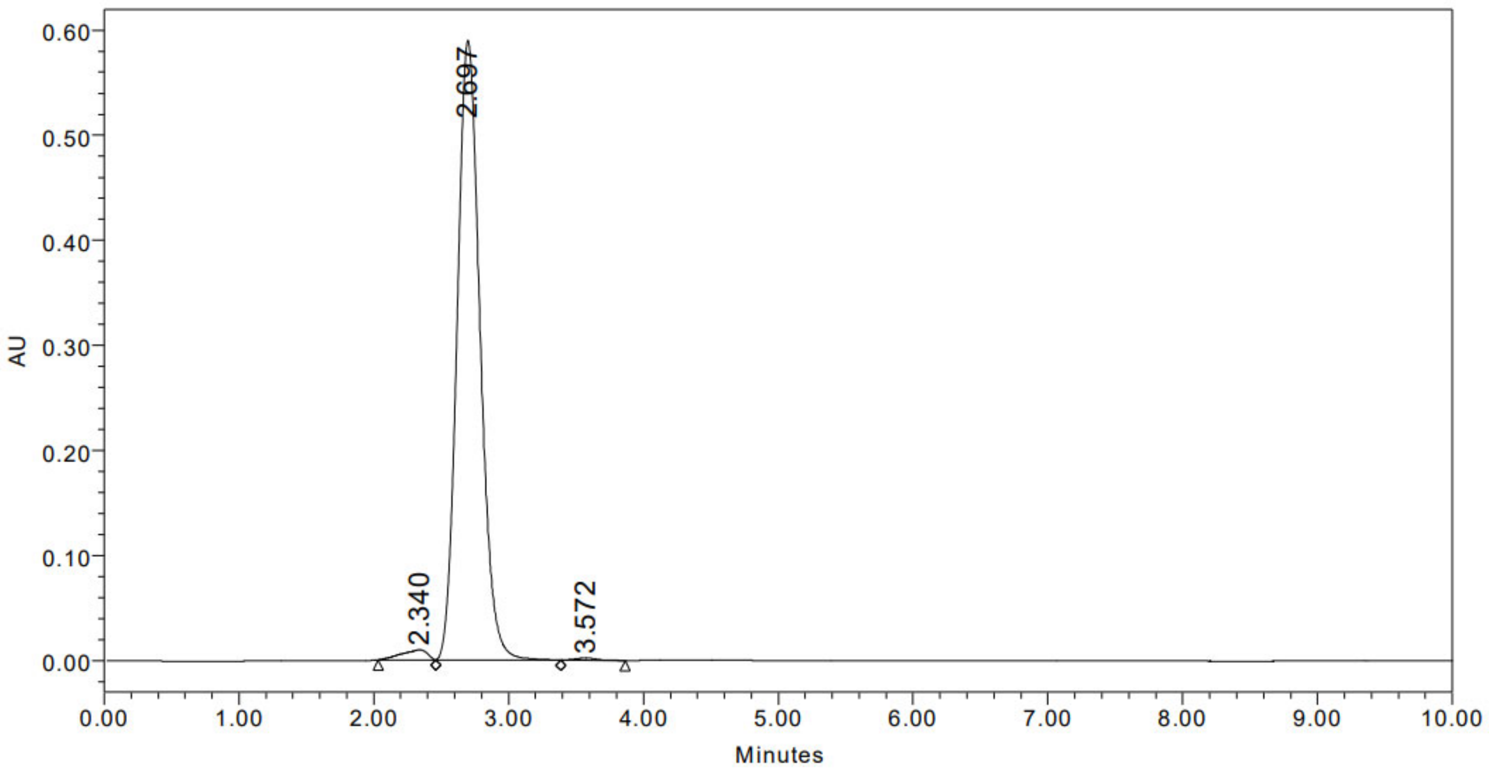

N-(1-(cyclobutylmethyl)azocan-5-yl)-N-phenylfuran-3-carboxamide hydrochloride**(55)**

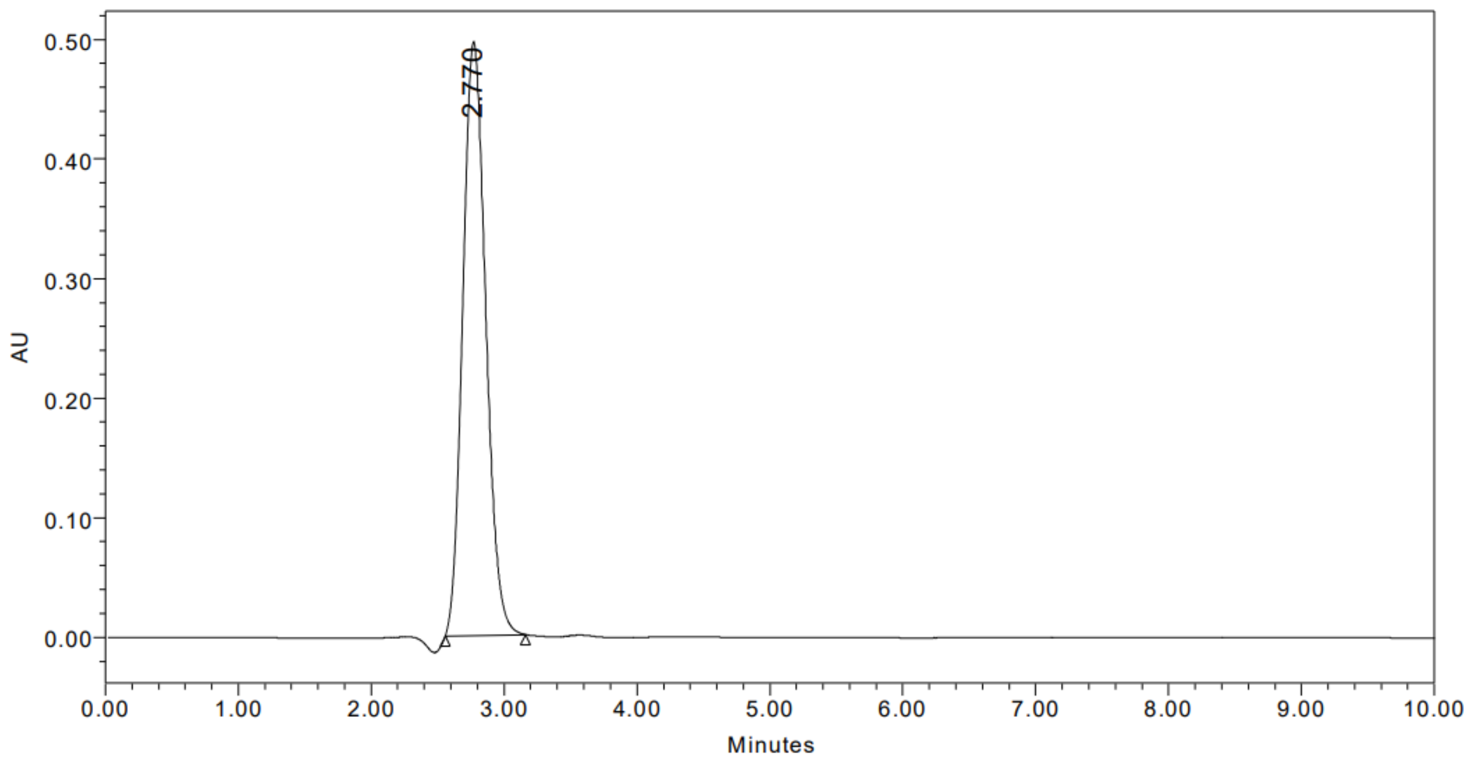

N-(1-(cyclopentylmethyl)azocan-5-yl)-N-phenylfuran-3-carboxamide hydrochloride **(56)**

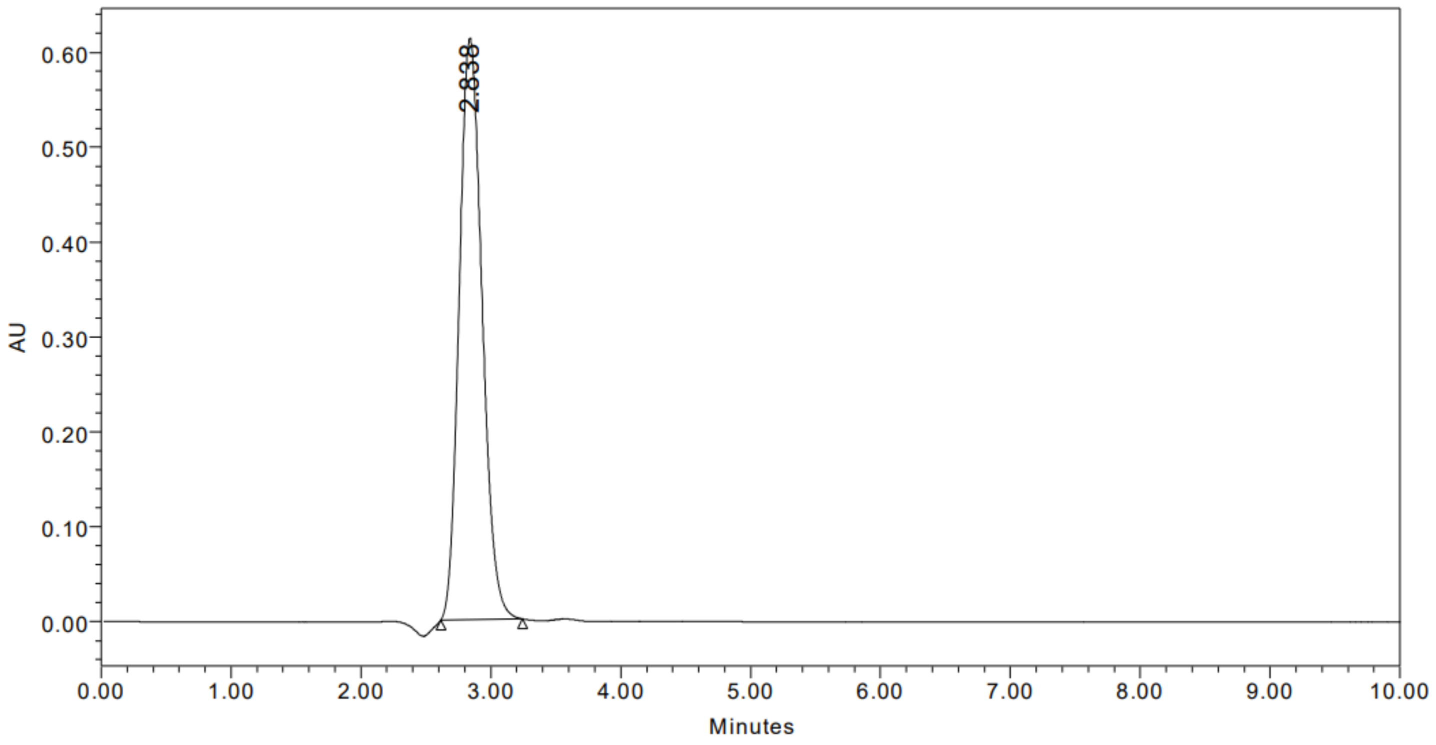

N-(1-(cyclohexylmethyl)azocan-5-yl)-N-phenylfuran-3-carboxamide hydrochloride **(57)**

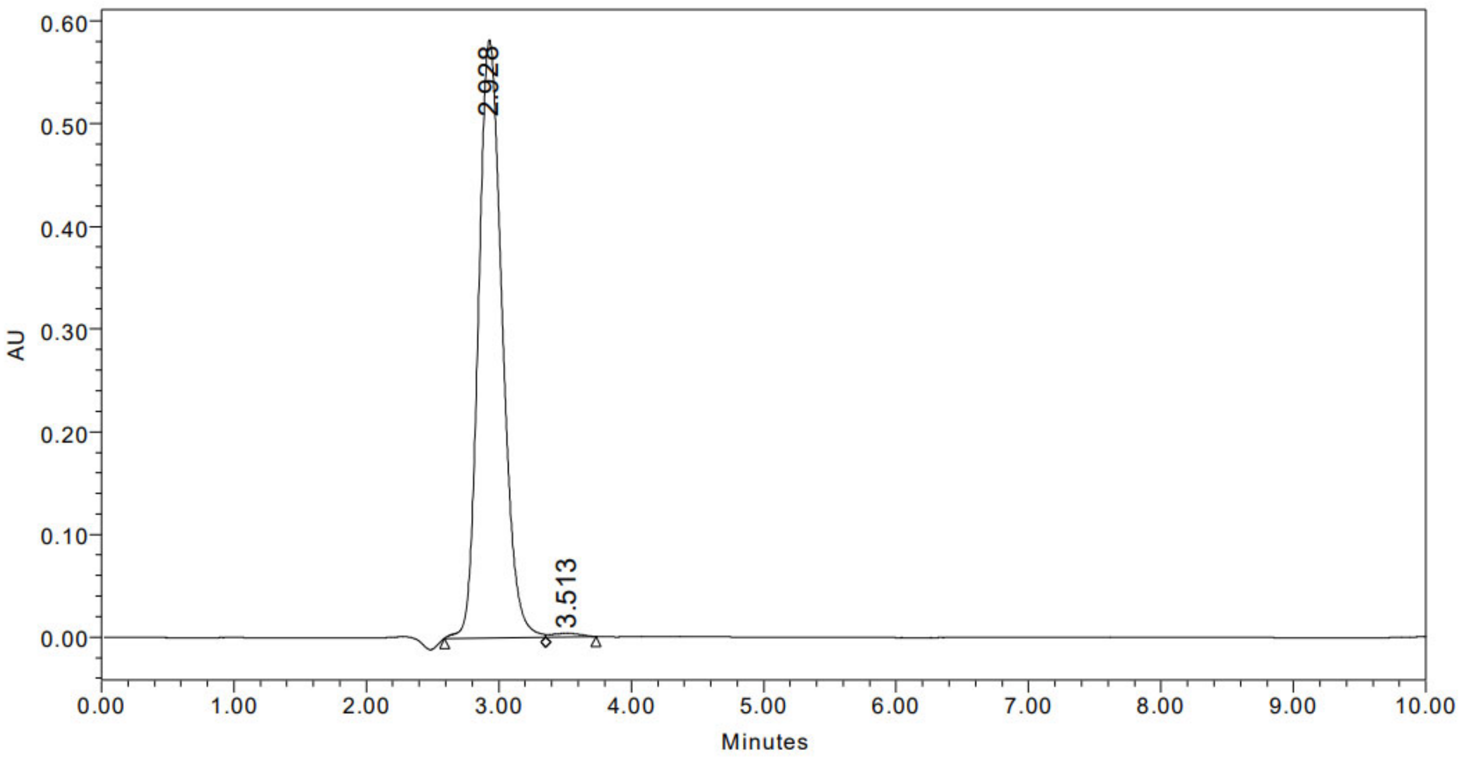

N-(1-benzylazocan-5-yl)-N-phenylfuran-3-carboxamide hydrochloride **(58)**

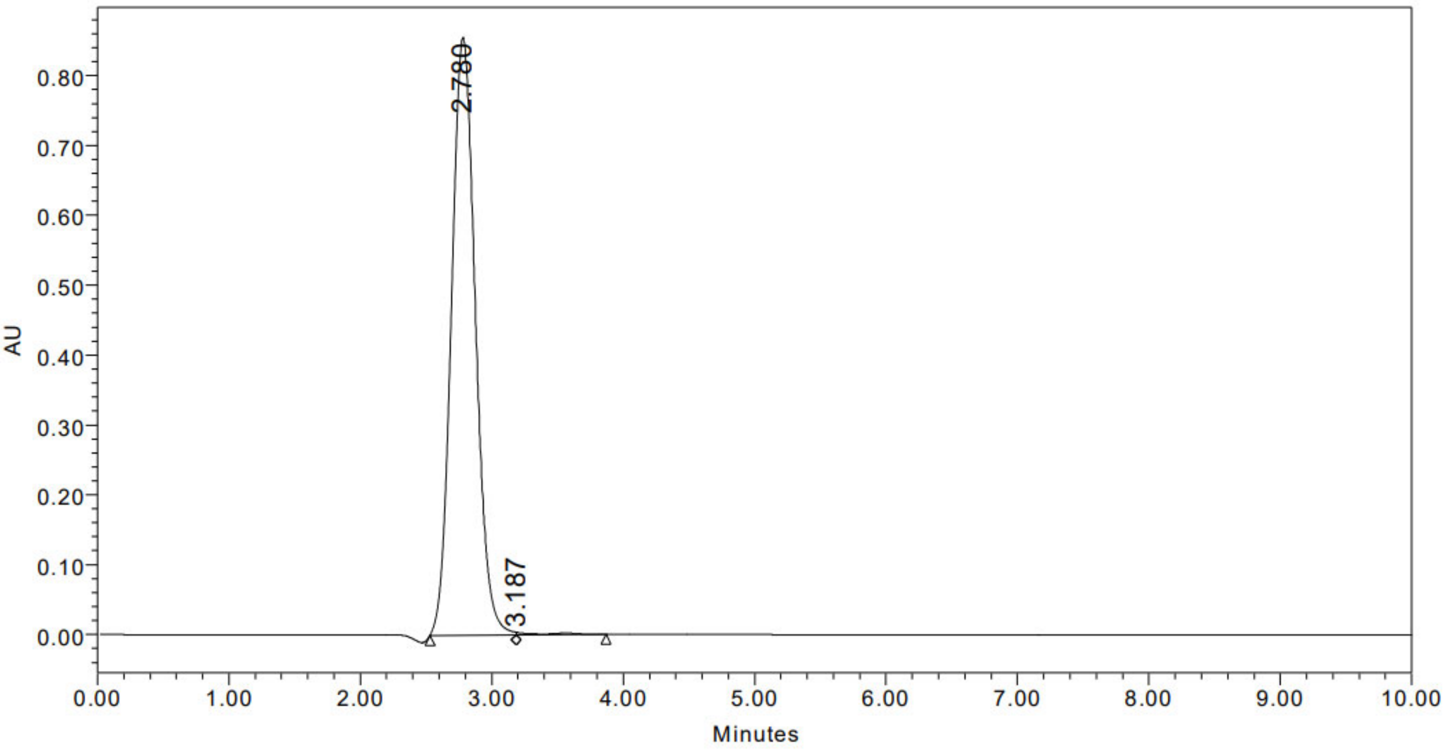

N-(1-phenethylazocan-5-yl)-N-phenylfuran-3-carboxamide hydrochloride **(59)**

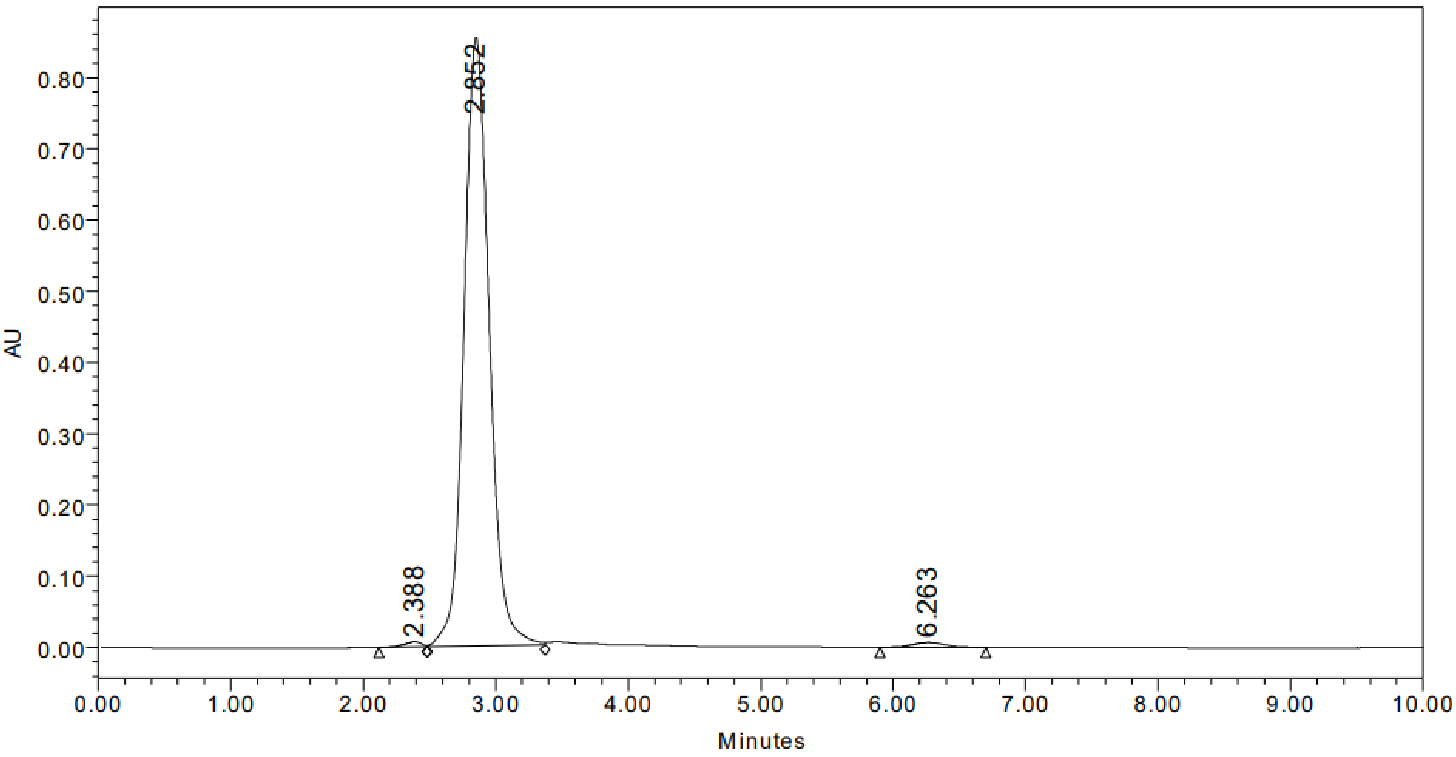

N-(1-allylazepan-4-yl)-N-phenylthiophene-2-carboxamide hydrogen chloride **(60)**

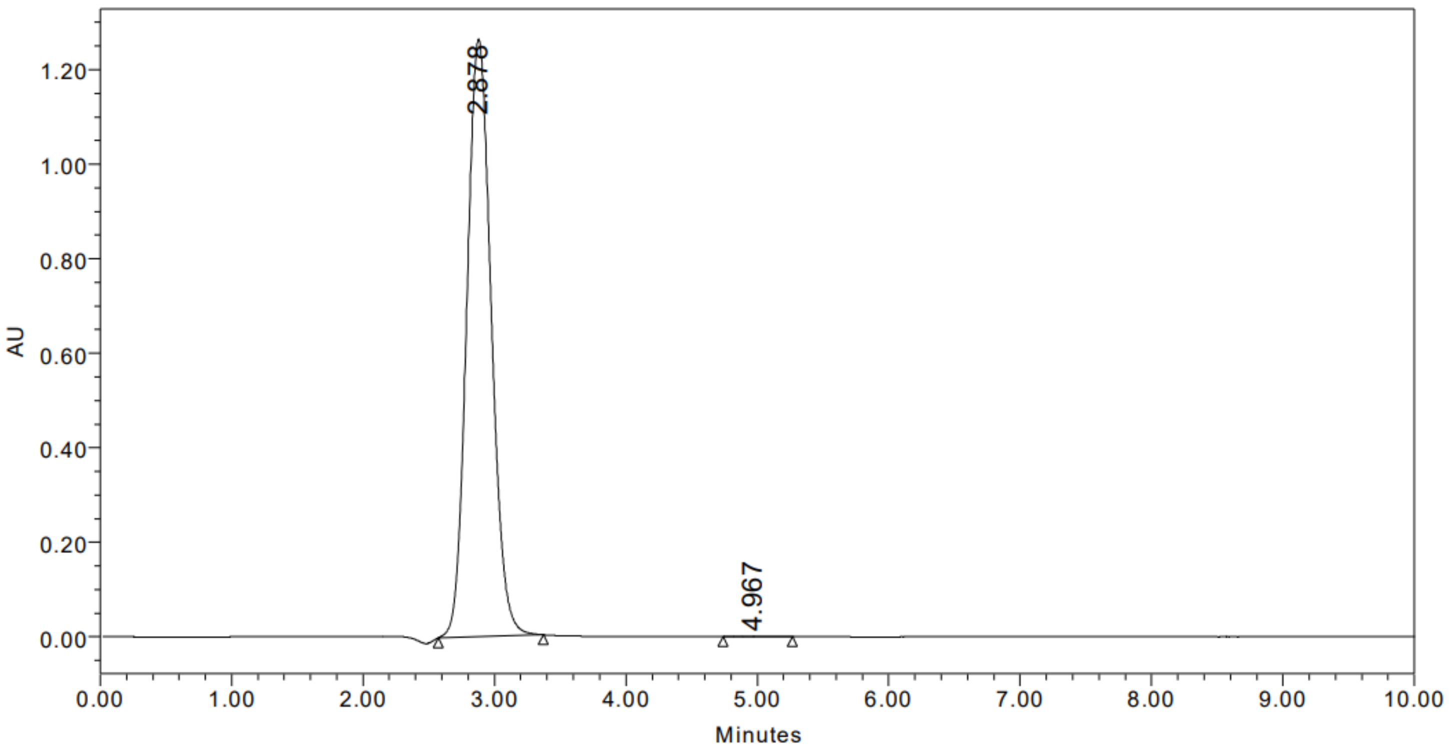

N-(1-(cyclopropylmethyl)azepan-4-yl)-N-phenylthiophene-2-carboxamide hydrogen chloride **(61)**

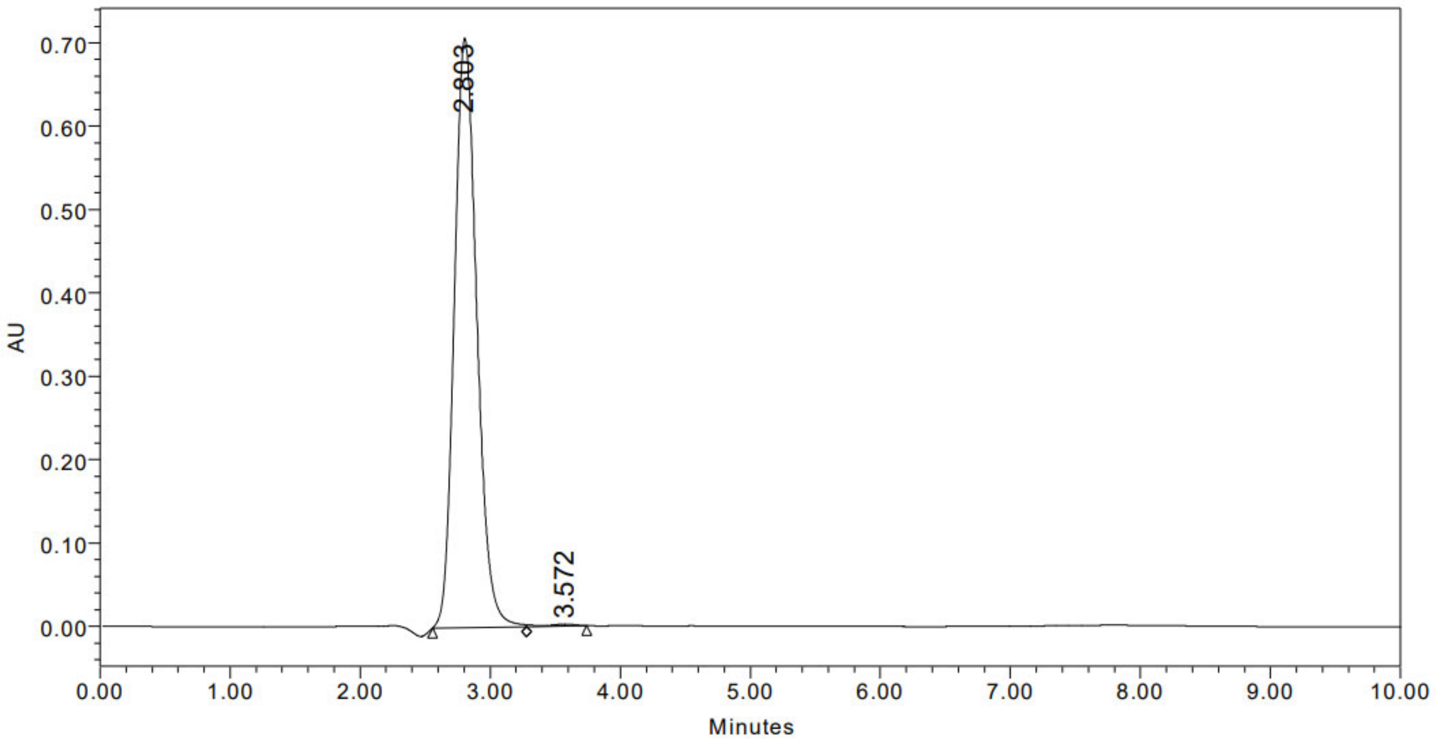

N-(1-(cyclobutylmethyl)azepan-4-yl)-N-phenylthiophene-2-carboxamide hydrogen chloride **(62)**

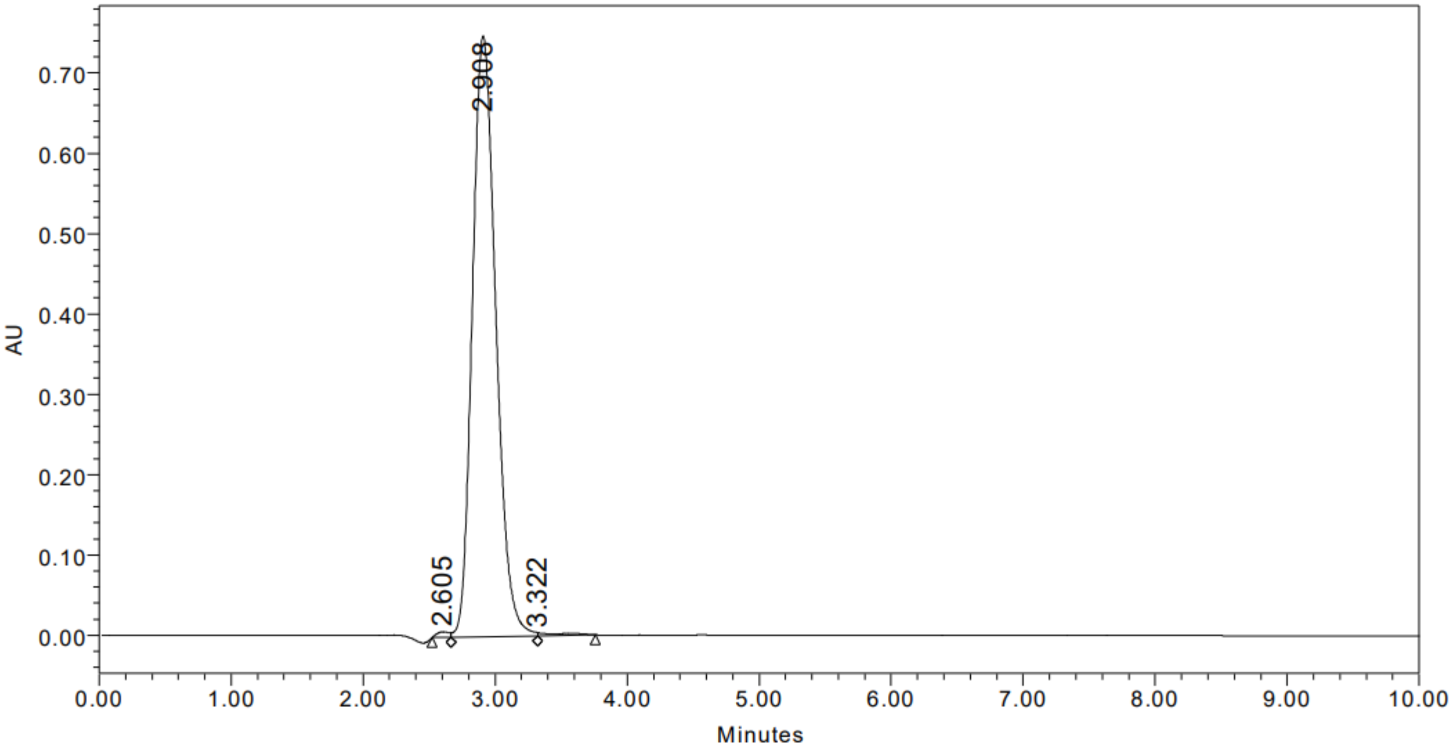

N-(1-(cyclopentylmethyl)azepan-4-yl)-N-phenylthiophene-2-carboxamide hydrogen chloride **(63)**

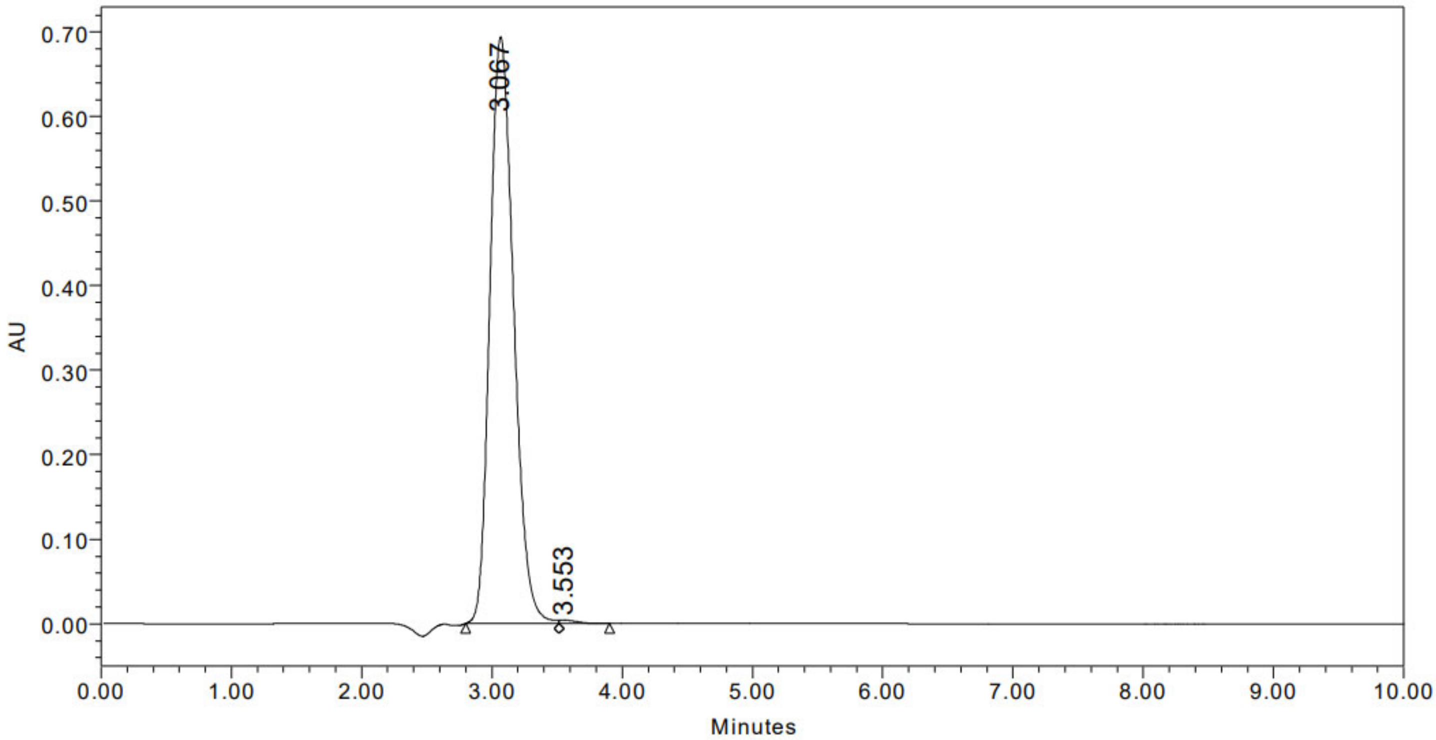

N-(1-(cyclohexylmethyl)azepan-4-yl)-N-phenylthiophene-2-carboxamide hydrogen chloride **(64)**

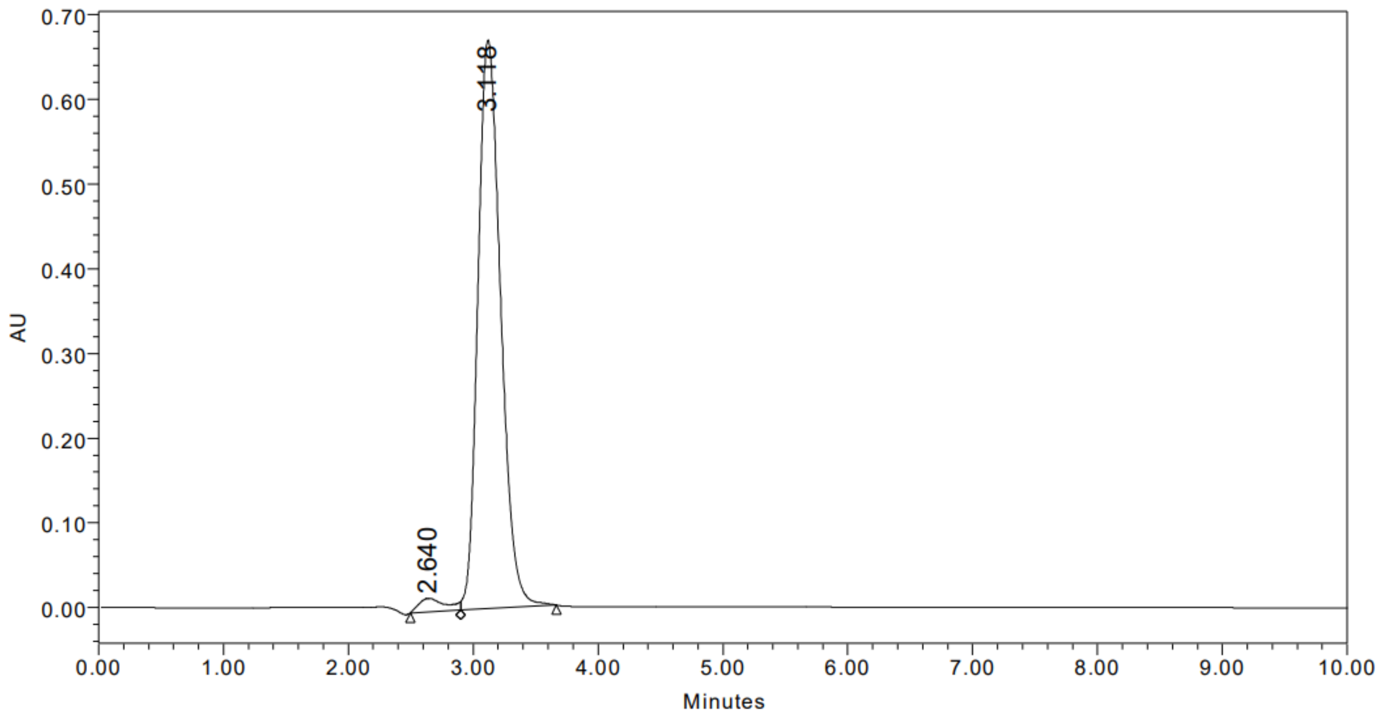

N-(1-benzylazepan-4-yl)-N-phenylthiophene-2-carboxamide hydrogen chloride **(65)**

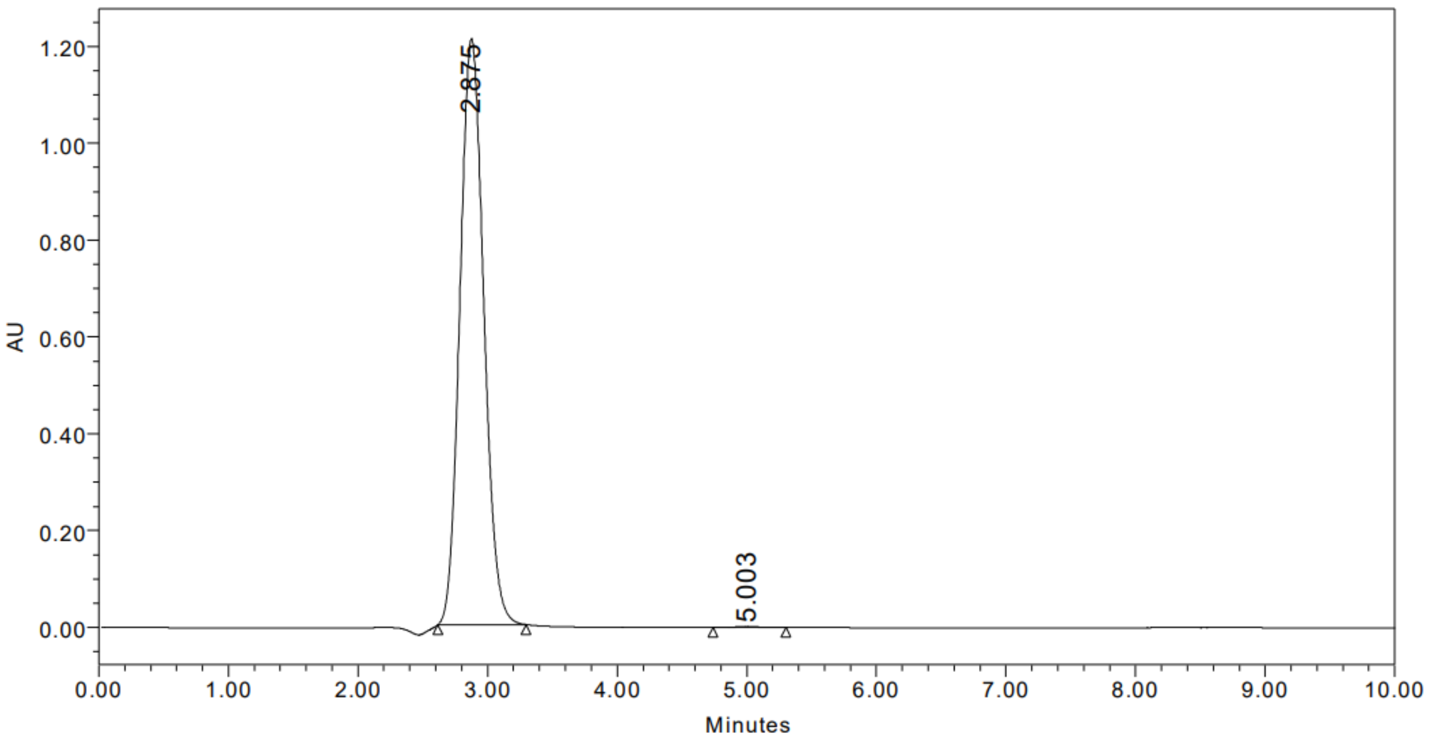

N-(1-phenethylazepan-4-yl)-N-phenylthiophene-2-carboxamide hydrogen chloride **(66)**

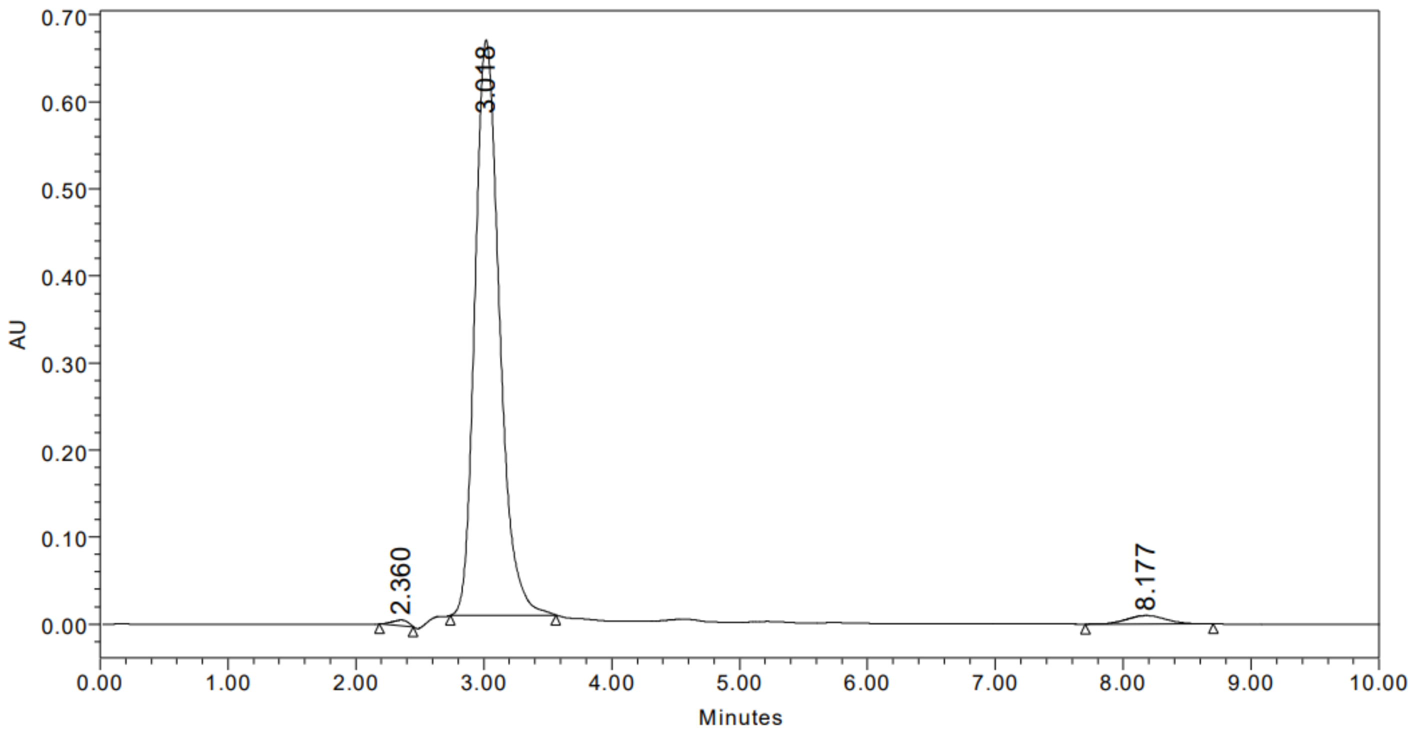

N-(1-allylazepan-4-yl)-N-phenyl-1H-pyrrole-2-carboxamide hydrogen chloride **(67)**

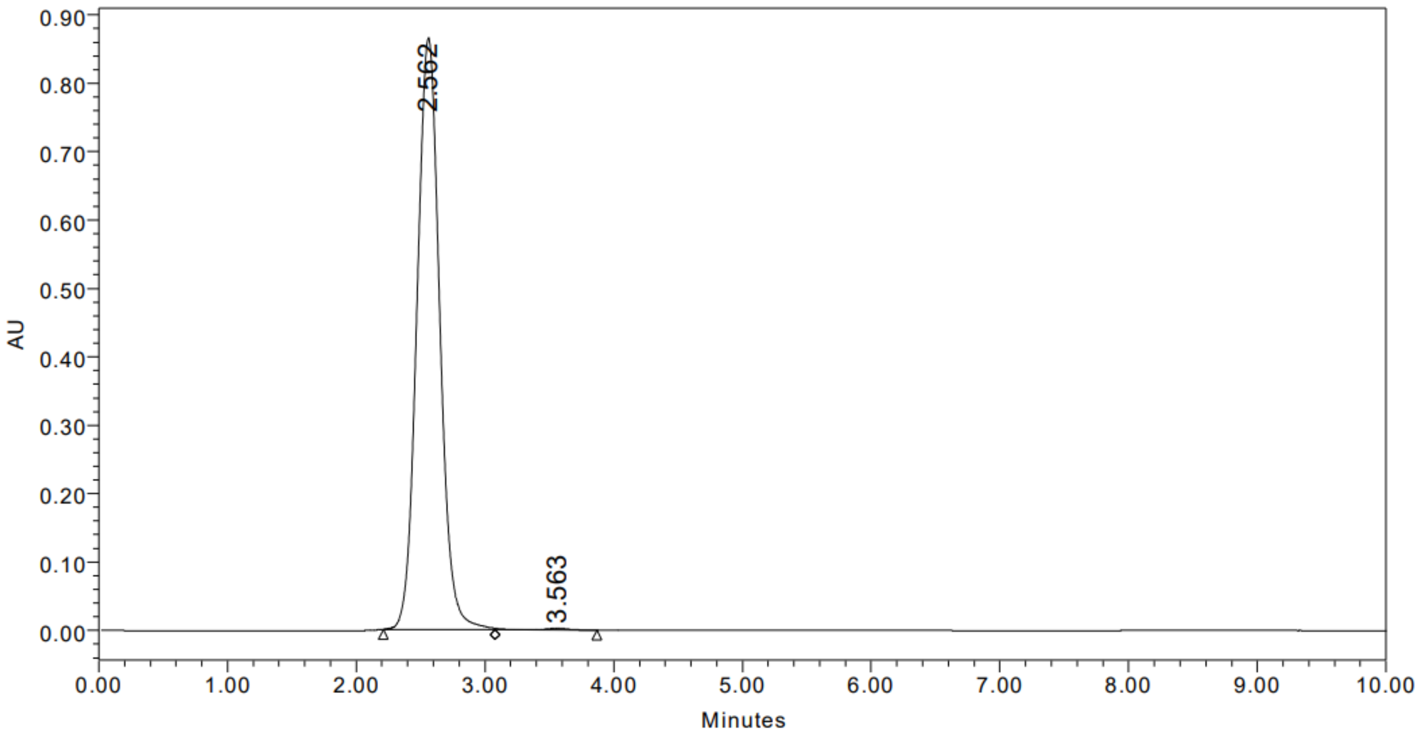

N-(1-(cyclopropylmethyl)azepan-4-yl)-N-phenyl-1H-pyrrole-2-carboxamide hydrogen chloride **(68)**

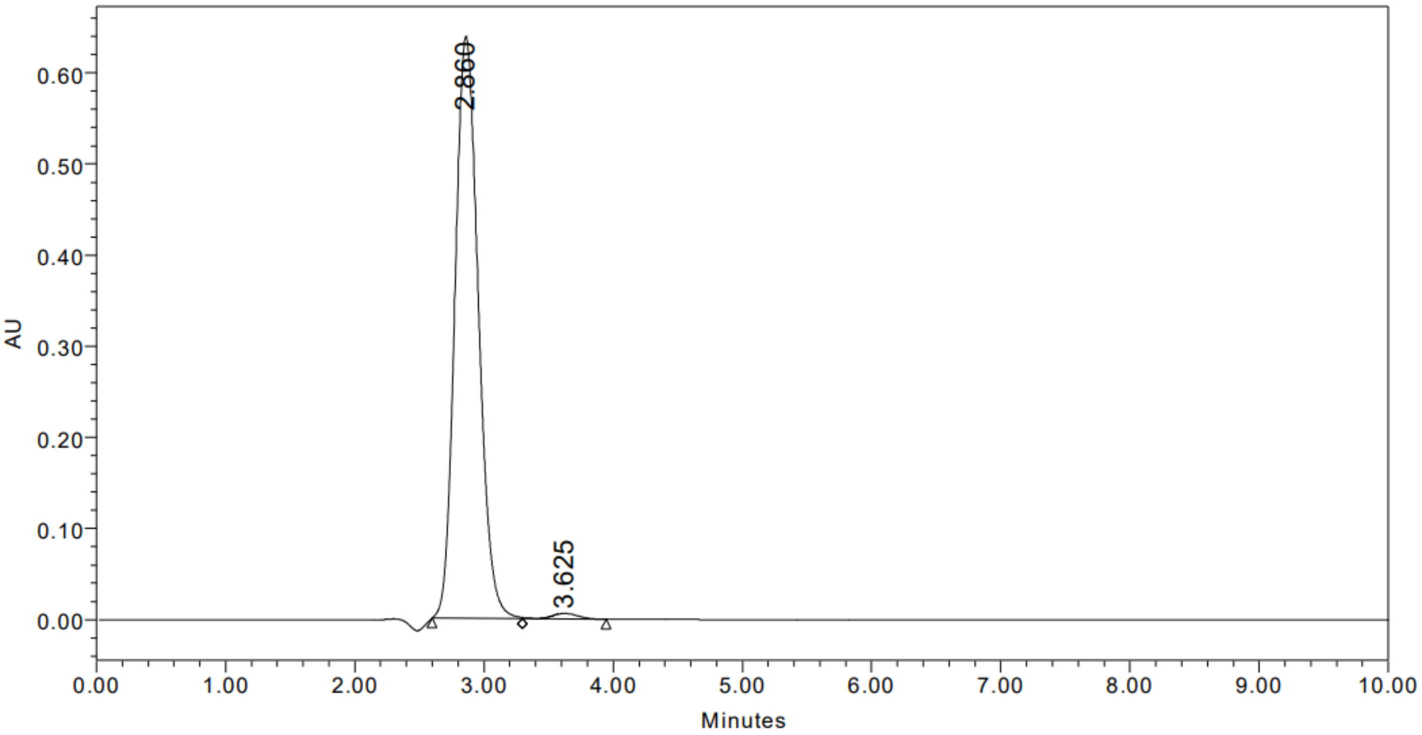

N-(1-(cyclobutylmethyl)azepan-4-yl)-N-phenyl-1H-pyrrole-2-carboxamide hydrogen chloride **(69)**

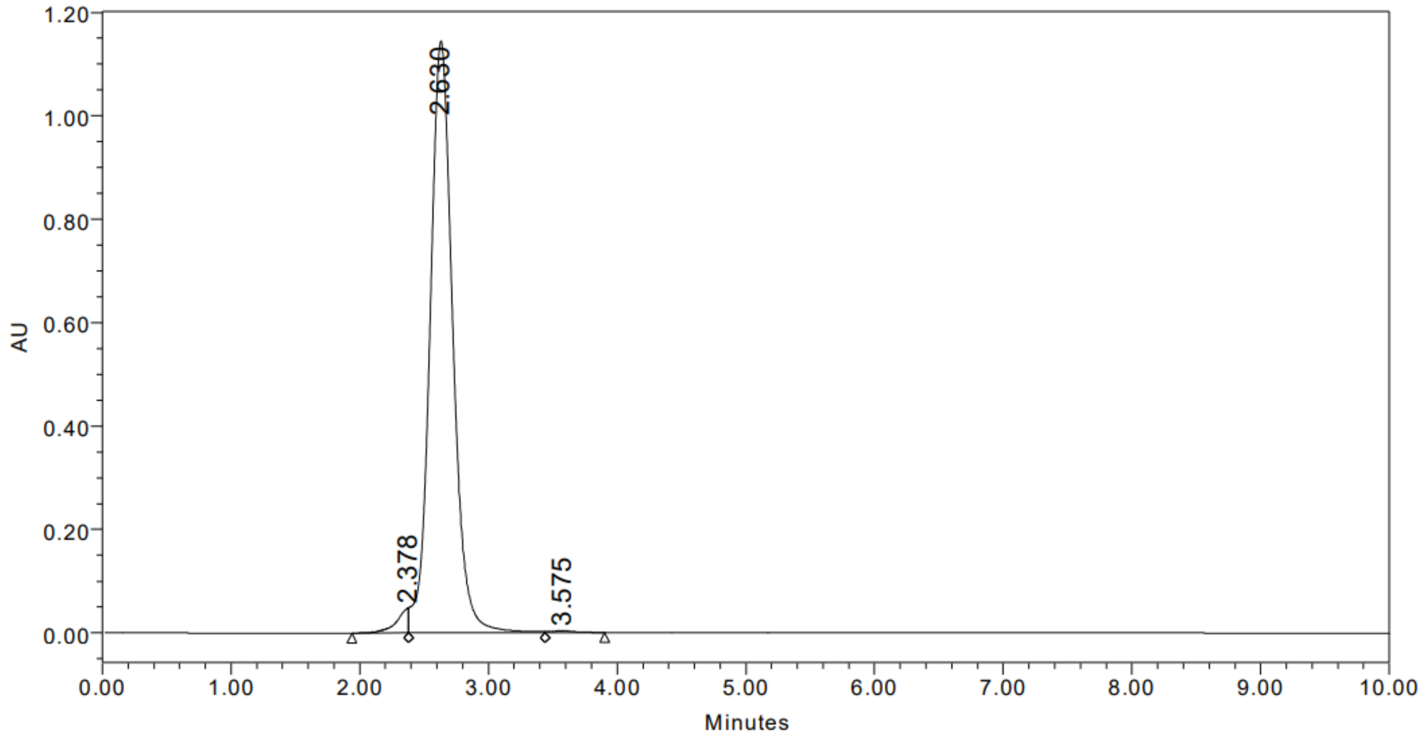

N-(1-(cyclopentylmethyl)azepan-4-yl)-N-phenyl-1H-pyrrole-2-carboxamide hydrogen chloride **(70)**

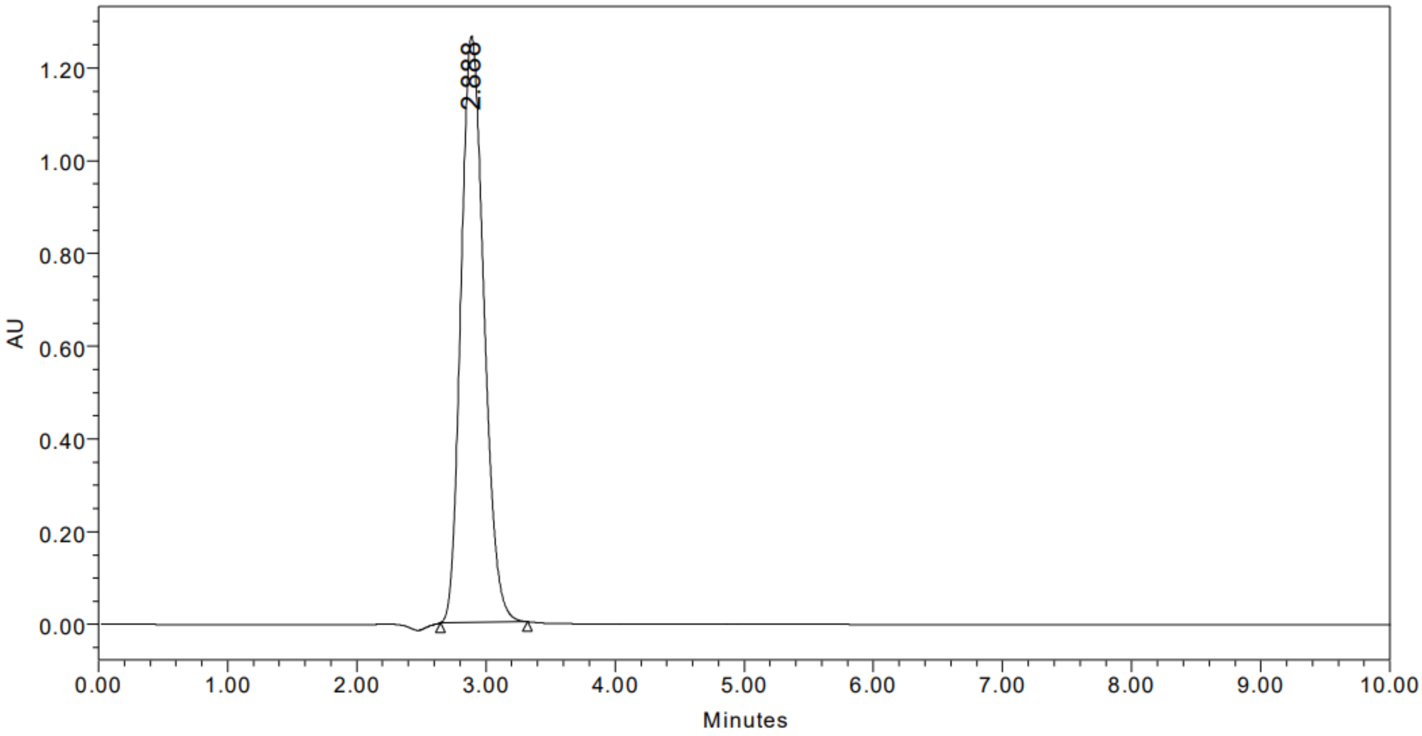

N-(1-(cyclohexylmethyl)azepan-4-yl)-N-phenyl-1H-pyrrole-2-carboxamide hydrogen chloride **(71)**

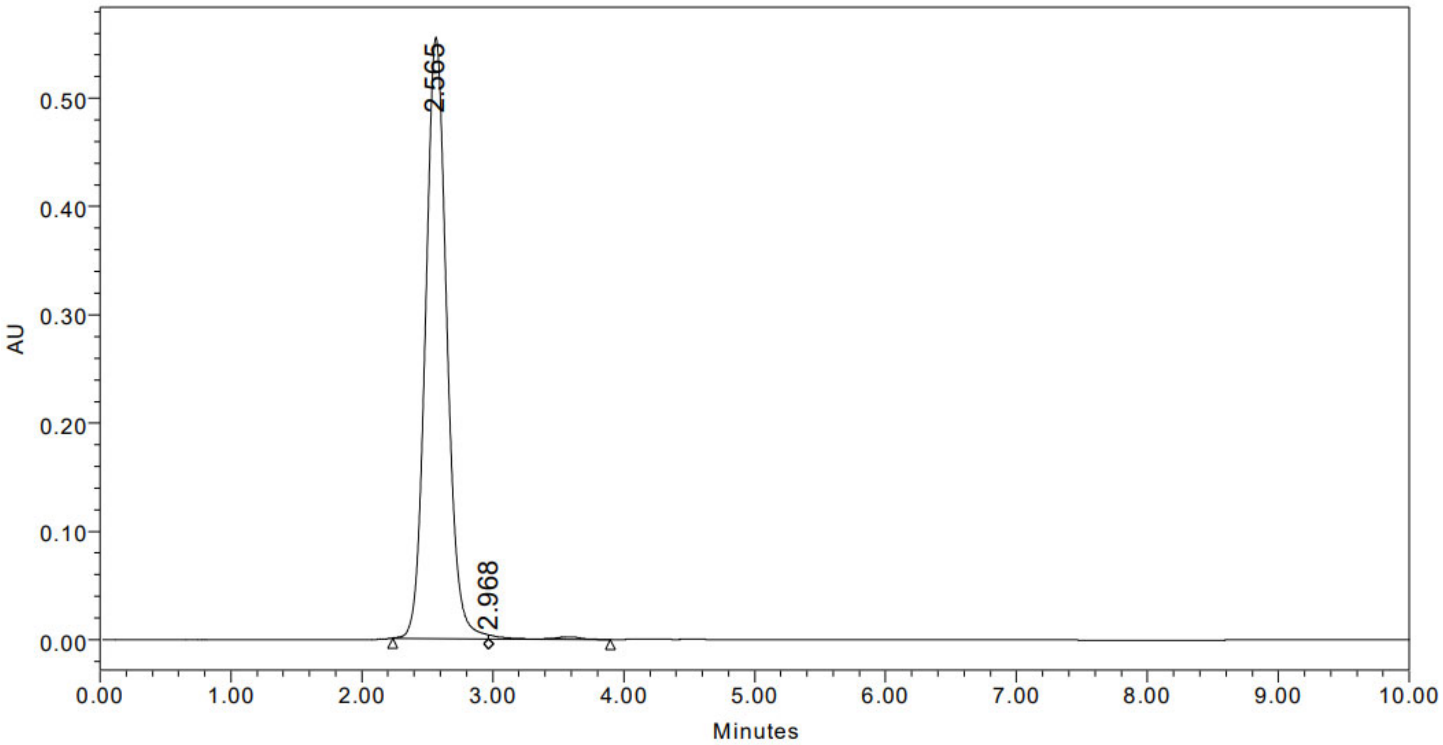

N-(1-benzylazepan-4-yl)-N-phenyl-1H-pyrrole-2-carboxamide hydrogen chloride **(72)**

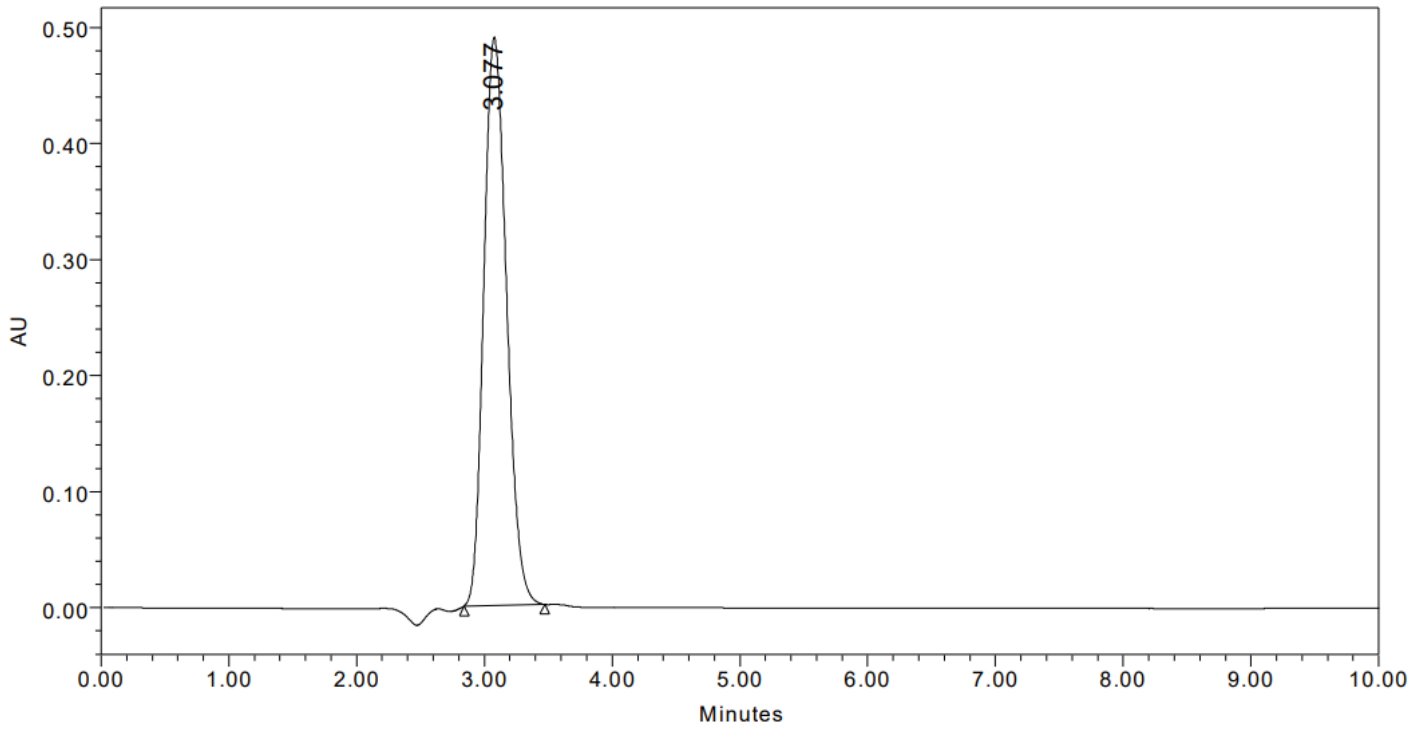

N-(1-phenethylazocan-5-yl)-N-phenyl-1H-pyrrole-2-carboxamide hydrogen chloride **(73)**

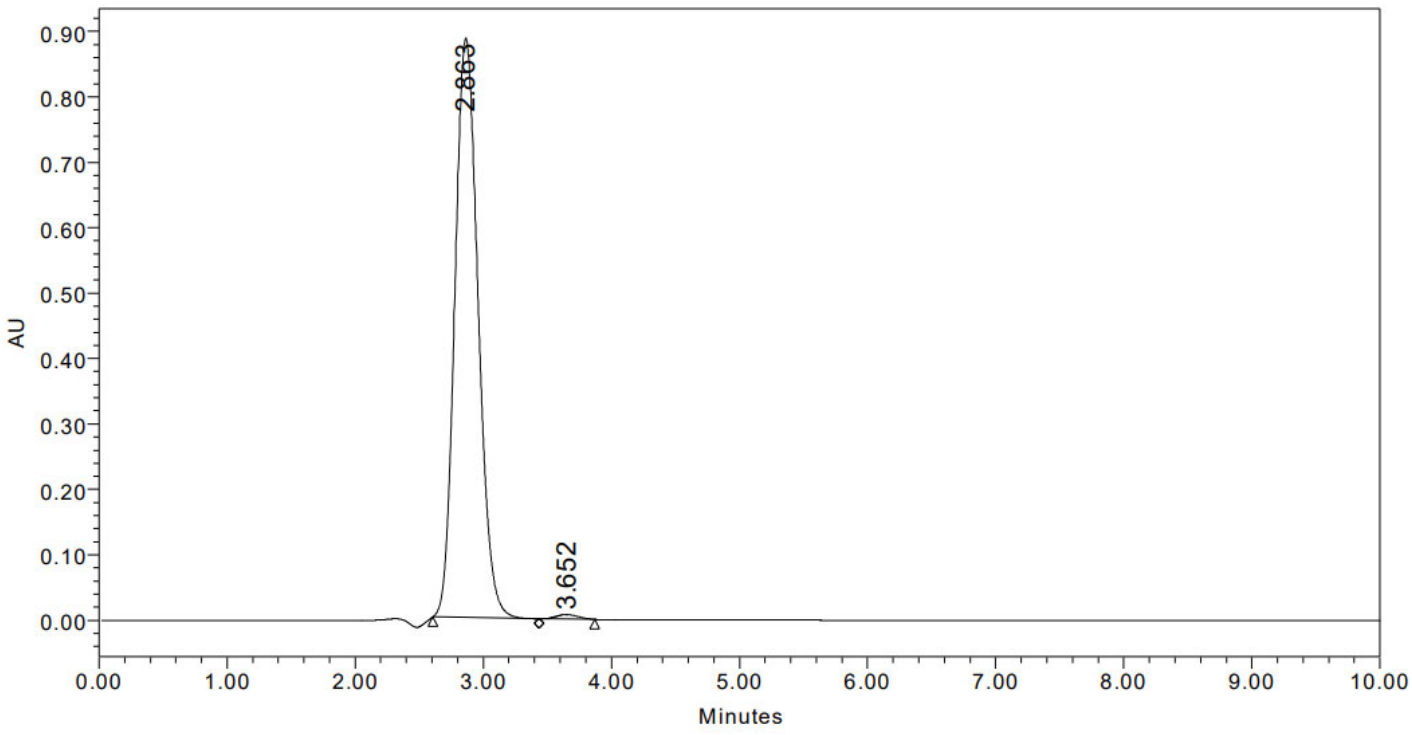

N-(1-allylazocan-5-yl)-N-phenylthiophene-3-carboxamide hydrochloride **(74)**

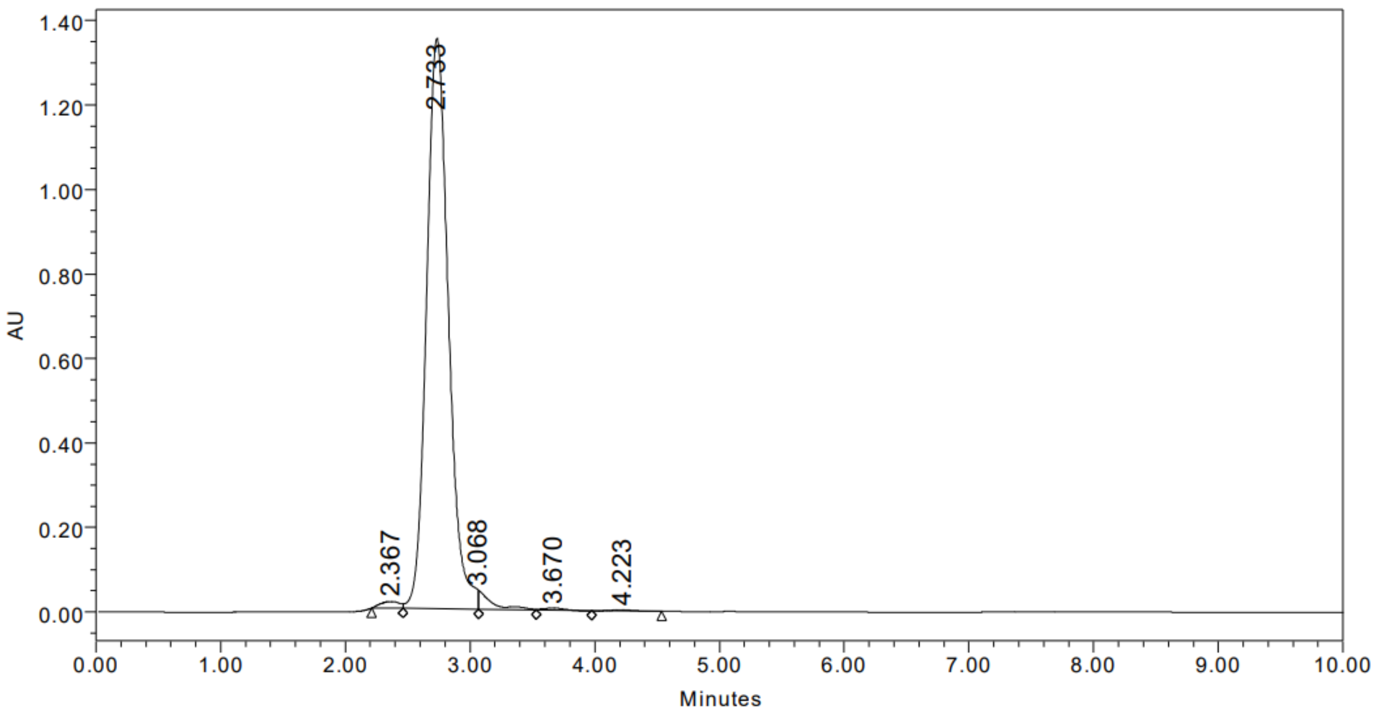

N-(1-(cyclopropylmethyl)azocan-5-yl)-N-phenylthiophene-3-carboxamide hydrogen chloride **(75)**

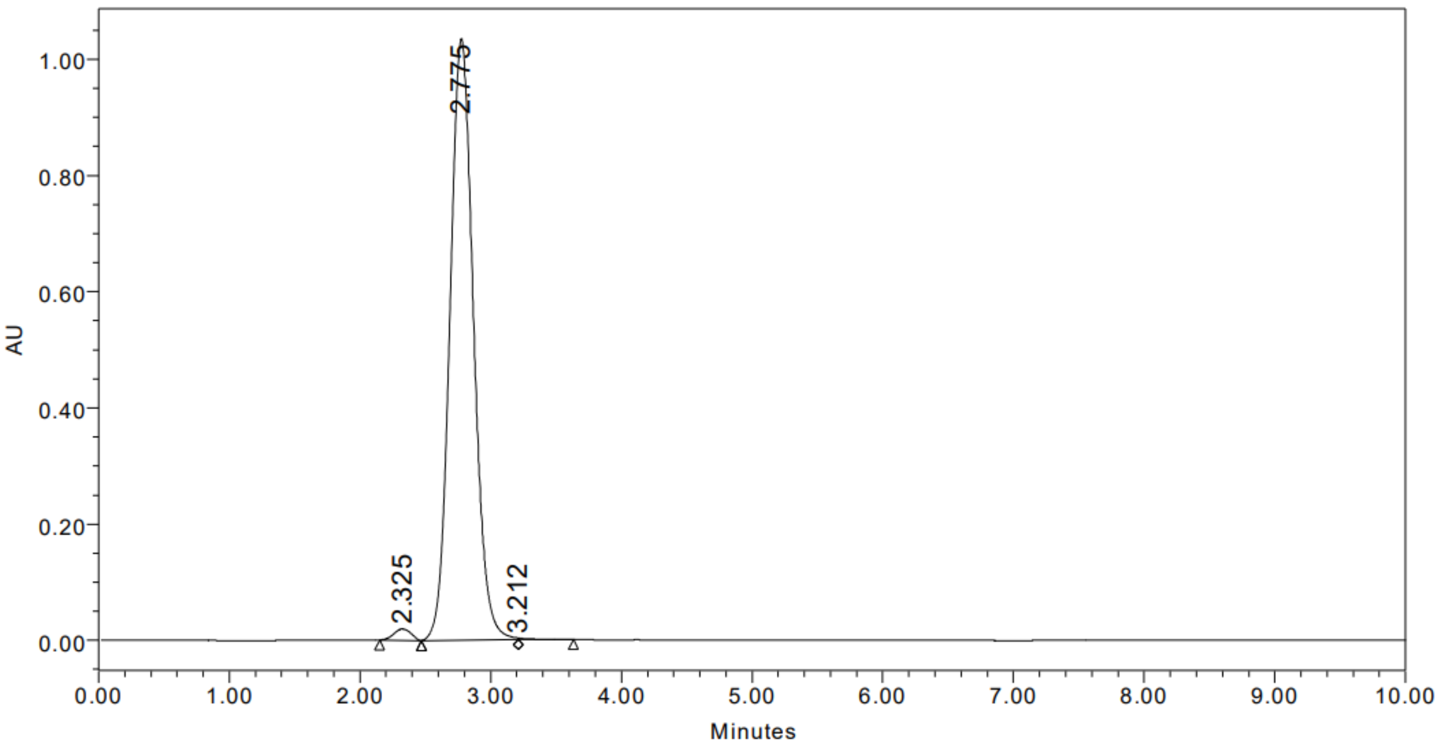

N-(1-(cyclobutylmethyl)azocan-5-yl)-N-phenylthiophene-3-carboxamide hydrogen chloride **(76)**

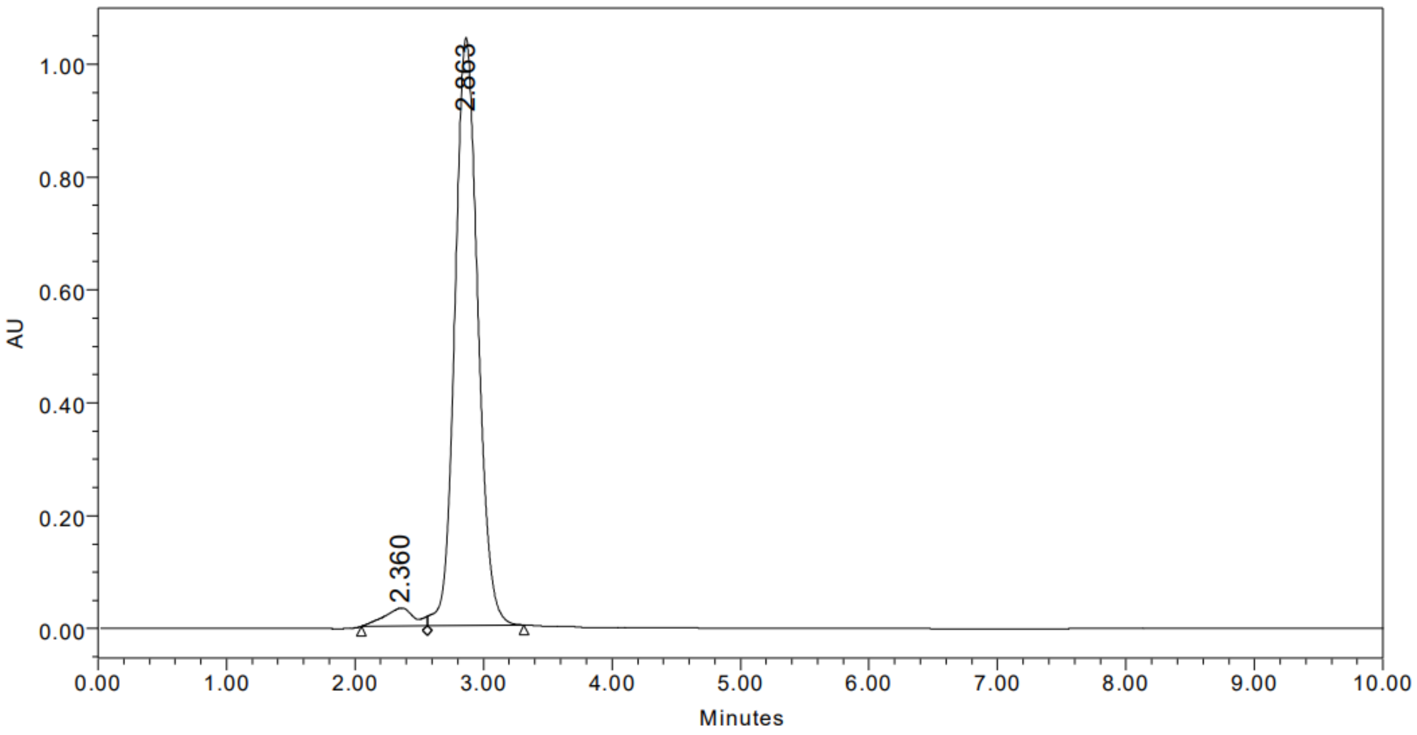

N-(1-(cyclopentylmethyl)azocan-5-yl)-N-phenylthiophene-3-carboxamide hydrogen chloride **(77)**

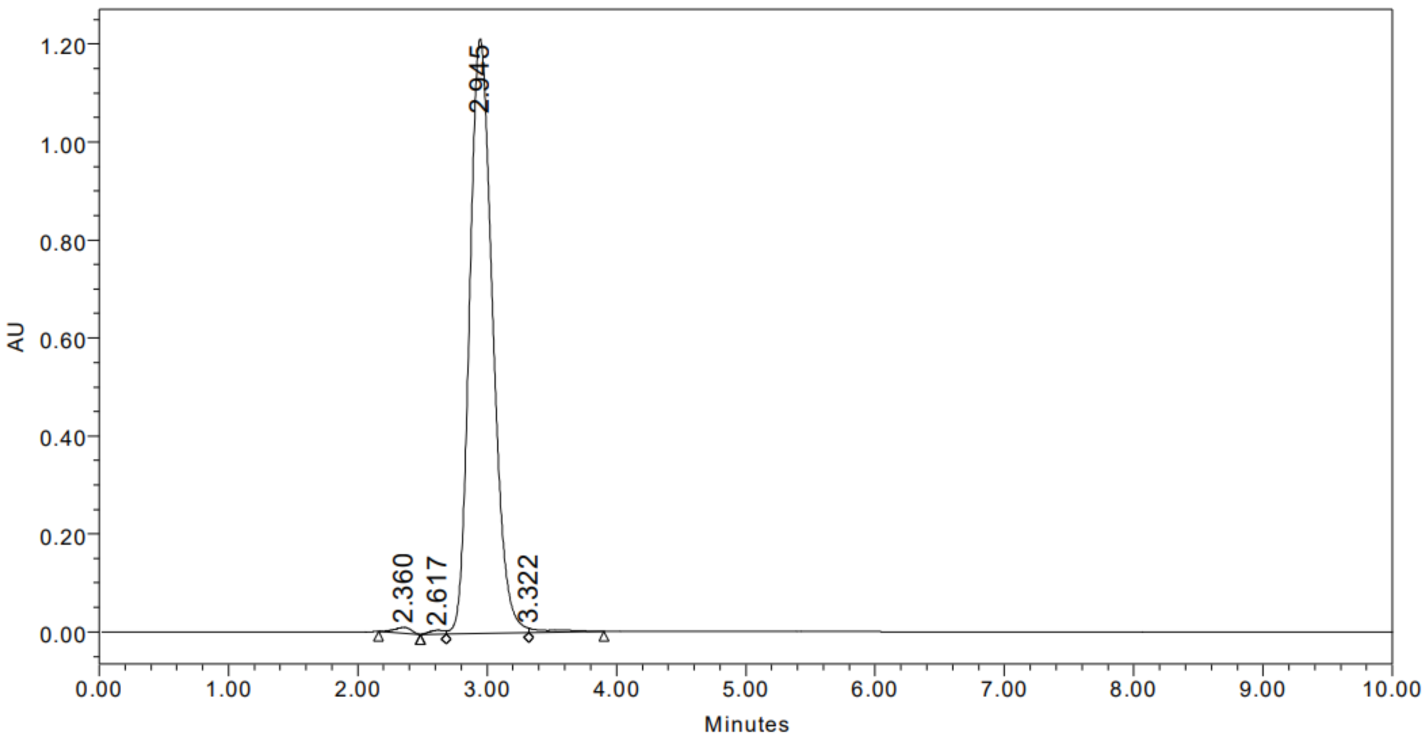

N-(1-(cyclohexylmethyl)azocan-5-yl)-N-phenylthiophene-3-carboxamide hydrogen chloride **(78)**

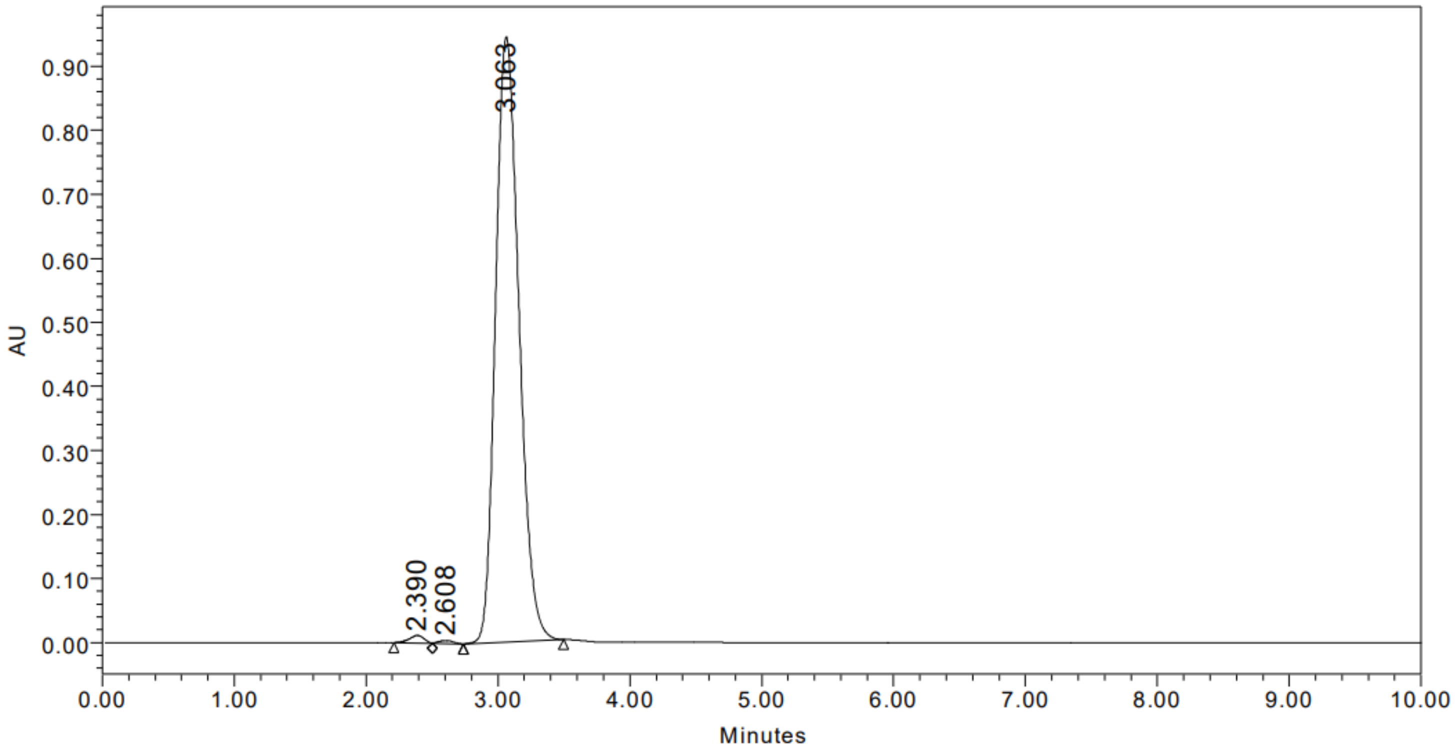

N-(1-benzylazocan-5-yl)-N-phenylthiophene-3-carboxamide hydrogen chloride **(79)**

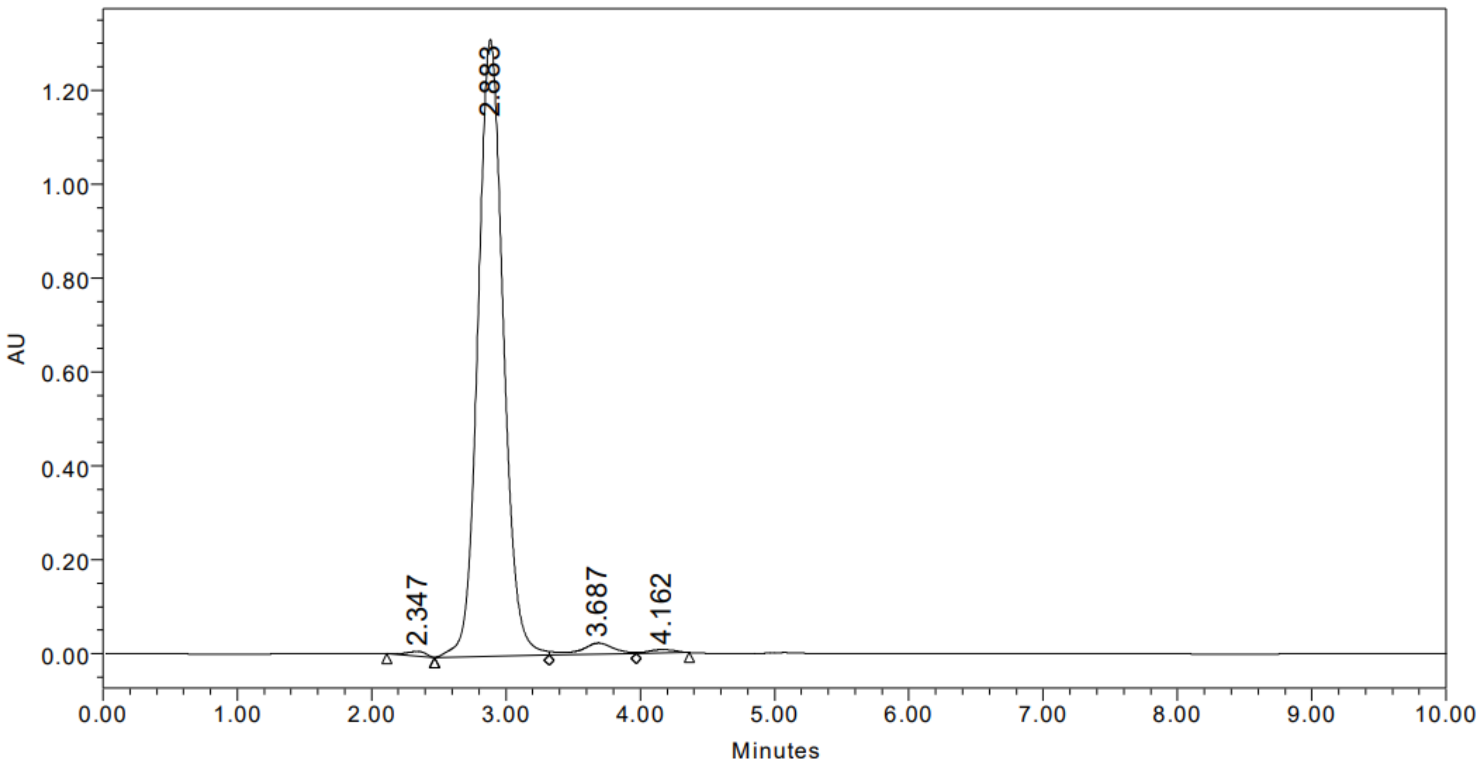

N-(1-phenethylazocan-5-yl)-N-phenylthiophene-3-carboxamide hydrogen chloride **(80)**

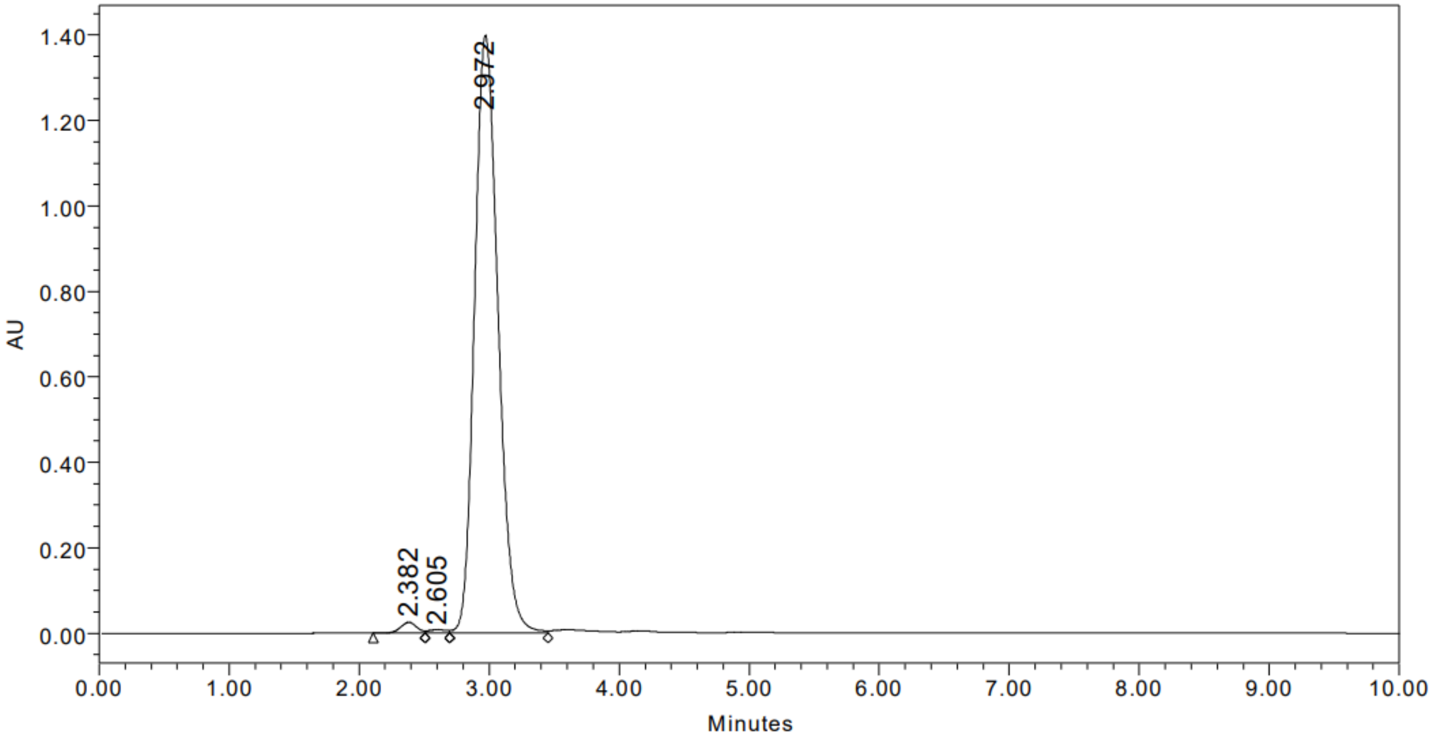

N-(1-allylazocan-5-yl)-N-phenyl-1H-pyrrole-3-carboxamide hydrogen chloride **(81)**

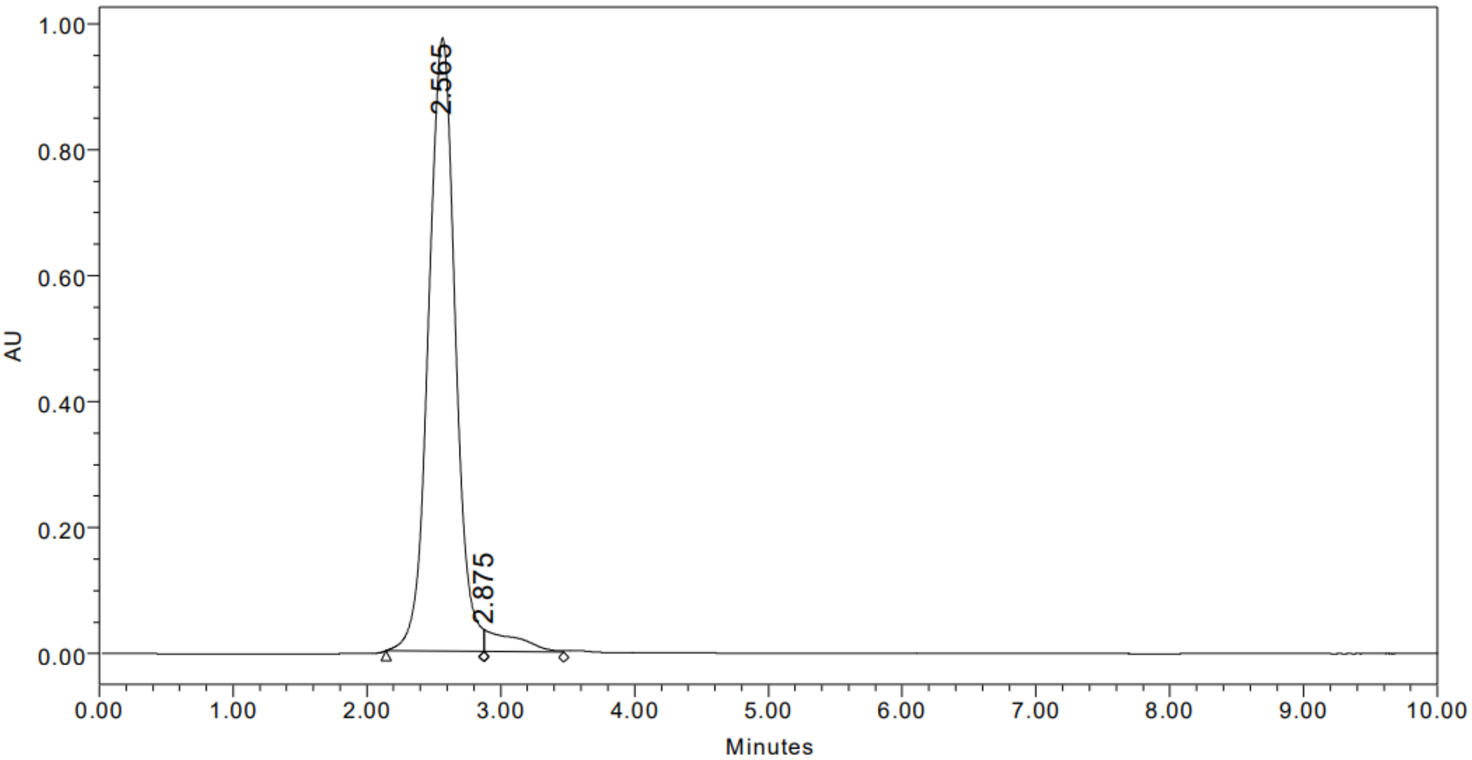

N-(1-(cyclopropylmethyl)azocan-5-yl)-N-phenyl-1H-pyrrole-3-carboxamide hydrogen chloride **(82)**

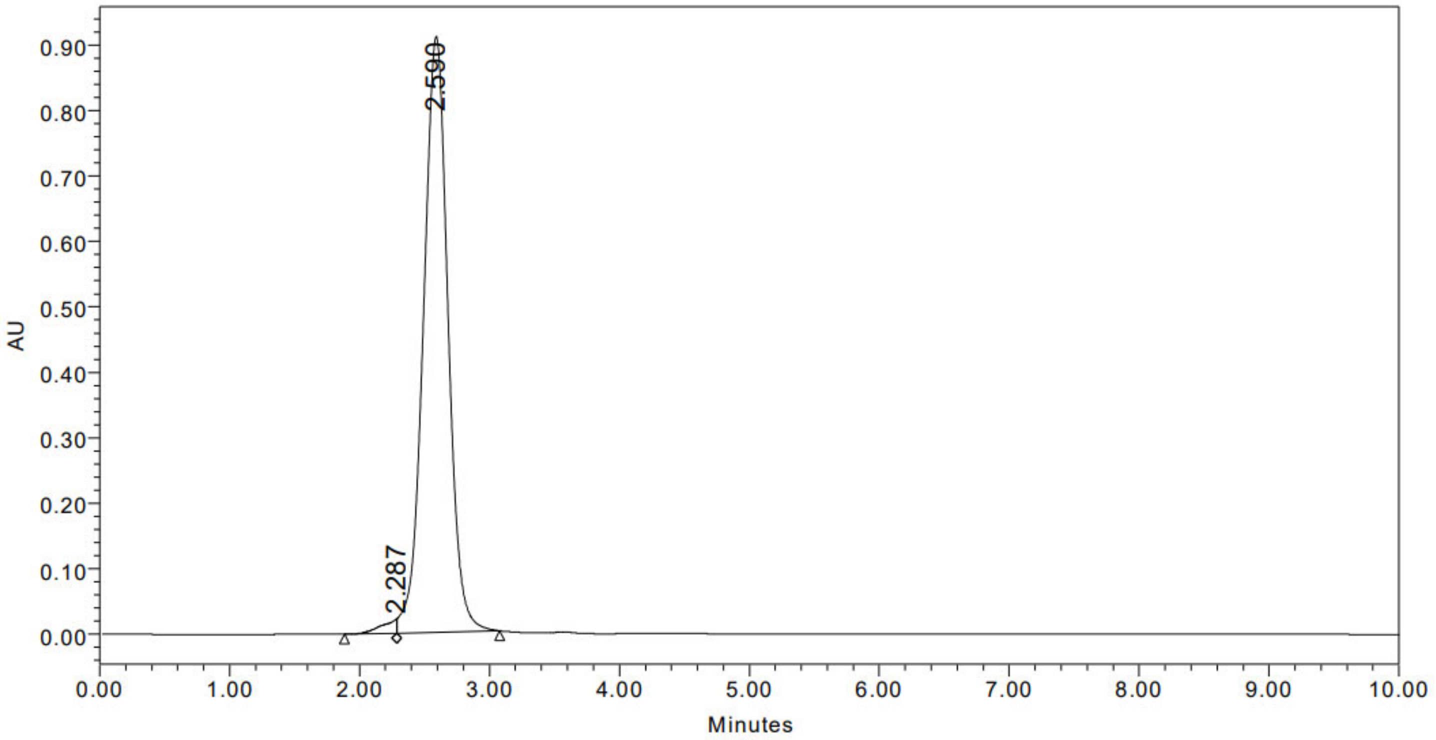

N-(1-(cyclobutylmethyl)azocan-5-yl)-N-phenyl-1H-pyrrole-3-carboxamide hydrogen chloride **(83)**

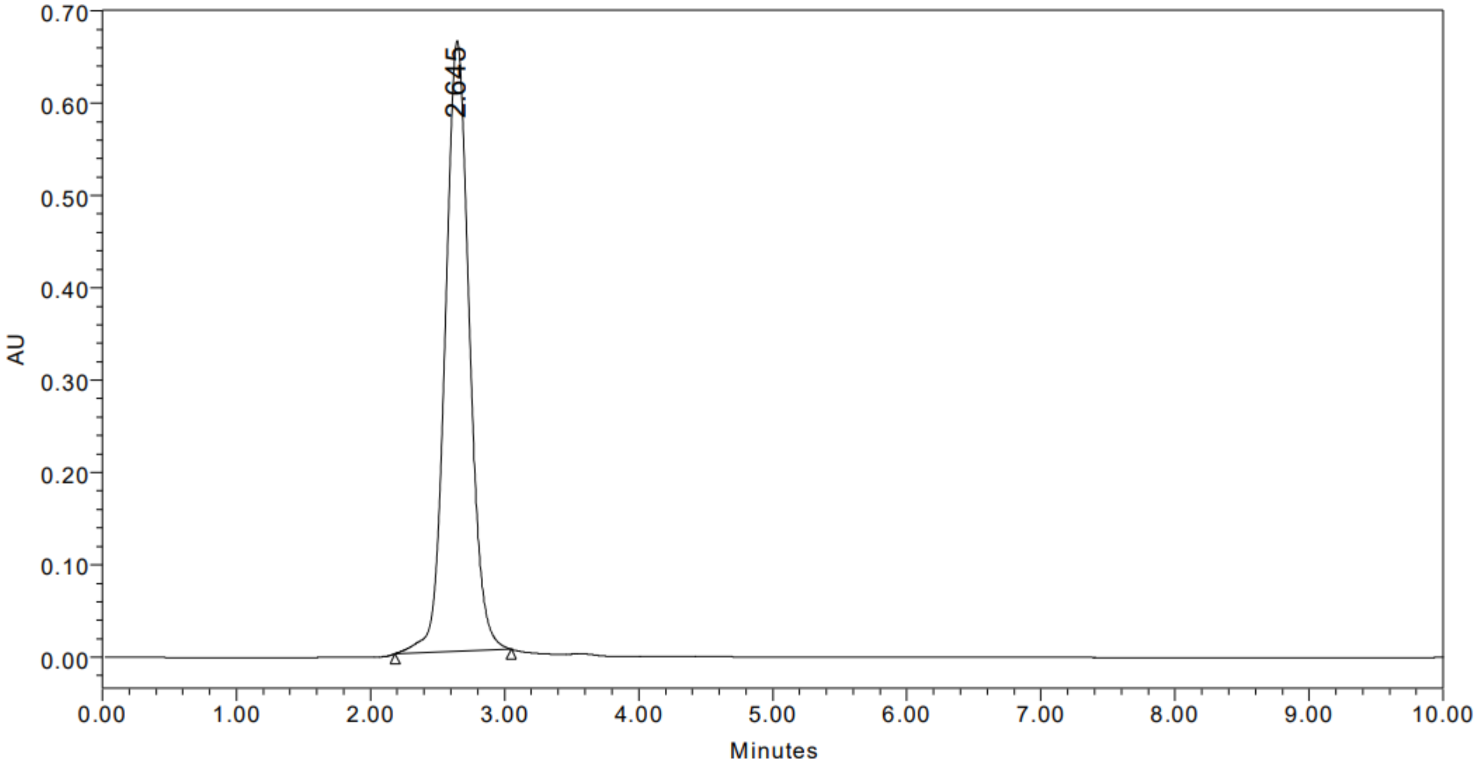

N-(1-(cyclopentylmethyl)azocan-5-yl)-N-phenyl-1H-pyrrole-3-carboxamide hydrogen chloride **(84)**

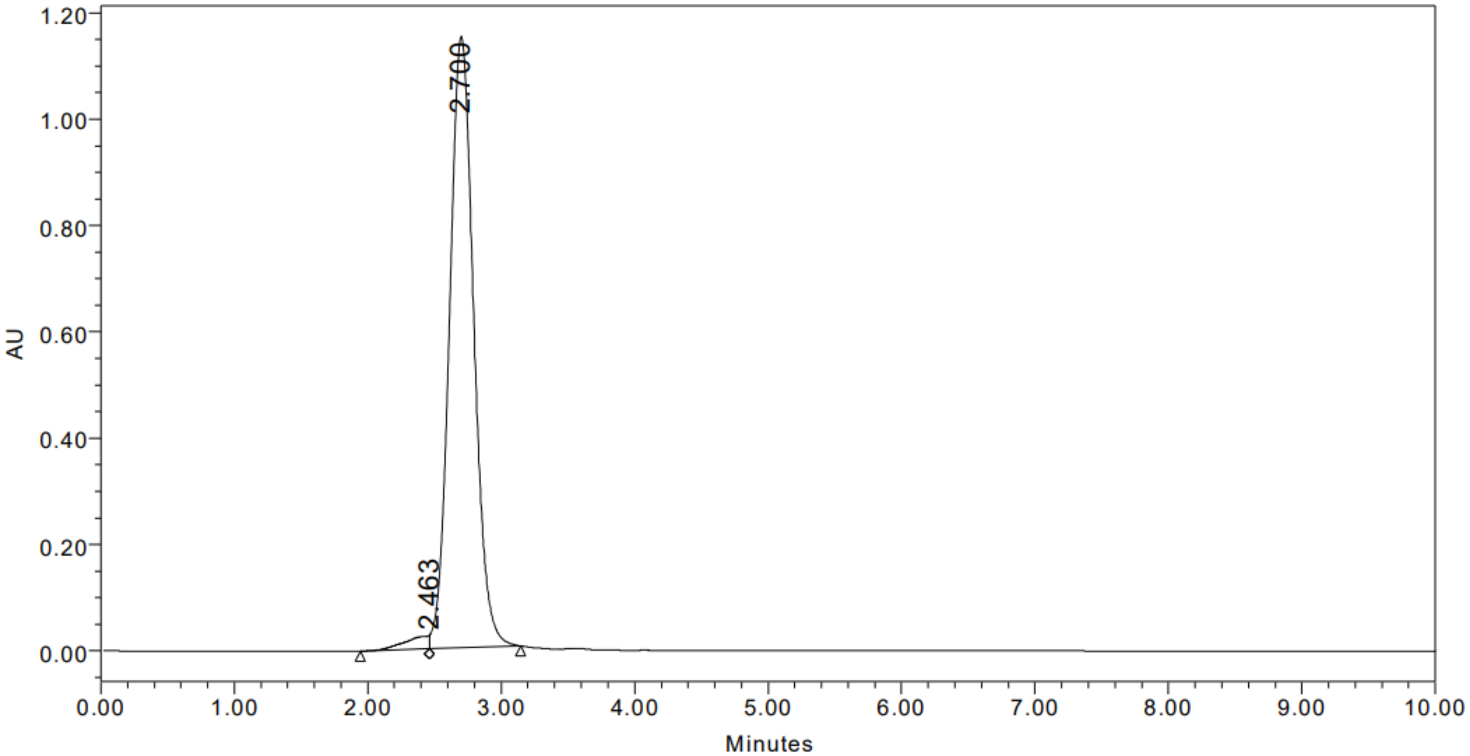

N-(1-(cyclohexylmethyl)azocan-5-yl)-N-phenyl-1H-pyrrole-3-carboxamide hydrogen chloride **(85)**

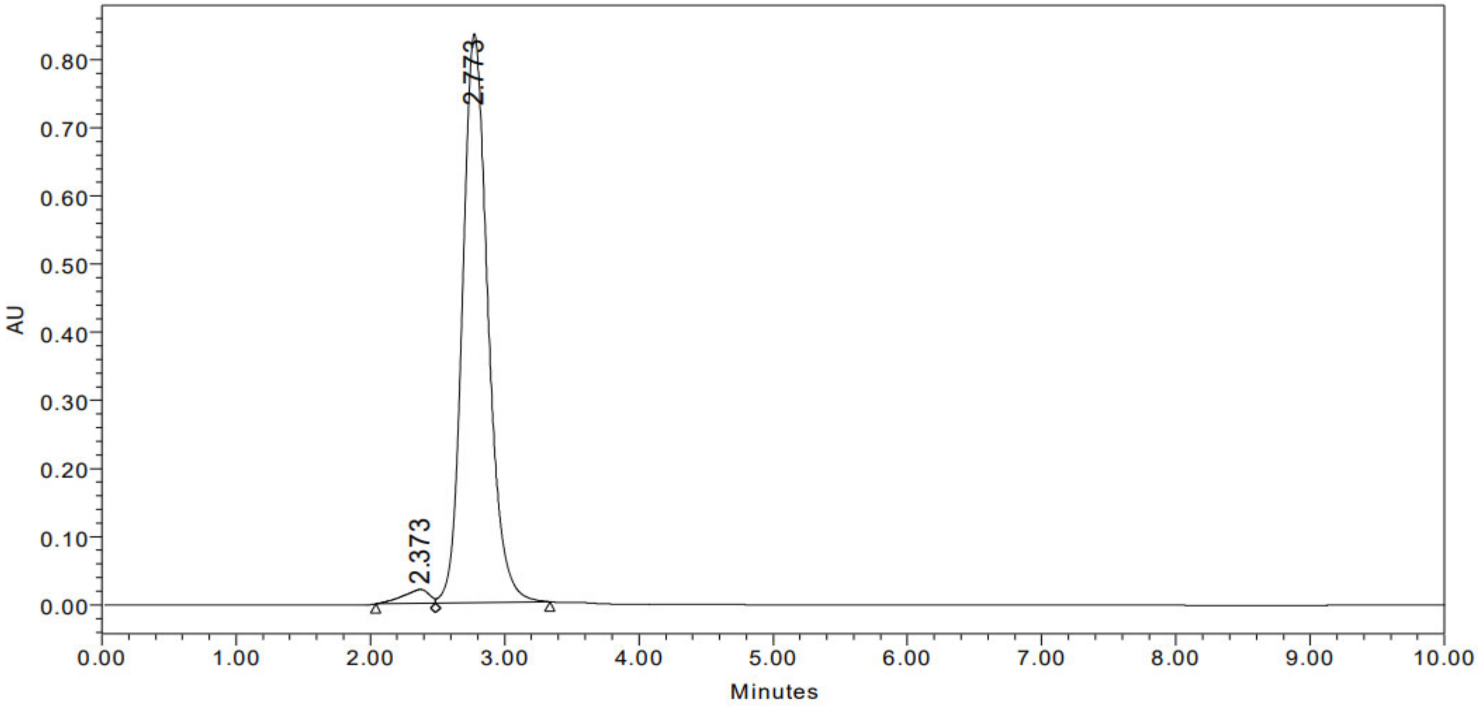

N-(1-benzylazocan-5-yl)-N-phenyl-1H-pyrrole-3-carboxamide hydrogen chloride **(86)**

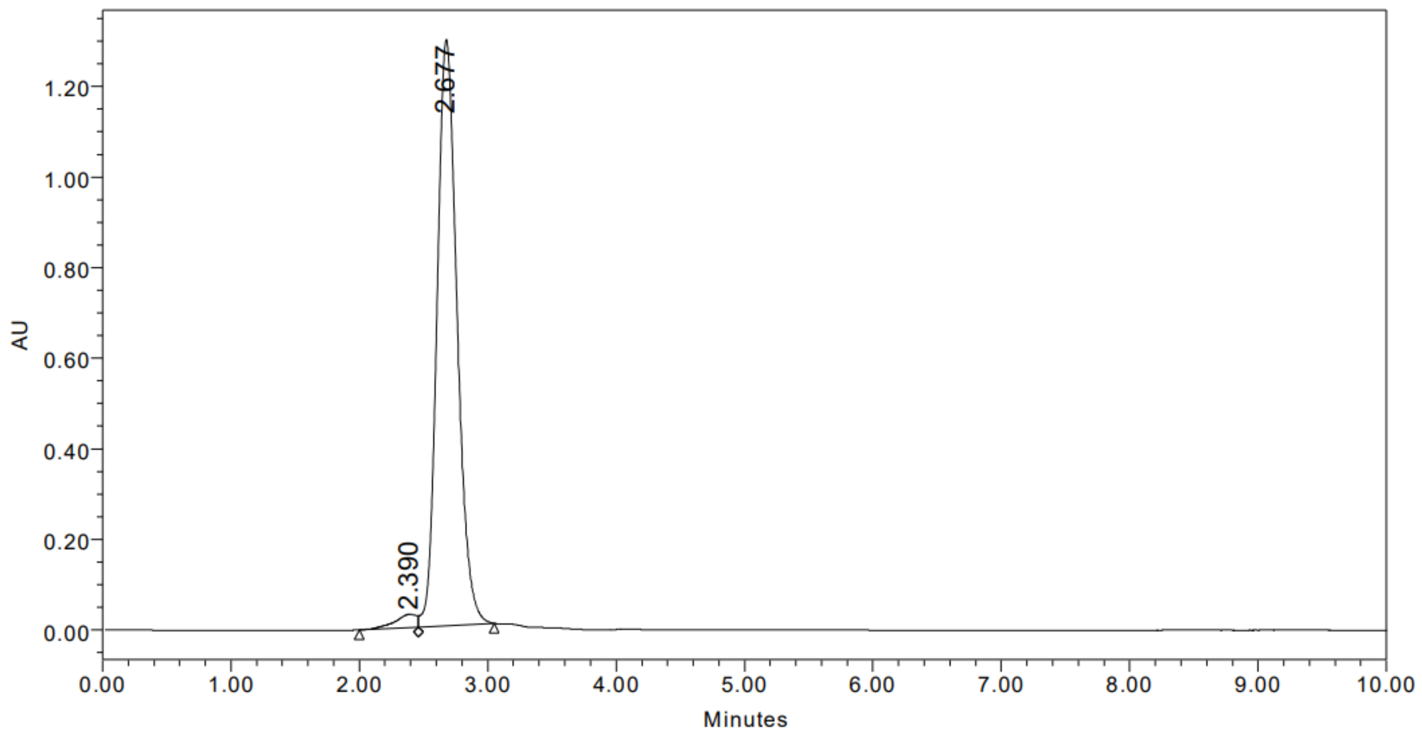

N-(1-phenethylazocan-5-yl)-N-phenyl-1H-pyrrole-3-carboxamide hydrogen chloride **(87)**

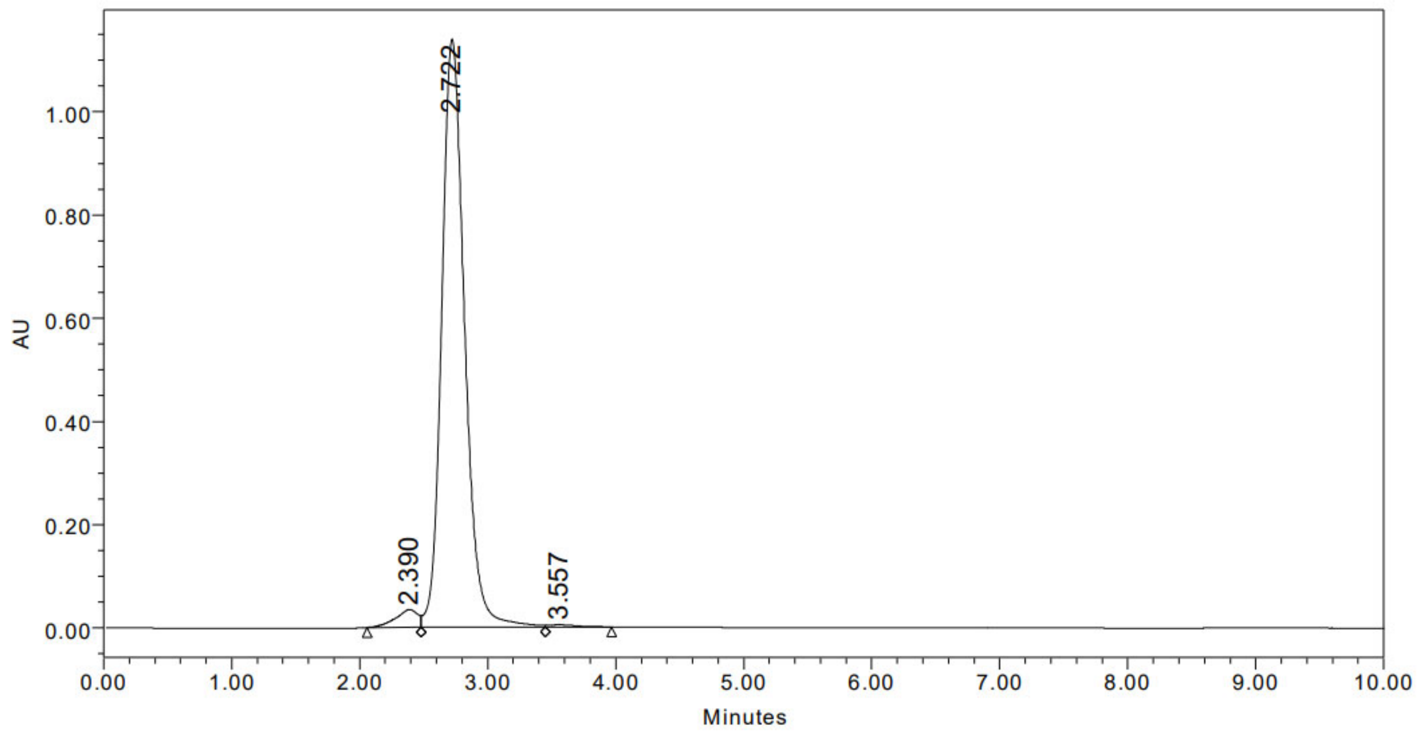

Supplement: Supplementary file 3 [file jm5c00528_si_003.pdf]
